# Viability of the Cocapture of CO_2_ and Impurities from Oxy-Fuel Combustion and Other Processes in Carbon Capture and Storage Technology

**DOI:** 10.1021/acs.energyfuels.4c04818

**Published:** 2025-06-02

**Authors:** Héctor Almazán, Javier Fernández, Sofía T. Blanco

**Affiliations:** † Departamento de Química Física, Facultad de Ciencias, 16765 Universidad de Zaragoza, 50009 Zaragoza, Spain; ‡ Instituto de Investigación en Ingeniería de Aragón (I3A), Universidad de Zaragoza, Mariano Esquillor s/n, 50018 Zaragoza, Spain

## Abstract

The feasibility of cocapturing CO_2_ with SO_2_, CO, and (O_2_ or NO) from unpurified flue gas produced by oxy-fuel combustion and other processes was assessed by determining the influence of the simultaneous presence of these impurities on selected carbon capture and storage (CCS) operational parameters. These parameters were calculated based on experimental results obtained under CCS conditions. The density, vapor–liquid equilibrium, and speed of sound of [CO_2_ + 3.0038 mol % O_2_ + 0.09035 mol % SO_2_ + 0.17032 mol % CO] and [CO_2_ + 0.1410 mol % NO + 0.09100 mol % SO_2_ + 0.17002 mol % CO] mixtures were experimentally determined at temperatures between 263 and 373 K and pressures of up to 30 MPa for density and 190 MPa for speed of sound. Joule–Thomson coefficients and isentropic compressibilities of the mixtures were calculated from our experimental results. Using experimental and calculated data, we assessed the predictive capability of the EOS-CG, GERG-2008, and PC-SAFT equations of state. The simultaneous presence of the investigated impurities at the studied concentrations adversely affects the transport and storage steps in CCS; however, the behavior of the NO-containing mixture is very similar to that of pure CO_2_. The implications of the chemical effects of the impurities were overlooked.

## Graphical abstract

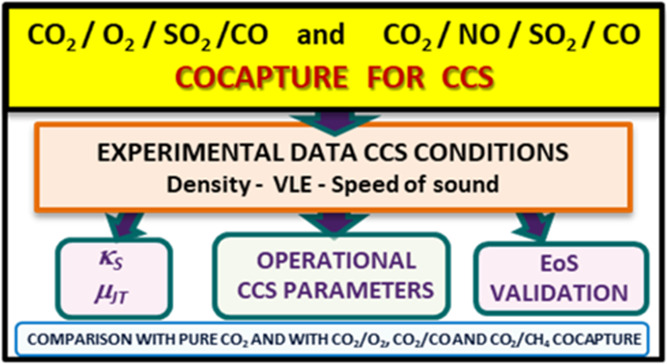

## 1 Introduction

Despite the strong alerts about climate change,^1–5^ international agreements to fight it are clearly insufficient,^6,7^ and investment in fossil fuels continues.^8^ In the current global geopolitical scenario, it seems clear that the use of fossil fuels will not be abandoned in the near future. Therefore, developing new, greener fuels and strengthening carbon capture and storage (CCS) technologies may be indispensable tools to avoid atmospheric emissions and fight climate change.

This study is part of a broader project with the overarching goal of identifying the optimal conditions for integrating the oxy-fuel combustion of biomass, either pure or blended with coal, combined with CCS technology (bioenergy CCS, BECCS, or bio-CCS processes), into power production.^9^ In oxy-fuel combustion, oxygen diluted with recycled flue gas oxidizes the fuel, enriching the exhaust gas in CO_2_, which facilitates its capture and subsequent liquefaction.^10,11^ Typically, carbon dioxide is compressed and separated from impurities to produce a stream suitable for storage. In this study, we evaluate the feasibility of bypassing the separation process for CO_2_ by employing CO_2_/impurity cocapture, as suggested in the literature.^12–20^ Cocapture would simplify oxy-fuel combustion processes since avoiding impurity separation saves costs, and moreover, the presence of these substances might improve postcapture stages. Additionally, cocapture prevents the emission of not only CO_2_ but also impurities, which is especially advantageous for toxic pollutants such as NO, SO_2_, and CO. Specifically, this study assesses the influence of the simultaneous presence of a condensable impurity (i.e., SO_2_) and two noncondensable impurities (i.e., CO and O_2_ or CO and NO) on the properties of flue gas produced by the oxy-fuel combustion of biomass (pure or blended with coal), which is captured without further purification (CO_2_/impurity cocapture) for subsequent transport and storage in CCS technology.^21^ To achieve this, two quaternary mixtures were experimentally studied, namely, CO_2_ + 3.0038 mol % O_2_ + 0.09035 mol % SO_2_ + 0.17032 mol % CO and CO_2_ + 0.1410 mol % NO + 0.09100 mol % SO_2_ + 0.17002 mol % CO, with concentrations of impurities characteristic of the above processes. Additionally, the NO-containing mixture models emissions without purification from other processes, such as gas engine combustion.^22^ According to previous studies, postcombustion CO_2_ capture is the preferred method for internal combustion engines (ICEs). Researchers such as Wang et al.^23^ have explored the use of amine absorption, temperature swing adsorption (TSA), cryogenics, and membrane technologies in ICEs installed on ships. Similarly, for road transport vehicles, post-combustion capture technologies have been identified as the most easily adaptable, with amine absorption and TSA emerging as the most promising methods.^24^ Avoiding the subsequent purification stage through cocapture would significantly simplify the design and complexity of capture systems, which is particularly useful for nonstationary engines. However, anthropogenic CO_2_ impurities can strongly alter fluid properties such as density (ρ), vapor–liquid equilibrium (VLE), speed of sound (*c*), and viscosity (η), impacting pipeline hydraulics and CCS technology design.^12,25,26^ Understanding the properties of the impure stream is crucial for optimizing the operation of CCS facilities and assessing the feasibility of the CO_2_/impurity cocapture.

As part of an ongoing research line, the authors systematically determined experimental values of thermodynamic properties (ρ, VLE, and *c*) for binary CO_2_ + impurity systems and evaluated the influence of the studied impurity on CCS technology, considering both the noncondensable impurities addressed in this study (O_2_, CO, and NO)^27–29^ and the condensable impurity SO_2_,^30–32^ as well as noncondensable CH_4_.^27,28,33^ Furthermore, the authors analyzed the influence of CO or CH_4_ on CO_2_/SO_2_ cocapture processes.^34,35^ Experimental determinations of thermodynamic properties of CO_2_ + impurity systems have also been conducted by other researchers,^15,36–45^ but only a few have applied their results to CCS technology.^15,46^ Nonetheless, no published work has experimentally quantified the combined influence of multiple impurities present in unpurified emissions from oxy-fuel combustion or gas engines on thermodynamic properties, CCS technology, or even cocapture processes. Thus, this study takes a novel approach by addressing the influence of real emission compositions, along with temperature and pressure, on CCS processes, including cocapture processes, using experimental thermodynamic results. This is particularly important since combustion emissions typically contain multiple impurities of differing natures, which may exert opposing effects.

Pressure–density–temperature (*p*–ρ–*T*), bubble pressure (*p*_bubble_), dew pressure (*p*_dew_), densities of liquid and vapor phases (ρ_L_ and ρ_V_, respectively) in the VLE, and pressure–speed of sound–temperature (*p*–*c*–*T*) data were experimentally determined for the studied mixtures in the *T* range of 263.15–373.15 K and a *p* of up to 30 MPa for densities and VLE and *p* of up to 190 MPa for the speeds of sound. These conditions cover the relevant ranges for CCS steps such as transport, injection, and storage.^25^ The speeds of sound were measured by adding methanol to the mixtures because of the acoustic opacity of CO_2_-rich mixtures at an operating frequency of 5 MHz, a technique previously tested by the authors.^29,30^ Even with methanol doping, the acoustic signals of CO_2_-rich mixtures could not be detected within the low-pressure range pertinent to CCS. Consequently, experimental data were extended to lower pressures via extrapolation, and the extrapolated values were validated using the GERG-2008 ^47^ and PC-SAFT^48^ equations of state (EoSs; Section 3.2).

In addition, from our experimental data, we calculated values of the isentropic compressibility (κ_S_) and Joule–Thomson coefficient (μ_JT_) for the two quaternary mixtures at the studied temperatures and at pressures higher than 5 MPa. κ_S_ is necessary to predict how the fluid will behave during compression for transport and storage,^49–51^ and μ_JT_ describes the thermal behavior of the fluid under depressurization.^52–54^

Because there is currently no established EoS deemed optimal for CCS technology, we utilized our experimental and calculated thermodynamic data to assess different EoSs. The evaluated EoSs include EOS-CG (an EoS for combustion gases specifically designed for CO_2_-rich mixtures relevant to CCS applications),^55^ GERG-2008 (the Groupe Européen de Recherches Gazières model upon which the former is built),^47^ and PC-SAFT (the perturbed-chain statistical associating fluid theory EoS that is widely applied in the engineering field).^48^

Finally, from our experimental thermodynamic data and calculated viscosities, we obtained values for selected technical parameters related to the transport, injection, and storage stages of CCS technology for the investigated mixtures (minimum operational pressure, pressure and density drop along the pipeline, inner diameter of the pipeline, normalized storage capacity, normalized velocity of the rising plume in saline aquifers, and normalized permeation flux). By comparing these values with those calculated for pure CO_2_,^56^ we determined the impact of the impurities in the studied mixtures on CCS technology. The technical storage parameters were also calculated for different actual saline aquifers utilized in CCS (Table 1).

**Table 1:** Conditions of the Saline Aquifers Investigated in This Study^a^

reservoir	sleipner	nagaoka	frio	nisku Fm. #1	deadwood Fm. #2	basal Cambrian Fm.	snøhvit
*p*/MPa	10.3	11.9	15.2	17.4	23.6	27.0	29.0
*T*/K	317	319	329	329	338	348	373
depth/m	1000	1100	1546	2050	2560	2734	2600
ρ_br_/kg m^–3^	1017	999	1048	1076	1009	1137	1090
references	( ^57,58^ )	( ^57,58^ )	( ^57,58^ )	( ^58,59^ )	( ^58,60^ )	( ^58,59^ )	( ^58–63^ )

a ρ_br_ is the density of the brine.

Additionally, we compared the results obtained for the O_2_-containing quaternary mixture to those of the binary mixtures CO_2_ + O_2_, CO_2_ + CO, and CO_2_ + CH_4_ with noncondensable impurities concentrations similar to that of O_2_ in the quaternary mixture, obtained previously by the authors.^27–29,33^

This study investigates the reduction in CO_2_ emissions by assessing the viability of transporting and storing CO_2_-rich flue gases containing impurities found in unpurified flue gas produced by oxy-fuel combustion and other processes. It explores CO_2_/SO_2_–CO–O_2_ or CO_2_/SO_2_–CO–NO cotransport, coinjection, and costorage within CCS technology to reduce purification costs. To achieve this, two CO_2_-rich mixtures with SO_2_, CO, and (O_2_ or NO) were thermodynamically characterized. The presented findings are essential for advancing CCS technology and its role in mitigating climate change.

## 2 Experimental Section

### 2.1 Materials

Table 2 compiles the compositions of the mixtures (Mix 1 and Mix 2) investigated in this study, both of which were provided by Carburos Metálicos (Air Products Group).

**Table 2:** Compositions (mol %) of the Studied Quaternary Mixtures and Expanded Uncertainties (% rel., Coverage Factor *k* = 2) in Parentheses^a^

components	Mix 1	Mix 2
CO_2_	96.734 (±0.05)	99.592 (±0.05)
O_2_	3.0038 (±0.2)	NA
NO	NA	0.1410 (±2)*
SO_2_	0.09035 (±0.5)	0.09100 (±0.5)
CO	0.17032 (±0.5)	0.17002 (±0.5)
analysis method	gravimetric	gravimetric *analytic

a Conformed to international standard ISO 6141:2015 (ISO 6141). Gas analysis-contents of certificates for calibration gas mixtures^64^ according to the manufacturer.

To measure the speed of sound, the quaternary mixtures were doped with methanol (biotech grade, 99.996% purity according to gas chromatography) supplied by Sigma-Aldrich, which was used immediately after degassing.

### 2.2 Apparatus and Methods

In the experimental phase of our research, there were several hazards to consider, primarily related to the high pressures generated within the equipment and the reactivity and toxicity of the impurities being studied. O_2_ is a potent oxidizing gas, and NO is a toxic gas that rapidly reacts with air to form NO_2_, which is also toxic. The short-term exposure limit of NO_2_ and SO_2_ is 5 ppm, and that of CO is 100 ppm.^65^ To mitigate the risks associated with our experimental work, we adopted several safety measures. First, we evacuated experimental apparatuses under vacuum for at least 2 h before introducing the studied mixtures. We also employed leak detectors to identify and address any gas leaks that might occur once fluids were introduced into the equipment. Furthermore, to safeguard personnel from accidental exposure, we placed mobile transparent polycarbonate barriers around the experimental facilities.

The combined standard uncertainty values for the experimental data obtained in this work are calculated according to the “Evaluation of Measurement Data–Guide to the Expression of Uncertainty in Measurement (GUM)”,^66^ as suggested by the National Institute of Standards and Technology.

The quaternary mixtures in Table 2 were used for the density and VLE measurements. The setup, provided by ARMINES (Figure 1), is designed to generate precise *p*–ρ–*T* data for fluids spanning the vapor and liquid phases and supercritical state.^67^ This apparatus centers around an Anton Paar DMA HPM vibrating tube densimeter, fully integrated into the installation, which operates within the temperature range of 263–423 K and pressures of up to 70 MPa. For a comprehensive understanding of the facility and the measurement procedures, see ref 28,29,31,68 .

**Figure 1:**
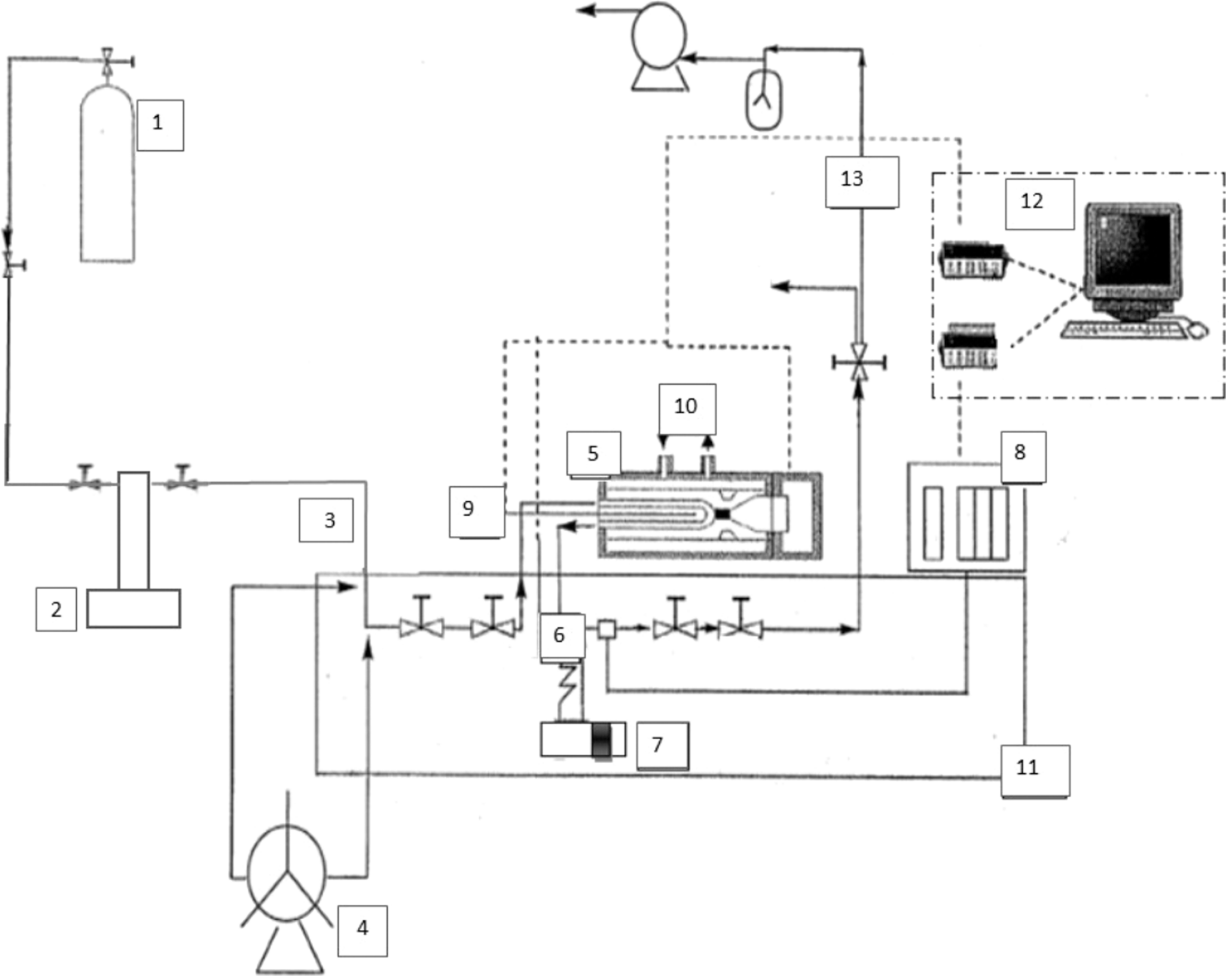
Experimental setup for volumetric measurements: (1) mixtures from Table 2; (2) ISCO pump; (3) fluid inlet; (4) manual pump; (5) densimeter; (6) vibrating tube output; (7) rupture disk; (8) thermoregulated pressure transducers; (9) platinum temperature probe; (10) connected external liquid thermoregulated bath; (11) liquid thermoregulated bath; (12) evaluation unit and data acquisition; and (13) evacuation and vacuum line. Adapted from Bouchot and Richon.^67^

The densimeter operates by determining the vibration period (τ) with an uncertainty of *u*(τ) = 2 × 10^–5^ ms, as provided by the manufacturer. The temperature of the fluid within the vibrating tube was measured using a 100 Ω platinum probe previously calibrated by the Centro Español de Metroloíga (CEM, 2000). The calculated standard uncertainty in temperature (*u*(*T*)) was 0.006 K, and the temperature variation during the measurement of the *p*–ρ–*T* isotherm remained within ±0.02 K except for the measurement of the vapor phase of Mix 1 at 263 K where the variation of *T* was ±0.05 K. Two pressure transducers were employed: one for pressures below 6 MPa and the other for pressures ranging from 6 to 70 MPa. We calibrated both transducers by using a Wika CPH 6000 calibrator. The combined standard uncertainty in the pressure (*u*(*p*)) was 0.0020 MPa for pressures below 6 and 0.024 MPa for pressures ranging from 6 to 70 MPa, as per Euramet standards.^69^ The vibrating tube was calibrated according to the forced path mechanical calibration (FMPC) model, as recommended by the device manufacturers.^70^ A comprehensive explanation of the vibrating tube calibration procedure can be found in ref 29.

The combined standard uncertainty in density (*u*(ρ)) for each experimental *p*–ρ–*T* point is provided alongside the experimental density data in Table S1, Supporting Information, and typically ranges from 0.20 to 0.40 kg m^–3^.

By applying the tangents method to the *p*–ρ–*T* data, as described in ref 31, the values for *p*_dew_, *p*_bubble_, ρ_V_, and ρ_L_ in the VLE and their respective combined standard uncertainties were obtained. The VLE data, along with their uncertainties, are presented in Table S2.

To measure the speed of sound in Mix 1 and Mix 2, mixtures were doped with methanol. This addition was necessary because the original undoped mixtures, as well as pure CO_2_, exhibited significant sound absorption at the frequency used (5 MHz), rendering them opaque to sound at this frequency. We have previously discovered that ∼1.0 mol % CH_3_OH can be added to CO_2_ to obtain usable signals over a suitable pressure range.^30^ These signals resulted in the speed of sound values that exhibited mean deviations of only 0.38% compared to pure CO_2_, a deviation lower than the tolerance margin specified by the Span and Wagner EoS under the experimental conditions, which ranged between 0.5 and 2%.^55^ Consequently, we applied the same doping method to measure the speed of sound in Mix 1 and Mix 2, both of which were doped with ∼1.0 mol % CH_3_OH. We have previously used this doping technique in other studies.^29,30^

The doped mixtures were prepared in a variable-volume cell. In the preparation process, first, degassed methanol was introduced into the variable-volume cell, and then either Mix 1 or Mix 2 was introduced. The mass of each fluid introduced into the cell was determined by measuring the difference in the cell mass before and after fluid introduction using a Sartorius CCE 2004 mass comparator with a repeatability of >0.0002 g. Then, the doped mixture was transferred to our experimental speed of sound installation. The details of the procedure are provided in ref 31. The uncertainties in the composition of the doped mixtures were calculated, and the compositions of the doped mixtures, along with their uncertainties, are shown in Table 3. The method used to calculate the uncertainties is detailed in the Supporting Information (pp S4–S7).

**Table 3:** Compositions (Mole Fractions) of the Doped Mixtures Investigated in the Speed of Sound Installation (*x*_*i*_) along with Their Respective Combined Standard Uncertainties (*u*(*x*_*i*_))

	doped Mix 1		doped Mix 2	
component	*x*_*i*_	*u*(*x*_*i*_)	*x*_*i*_	*u*(*x*_*i*_)
CO_2_	0.95769	0.00058	0.98586	0.00016
CH_3_OH	0.00999	0.00011	0.01016	0.00011
O_2_	0.02974	0.00047	NA	NA
NO	NA	NA	0.001396	0.000026
SO_2_	0.000893	0.000015	0.000899	0.000015
CO	0.001686	0.000028	0.001683	0.000027

Our speed of sound study involved the determination of *p*–*c*–*T* isotherms using a 5 MHz pulsed ultrasonic system, as described in ref 30 (Figure 2). This system can operate within the temperature range 253–473 K and at pressures ranging from 0.1 to 200 MPa, with uncertainties of *u*(*T*) = 0.015 K and *u*(*p*) = 0.02 MPa, respectively.

**Figure 2:**
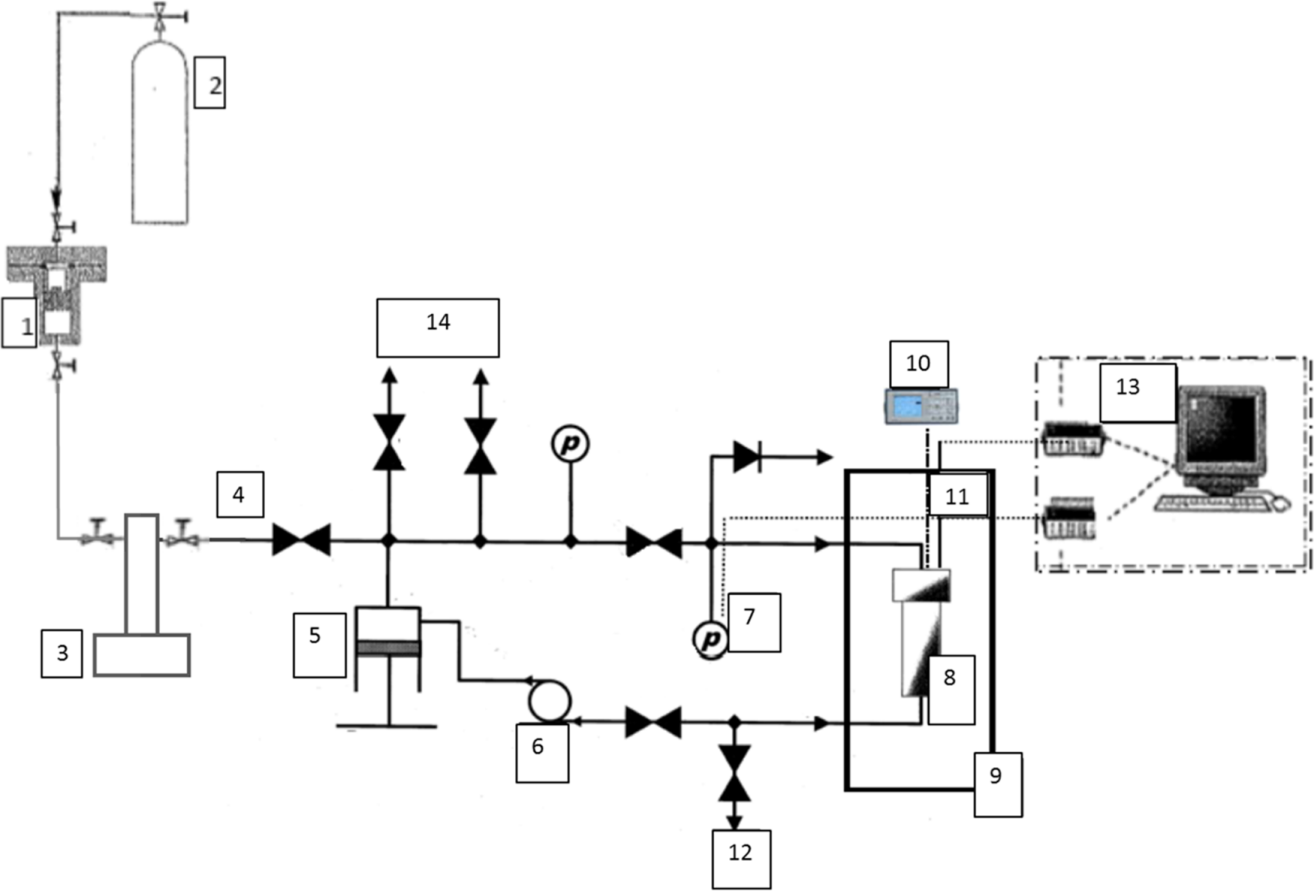
Experimental setup for speed of sound measurements: (1) load cell; (2) nitrogen (to push the piston of the load cell); (3) ISCO pump; (4) fluid inlet; (5) manual pump; (6) circulation pump; (7) pressure transducer; (8) pressure vessel containing the 5 MHz ultrasonic cell; (9) liquid thermoregulated bath; (10) oscilloscope; (11) platinum temperature probe; (12) drain line; (13) evaluation unit and data acquisition; and (14) clean and vacuum line.

Equation 1 ^71^ was utilized to compute the combined standard uncertainty (*u*(*c*)) for the experimental values of *c*.

1 $${\left(u\left(c\right)\right)}^{2}={\left[{\left(\frac{\partial c}{\partial T}\right)}_{p,x}u\left(T\right)\right]}^{2}+{\left[{\left(\frac{\partial c}{\partial p}\right)}_{T,x}u\left(p\right)\right]}^{2}+\sum_{i}{\left[{\left(\frac{\partial c}{\partial x_{i}}\right)}_{p,T}u\left(x_{i}\right)\right]}^{2}{+\left(u^{*}\left(c\right)\right)}^{2}$$

where *i* is each component of the doped Mix 1 and doped Mix 2 and u*­(*c*) is the standard uncertainty of repeatability in *c*. To determine u*­(*c*) for these systems, we prepared two mixtures with identical compositions for each system and measured the *p*–*c*–*T* isotherms for each mixture at temperatures of 263, 293, and 313 K, covering a pressure range of 10–195 MPa. For each isotherm, we conducted two measurements; however, only one measurement was performed for the mixtures containing O_2_ at 263 K. The compositions of these mixtures and their associated uncertainties are detailed in Tables 3 and S3, and the measured *c* values are shown in Table S4. From these experiments, we determined *u*(*c*) = 8.9 × 10^–4^ · *c* for the doped Mix 1, and *u*(*c*) = 4.6 × 10^–4^ · *c* for the doped Mix 2. These values are consistent with those reported in the literature for liquid and compressed gas mixtures measured using similar experimental setups.^29,30,72^

## 3 Results and Discussion

In Section 3.1, experimental and calculated results on density, VLE, and speed of sound are presented and discussed, and the impact of impurities on these results is explored. No data on these properties of the studied systems were found in the literature. Section 3.2 assesses the predictive ability of three EoSs. Section 3.3 examines how the simultaneous presence of SO_2_, CO, and (O_2_ or NO) affects various aspects of CCS stages, including their impact on seven specific saline aquifers (Table 1). For Mix 2, we have evaluated only its effect on the minimum operational pressure and storage capacity because the viscosity values for this system were unavailable.

We also compare our findings with previous studies on noncondensable impurities such as O_2_, CO, and CH_4_.^27–29,33^

### 3.1 Results and Discussion of the Experimental and Calculated Data

We measured nine *p*–ρ–*T* isotherms for Mix 1 (CO_2_ + O_2_ + SO_2_ + CO) and Mix 2 (CO_2_ + NO + SO_2_ + CO), as detailed in Table 2. These measurements were performed at specified nominal temperatures of 263.15, 273.15, 283.15, 293.15, 303.15, and 313.15 K, with pressures of up to 20 MPa. Additionally, we performed measurements at nominal temperatures of 333.15, 353.15, and 373.15 K, with pressures of up to 30 MPa. The *T* and *p* ranges were selected by considering the operational parameters relevant to pipeline transport^73–76^ and requirements for injection, storage, and prevailing conditions in typical geological storage sites.^57–63^ These ranges were extended to validate the EoSs over more extensive intervals.

Our experimental data set consists of ∼21 000 data points, each accompanied by its respective combined standard uncertainty. These results are provided in Table S1 and graphically illustrated in Figures 3a and S1. To facilitate their practical use, sets with ∼50 points per isotherm are compiled in Table S5. These sets include corresponding compressibility factor (*Z*) values and their respective combined standard uncertainties.

**Figure 3:**
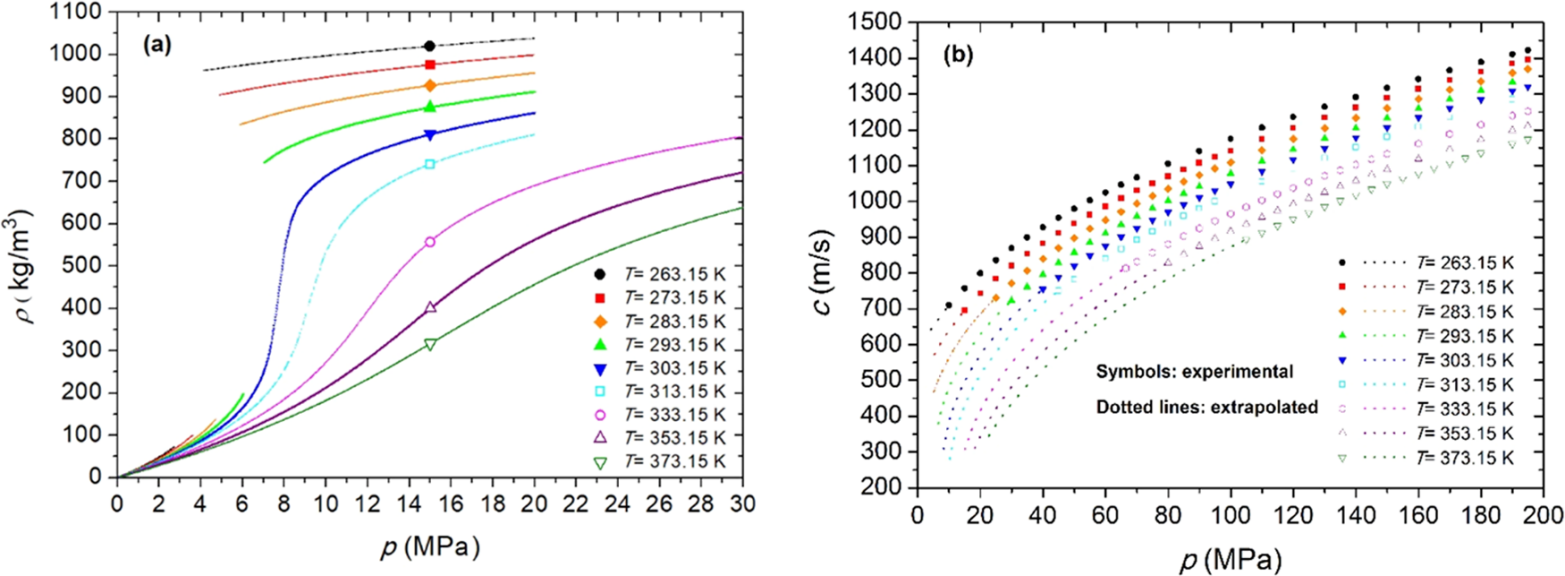
(a) This work’s experimental densities (ρ) of Mix 1. (b) This work’s experimental data and extrapolated values of the speed of sound (*c*) for doped Mix 2. Both were plotted against pressure (*p*) and at nominal temperatures *T*.

The examined mixtures exhibit subcritical behavior in the temperature range of 263.15–293.15 K. For the remaining investigated temperatures, the mixtures transitioned into the supercritical phase. Notably, 303.15 K is a supercritical temperature for the quaternary mixtures under investigation. However, this temperature remains subcritical for pure CO_2_ because its critical temperature (*T*_c_) is 304.21 K.^77^ At 303.15 K, Mix 2 exhibits continuous data behavior across the entire range of pressures studied, indicating its supercritical behavior. However, we could not obtain reproducible results in the pressure range of 6.7–8.0 MPa. Therefore, we have not reported density values for Mix 2 within this specific pressure interval.

Mix 1 contains 3.00 mol % of O_2_ and 0.17 mol % of CO, both of which are noncondensable impurities, unlike 0.09 mol % of SO_2_, which is a condensable impurity. Consequently, the measured density values for Mix 1 are lower than those for pure CO_2_ under the same *T* and *p* conditions throughout the studied *T* and *p* ranges^56^ (Figure S2). The most significant decreases in density are observed at 303 K with a mean relative deviation (MRD) of 6.25%, and the smallest decreases are observed at 273 K with an MRD of 2.27%.

The density data of Mix 1 are very close to those of the binary CO_2_ + O_2_ mixture with the same O_2_ content (3.0 mol %),^29^ with MRD values between 0.12% at 263 K and 1.55% at 303 K, respectively. The presence of O_2_ at the same concentration in both mixtures seems to have the most important effect on the fluid density, while the small amount of CO and SO_2_ (noncondensable CO and condensable SO_2_) in Mix 1 has a lesser influence than that of O_2_. The deviations at subcritical temperatures (263–293 K) are negative (lower density values for the quaternary mixture) for the entire vapor phase and for the liquid phase at pressures above values that increase with the temperature (5.61 MPa at 263 K up to 13.9 MPa at 293 K). At 373 K, lower density values are observed for the quaternary mixture compared with the binary mixture at all pressures with an MRD of 0.56%. In the remaining supercritical isotherms, lower density values are obtained except in certain pressure intervals for each temperature, i.e., from 5 to 9 MPa at 303 K, from 6 to 12 MPa at 313 K, from 9 to 16 MPa at 333 K, and from 12 to 18 MPa at 353 K. Therefore, CO has a predominant effect over SO_2_ in the quaternary mixture.

Mix 2 contains two noncondensable impurities (i.e., NO (0.14 mol %) and CO (0.17 mol %)) and one condensable impurity in a lower concentration (0.09 mol %), which decreases the density of Mix 2 compared to that of pure CO_2_ with MRD values between 0.28% at 283 and 373 K and 0.56% at 333 K (Figure S2). Therefore, the presence of SO_2_ in Mix 2 does not counteract the effect of noncondensable impurities.

Figure S3 compares the densities at the studied temperatures and selected pressures of 8.00, 14.00, 20.00, and 30.00 MPa for Mix 1, Mix 2, CO_2_ + 3.01 mol % O_2_,^29^ CO_2_ + 3.00 mol % CO,^27,28^ CO_2_ + 2.81 mol % CH_4_,^27,33^ and pure CO_2_.^56^ As shown in Figure S3, the densities are quite similar for Mix 1 and the three binary mixtures containing 3 mol % of a noncondensable impurity, all of which are clearly lower than those of pure CO_2_ at each temperature and pressure. The highest differences in density are observed near the critical zone of the systems, that is, at 8.00 MPa and temperatures between 300 and 310 K, and the lowest differences are observed at 8.00 MPa and high temperatures. The density values of Mix 2 are very close to those of pure CO_2_.

The VLE data for Mix 1 and Mix 2 at the subcritical temperatures (263.15 −293.15 K) are shown in Table S2 and depicted in Figure 4. Table S2 and Figure 4 also include data points for pure CO_2_ for comparative analysis.^56^

**Figure 4:**
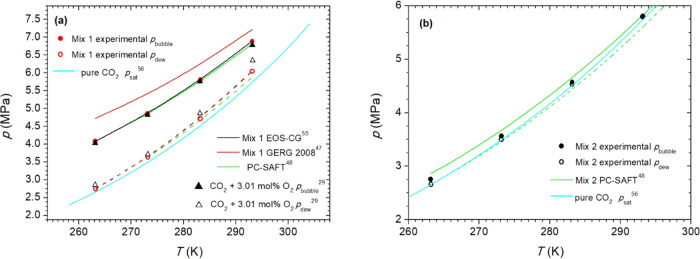
Experimental (this work) and calculated VLEs for Mix 1 (a) and Mix 2 (b). Panel (a) includes dew and bubble pressures of the CO_2_ + 3.01 mol % O_2_ mixture. Both graphs include *p*_sat_ of pure CO_2_. PC-SAFT parameters in panels (a, b) are taken from Table S11.

The dew pressures of Mix 1 are higher than the saturation pressures of pure CO_2_ (*p*_sat_; MRD = 4%), and the difference increases as the temperature increases (Table S2). However, the bubble pressures of Mix 1, which are also higher than *p*_sat_, exhibit the opposite behavior; the difference between the bubble pressure and *p*_sat_ of CO_2_ decreases as the temperature increases, with an average MRD of 36%. Reductions in the ρ_L_ of Mix 1 compared with the ρ_L_ of CO_2_ are obtained with MRD increasing with temperature and ranging from 2% at 263 K to 4% at 293 K; and increases of 2% are observed in the ρ_V_ of Mix 1 at 263.15, 273.15, 283.15, and 293.15 K.

Compared with the mixture CO_2_ + O_2_ 3.0 mol %,^29^ the *p*_dew_ of Mix 1 are lower than those of the binary mixture at the same *T* with an average MRD of 3%, and no trends between *p*_dew_ and *T* are observed. The corresponding average MRD for the *p*_bubble_ values is 1%, which can be considered not significant because it is in the same order as the *u*(*p*_bubble_) for Mix 1. For Mix 1, its ρ_L_ is greater than that of the binary mixture, and the differences in their ρ_L_ increase with *T* and MRD = 1%. The ρ_V_ values of Mix 1 are lower than those of the binary mixture, and the differences increase as the temperature increases and MRD = 7%.

*p*_dew_ and *p*_bubble_ of Mix 2 are higher than the *p*_sat_ of CO_2_ at the same temperature with MRDs of 1 and 2%, respectively. The ρ_L_ and ρ_V_ of Mix 2 can be considered equal to those of pure CO_2_ at each temperature because the MRD values (0.3 and 0.2%, respectively) are lower than the uncertainties of ρ_L_ and ρ_V_ for Mix 2.

For the speed of sound measurements, we measured nine *p*–*c*–*T*–*x* isotherms for each mixture in doped Mix 1 and doped Mix 2 (Table 3). The nominal temperatures matched those employed for density measurements, and the utilized pressures in experiments extended up to 190 MPa. The results are shown in Table S6, Figures 3b and S4.

Figure S5 compares the measured values of *c* for doped Mix 1 and pure CO_2_ under the same *T* and *p* conditions.^56^ Negative deviations in the *c* of doped Mix 1 are observed compared to that of pure CO_2_ across all studied *T* and *p* ranges, with MRD values decreasing from 2.38% at 263 K to 0.95% at 373 K. Regarding the comparison between the *c* values of doped Mix 1 and the doped binary CO_2_ + O_2_ with the same amount of O_2_,^29^ the doped Mix 1 exhibits lower *c* values than the doped binary mixture across all *T* and *p* ranges, with MRD values per isotherm decreasing from 0.79% at 263 K to 0.17% at 373 K. These behaviors highlight the stronger effect of the presence of O_2_ on the *c* of the doped Mix 1 compared with the effect of CO and SO_2_, and among these two minority impurities, CO has a greater effect on the *c* of the doped Mix 1 than SO_2_.

The measured *c* of the doped Mix 2 shows MRD values per isotherm, which decrease from 0.42% at 263 K to 0.09% at 373 K compared with pure CO_2_.^56^ At 373 K, the *c* values of the doped Mix 2 are lower than those of pure CO_2_ across the entire experimental pressure range, whereas at temperatures between 273 and 353 K, the deviations in the *c* values are positive from the lowest experimental pressure up to pressure values that increase with *T*, as shown in Figure S6 (up to 60 MPa at 273 K and up to 95 MPa at 353 K). At the remaining pressures, the deviations in the *c* values of the doped Mix 2 are negative. At 263 K, the *c* values of the doped Mix 2 at 10 and 15 MPa show negative deviations with respect to CO_2_; from 20 to 60 MPa, they show positive deviations, and at higher pressures, the deviations in *c* become negative again.

We correlated the experimental data obtained for the speed of sound as a function of pressure for each temperature and composition using polynomials, such as those in eq 2.^71^ These polynomials were employed to calculate the uncertainty in the experimental measurements of the speed of sound and to obtain values of this property at pressures lower than the experimental ones. This is particularly relevant, among other applications, for CCS technology, for which it is not possible to obtain values of the speed of sound at pressures of interest using our setup, even after the doping of mixtures.

2 $$\left(p-p^{\#}\right)=\sum_{i=1}^{3}a_{i}{\left(c-c^{\#}\right)}^{i}$$

where *p* ^*#*^ represents a suitable reference pressure for each isotherm and *c* ^#^ is the corresponding speed of sound at *p* = *p* ^#^. Table S7 shows the values of *p* ^*#*^ and the coefficients *a*_*i*_ in eq 2, along with the MRD_c_ (%) between the fitted and experimental data. The overall average $\overset{̿}{{\mathrm{MRD}}_{c}}$ values are 0.005 and 0.003% for doped Mix 1 and doped Mix 2, respectively, which are below the respective uncertainties associated with the experimental data.

The extrapolated data for Mix 1, shown in Table S8, were validated using the GERG-2008 EoS^47^ because this EoS best reproduces the experimental data for the sound velocity of Mix 1 compared with other EoSs evaluated in this study. In the case of Mix 2, the PC-SAFT EoS was used to validate the extrapolated data^48^ (see Section 3.2).

Using our experimental ρ data and experimental and extrapolated *c* data, we calculated isentropic compressibilities and Joule–Thomson coefficients for Mix 1 and Mix 2 throughout the entire temperature range using eqs 3–5.

3 $$\kappa_{S}=\frac{1}{\rho c^{2}}$$

4 $$C_{p}=\frac{\alpha_{p}^{2}T}{\rho \left(\kappa_{T}-\kappa_{S}\right)}$$

5 $$\mu_{\mathrm{JT}}={\left(\frac{\partial T}{\partial p}\right)}_{H}=\frac{V}{C_{p}}\left(\alpha_{p}T-1\right)$$

where *C*_p_ is the heat capacity of Mix 1 or Mix 2 at constant pressure, α_p_ and κ_T_ are the isobaric thermal expansivity and isothermal compressibility, respectively, and *V* is the molar volume. κ_S_ was determined using the experimental ρ data and experimental and extrapolated *c* values. α_p_ was calculated using our experimental density data in the temperature range of 263–373 K. To improve accuracy, density values calculated using the EOS-CG EoS^55^ for Mix 1 and PC-SAFT EoS^48^ for Mix 2 at temperatures of 253.15 and 383.15 K, respectively, were also incorporated, particularly to refine calculations at the temperature end points of the interval (see Section 3.2). κ_T_ was derived from the experimental ρ values. The results of κ_S_ and μ_JT_ are shown in Tables S9 and S10 and represented in Figures 5 and 6, respectively, along with those of pure CO_2_ for comparison.^56^

**Figure 5:**
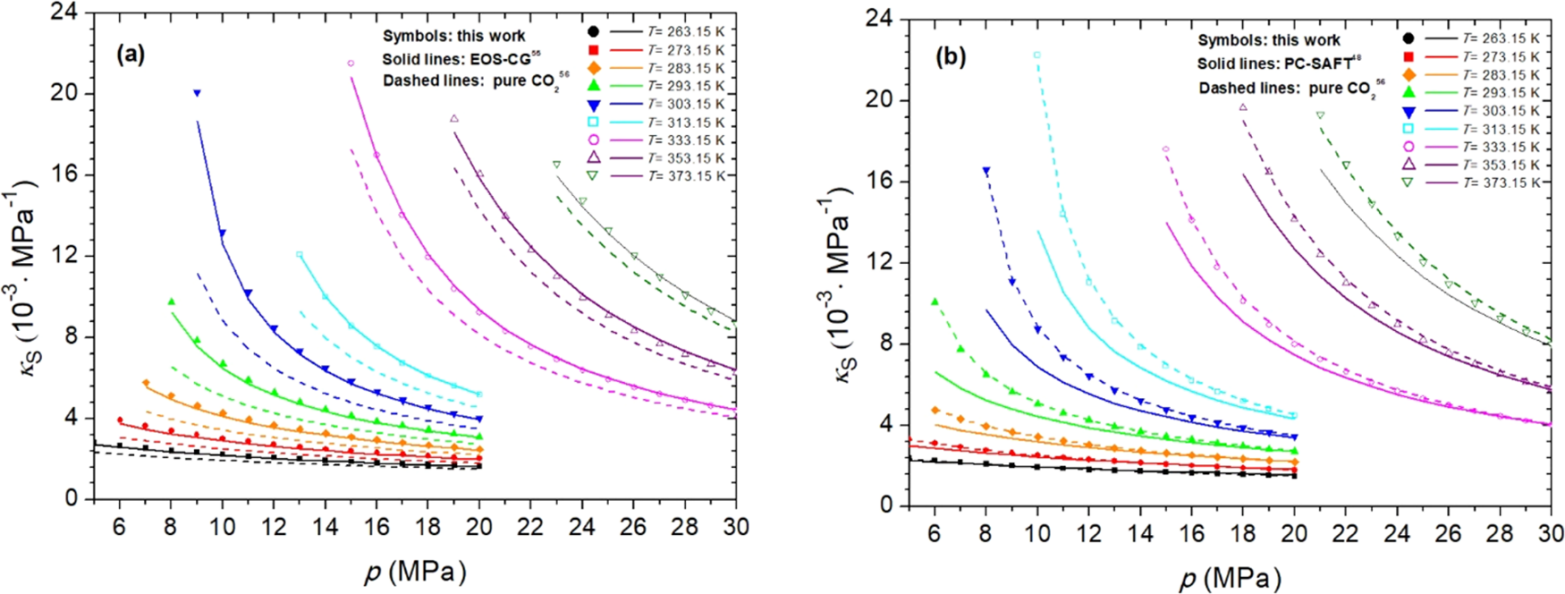
Calculated isentropic compressibilities (κ_S_) at several pressures (*p*) and temperatures (*T*) for Mix 1 (a) and Mix 2 (b). PC-SAFT EoS in panel (b) uses coefficients from Table S11.

**Figure 6:**
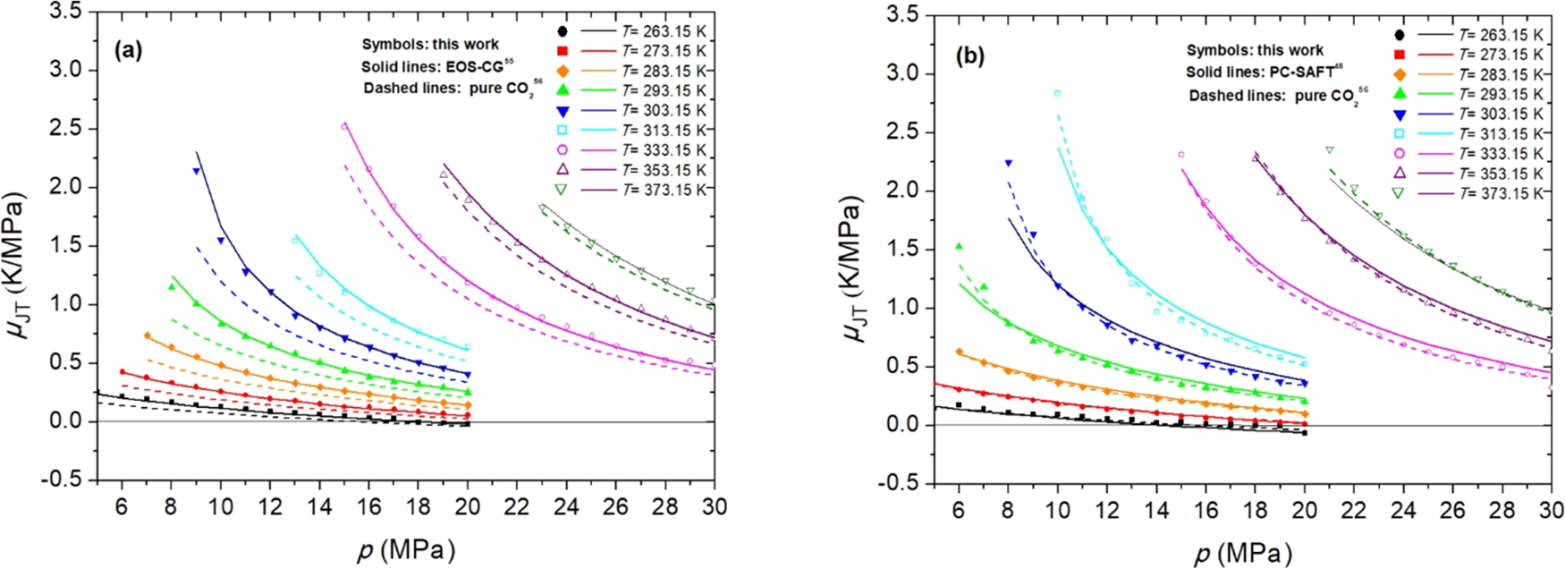
Calculated Joule–Thomson coefficients (μ_JT_) at several pressures (*p*) and temperatures (*T*) for Mix 1 (a) and Mix 2 (b). PC-SAFT EoS in panel (b) uses coefficients from Table S11.

κ_S_ and μ_JT_ are used to design and operate systems more efficiently, minimizing energy losses and ensuring the safe and effective management of anthropogenic CO_2_. Isentropic compressibility is used to understand and predict how a fluid will behave under different pressure and temperature conditions during compression for transport and storage.^49–51^ During transport, the Joule–Thomson coefficients are used to anticipate how the fluid temperature will change with varying pressures, which is crucial to avoid operational issues and ensure system efficiency.^52,54^ During the storage phase, μ_JT_ can be used to understand how the fluid will behave during injection and how it will affect the reservoir pressure and temperature.^53^

The impurities present in Mix 1 (3 mol % O_2_ + 0.09 mol % SO_2_ + 0.17 mol % CO) cause positive deviations in κ_S_ compared to pure CO_2_,^56^ with an absolute average deviation (AAD) of 0.43 × 10^–3^ MPa^–1^ at 263 K, which increases to 2.12 × 10^–3^ MPa^–1^ at 303 K. At higher temperatures, the AADs decrease as the temperature increases, reaching 0.87 × 10^–3^ MPa^–1^ at 373 K.

The impurities in Mix 1 also increase μ_JT_ compared to that of pure CO_2_ across all studied *T* and *p* ranges.^56^ The AADs increase from 0.05 K MPa^–1^ at 263 to 0.18 K MPa^–1^ at 303 K. At higher temperatures, the AADs decrease with an increase in temperature, reaching 0.07 K MPa^–1^ at 373 K.

The κ_*S*_ values obtained for Mix 2 are practically identical to those of CO_2_,^56^ and at temperatures where the highest deviations are observed, i.e., 263 K (AAD = 0.02 × 10^–3^ MPa^–1^) and 283 K (AAD = 0.09 × 10^–3^ MPa^–1^), the κ_S_ values for Mix 2 are slightly higher than those of pure CO_2_, with differences increasing as the pressure decreases. The μ_JT_ values obtained for Mix 2 are lower than those of CO_2_ at 273 K but higher at most of the other studied temperatures and pressures. However, the effect of impurities (0.14 mol % NO + 0.09 mol % SO_2_ + 0.17 mol % CO) on this property is very small (the AAD ranges from 0.005 to 0.09 K MPa^–1^).

The μ_JT_ of Mix 1 and Mix 2 exhibit positive values across the studied *T* and *p* ranges, except at *T* = 263.15 K and *p* ≳ 17.6 MPa for Mix 1 and *T* = 263.15 K and *p* ≳ 18.4 MPa for Mix 2, where they transition to negative values. These inversion pressures surpass the inversion pressure of pure CO_2_ at 263.15 K, which is 15.65 MPa.^56^ A positive μ_JT_ value implies that the fluid cools during depressurization, whereas a negative μ_JT_ value indicates that the fluid warms during depressurization.

### 3.2 EoS Validation and Comparison of Results

Our experimental and calculated results and results obtained from specific EoSs were compared to assess the suitability of these EoSs for use in CCS technology because currently there is no identified optimal equation in the literature for this purpose.

The equations employed are the EOS-CG mixture model,^55^ GERG-2008 model,^47^ and PC-SAFT EoS,^48^ applied using TREND 4.0 software,^78^ REFPROP 10.0 software,^79^ and VLXE software,^80^ respectively. All three equations can be applied to Mix 1, but only the PC-SAFT EoS can be applied to Mix 2 because the other two lack a mixture model for CO_2_ + NO. The parameters for pure components and binary interaction parameters used to apply the PC-SAFT EoS to both mixtures are detailed in Table S11. The parameters used for O_2_ and the binary interaction coefficient of CO_2_ + O_2_ ^81^ are the best for this binary mixture, as reported in a previous study,^29^ where various parameters from the literature were evaluated. Meanwhile, the parameters used for NO and CO_2_ + NO were those obtained in a previous study.^29^

The differences between our results and those calculated using evaluated EoSs are given by the MRD for each property *X* (MRD_*X*_) and are shown in Tables S12–S15, along with the overall average MRD values for each property *X* ( $\overset{̿}{{\mathrm{MRD}}_{X}}$ ). The relative deviations between the values derived from the assessed EoSs and experimental properties obtained in this study are illustrated for each isotherm in Figures S7 and S8.

The EOS-CG EoS reproduces all density isotherms of Mix 1 better than the GERG-2008 EoS; however, the differences are small, and both equations yield very good results with overall deviations of 0.50 and 0.73%, respectively. The PC-SAFT EoS adequately reproduces the density isotherms of Mix 1 and Mix 2, with higher overall deviations than the EOS-CG and GERG-2008 EoSs, which are 1.42 and 1.30% for Mix 1 and Mix 2, respectively.

Regarding the VLE, the EOS-CG EoS reproduces the *p*_dew_ and *p*_bubble_ of Mix 1 with very low errors (0.06 and 0.09%, respectively), whereas the other two equations show larger deviations, with GERG-2008 being the poorest in reproducing the *p*_bubble_ values of Mix 1. The results of the three EoSs for ρ_V_ and ρ_L_ of Mix 1 are similar except for the prediction of ρ_V_ using the PC-SAFT EoS, where significantly higher deviations are observed. The PC-SAFT EoS correctly calculates the studied properties of the VLE of Mix 2, yielding better results in the calculation of *p*_bubble_ and ρ_L_ (1.62 and 1.23%, respectively) than in the calculation of *p*_dew_ and ρ_V_ (2.31 and 3.47%, respectively).

The *c* measurements of doped Mix 1 and Mix 2 were modeled by treating them as pseudobinary mixtures, i.e., by incorporating the mole fractions of methanol into those of CO_2_.^34,35^ The GERG-2008 EoS best reproduces the *c* measurements of doped Mix 1 with an $\overset{̿}{{\mathrm{MRD}}_{c}}$ = 0.21%; however, results obtained using the EOS-CG EoS are also very good with an $\overset{̿}{{\mathrm{MRD}}_{c}}$ of 0.42%. The PC-SAFT EoS provides poorer results compared to the other two EoSs, with overall deviations of 4.27 and 4.45% in the prediction of *c* for Mix 1 and Mix 2, respectively.

Because good results were obtained using the GERG-2008 EoS, this EoS was used to validate the results of *c* extrapolated from the experimental *c* values for Mix 1. The deviations between the EoS-predicted *c* values and extrapolated *c* values are somewhat worse than those corresponding to experimental *c* values in the case of EOS-CG and GERG-2008 EoSs, which are 0.59 and 0.70%, respectively (Table S12). However, in the case of the PC-SAFT EoS, the opposite occurs. The PC-SAFT EoS predicts the extrapolated *c* values of Mix 1 slightly better than the experimental data, with an overall deviation of 3.23%. The extrapolated *c* values of Mix 2 were validated using the PC-SAFT EoS, and the obtained deviation (3.10%) was also lower than that with experimental *c* values.

Similarly, the predictive capability of the evaluated EoSs was assessed for κ_S_ and μ_JT_. The differences between the predictions and experimental values of these properties are reported as the AAD values in Tables S14 and S15. The EOS-CG EoS yields the best results for κ_S_ and μ_JT_ of Mix 1. The results obtained with the PC-SAFT EoS for the κ_S_ of Mix 2 are more favorable than that for Mix 1, and the opposite is observed for μ_JT_.

### 3.3 Influence of the Simultaneous Presence of SO_2_, CO, and (O_2_ or NO) Impurities on the Transport, Injection, and Storage Stages of CCS Technology

After evaluating the influence of the simultaneous presence of noncondensable impurities (i.e., CO and O_2_ or CO and NO) and condensable impurities (i.e., SO_2_) on the thermodynamic properties of Mix 1 and Mix 2, we quantified the effect of these impurities on selected parameters in the transport, injection, and storage stages of CCS technology. To this end, we compared the parameter values obtained for the mixtures with those calculated for pure CO_2_.

The chosen parameters for the transport step include the minimum operational pressure (*p*_min_), pressure drop (*p*(*d*)) and density drop (ρ­(*d*)), both as functions of the distance along the pipeline (*d*), and inner diameter of the pipeline (*D*). The injection and storage parameters were normalized as $\frac{X}{X_{0}}$ , where *X* is the mixture value and *X*_0_ is the value for pure CO_2_. These parameters include the reservoir storage capacity (*M*), rising plume velocity within deep saline aquifers (*v*), and permeation flux (*Ṁ*).

Transport parameters were calculated at temperatures ranging from 263 to 303 K and pressures up to 20 MPa, and injection and storage parameters were assessed under storage conditions, i.e., nominal temperatures ranging from 303 to 373 K and *p* ≥ 7 MPa.

The equations used to calculate the CCS parameters are detailed in Table S16.^12,73,74^ For their application, we utilized the experimental density values obtained in this study for Mix 1 and Mix 2 along with those from the literature for CO_2_.^56^ The viscosity values for Mix 1 were determined using REFPROP 10.0 software.^79^ However, we did not find a valid calculation method in the literature for estimating the viscosity of Mix 2, precluding the calculation of parameters associated with this property for Mix 2. For this reason, only the minimum operational pressure and normalized storage capacity could be determined for Mix 2. The CCS parameter values obtained for Mix 1 and Mix 2 were also compared with those of CO_2_ + O_2_, CO_2_ + CO, and CO_2_ + CH_4_ with 3 mol % of noncondensable impurities.^27–29,33^

The densities of brine in saline aquifers (ρ_br_; Table 1) were estimated based on the salinity, temperature, and pressure conditions in the corresponding reservoirs.^58^

#### 3.3.1 Influence of the Simultaneous Presence of SO_2_, CO, and (O_2_ or NO) Impurities on Transport

##### 3.3.1.1 Minimum Operational Pressure

Because the fluid must be transported in a dense phase to avoid the biphasic flow,^75,76^ the bubble pressure of the system at each temperature marks the lower limit of the operating pressure (plus an adequate safety margin). As shown in Figure S9, the impurities in Mix 1 jointly impact the *p*_bubble_ very similar to that in the unique presence of O_2_ (3.0 mol %),^29^ a noncondensable impurity. In this way, the *p*_bubble_ of Mix 1 increases between 20 and 54% with respect to the saturation pressure of pure CO_2_ at the studied temperatures, and the differences are larger at lower temperatures. The opposite effects of CO (noncondensable impurity) and SO_2_ (condensable impurity)^34^ at the studied concentrations practically cancel each other out, with a slight predominance of CO. Instead, in Mix 2, the effect of impurities on the *p*_bubble_ is much lower (Figure S9), which increases from 1 to 4% with respect to CO_2_, and the same trend as in Mix 1 is observed with the temperature.

Figure S9 also includes the *p*_bubble_ of the CO_2_ + 3.00 mol % CO and CO_2_ + 2.81 mol % CH_4_ mixtures.^28,29,33^ As shown in the figure, the effect of CO on *p*_bubble_ is the greatest among all studied impurities, approximately double that of O_2_, whereas CH_4_ has a minor influence on *p*_bubble_ of the fluid at the same concentration.

##### 3.3.1.2 Pressure and Density Drop along the Pipeline, *p*(*d*) and ρ­(*d*)

Figures S10 and S11 show the pressure and density drops, respectively, at the studied temperatures along a model pipeline for Mix 1, compared with pure CO_2_. The model pipeline has an inner diameter of 20.0 inch (0.508 m) and a roughness height of 0.00015 ft. (4 × 10^–5^ m) and transports a mass flow of 10.00 Mt/year (317.1 kg/s) with an initial pressure of 20.00 MPa. Figures S12 and S13 show the pressure and the density, respectively, 300 km away from the pipeline entrance for pure CO_2_, Mix 1, and binary CO_2_ + 3.01 mol % O_2_, CO_2_ + 3.00 mol % CO, and CO_2_ + 2.81 mol % CH_4_ mixtures,^27–29,33^ where all mixtures having similar concentrations of noncondensable impurities. The four mixtures exhibit similar pressure and density drops, faster than those of pure CO_2_, with differences that increase with an increase in the temperature and are the maximum near the critical zone (*T* > 300 K). For Mix 1, at 263.15, 273.15, 283.15, 293.15, and 303.15 K, the pressure 300 km away from the pipeline entrance decreases to 57.0, 55.3, 53.0, 50.1, and 45.6% of the initial pressure, respectively. For pure CO_2_, the pressure decreases to 57.6, 56.3, 54.3, 51.9, and 48.4% at the same temperatures, respectively. The density of Mix 1 300 km away from the pipeline entrance decreases to 95.7, 95.4, 93.4, 89.50, and 78.3% of the initial density, while that of pure CO_2_ decreases to 97.0, 96.2, 94.6, 91.9, and 85.7% at the same temperatures, respectively.

##### 3.3.1.3 Inner Diameter of the Pipeline, D

Figure S14 shows the inner diameter that a pipeline with roughness height of 0.00015 ft. must have to be able to transport a mass flow of 317.1 kg/s (10 Mt/year) for Mix 1, CO_2_ and CO_2_ + 3.01 mol % O_2_, CO_2_ + 3.00 mol % CO, and CO_2_ + 2.81 mol % CH_4_ mixtures^27–29,33^ at selected pressures of 8.00, 14.00, and 20.00 MPa. An average value of 31.75 Pa/m was used for the pressure drop per meter. All studied mixtures require a pipeline diameter larger than that of pure CO_2_ at the studied temperatures and pressures, and the differences increase as the temperature increases and the pressure decreases. At *T* of 263.15, 273.15, 283.15, and 293.15 K and at the three pressures studied (8.00, 14.00, and 20.00 MPa) as well as at 303.15 or 304.21 K and 14 or 20 MPa, the required pipeline diameters are very similar for all mixtures, and the highest differences between pipeline diameters for all mixtures are ∼1 mm at each *T* and *p*, and differences with respect to pure CO_2_ range from 1 to 8 mm. The latter differences increase with an increase in temperature and a decrease in pressure.

At 303.15 or 304.21 K and 8 MPa, close to the critical zones, the differences in pipeline diameters increase strongly. The pipeline diameter for the binary CO_2_ + 3.01 mol % O_2_ mixture is very similar to that for Mix 1, both ∼42 mm larger than that for CO_2_. The effect of CH_4_ is smaller, and that of CO is stronger on the pipeline diameter, obtaining diameters 32 and 60 mm larger than that for CO_2_, respectively.

For a standard carbon steel API 5L X70 pipeline with an inner diameter of 0.508 m (20.0 inch) and a wall thickness of 12.7 mm (0.5 inch), an increase of 10.0 mm in the inner diameter leads to an increase of 3.1 tons of steel per km of the pipeline.

#### 3.3.2 Influence of the Simultaneous Presence of SO_2_, CO, and (O_2_ or NO) Impurities on Injection and Storage

##### Normalized Storage Capacity, $\frac{M}{M_{0}}$

3.3.2.1

Figure S15 shows the $\frac{M}{M_{0}}-p$ isotherms for Mix 1 and Mix 2 based on our experimental density results. These representations allow for an assessment of the influence of impurities present in the studied mixtures, as well as *T* and *p*, on the efficiency of a specific geological storage. In the isotherms of Mix 1 (Figure S15a), minima are observed. These minima become less pronounced as the temperature increases and also shift to higher pressure values. This behavior is typical of noncondensable impurities and indicates the predominance of the effects of O_2_ and CO in this mixture. The smallest reductions in *M* ≅ 5% are obtained under the highest studied *T* and *p* conditions corresponding to deep storage.

The impurities in Mix 2 have a lesser impact on $\frac{M}{M_{0}}$ compared to those in Mix 1, and the same is observed for *T* and *p* (Figure S15b). The values of $\frac{M}{M_{0}}$ are less than unity in all of the studied *T* and *p* ranges, suggesting the predominance of the effects of NO and CO over the effect of SO_2_ in the mixture. The reductions in *M* range between 0.3 and 1.9%, with the greatest reduction observed at 313 K and 9 MPa.

The storage of Mix 2 in the actual reservoirs listed in Table 1 (Figure 7a) results in reductions of less than ≅1% in *M* compared to pure CO_2_, regardless of the reservoir. For the rest of the mixtures shown in Figure 7a, the greatest reductions are observed in the four least deep storages. It is within these storages that the most significant differences among the five studied mixtures become evident, and the CO_2_ + 3.01 mol % O_2_ mixture^29^ appears to be the least favorable for storing. Among them, the presence of SO_2_ in Mix 1 results in a partial counteraction against the adverse effects of the presence of O_2_ and CO.

**Figure 7:**
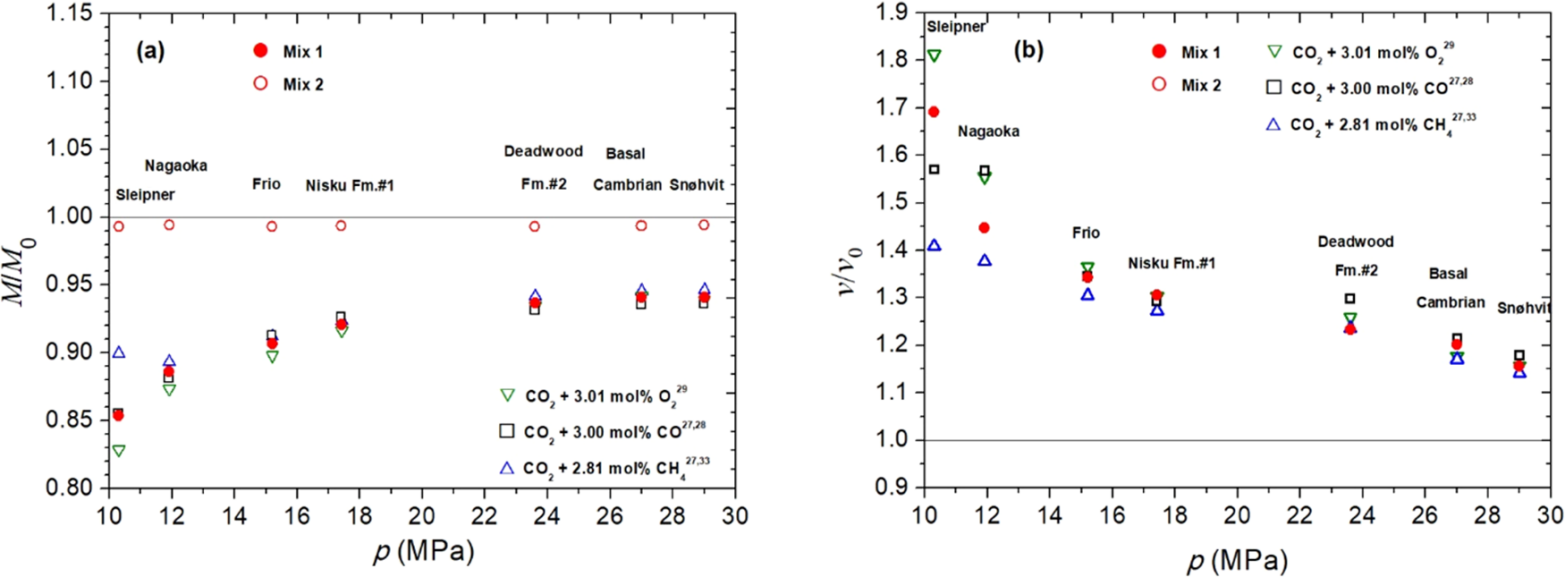
(a) Normalized storage capacity (*M*/*M*_0_) of the studied saline aquifers in Table 1. (b) Normalized rising velocity (*v*/*v*_0_) in the same reservoirs. Different mixtures were considered for comparison.

##### Normalized Velocity of the Rising Plume in Saline Aquifers, $\frac{v}{v_{0}}$

3.3.2.2

The isotherms $\frac{v}{v_{0}}-p$ of Mix 1 are shown in Figure S16, considering two types of brine in the saline aquifer, i.e., a concentrated brine and a diluted brine. In both graphs, functions with maxima are observed, which are typical of noncondensable impurities. Therefore, it can be inferred that the effect of the condensable impurity (i.e., SO_2_) in Mix 1 is surpassed by the presence of the other two impurities (i.e., CO and O_2_). At a given temperature, the maximum reaches higher values of $\frac{v}{v_{0}}$ in the case of the concentrated brine than in the diluted one, and in both instances, the maxima decrease as the temperature increases and shift toward higher pressure values. $\frac{v}{v_{0}}>1$ implies that the mixture is less favorable for storing than pure CO_2_ because a higher plume rise velocity worsens fluid trapping in the saline aquifer.

The presence of Mix 1 is unfavorable in all actual wells depicted in Figure 7b; however, as the storage depth increases, the effect of impurities present in this mixture becomes less significant, reaching the lowest increase in *v* at Snøhvit with a 15% difference compared to pure CO_2_. In the shallowest storage well, the CO_2_ + 3 mol % O_2_ mixture^29^ is the most detrimental and the CO_2_ + 3 mol % CH_4_ mixture^27,28,33^ is the least unfavorable for all reservoirs.

##### Normalized Permeation Flux, $\frac{Ṁ}{{Ṁ}_{0}}$

3.3.2.3

The $\frac{Ṁ}{{Ṁ}_{0}}-p$ isotherms shown in Figure S17 allow for the analysis of the dependency of the injectivity of Mix 1 on *T* and *p* within the reservoir. The most favorable temperatures are the three lowest, ranging from 303 to 333 K, and the pressures depend on the isotherm. At 303 K, practically all studied pressures yield *Ṁ* > *Ṁ*_0_; at 313 K, this situation occurs for *p* ≳ 9 MPa and for *p* ≳ 14 MPa at 333 K. However, in cases where $\frac{Ṁ}{{Ṁ}_{0}}<1$ , the greatest reduction is not very high, at most ∼7%. Because $\frac{Ṁ}{{Ṁ}_{0}}=\frac{M}{M_{0}}\cdot \frac{\eta_{0}}{\eta }$ , by comparing Figures S17 and S15a, it can be inferred that under the specified *T* and *p* conditions, where $\frac{Ṁ}{{Ṁ}_{0}}>1$ , the favorable behavior of Mix 1 compared to pure CO_2_ is attributed to the low viscosity of the mixture under those conditions.

All studied mixtures generally yield values of *Ṁ* that are equal to or better than those of pure CO_2_ in the shallow reservoirs (Figure S18). They are worse in the three deepest reservoirs, but the reductions are very low, <2.5%. Injectivity is more favored in the two shallowest reservoirs, and in them, binary mixtures with CH_4_ or CO^27,28,33^ produce the best results.

## 4 Conclusions

We conducted experiments to determine the density, the limits of the VLE, and the speed of sound for two quaternary mixtures, i.e., Mix 1 [CO_2_ + 3.0038 mol % O_2_ + 0.09035 mol % SO_2_ + 0.17032 mol % CO] and Mix 2 [CO_2_ + 0.1410 mol % NO + 0.09100 mol % SO_2_ + 0.17002 mol % CO], which model the emissions from the oxy-fuel combustion of biomass (pure or blended with coal) without further purification as their minor component concentrations replicate the impurity levels present in flue gas from such processes. Mix 2 additionally serves as a representative model for emissions from gas engine combustion. Experimental conditions included temperatures and pressures ranging from 263 to 373 K and up to 30 MPa for density measurements and up to 190 MPa for speed of sound measurements, respectively. These conditions include those of the transport, injection, and storage phases of the CCS technology.

The densities and speeds of sound for Mix 1 are reduced with respect to pure CO_2_ under the same temperature and pressure conditions due to the effect of impurities, with O_2_ (a noncondensable impurity) causing the most significant influence. Among the two minor impurities, CO (noncondensable) has a greater effect than that of SO_2_ (condensable).

For Mix 2, the condensable impurity (i.e., SO_2_) does not compensate for the effects of NO and CO, resulting in lower densities compared to pure CO_2_ under the same temperature and pressure conditions. However, these density reductions are considerably less pronounced than those observed for Mix 1. The speeds of sound of Mix 2 are quite similar to those of CO_2_ under the same conditions, with differences alternating in sign across the studied pressure range.

For temperatures between 263.15 and 293.15 K, both Mix 1 and Mix 2 exhibit subcritical behavior. In both mixtures, the *p*_dew_ and *p*_bubble_ are higher than the *p*_sat_ of pure CO_2_, with Mix 1 showing significantly larger deviations.

The impurities in Mix 1 increase its κ_S_ and μ_JT_ values compared to those of pure CO_2_ across the studied *T* and *p* ranges. In Mix 2, the impurities lead to only minimal variations in these properties relative to those of pure CO_2_. μ_JT_ inversions are observed in both mixtures at 263.15 K, with pressure values exceeding the inversion pressure of pure CO_2_ at this temperature.

Using our experimental data, we validated the EOS-CG, GERG-2008, and PC-SAFT EoSs for Mix 1, with EOS-CG and GERG-2008 yielding the most accurate results. For Mix 2, where only the PC-SAFT EoS is applicable, the validation demonstrated its reliability, particularly for predicting density, *p*_bubble_, and ρ_L_.

The cotransport and costorage of CO_2_/O_2_ + SO_2_ + CO exhibit drawbacks similar to other noncondensable impurities (e.g., O_2_, CO, or CH_4_) under the entire range of studied transport conditions. However, the coinjection of CO_2_/O_2_ + SO_2_ + CO can prove advantageous in the majority of examined aquifers, and deep reservoirs are recommended for the CO_2_/O_2_ + SO_2_ + CO costorage because this helps mitigate the adverse effects of O_2_ and CO.

The impact of impurities in Mix 2 is minimal; consequently, it behaves very similarly to pure CO_2_ in terms of the minimum operational pressure and storage efficiency.

Our analysis exclusively focused on the thermodynamic and hydraulic aspects of the cocapture processes of CO_2_/impurities without addressing potential chemical effects arising from the presence of impurities.

## Supplementary Material



## Notes

Determination of the combined standard uncertainties in the compositions of the doped Mix 1 and doped Mix 2 used for the speed of sound measurements; tables and figures displaying the experimental and calculated data; outcomes of EoS modeling; and results for the calculated parameters related to CCS (PDF)

## Nomenclature

*a*_*i*_: coefficients of the polynomials used to correlate the speed of sound values.
*c*: speed of sound
CCS: carbon capture and storage
*d*: distance advanced by the stream along a pipeline
*D*: inner diameter of a pipeline
EoS: equation of state
*k*_*ij*_: binary interaction parameter (PC-SAFT EoS)
*M*/*M*_0_: normalized storage capacity of a reservoir
*Ṁ*/*Ṁ*_0_: normalized permeation flux of a plume in a reservoir
MRD_*X*_: mean relative deviation for a property *X* ${\mathrm{MRD}}_{X}\left(\%\right)=\frac{100}{N}\sum \left|\frac{X-X_{\mathrm{ref}}}{X_{\mathrm{ref}}}\right|$ *N*: number of points for each composition and temperature
$\overset{̿}{{\mathrm{MRD}}_{X}}$: overall average mean relative deviation for a property *X* $\overset{̿}{{\mathrm{MRD}}_{X}}\left(\%\right)=\frac{100}{N^{\prime }}\sum \left|\frac{X-X_{\mathrm{ref}}}{X_{\mathrm{ref}}}\right|$ *N*′: total number of points for each property
*n*: mole number
*p*: pressure
*p* ^#^: reference pressure in the polynomials used to correlate the speed of sound values
*p*_bubble_: bubble pressure
*p*_dew_: dew pressure
*p*_sat_: saturation pressure
*T*: temperature
*T*_c_: critical temperature
*u*(*X*): standard combined uncertainty of a property *X*
*v*/*v*_0_: normalized velocity of the rising plume in saline aquifers
VLE: vapor–liquid equilibrium
*x*: mole fraction
*Z*: compressibility factor
η: viscosity
κ_S_: isentropic compressibility
μ_JT_: Joule–Thomson coefficient
ρ: density
τ: vibration period of the densimeter cell
