# Supplementary material for: Viability of the Cocapture of CO2 and Impurities from Oxy-Fuel Combustion and Other Processes in Carbon Capture and Storage Technology
Source: Energy Fuels. 2025 Jun 2;39(23):11173–86. doi: 10.1021/acs.energyfuels.4c04818 (PMC12172091; doi:10.1021/acs.energyfuels.4c04818)

Viability of the cocapture of CO_2 and impurities from oxy-fuel combustion and other processes in carbon capture and storage technology

Héctor Almazán [a], *Javier Fernández* [a b]*, *and Sofía T. Blanco* [a b]

[a] Departamento de Química Física, Facultad de Ciencias, Universidad de Zaragoza, 50009 Zaragoza, Spain

[b] Instituto de Investigación en Ingeniería de Aragón (I3A). Universidad de Zaragoza, Mariano Esquillor s/n, 50018, Zaragoza, Spain.

*Email: javierf@unizar.es

SUPPORTING INFORMATION

TABLE OF CONTENTS	pp.
Determination of the combined standard uncertainties in the compositions of the doped Mix 1 an doped Mix 2 used for the speed of sound measurements	S4
Table S1. Experimental densities of the CO_2 + O_2 + SO_2 + CO (Mix 1) and CO_2 + NO + SO_2 + CO (Mix 2) mixtures.	S8
Table S2. Experimental VLE of the CO_2 + O_2 + SO_2 + CO (Mix 1) and CO_2 + NO + SO_2 + CO (Mix 2) mixtures.	S198
Table S3. Composition of the mixtures for the uncertainty study of the experimental speed of sound	S199
Table S4. Experimental speeds of sound for the CO_2 + CH_3OH + O_2 + SO_2 + CO and CO_2 + CH_3OH + NO + SO_2 + CO mixtures for the uncertainty study (doped Mix 1 and Mix 2 for uncertainty).	S200
Table S5. Reduced experimental densities of the CO_2 + O_2 + SO_2 + CO (Mix 1) and CO_2 + NO + SO_2 + CO (Mix 2) mixtures.	S208
Table S6. Experimental speeds of sound for the CO_2 + CH_3OH + O_2 + SO_2 + CO and CO_2 + CH_3OH + NO + SO_2 + CO (doped Mix 1 and Mix 2) mixtures.	S222
Table S7. Coefficients in the correlation of the experimental speeds of sound.	S232
Table S8. Extrapolated speeds of sound for the CO_2 + CH_3OH + O_2 + SO_2 + CO and CO_2 + CH_3OH + NO + SO_2 + CO (doped Mix 1 and Mix 2) mixtures.	S233
Table S9. Isentropic compressibilities for the CO_2 + O_2 + SO_2 + CO (Mix 1) and CO_2 + NO + SO_2 + CO (Mix 2) mixtures.	S246
Table S10. Joule-Thomson coefficients for the CO_2 + O_2 + SO_2 + CO (Mix 1) and CO_2 + NO + SO_2 + CO (Mix 2) mixtures.	S250
Table S11. Parameters used in the modeling of the CO_2 + O_2 + SO_2 + CO (Mix 1) and CO_2 + NO + SO_2 + CO (Mix 2) systems with the PC-SAFT EoS	S254
Table S12. Comparison between this work experimental density and experimental and extrapolated speed of sound data and those calculated using the evaluated EoS.	S255
Table S13. Comparison between this work experimental VLE data and those calculated using the evaluated EoS	S257
Table S14. Comparison between this work isentropic compressibilities and those calculated using the evaluated EoS.	S258
Table S15. Comparison between this work Joule-Thomson coefficients and those calculated using the evaluated EoS.	S260
Table S16. Equation overview for the calculation of the CCS parameters	S262
Figure S1. Experimental densities of the CO_2 + NO + SO_2 + CO (Mix 2) mixture.	S263
Figure S2. Comparison between the experimental densities of the CO_2 + O_2 + SO_2 + CO (Mix 1) and CO_2 + NO + SO_2 + CO (Mix 2) mixtures and those of pure CO_2.	S264

Figure S3. Comparison between the experimental densities obtained in this work for the CO_2 + O_2 + SO_2 + CO (Mix 1) and CO_2 + NO + SO_2 + CO (Mix 1) mixtures and those found in the literature for CO_2 + O_2, CO_2 + CO, CO_2 + CH_4, and CO_2.	S266
Figure S4. Experimental and extrapolated speeds of sound for doped Mix 1 (CO_2 + CH_3OH + O_2 + SO_2 + CO).	S267
Figure S5. Deviations between the experimental speeds of sound of doped Mix 1 (CO_2 + CH_3OH + O_2 + SO_2 + CO) and those of pure CO_2.	S268
Figure S6. Deviations between the experimental speeds of sound of doped Mix 2 (CO_2 + CH_3OH + NO + SO_2 + CO) and those of pure CO_2.	S269
Figure S7. Deviations between the experimental densities obtained in this work for the studied mixtures and those calculated using the evaluated EoS.	S270
Figure S8. Deviations between the experimental speeds of sound obtained in this work for doped Mix 1 and Mix 2 and those calculated using the evaluated EoS.	S271
Figure S9. Comparison of the experimental bubble pressures obtained in this work for the CO_2 + O_2 + SO_2 + CO (Mix 1) and CO_2 + NO + SO_2 + CO (Mix 2) mixtures and those found in the literature for CO_2 + O_2, CO_2 + CO and CO_2 + CH_4, and the saturation pressure of CO_2.	S272
Figure S10. Pressure profile along the pipeline for the studied CO_2 + O_2 + SO_2 + CO mixture (Mix 1) and pure CO_2.	S273
Figure S11. Density profile along the pipeline for the studied CO_2 + O_2 + SO_2 + CO mixture (Mix 1) and pure CO_2.	S274
Figure S12. Comparison of the pressure at 300 km from the pipeline inlet for the mixtures CO_2 + O_2 + SO_2 + CO (Mix 1), CO_2 + O_2, CO_2 + CO, CO_2 + CH_4 and pure CO_2.	S275
Figure S13. Comparison of the density at 300 km from the pipeline inlet for the mixtures CO_2 + O_2 + SO_2 + CO (Mix 1), CO_2 + O_2, CO_2 + CO, CO_2 + CH_4 and pure CO_2.	S276
Figure S14. Pipeline inner diameter for the mixtures CO_2 + O_2 + SO_2 + CO (Mix 1), CO_2 + O_2, CO_2 + CO, CO_2 + CH_4 and pure CO_2.	S277
Figure S15. Normalized storage capacity for the studied mixtures CO_2 + O_2 + SO_2 + CO (Mix 1) and CO_2 + NO + SO_2 + CO (Mix 2).	S278
Figure S16. Normalized rising velocity for the CO_2 + O_2 + SO_2 + CO (Mix 1) mixture.	S279
Figure S17. Normalized permeation flux for the CO_2 + O_2 + SO_2 + CO (Mix 1) mixture.	S280
Figure S18. Normalized permeation flux for the mixtures CO_2 + O_2 + SO_2 + CO (Mix 1), CO_2 + O_2, CO_2 + CO, CO_2 + CH_4 in the studied real reservoirs.	S281

Determination of the combined standard uncertainties in the compositions of the doped Mix 1 an doped Mix 2 used for the speed of sound measurements

The combined standard uncertainty values for the compositions of the doped mixtures obtained in this work are calculated according to the “Evaluation of Measurement Data–Guide to the Expression of Uncertainty in Measurement (GUM)”,[1] as suggested by the National Institute of Standards and Technology.

The $u(x_i)$ of each component in the doped mixtures was estimated using Eq. (1)[2] and is given in Table 3.

$$u(x_i) = \left[\frac{1}{\sum_{j=1}^{N} n_j}\right] \sqrt{(1-2x_i)u(n_i)^2 + x_i^2 \sum_{j=1}^{N} u(n_j)^2} \qquad (1)$$

where n_i is the number of moles of component i added to the mixture based on the change in the mass of the system. The masses were determined by successive weighing using the mass comparator. The standard uncertainty in the calculated number of moles ($u(n_i)$) of each substance was estimated as explained hereafter.

For the CO_2 + CH_3OH + O_2 + SO_2 + CO mixture (called doped Mix 1), the mole number of methanol (n_{CH_3OH}) was determined using Eq. (2):

$$n_{CH_3OH} = \frac{m_2 - m_1}{M_{CH_3OH}} \qquad (2)$$

where m_1 is the mass of the empty variable-volume cell, m_2 is the mass of the cell after the introduction of methanol, and M_{CH_3OH} is the molar mass of methanol. For the rest of the components in the mixture, the number of moles of each *i* component (n_i) was calculated using Eq. (3):

$$n_i = \frac{(m_3 - m_2)}{M_{\text{Mix 1}}} x_{i,\text{Mix 1}} \qquad (3)$$

where m_3 is the mass after Mix 1 is introduced into the variable-volume cell, $x_{i,\text{Mix 1}}$ is the mole fraction of each *i* component in Table 2, and $M_{\text{Mix 1}}$ is calculated as $\left[M_{CO_2} \cdot x_{CO_2,\text{Mix 1}} + M_{O_2} \cdot x_{O_2,\text{Mix 1}} + M_{SO_2} \cdot x_{SO_2,\text{Mix 1}} + M_{CO} \cdot x_{CO,\text{Mix 1}}\right]$ using the values of the mole fractions of Mix 1 taken from Table 2.

The standard uncertainty in the mole number of methanol ($u(n_{CH_3OH})$) was calculated from reference (1) using the equation (4):

$$u(n_{CH_3OH}) = \sqrt{\left(\frac{\partial n_{CH_3OH}}{\partial m_2}\right)^2 u(m_2)^2 + \left(\frac{\partial n_{CH_3OH}}{\partial m_1}\right)^2 u(m_1)^2} = \frac{1}{M_{CH_3OH}}\sqrt{u(m_2)^2 + u(m_1)^2} \quad (4)$$

For the rest of the components in doped Mix 1, the standard uncertainty in the mole number of each substance ($u(n_i)$) was calculated from reference (1) using the equation (5):

$$u(n_i) = \left\{\left(\frac{\partial n_i}{\partial m_2}\right)^2 u(m_2)^2 + \left(\frac{\partial n_i}{\partial m_3}\right)^2 u(m_3)^2 + \left(\frac{\partial n_i}{\partial x_{CO_2,\text{Mix 1}}}\right)^2 u\left(x_{CO_2,\text{Mix 1}}\right)^2 + \left(\frac{\partial n_i}{\partial x_{O_2,\text{Mix 1}}}\right)^2 u\left(x_{O_2,\text{Mix 1}}\right)^2 + \left(\frac{\partial n_i}{\partial x_{SO_2,\text{Mix 1}}}\right)^2 u\left(x_{SO_2,\text{Mix 1}}\right)^2 + + \left(\frac{\partial n_i}{\partial x_{CO,\text{Mix 1}}}\right)^2 u\left(x_{CO,\text{Mix 1}}\right)^2\right\}^{1/2} \quad (5)$$

In Eqs. (4) and (5), $u(m_1), u(m_2), and\ u(m_3)$ are the standard deviations of the repetitive mass measurements of the empty cell, the cell after methanol addition, and the cell after Mix 1 addition, respectively. $u\left(x_{CO_2,\text{Mix 1}}\right)$, $u\left(x_{O_2,\text{Mix 1}}\right)$, $u\left(x_{SO_2,\text{Mix 1}}\right), and\ u\left(x_{CO,\text{Mix 1}}\right)$ are the standard uncertainties of CO_2, O_2, SO_2, and CO mole fractions in Mix 1, respectively, which were calculated from the respective values of the expanded uncertainties given in Table 2. In

Eqs. (4) and (5), the contributions due to the uncertainty of the molar masses of the components of the doped mixture are considered negligible.

As an example, the application of Eq. (5) to calculate $u(n_{CO_2})$ results in Eq. (6):

$$u(n_{CO_2}) = \left\{ \left(\frac{x_{CO_2}}{M_{Mix\,1}} \right)^2 (u(m_2)^2 + u(m_3)^2) + \frac{(m_3 - m_2)^2 x_{CO_2,Mix\,1}{}^2}{M_{Mix\,1}{}^4} \left[M_{O_2}^2 u(x_{O_2,Mix\,1})^2 + M_{SO_2}^2 u(x_{SO_2,Mix\,1})^2 + M_{CO}^2 u(x_{CO,Mix\,1})^2 \right] + \frac{(m_3 - m_2)^2}{M_{Mix\,1}{}^4} (M_{Mix\,1} - M_{CO_2} x_{CO_2})^2 u(x_{CO_2,Mix\,1})^2 \right\}^{1/2} \quad (6)$$

Similar equations were used to estimate $u(n_{O_2}),\ u(n_{SO_2}), and\ u(n_{CO})$.

x_i and $u(x_i)$ for the CO_2 + CH_3OH + NO + SO_2 + CO mixture (called doped Mix 2) were obtained using the same method as that used for doped Mix 1, and they are shown in Table 3.

(1) JCGM 100:2008, 2008. GUM 1995 with minor corrections. *Evaluation of measurement data — Guide to the expression of uncertainty in measurement*. JCGM 2008. First edition 2008. Corrected version 2010. https://www.google.com/url?sa=t&source=web&rct=j&opi=89978449&url=https://www.bipm.org/documents/20126/2071204/JCGM_100_2008_E.pdf&ved=2ahUKEwj-ldeo6d2IAxXN9gIHHQ2IJq8QFnoECBMQAQ&usg=AOvVaw0y6DQ4RJkdZh6KI6wpTtxg (accessed 2024-12-11)

(2) Saif, Z.; Ghafri, A.; Czubinski, F. F.; May, E. F. *Fuel* **2018**, *231*, 187-96. DOI: 10.1016/j.fuel.2018.05.087.

Table S1. $p\rho T$ experimental data for the $CO_2 + O_2 + SO_2 + CO$ (Mix 1) and $CO_2 + NO + SO_2 + CO$ (Mix 2) mixtures. $u(\rho)$: combined standard uncertainty.

**Mix 1: x_{CO_2} = 0.96734; x_{O_2} = 0.030038; x_{SO_2} = 0.0009035; x_{CO} = 0.0017032**											
***T*= 263.18±0.02 K**			***T*= 273.19±0.01 K**			***T*= 283.20±0.02 K**			***T*= 293.13±0.02 K**		
***p* (MPa)**	**ρ (kg.m^{-3})**	**$u(\rho)$ (kg.m^{-3})**	***p* (MPa)**	**ρ (kg.m^{-3})**	**$u(\rho)$ (kg.m^{-3})**	***p* (MPa)**	**ρ (kg.m^{-3})**	**$u(\rho)$ (kg.m^{-3})**	***p* (MPa)**	**ρ (kg.m^{-3})**	**$u(\rho)$ (kg.m^{-3})**
0.100	1.95	0.22	0.100	1.84	0.22	0.100	1.82	0.22	0.100	1.74	0.22
0.111	2.16	0.22	0.113	2.08	0.22	0.115	2.06	0.22	0.117	2.05	0.22
0.122	2.41	0.22	0.127	2.36	0.22	0.130	2.35	0.22	0.133	2.35	0.22
0.133	2.65	0.22	0.140	2.62	0.22	0.145	2.66	0.22	0.150	2.66	0.22
0.144	2.89	0.22	0.153	2.87	0.22	0.160	2.92	0.22	0.166	2.96	0.22
0.156	3.13	0.22	0.166	3.14	0.22	0.175	3.24	0.22	0.183	3.27	0.22
0.167	3.36	0.22	0.180	3.36	0.22	0.190	3.51	0.22	0.200	3.55	0.22
0.178	3.59	0.22	0.193	3.62	0.22	0.205	3.75	0.22	0.216	3.83	0.22
0.189	3.81	0.22	0.206	3.90	0.22	0.220	4.09	0.22	0.233	4.14	0.22
0.200	4.03	0.22	0.219	4.19	0.22	0.235	4.38	0.22	0.250	4.45	0.22
0.211	4.25	0.22	0.233	4.43	0.22	0.250	4.66	0.22	0.266	4.74	0.22
0.222	4.47	0.22	0.246	4.68	0.22	0.265	4.93	0.22	0.283	5.05	0.22
0.233	4.69	0.22	0.259	4.98	0.22	0.280	5.22	0.22	0.299	5.34	0.22
0.244	4.96	0.22	0.273	5.27	0.22	0.295	5.50	0.22	0.316	5.68	0.22
0.256	5.19	0.22	0.286	5.52	0.22	0.310	5.78	0.22	0.333	6.01	0.22
0.267	5.41	0.22	0.299	5.76	0.22	0.325	6.06	0.22	0.349	6.31	0.22
0.278	5.61	0.22	0.312	6.04	0.22	0.340	6.40	0.22	0.366	6.61	0.22
0.289	5.85	0.22	0.326	6.31	0.22	0.354	6.69	0.22	0.382	6.90	0.22
0.300	6.10	0.22	0.339	6.59	0.22	0.369	6.97	0.22	0.399	7.21	0.22
0.311	6.34	0.22	0.352	6.86	0.22	0.384	7.25	0.22	0.416	7.56	0.22
0.322	6.57	0.22	0.366	7.08	0.22	0.399	7.53	0.22	0.432	7.85	0.22
0.333	6.77	0.22	0.379	7.34	0.22	0.414	7.81	0.22	0.449	8.14	0.22
0.344	7.01	0.22	0.392	7.62	0.22	0.429	8.10	0.22	0.466	8.44	0.22
0.356	7.26	0.22	0.405	7.90	0.22	0.444	8.44	0.22	0.482	8.81	0.22
0.367	7.51	0.22	0.419	8.18	0.22	0.459	8.70	0.22	0.499	9.11	0.22
0.378	7.73	0.22	0.432	8.46	0.22	0.474	9.01	0.22	0.515	9.42	0.22
0.389	7.93	0.22	0.445	8.75	0.22	0.489	9.30	0.22	0.532	9.72	0.22
0.400	8.18	0.22	0.458	8.98	0.22	0.504	9.57	0.22	0.549	10.03	0.22
0.411	8.43	0.22	0.472	9.24	0.22	0.519	9.85	0.22	0.565	10.33	0.22
0.422	8.69	0.22	0.485	9.53	0.22	0.534	10.19	0.22	0.582	10.68	0.22
0.433	8.94	0.22	0.498	9.83	0.22	0.549	10.46	0.22	0.598	11.00	0.22
0.445	9.19	0.22	0.512	10.07	0.22	0.564	10.73	0.22	0.615	11.30	0.22
0.456	9.39	0.22	0.525	10.34	0.22	0.579	11.06	0.22	0.632	11.64	0.22
0.467	9.63	0.22	0.538	10.63	0.22	0.594	11.36	0.22	0.648	11.98	0.22
0.478	9.88	0.22	0.551	10.93	0.22	0.609	11.66	0.22	0.665	12.27	0.22

0.489	10.08	0.22	0.565	11.18	0.22	0.624	11.98	0.22	0.682	12.57	0.22
0.500	10.32	0.22	0.578	11.46	0.22	0.639	12.26	0.22	0.698	12.88	0.22
0.511	10.58	0.22	0.591	11.74	0.22	0.654	12.54	0.22	0.715	13.23	0.22
0.522	10.83	0.22	0.604	11.98	0.22	0.669	12.88	0.22	0.731	13.53	0.22
0.533	11.08	0.22	0.618	12.26	0.22	0.684	13.17	0.22	0.748	13.83	0.22
0.545	11.34	0.22	0.631	12.55	0.22	0.699	13.44	0.22	0.765	14.20	0.22
0.556	11.54	0.22	0.644	12.84	0.22	0.714	13.74	0.22	0.781	14.50	0.22
0.567	11.77	0.22	0.658	13.13	0.22	0.729	14.05	0.22	0.798	14.79	0.22
0.578	12.02	0.22	0.671	13.42	0.22	0.744	14.33	0.22	0.814	15.16	0.22
0.589	12.26	0.22	0.684	13.70	0.22	0.759	14.67	0.22	0.831	15.47	0.22
0.600	12.51	0.22	0.697	13.97	0.22	0.774	15.01	0.22	0.848	15.83	0.22
0.611	12.76	0.22	0.711	14.24	0.22	0.789	15.29	0.22	0.864	16.15	0.22
0.622	13.01	0.22	0.724	14.51	0.22	0.804	15.61	0.22	0.881	16.45	0.22
0.633	13.25	0.22	0.737	14.78	0.22	0.819	15.91	0.22	0.898	16.75	0.22
0.645	13.50	0.22	0.751	15.05	0.22	0.834	16.20	0.22	0.914	17.10	0.22
0.656	13.75	0.22	0.764	15.32	0.22	0.849	16.53	0.22	0.931	17.44	0.22
0.667	14.00	0.22	0.777	15.60	0.22	0.863	16.79	0.22	0.947	17.77	0.22
0.678	14.25	0.22	0.790	15.93	0.22	0.878	17.12	0.22	0.964	18.10	0.22
0.689	14.50	0.22	0.804	16.23	0.22	0.893	17.41	0.22	0.981	18.40	0.22
0.700	14.76	0.22	0.817	16.51	0.22	0.908	17.72	0.22	0.997	18.74	0.22
0.711	14.98	0.22	0.830	16.79	0.22	0.923	18.06	0.22	1.014	19.03	0.22
0.722	15.18	0.22	0.843	17.07	0.22	0.938	18.38	0.22	1.030	19.39	0.22
0.733	15.44	0.22	0.857	17.35	0.22	0.953	18.65	0.22	1.047	19.76	0.22
0.745	15.69	0.22	0.870	17.64	0.22	0.968	18.99	0.22	1.064	20.07	0.22
0.756	15.94	0.22	0.883	17.92	0.22	0.983	19.32	0.22	1.080	20.42	0.22
0.767	16.19	0.22	0.897	18.21	0.22	0.998	19.61	0.22	1.097	20.74	0.22
0.778	16.44	0.22	0.910	18.50	0.22	1.013	19.92	0.22	1.114	21.09	0.22
0.789	16.69	0.22	0.923	18.79	0.22	1.028	20.26	0.22	1.130	21.43	0.22
0.800	16.95	0.22	0.936	19.08	0.22	1.043	20.59	0.22	1.147	21.73	0.22
0.811	17.20	0.22	0.950	19.37	0.22	1.058	20.88	0.22	1.163	22.06	0.22
0.822	17.46	0.22	0.963	19.65	0.22	1.073	21.23	0.22	1.180	22.39	0.22
0.833	17.71	0.22	0.976	19.93	0.22	1.088	21.52	0.22	1.197	22.71	0.22
0.845	17.97	0.22	0.989	20.25	0.22	1.103	21.80	0.22	1.213	23.09	0.22
0.856	18.23	0.22	1.003	20.57	0.22	1.118	22.14	0.22	1.230	23.44	0.22
0.867	18.48	0.22	1.016	20.85	0.22	1.133	22.45	0.22	1.246	23.75	0.22
0.878	18.74	0.22	1.029	21.13	0.22	1.148	22.75	0.22	1.263	24.12	0.22
0.889	19.00	0.22	1.043	21.41	0.22	1.163	23.09	0.22	1.280	24.42	0.22
0.900	19.26	0.22	1.056	21.70	0.22	1.178	23.43	0.22	1.296	24.79	0.22
0.911	19.52	0.22	1.069	21.99	0.22	1.193	23.77	0.22	1.313	25.17	0.22
0.922	19.77	0.22	1.082	22.27	0.22	1.208	24.05	0.22	1.330	25.48	0.22
0.933	20.03	0.22	1.096	22.58	0.22	1.223	24.37	0.22	1.346	25.85	0.22
0.945	20.28	0.22	1.109	22.93	0.22	1.238	24.69	0.22	1.363	26.17	0.22
0.956	20.53	0.22	1.122	23.22	0.22	1.253	25.01	0.22	1.379	26.55	0.22
0.967	20.78	0.22	1.135	23.50	0.22	1.268	25.33	0.22	1.396	26.87	0.22
0.978	21.02	0.22	1.149	23.77	0.22	1.283	25.65	0.22	1.413	27.24	0.22

0.989	21.30	0.22	1.162	24.09	0.22	1.298	26.02	0.22	1.429	27.57	0.22
1.000	21.59	0.22	1.175	24.42	0.22	1.313	26.37	0.22	1.446	27.87	0.22
1.011	21.83	0.22	1.189	24.73	0.22	1.328	26.70	0.22	1.462	28.22	0.22
1.022	22.08	0.22	1.202	25.00	0.22	1.343	27.03	0.22	1.479	28.57	0.22
1.033	22.32	0.22	1.215	25.31	0.22	1.357	27.35	0.22	1.496	28.93	0.22
1.045	22.61	0.22	1.228	25.62	0.22	1.372	27.69	0.22	1.512	29.29	0.22
1.056	22.90	0.22	1.242	25.89	0.22	1.387	28.03	0.22	1.529	29.66	0.22
1.067	23.14	0.22	1.255	26.21	0.22	1.402	28.32	0.22	1.546	30.02	0.22
1.078	23.39	0.22	1.268	26.54	0.22	1.417	28.63	0.22	1.562	30.38	0.22
1.089	23.64	0.22	1.282	26.87	0.22	1.432	28.97	0.22	1.579	30.68	0.22
1.100	23.94	0.22	1.295	27.15	0.22	1.447	29.30	0.22	1.595	31.06	0.22
1.111	24.22	0.22	1.308	27.44	0.22	1.462	29.64	0.22	1.612	31.44	0.22
1.122	24.48	0.22	1.321	27.72	0.22	1.477	29.97	0.22	1.629	31.81	0.22
1.134	24.73	0.22	1.335	28.06	0.22	1.492	30.31	0.22	1.645	32.16	0.22
1.145	24.99	0.22	1.348	28.37	0.22	1.507	30.64	0.22	1.662	32.51	0.22
1.156	25.24	0.22	1.361	28.67	0.22	1.522	30.99	0.22	1.678	32.87	0.22
1.167	25.53	0.22	1.374	29.01	0.22	1.537	31.33	0.22	1.695	33.22	0.22
1.178	25.83	0.22	1.388	29.29	0.22	1.552	31.68	0.22	1.712	33.58	0.22
1.189	26.08	0.22	1.401	29.59	0.22	1.567	32.03	0.22	1.728	33.94	0.22
1.200	26.32	0.22	1.414	29.91	0.22	1.582	32.37	0.22	1.745	34.31	0.22
1.211	26.57	0.22	1.428	30.23	0.22	1.597	32.72	0.22	1.762	34.68	0.22
1.222	26.86	0.22	1.441	30.55	0.22	1.612	33.02	0.22	1.778	35.04	0.22
1.234	27.15	0.22	1.454	30.88	0.22	1.627	33.34	0.22	1.795	35.40	0.22
1.245	27.44	0.22	1.467	31.20	0.22	1.642	33.70	0.22	1.811	35.76	0.22
1.256	27.71	0.22	1.481	31.53	0.22	1.657	34.08	0.22	1.828	36.11	0.22
1.267	27.95	0.22	1.494	31.85	0.22	1.672	34.41	0.22	1.845	36.52	0.22
1.278	28.25	0.22	1.507	32.18	0.22	1.687	34.75	0.22	1.861	36.89	0.22
1.289	28.53	0.22	1.520	32.48	0.22	1.702	35.09	0.22	1.878	37.28	0.22
1.300	28.78	0.22	1.534	32.77	0.22	1.717	35.43	0.22	1.894	37.68	0.22
1.311	29.07	0.22	1.547	33.10	0.22	1.732	35.77	0.22	1.911	38.04	0.22
1.322	29.36	0.22	1.560	33.44	0.22	1.747	36.14	0.22	1.928	38.41	0.22
1.334	29.62	0.22	1.574	33.78	0.22	1.762	36.53	0.22	1.944	38.78	0.22
1.345	29.91	0.22	1.587	34.12	0.22	1.777	36.88	0.22	1.961	39.16	0.22
1.356	30.21	0.22	1.600	34.41	0.22	1.792	37.22	0.22	1.978	39.55	0.22
1.367	30.46	0.22	1.613	34.73	0.22	1.807	37.56	0.22	1.994	40.01	0.22
1.378	30.72	0.22	1.627	35.08	0.22	1.822	37.94	0.22	2.011	40.40	0.22
1.389	30.99	0.22	1.640	35.43	0.22	1.837	38.30	0.22	2.027	40.78	0.22
1.400	31.30	0.22	1.653	35.71	0.22	1.851	38.63	0.22	2.044	41.11	0.22
1.411	31.59	0.22	1.667	36.01	0.22	1.866	38.98	0.22	2.061	41.50	0.22
1.422	31.85	0.22	1.680	36.34	0.22	1.881	39.39	0.22	2.077	41.88	0.22
1.434	32.16	0.22	1.693	36.67	0.22	1.896	39.73	0.22	2.094	42.27	0.22
1.445	32.44	0.22	1.706	37.00	0.22	1.911	40.07	0.22	2.110	42.63	0.22
1.456	32.70	0.22	1.720	37.33	0.22	1.926	40.45	0.22	2.127	43.06	0.22
1.467	33.00	0.22	1.733	37.67	0.22	1.941	40.81	0.22	2.144	43.43	0.22
1.478	33.29	0.22	1.746	38.01	0.22	1.956	41.15	0.22	2.160	43.84	0.22

1.489	33.54	0.22	1.759	38.34	0.22	1.971	41.54	0.22	2.177	44.24	0.22
1.500	33.83	0.22	1.773	38.69	0.22	1.986	41.87	0.22	2.194	44.60	0.22
1.511	34.14	0.22	1.786	39.03	0.22	2.001	42.26	0.22	2.210	44.98	0.22
1.522	34.45	0.22	1.799	39.37	0.22	2.016	42.62	0.22	2.227	45.38	0.22
1.534	34.72	0.22	1.813	39.72	0.22	2.031	42.99	0.22	2.243	45.81	0.22
1.545	34.98	0.22	1.826	40.07	0.22	2.046	43.38	0.22	2.260	46.19	0.22
1.556	35.29	0.22	1.839	40.42	0.22	2.061	43.73	0.22	2.277	46.58	0.22
1.567	35.59	0.22	1.852	40.76	0.22	2.076	44.14	0.22	2.293	46.97	0.22
1.578	35.90	0.22	1.866	41.09	0.22	2.091	44.48	0.22	2.310	47.36	0.22
1.589	36.21	0.22	1.879	41.42	0.22	2.106	44.88	0.22	2.326	47.75	0.22
1.600	36.51	0.22	1.892	41.78	0.22	2.121	45.24	0.22	2.343	48.15	0.22
1.611	36.81	0.22	1.905	42.17	0.22	2.136	45.61	0.22	2.360	48.59	0.22
1.622	37.09	0.22	1.919	42.50	0.22	2.151	46.00	0.22	2.376	48.97	0.22
1.634	37.38	0.22	1.932	42.83	0.22	2.166	46.40	0.22	2.393	49.36	0.22
1.645	37.66	0.22	1.945	43.17	0.22	2.181	46.74	0.22	2.410	49.74	0.22
1.656	37.94	0.22	1.959	43.51	0.22	2.196	47.15	0.22	2.426	50.19	0.22
1.667	38.22	0.22	1.972	43.85	0.22	2.211	47.51	0.22	2.443	50.56	0.22
1.678	38.53	0.22	1.985	44.22	0.22	2.226	47.88	0.22	2.459	50.99	0.22
1.689	38.85	0.22	1.998	44.62	0.22	2.241	48.27	0.22	2.476	51.41	0.22
1.700	39.13	0.22	2.012	44.96	0.22	2.256	48.66	0.22	2.493	51.78	0.22
1.711	39.42	0.22	2.025	45.31	0.22	2.271	49.05	0.22	2.509	52.18	0.22
1.722	39.75	0.22	2.038	45.66	0.22	2.286	49.44	0.22	2.526	52.61	0.22
1.734	40.05	0.22	2.051	46.01	0.22	2.301	49.84	0.22	2.542	53.05	0.22
1.745	40.33	0.22	2.065	46.34	0.22	2.316	50.24	0.22	2.559	53.46	0.22
1.756	40.66	0.22	2.078	46.70	0.22	2.331	50.62	0.22	2.576	53.83	0.22
1.767	40.99	0.22	2.091	47.09	0.22	2.345	50.98	0.22	2.592	54.24	0.22
1.778	41.32	0.22	2.105	47.48	0.22	2.360	51.39	0.22	2.609	54.66	0.22
1.789	41.62	0.22	2.118	47.83	0.22	2.375	51.81	0.22	2.626	55.07	0.22
1.800	41.90	0.22	2.131	48.18	0.22	2.390	52.21	0.22	2.642	55.49	0.22
1.811	42.22	0.22	2.144	48.57	0.22	2.405	52.61	0.22	2.659	55.92	0.22
1.823	42.55	0.22	2.158	48.92	0.22	2.420	53.01	0.22	2.675	56.35	0.22
1.834	42.88	0.22	2.171	49.28	0.22	2.435	53.39	0.22	2.692	56.78	0.22
1.845	43.20	0.22	2.184	49.68	0.22	2.450	53.74	0.22	2.709	57.22	0.22
1.856	43.48	0.22	2.198	50.03	0.22	2.465	54.18	0.22	2.725	57.64	0.22
1.867	43.81	0.22	2.211	50.41	0.22	2.480	54.57	0.22	2.742	58.02	0.22
1.878	44.14	0.22	2.224	50.80	0.22	2.495	54.95	0.22	2.759	58.47	0.22
1.889	44.43	0.22	2.237	51.15	0.22	2.510	55.36	0.22	2.775	58.92	0.22
1.900	44.75	0.22	2.251	51.50	0.22	2.525	55.81	0.22	2.792	59.37	0.22
1.911	45.10	0.22	2.264	51.86	0.22	2.540	56.20	0.22	2.808	59.76	0.22
1.923	45.44	0.22	2.277	52.25	0.22	2.555	56.61	0.22	2.825	60.20	0.22
1.934	45.76	0.22	2.290	52.64	0.22	2.570	57.01	0.22	2.842	60.64	0.22
1.945	46.06	0.22	2.304	53.03	0.22	2.585	57.42	0.22	2.858	61.08	0.22
1.956	46.38	0.22	2.317	53.42	0.22	2.600	57.83	0.22	2.875	61.52	0.22
1.967	46.72	0.22	2.330	53.81	0.22	2.615	58.25	0.22	2.891	61.97	0.22
1.978	47.02	0.22	2.344	54.16	0.22	2.630	58.67	0.22	2.908	62.42	0.22

1.989	47.33	0.22	2.357	54.53	0.22	2.645	59.09	0.22	2.925	62.82	0.22
2.000	47.67	0.22	2.370	54.93	0.22	2.660	59.46	0.22	2.941	63.23	0.22
2.011	48.01	0.22	2.383	55.33	0.22	2.675	59.91	0.22	2.958	63.70	0.22
2.023	48.35	0.22	2.397	55.73	0.22	2.690	60.36	0.22	2.975	64.15	0.22
2.034	48.68	0.22	2.410	56.13	0.22	2.705	60.76	0.22	2.991	64.56	0.22
2.045	49.02	0.22	2.423	56.48	0.22	2.720	61.22	0.22	3.008	65.06	0.22
2.056	49.35	0.22	2.436	56.86	0.22	2.735	61.62	0.22	3.024	65.50	0.22
2.067	49.69	0.22	2.450	57.25	0.22	2.750	62.01	0.22	3.041	65.95	0.22
2.078	50.03	0.22	2.463	57.64	0.22	2.765	62.41	0.22	3.058	66.40	0.22
2.089	50.37	0.22	2.476	58.08	0.22	2.780	62.86	0.22	3.074	66.85	0.22
2.100	50.71	0.22	2.490	58.49	0.22	2.795	63.30	0.22	3.091	67.31	0.22
2.111	51.05	0.22	2.503	58.89	0.22	2.810	63.71	0.22	3.107	67.78	0.22
2.123	51.39	0.22	2.516	59.28	0.22	2.825	64.13	0.22	3.124	68.17	0.22
2.134	51.73	0.22	2.529	59.68	0.22	2.839	64.60	0.22	3.141	68.64	0.22
2.145	52.06	0.22	2.543	60.08	0.22	2.854	65.01	0.22	3.157	69.11	0.22
2.156	52.40	0.22	2.556	60.48	0.22	2.869	65.48	0.22	3.174	69.58	0.22
2.167	52.73	0.22	2.569	60.88	0.22	2.884	65.92	0.22	3.191	70.04	0.22
2.178	53.07	0.22	2.583	61.28	0.22	2.899	66.34	0.22	3.207	70.49	0.22
2.189	53.42	0.22	2.596	61.70	0.22	2.914	66.78	0.22	3.224	70.93	0.22
2.200	53.80	0.22	2.609	62.19	0.22	2.929	67.24	0.22	3.240	71.43	0.22
2.211	54.16	0.22	2.622	62.56	0.22	2.944	67.65	0.22	3.257	71.90	0.22
2.223	54.50	0.22	2.636	62.93	0.22	2.959	68.13	0.22	3.274	72.35	0.22
2.234	54.85	0.22	2.649	63.37	0.22	2.974	68.55	0.22	3.290	72.84	0.22
2.245	55.21	0.22	2.662	63.77	0.22	2.989	68.99	0.22	3.307	73.33	0.22
2.256	55.61	0.22	2.675	64.16	0.22	3.004	69.46	0.22	3.323	73.79	0.22
2.267	55.98	0.22	2.689	64.56	0.22	3.019	69.92	0.22	3.340	74.25	0.22
2.278	56.32	0.22	2.702	65.07	0.22	3.034	70.38	0.22	3.357	74.73	0.22
2.289	56.62	0.22	2.715	65.55	0.22	3.049	70.79	0.22	3.373	75.24	0.22
2.300	57.00	0.22	2.729	66.00	0.22	3.064	71.26	0.22	3.390	75.69	0.22
2.311	57.45	0.22	2.742	66.40	0.22	3.079	71.74	0.22	3.407	76.15	0.22
2.323	57.80	0.22	2.755	66.83	0.22	3.094	72.22	0.22	3.423	76.66	0.22
2.334	58.17	0.22	2.768	67.28	0.22	3.109	72.67	0.22	3.440	77.10	0.22
2.345	58.56	0.22	2.782	67.72	0.22	3.124	73.09	0.22	3.456	77.60	0.22
2.356	58.92	0.22	2.795	68.16	0.22	3.139	73.54	0.22	3.473	78.11	0.22
2.367	59.27	0.22	2.808	68.58	0.22	3.154	74.00	0.22	3.490	78.56	0.22
2.378	59.61	0.22	2.821	68.98	0.22	3.169	74.51	0.22	3.506	79.06	0.22
2.389	59.99	0.22	2.835	69.43	0.22	3.184	75.00	0.22	3.523	79.58	0.22
2.400	60.40	0.22	2.848	69.88	0.22	3.199	75.46	0.22	3.539	80.11	0.22
2.412	60.75	0.22	2.861	70.34	0.22	3.214	75.93	0.22	3.556	80.59	0.22
2.423	61.12	0.22	2.875	70.80	0.22	3.229	76.41	0.22	3.573	81.08	0.22
2.434	61.51	0.22	2.888	71.21	0.22	3.244	76.89	0.22	3.589	81.60	0.22
2.445	61.88	0.22	2.901	71.66	0.22	3.259	77.38	0.22	3.606	82.10	0.22
2.456	62.24	0.22	2.914	72.12	0.22	3.274	77.81	0.22	3.623	82.58	0.22
2.467	62.64	0.22	2.928	72.59	0.22	3.289	78.27	0.22	3.639	83.10	0.22
2.478	63.04	0.22	2.941	73.04	0.22	3.304	78.80	0.22	3.656	83.61	0.22

2.489	63.44	0.22	2.954	73.50	0.22	3.319	79.30	0.22	3.672	84.11	0.22
2.500	63.79	0.22	2.968	73.96	0.22	3.334	79.74	0.22	3.689	84.62	0.22
2.512	64.15	0.22	2.981	74.43	0.22	3.348	80.28	0.22	3.706	85.14	0.22
2.523	64.54	0.22	2.994	74.89	0.22	3.363	80.75	0.22	3.722	85.66	0.22
2.534	64.94	0.22	3.007	75.35	0.22	3.378	81.23	0.22	3.739	86.19	0.22
2.545	65.34	0.22	3.021	75.80	0.22	3.393	81.76	0.22	3.755	86.72	0.22
2.556	65.74	0.22	3.034	76.24	0.22	3.408	82.26	0.22	3.772	87.19	0.22
2.567	66.14	0.22	3.047	76.71	0.22	3.423	82.75	0.22	3.789	87.71	0.22
2.578	66.54	0.22	3.060	77.20	0.22	3.438	83.26	0.22	3.805	88.24	0.22
2.589	66.95	0.22	3.074	77.65	0.22	3.453	83.73	0.22	3.822	88.77	0.22
2.600	67.35	0.22	3.087	78.13	0.22	3.468	84.26	0.22	3.839	89.32	0.22
2.612	67.75	0.22	3.100	78.63	0.22	3.483	84.80	0.22	3.855	89.86	0.22
2.623	68.15	0.22	3.114	79.08	0.22	3.498	85.30	0.22	3.872	90.37	0.22
2.634	68.55	0.22	3.127	79.59	0.22	3.513	85.80	0.22	3.888	90.90	0.22
2.645	68.95	0.22	3.140	80.08	0.22	3.528	86.33	0.22	3.905	91.47	0.22
2.656	69.38	0.22	3.153	80.54	0.22	3.543	86.83	0.22	3.922	91.99	0.22
2.667	69.83	0.22	3.167	81.07	0.22	3.558	87.38	0.22	3.938	92.52	0.22
2.678	70.22	0.22	3.180	81.56	0.22	3.573	87.88	0.22	3.955	93.12	0.22
2.689	70.64	0.22	3.193	82.04	0.22	3.588	88.38	0.22	3.971	93.64	0.22
2.700	71.09	0.22	3.206	82.52	0.22	3.603	88.95	0.22	3.988	94.16	0.22
2.712	71.51	0.22	3.220	83.06	0.22	3.618	89.48	0.22	4.005	94.74	0.22
2.723	71.93	0.22	3.233	83.55	0.22	3.633	90.01	0.22	4.021	95.27	0.22
2.734	72.37	0.22	3.246	84.03	0.22	3.648	90.57	0.22	4.038	95.86	0.22
			3.260	84.56	0.22	3.663	91.12	0.22	4.055	96.42	0.22
			3.273	85.05	0.22	3.678	91.63	0.22	4.071	97.01	0.22
			3.286	85.56	0.22	3.693	92.20	0.22	4.088	97.59	0.22
			3.299	86.10	0.22	3.708	92.70	0.22	4.104	98.13	0.22
			3.313	86.59	0.22	3.723	93.26	0.22	4.121	98.67	0.22
			3.326	87.10	0.22	3.738	93.82	0.22	4.138	99.24	0.22
			3.339	87.65	0.22	3.753	94.36	0.22	4.154	99.84	0.22
			3.352	88.21	0.22	3.768	94.90	0.22	4.171	100.43	0.22
			3.366	88.73	0.22	3.783	95.46	0.22	4.187	101.00	0.22
			3.379	89.24	0.22	3.798	96.06	0.22	4.204	101.55	0.22
			3.392	89.81	0.22	3.813	96.61	0.22	4.221	102.14	0.22
			3.406	90.34	0.22	3.828	97.16	0.22	4.237	102.73	0.22
			3.419	90.88	0.22	3.842	97.77	0.22	4.254	103.32	0.22
			3.432	91.46	0.22	3.857	98.34	0.22	4.271	103.91	0.22
			3.445	91.99	0.22	3.872	98.93	0.22	4.287	104.51	0.22
			3.459	92.55	0.22	3.887	99.52	0.22	4.304	105.12	0.22
			3.472	93.11	0.22	3.902	100.07	0.22	4.320	105.73	0.22
			3.485	93.62	0.22	3.917	100.66	0.22	4.337	106.33	0.22
			3.499	94.21	0.22	3.932	101.26	0.22	4.354	106.94	0.22
			3.512	94.78	0.22	3.947	101.86	0.22	4.370	107.55	0.22
			3.525	95.33	0.22	3.962	102.47	0.22	4.387	108.16	0.22
			3.538	95.94	0.22	3.977	103.08	0.22	4.403	108.77	0.22

			3.552	96.53	0.22	3.992	103.69	0.22	4.420	109.38	0.22
			3.565	97.12	0.22	4.007	104.31	0.22	4.437	110.03	0.22
			3.578	97.74	0.22	4.022	104.93	0.22	4.453	110.67	0.22
			3.591	98.34	0.22	4.037	105.48	0.22	4.470	111.29	0.22
			3.605	98.94	0.22	4.052	106.12	0.22	4.487	111.92	0.22
			3.618	99.55	0.22	4.067	106.76	0.22	4.503	112.56	0.22
						4.082	107.36	0.22	4.520	113.20	0.22
						4.097	107.99	0.22	4.536	113.84	0.22
						4.112	108.67	0.22	4.553	114.48	0.22
						4.127	109.30	0.22	4.570	115.11	0.22
						4.142	109.93	0.22	4.586	115.77	0.22
						4.157	110.60	0.22	4.603	116.43	0.22
						4.172	111.23	0.22	4.619	117.09	0.22
						4.187	111.85	0.22	4.636	117.77	0.22
						4.202	112.53	0.22	4.653	118.44	0.22
						4.217	113.20	0.22	4.669	119.13	0.22
						4.232	113.88	0.22	4.686	119.81	0.22
						4.247	114.55	0.22	4.703	120.48	0.22
						4.262	115.23	0.22	4.719	121.14	0.22
						4.277	115.90	0.22	4.736	121.84	0.22
						4.292	116.58	0.22	4.752	122.56	0.22
						4.307	117.28	0.22	4.769	123.25	0.22
						4.322	118.03	0.22	4.786	123.91	0.22
						4.336	118.72	0.22	4.802	124.62	0.22
						4.351	119.41	0.22	4.819	125.32	0.22
						4.366	120.12	0.22	4.835	126.03	0.22
						4.381	120.86	0.22	4.852	126.76	0.22
						4.396	121.61	0.22	4.869	127.49	0.22
						4.411	122.34	0.22	4.885	128.20	0.22
						4.426	123.08	0.22	4.902	128.99	0.22
						4.441	123.79	0.22	4.919	129.69	0.22
						4.456	124.56	0.22	4.935	130.47	0.22
						4.471	125.33	0.22	4.952	131.24	0.22
						4.486	126.10	0.22	4.968	131.94	0.22
						4.501	126.85	0.22	4.985	132.71	0.22
						4.516	127.61	0.21	5.002	133.49	0.22
						4.531	128.40	0.21	5.018	134.26	0.22
						4.546	129.22	0.21	5.035	135.03	0.22
						4.561	130.01	0.21	5.051	135.81	0.22
						4.576	130.80	0.21	5.068	136.60	0.22
						4.591	131.60	0.21	5.085	137.39	0.22
						4.606	132.41	0.21	5.101	138.18	0.22
						4.621	133.28	0.21	5.118	138.94	0.22
						4.636	134.10	0.21	5.135	139.77	0.22
						4.651	134.91	0.21	5.151	140.58	0.22

						4.666	135.74	0.21	5.168	141.39	0.22
						4.681	136.57	0.21	5.184	142.21	0.22
						4.696	137.52	0.21	5.201	143.04	0.22
									5.218	143.88	0.22
									5.234	144.74	0.22
									5.251	145.61	0.22
									5.267	146.50	0.22
									5.284	147.36	0.22
									5.301	148.21	0.22
									5.317	149.09	0.22
									5.334	149.97	0.22
									5.351	150.84	0.22
									5.367	151.75	0.22
									5.384	152.70	0.22
									5.400	153.60	0.22
									5.417	154.53	0.22
									5.434	155.47	0.22
									5.450	156.47	0.22
									5.467	157.42	0.22
									5.483	158.37	0.22
									5.500	159.38	0.22
									5.517	160.35	0.22
									5.533	161.37	0.22
									5.550	162.39	0.22
									5.567	163.36	0.22
									5.583	164.42	0.22
									5.600	165.48	0.22
									5.616	166.55	0.22
									5.633	167.60	0.22
									5.650	168.62	0.22
									5.666	169.74	0.22
									5.683	170.87	0.22
									5.699	171.98	0.22
									5.716	173.12	0.22
									5.733	174.23	0.21
									5.749	175.35	0.21
									5.766	176.50	0.21
									5.783	177.69	0.21
									5.799	178.90	0.21
									5.816	180.13	0.21
									5.832	181.40	0.21
									5.849	182.64	0.21
									5.866	183.87	0.21
									5.882	184.96	0.21
									5.899	186.24	0.21

									5.915	187.69	0.21
									5.932	189.11	0.21
									5.949	190.45	0.21
									5.965	191.91	0.21
									5.982	193.29	0.21
									5.999	194.75	0.21
									6.015	196.29	0.21
									6.032	197.81	0.21

Combined standard uncertainties:

$u(T)$= 0.006 K; $u(p)$= 0.0020 MPa for p < 6 MPa; $u(p)$= 0.024 MPa for 6 MPa ≤ p ≤ 70 MPa

$u(x_{CO_2})$= 0.00024; $u(x_{O_2})$= 0.000030; $u(x_{SO_2})$= 0.0000023; $u(x_{CO})$= 0.0000043

Table S1 (continued). $p\rho T$ experimental data for the CO_2 + O_2 + SO_2 + CO (Mix 1) and CO_2 + NO + SO_2 + CO (Mix 2) mixtures. $u(\rho)$: combined standard uncertainty.

**Mix 1: x_{CO_2} = 0.96734; x_{O_2} = 0.030038; x_{SO_2} = 0.0009035; x_{CO} = 0.0017032**											
***T*= 263.14±0.02 K**			***T*= 273.10±0.01 K**			***T*= 283.22±0.02 K**			***T*= 293.13±0.02 K**		
***p* (MPa)**	**ρ ($kg.m^{-3}$)**	**$u(\rho)$ ($kg.m^{-3}$)**	***p* (MPa)**	**ρ ($kg.m^{-3}$)**	**$u(\rho)$ ($kg.m^{-3}$)**	***p* (MPa)**	**ρ ($kg.m^{-3}$)**	**$u(\rho)$ ($kg.m^{-3}$)**	***p* (MPa)**	**ρ ($kg.m^{-3}$)**	**$u(\rho)$ ($kg.m^{-3}$)**
4.103	961.17	0.37	4.910	903.43	0.36	5.869	834.26	0.34	7.056	745.06	0.32
4.124	961.31	0.37	4.930	903.75	0.36	5.889	834.60	0.34	7.077	746.10	0.32
4.145	961.45	0.37	4.951	904.01	0.36	5.910	834.93	0.34	7.097	746.79	0.32
4.166	961.59	0.37	4.971	904.20	0.36	5.930	835.25	0.34	7.117	747.49	0.32
4.187	961.73	0.37	4.992	904.39	0.36	5.951	835.56	0.34	7.137	748.19	0.32
4.208	961.87	0.37	5.012	904.58	0.36	5.972	835.88	0.34	7.158	749.01	0.32
4.229	962.02	0.37	5.033	904.77	0.36	5.992	836.19	0.34	7.178	749.89	0.32
4.250	962.16	0.37	5.054	904.94	0.36	6.013	836.51	0.34	7.198	750.71	0.32
4.271	962.30	0.37	5.074	905.07	0.36	6.033	836.82	0.34	7.218	751.45	0.32
4.291	962.44	0.37	5.095	905.42	0.36	6.054	837.13	0.34	7.239	752.18	0.32
4.312	962.58	0.37	5.115	905.64	0.36	6.074	837.45	0.34	7.259	752.94	0.32
4.333	962.72	0.37	5.136	905.83	0.36	6.095	837.76	0.34	7.279	753.65	0.32
4.354	962.87	0.37	5.156	906.00	0.36	6.115	838.08	0.34	7.299	754.28	0.32
4.375	963.01	0.37	5.177	906.20	0.36	6.136	838.39	0.34	7.320	754.93	0.32
4.396	963.15	0.37	5.197	906.41	0.36	6.156	838.71	0.34	7.340	755.68	0.32
4.417	963.29	0.37	5.218	906.60	0.36	6.177	839.02	0.34	7.360	756.39	0.32
4.438	963.43	0.37	5.239	906.78	0.36	6.197	839.34	0.34	7.380	757.02	0.32
4.459	963.57	0.37	5.259	906.96	0.36	6.218	839.65	0.34	7.401	757.66	0.32
4.480	963.72	0.37	5.280	907.19	0.36	6.238	839.96	0.34	7.421	758.36	0.32
4.501	963.86	0.37	5.300	907.39	0.36	6.259	840.28	0.34	7.441	759.05	0.32
4.522	964.00	0.37	5.321	907.56	0.36	6.279	840.59	0.34	7.462	759.66	0.32
4.543	964.14	0.37	5.341	907.80	0.36	6.300	840.91	0.34	7.482	760.36	0.32
4.564	964.28	0.37	5.362	907.99	0.36	6.320	841.22	0.34	7.502	760.98	0.32
4.585	964.42	0.37	5.382	908.17	0.36	6.341	841.54	0.34	7.522	761.58	0.32
4.606	964.57	0.37	5.403	908.41	0.36	6.361	841.85	0.34	7.543	762.23	0.32
4.627	964.71	0.37	5.424	908.60	0.36	6.382	842.17	0.34	7.563	762.89	0.32
4.648	964.85	0.37	5.444	908.78	0.36	6.402	842.48	0.34	7.583	763.57	0.32
4.668	964.99	0.37	5.465	908.96	0.36	6.423	842.80	0.34	7.603	764.24	0.32
4.689	965.13	0.37	5.485	909.14	0.36	6.443	843.11	0.34	7.624	764.88	0.32
4.710	965.27	0.37	5.506	909.32	0.36	6.464	843.42	0.34	7.644	765.49	0.32
4.731	965.42	0.37	5.526	909.51	0.36	6.484	843.74	0.34	7.664	766.10	0.32
4.752	965.56	0.37	5.547	909.69	0.36	6.505	844.05	0.34	7.684	766.73	0.32
4.773	965.70	0.37	5.568	909.90	0.36	6.525	844.37	0.34	7.705	767.26	0.32
4.794	965.84	0.37	5.588	910.10	0.36	6.546	844.68	0.34	7.725	767.86	0.32
4.815	965.98	0.37	5.609	910.27	0.36	6.566	844.99	0.34	7.745	768.43	0.32

4.836	966.12	0.37	5.629	910.43	0.36	6.587	845.28	0.34	7.765	768.96	0.32
4.857	966.26	0.37	5.650	910.58	0.36	6.607	845.64	0.34	7.786	769.47	0.32
4.878	966.41	0.37	5.670	910.72	0.36	6.628	845.94	0.34	7.806	770.01	0.32
4.899	966.55	0.37	5.691	910.85	0.36	6.648	846.24	0.34	7.826	770.55	0.32
4.920	966.74	0.37	5.711	910.96	0.36	6.669	846.60	0.34	7.846	771.06	0.32
4.941	966.86	0.37	5.732	911.22	0.36	6.689	846.90	0.34	7.867	771.67	0.32
4.962	966.97	0.37	5.753	911.65	0.36	6.710	847.20	0.34	7.887	772.25	0.32
4.983	967.14	0.37	5.773	911.85	0.36	6.730	847.56	0.34	7.907	772.80	0.32
5.004	967.27	0.37	5.794	912.05	0.36	6.751	847.87	0.34	7.927	773.35	0.32
5.025	967.40	0.37	5.814	912.25	0.36	6.771	848.18	0.34	7.948	773.87	0.32
5.045	967.59	0.37	5.835	912.45	0.36	6.792	848.49	0.34	7.968	774.37	0.32
5.066	967.71	0.37	5.855	912.64	0.36	6.812	848.80	0.34	7.988	774.93	0.32
5.087	967.78	0.37	5.876	912.83	0.36	6.833	849.11	0.34	8.008	775.52	0.32
5.108	968.01	0.37	5.896	913.02	0.36	6.853	849.41	0.34	8.029	776.02	0.32
5.129	968.23	0.37	5.917	913.21	0.36	6.874	849.70	0.34	8.049	776.57	0.32
5.150	968.31	0.37	5.938	913.40	0.36	6.894	849.99	0.34	8.069	777.14	0.32
5.171	968.33	0.37	5.958	913.59	0.36	6.915	850.35	0.34	8.089	777.67	0.32
5.192	968.65	0.37	5.979	913.78	0.36	6.935	850.67	0.34	8.110	778.15	0.32
5.213	968.81	0.37	5.999	913.97	0.36	6.956	850.97	0.34	8.130	778.62	0.32
5.234	968.95	0.37	6.020	914.16	0.36	6.976	851.26	0.34	8.150	779.13	0.32
5.255	969.13	0.37	6.040	914.35	0.36	6.997	851.54	0.34	8.170	779.66	0.33
5.276	969.31	0.37	6.061	914.54	0.36	7.017	851.82	0.34	8.191	780.15	0.33
5.297	969.39	0.37	6.081	914.75	0.36	7.038	852.12	0.34	8.211	780.61	0.33
5.318	969.57	0.37	6.102	914.93	0.36	7.059	852.41	0.34	8.231	781.09	0.33
5.339	969.67	0.37	6.123	915.11	0.36	7.079	852.71	0.34	8.252	781.55	0.33
5.360	969.83	0.37	6.143	915.34	0.36	7.100	853.00	0.34	8.272	782.02	0.33
5.381	969.95	0.37	6.164	915.54	0.36	7.120	853.31	0.34	8.292	782.48	0.33
5.402	970.12	0.37	6.184	915.69	0.36	7.141	853.62	0.34	8.312	782.92	0.33
5.422	970.25	0.37	6.205	915.91	0.36	7.161	853.91	0.34	8.333	783.41	0.33
5.443	970.37	0.37	6.225	916.13	0.36	7.182	854.20	0.34	8.353	783.94	0.33
5.464	970.50	0.37	6.246	916.29	0.36	7.202	854.49	0.34	8.373	784.40	0.33
5.485	970.64	0.37	6.267	916.48	0.36	7.223	854.78	0.34	8.393	784.83	0.33
5.506	970.81	0.37	6.287	916.70	0.36	7.243	855.05	0.34	8.414	785.31	0.33
5.527	970.92	0.37	6.308	916.88	0.36	7.264	855.33	0.34	8.434	785.79	0.33
5.548	971.05	0.37	6.328	917.04	0.36	7.284	855.62	0.34	8.454	786.25	0.33
5.569	971.22	0.37	6.349	917.26	0.36	7.305	855.92	0.34	8.474	786.71	0.33
5.590	971.33	0.37	6.369	917.41	0.36	7.325	856.21	0.34	8.495	787.15	0.33
5.611	971.46	0.37	6.390	917.62	0.36	7.346	856.48	0.34	8.515	787.58	0.33
5.632	971.62	0.37	6.410	917.77	0.36	7.366	856.73	0.34	8.535	788.05	0.33
5.653	971.78	0.37	6.431	917.96	0.36	7.387	857.01	0.34	8.555	788.50	0.33
5.674	971.90	0.37	6.452	918.17	0.36	7.407	857.30	0.34	8.576	788.96	0.33
5.695	972.01	0.37	6.472	918.36	0.36	7.428	857.59	0.34	8.596	789.41	0.33
5.716	972.17	0.37	6.493	918.51	0.36	7.448	857.87	0.34	8.616	789.84	0.33
5.737	972.30	0.37	6.513	918.72	0.36	7.469	858.16	0.34	8.636	790.26	0.33
5.758	972.40	0.37	6.534	918.93	0.36	7.489	858.40	0.34	8.657	790.67	0.33

5.779	972.55	0.37	6.554	919.08	0.36	7.510	858.65	0.34	8.677	791.07	0.33
5.799	972.69	0.37	6.575	919.26	0.36	7.530	858.92	0.34	8.697	791.47	0.33
5.820	972.76	0.37	6.595	919.44	0.36	7.551	859.20	0.34	8.717	791.92	0.33
5.841	972.92	0.37	6.616	919.59	0.36	7.571	859.49	0.34	8.738	792.35	0.33
5.862	973.08	0.37	6.637	919.80	0.36	7.592	859.78	0.35	8.758	792.77	0.33
5.883	973.23	0.37	6.657	920.01	0.36	7.612	860.06	0.35	8.778	793.19	0.33
5.904	973.32	0.37	6.678	920.16	0.36	7.633	860.33	0.35	8.798	793.67	0.33
5.925	973.46	0.37	6.698	920.36	0.36	7.653	860.60	0.35	8.819	794.05	0.33
5.946	973.62	0.37	6.719	920.53	0.36	7.674	860.87	0.35	8.839	794.48	0.33
5.967	973.72	0.37	6.739	920.69	0.36	7.694	861.15	0.35	8.859	794.88	0.33
5.988	973.85	0.37	6.760	920.89	0.36	7.715	861.36	0.35	8.879	795.25	0.33
6.009	974.00	0.37	6.781	921.09	0.36	7.735	861.64	0.35	8.900	795.67	0.33
6.030	974.12	0.37	6.801	921.23	0.36	7.756	861.92	0.35	8.920	796.07	0.33
6.051	974.23	0.37	6.822	921.42	0.36	7.776	862.17	0.35	8.940	796.46	0.33
6.072	974.38	0.37	6.842	921.63	0.36	7.797	862.42	0.35	8.960	796.87	0.33
6.093	974.53	0.37	6.863	921.81	0.36	7.817	862.71	0.35	8.981	797.29	0.33
6.114	974.67	0.37	6.883	921.95	0.36	7.838	862.99	0.35	9.001	797.66	0.33
6.135	974.76	0.37	6.904	922.16	0.36	7.858	863.21	0.35	9.021	798.10	0.33
6.156	974.91	0.37	6.924	922.30	0.36	7.879	863.51	0.35	9.041	798.51	0.33
6.176	975.06	0.37	6.945	922.48	0.36	7.899	863.72	0.35	9.062	798.92	0.33
6.197	975.20	0.37	6.966	922.68	0.36	7.920	863.98	0.35	9.082	799.31	0.33
6.218	975.29	0.37	6.986	922.86	0.36	7.940	864.26	0.35	9.102	799.71	0.33
6.239	975.43	0.37	7.007	922.99	0.36	7.961	864.54	0.35	9.123	800.13	0.33
6.260	975.59	0.37	7.027	923.18	0.36	7.981	864.81	0.35	9.143	800.51	0.33
6.281	975.69	0.37	7.048	923.38	0.36	8.002	865.02	0.35	9.163	800.89	0.33
6.302	975.81	0.37	7.068	923.55	0.36	8.022	865.32	0.35	9.183	801.26	0.33
6.323	975.96	0.37	7.089	923.69	0.36	8.043	865.54	0.35	9.204	801.59	0.33
6.344	976.08	0.37	7.109	923.87	0.36	8.063	865.80	0.35	9.224	801.97	0.33
6.365	976.18	0.37	7.130	924.06	0.36	8.084	866.04	0.35	9.244	802.36	0.33
6.386	976.33	0.37	7.151	924.21	0.36	8.104	866.29	0.35	9.264	802.73	0.33
6.407	976.47	0.37	7.171	924.35	0.36	8.125	866.55	0.35	9.285	803.10	0.33
6.428	976.54	0.37	7.192	924.54	0.36	8.146	866.76	0.35	9.305	803.46	0.33
6.449	976.69	0.37	7.212	924.73	0.36	8.166	867.03	0.35	9.325	803.78	0.33
6.470	976.83	0.37	7.233	924.88	0.36	8.187	867.31	0.35	9.345	804.12	0.33
6.491	976.93	0.37	7.253	925.02	0.36	8.207	867.54	0.35	9.366	804.49	0.33
6.512	977.05	0.37	7.274	925.20	0.36	8.228	867.78	0.35	9.386	804.86	0.33
6.533	977.19	0.37	7.294	925.39	0.36	8.248	868.04	0.35	9.406	805.23	0.33
6.553	977.33	0.37	7.315	925.57	0.36	8.269	868.26	0.35	9.426	805.60	0.33
6.574	977.46	0.37	7.336	925.74	0.36	8.289	868.54	0.35	9.447	805.98	0.33
6.595	977.54	0.37	7.356	925.89	0.36	8.310	868.75	0.35	9.467	806.40	0.33
6.616	977.69	0.37	7.377	926.09	0.36	8.330	869.02	0.35	9.487	806.76	0.33
6.637	977.85	0.37	7.397	926.24	0.36	8.351	869.31	0.35	9.507	807.16	0.33
6.658	977.93	0.37	7.418	926.41	0.36	8.371	869.56	0.35	9.528	807.59	0.33
6.679	978.06	0.37	7.438	926.58	0.36	8.392	869.80	0.35	9.548	807.96	0.33
6.700	978.22	0.37	7.459	926.71	0.36	8.412	870.07	0.35	9.568	808.32	0.33

6.721	978.38	0.37	7.480	926.91	0.36	8.433	870.28	0.35	9.588	808.68	0.33
6.742	978.48	0.37	7.500	927.05	0.36	8.453	870.55	0.35	9.609	809.05	0.33
6.763	978.56	0.37	7.521	927.22	0.36	8.474	870.76	0.35	9.629	809.39	0.33
6.784	978.68	0.37	7.541	927.39	0.36	8.494	871.01	0.35	9.649	809.78	0.33
6.805	978.84	0.37	7.562	927.53	0.36	8.515	871.25	0.35	9.669	810.12	0.33
6.826	979.00	0.37	7.582	927.72	0.36	8.535	871.48	0.35	9.690	810.45	0.33
6.847	979.12	0.37	7.603	927.91	0.36	8.556	871.75	0.35	9.710	810.83	0.33
6.868	979.22	0.37	7.623	928.07	0.36	8.576	871.95	0.35	9.730	811.15	0.33
6.889	979.36	0.37	7.644	928.21	0.36	8.597	872.21	0.35	9.750	811.49	0.33
6.910	979.45	0.37	7.665	928.34	0.36	8.617	872.44	0.35	9.771	811.85	0.33
6.930	979.59	0.37	7.685	928.55	0.36	8.638	872.64	0.35	9.791	812.22	0.33
6.951	979.68	0.37	7.706	928.69	0.36	8.658	872.85	0.35	9.811	812.52	0.33
6.972	979.83	0.37	7.726	928.86	0.36	8.679	873.12	0.35	9.831	812.86	0.33
6.993	979.99	0.38	7.747	929.04	0.36	8.699	873.34	0.35	9.852	813.22	0.33
7.014	980.07	0.38	7.767	929.19	0.36	8.720	873.55	0.35	9.872	813.57	0.33
7.035	980.22	0.38	7.788	929.32	0.36	8.740	873.80	0.35	9.892	813.88	0.33
7.056	980.37	0.38	7.808	929.53	0.36	8.761	874.05	0.35	9.913	814.21	0.33
7.077	980.47	0.38	7.829	929.69	0.36	8.781	874.27	0.35	9.933	814.55	0.33
7.098	980.61	0.38	7.850	929.83	0.36	8.802	874.54	0.35	9.953	814.89	0.33
7.119	980.70	0.38	7.870	930.00	0.36	8.822	874.74	0.35	9.973	815.22	0.33
7.140	980.82	0.38	7.891	930.19	0.36	8.843	874.95	0.35	9.994	815.55	0.33
7.161	980.97	0.38	7.911	930.33	0.36	8.863	875.20	0.35	10.014	815.87	0.33
7.182	981.12	0.38	7.932	930.47	0.36	8.884	875.44	0.35	10.034	816.18	0.33
7.203	981.24	0.38	7.952	930.62	0.36	8.904	875.65	0.35	10.054	816.50	0.33
7.224	981.31	0.38	7.973	930.83	0.36	8.925	875.85	0.35	10.075	816.80	0.33
7.245	981.46	0.38	7.993	930.99	0.36	8.945	876.07	0.35	10.095	817.11	0.33
7.266	981.54	0.38	8.014	931.14	0.36	8.966	876.34	0.35	10.115	817.41	0.33
7.287	981.67	0.38	8.035	931.28	0.36	8.986	876.58	0.35	10.135	817.75	0.33
7.307	981.81	0.38	8.055	931.43	0.36	9.007	876.78	0.35	10.156	818.09	0.33
7.328	981.96	0.38	8.076	931.60	0.36	9.027	877.01	0.35	10.176	818.39	0.33
7.349	982.07	0.38	8.096	931.81	0.36	9.048	877.26	0.35	10.196	818.68	0.33
7.370	982.16	0.38	8.117	931.96	0.36	9.068	877.46	0.35	10.216	818.97	0.34
7.391	982.30	0.38	8.137	932.12	0.36	9.089	877.67	0.35	10.237	819.29	0.34
7.412	982.45	0.38	8.158	932.26	0.36	9.109	877.87	0.35	10.257	819.61	0.34
7.433	982.52	0.38	8.179	932.40	0.36	9.130	878.11	0.35	10.277	819.89	0.34
7.454	982.64	0.38	8.199	932.55	0.36	9.150	878.36	0.35	10.297	820.17	0.34
7.475	982.78	0.38	8.220	932.73	0.36	9.171	878.57	0.35	10.318	820.50	0.34
7.496	982.92	0.38	8.240	932.92	0.36	9.192	878.78	0.35	10.338	820.80	0.34
7.517	983.04	0.38	8.261	933.06	0.36	9.212	878.99	0.35	10.358	821.07	0.34
7.538	983.11	0.38	8.281	933.20	0.36	9.233	879.19	0.35	10.378	821.34	0.34
7.559	983.24	0.38	8.302	933.42	0.36	9.253	879.43	0.35	10.399	821.62	0.34
7.580	983.38	0.38	8.322	933.56	0.36	9.274	879.68	0.35	10.419	821.90	0.34
7.601	983.48	0.38	8.343	933.70	0.36	9.294	879.88	0.35	10.439	822.18	0.34
7.622	983.57	0.38	8.364	933.84	0.36	9.315	880.08	0.35	10.459	822.47	0.34
7.643	983.71	0.38	8.384	933.99	0.36	9.335	880.28	0.35	10.480	822.78	0.34

7.664	983.85	0.38	8.405	934.15	0.36	9.356	880.49	0.35	10.500	823.09	0.34
7.684	983.92	0.38	8.425	934.35	0.36	9.376	880.71	0.35	10.520	823.39	0.34
7.705	984.05	0.38	8.446	934.48	0.36	9.397	880.92	0.35	10.540	823.72	0.34
7.726	984.18	0.38	8.466	934.62	0.36	9.417	881.15	0.35	10.561	824.05	0.34
7.747	984.32	0.38	8.487	934.80	0.36	9.438	881.42	0.35	10.581	824.34	0.34
7.768	984.45	0.38	8.507	934.97	0.36	9.458	881.58	0.35	10.601	824.63	0.34
7.789	984.59	0.38	8.528	935.11	0.36	9.479	881.85	0.35	10.621	824.98	0.34
7.810	984.65	0.38	8.549	935.24	0.36	9.499	882.02	0.35	10.642	825.26	0.34
7.831	984.76	0.38	8.569	935.37	0.36	9.520	882.25	0.35	10.662	825.59	0.34
7.852	984.90	0.38	8.590	935.52	0.36	9.540	882.50	0.35	10.682	825.93	0.34
7.873	985.02	0.38	8.610	935.70	0.36	9.561	882.71	0.35	10.702	826.24	0.34
7.894	985.09	0.38	8.631	935.88	0.36	9.581	882.91	0.35	10.723	826.53	0.34
7.915	985.24	0.38	8.651	936.01	0.36	9.602	883.11	0.35	10.743	826.85	0.34
7.936	985.32	0.38	8.672	936.14	0.36	9.622	883.31	0.35	10.763	827.12	0.34
7.957	985.46	0.38	8.693	936.34	0.36	9.643	883.51	0.35	10.784	827.41	0.34
7.978	985.55	0.38	8.713	936.49	0.36	9.663	883.73	0.35	10.804	827.73	0.34
7.999	985.67	0.38	8.734	936.63	0.36	9.684	883.97	0.35	10.824	828.05	0.34
8.020	985.82	0.38	8.754	936.77	0.36	9.704	884.16	0.35	10.844	828.31	0.34
8.041	985.93	0.38	8.775	936.92	0.36	9.725	884.35	0.35	10.865	828.61	0.34
8.062	986.01	0.38	8.795	937.12	0.36	9.745	884.54	0.35	10.885	828.91	0.34
8.082	986.14	0.38	8.816	937.25	0.36	9.766	884.74	0.35	10.905	829.20	0.34
8.103	986.28	0.38	8.836	937.38	0.36	9.786	884.95	0.35	10.925	829.49	0.34
8.124	986.42	0.38	8.857	937.54	0.36	9.807	885.17	0.35	10.946	829.77	0.34
8.145	986.52	0.38	8.878	937.73	0.36	9.827	885.38	0.35	10.966	830.05	0.34
8.166	986.60	0.38	8.898	937.86	0.36	9.848	885.55	0.35	10.986	830.32	0.34
8.187	986.72	0.38	8.919	937.99	0.36	9.868	885.73	0.35	11.006	830.60	0.34
8.208	986.86	0.38	8.939	938.12	0.36	9.889	885.94	0.35	11.027	830.88	0.34
8.229	986.99	0.38	8.960	938.27	0.36	9.909	886.15	0.35	11.047	831.16	0.34
8.250	987.09	0.38	8.980	938.46	0.36	9.930	886.35	0.35	11.067	831.44	0.34
8.271	987.17	0.38	9.001	938.58	0.36	9.950	886.56	0.35	11.087	831.72	0.34
8.292	987.31	0.38	9.021	938.72	0.36	9.971	886.76	0.35	11.108	831.99	0.34
8.313	987.43	0.38	9.042	938.89	0.36	9.991	886.97	0.35	11.128	832.26	0.34
8.334	987.51	0.38	9.063	939.06	0.36	10.012	887.17	0.35	11.148	832.53	0.34
8.355	987.61	0.38	9.083	939.20	0.36	10.032	887.36	0.35	11.168	832.80	0.34
8.376	987.74	0.38	9.104	939.33	0.36	10.053	887.56	0.35	11.189	833.07	0.34
8.397	987.87	0.38	9.124	939.46	0.36	10.073	887.82	0.35	11.209	833.33	0.34
8.418	987.94	0.38	9.145	939.59	0.36	10.094	887.99	0.35	11.229	833.60	0.34
8.439	988.07	0.38	9.165	939.75	0.36	10.114	888.15	0.35	11.249	833.85	0.34
8.459	988.21	0.38	9.186	939.93	0.36	10.135	888.36	0.35	11.270	834.11	0.34
8.480	988.32	0.38	9.206	940.07	0.36	10.155	888.56	0.35	11.290	834.42	0.34
8.501	988.40	0.38	9.227	940.19	0.36	10.176	888.77	0.35	11.310	834.70	0.34
8.522	988.53	0.38	9.248	940.32	0.37	10.196	888.97	0.35	11.330	834.95	0.34
8.543	988.67	0.38	9.268	940.48	0.37	10.217	889.17	0.35	11.351	835.19	0.34
8.564	988.75	0.38	9.289	940.67	0.37	10.237	889.37	0.35	11.371	835.44	0.34
8.585	988.86	0.38	9.309	940.80	0.37	10.258	889.56	0.35	11.391	835.73	0.34

8.606	988.97	0.38	9.330	940.92	0.37	10.279	889.75	0.35	11.411	835.99	0.34
8.627	989.05	0.38	9.350	941.05	0.37	10.299	889.95	0.35	11.432	836.22	0.34
8.648	989.18	0.38	9.371	941.20	0.37	10.320	890.15	0.35	11.452	836.47	0.34
8.669	989.32	0.38	9.392	941.40	0.37	10.340	890.33	0.35	11.472	836.74	0.34
8.690	989.41	0.38	9.412	941.52	0.37	10.361	890.48	0.35	11.492	837.00	0.34
8.711	989.52	0.38	9.433	941.65	0.37	10.381	890.69	0.35	11.513	837.26	0.34
8.732	989.66	0.38	9.453	941.79	0.37	10.402	890.90	0.35	11.533	837.51	0.34
8.753	989.78	0.38	9.474	941.98	0.37	10.422	891.11	0.35	11.553	837.76	0.34
8.774	989.86	0.38	9.494	942.10	0.37	10.443	891.28	0.35	11.573	838.00	0.34
8.795	989.99	0.38	9.515	942.23	0.37	10.463	891.45	0.35	11.594	838.23	0.34
8.816	990.13	0.38	9.535	942.36	0.37	10.484	891.65	0.35	11.614	838.51	0.34
8.836	990.22	0.38	9.556	942.50	0.37	10.504	891.85	0.35	11.634	838.79	0.34
8.857	990.32	0.38	9.577	942.68	0.37	10.525	892.06	0.35	11.655	839.03	0.34
8.878	990.44	0.38	9.597	942.81	0.37	10.545	892.26	0.35	11.675	839.27	0.34
8.899	990.51	0.38	9.618	942.93	0.37	10.566	892.46	0.35	11.695	839.50	0.34
8.920	990.64	0.38	9.638	943.07	0.37	10.586	892.64	0.35	11.715	839.73	0.34
8.941	990.77	0.38	9.659	943.26	0.37	10.607	892.82	0.35	11.736	839.97	0.34
8.962	990.90	0.38	9.679	943.38	0.37	10.627	893.04	0.35	11.756	840.23	0.34
8.983	991.01	0.38	9.700	943.50	0.37	10.648	893.24	0.35	11.776	840.47	0.34
9.004	991.07	0.38	9.720	943.63	0.37	10.668	893.39	0.35	11.796	840.68	0.34
9.025	991.20	0.38	9.741	943.82	0.37	10.689	893.60	0.35	11.817	840.89	0.34
9.046	991.29	0.38	9.762	943.96	0.37	10.709	893.76	0.35	11.837	841.04	0.34
9.067	991.39	0.38	9.782	944.09	0.37	10.730	893.95	0.35	11.857	841.19	0.34
9.088	991.53	0.38	9.803	944.22	0.37	10.750	894.17	0.35	11.877	841.43	0.34
9.109	991.66	0.38	9.823	944.35	0.37	10.771	894.35	0.35	11.898	841.72	0.34
9.130	991.73	0.38	9.844	944.49	0.37	10.791	894.51	0.35	11.918	842.00	0.34
9.151	991.87	0.38	9.864	944.62	0.37	10.812	894.72	0.35	11.938	842.26	0.34
9.172	991.95	0.38	9.885	944.79	0.37	10.832	894.93	0.35	11.958	842.48	0.34
9.193	992.05	0.38	9.905	944.97	0.37	10.853	895.13	0.35	11.979	842.75	0.34
9.213	992.18	0.38	9.926	945.10	0.37	10.873	895.29	0.35	11.999	842.98	0.34
9.234	992.31	0.38	9.947	945.23	0.37	10.894	895.45	0.35	12.019	843.24	0.34
9.255	992.37	0.38	9.967	945.36	0.37	10.914	895.65	0.35	12.039	843.48	0.34
9.276	992.49	0.38	9.988	945.48	0.37	10.935	895.85	0.35	12.060	843.73	0.34
9.297	992.62	0.38	10.008	945.61	0.37	10.955	895.98	0.35	12.080	843.99	0.34
9.318	992.74	0.38	10.029	945.74	0.37	10.976	896.16	0.35	12.100	844.20	0.34
9.339	992.81	0.38	10.049	945.87	0.37	10.996	896.37	0.35	12.120	844.47	0.34
9.360	992.93	0.38	10.070	946.00	0.37	11.017	896.55	0.35	12.141	844.74	0.34
9.381	993.07	0.38	10.091	946.17	0.37	11.037	896.72	0.35	12.161	844.96	0.34
9.402	993.17	0.38	10.111	946.35	0.37	11.058	896.94	0.35	12.181	845.19	0.34
9.423	993.26	0.38	10.132	946.48	0.37	11.078	897.09	0.35	12.201	845.44	0.34
9.444	993.39	0.38	10.152	946.61	0.37	11.099	897.31	0.35	12.222	845.68	0.34
9.465	993.47	0.38	10.173	946.73	0.37	11.119	897.46	0.35	12.242	845.96	0.34
9.486	993.61	0.38	10.193	946.86	0.37	11.140	897.66	0.35	12.262	846.22	0.34
9.507	993.68	0.38	10.214	946.98	0.37	11.160	897.83	0.35	12.282	846.44	0.34
9.528	993.81	0.38	10.234	947.11	0.37	11.181	898.00	0.35	12.303	846.68	0.34

9.549	993.90	0.38	10.255	947.24	0.37	11.201	898.21	0.35	12.323	846.92	0.34
9.570	993.99	0.38	10.276	947.38	0.37	11.222	898.42	0.35	12.343	847.15	0.34
9.590	994.13	0.38	10.296	947.51	0.37	11.242	898.55	0.35	12.363	847.38	0.34
9.611	994.26	0.38	10.317	947.66	0.37	11.263	898.75	0.35	12.384	847.64	0.34
9.632	994.33	0.38	10.337	947.85	0.37	11.283	898.94	0.35	12.404	847.88	0.34
9.653	994.43	0.38	10.358	947.99	0.37	11.304	899.11	0.35	12.424	848.09	0.34
9.674	994.54	0.38	10.378	948.12	0.37	11.324	899.26	0.35	12.445	848.29	0.34
9.695	994.62	0.38	10.399	948.25	0.37	11.345	899.46	0.35	12.465	848.49	0.34
9.716	994.75	0.38	10.419	948.38	0.37	11.366	899.65	0.36	12.485	848.67	0.34
9.737	994.87	0.38	10.440	948.51	0.37	11.386	899.85	0.36	12.505	848.84	0.34
9.758	994.96	0.38	10.461	948.64	0.37	11.407	900.01	0.36	12.526	849.08	0.34
9.779	995.03	0.38	10.481	948.76	0.37	11.427	900.15	0.36	12.546	849.36	0.34
9.800	995.17	0.38	10.502	948.88	0.37	11.448	900.35	0.36	12.566	849.59	0.34
9.821	995.30	0.38	10.522	949.00	0.37	11.468	900.54	0.36	12.586	849.83	0.34
9.842	995.39	0.38	10.543	949.17	0.37	11.489	900.69	0.36	12.607	850.08	0.34
9.863	995.50	0.38	10.563	949.33	0.37	11.509	900.85	0.36	12.627	850.31	0.34
9.884	995.61	0.38	10.584	949.45	0.37	11.530	901.05	0.36	12.647	850.55	0.34
9.905	995.68	0.38	10.604	949.58	0.37	11.550	901.24	0.36	12.667	850.76	0.34
9.926	995.76	0.38	10.625	949.70	0.37	11.571	901.37	0.36	12.688	850.96	0.34
9.947	995.91	0.38	10.646	949.83	0.37	11.591	901.53	0.36	12.708	851.20	0.34
9.967	996.05	0.38	10.666	949.96	0.37	11.612	901.73	0.36	12.728	851.43	0.34
9.988	996.13	0.38	10.687	950.08	0.37	11.632	901.94	0.36	12.748	851.66	0.34
10.009	996.24	0.38	10.707	950.25	0.37	11.653	902.08	0.36	12.769	851.88	0.34
10.030	996.35	0.38	10.728	950.41	0.37	11.673	902.29	0.36	12.789	852.11	0.34
10.051	996.42	0.38	10.748	950.53	0.37	11.694	902.44	0.36	12.809	852.41	0.34
10.072	996.55	0.38	10.769	950.65	0.37	11.714	902.62	0.36	12.829	852.64	0.34
10.093	996.62	0.38	10.790	950.77	0.37	11.735	902.80	0.36	12.850	852.86	0.34
10.114	996.73	0.38	10.810	950.89	0.37	11.755	902.95	0.36	12.870	853.07	0.34
10.135	996.86	0.38	10.831	951.01	0.37	11.776	903.15	0.36	12.890	853.32	0.34
10.156	996.99	0.38	10.851	951.19	0.37	11.796	903.28	0.36	12.910	853.57	0.34
10.177	997.05	0.38	10.872	951.33	0.37	11.817	903.47	0.36	12.931	853.77	0.34
10.198	997.17	0.38	10.892	951.45	0.37	11.837	903.63	0.36	12.951	853.97	0.34
10.219	997.27	0.38	10.913	951.57	0.37	11.858	903.79	0.36	12.971	854.22	0.34
10.240	997.37	0.38	10.933	951.69	0.37	11.878	903.99	0.36	12.991	854.45	0.34
10.261	997.49	0.38	10.954	951.81	0.37	11.899	904.12	0.36	13.012	854.67	0.34
10.282	997.58	0.38	10.975	951.94	0.37	11.919	904.31	0.36	13.032	854.92	0.34
10.303	997.72	0.38	10.995	952.08	0.37	11.940	904.51	0.36	13.052	855.11	0.34
10.324	997.79	0.38	11.016	952.21	0.37	11.960	904.68	0.36	13.072	855.35	0.34
10.344	997.91	0.38	11.036	952.35	0.37	11.981	904.82	0.36	13.093	855.61	0.34
10.365	998.01	0.38	11.057	952.48	0.37	12.001	905.00	0.36	13.113	855.84	0.34
10.386	998.10	0.38	11.077	952.61	0.37	12.022	905.14	0.36	13.133	856.03	0.34
10.407	998.22	0.38	11.098	952.75	0.37	12.042	905.30	0.36	13.153	856.22	0.34
10.428	998.29	0.38	11.118	952.88	0.37	12.063	905.49	0.36	13.174	856.44	0.34
10.449	998.43	0.38	11.139	953.02	0.37	12.083	905.68	0.36	13.194	856.69	0.34
10.470	998.51	0.38	11.160	953.15	0.37	12.104	905.80	0.36	13.214	856.94	0.34

10.491	998.61	0.38	11.180	953.25	0.37	12.124	905.96	0.36	13.234	857.13	0.34
10.512	998.73	0.38	11.201	953.35	0.37	12.145	906.15	0.36	13.255	857.36	0.34
10.533	998.81	0.38	11.221	953.54	0.37	12.165	906.34	0.36	13.275	857.59	0.34
10.554	998.94	0.38	11.242	953.60	0.37	12.186	906.52	0.36	13.295	857.78	0.34
10.575	999.01	0.38	11.262	953.77	0.37	12.206	906.66	0.36	13.316	857.96	0.34
10.596	999.12	0.38	11.283	953.93	0.37	12.227	906.79	0.36	13.336	858.20	0.34
10.617	999.25	0.38	11.304	954.05	0.37	12.247	906.98	0.36	13.356	858.44	0.34
10.638	999.37	0.38	11.324	954.18	0.37	12.268	907.13	0.36	13.376	858.69	0.34
10.659	999.43	0.38	11.345	954.30	0.37	12.288	907.32	0.36	13.397	858.87	0.34
10.680	999.56	0.38	11.365	954.42	0.37	12.309	907.49	0.36	13.417	859.10	0.34
10.701	999.65	0.38	11.386	954.54	0.37	12.329	907.63	0.36	13.437	859.32	0.35
10.721	999.74	0.38	11.406	954.67	0.37	12.350	907.79	0.36	13.457	859.51	0.35
10.742	999.86	0.38	11.427	954.79	0.37	12.370	908.00	0.36	13.478	859.75	0.35
10.763	999.98	0.38	11.447	954.91	0.37	12.391	908.13	0.36	13.498	859.95	0.35
10.784	1000.06	0.38	11.468	955.03	0.37	12.411	908.28	0.36	13.518	860.16	0.35
10.805	1000.14	0.38	11.489	955.14	0.37	12.432	908.48	0.36	13.538	860.39	0.35
10.826	1000.26	0.38	11.509	955.30	0.37	12.453	908.64	0.36	13.559	860.62	0.35
10.847	1000.32	0.38	11.530	955.46	0.37	12.473	908.81	0.36	13.579	860.85	0.35
10.868	1000.44	0.38	11.550	955.58	0.37	12.494	909.00	0.36	13.599	861.07	0.35
10.889	1000.54	0.38	11.571	955.69	0.37	12.514	909.14	0.36	13.619	861.24	0.35
10.910	1000.64	0.38	11.591	955.81	0.37	12.535	909.27	0.36	13.640	861.45	0.35
10.931	1000.77	0.38	11.612	955.93	0.37	12.555	909.45	0.36	13.660	861.67	0.35
10.952	1000.84	0.38	11.632	956.05	0.37	12.576	909.62	0.36	13.680	861.89	0.35
10.973	1000.98	0.38	11.653	956.17	0.37	12.596	909.77	0.36	13.700	862.11	0.35
10.994	1001.06	0.38	11.674	956.30	0.37	12.617	909.96	0.36	13.721	862.32	0.35
11.015	1001.17	0.38	11.694	956.44	0.37	12.637	910.09	0.36	13.741	862.49	0.35
11.036	1001.27	0.38	11.715	956.58	0.37	12.658	910.27	0.36	13.761	862.70	0.35
11.057	1001.36	0.38	11.735	956.70	0.37	12.678	910.43	0.36	13.781	862.92	0.35
11.078	1001.49	0.38	11.756	956.83	0.37	12.699	910.56	0.36	13.802	863.14	0.35
11.098	1001.56	0.38	11.776	956.95	0.37	12.719	910.69	0.36	13.822	863.31	0.35
11.119	1001.68	0.38	11.797	957.07	0.37	12.740	910.88	0.36	13.842	863.49	0.35
11.140	1001.77	0.38	11.817	957.20	0.37	12.760	911.03	0.36	13.862	863.72	0.35
11.161	1001.86	0.38	11.838	957.32	0.37	12.781	911.19	0.36	13.883	863.93	0.35
11.182	1001.99	0.38	11.859	957.45	0.37	12.801	911.38	0.36	13.903	864.14	0.35
11.203	1002.05	0.38	11.879	957.57	0.37	12.822	911.50	0.36	13.923	864.36	0.35
11.224	1002.17	0.38	11.900	957.70	0.37	12.842	911.69	0.36	13.943	864.58	0.35
11.245	1002.26	0.38	11.920	957.82	0.37	12.863	911.83	0.36	13.964	864.80	0.35
11.266	1002.35	0.38	11.941	957.94	0.37	12.883	911.98	0.36	13.984	865.01	0.35
11.287	1002.47	0.38	11.961	958.06	0.37	12.904	912.17	0.36	14.004	865.19	0.35
11.308	1002.59	0.38	11.982	958.18	0.37	12.924	912.29	0.36	14.024	865.38	0.35
11.329	1002.67	0.38	12.003	958.30	0.37	12.945	912.46	0.36	14.045	865.59	0.35
11.350	1002.75	0.38	12.023	958.42	0.37	12.965	912.62	0.36	14.065	865.81	0.35
11.371	1002.87	0.38	12.044	958.54	0.37	12.986	912.76	0.36	14.085	866.02	0.35
11.392	1002.94	0.38	12.064	958.66	0.37	13.006	912.89	0.36	14.105	866.23	0.35
11.413	1003.05	0.38	12.085	958.78	0.37	13.027	913.09	0.36	14.126	866.44	0.35

11.434	1003.15	0.38	12.105	958.90	0.37	13.047	913.24	0.36	14.146	866.64	0.35
11.455	1003.22	0.38	12.126	959.01	0.37	13.068	913.37	0.36	14.166	866.85	0.35
11.475	1003.36	0.38	12.146	959.13	0.37	13.088	913.52	0.36	14.187	867.05	0.35
11.496	1003.43	0.38	12.167	959.25	0.37	13.109	913.72	0.36	14.207	867.25	0.35
11.517	1003.54	0.38	12.188	959.38	0.37	13.129	913.85	0.36	14.227	867.45	0.35
11.538	1003.65	0.38	12.208	959.50	0.37	13.150	914.04	0.36	14.247	867.64	0.35
11.559	1003.73	0.38	12.229	959.62	0.37	13.170	914.20	0.36	14.268	867.83	0.35
11.580	1003.86	0.38	12.249	959.74	0.37	13.191	914.32	0.36	14.288	868.02	0.35
11.601	1003.93	0.38	12.270	959.86	0.37	13.211	914.45	0.36	14.308	868.20	0.35
11.622	1004.05	0.38	12.290	959.97	0.37	13.232	914.64	0.36	14.328	868.42	0.35
11.643	1004.15	0.38	12.311	960.12	0.37	13.252	914.78	0.36	14.349	868.65	0.35
11.664	1004.24	0.38	12.331	960.28	0.37	13.273	914.93	0.36	14.369	868.83	0.35
11.685	1004.36	0.38	12.352	960.40	0.37	13.293	915.12	0.36	14.389	869.01	0.35
11.706	1004.42	0.38	12.373	960.47	0.37	13.314	915.24	0.36	14.409	869.18	0.35
11.727	1004.55	0.38	12.393	960.55	0.37	13.334	915.37	0.36	14.430	869.37	0.35
11.748	1004.64	0.38	12.414	960.68	0.37	13.355	915.52	0.36	14.450	869.59	0.35
11.769	1004.73	0.38	12.434	960.80	0.37	13.375	915.70	0.36	14.470	869.79	0.35
11.790	1004.85	0.38	12.455	960.93	0.37	13.396	915.82	0.36	14.490	869.96	0.35
11.811	1004.91	0.38	12.475	961.06	0.37	13.416	916.00	0.36	14.511	870.16	0.35
11.832	1005.03	0.38	12.496	961.18	0.37	13.437	916.15	0.36	14.531	870.37	0.35
11.853	1005.12	0.38	12.516	961.30	0.37	13.457	916.29	0.36	14.551	870.56	0.35
11.873	1005.19	0.38	12.537	961.43	0.37	13.478	916.46	0.36	14.571	870.73	0.35
11.894	1005.32	0.38	12.558	961.55	0.37	13.498	916.60	0.36	14.592	870.90	0.35
11.915	1005.39	0.38	12.578	961.67	0.37	13.519	916.74	0.36	14.612	871.07	0.35
11.936	1005.49	0.38	12.599	961.79	0.37	13.540	916.91	0.36	14.632	871.26	0.35
11.957	1005.62	0.38	12.619	961.91	0.37	13.560	917.09	0.36	14.652	871.47	0.35
11.978	1005.74	0.38	12.640	962.03	0.37	13.581	917.23	0.36	14.673	871.68	0.35
11.999	1005.80	0.38	12.660	962.15	0.37	13.601	917.35	0.36	14.693	871.89	0.35
12.020	1005.90	0.38	12.681	962.27	0.37	13.622	917.47	0.36	14.713	872.07	0.35
12.041	1006.00	0.38	12.702	962.39	0.37	13.642	917.60	0.36	14.733	872.24	0.35
12.062	1006.06	0.38	12.722	962.51	0.37	13.663	917.79	0.36	14.754	872.43	0.35
12.083	1006.16	0.38	12.743	962.63	0.37	13.683	917.96	0.36	14.774	872.63	0.35
12.104	1006.28	0.38	12.763	962.74	0.37	13.704	918.10	0.36	14.794	872.80	0.35
12.125	1006.39	0.38	12.784	962.86	0.37	13.724	918.23	0.36	14.814	872.97	0.35
12.146	1006.45	0.38	12.804	962.98	0.37	13.745	918.37	0.36	14.835	873.16	0.35
12.167	1006.54	0.38	12.825	963.10	0.37	13.765	918.50	0.36	14.855	873.35	0.35
12.188	1006.66	0.38	12.845	963.21	0.37	13.786	918.68	0.36	14.875	873.52	0.35
12.209	1006.78	0.38	12.866	963.33	0.37	13.806	918.85	0.36	14.895	873.68	0.35
12.230	1006.86	0.38	12.887	963.44	0.37	13.827	918.98	0.36	14.916	873.86	0.35
12.250	1006.92	0.38	12.907	963.56	0.37	13.847	919.12	0.36	14.936	874.05	0.35
12.271	1007.01	0.38	12.928	963.67	0.37	13.868	919.31	0.36	14.956	874.25	0.35
12.292	1007.15	0.38	12.948	963.78	0.37	13.888	919.46	0.36	14.977	874.44	0.35
12.313	1007.21	0.38	12.969	963.90	0.37	13.909	919.58	0.36	14.997	874.63	0.35
12.334	1007.34	0.38	12.989	964.03	0.37	13.929	919.71	0.36	15.017	874.82	0.35
12.355	1007.43	0.38	13.010	964.15	0.37	13.950	919.84	0.36	15.037	875.01	0.35

12.376	1007.53	0.38	13.030	964.28	0.37	13.970	920.01	0.36	15.058	875.19	0.35
12.397	1007.64	0.38	13.051	964.41	0.37	13.991	920.17	0.36	15.078	875.37	0.35
12.418	1007.71	0.38	13.072	964.50	0.37	14.011	920.30	0.36	15.098	875.54	0.35
12.439	1007.77	0.38	13.092	964.61	0.37	14.032	920.48	0.36	15.118	875.71	0.35
12.460	1007.87	0.38	13.113	964.73	0.37	14.052	920.61	0.36	15.139	875.88	0.35
12.481	1007.98	0.38	13.133	964.85	0.37	14.073	920.73	0.36	15.159	876.04	0.35
12.502	1008.05	0.38	13.154	964.97	0.37	14.093	920.86	0.36	15.179	876.21	0.35
12.523	1008.18	0.38	13.174	965.09	0.37	14.114	921.05	0.36	15.199	876.40	0.35
12.544	1008.26	0.38	13.195	965.21	0.37	14.134	921.18	0.36	15.220	876.59	0.35
12.565	1008.36	0.38	13.216	965.33	0.37	14.155	921.30	0.36	15.240	876.78	0.35
12.586	1008.47	0.38	13.236	965.45	0.37	14.175	921.43	0.36	15.260	876.97	0.35
12.607	1008.53	0.38	13.257	965.58	0.37	14.196	921.60	0.36	15.280	877.15	0.35
12.627	1008.66	0.38	13.277	965.69	0.37	14.216	921.74	0.36	15.301	877.31	0.35
12.648	1008.74	0.38	13.298	965.75	0.37	14.237	921.87	0.36	15.321	877.45	0.35
12.669	1008.84	0.38	13.318	965.94	0.37	14.257	922.05	0.36	15.341	877.61	0.35
12.690	1008.96	0.38	13.339	966.04	0.37	14.278	922.18	0.36	15.361	877.78	0.35
12.711	1009.02	0.38	13.359	966.12	0.37	14.298	922.31	0.36	15.382	877.96	0.35
12.732	1009.09	0.38	13.380	966.24	0.37	14.319	922.45	0.36	15.402	878.13	0.35
12.753	1009.20	0.38	13.401	966.36	0.37	14.339	922.59	0.36	15.422	878.30	0.35
12.774	1009.30	0.38	13.421	966.48	0.37	14.360	922.72	0.36	15.442	878.46	0.35
12.795	1009.37	0.38	13.442	966.60	0.37	14.380	922.87	0.36	15.463	878.63	0.35
12.816	1009.49	0.38	13.462	966.72	0.37	14.401	923.01	0.36	15.483	878.79	0.35
12.837	1009.57	0.38	13.483	966.85	0.37	14.421	923.15	0.36	15.503	878.95	0.35
12.858	1009.66	0.38	13.503	966.97	0.37	14.442	923.29	0.36	15.523	879.12	0.35
12.879	1009.77	0.38	13.524	967.09	0.37	14.462	923.43	0.36	15.544	879.29	0.35
12.900	1009.83	0.38	13.544	967.21	0.37	14.483	923.57	0.36	15.564	879.47	0.35
12.921	1009.95	0.38	13.565	967.28	0.37	14.503	923.77	0.36	15.584	879.65	0.35
12.942	1010.03	0.38	13.586	967.37	0.37	14.524	923.91	0.36	15.604	879.83	0.35
12.963	1010.10	0.38	13.606	967.49	0.37	14.544	924.05	0.36	15.625	880.01	0.35
12.984	1010.23	0.38	13.627	967.61	0.37	14.565	924.18	0.36	15.645	880.19	0.35
13.004	1010.30	0.38	13.647	967.73	0.37	14.585	924.32	0.36	15.665	880.37	0.35
13.025	1010.39	0.38	13.668	967.84	0.37	14.606	924.46	0.36	15.685	880.55	0.35
13.046	1010.50	0.38	13.688	967.97	0.37	14.627	924.59	0.36	15.706	880.74	0.35
13.067	1010.57	0.38	13.709	968.08	0.37	14.647	924.72	0.36	15.726	880.92	0.35
13.088	1010.69	0.38	13.729	968.20	0.37	14.668	924.85	0.36	15.746	881.10	0.35
13.109	1010.78	0.38	13.750	968.32	0.37	14.688	924.98	0.36	15.766	881.25	0.35
13.130	1010.85	0.38	13.771	968.44	0.37	14.709	925.12	0.36	15.787	881.40	0.35
13.151	1010.94	0.38	13.791	968.51	0.37	14.729	925.32	0.36	15.807	881.54	0.35
13.172	1011.07	0.38	13.812	968.60	0.37	14.750	925.46	0.36	15.827	881.69	0.35
13.193	1011.13	0.38	13.832	968.72	0.37	14.770	925.59	0.36	15.848	881.84	0.35
13.214	1011.25	0.38	13.853	968.83	0.37	14.791	925.72	0.36	15.868	881.99	0.35
13.235	1011.35	0.38	13.873	968.95	0.37	14.811	925.85	0.36	15.888	882.14	0.35
13.256	1011.41	0.38	13.894	969.05	0.37	14.832	925.98	0.36	15.908	882.29	0.35
13.277	1011.48	0.38	13.915	969.13	0.37	14.852	926.10	0.36	15.929	882.44	0.35
13.298	1011.61	0.38	13.935	969.28	0.37	14.873	926.26	0.36	15.949	882.59	0.35

13.319	1011.69	0.38	13.956	969.42	0.37	14.893	926.43	0.36	15.969	882.69	0.35
13.340	1011.79	0.38	13.976	969.49	0.37	14.914	926.56	0.36	15.989	882.79	0.35
13.361	1011.90	0.38	13.997	969.60	0.37	14.934	926.68	0.36	16.010	882.89	0.35
13.381	1011.97	0.38	14.017	969.74	0.37	14.955	926.80	0.36	16.030	882.98	0.35
13.402	1012.03	0.38	14.038	969.87	0.37	14.975	926.93	0.36	16.050	883.08	0.35
13.423	1012.15	0.38	14.058	970.01	0.37	14.996	927.12	0.36	16.070	883.18	0.35
13.444	1012.25	0.38	14.079	970.07	0.37	15.016	927.25	0.36	16.091	883.32	0.35
13.465	1012.33	0.38	14.100	970.20	0.37	15.037	927.34	0.36	16.111	883.51	0.35
13.486	1012.46	0.38	14.120	970.33	0.37	15.057	927.46	0.36	16.131	883.65	0.35
13.507	1012.52	0.38	14.141	970.44	0.37	15.078	927.66	0.36	16.151	883.85	0.35
13.528	1012.62	0.38	14.161	970.50	0.37	15.098	927.80	0.36	16.172	883.99	0.35
13.549	1012.72	0.38	14.182	970.64	0.37	15.119	927.93	0.36	16.192	884.16	0.35
13.570	1012.78	0.38	14.202	970.76	0.37	15.139	928.07	0.36	16.212	884.36	0.35
13.591	1012.84	0.38	14.223	970.86	0.37	15.160	928.20	0.36	16.232	884.55	0.35
13.612	1012.95	0.38	14.243	970.94	0.37	15.180	928.33	0.36	16.253	884.73	0.35
13.633	1013.05	0.38	14.264	971.07	0.37	15.201	928.46	0.36	16.273	884.86	0.35
13.654	1013.11	0.38	14.285	971.19	0.37	15.221	928.59	0.36	16.293	885.00	0.35
13.675	1013.24	0.38	14.305	971.32	0.37	15.242	928.72	0.36	16.313	885.19	0.35
13.696	1013.32	0.38	14.326	971.42	0.37	15.262	928.84	0.36	16.334	885.34	0.35
13.717	1013.40	0.38	14.346	971.49	0.37	15.283	928.96	0.36	16.354	885.45	0.35
13.738	1013.52	0.38	14.367	971.62	0.37	15.303	929.09	0.36	16.374	885.61	0.35
13.758	1013.58	0.38	14.387	971.74	0.37	15.324	929.23	0.36	16.394	885.84	0.35
13.779	1013.65	0.38	14.408	971.87	0.37	15.344	929.36	0.36	16.415	885.99	0.35
13.800	1013.75	0.38	14.428	971.98	0.37	15.365	929.50	0.36	16.435	886.12	0.35
13.821	1013.87	0.38	14.449	972.05	0.37	15.385	929.64	0.36	16.455	886.29	0.35
13.842	1013.94	0.38	14.470	972.17	0.37	15.406	929.77	0.36	16.475	886.47	0.35
13.863	1014.01	0.38	14.490	972.29	0.37	15.426	929.91	0.36	16.496	886.66	0.35
13.884	1014.08	0.38	14.511	972.42	0.37	15.447	930.04	0.36	16.516	886.82	0.35
13.905	1014.21	0.38	14.531	972.54	0.37	15.467	930.17	0.36	16.536	886.96	0.35
13.926	1014.30	0.38	14.552	972.67	0.37	15.488	930.31	0.36	16.556	887.08	0.35
13.947	1014.37	0.38	14.572	972.75	0.37	15.508	930.45	0.36	16.577	887.23	0.35
13.968	1014.45	0.38	14.593	972.83	0.37	15.529	930.58	0.36	16.597	887.46	0.35
13.989	1014.59	0.38	14.614	972.96	0.37	15.549	930.71	0.36	16.617	887.61	0.35
14.010	1014.65	0.38	14.634	973.08	0.37	15.570	930.84	0.36	16.638	887.76	0.35
14.031	1014.71	0.38	14.655	973.20	0.37	15.590	930.97	0.36	16.658	887.90	0.35
14.052	1014.81	0.38	14.675	973.30	0.37	15.611	931.10	0.36	16.678	888.04	0.35
14.073	1014.93	0.38	14.696	973.36	0.37	15.631	931.23	0.36	16.698	888.20	0.35
14.094	1014.99	0.38	14.716	973.49	0.37	15.652	931.35	0.36	16.719	888.40	0.35
14.115	1015.12	0.38	14.737	973.61	0.37	15.673	931.51	0.36	16.739	888.54	0.35
14.135	1015.20	0.38	14.757	973.73	0.37	15.693	931.67	0.36	16.759	888.75	0.35
14.156	1015.26	0.38	14.778	973.85	0.37	15.714	931.79	0.36	16.779	888.89	0.35
14.177	1015.34	0.38	14.799	973.91	0.37	15.734	931.91	0.36	16.800	889.02	0.35
14.198	1015.48	0.38	14.819	974.00	0.37	15.755	932.03	0.36	16.820	889.17	0.35
14.219	1015.55	0.38	14.840	974.12	0.37	15.775	932.14	0.36	16.840	889.36	0.35
14.240	1015.62	0.38	14.860	974.23	0.37	15.796	932.28	0.36	16.860	889.55	0.35

14.261	1015.69	0.38	14.881	974.35	0.37	15.816	932.42	0.36	16.881	889.69	0.35
14.282	1015.76	0.38	14.901	974.46	0.37	15.837	932.56	0.36	16.901	889.83	0.35
14.303	1015.86	0.38	14.922	974.58	0.37	15.857	932.71	0.36	16.921	890.00	0.35
14.324	1015.99	0.38	14.942	974.69	0.37	15.878	932.82	0.36	16.941	890.13	0.35
14.345	1016.06	0.38	14.963	974.80	0.37	15.898	932.91	0.36	16.962	890.27	0.35
14.366	1016.13	0.38	14.984	974.91	0.37	15.919	933.06	0.36	16.982	890.45	0.35
14.387	1016.26	0.38	15.004	975.03	0.37	15.939	933.20	0.36	17.002	890.65	0.35
14.408	1016.35	0.38	15.025	975.14	0.37	15.960	933.35	0.36	17.022	890.79	0.35
14.429	1016.42	0.38	15.045	975.26	0.37	15.980	933.49	0.36	17.043	890.93	0.35
14.450	1016.49	0.38	15.066	975.37	0.37	16.001	933.63	0.36	17.063	891.07	0.35
14.471	1016.57	0.38	15.086	975.44	0.37	16.021	933.77	0.36	17.083	891.28	0.35
14.492	1016.71	0.38	15.107	975.50	0.37	16.042	933.91	0.36	17.103	891.43	0.35
14.512	1016.77	0.38	15.127	975.61	0.37	16.062	934.02	0.36	17.124	891.57	0.35
14.533	1016.84	0.38	15.148	975.71	0.37	16.083	934.09	0.36	17.144	891.72	0.35
14.554	1016.91	0.38	15.169	975.82	0.37	16.103	934.23	0.36	17.164	891.91	0.35
14.575	1017.01	0.38	15.189	975.93	0.37	16.124	934.41	0.36	17.184	892.05	0.35
14.596	1017.13	0.38	15.210	976.03	0.37	16.144	934.53	0.36	17.205	892.23	0.35
14.617	1017.20	0.38	15.230	976.14	0.37	16.165	934.61	0.36	17.225	892.38	0.35
14.638	1017.27	0.38	15.251	976.24	0.37	16.185	934.74	0.36	17.245	892.51	0.35
14.659	1017.40	0.38	15.271	976.34	0.37	16.206	934.87	0.36	17.265	892.68	0.35
14.680	1017.49	0.38	15.292	976.44	0.37	16.226	935.04	0.36	17.286	892.81	0.35
14.701	1017.56	0.38	15.313	976.55	0.37	16.247	935.18	0.36	17.306	892.94	0.35
14.722	1017.63	0.38	15.333	976.66	0.37	16.267	935.25	0.36	17.326	893.09	0.35
14.743	1017.71	0.38	15.354	976.81	0.37	16.288	935.40	0.36	17.346	893.24	0.35
14.764	1017.85	0.38	15.374	976.93	0.37	16.308	935.54	0.36	17.367	893.46	0.35
14.785	1017.93	0.38	15.395	977.03	0.37	16.329	935.68	0.36	17.387	893.60	0.35
14.806	1018.00	0.38	15.415	977.14	0.37	16.349	935.82	0.36	17.407	893.74	0.35
14.827	1018.07	0.38	15.436	977.24	0.37	16.370	935.92	0.36	17.427	893.87	0.35
14.848	1018.19	0.38	15.456	977.34	0.37	16.390	936.01	0.36	17.448	894.03	0.35
14.869	1018.30	0.38	15.477	977.44	0.37	16.411	936.15	0.36	17.468	894.22	0.35
14.889	1018.37	0.38	15.498	977.53	0.37	16.431	936.29	0.36	17.488	894.35	0.35
14.910	1018.43	0.38	15.518	977.63	0.37	16.452	936.44	0.36	17.509	894.48	0.35
14.931	1018.50	0.38	15.539	977.73	0.37	16.472	936.58	0.36	17.529	894.65	0.35
14.952	1018.63	0.38	15.559	977.82	0.37	16.493	936.72	0.36	17.549	894.82	0.35
14.973	1018.72	0.38	15.580	977.92	0.37	16.513	936.86	0.36	17.569	894.95	0.35
14.994	1018.78	0.38	15.600	978.01	0.37	16.534	936.97	0.36	17.590	895.14	0.35
15.015	1018.86	0.38	15.621	978.10	0.37	16.554	937.05	0.36	17.610	895.28	0.35
15.036	1018.99	0.38	15.641	978.20	0.37	16.575	937.18	0.36	17.630	895.44	0.35
15.057	1019.06	0.38	15.662	978.29	0.37	16.595	937.31	0.36	17.650	895.61	0.35
15.078	1019.12	0.38	15.683	978.40	0.37	16.616	937.44	0.36	17.671	895.72	0.35
15.099	1019.18	0.38	15.703	978.54	0.37	16.636	937.58	0.36	17.691	895.88	0.35
15.120	1019.25	0.38	15.724	978.65	0.37	16.657	937.71	0.36	17.711	896.05	0.35
15.141	1019.37	0.38	15.744	978.74	0.37	16.677	937.83	0.36	17.731	896.21	0.35
15.162	1019.50	0.38	15.765	978.84	0.37	16.698	937.96	0.36	17.752	896.33	0.35
15.183	1019.59	0.38	15.785	978.93	0.37	16.718	938.09	0.36	17.772	896.46	0.35

15.204	1019.65	0.38	15.806	979.02	0.37	16.739	938.22	0.36	17.792	896.61	0.35
15.225	1019.71	0.39	15.827	979.12	0.37	16.760	938.35	0.36	17.812	896.74	0.35
15.246	1019.80	0.39	15.847	979.21	0.37	16.780	938.48	0.36	17.833	896.88	0.35
15.266	1019.91	0.39	15.868	979.30	0.37	16.801	938.61	0.36	17.853	897.08	0.35
15.287	1019.96	0.39	15.888	979.40	0.37	16.821	938.74	0.36	17.873	897.23	0.35
15.308	1020.06	0.39	15.909	979.50	0.37	16.842	938.85	0.36	17.893	897.36	0.35
15.329	1020.15	0.39	15.929	979.60	0.37	16.862	938.91	0.36	17.914	897.49	0.35
15.350	1020.20	0.39	15.950	979.66	0.37	16.883	939.04	0.36	17.934	897.62	0.35
15.371	1020.29	0.39	15.970	979.80	0.37	16.903	939.22	0.36	17.954	897.81	0.35
15.392	1020.38	0.39	15.991	979.88	0.37	16.924	939.35	0.36	17.974	897.96	0.35
15.413	1020.48	0.39	16.012	979.98	0.37	16.944	939.43	0.36	17.995	898.09	0.35
15.434	1020.57	0.39	16.032	980.11	0.37	16.965	939.56	0.36	18.015	898.22	0.35
15.455	1020.66	0.39	16.053	980.24	0.38	16.985	939.69	0.36	18.035	898.40	0.35
15.476	1020.75	0.39	16.073	980.30	0.38	17.006	939.81	0.36	18.055	898.53	0.35
15.497	1020.83	0.39	16.094	980.41	0.38	17.026	939.93	0.37	18.076	898.67	0.35
15.518	1020.91	0.39	16.114	980.53	0.38	17.047	940.05	0.37	18.096	898.84	0.35
15.539	1020.98	0.39	16.135	980.65	0.38	17.067	940.17	0.37	18.116	899.00	0.35
15.560	1021.06	0.39	16.155	980.71	0.38	17.088	940.29	0.37	18.136	899.14	0.35
15.581	1021.16	0.39	16.176	980.82	0.38	17.108	940.41	0.37	18.157	899.24	0.35
15.602	1021.26	0.39	16.197	980.91	0.38	17.129	940.58	0.37	18.177	899.37	0.35
15.623	1021.35	0.39	16.217	980.98	0.38	17.149	940.71	0.37	18.197	899.51	0.35
15.643	1021.44	0.39	16.238	981.10	0.38	17.170	940.82	0.37	18.217	899.65	0.35
15.664	1021.53	0.39	16.258	981.23	0.38	17.190	940.94	0.37	18.238	899.79	0.35
15.685	1021.59	0.39	16.279	981.32	0.38	17.211	941.05	0.37	18.258	899.95	0.35
15.706	1021.65	0.39	16.299	981.39	0.38	17.231	941.16	0.37	18.278	900.16	0.36
15.727	1021.71	0.39	16.320	981.51	0.38	17.252	941.28	0.37	18.298	900.23	0.36
15.748	1021.76	0.39	16.340	981.63	0.38	17.272	941.41	0.37	18.319	900.42	0.36
15.769	1021.84	0.39	16.361	981.75	0.38	17.293	941.54	0.37	18.339	900.59	0.36
15.790	1021.86	0.39	16.382	981.87	0.38	17.313	941.65	0.37	18.359	900.72	0.36
15.811	1021.98	0.39	16.402	981.92	0.38	17.334	941.74	0.37	18.380	900.86	0.36
15.832	1022.08	0.39	16.423	982.01	0.38	17.354	941.88	0.37	18.400	900.99	0.36
15.853	1022.18	0.39	16.443	982.12	0.38	17.375	942.02	0.37	18.420	901.13	0.36
15.874	1022.28	0.39	16.464	982.23	0.38	17.395	942.15	0.37	18.440	901.26	0.36
15.895	1022.38	0.39	16.484	982.31	0.38	17.416	942.29	0.37	18.461	901.44	0.36
15.916	1022.43	0.39	16.505	982.38	0.38	17.436	942.39	0.37	18.481	901.61	0.36
15.937	1022.51	0.39	16.526	982.49	0.38	17.457	942.48	0.37	18.501	901.74	0.36
15.958	1022.59	0.39	16.546	982.61	0.38	17.477	942.61	0.37	18.521	901.86	0.36
15.979	1022.73	0.39	16.567	982.71	0.38	17.498	942.74	0.37	18.542	902.04	0.36
16.000	1022.83	0.39	16.587	982.78	0.38	17.518	942.87	0.37	18.562	902.19	0.36
16.021	1022.94	0.39	16.608	982.86	0.38	17.539	943.00	0.37	18.582	902.30	0.36
16.041	1023.01	0.39	16.628	983.00	0.38	17.559	943.13	0.37	18.602	902.42	0.36
16.062	1023.08	0.39	16.649	983.13	0.38	17.580	943.25	0.37	18.623	902.59	0.36
16.083	1023.14	0.39	16.669	983.21	0.38	17.600	943.38	0.37	18.643	902.74	0.36
16.104	1023.26	0.39	16.690	983.30	0.38	17.621	943.46	0.37	18.663	902.85	0.36
16.125	1023.36	0.39	16.711	983.42	0.38	17.641	943.55	0.37	18.683	903.01	0.36

16.146	1023.42	0.39	16.731	983.49	0.38	17.662	943.68	0.37	18.704	903.15	0.36
16.167	1023.49	0.39	16.752	983.60	0.38	17.682	943.80	0.37	18.724	903.26	0.36
16.188	1023.55	0.39	16.772	983.70	0.38	17.703	943.93	0.37	18.744	903.42	0.36
16.209	1023.62	0.39	16.793	983.77	0.38	17.723	944.05	0.37	18.764	903.57	0.36
16.230	1023.71	0.39	16.813	983.89	0.38	17.744	944.17	0.37	18.785	903.73	0.36
16.251	1023.83	0.39	16.834	983.96	0.38	17.764	944.30	0.37	18.805	903.84	0.36
16.272	1023.90	0.39	16.854	984.07	0.38	17.785	944.42	0.37	18.825	903.95	0.36
16.293	1023.97	0.39	16.875	984.18	0.38	17.805	944.54	0.37	18.845	904.09	0.36
16.314	1024.09	0.39	16.896	984.27	0.38	17.826	944.67	0.37	18.866	904.23	0.36
16.335	1024.18	0.39	16.916	984.40	0.38	17.847	944.78	0.37	18.886	904.37	0.36
16.356	1024.24	0.39	16.937	984.47	0.38	17.867	944.90	0.37	18.906	904.50	0.36
16.377	1024.30	0.39	16.957	984.58	0.38	17.888	945.02	0.37	18.926	904.63	0.36
16.398	1024.36	0.39	16.978	984.68	0.38	17.908	945.14	0.37	18.947	904.76	0.36
16.418	1024.47	0.39	16.998	984.77	0.38	17.929	945.25	0.37	18.967	904.88	0.36
16.439	1024.57	0.39	17.019	984.89	0.38	17.949	945.37	0.37	18.987	905.02	0.36
16.460	1024.64	0.39	17.039	984.96	0.38	17.970	945.49	0.37	19.007	905.21	0.36
16.481	1024.76	0.39	17.060	985.07	0.38	17.990	945.61	0.37	19.028	905.34	0.36
16.502	1024.84	0.39	17.081	985.17	0.38	18.011	945.72	0.37	19.048	905.47	0.36
16.523	1024.90	0.39	17.101	985.25	0.38	18.031	945.84	0.37	19.068	905.59	0.36
16.544	1024.96	0.39	17.122	985.38	0.38	18.052	945.95	0.37	19.088	905.71	0.36
16.565	1025.02	0.39	17.142	985.44	0.38	18.072	946.06	0.37	19.109	905.88	0.36
16.586	1025.13	0.39	17.163	985.55	0.38	18.093	946.17	0.37	19.129	906.02	0.36
16.607	1025.23	0.39	17.183	985.65	0.38	18.113	946.29	0.37	19.149	906.14	0.36
16.628	1025.29	0.39	17.204	985.72	0.38	18.134	946.39	0.37	19.170	906.31	0.36
16.649	1025.36	0.39	17.225	985.79	0.38	18.154	946.54	0.37	19.190	906.44	0.36
16.670	1025.42	0.39	17.245	985.92	0.38	18.175	946.70	0.37	19.210	906.56	0.36
16.691	1025.52	0.39	17.266	986.05	0.38	18.195	946.81	0.37	19.230	906.72	0.36
16.712	1025.63	0.39	17.286	986.15	0.38	18.216	946.92	0.37	19.251	906.84	0.36
16.733	1025.70	0.39	17.307	986.22	0.38	18.236	947.03	0.37	19.271	906.94	0.36
16.754	1025.76	0.39	17.327	986.34	0.38	18.257	947.13	0.37	19.291	907.09	0.36
16.775	1025.88	0.39	17.348	986.42	0.38	18.277	947.24	0.37	19.311	907.22	0.36
16.795	1025.97	0.39	17.368	986.51	0.38	18.298	947.35	0.37	19.332	907.33	0.36
16.816	1026.04	0.39	17.389	986.62	0.38	18.318	947.45	0.37	19.352	907.47	0.36
16.837	1026.10	0.39	17.410	986.68	0.38	18.339	947.56	0.37	19.372	907.60	0.36
16.858	1026.22	0.39	17.430	986.80	0.38	18.359	947.68	0.37	19.392	907.73	0.36
16.879	1026.31	0.39	17.451	986.89	0.38	18.380	947.84	0.37	19.413	907.86	0.36
16.900	1026.37	0.39	17.471	986.96	0.38	18.400	947.97	0.37	19.433	907.99	0.36
16.921	1026.43	0.39	17.492	987.08	0.38	18.421	948.07	0.37	19.453	908.12	0.36
16.942	1026.49	0.39	17.512	987.15	0.38	18.441	948.18	0.37	19.473	908.28	0.36
16.963	1026.59	0.39	17.533	987.26	0.38	18.462	948.28	0.37	19.494	908.45	0.36
16.984	1026.70	0.39	17.553	987.36	0.38	18.482	948.38	0.37	19.514	908.58	0.36
17.005	1026.76	0.39	17.574	987.43	0.38	18.503	948.48	0.37	19.534	908.72	0.36
17.026	1026.82	0.39	17.595	987.56	0.38	18.523	948.60	0.37	19.554	908.85	0.36
17.047	1026.88	0.39	17.615	987.64	0.38	18.544	948.74	0.37	19.575	908.98	0.36
17.068	1026.98	0.39	17.636	987.73	0.38	18.564	948.86	0.37	19.595	909.10	0.36

17.089	1027.10	0.39	17.656	987.85	0.38	18.585	948.96	0.37	19.615	909.22	0.36
17.110	1027.17	0.39	17.677	987.91	0.38	18.605	949.05	0.37	19.635	909.34	0.36
17.131	1027.24	0.39	17.697	988.04	0.38	18.626	949.16	0.37	19.656	909.46	0.36
17.152	1027.30	0.39	17.718	988.12	0.38	18.646	949.30	0.37	19.676	909.58	0.36
17.172	1027.40	0.39	17.739	988.20	0.38	18.667	949.42	0.37	19.696	909.75	0.36
17.193	1027.51	0.39	17.759	988.33	0.38	18.687	949.51	0.37	19.716	909.88	0.36
17.214	1027.57	0.39	17.780	988.45	0.38	18.708	949.64	0.37	19.737	910.00	0.36
17.235	1027.63	0.39	17.800	988.53	0.38	18.728	949.78	0.37	19.757	910.13	0.36
17.256	1027.69	0.39	17.821	988.62	0.38	18.749	949.87	0.37	19.777	910.27	0.36
17.277	1027.77	0.39	17.841	988.74	0.38	18.769	949.96	0.37	19.797	910.40	0.36
17.298	1027.89	0.39	17.862	988.80	0.38	18.790	950.07	0.37	19.818	910.53	0.36
17.319	1027.96	0.39	17.882	988.87	0.38	18.810	950.15	0.37	19.838	910.66	0.36
17.340	1028.02	0.39	17.903	988.98	0.38	18.831	950.24	0.37	19.858	910.78	0.36
17.361	1028.09	0.39	17.924	989.08	0.38	18.851	950.38	0.37	19.878	910.90	0.36
17.382	1028.21	0.39	17.944	989.15	0.38	18.872	950.51	0.37	19.899	911.01	0.36
17.403	1028.28	0.39	17.965	989.28	0.38	18.892	950.64	0.37	19.919	911.12	0.36
17.424	1028.34	0.39	17.985	989.35	0.38	18.913	950.72	0.37	19.939	911.24	0.36
17.445	1028.41	0.39	18.006	989.45	0.38	18.934	950.81	0.37	19.959	911.38	0.36
17.466	1028.54	0.39	18.026	989.56	0.38	18.954	950.95	0.37	19.980	911.53	0.36
17.487	1028.61	0.39	18.047	989.63	0.38	18.975	951.08	0.37	20.000	911.67	0.36
17.508	1028.67	0.39	18.067	989.75	0.38	18.995	951.15	0.37			
17.529	1028.73	0.39	18.088	989.83	0.38	19.016	951.26	0.37			
17.549	1028.79	0.39	18.109	989.92	0.38	19.036	951.39	0.37			
17.570	1028.92	0.39	18.129	990.04	0.38	19.057	951.51	0.37			
17.591	1029.00	0.39	18.150	990.10	0.38	19.077	951.58	0.37			
17.612	1029.06	0.39	18.170	990.21	0.38	19.098	951.71	0.37			
17.633	1029.13	0.39	18.191	990.31	0.38	19.118	951.85	0.37			
17.654	1029.19	0.39	18.211	990.38	0.38	19.139	951.94	0.37			
17.675	1029.29	0.39	18.232	990.45	0.38	19.159	952.02	0.37			
17.696	1029.39	0.39	18.252	990.58	0.38	19.180	952.15	0.37			
17.717	1029.45	0.39	18.273	990.66	0.38	19.200	952.27	0.37			
17.738	1029.51	0.39	18.294	990.77	0.38	19.221	952.39	0.37			
17.759	1029.61	0.39	18.314	990.88	0.38	19.241	952.52	0.37			
17.780	1029.71	0.39	18.335	990.94	0.38	19.262	952.64	0.37			
17.801	1029.77	0.39	18.355	991.07	0.38	19.282	952.77	0.37			
17.822	1029.83	0.39	18.376	991.14	0.38	19.303	952.84	0.37			
17.843	1029.90	0.39	18.396	991.24	0.38	19.323	952.92	0.37			
17.864	1029.99	0.39	18.417	991.35	0.38	19.344	953.05	0.37			
17.885	1030.11	0.39	18.438	991.41	0.38	19.364	953.17	0.37			
17.906	1030.16	0.39	18.458	991.52	0.38	19.385	953.29	0.37			
17.926	1030.22	0.39	18.479	991.61	0.38	19.405	953.39	0.37			
17.947	1030.28	0.39	18.499	991.67	0.38	19.426	953.44	0.37			
17.968	1030.34	0.39	18.520	991.73	0.38	19.446	953.56	0.37			
17.989	1030.48	0.39	18.540	991.84	0.38	19.467	953.71	0.37			
18.010	1030.56	0.39	18.561	991.94	0.38	19.487	953.87	0.37			

18.031	1030.62	0.39	18.581	992.01	0.38	19.508	953.92	0.37			
18.052	1030.68	0.39	18.602	992.15	0.38	19.530	954.02	0.37			
18.073	1030.74	0.39	18.623	992.22	0.38	19.550	954.14	0.37			
18.094	1030.84	0.39	18.643	992.33	0.38	19.570	954.26	0.37			
18.115	1030.94	0.39	18.664	992.43	0.38	19.590	954.39	0.37			
18.136	1031.00	0.39	18.684	992.50	0.38	19.610	954.47	0.37			
18.157	1031.05	0.39	18.705	992.56	0.38	19.630	954.57	0.37			
18.178	1031.14	0.39	18.725	992.67	0.38	19.650	954.69	0.37			
18.199	1031.25	0.39	18.746	992.80	0.38	19.670	954.75	0.37			
18.220	1031.30	0.39	18.766	992.91	0.38	19.690	954.88	0.37			
18.241	1031.37	0.39	18.787	992.97	0.38	19.710	955.00	0.37			
18.262	1031.47	0.39	18.808	993.08	0.38	19.730	955.13	0.37			
18.283	1031.59	0.39	18.828	993.17	0.38	19.750	955.25	0.37			
18.303	1031.66	0.39	18.849	993.24	0.38	19.770	955.31	0.37			
18.324	1031.73	0.39	18.869	993.36	0.38	19.790	955.42	0.37			
18.345	1031.80	0.39	18.890	993.43	0.38	19.820	955.55	0.37			
18.366	1031.87	0.39	18.910	993.52	0.38	19.840	955.68	0.37			
18.387	1031.93	0.39	18.931	993.63	0.38	19.860	955.80	0.37			
18.408	1032.00	0.39	18.951	993.69	0.38	19.880	955.87	0.37			
18.429	1032.06	0.39	18.972	993.76	0.38	19.900	955.96	0.37			
18.450	1032.13	0.39	18.993	993.85	0.38	19.920	956.08	0.37			
18.471	1032.19	0.39	19.013	993.97	0.38	19.940	956.20	0.37			
18.492	1032.31	0.39	19.034	994.03	0.38	19.960	956.32	0.37			
18.513	1032.41	0.39	19.054	994.16	0.38	19.980	956.44	0.37			
18.534	1032.48	0.39	19.075	994.24	0.38	20.000	956.56	0.37			
18.555	1032.55	0.39	19.095	994.32	0.38						
18.576	1032.61	0.39	19.116	994.45	0.38						
18.597	1032.68	0.39	19.137	994.51	0.38						
18.618	1032.78	0.39	19.157	994.57	0.38						
18.639	1032.89	0.39	19.178	994.66	0.38						
18.660	1032.95	0.39	19.198	994.78	0.38						
18.680	1033.01	0.39	19.219	994.84	0.38						
18.701	1033.08	0.39	19.239	994.97	0.38						
18.722	1033.15	0.39	19.260	995.06	0.38						
18.743	1033.22	0.39	19.280	995.12	0.38						
18.764	1033.29	0.39	19.301	995.20	0.38						
18.785	1033.36	0.39	19.322	995.32	0.38						
18.806	1033.49	0.39	19.342	995.40	0.38						
18.827	1033.57	0.39	19.363	995.49	0.38						
18.848	1033.64	0.39	19.383	995.60	0.38						
18.869	1033.72	0.39	19.404	995.66	0.38						
18.890	1033.85	0.39	19.424	995.78	0.38						
18.911	1033.92	0.39	19.445	995.87	0.38						
18.932	1033.98	0.39	19.465	995.93	0.38						
18.953	1034.05	0.39	19.486	995.99	0.38						

18.974	1034.11	0.39	19.507	996.13	0.38						
18.995	1034.18	0.39	19.527	996.21	0.38						
19.016	1034.25	0.39	19.548	996.27	0.38						
19.037	1034.31	0.39	19.568	996.35	0.38						
19.057	1034.41	0.39	19.589	996.47	0.38						
19.078	1034.52	0.39	19.609	996.54	0.38						
19.099	1034.59	0.39	19.630	996.64	0.38						
19.120	1034.65	0.39	19.651	996.75	0.38						
19.141	1034.71	0.39	19.671	996.81	0.38						
19.162	1034.77	0.39	19.692	996.87	0.38						
19.183	1034.84	0.39	19.712	996.97	0.38						
19.204	1034.97	0.39	19.733	997.07	0.38						
19.225	1035.04	0.39	19.753	997.13	0.38						
19.246	1035.10	0.39	19.774	997.25	0.38						
19.267	1035.16	0.39	19.794	997.33	0.38						
19.288	1035.28	0.39	19.815	997.41	0.38						
19.309	1035.36	0.39	19.836	997.53	0.38						
19.330	1035.41	0.39	19.856	997.60	0.38						
19.351	1035.47	0.39	19.877	997.66	0.38						
19.372	1035.53	0.39	19.897	997.75	0.38						
19.393	1035.62	0.39	19.918	997.88	0.38						
19.414	1035.73	0.39	19.938	997.94	0.38						
19.434	1035.79	0.39	19.959	998.01	0.38						
19.455	1035.85	0.39	19.979	998.11	0.38						
19.476	1035.91	0.39	20.000	998.22	0.38						
19.497	1035.97	0.39									
19.518	1036.09	0.39									
19.539	1036.18	0.39									
19.560	1036.24	0.39									
19.581	1036.30	0.39									
19.602	1036.36	0.39									
19.623	1036.46	0.39									
19.644	1036.56	0.39									
19.665	1036.62	0.39									
19.686	1036.68	0.39									
19.707	1036.73	0.39									
19.728	1036.79	0.39									
19.749	1036.92	0.39									
19.770	1036.99	0.39									
19.791	1037.05	0.39									
19.812	1037.10	0.39									
19.832	1037.21	0.39									
19.853	1037.30	0.39									
19.874	1037.36	0.39									
19.895	1037.41	0.39									

19.916	1037.49	0.39									
19.937	1037.60	0.39									
19.958	1037.65	0.39									
19.979	1037.71	0.39									
20.000	1037.77	0.39									

Combined standard uncertainties:

$u(T)$= 0.006 K; $u(p)$= 0.0020 MPa for p < 6 MPa; $u(p)$= 0.024 MPa for 6 MPa ≤ p ≤ 70 MPa

$u(x_{\mathrm{CO_2}})$= 0.00024; $u(x_{\mathrm{O_2}})$= 0.000030; $u(x_{\mathrm{SO_2}})$= 0.0000023; $u(x_{\mathrm{CO}})$= 0.0000043

Table S1 (continued). $p\rho T$ experimental data for the CO_2 + O_2 + SO_2 + CO (Mix 1) and CO_2 + NO + SO_2 + CO (Mix 1) mixtures. $u(\rho)$: combined standard uncertainty.

Mix 2: x_{CO_2} = 0.99592; x_{NO} = 0.001410; x_{SO_2} = 0.0009100; x_{CO} = 0.0017002											
T= 263.17±0.05 K			T= 273.15±0.01 K			T= 283.15±0.01 K			T= 293.14±0.01 K		
p (MPa)	ρ (kg.m^{-3})	$u(\rho)$ (kg.m^{-3})	p (MPa)	ρ (kg.m^{-3})	$u(\rho)$ (kg.m^{-3})	p (MPa)	ρ (kg.m^{-3})	$u(\rho)$ (kg.m^{-3})	p (MPa)	ρ (kg.m^{-3})	$u(\rho)$ (kg.m^{-3})
0.100	1.79	0.21	0.100	1.74	0.21	0.100	1.84	0.22	0.100	1.76	0.22
0.119	2.16	0.21	0.118	2.15	0.22	0.119	2.16	0.22	0.120	2.13	0.22
0.138	2.57	0.22	0.137	2.53	0.22	0.137	2.55	0.22	0.139	2.46	0.22
0.157	2.98	0.22	0.155	2.89	0.22	0.156	2.89	0.22	0.159	2.85	0.22
0.175	3.37	0.22	0.174	3.25	0.22	0.175	3.18	0.22	0.179	3.17	0.22
0.194	3.76	0.22	0.192	3.62	0.22	0.194	3.61	0.22	0.198	3.53	0.22
0.213	4.14	0.22	0.210	3.99	0.22	0.212	3.97	0.22	0.218	3.88	0.22
0.232	4.52	0.22	0.229	4.36	0.22	0.231	4.32	0.22	0.238	4.24	0.22
0.251	4.92	0.22	0.247	4.73	0.22	0.250	4.67	0.22	0.257	4.63	0.22
0.270	5.35	0.22	0.266	5.11	0.22	0.269	5.03	0.22	0.277	5.00	0.22
0.288	5.74	0.22	0.284	5.49	0.22	0.287	5.39	0.22	0.297	5.36	0.22
0.307	6.13	0.22	0.302	5.87	0.22	0.306	5.74	0.22	0.316	5.75	0.22
0.326	6.52	0.22	0.321	6.26	0.22	0.325	6.09	0.22	0.336	6.10	0.22
0.345	6.92	0.22	0.339	6.64	0.22	0.343	6.46	0.22	0.356	6.46	0.22
0.364	7.33	0.22	0.358	6.98	0.22	0.362	6.88	0.22	0.375	6.85	0.22
0.383	7.74	0.22	0.376	7.38	0.22	0.381	7.23	0.22	0.395	7.21	0.22
0.402	8.15	0.22	0.394	7.79	0.22	0.400	7.56	0.22	0.415	7.57	0.22
0.420	8.58	0.22	0.413	8.16	0.22	0.418	7.94	0.22	0.434	7.94	0.22
0.439	9.01	0.22	0.431	8.55	0.22	0.437	8.31	0.22	0.454	8.31	0.22
0.458	9.42	0.22	0.450	8.95	0.22	0.456	8.66	0.22	0.474	8.67	0.22
0.477	9.81	0.22	0.468	9.31	0.22	0.474	9.06	0.22	0.493	9.04	0.22
0.496	10.26	0.22	0.486	9.68	0.22	0.493	9.42	0.22	0.513	9.44	0.22
0.515	10.66	0.22	0.505	10.05	0.22	0.512	9.77	0.22	0.533	9.82	0.22
0.533	11.06	0.22	0.523	10.43	0.22	0.531	10.19	0.22	0.552	10.21	0.22
0.552	11.46	0.22	0.542	10.81	0.22	0.549	10.55	0.22	0.572	10.57	0.22
0.571	11.88	0.22	0.560	11.20	0.22	0.568	10.95	0.22	0.592	10.94	0.22
0.590	12.31	0.22	0.578	11.60	0.22	0.587	11.30	0.22	0.611	11.32	0.22
0.609	12.74	0.22	0.597	12.00	0.22	0.606	11.69	0.22	0.631	11.71	0.22
0.628	13.18	0.22	0.615	12.40	0.22	0.624	12.05	0.22	0.651	12.09	0.22
0.646	13.56	0.22	0.634	12.81	0.22	0.643	12.44	0.22	0.670	12.48	0.22
0.665	14.02	0.22	0.652	13.15	0.22	0.662	12.81	0.22	0.690	12.85	0.22
0.684	14.41	0.22	0.670	13.56	0.22	0.680	13.20	0.22	0.709	13.26	0.22
0.703	14.87	0.22	0.689	13.93	0.22	0.699	13.58	0.22	0.729	13.62	0.22
0.722	15.28	0.22	0.707	14.36	0.22	0.718	13.95	0.22	0.749	14.04	0.22
0.741	15.69	0.22	0.726	14.76	0.22	0.737	14.33	0.22	0.768	14.39	0.22

0.760	16.11	0.22	0.744	15.12	0.22	0.755	14.73	0.22	0.788	14.74	0.22
0.778	16.54	0.22	0.762	15.53	0.22	0.774	15.09	0.22	0.808	15.17	0.22
0.797	16.98	0.22	0.781	15.90	0.22	0.793	15.48	0.22	0.827	15.58	0.22
0.816	17.43	0.22	0.799	16.31	0.22	0.811	15.90	0.22	0.847	15.94	0.22
0.835	17.86	0.22	0.817	16.73	0.22	0.830	16.26	0.22	0.867	16.32	0.22
0.854	18.27	0.22	0.836	17.14	0.22	0.849	16.68	0.22	0.886	16.73	0.22
0.873	18.69	0.22	0.854	17.54	0.22	0.868	17.08	0.22	0.906	17.13	0.22
0.891	19.12	0.22	0.873	17.93	0.22	0.886	17.40	0.22	0.926	17.51	0.22
0.910	19.57	0.22	0.891	18.32	0.22	0.905	17.81	0.22	0.945	17.88	0.22
0.929	20.02	0.22	0.909	18.72	0.22	0.924	18.23	0.22	0.965	18.29	0.22
0.948	20.44	0.22	0.928	19.19	0.22	0.943	18.62	0.22	0.985	18.68	0.22
0.967	20.88	0.22	0.946	19.60	0.22	0.961	18.97	0.22	1.004	19.10	0.22
0.986	21.34	0.22	0.965	20.00	0.22	0.980	19.39	0.22	1.024	19.47	0.22
1.005	21.78	0.22	0.983	20.39	0.22	0.999	19.80	0.22	1.044	19.87	0.22
1.023	22.29	0.22	1.001	20.78	0.22	1.017	20.20	0.22	1.063	20.28	0.22
1.042	22.74	0.22	1.020	21.24	0.22	1.036	20.60	0.22	1.083	20.68	0.22
1.061	23.19	0.22	1.038	21.61	0.22	1.055	21.01	0.22	1.103	21.09	0.22
1.080	23.59	0.22	1.057	22.05	0.22	1.074	21.39	0.22	1.122	21.49	0.22
1.099	24.06	0.22	1.075	22.43	0.22	1.092	21.75	0.22	1.142	21.90	0.22
1.118	24.55	0.22	1.093	22.86	0.22	1.111	22.16	0.22	1.162	22.30	0.22
1.136	24.98	0.22	1.112	23.31	0.22	1.130	22.59	0.22	1.181	22.70	0.22
1.155	25.48	0.22	1.130	23.72	0.22	1.148	23.02	0.22	1.201	23.09	0.22
1.174	25.92	0.22	1.149	24.11	0.22	1.167	23.41	0.22	1.221	23.49	0.22
1.193	26.37	0.22	1.167	24.55	0.22	1.186	23.80	0.22	1.240	23.92	0.22
1.212	26.83	0.22	1.185	24.97	0.22	1.205	24.20	0.22	1.260	24.35	0.22
1.231	27.34	0.22	1.204	25.40	0.22	1.223	24.60	0.22	1.280	24.75	0.22
1.250	27.78	0.22	1.222	25.82	0.22	1.242	25.03	0.22	1.299	25.13	0.22
1.268	28.25	0.22	1.241	26.25	0.22	1.261	25.48	0.22	1.319	25.54	0.22
1.287	28.74	0.22	1.259	26.68	0.22	1.280	25.89	0.22	1.339	25.96	0.22
1.306	29.24	0.22	1.277	27.12	0.22	1.298	26.30	0.22	1.358	26.37	0.22
1.325	29.69	0.22	1.296	27.58	0.22	1.317	26.71	0.22	1.378	26.78	0.22
1.344	30.20	0.22	1.314	28.00	0.22	1.336	27.13	0.22	1.398	27.19	0.22
1.363	30.69	0.22	1.333	28.42	0.22	1.354	27.53	0.22	1.417	27.63	0.22
1.381	31.19	0.23	1.351	28.87	0.22	1.373	27.92	0.22	1.437	28.08	0.22
1.400	31.65	0.23	1.369	29.32	0.22	1.392	28.36	0.22	1.457	28.49	0.22
1.419	32.17	0.23	1.388	29.76	0.22	1.411	28.80	0.22	1.476	28.89	0.22
1.438	32.66	0.23	1.406	30.19	0.22	1.429	29.21	0.22	1.496	29.30	0.22
1.457	33.14	0.23	1.425	30.63	0.22	1.448	29.62	0.22	1.516	29.71	0.22
1.476	33.63	0.23	1.443	31.07	0.22	1.467	30.03	0.22	1.535	30.13	0.22
1.494	34.13	0.23	1.461	31.51	0.22	1.486	30.49	0.22	1.555	30.60	0.22
1.513	34.64	0.23	1.480	31.96	0.22	1.504	30.90	0.22	1.575	31.05	0.22
1.532	35.16	0.23	1.498	32.42	0.22	1.523	31.36	0.22	1.594	31.47	0.22
1.551	35.62	0.23	1.517	32.88	0.22	1.542	31.78	0.22	1.614	31.90	0.22
1.570	36.17	0.23	1.535	33.36	0.22	1.560	32.19	0.22	1.634	32.33	0.22
1.589	36.66	0.23	1.553	33.77	0.22	1.579	32.66	0.22	1.653	32.76	0.22

1.608	37.16	0.23	1.572	34.24	0.22	1.598	33.08	0.22	1.673	33.19	0.22
1.626	37.74	0.23	1.590	34.72	0.22	1.617	33.53	0.22	1.693	33.62	0.22
1.645	38.21	0.23	1.609	35.18	0.22	1.635	33.92	0.22	1.712	34.05	0.22
1.664	38.74	0.23	1.627	35.66	0.23	1.654	34.37	0.22	1.732	34.48	0.22
1.683	39.28	0.23	1.645	36.07	0.22	1.673	34.83	0.22	1.751	34.92	0.22
1.702	39.79	0.23	1.664	36.54	0.22	1.691	35.30	0.22	1.771	35.36	0.22
1.721	40.36	0.23	1.682	37.01	0.22	1.710	35.72	0.22	1.791	35.79	0.22
1.739	40.86	0.23	1.701	37.47	0.22	1.729	36.18	0.22	1.810	36.23	0.22
1.758	41.38	0.23	1.719	37.93	0.22	1.748	36.60	0.22	1.830	36.68	0.22
1.777	41.97	0.23	1.737	38.44	0.23	1.766	37.07	0.22	1.850	37.16	0.22
1.796	42.50	0.23	1.756	38.93	0.23	1.785	37.50	0.22	1.869	37.62	0.22
1.815	43.03	0.23	1.774	39.40	0.23	1.804	37.99	0.22	1.889	38.07	0.22
1.834	43.58	0.23	1.793	39.88	0.23	1.823	38.41	0.22	1.909	38.51	0.22
1.853	44.13	0.23	1.811	40.36	0.23	1.841	38.91	0.22	1.928	38.94	0.22
1.871	44.73	0.23	1.829	40.82	0.23	1.860	39.34	0.22	1.948	39.41	0.22
1.890	45.26	0.23	1.848	41.33	0.23	1.879	39.86	0.22	1.968	39.87	0.22
1.909	45.82	0.23	1.866	41.83	0.23	1.897	40.35	0.23	1.987	40.31	0.22
1.928	46.37	0.23	1.885	42.29	0.23	1.916	40.82	0.23	2.007	40.79	0.22
1.947	47.01	0.23	1.903	42.78	0.23	1.935	41.22	0.22	2.027	41.23	0.22
1.966	47.53	0.23	1.921	43.29	0.23	1.954	41.67	0.22	2.046	41.66	0.22
1.984	48.13	0.23	1.940	43.80	0.23	1.972	42.23	0.23	2.066	42.13	0.22
2.003	48.72	0.23	1.958	44.32	0.23	1.991	42.69	0.23	2.086	42.59	0.22
2.022	49.27	0.23	1.977	44.82	0.23	2.010	43.12	0.23	2.105	43.08	0.22
2.041	49.85	0.23	1.995	45.28	0.23	2.028	43.65	0.23	2.125	43.56	0.22
2.060	50.47	0.23	2.013	45.81	0.23	2.047	44.12	0.23	2.145	44.01	0.22
2.079	51.06	0.23	2.032	46.35	0.23	2.066	44.59	0.23	2.164	44.48	0.22
2.098	51.63	0.23	2.050	46.81	0.23	2.085	45.07	0.23	2.184	44.94	0.22
2.116	52.25	0.23	2.069	47.36	0.23	2.103	45.55	0.23	2.204	45.44	0.22
2.135	52.84	0.23	2.087	47.84	0.23	2.122	46.03	0.23	2.223	45.90	0.22
2.154	53.44	0.23	2.105	48.40	0.23	2.141	46.50	0.23	2.243	46.36	0.22
2.173	54.06	0.23	2.124	48.89	0.23	2.160	46.96	0.23	2.263	46.82	0.22
2.192	54.65	0.23	2.142	49.44	0.23	2.178	47.43	0.23	2.282	47.30	0.22
2.211	55.25	0.23	2.160	49.99	0.23	2.197	47.95	0.23	2.302	47.80	0.22
2.229	55.88	0.23	2.179	50.46	0.23	2.216	48.42	0.23	2.322	48.27	0.22
2.248	56.51	0.23	2.197	51.01	0.23	2.234	48.90	0.23	2.341	48.75	0.22
2.267	57.12	0.23	2.216	51.57	0.23	2.253	49.44	0.23	2.361	49.22	0.22
2.286	57.78	0.23	2.234	52.05	0.23	2.272	49.91	0.23	2.381	49.72	0.22
2.305	58.39	0.23	2.252	52.62	0.23	2.291	50.39	0.23	2.400	50.22	0.22
2.324	59.07	0.23	2.271	53.19	0.23	2.309	50.89	0.23	2.420	50.71	0.22
2.342	59.70	0.23	2.289	53.69	0.23	2.328	51.44	0.23	2.440	51.17	0.22
2.361	60.38	0.23	2.308	54.24	0.23	2.347	51.93	0.23	2.459	51.68	0.22
2.380	60.99	0.23	2.326	54.80	0.23	2.365	52.43	0.23	2.479	52.19	0.22
2.399	61.66	0.23	2.344	55.36	0.23	2.384	52.92	0.23	2.499	52.70	0.22
2.418	62.30	0.23	2.363	55.85	0.23	2.403	53.42	0.23	2.518	53.19	0.22
2.437	62.98	0.23	2.381	56.46	0.23	2.422	53.94	0.23	2.538	53.68	0.22

2.456	63.69	0.23	2.400	57.00	0.23	2.440	54.46	0.23	2.558	54.20	0.22
2.474	64.32	0.23	2.418	57.53	0.23	2.459	54.96	0.23	2.577	54.71	0.22
2.493	65.04	0.23	2.436	58.07	0.23	2.478	55.49	0.23	2.597	55.20	0.22
2.512	65.75	0.23	2.455	58.69	0.23	2.497	56.00	0.23	2.617	55.67	0.22
2.531	66.42	0.23	2.473	59.25	0.23	2.515	56.54	0.23	2.636	56.17	0.22
2.550	67.16	0.23	2.492	59.80	0.23	2.534	57.03	0.23	2.656	56.68	0.22
2.569	67.86	0.23	2.510	60.36	0.23	2.553	57.56	0.23	2.676	57.20	0.22
2.587	68.56	0.23	2.528	61.01	0.23	2.571	58.10	0.23	2.695	57.72	0.22
2.606	69.25	0.23	2.547	61.59	0.23	2.590	58.65	0.23	2.715	58.22	0.22
2.625	70.01	0.23	2.565	62.18	0.23	2.609	59.19	0.23	2.735	58.78	0.22
			2.584	62.76	0.23	2.628	59.70	0.23	2.754	59.30	0.22
			2.602	63.36	0.23	2.646	60.21	0.23	2.774	59.79	0.22
			2.620	63.94	0.23	2.665	60.75	0.23	2.794	60.35	0.22
			2.639	64.52	0.23	2.684	61.31	0.23	2.813	60.86	0.22
			2.657	65.10	0.23	2.703	61.87	0.23	2.833	61.36	0.22
			2.676	65.69	0.23	2.721	62.43	0.23	2.852	61.86	0.22
			2.694	66.33	0.23	2.740	62.93	0.23	2.872	62.44	0.23
			2.712	66.93	0.23	2.759	63.49	0.23	2.892	62.95	0.22
			2.731	67.49	0.23	2.777	64.07	0.23	2.911	63.47	0.22
			2.749	68.14	0.23	2.796	64.65	0.23	2.931	64.05	0.23
			2.768	68.75	0.23	2.815	65.16	0.23	2.951	64.56	0.23
			2.786	69.41	0.23	2.834	65.75	0.23	2.970	65.09	0.23
			2.804	70.02	0.23	2.852	66.34	0.23	2.990	65.66	0.23
			2.823	70.63	0.23	2.871	66.87	0.23	3.010	66.18	0.23
			2.841	71.32	0.23	2.890	67.47	0.23	3.029	66.74	0.23
			2.860	71.95	0.23	2.908	68.03	0.23	3.049	67.28	0.23
			2.878	72.58	0.23	2.927	68.55	0.23	3.069	67.85	0.23
			2.896	73.28	0.23	2.946	69.15	0.23	3.088	68.40	0.23
			2.915	73.93	0.23	2.965	69.77	0.23	3.108	68.93	0.23
			2.933	74.56	0.23	2.983	70.35	0.23	3.128	69.48	0.23
			2.952	75.26	0.23	3.002	70.94	0.23	3.147	70.03	0.23
			2.970	75.90	0.23	3.021	71.52	0.23	3.167	70.61	0.23
			2.988	76.61	0.23	3.040	72.12	0.23	3.187	71.20	0.23
			3.007	77.27	0.23	3.058	72.72	0.23	3.206	71.77	0.23
			3.025	77.92	0.23	3.077	73.29	0.23	3.226	72.31	0.23
			3.044	78.65	0.23	3.096	73.86	0.23	3.246	72.89	0.23
			3.062	79.30	0.23	3.114	74.49	0.23	3.265	73.49	0.23
			3.080	80.02	0.23	3.133	75.09	0.23	3.285	74.06	0.23
			3.099	80.75	0.23	3.152	75.67	0.23	3.305	74.63	0.23
			3.117	81.48	0.23	3.171	76.33	0.23	3.324	75.24	0.23
			3.136	82.11	0.23	3.189	76.92	0.23	3.344	75.81	0.23
			3.154	82.90	0.23	3.208	77.59	0.23	3.364	76.39	0.23
			3.172	83.62	0.23	3.227	78.19	0.23	3.383	77.00	0.23
			3.191	84.34	0.23	3.245	78.81	0.23	3.403	77.58	0.23
			3.209	85.07	0.23	3.264	79.47	0.23	3.423	78.17	0.23

			3.228	85.86	0.23	3.283	80.08	0.23	3.442	78.78	0.23
			3.246	86.57	0.23	3.302	80.71	0.23	3.462	79.43	0.23
			3.264	87.33	0.23	3.320	81.37	0.23	3.482	79.98	0.23
			3.283	88.09	0.23	3.339	82.02	0.23	3.501	80.54	0.23
			3.301	88.86	0.23	3.358	82.68	0.23	3.521	81.16	0.23
			3.320	89.65	0.23	3.377	83.31	0.23	3.541	81.85	0.23
			3.338	90.46	0.23	3.395	83.96	0.23	3.560	82.48	0.23
			3.356	91.28	0.23	3.414	84.62	0.23	3.580	83.10	0.23
			3.375	92.11	0.23	3.433	85.27	0.23	3.600	83.72	0.23
			3.393	92.84	0.23	3.451	85.94	0.23	3.619	84.33	0.23
			3.412	93.70	0.23	3.470	86.60	0.23	3.639	84.98	0.23
			3.430	94.43	0.23	3.489	87.31	0.23	3.659	85.66	0.23
			3.448	95.33	0.23	3.508	87.99	0.23	3.678	86.29	0.23
			3.467	96.27	0.23	3.526	88.67	0.23	3.698	86.91	0.23
						3.545	89.37	0.23	3.718	87.53	0.23
						3.564	90.06	0.23	3.737	88.15	0.23
						3.582	90.75	0.23	3.757	88.83	0.23
						3.601	91.45	0.23	3.777	89.47	0.23
						3.620	92.18	0.23	3.796	90.10	0.23
						3.639	92.88	0.23	3.816	90.75	0.23
						3.657	93.59	0.23	3.836	91.42	0.23
						3.676	94.30	0.23	3.855	92.07	0.23
						3.695	95.03	0.23	3.875	92.75	0.23
						3.714	95.79	0.23	3.894	93.42	0.23
						3.732	96.57	0.23	3.914	94.08	0.23
						3.751	97.30	0.23	3.934	94.76	0.23
						3.770	98.03	0.23	3.953	95.45	0.23
						3.788	98.77	0.23	3.973	96.13	0.23
						3.807	99.55	0.23	3.993	96.82	0.23
						3.826	100.34	0.23	4.012	97.54	0.23
						3.845	101.09	0.23	4.032	98.23	0.23
						3.863	101.84	0.23	4.052	98.92	0.23
						3.882	102.65	0.23	4.071	99.61	0.23
						3.901	103.46	0.23	4.091	100.32	0.23
						3.920	104.25	0.23	4.111	101.02	0.23
						3.938	105.03	0.23	4.130	101.74	0.23
						3.957	105.87	0.23	4.150	102.52	0.23
						3.976	106.70	0.23	4.170	103.25	0.23
						3.994	107.54	0.23	4.189	103.97	0.23
						4.013	108.38	0.23	4.209	104.69	0.23
						4.032	109.19	0.23	4.229	105.42	0.23
						4.051	110.06	0.23	4.248	106.17	0.23
						4.069	110.93	0.23	4.268	106.90	0.23
						4.088	111.78	0.23	4.288	107.67	0.23
						4.107	112.70	0.23	4.307	108.44	0.23

						4.125	113.60	0.23	4.327	109.21	0.23
						4.144	114.44	0.23	4.347	109.98	0.23
						4.163	115.35	0.23	4.366	110.70	0.23
						4.182	116.27	0.23	4.386	111.49	0.23
						4.200	117.20	0.23	4.406	112.29	0.23
						4.219	118.08	0.23	4.425	113.10	0.23
						4.238	119.04	0.23	4.445	113.89	0.23
						4.257	120.00	0.23	4.465	114.68	0.23
						4.275	120.96	0.23	4.484	115.53	0.23
						4.294	121.86	0.23	4.504	116.33	0.23
						4.313	122.85	0.23	4.524	117.14	0.23
						4.331	123.88	0.23	4.543	117.96	0.23
						4.350	124.97	0.23	4.563	118.78	0.23
						4.369	125.93	0.23	4.583	119.64	0.23
						4.388	126.90	0.23	4.602	120.52	0.23
						4.406	127.96	0.23	4.622	121.35	0.23
						4.425	129.08	0.23	4.642	122.20	0.23
						4.444	130.14	0.23	4.661	123.07	0.23
						4.462	131.25	0.23	4.681	123.95	0.23
						4.481	132.45	0.23	4.701	124.84	0.23
						4.507	134.14	0.23	4.720	125.70	0.23
									4.740	126.61	0.23
									4.760	127.54	0.23
									4.779	128.43	0.23
									4.799	129.33	0.23
									4.819	130.28	0.23
									4.838	131.24	0.23
									4.858	132.16	0.23
									4.878	133.11	0.23
									4.897	134.09	0.23
									4.917	135.06	0.23
									4.937	136.02	0.23
									4.956	136.99	0.23
									4.976	138.02	0.23
									4.995	139.01	0.23
									5.015	140.08	0.23
									5.035	141.08	0.23
									5.054	142.15	0.23
									5.074	143.21	0.23
									5.094	144.26	0.23
									5.113	145.37	0.23
									5.133	146.48	0.23
									5.153	147.56	0.23
									5.172	148.65	0.23
									5.192	149.80	0.23

									5.212	150.95	0.23
									5.231	152.08	0.23
									5.251	153.28	0.23
									5.271	154.47	0.23
									5.290	155.67	0.23
									5.310	156.88	0.23
									5.330	158.12	0.23
									5.349	159.38	0.23
									5.369	160.66	0.23
									5.389	161.98	0.23
									5.408	163.32	0.23
									5.428	164.68	0.23
									5.448	166.04	0.23
									5.467	167.43	0.23
									5.487	168.82	0.23
									5.507	170.25	0.23
									5.526	171.75	0.23
									5.546	173.23	0.23
									5.566	174.69	0.23
									5.585	176.21	0.23
									5.605	177.75	0.23
									5.625	179.37	0.23
									5.644	181.08	0.23
									5.664	182.76	0.23
									5.684	184.52	0.23
									5.703	186.32	0.23
									5.723	188.06	0.23
									5.743	190.17	0.21

Combined standard uncertainties:

$u(T)$= 0.006 K; $u(p)$= 0.0020 MPa for p < 6 MPa; $u(p)$= 0.024 MPa for 6 MPa ≤ p ≤ 70 MPa

$u(x_{CO_2})$= 0.00025; $u(x_{NO})$= 0.000014; $u(x_{SO_2})$= 0.0000023; $u(x_{CO})$= 0.0000043

Table S1 (continued). $p\rho T$ experimental data for the $CO_2 + O_2 + SO_2 + CO$ (Mix 1) and $CO_2 + NO + SO_2 + CO$ (Mix 1) mixtures. $u(\rho)$: combined standard uncertainty.

**Mix 2: x_{CO_2} = 0.99592; x_{NO} = 0.001410; x_{SO_2} = 0.0009100; x_{CO} = 0.0017002**											
***T*= 263.15±0.01 K**			***T*= 273.17±0.01 K**			***T*= 283.18±0.01 K**			***T*= 293.17±0.01 K**		
***p* (MPa)**	**ρ ($kg.m^{-3}$)**	**$u(\rho)$ ($kg.m^{-3}$)**	***p* (MPa)**	**ρ ($kg.m^{-3}$)**	**$u(\rho)$ ($kg.m^{-3}$)**	***p* (MPa)**	**ρ ($kg.m^{-3}$)**	**$u(\rho)$ ($kg.m^{-3}$)**	***p* (MPa)**	**ρ ($kg.m^{-3}$)**	**$u(\rho)$ ($kg.m^{-3}$)**
2.825	981.54	0.38	3.714	927.02	0.36	4.701	861.34	0.35	5.888	773.29	0.33
2.845	981.66	0.38	3.734	927.20	0.36	4.721	861.67	0.35	5.908	774.00	0.33
2.864	981.78	0.38	3.754	927.39	0.36	4.741	862.00	0.35	5.928	774.67	0.33
2.884	981.89	0.38	3.774	927.57	0.36	4.760	862.34	0.35	5.948	775.33	0.33
2.904	982.01	0.38	3.794	927.74	0.36	4.780	862.67	0.35	5.968	776.00	0.33
2.924	982.13	0.38	3.813	927.92	0.36	4.800	863.01	0.35	5.988	776.67	0.33
2.944	982.25	0.38	3.833	928.14	0.36	4.820	863.33	0.35	6.008	777.33	0.33
2.964	982.37	0.38	3.853	928.37	0.36	4.840	863.66	0.35	6.028	778.05	0.33
2.984	982.49	0.38	3.873	928.56	0.36	4.859	863.99	0.35	6.048	778.76	0.33
3.004	982.61	0.38	3.893	928.69	0.36	4.879	864.32	0.35	6.067	779.48	0.33
3.024	982.73	0.38	3.912	928.86	0.36	4.899	864.65	0.35	6.087	780.19	0.33
3.044	982.84	0.38	3.932	929.06	0.36	4.919	864.94	0.35	6.107	780.91	0.33
3.064	982.94	0.38	3.952	929.26	0.36	4.939	865.23	0.35	6.127	781.53	0.33
3.084	983.05	0.38	3.972	929.47	0.36	4.958	865.53	0.35	6.147	782.15	0.33
3.104	983.17	0.38	3.992	929.64	0.36	4.978	865.82	0.35	6.167	782.77	0.33
3.123	983.28	0.38	4.011	929.80	0.36	4.998	866.12	0.35	6.187	783.39	0.33
3.143	983.40	0.38	4.031	929.94	0.36	5.018	866.41	0.35	6.207	784.01	0.33
3.163	983.51	0.38	4.051	930.16	0.36	5.038	866.71	0.35	6.227	784.66	0.33
3.183	983.63	0.38	4.071	930.37	0.36	5.058	867.01	0.35	6.247	785.31	0.33
3.203	983.74	0.38	4.091	930.56	0.36	5.077	867.31	0.35	6.266	785.95	0.33
3.223	983.86	0.38	4.111	930.72	0.36	5.097	867.61	0.35	6.286	786.60	0.33
3.243	983.97	0.38	4.130	930.88	0.36	5.117	867.91	0.35	6.306	787.25	0.33
3.263	984.09	0.38	4.150	931.05	0.36	5.137	868.21	0.35	6.326	787.85	0.33
3.283	984.20	0.38	4.170	931.24	0.36	5.157	868.51	0.35	6.346	788.45	0.33
3.303	984.32	0.38	4.190	931.43	0.36	5.176	868.81	0.35	6.366	789.05	0.33
3.323	984.43	0.38	4.210	931.60	0.36	5.196	869.11	0.35	6.386	789.66	0.33
3.343	984.55	0.38	4.229	931.73	0.36	5.216	869.39	0.35	6.406	790.26	0.33
3.363	984.66	0.38	4.249	931.91	0.37	5.236	869.66	0.35	6.426	790.85	0.33
3.382	984.78	0.38	4.269	932.13	0.37	5.256	869.94	0.35	6.446	791.45	0.33
3.402	984.90	0.38	4.289	932.30	0.37	5.276	870.22	0.35	6.466	792.04	0.33
3.422	985.01	0.38	4.309	932.48	0.37	5.295	870.50	0.35	6.485	792.63	0.33
3.442	985.13	0.38	4.328	932.63	0.37	5.315	870.77	0.35	6.505	793.23	0.33
3.462	985.24	0.38	4.348	932.79	0.37	5.335	871.05	0.35	6.525	793.76	0.33
3.482	985.36	0.38	4.368	932.99	0.37	5.355	871.33	0.35	6.545	794.30	0.33
3.502	985.47	0.38	4.388	933.15	0.37	5.375	871.61	0.35	6.565	794.83	0.33

3.522	985.59	0.38	4.408	933.26	0.37	5.394	871.89	0.35	6.585	795.37	0.33
3.542	985.70	0.38	4.428	933.38	0.37	5.414	872.17	0.35	6.605	795.90	0.33
3.562	985.82	0.38	4.447	933.62	0.37	5.434	872.46	0.35	6.625	796.41	0.33
3.582	985.93	0.38	4.467	933.83	0.37	5.454	872.74	0.35	6.645	796.91	0.33
3.602	986.05	0.38	4.487	934.00	0.37	5.474	872.99	0.35	6.665	797.41	0.33
3.622	986.16	0.38	4.507	934.16	0.37	5.494	873.25	0.35	6.684	797.92	0.33
3.642	986.28	0.38	4.527	934.31	0.37	5.513	873.51	0.35	6.704	798.42	0.33
3.661	986.39	0.38	4.546	934.47	0.37	5.533	873.76	0.35	6.724	798.94	0.33
3.681	986.51	0.38	4.566	934.62	0.37	5.553	874.02	0.35	6.744	799.45	0.33
3.701	986.62	0.38	4.586	934.77	0.37	5.573	874.28	0.35	6.764	799.97	0.33
3.721	986.74	0.38	4.606	934.92	0.37	5.593	874.54	0.35	6.784	800.49	0.33
3.741	986.85	0.38	4.626	935.08	0.37	5.612	874.79	0.35	6.804	801.00	0.33
3.761	986.97	0.38	4.645	935.23	0.37	5.632	875.05	0.35	6.824	801.46	0.33
3.781	987.08	0.38	4.665	935.38	0.37	5.652	875.31	0.35	6.844	801.91	0.33
3.801	987.20	0.38	4.685	935.53	0.37	5.672	875.57	0.35	6.864	802.36	0.33
3.821	987.31	0.38	4.705	935.69	0.37	5.692	875.83	0.35	6.883	802.81	0.33
3.841	987.43	0.38	4.725	935.84	0.37	5.712	876.10	0.35	6.903	803.26	0.33
3.861	987.54	0.38	4.745	935.99	0.37	5.731	876.36	0.35	6.923	803.74	0.33
3.881	987.66	0.38	4.764	936.14	0.37	5.751	876.62	0.35	6.943	804.21	0.33
3.901	987.77	0.38	4.784	936.30	0.37	5.771	876.88	0.35	6.963	804.69	0.34
3.920	987.89	0.38	4.804	936.45	0.37	5.791	877.14	0.35	6.983	805.17	0.34
3.940	988.00	0.38	4.824	936.60	0.37	5.811	877.39	0.35	7.003	805.64	0.34
3.960	988.12	0.38	4.844	936.76	0.37	5.830	877.63	0.35	7.023	806.06	0.34
3.980	988.23	0.38	4.863	936.91	0.37	5.850	877.88	0.35	7.043	806.50	0.34
4.000	988.35	0.38	4.883	937.06	0.37	5.870	878.13	0.35	7.063	806.92	0.34
4.020	988.46	0.38	4.903	937.21	0.37	5.890	878.37	0.35	7.083	807.31	0.34
4.040	988.58	0.38	4.923	937.37	0.37	5.910	878.62	0.35	7.102	807.71	0.34
4.060	988.70	0.38	4.943	937.52	0.37	5.930	878.88	0.35	7.122	808.12	0.34
4.080	988.81	0.38	4.962	937.67	0.37	5.949	879.13	0.35	7.142	808.50	0.34
4.100	988.93	0.38	4.982	937.82	0.37	5.969	879.38	0.35	7.162	808.92	0.34
4.120	989.04	0.38	5.002	937.98	0.37	5.989	879.63	0.35	7.182	809.34	0.34
4.140	989.16	0.38	5.022	938.13	0.37	6.009	879.88	0.35	7.202	809.75	0.34
4.160	989.27	0.38	5.042	938.28	0.37	6.029	880.13	0.35	7.222	810.16	0.34
4.179	989.39	0.38	5.062	938.43	0.37	6.048	880.38	0.35	7.242	810.56	0.34
4.199	989.50	0.38	5.081	938.59	0.37	6.068	880.62	0.35	7.262	810.96	0.34
4.219	989.62	0.38	5.101	938.74	0.37	6.088	880.87	0.35	7.282	811.36	0.34
4.239	989.73	0.38	5.121	938.89	0.37	6.108	881.11	0.35	7.301	811.71	0.34
4.259	989.85	0.38	5.141	939.05	0.37	6.128	881.36	0.35	7.321	812.12	0.34
4.279	989.96	0.38	5.161	939.20	0.37	6.148	881.60	0.35	7.341	812.53	0.34
4.299	990.08	0.38	5.180	939.35	0.37	6.167	881.85	0.35	7.361	812.92	0.34
4.319	990.19	0.38	5.200	939.50	0.37	6.187	882.09	0.35	7.381	813.28	0.34
4.339	990.31	0.38	5.220	939.66	0.37	6.207	882.34	0.35	7.401	813.69	0.34
4.359	990.42	0.38	5.240	939.81	0.37	6.227	882.60	0.35	7.421	814.10	0.34
4.379	990.54	0.38	5.260	939.96	0.37	6.247	882.84	0.35	7.441	814.50	0.34
4.399	990.65	0.38	5.279	940.11	0.37	6.266	883.08	0.35	7.461	814.85	0.34

4.419	990.78	0.38	5.299	940.27	0.37	6.286	883.33	0.35	7.481	815.21	0.34
4.439	990.90	0.38	5.319	940.42	0.37	6.306	883.57	0.35	7.501	815.60	0.34
4.458	991.02	0.38	5.339	940.57	0.37	6.326	883.79	0.35	7.520	815.99	0.34
4.478	991.14	0.38	5.359	940.72	0.37	6.346	884.01	0.35	7.540	816.32	0.34
4.498	991.27	0.38	5.379	940.88	0.37	6.366	884.23	0.35	7.560	816.67	0.34
4.518	991.39	0.38	5.398	941.03	0.37	6.385	884.45	0.35	7.580	817.05	0.34
4.538	991.51	0.38	5.418	941.18	0.37	6.405	884.67	0.35	7.600	817.42	0.34
4.558	991.64	0.38	5.438	941.34	0.37	6.425	884.89	0.35	7.620	817.78	0.34
4.578	991.76	0.38	5.458	941.49	0.37	6.445	885.09	0.35	7.640	818.13	0.34
4.598	991.89	0.38	5.478	941.64	0.37	6.465	885.32	0.35	7.660	818.49	0.34
4.618	992.01	0.38	5.497	941.79	0.37	6.484	885.58	0.35	7.680	818.85	0.34
4.638	992.14	0.38	5.517	941.95	0.37	6.504	885.81	0.35	7.700	819.22	0.34
4.658	992.20	0.38	5.537	942.10	0.37	6.524	886.02	0.35	7.719	819.58	0.34
4.678	992.31	0.38	5.557	942.25	0.37	6.544	886.25	0.35	7.739	819.92	0.34
4.698	992.44	0.38	5.577	942.40	0.37	6.564	886.50	0.35	7.759	820.22	0.34
4.717	992.56	0.38	5.596	942.56	0.37	6.584	886.70	0.35	7.779	820.56	0.34
4.737	992.62	0.38	5.616	942.71	0.37	6.603	886.91	0.35	7.799	820.90	0.34
4.757	992.75	0.38	5.636	942.86	0.37	6.623	887.15	0.35	7.819	821.23	0.34
4.777	992.85	0.38	5.656	943.01	0.37	6.643	887.33	0.35	7.839	821.56	0.34
4.797	992.95	0.38	5.676	943.17	0.37	6.663	887.55	0.35	7.859	821.96	0.34
4.817	993.08	0.38	5.696	943.32	0.37	6.683	887.77	0.35	7.879	822.31	0.34
4.837	993.18	0.38	5.715	943.47	0.37	6.702	887.97	0.35	7.899	822.63	0.34
4.857	993.32	0.38	5.735	943.64	0.37	6.722	888.20	0.35	7.918	822.95	0.34
4.877	993.41	0.38	5.755	943.78	0.37	6.742	888.45	0.35	7.938	823.27	0.34
4.897	993.56	0.38	5.775	943.96	0.37	6.762	888.66	0.35	7.958	823.64	0.34
4.917	993.65	0.38	5.795	944.07	0.37	6.782	888.86	0.35	7.978	823.98	0.34
4.937	993.80	0.38	5.814	944.25	0.37	6.802	889.08	0.35	7.998	824.30	0.34
4.957	993.88	0.38	5.834	944.40	0.37	6.821	889.35	0.35	8.018	824.63	0.34
4.976	993.96	0.38	5.854	944.60	0.37	6.841	889.53	0.35	8.038	824.93	0.34
4.996	994.11	0.38	5.874	944.79	0.37	6.861	889.72	0.35	8.058	825.25	0.34
5.016	994.19	0.38	5.894	944.90	0.37	6.881	889.95	0.36	8.078	825.57	0.34
5.036	994.33	0.38	5.913	945.00	0.37	6.901	890.20	0.36	8.098	825.90	0.34
5.056	994.41	0.38	5.933	945.23	0.37	6.920	890.38	0.36	8.118	826.24	0.34
5.076	994.53	0.38	5.953	945.34	0.37	6.940	890.57	0.36	8.137	826.53	0.34
5.096	994.64	0.38	5.973	945.47	0.37	6.960	890.82	0.36	8.157	826.82	0.34
5.116	994.73	0.38	5.993	945.70	0.37	6.980	891.06	0.36	8.177	827.18	0.34
5.136	994.87	0.38	6.013	945.85	0.37	7.000	891.21	0.36	8.197	827.54	0.34
5.156	995.00	0.38	6.032	945.96	0.37	7.020	891.44	0.36	8.217	827.88	0.34
5.176	995.08	0.38	6.052	946.10	0.37	7.039	891.66	0.36	8.237	828.19	0.34
5.196	995.21	0.38	6.072	946.29	0.37	7.059	891.84	0.36	8.257	828.51	0.34
5.216	995.31	0.38	6.092	946.41	0.37	7.079	892.08	0.36	8.277	828.82	0.34
5.236	995.40	0.38	6.112	946.51	0.37	7.099	892.29	0.36	8.297	829.16	0.34
5.255	995.53	0.38	6.131	946.70	0.37	7.119	892.50	0.36	8.317	829.51	0.34
5.275	995.61	0.38	6.151	946.84	0.37	7.138	892.68	0.36	8.336	829.82	0.34
5.295	995.75	0.38	6.171	946.96	0.37	7.158	892.90	0.36	8.356	830.15	0.34

5.315	995.83	0.38	6.191	947.09	0.37	7.178	893.13	0.36	8.376	830.51	0.34
5.335	995.95	0.38	6.211	947.23	0.37	7.198	893.32	0.36	8.396	830.82	0.34
5.355	996.09	0.38	6.230	947.39	0.37	7.218	893.50	0.36	8.416	831.12	0.34
5.375	996.19	0.38	6.250	947.53	0.37	7.238	893.70	0.36	8.436	831.42	0.34
5.395	996.27	0.38	6.270	947.66	0.37	7.257	893.91	0.36	8.456	831.71	0.34
5.415	996.41	0.38	6.290	947.82	0.37	7.277	894.13	0.36	8.476	832.07	0.34
5.435	996.54	0.38	6.310	948.00	0.37	7.297	894.33	0.36	8.496	832.37	0.34
5.455	996.63	0.38	6.330	948.16	0.37	7.317	894.52	0.36	8.516	832.70	0.34
5.475	996.73	0.38	6.349	948.27	0.37	7.337	894.73	0.36	8.536	833.02	0.34
5.495	996.85	0.38	6.369	948.45	0.37	7.356	894.90	0.36	8.555	833.30	0.34
5.514	996.93	0.38	6.389	948.63	0.37	7.376	895.12	0.36	8.575	833.58	0.34
5.534	997.07	0.38	6.409	948.75	0.37	7.396	895.34	0.36	8.595	833.92	0.34
5.554	997.14	0.38	6.429	948.85	0.37	7.416	895.55	0.36	8.615	834.21	0.34
5.574	997.26	0.38	6.448	948.98	0.37	7.436	895.78	0.36	8.635	834.53	0.34
5.594	997.40	0.38	6.468	949.13	0.37	7.456	895.95	0.36	8.655	834.83	0.34
5.614	997.51	0.38	6.488	949.28	0.37	7.475	896.11	0.36	8.675	835.12	0.34
5.634	997.57	0.38	6.508	949.45	0.37	7.495	896.28	0.36	8.695	835.44	0.34
5.654	997.70	0.38	6.528	949.60	0.37	7.515	896.52	0.36	8.715	835.71	0.34
5.674	997.78	0.38	6.547	949.71	0.37	7.535	896.75	0.36	8.735	836.01	0.34
5.694	997.88	0.38	6.567	949.82	0.37	7.555	896.91	0.36	8.754	836.30	0.34
5.714	998.01	0.38	6.587	949.98	0.37	7.574	897.09	0.36	8.774	836.55	0.34
5.734	998.14	0.38	6.607	950.16	0.37	7.594	897.34	0.36	8.794	836.87	0.34
5.754	998.26	0.38	6.627	950.27	0.37	7.614	897.51	0.36	8.814	837.18	0.34
5.774	998.34	0.38	6.647	950.41	0.37	7.634	897.69	0.36	8.834	837.47	0.34
5.793	998.41	0.38	6.666	950.55	0.37	7.654	897.87	0.36	8.854	837.74	0.34
5.813	998.53	0.38	6.686	950.69	0.37	7.673	898.08	0.36	8.874	838.05	0.34
5.833	998.65	0.38	6.706	950.84	0.37	7.693	898.30	0.36	8.894	838.31	0.34
5.853	998.77	0.38	6.726	950.97	0.37	7.713	898.51	0.36	8.914	838.60	0.34
5.873	998.88	0.38	6.746	951.09	0.37	7.733	898.71	0.36	8.934	838.88	0.34
5.893	998.94	0.38	6.765	951.21	0.37	7.753	898.88	0.36	8.953	839.12	0.34
5.913	999.04	0.38	6.785	951.34	0.37	7.773	899.06	0.36	8.973	839.43	0.34
5.933	999.15	0.38	6.805	951.49	0.37	7.792	899.25	0.36	8.993	839.70	0.34
5.953	999.22	0.38	6.825	951.64	0.37	7.812	899.42	0.36	9.013	839.98	0.34
5.973	999.35	0.38	6.845	951.80	0.37	7.832	899.63	0.36	9.033	840.29	0.34
5.993	999.48	0.38	6.864	951.94	0.37	7.852	899.83	0.36	9.053	840.56	0.34
6.013	999.57	0.38	6.884	952.05	0.37	7.872	900.05	0.36	9.073	840.82	0.34
6.033	999.66	0.38	6.904	952.17	0.37	7.891	900.27	0.36	9.093	841.09	0.34
6.052	999.79	0.38	6.924	952.33	0.37	7.911	900.39	0.36	9.113	841.36	0.34
6.072	999.85	0.38	6.944	952.48	0.37	7.931	900.56	0.36	9.133	841.64	0.34
6.092	999.98	0.38	6.964	952.60	0.37	7.951	900.77	0.36	9.153	841.96	0.34
6.112	1000.07	0.38	6.983	952.77	0.37	7.971	901.01	0.36	9.172	842.24	0.34
6.132	1000.17	0.38	7.003	952.90	0.37	7.991	901.18	0.36	9.192	842.50	0.34
6.152	1000.29	0.38	7.023	953.04	0.37	8.010	901.36	0.36	9.212	842.76	0.34
6.172	1000.36	0.38	7.043	953.15	0.37	8.030	901.55	0.36	9.232	843.03	0.34
6.192	1000.50	0.38	7.063	953.32	0.37	8.050	901.75	0.36	9.252	843.29	0.34

6.212	1000.58	0.38	7.082	953.48	0.37	8.070	901.96	0.36	9.272	843.56	0.34
6.232	1000.71	0.38	7.102	953.62	0.37	8.090	902.13	0.36	9.292	843.81	0.34
6.252	1000.81	0.38	7.122	953.74	0.37	8.109	902.27	0.36	9.312	844.10	0.34
6.272	1000.90	0.38	7.142	953.85	0.37	8.129	902.55	0.36	9.332	844.41	0.34
6.292	1001.03	0.38	7.162	953.98	0.37	8.149	902.71	0.36	9.352	844.67	0.34
6.311	1001.10	0.38	7.181	954.11	0.37	8.169	902.86	0.36	9.371	844.94	0.34
6.331	1001.23	0.38	7.201	954.21	0.37	8.189	903.04	0.36	9.391	845.20	0.34
6.351	1001.32	0.38	7.221	954.41	0.37	8.209	903.23	0.36	9.411	845.45	0.34
6.371	1001.39	0.38	7.241	954.52	0.37	8.228	903.43	0.36	9.431	845.71	0.34
6.391	1001.50	0.38	7.261	954.64	0.37	8.248	903.63	0.36	9.451	845.98	0.34
6.411	1001.64	0.38	7.281	954.81	0.37	8.268	903.79	0.36	9.471	846.24	0.34
6.431	1001.77	0.38	7.300	954.96	0.37	8.288	903.97	0.36	9.491	846.50	0.34
6.451	1001.84	0.38	7.320	955.07	0.37	8.308	904.17	0.36	9.511	846.75	0.34
6.471	1001.98	0.38	7.340	955.18	0.37	8.327	904.37	0.36	9.531	847.00	0.34
6.491	1002.06	0.38	7.360	955.30	0.37	8.347	904.52	0.36	9.551	847.25	0.34
6.511	1002.17	0.38	7.380	955.44	0.37	8.367	904.68	0.36	9.570	847.50	0.34
6.531	1002.28	0.38	7.399	955.61	0.37	8.387	904.87	0.36	9.590	847.76	0.34
6.551	1002.35	0.38	7.419	955.74	0.37	8.407	905.07	0.36	9.610	848.01	0.34
6.571	1002.42	0.38	7.439	955.85	0.37	8.427	905.27	0.36	9.630	848.32	0.34
6.590	1002.55	0.38	7.459	955.98	0.37	8.446	905.46	0.36	9.650	848.53	0.34
6.610	1002.68	0.38	7.479	956.12	0.37	8.466	905.59	0.36	9.670	848.75	0.34
6.630	1002.78	0.38	7.498	956.25	0.37	8.486	905.79	0.36	9.690	849.01	0.35
6.650	1002.88	0.38	7.518	956.40	0.37	8.506	906.00	0.36	9.710	849.27	0.35
6.670	1002.99	0.38	7.538	956.56	0.37	8.526	906.20	0.36	9.730	849.53	0.35
6.690	1003.06	0.38	7.558	956.70	0.37	8.545	906.32	0.36	9.750	849.79	0.35
6.710	1003.18	0.38	7.578	956.81	0.37	8.565	906.50	0.36	9.770	850.05	0.35
6.730	1003.27	0.38	7.598	956.93	0.37	8.585	906.71	0.36	9.789	850.32	0.35
6.750	1003.36	0.38	7.617	957.06	0.37	8.605	906.90	0.36	9.809	850.58	0.35
6.770	1003.48	0.38	7.637	957.19	0.37	8.625	907.06	0.36	9.829	850.83	0.35
6.790	1003.54	0.38	7.657	957.29	0.37	8.645	907.26	0.36	9.849	851.05	0.35
6.810	1003.66	0.38	7.677	957.46	0.37	8.664	907.46	0.36	9.869	851.25	0.35
6.830	1003.78	0.38	7.697	957.64	0.37	8.684	907.64	0.36	9.889	851.51	0.35
6.849	1003.89	0.38	7.716	957.76	0.37	8.704	907.79	0.36	9.909	851.77	0.35
6.869	1003.95	0.38	7.736	957.87	0.37	8.724	907.96	0.36	9.929	852.03	0.35
6.889	1004.06	0.38	7.756	957.99	0.37	8.744	908.14	0.36	9.949	852.29	0.35
6.909	1004.18	0.38	7.776	958.11	0.37	8.763	908.34	0.36	9.969	852.54	0.35
6.929	1004.30	0.38	7.796	958.21	0.37	8.783	908.55	0.36	9.988	852.79	0.35
6.949	1004.35	0.38	7.815	958.34	0.37	8.803	908.72	0.36	10.008	853.03	0.35
6.969	1004.45	0.38	7.835	958.47	0.37	8.823	908.87	0.36	10.028	853.25	0.35
6.989	1004.56	0.38	7.855	958.59	0.37	8.843	909.00	0.36	10.048	853.45	0.35
7.009	1004.68	0.38	7.875	958.70	0.37	8.863	909.17	0.36	10.068	853.70	0.35
7.029	1004.75	0.38	7.895	958.89	0.37	8.882	909.39	0.36	10.088	853.94	0.35
7.049	1004.84	0.38	7.915	959.04	0.37	8.902	909.53	0.36	10.108	854.22	0.35
7.069	1004.95	0.38	7.934	959.14	0.37	8.922	909.68	0.36	10.128	854.51	0.35
7.089	1005.07	0.38	7.954	959.25	0.37	8.942	909.86	0.36	10.148	854.74	0.35

7.108	1005.16	0.38	7.974	959.38	0.37	8.962	910.05	0.36	10.168	854.98	0.35
7.128	1005.23	0.38	7.994	959.51	0.37	8.981	910.23	0.36	10.188	855.22	0.35
7.148	1005.31	0.38	8.014	959.65	0.37	9.001	910.42	0.36	10.207	855.45	0.35
7.168	1005.45	0.38	8.033	959.82	0.37	9.021	910.58	0.36	10.227	855.69	0.35
7.188	1005.53	0.38	8.053	959.93	0.37	9.041	910.74	0.36	10.247	855.92	0.35
7.208	1005.61	0.38	8.073	960.07	0.37	9.061	910.92	0.36	10.267	856.15	0.35
7.228	1005.69	0.38	8.093	960.20	0.37	9.081	911.10	0.36	10.287	856.38	0.35
7.248	1005.85	0.38	8.113	960.33	0.37	9.100	911.28	0.36	10.307	856.62	0.35
7.268	1005.93	0.38	8.132	960.44	0.37	9.120	911.41	0.36	10.327	856.83	0.35
7.288	1006.01	0.38	8.152	960.60	0.37	9.140	911.55	0.36	10.347	857.00	0.35
7.308	1006.15	0.38	8.172	960.72	0.37	9.160	911.71	0.36	10.367	857.25	0.35
7.328	1006.24	0.38	8.192	960.83	0.37	9.180	911.90	0.36	10.387	857.46	0.35
7.348	1006.31	0.38	8.212	960.95	0.37	9.199	912.10	0.36	10.406	857.69	0.35
7.368	1006.44	0.38	8.232	961.07	0.37	9.219	912.23	0.36	10.426	857.95	0.35
7.387	1006.55	0.38	8.251	961.19	0.37	9.239	912.37	0.36	10.446	858.16	0.35
7.407	1006.62	0.38	8.271	961.31	0.37	9.259	912.54	0.36	10.466	858.37	0.35
7.427	1006.70	0.38	8.291	961.48	0.37	9.279	912.71	0.36	10.486	858.64	0.35
7.447	1006.80	0.38	8.311	961.60	0.37	9.299	912.87	0.36	10.506	858.86	0.35
7.467	1006.93	0.38	8.331	961.71	0.37	9.318	913.03	0.36	10.526	859.10	0.35
7.487	1007.00	0.38	8.350	961.83	0.37	9.338	913.20	0.36	10.546	859.35	0.35
7.507	1007.07	0.38	8.370	961.97	0.37	9.358	913.38	0.36	10.566	859.55	0.35
7.527	1007.21	0.38	8.390	962.07	0.37	9.378	913.57	0.36	10.586	859.75	0.35
7.547	1007.30	0.38	8.410	962.20	0.37	9.398	913.74	0.36	10.605	860.00	0.35
7.567	1007.38	0.38	8.430	962.32	0.37	9.417	913.87	0.36	10.625	860.23	0.35
7.587	1007.49	0.38	8.449	962.44	0.37	9.437	913.98	0.36	10.645	860.44	0.35
7.607	1007.61	0.38	8.469	962.57	0.37	9.457	914.20	0.36	10.665	860.66	0.35
7.627	1007.68	0.38	8.489	962.71	0.37	9.477	914.38	0.36	10.685	860.93	0.35
7.646	1007.77	0.38	8.509	962.83	0.37	9.497	914.55	0.36	10.705	861.14	0.35
7.666	1007.91	0.38	8.529	962.94	0.37	9.517	914.70	0.36	10.725	861.35	0.35
7.686	1007.98	0.38	8.548	963.08	0.37	9.536	914.83	0.36	10.745	861.57	0.35
7.706	1008.05	0.38	8.568	963.20	0.37	9.556	915.01	0.36	10.765	861.79	0.35
7.726	1008.19	0.38	8.588	963.32	0.37	9.576	915.18	0.36	10.785	862.02	0.35
7.746	1008.28	0.38	8.608	963.44	0.37	9.596	915.33	0.36	10.805	862.24	0.35
7.766	1008.39	0.38	8.628	963.57	0.37	9.616	915.50	0.36	10.824	862.46	0.35
7.786	1008.50	0.38	8.648	963.68	0.37	9.635	915.66	0.36	10.844	862.68	0.35
7.806	1008.57	0.38	8.667	963.79	0.37	9.655	915.81	0.36	10.864	862.91	0.35
7.826	1008.64	0.38	8.687	963.92	0.37	9.675	916.03	0.36	10.884	863.13	0.35
7.846	1008.78	0.38	8.707	964.03	0.37	9.695	916.18	0.36	10.904	863.35	0.35
7.866	1008.86	0.38	8.727	964.18	0.37	9.715	916.29	0.36	10.924	863.57	0.35
7.886	1008.98	0.38	8.747	964.33	0.37	9.735	916.44	0.36	10.944	863.79	0.35
7.905	1009.08	0.38	8.766	964.44	0.37	9.754	916.64	0.36	10.964	864.00	0.35
7.925	1009.15	0.38	8.786	964.55	0.37	9.774	916.82	0.36	10.984	864.23	0.35
7.945	1009.24	0.38	8.806	964.66	0.37	9.794	916.98	0.36	11.004	864.47	0.35
7.965	1009.38	0.38	8.826	964.78	0.37	9.814	917.15	0.36	11.023	864.70	0.35
7.985	1009.45	0.38	8.846	964.90	0.37	9.834	917.35	0.36	11.043	864.91	0.35

8.005	1009.52	0.38	8.865	965.01	0.37	9.853	917.45	0.36	11.063	865.09	0.35
8.025	1009.64	0.38	8.885	965.12	0.37	9.873	917.61	0.36	11.083	865.32	0.35
8.045	1009.74	0.38	8.905	965.25	0.37	9.893	917.80	0.36	11.103	865.56	0.35
8.065	1009.83	0.38	8.925	965.36	0.37	9.913	918.00	0.36	11.123	865.78	0.35
8.085	1009.96	0.38	8.945	965.47	0.37	9.933	918.16	0.36	11.143	865.95	0.35
8.105	1010.03	0.38	8.965	965.65	0.37	9.953	918.30	0.36	11.163	866.18	0.35
8.125	1010.09	0.38	8.984	965.76	0.37	9.972	918.41	0.36	11.183	866.43	0.35
8.145	1010.21	0.38	9.004	965.84	0.37	9.992	918.61	0.36	11.203	866.65	0.35
8.165	1010.31	0.38	9.024	965.95	0.37	10.012	918.78	0.36	11.222	866.90	0.35
8.184	1010.40	0.38	9.044	966.10	0.37	10.032	918.94	0.36	11.242	867.13	0.35
8.204	1010.53	0.38	9.064	966.21	0.37	10.052	919.10	0.36	11.262	867.35	0.35
8.224	1010.60	0.38	9.083	966.33	0.37	10.071	919.23	0.36	11.282	867.56	0.35
8.244	1010.66	0.38	9.103	966.45	0.37	10.091	919.39	0.36	11.302	867.79	0.35
8.264	1010.78	0.38	9.123	966.59	0.37	10.111	919.56	0.36	11.322	868.00	0.35
8.284	1010.89	0.38	9.143	966.69	0.37	10.131	919.70	0.36	11.342	868.22	0.35
8.304	1010.95	0.38	9.163	966.80	0.37	10.151	919.85	0.36	11.362	868.44	0.35
8.324	1011.03	0.38	9.182	966.95	0.37	10.171	920.03	0.36	11.382	868.66	0.35
8.344	1011.17	0.38	9.202	967.05	0.37	10.190	920.21	0.36	11.402	868.86	0.35
8.364	1011.24	0.38	9.222	967.16	0.37	10.210	920.37	0.36	11.422	869.08	0.35
8.384	1011.37	0.38	9.242	967.26	0.37	10.230	920.51	0.36	11.441	869.29	0.35
8.404	1011.47	0.38	9.262	967.40	0.37	10.250	920.62	0.36	11.461	869.49	0.35
8.424	1011.54	0.38	9.282	967.52	0.37	10.270	920.76	0.36	11.481	869.69	0.35
8.443	1011.62	0.38	9.301	967.63	0.37	10.289	920.94	0.36	11.501	869.90	0.35
8.463	1011.76	0.38	9.321	967.76	0.37	10.309	921.10	0.36	11.521	870.10	0.35
8.483	1011.82	0.38	9.341	967.89	0.37	10.329	921.25	0.36	11.541	870.31	0.35
8.503	1011.89	0.38	9.361	968.01	0.37	10.349	921.40	0.36	11.561	870.50	0.35
8.523	1012.00	0.38	9.381	968.12	0.37	10.369	921.57	0.36	11.581	870.70	0.35
8.543	1012.11	0.38	9.400	968.21	0.37	10.388	921.74	0.36	11.601	870.90	0.35
8.563	1012.18	0.38	9.420	968.31	0.37	10.408	921.91	0.36	11.621	871.09	0.35
8.583	1012.32	0.38	9.440	968.45	0.37	10.428	922.04	0.36	11.640	871.35	0.35
8.603	1012.39	0.38	9.460	968.57	0.37	10.448	922.17	0.36	11.660	871.56	0.35
8.623	1012.46	0.38	9.480	968.68	0.37	10.468	922.34	0.36	11.680	871.75	0.35
8.643	1012.56	0.38	9.499	968.80	0.37	10.488	922.50	0.36	11.700	871.94	0.35
8.663	1012.67	0.38	9.519	968.95	0.37	10.507	922.66	0.36	11.720	872.12	0.35
8.683	1012.74	0.38	9.539	969.07	0.37	10.527	922.78	0.36	11.740	872.35	0.35
8.702	1012.87	0.38	9.559	969.18	0.37	10.547	922.90	0.36	11.760	872.57	0.35
8.722	1012.96	0.38	9.579	969.31	0.37	10.567	923.05	0.36	11.780	872.75	0.35
8.742	1013.02	0.38	9.599	969.44	0.37	10.587	923.20	0.36	11.800	872.98	0.35
8.762	1013.12	0.38	9.618	969.56	0.37	10.606	923.36	0.36	11.820	873.17	0.35
8.782	1013.24	0.38	9.638	969.66	0.37	10.626	923.53	0.36	11.840	873.37	0.35
8.802	1013.30	0.38	9.658	969.72	0.37	10.646	923.70	0.36	11.859	873.60	0.35
8.822	1013.44	0.38	9.678	969.87	0.37	10.666	923.83	0.36	11.879	873.77	0.35
8.842	1013.52	0.38	9.698	970.01	0.37	10.686	923.96	0.36	11.899	873.95	0.35
8.862	1013.59	0.38	9.717	970.14	0.37	10.706	924.11	0.36	11.919	874.17	0.35
8.882	1013.68	0.38	9.737	970.21	0.37	10.725	924.26	0.36	11.939	874.34	0.35

8.902	1013.81	0.38	9.757	970.34	0.37	10.745	924.41	0.36	11.959	874.52	0.35
8.922	1013.88	0.38	9.777	970.48	0.37	10.765	924.54	0.36	11.979	874.73	0.35
8.942	1013.94	0.38	9.797	970.54	0.37	10.785	924.67	0.36	11.999	874.95	0.35
8.962	1014.05	0.38	9.816	970.65	0.37	10.805	924.79	0.36	12.019	875.15	0.35
8.981	1014.16	0.38	9.836	970.77	0.37	10.824	924.93	0.36	12.039	875.31	0.35
9.001	1014.23	0.38	9.856	970.89	0.37	10.844	925.12	0.36	12.058	875.50	0.35
9.021	1014.36	0.39	9.876	971.05	0.37	10.864	925.22	0.36	12.078	875.69	0.35
9.041	1014.43	0.39	9.896	971.15	0.37	10.884	925.36	0.36	12.098	875.88	0.35
9.061	1014.54	0.39	9.916	971.27	0.37	10.904	925.54	0.36	12.118	876.08	0.35
9.081	1014.65	0.39	9.935	971.39	0.37	10.924	925.68	0.36	12.138	876.28	0.35
9.101	1014.71	0.39	9.955	971.44	0.37	10.943	925.85	0.36	12.158	876.44	0.35
9.121	1014.78	0.39	9.975	971.56	0.37	10.963	926.00	0.36	12.178	876.60	0.35
9.141	1014.91	0.39	9.995	971.71	0.37	10.983	926.12	0.36	12.198	876.79	0.35
9.161	1015.00	0.39	10.015	971.84	0.37	11.003	926.26	0.36	12.218	876.99	0.35
9.181	1015.09	0.39	10.034	971.94	0.37	11.023	926.45	0.36	12.238	877.19	0.35
9.201	1015.21	0.39	10.054	972.09	0.37	11.042	926.61	0.36	12.257	877.35	0.35
9.221	1015.27	0.39	10.074	972.15	0.37	11.062	926.75	0.36	12.277	877.51	0.35
9.240	1015.34	0.39	10.094	972.23	0.37	11.082	926.88	0.36	12.297	877.67	0.35
9.260	1015.44	0.39	10.114	972.35	0.37	11.102	927.05	0.36	12.317	877.85	0.35
9.280	1015.55	0.39	10.133	972.50	0.37	11.122	927.20	0.36	12.337	878.04	0.35
9.300	1015.62	0.39	10.153	972.61	0.37	11.142	927.30	0.36	12.357	878.22	0.35
9.320	1015.74	0.39	10.173	972.73	0.37	11.161	927.45	0.36	12.377	878.41	0.35
9.340	1015.82	0.39	10.193	972.86	0.37	11.181	927.61	0.36	12.397	878.60	0.35
9.360	1015.89	0.39	10.213	972.92	0.37	11.201	927.74	0.36	12.417	878.81	0.35
9.380	1015.96	0.39	10.233	973.05	0.37	11.221	927.91	0.36	12.437	879.05	0.35
9.400	1016.05	0.39	10.252	973.18	0.37	11.241	928.04	0.36	12.457	879.23	0.35
9.420	1016.18	0.39	10.272	973.30	0.37	11.260	928.15	0.36	12.476	879.41	0.35
9.440	1016.25	0.39	10.292	973.41	0.37	11.280	928.29	0.36	12.496	879.57	0.35
9.460	1016.32	0.39	10.312	973.54	0.37	11.300	928.47	0.36	12.516	879.71	0.35
9.480	1016.44	0.39	10.332	973.63	0.37	11.320	928.61	0.36	12.536	879.96	0.35
9.499	1016.54	0.39	10.351	973.72	0.37	11.340	928.72	0.36	12.556	880.12	0.35
9.519	1016.61	0.39	10.371	973.83	0.37	11.360	928.86	0.36	12.576	880.28	0.35
9.539	1016.70	0.39	10.391	973.97	0.38	11.379	929.05	0.36	12.596	880.46	0.35
9.559	1016.83	0.39	10.411	974.10	0.38	11.399	929.19	0.36	12.616	880.65	0.35
9.579	1016.90	0.39	10.431	974.19	0.38	11.419	929.29	0.36	12.636	880.84	0.35
9.599	1016.97	0.39	10.450	974.28	0.38	11.439	929.47	0.36	12.656	881.02	0.35
9.619	1017.07	0.39	10.470	974.41	0.38	11.459	929.63	0.36	12.675	881.21	0.35
9.639	1017.18	0.39	10.490	974.51	0.38	11.478	929.77	0.36	12.695	881.38	0.35
9.659	1017.25	0.39	10.510	974.63	0.38	11.498	929.92	0.36	12.715	881.56	0.35
9.679	1017.31	0.39	10.530	974.75	0.38	11.518	930.04	0.36	12.735	881.74	0.35
9.699	1017.43	0.39	10.550	974.85	0.38	11.538	930.17	0.36	12.755	881.92	0.35
9.719	1017.52	0.39	10.569	974.96	0.38	11.558	930.36	0.36	12.775	882.10	0.35
9.739	1017.61	0.39	10.589	975.09	0.38	11.578	930.52	0.36	12.795	882.28	0.35
9.759	1017.74	0.39	10.609	975.23	0.38	11.597	930.65	0.36	12.815	882.46	0.35
9.778	1017.80	0.39	10.629	975.34	0.38	11.617	930.75	0.36	12.835	882.64	0.35

9.798	1017.87	0.39	10.649	975.43	0.38	11.637	930.87	0.36	12.855	882.83	0.35
9.818	1017.97	0.39	10.668	975.52	0.38	11.657	931.03	0.36	12.874	883.01	0.35
9.838	1018.09	0.39	10.688	975.64	0.38	11.677	931.21	0.36	12.894	883.20	0.35
9.858	1018.15	0.39	10.708	975.76	0.38	11.696	931.34	0.36	12.914	883.38	0.35
9.878	1018.22	0.39	10.728	975.90	0.38	11.716	931.47	0.36	12.934	883.57	0.35
9.898	1018.28	0.39	10.748	975.99	0.38	11.736	931.58	0.36	12.954	883.75	0.35
9.918	1018.40	0.39	10.767	976.06	0.38	11.756	931.76	0.36	12.974	883.94	0.35
9.938	1018.50	0.39	10.787	976.18	0.38	11.776	931.92	0.36	12.994	884.12	0.35
9.958	1018.58	0.39	10.807	976.30	0.38	11.796	932.05	0.36	13.014	884.24	0.35
9.978	1018.71	0.39	10.827	976.42	0.38	11.815	932.16	0.36	13.034	884.41	0.35
9.998	1018.78	0.39	10.847	976.54	0.38	11.835	932.27	0.36	13.054	884.60	0.35
10.018	1018.84	0.39	10.867	976.69	0.38	11.855	932.45	0.36	13.074	884.79	0.35
10.037	1018.93	0.39	10.886	976.74	0.38	11.875	932.60	0.37	13.093	884.98	0.35
10.057	1019.05	0.39	10.906	976.81	0.38	11.895	932.75	0.37	13.113	885.17	0.35
10.077	1019.11	0.39	10.926	976.95	0.38	11.914	932.89	0.37	13.133	885.35	0.35
10.097	1019.18	0.39	10.946	977.06	0.38	11.934	933.01	0.37	13.153	885.52	0.35
10.117	1019.27	0.39	10.966	977.18	0.38	11.954	933.12	0.37	13.173	885.63	0.35
10.137	1019.39	0.39	10.985	977.28	0.38	11.974	933.27	0.37	13.193	885.79	0.35
10.157	1019.45	0.39	11.005	977.38	0.38	11.994	933.42	0.37	13.213	885.97	0.35
10.177	1019.58	0.39	11.025	977.49	0.38	12.014	933.55	0.37	13.233	886.14	0.35
10.197	1019.67	0.39	11.045	977.63	0.38	12.033	933.67	0.37	13.253	886.31	0.35
10.217	1019.73	0.39	11.065	977.72	0.38	12.053	933.78	0.37	13.273	886.49	0.35
10.237	1019.81	0.39	11.084	977.81	0.38	12.073	933.94	0.37	13.292	886.66	0.35
10.257	1019.95	0.39	11.104	977.91	0.38	12.093	934.10	0.37	13.312	886.84	0.35
10.277	1020.01	0.39	11.124	978.04	0.38	12.113	934.24	0.37	13.332	887.02	0.35
10.296	1020.07	0.39	11.144	978.13	0.38	12.132	934.35	0.37	13.352	887.19	0.35
10.316	1020.14	0.39	11.164	978.21	0.38	12.152	934.45	0.37	13.372	887.37	0.35
10.336	1020.22	0.39	11.184	978.36	0.38	12.172	934.62	0.37	13.392	887.49	0.35
10.356	1020.35	0.39	11.203	978.49	0.38	12.192	934.77	0.37	13.412	887.64	0.35
10.376	1020.41	0.39	11.223	978.55	0.38	12.212	934.91	0.37	13.432	887.82	0.35
10.396	1020.48	0.39	11.243	978.65	0.38	12.232	935.04	0.37	13.452	888.02	0.35
10.416	1020.57	0.39	11.263	978.79	0.38	12.251	935.17	0.37	13.472	888.15	0.35
10.436	1020.69	0.39	11.283	978.91	0.38	12.271	935.28	0.37	13.492	888.33	0.35
10.456	1020.76	0.39	11.302	979.02	0.38	12.291	935.39	0.37	13.511	888.51	0.35
10.476	1020.88	0.39	11.322	979.11	0.38	12.311	935.53	0.37	13.531	888.66	0.35
10.496	1020.97	0.39	11.342	979.19	0.38	12.331	935.66	0.37	13.551	888.86	0.35
10.516	1021.03	0.39	11.362	979.30	0.38	12.350	935.78	0.37	13.571	889.00	0.35
10.536	1021.10	0.39	11.382	979.42	0.38	12.370	935.92	0.37	13.591	889.13	0.35
10.556	1021.22	0.39	11.401	979.55	0.38	12.390	936.08	0.37	13.611	889.31	0.35
10.575	1021.30	0.39	11.421	979.66	0.38	12.410	936.23	0.37	13.631	889.48	0.35
10.595	1021.36	0.39	11.441	979.72	0.38	12.430	936.36	0.37	13.651	889.63	0.35
10.615	1021.43	0.39	11.461	979.87	0.38	12.450	936.45	0.37	13.671	889.84	0.35
10.635	1021.56	0.39	11.481	979.94	0.38	12.469	936.57	0.37	13.691	889.98	0.35
10.655	1021.64	0.39	11.501	980.03	0.38	12.489	936.74	0.37	13.710	890.12	0.35
10.675	1021.70	0.39	11.520	980.18	0.38	12.509	936.84	0.37	13.730	890.27	0.35

10.695	1021.78	0.39	11.540	980.30	0.38	12.529	936.96	0.37	13.750	890.45	0.35
10.715	1021.91	0.39	11.560	980.41	0.38	12.549	937.10	0.37	13.770	890.57	0.35
10.735	1021.97	0.39	11.580	980.50	0.38	12.568	937.22	0.37	13.790	890.75	0.35
10.755	1022.07	0.39	11.600	980.58	0.38	12.588	937.34	0.37	13.810	890.89	0.35
10.775	1022.17	0.39	11.619	980.69	0.38	12.608	937.49	0.37	13.830	891.11	0.35
10.795	1022.24	0.39	11.639	980.82	0.38	12.628	937.66	0.37	13.850	891.23	0.35
10.815	1022.30	0.39	11.659	980.91	0.38	12.648	937.79	0.37	13.870	891.44	0.35
10.834	1022.36	0.39	11.679	981.00	0.38	12.668	937.89	0.37	13.890	891.60	0.35
10.854	1022.45	0.39	11.699	981.11	0.38	12.687	938.03	0.37	13.909	891.77	0.35
10.874	1022.57	0.39	11.718	981.24	0.38	12.707	938.15	0.37	13.929	891.97	0.35
10.894	1022.63	0.39	11.738	981.32	0.38	12.727	938.28	0.37	13.949	892.09	0.35
10.914	1022.73	0.39	11.758	981.39	0.38	12.747	938.42	0.37	13.969	892.29	0.35
10.934	1022.83	0.39	11.778	981.53	0.38	12.767	938.55	0.37	13.989	892.44	0.36
10.954	1022.89	0.39	11.798	981.66	0.38	12.786	938.67	0.37	14.009	892.62	0.36
10.974	1022.95	0.39	11.818	981.76	0.38	12.806	938.80	0.37	14.029	892.81	0.36
10.994	1023.07	0.39	11.837	981.86	0.38	12.826	938.94	0.37	14.049	892.94	0.36
11.014	1023.16	0.39	11.857	981.93	0.38	12.846	939.07	0.37	14.069	893.15	0.36
11.034	1023.23	0.39	11.877	982.04	0.38	12.866	939.18	0.37	14.089	893.27	0.36
11.054	1023.29	0.39	11.897	982.15	0.38	12.886	939.30	0.37	14.109	893.45	0.36
11.074	1023.42	0.39	11.917	982.27	0.38	12.905	939.44	0.37	14.128	893.63	0.36
11.093	1023.51	0.39	11.936	982.41	0.38	12.925	939.58	0.37	14.148	893.78	0.36
11.113	1023.57	0.39	11.956	982.47	0.38	12.945	939.68	0.37	14.168	893.92	0.36
11.133	1023.65	0.39	11.976	982.57	0.38	12.965	939.78	0.37	14.188	894.09	0.36
11.153	1023.78	0.39	11.996	982.71	0.38	12.985	939.96	0.37	14.208	894.28	0.36
11.173	1023.85	0.39	12.016	982.81	0.38	13.004	940.09	0.37	14.228	894.41	0.36
11.193	1023.91	0.39	12.035	982.88	0.38	13.024	940.20	0.37	14.248	894.60	0.36
11.213	1023.99	0.39	12.055	982.96	0.38	13.044	940.34	0.37	14.268	894.74	0.36
11.233	1024.12	0.39	12.075	983.09	0.38	13.064	940.47	0.37	14.288	894.92	0.36
11.253	1024.18	0.39	12.095	983.20	0.38	13.084	940.58	0.37	14.308	895.10	0.36
11.273	1024.24	0.39	12.115	983.32	0.38	13.104	940.71	0.37	14.327	895.24	0.36
11.293	1024.32	0.39	12.135	983.43	0.38	13.123	940.84	0.37	14.347	895.43	0.36
11.313	1024.44	0.39	12.154	983.49	0.38	13.143	940.99	0.37	14.367	895.56	0.36
11.333	1024.51	0.39	12.174	983.61	0.38	13.163	941.06	0.37	14.387	895.74	0.36
11.353	1024.57	0.39	12.194	983.70	0.38	13.183	941.19	0.37	14.407	895.85	0.36
11.372	1024.65	0.39	12.214	983.80	0.38	13.203	941.35	0.37	14.427	896.05	0.36
11.392	1024.78	0.39	12.234	983.91	0.38	13.222	941.50	0.37	14.447	896.19	0.36
11.412	1024.84	0.39	12.253	984.03	0.38	13.242	941.55	0.37	14.467	896.34	0.36
11.432	1024.90	0.39	12.273	984.11	0.38	13.262	941.68	0.37	14.487	896.54	0.36
11.452	1024.99	0.39	12.293	984.20	0.38	13.282	941.86	0.37	14.507	896.72	0.36
11.472	1025.11	0.39	12.313	984.31	0.38	13.302	942.02	0.37	14.527	896.84	0.36
11.492	1025.17	0.39	12.333	984.39	0.38	13.321	942.12	0.37	14.546	897.03	0.36
11.512	1025.23	0.39	12.352	984.50	0.38	13.341	942.26	0.37	14.566	897.17	0.36
11.532	1025.31	0.39	12.372	984.62	0.38	13.361	942.41	0.37	14.586	897.33	0.36
11.552	1025.43	0.39	12.392	984.74	0.38	13.381	942.53	0.37	14.606	897.45	0.36
11.572	1025.49	0.39	12.412	984.80	0.38	13.401	942.62	0.37	14.626	897.65	0.36

11.592	1025.59	0.39	12.432	984.90	0.38	13.421	942.74	0.37	14.646	897.81	0.36
11.612	1025.69	0.39	12.452	985.01	0.38	13.440	942.87	0.37	14.666	897.95	0.36
11.631	1025.75	0.39	12.471	985.14	0.38	13.460	943.01	0.37	14.686	898.14	0.36
11.651	1025.81	0.39	12.491	985.19	0.38	13.480	943.16	0.37	14.706	898.26	0.36
11.671	1025.91	0.39	12.511	985.29	0.38	13.500	943.28	0.37	14.726	898.43	0.36
11.691	1026.02	0.39	12.531	985.41	0.38	13.520	943.40	0.37	14.745	898.62	0.36
11.711	1026.08	0.39	12.551	985.55	0.38	13.539	943.53	0.37	14.765	898.78	0.36
11.731	1026.14	0.39	12.570	985.64	0.38	13.559	943.65	0.37	14.785	898.90	0.36
11.751	1026.23	0.39	12.590	985.75	0.38	13.579	943.76	0.37	14.805	899.08	0.36
11.771	1026.34	0.39	12.610	985.87	0.38	13.599	943.86	0.37	14.825	899.27	0.36
11.791	1026.40	0.39	12.630	985.93	0.38	13.619	944.00	0.37	14.845	899.40	0.36
11.811	1026.50	0.39	12.650	986.04	0.38	13.639	944.13	0.37	14.865	899.56	0.36
11.831	1026.60	0.39	12.669	986.14	0.38	13.658	944.24	0.37	14.885	899.75	0.36
11.851	1026.65	0.39	12.689	986.22	0.38	13.678	944.34	0.37	14.905	899.85	0.36
11.871	1026.71	0.39	12.709	986.32	0.38	13.698	944.47	0.37	14.925	900.04	0.36
11.890	1026.81	0.39	12.729	986.44	0.38	13.718	944.65	0.37	14.944	900.19	0.36
11.910	1026.91	0.39	12.749	986.54	0.38	13.738	944.81	0.37	14.964	900.32	0.36
11.930	1026.97	0.39	12.769	986.61	0.38	13.757	944.91	0.37	14.984	900.50	0.36
11.950	1027.03	0.39	12.788	986.73	0.38	13.777	945.02	0.37	15.004	900.67	0.36
11.970	1027.12	0.39	12.808	986.81	0.38	13.797	945.13	0.37	15.024	900.79	0.36
11.990	1027.23	0.39	12.828	986.90	0.38	13.817	945.26	0.37	15.044	900.95	0.36
12.010	1027.29	0.39	12.848	987.00	0.38	13.837	945.37	0.37	15.064	901.13	0.36
12.030	1027.35	0.39	12.868	987.12	0.38	13.857	945.47	0.37	15.084	901.23	0.36
12.050	1027.44	0.39	12.887	987.21	0.38	13.876	945.58	0.37	15.104	901.41	0.36
12.070	1027.55	0.39	12.907	987.28	0.38	13.896	945.71	0.37	15.124	901.57	0.36
12.090	1027.61	0.39	12.927	987.39	0.38	13.916	945.87	0.37	15.144	901.70	0.36
12.110	1027.71	0.39	12.947	987.52	0.38	13.936	946.02	0.37	15.163	901.88	0.36
12.130	1027.82	0.39	12.967	987.61	0.38	13.956	946.11	0.37	15.183	902.06	0.36
12.150	1027.87	0.39	12.986	987.66	0.38	13.975	946.22	0.37	15.203	902.18	0.36
12.169	1027.93	0.39	13.006	987.79	0.38	13.995	946.33	0.37	15.223	902.33	0.36
12.189	1027.99	0.39	13.026	987.92	0.38	14.015	946.45	0.37	15.243	902.51	0.36
12.209	1028.06	0.39	13.046	988.01	0.38	14.035	946.59	0.37	15.263	902.69	0.36
12.229	1028.19	0.39	13.066	988.07	0.38	14.055	946.73	0.37	15.283	902.78	0.36
12.249	1028.26	0.39	13.086	988.21	0.38	14.075	946.83	0.37	15.303	902.94	0.36
12.269	1028.32	0.39	13.105	988.28	0.38	14.094	946.94	0.37	15.323	903.12	0.36
12.289	1028.40	0.39	13.125	988.35	0.38	14.114	947.05	0.37	15.343	903.28	0.36
12.309	1028.53	0.39	13.145	988.47	0.38	14.134	947.20	0.37	15.362	903.38	0.36
12.329	1028.59	0.39	13.165	988.61	0.38	14.154	947.32	0.37	15.382	903.56	0.36
12.349	1028.65	0.39	13.185	988.69	0.38	14.174	947.42	0.37	15.402	903.71	0.36
12.369	1028.72	0.39	13.204	988.77	0.38	14.193	947.52	0.37	15.422	903.83	0.36
12.389	1028.78	0.39	13.224	988.89	0.38	14.213	947.65	0.37	15.442	904.02	0.36
12.409	1028.91	0.39	13.244	988.95	0.38	14.233	947.82	0.37	15.462	904.20	0.36
12.428	1028.99	0.39	13.264	989.04	0.38	14.253	947.94	0.37	15.482	904.31	0.36
12.448	1029.05	0.39	13.284	989.15	0.38	14.273	948.04	0.37	15.502	904.46	0.36
12.468	1029.12	0.39	13.303	989.27	0.38	14.293	948.10	0.37	15.522	904.63	0.36

12.488	1029.24	0.39	13.323	989.38	0.38	14.312	948.23	0.37	15.542	904.80	0.36
12.508	1029.31	0.39	13.343	989.48	0.38	14.332	948.38	0.37	15.561	904.89	0.36
12.528	1029.37	0.39	13.363	989.55	0.38	14.352	948.53	0.37	15.581	905.04	0.36
12.548	1029.43	0.39	13.383	989.66	0.38	14.372	948.65	0.37	15.601	905.21	0.36
12.568	1029.50	0.39	13.403	989.77	0.38	14.392	948.74	0.37	15.621	905.37	0.36
12.588	1029.61	0.39	13.422	989.87	0.38	14.411	948.86	0.37	15.641	905.55	0.36
12.608	1029.70	0.39	13.442	989.94	0.38	14.431	949.00	0.37	15.661	905.63	0.36
12.628	1029.77	0.39	13.462	990.03	0.38	14.451	949.11	0.37	15.681	905.79	0.36
12.648	1029.83	0.39	13.482	990.12	0.38	14.471	949.21	0.37	15.701	905.96	0.36
12.668	1029.94	0.39	13.502	990.24	0.38	14.491	949.33	0.37	15.721	906.13	0.36
12.688	1030.03	0.39	13.521	990.32	0.38	14.511	949.44	0.37	15.741	906.22	0.36
12.707	1030.10	0.39	13.541	990.39	0.38	14.530	949.55	0.37	15.761	906.38	0.36
12.727	1030.16	0.39	13.561	990.50	0.38	14.550	949.65	0.37	15.780	906.55	0.36
12.747	1030.22	0.39	13.581	990.61	0.38	14.570	949.78	0.37	15.800	906.71	0.36
12.767	1030.33	0.39	13.601	990.71	0.38	14.590	949.92	0.37	15.820	906.87	0.36
12.787	1030.43	0.39	13.620	990.76	0.38	14.610	950.04	0.37	15.840	906.95	0.36
12.807	1030.49	0.39	13.640	990.87	0.38	14.629	950.14	0.37	15.860	907.11	0.36
12.827	1030.55	0.39	13.660	990.97	0.38	14.649	950.27	0.37	15.880	907.27	0.36
12.847	1030.66	0.39	13.680	991.05	0.38	14.669	950.41	0.37	15.900	907.43	0.36
12.867	1030.76	0.39	13.700	991.16	0.38	14.689	950.54	0.37	15.920	907.59	0.36
12.887	1030.82	0.39	13.720	991.31	0.38	14.709	950.64	0.37	15.940	907.75	0.36
12.907	1030.89	0.39	13.739	991.39	0.38	14.729	950.71	0.37	15.960	907.91	0.36
12.927	1030.95	0.39	13.759	991.47	0.38	14.748	950.83	0.37	15.979	908.00	0.36
12.947	1031.02	0.39	13.779	991.59	0.38	14.768	950.96	0.37	15.999	908.14	0.36
12.966	1031.11	0.39	13.799	991.67	0.38	14.788	951.08	0.37	16.019	908.30	0.36
12.986	1031.24	0.39	13.819	991.73	0.38	14.808	951.23	0.37	16.039	908.46	0.36
13.006	1031.30	0.39	13.838	991.86	0.38	14.828	951.34	0.37	16.059	908.62	0.36
13.026	1031.37	0.39	13.858	991.96	0.38	14.847	951.43	0.37	16.079	908.72	0.36
13.046	1031.43	0.39	13.878	992.02	0.38	14.867	951.61	0.37	16.099	908.85	0.36
13.066	1031.49	0.39	13.898	992.10	0.38	14.887	951.71	0.37	16.119	909.01	0.36
13.086	1031.57	0.39	13.918	992.26	0.38	14.907	951.81	0.37	16.139	909.17	0.36
13.106	1031.69	0.39	13.937	992.31	0.38	14.927	951.94	0.37	16.159	909.33	0.36
13.126	1031.77	0.39	13.957	992.44	0.38	14.947	952.07	0.37	16.179	909.48	0.36
13.146	1031.83	0.39	13.977	992.53	0.38	14.966	952.17	0.37	16.198	909.64	0.36
13.166	1031.89	0.39	13.997	992.61	0.38	14.986	952.27	0.37	16.218	909.79	0.36
13.186	1031.95	0.39	14.017	992.72	0.38	15.006	952.37	0.37	16.238	909.93	0.36
13.206	1032.07	0.39	14.037	992.80	0.38	15.026	952.49	0.37	16.258	910.07	0.36
13.225	1032.16	0.39	14.056	992.87	0.38	15.046	952.62	0.37	16.278	910.15	0.36
13.245	1032.22	0.39	14.076	992.96	0.38	15.065	952.71	0.37	16.298	910.29	0.36
13.265	1032.29	0.39	14.096	993.09	0.38	15.085	952.81	0.37	16.318	910.44	0.36
13.285	1032.35	0.39	14.116	993.21	0.38	15.105	952.91	0.37	16.338	910.59	0.36
13.305	1032.41	0.39	14.136	993.27	0.38	15.125	953.02	0.37	16.358	910.74	0.36
13.325	1032.50	0.39	14.155	993.36	0.38	15.145	953.16	0.37	16.378	910.90	0.36
13.345	1032.62	0.39	14.175	993.48	0.38	15.165	953.33	0.37	16.397	911.05	0.36
13.365	1032.69	0.39	14.195	993.53	0.38	15.184	953.43	0.37	16.417	911.16	0.36

13.385	1032.75	0.39	14.215	993.63	0.38	15.204	953.52	0.37	16.437	911.32	0.36
13.405	1032.85	0.39	14.235	993.74	0.38	15.224	953.63	0.37	16.457	911.48	0.36
13.425	1032.96	0.39	14.254	993.80	0.38	15.244	953.75	0.37	16.477	911.57	0.36
13.445	1033.03	0.39	14.274	993.92	0.38	15.264	953.86	0.37	16.497	911.72	0.36
13.465	1033.09	0.39	14.294	994.01	0.38	15.283	953.95	0.37	16.517	911.88	0.36
13.485	1033.15	0.39	14.314	994.11	0.38	15.303	954.05	0.37	16.537	912.02	0.36
13.504	1033.22	0.39	14.334	994.21	0.38	15.323	954.17	0.37	16.557	912.17	0.36
13.524	1033.28	0.39	14.354	994.27	0.38	15.343	954.28	0.37	16.577	912.31	0.36
13.544	1033.34	0.39	14.373	994.39	0.38	15.363	954.41	0.37	16.596	912.45	0.36
13.564	1033.46	0.39	14.393	994.48	0.38	15.383	954.54	0.37	16.616	912.59	0.36
13.584	1033.55	0.39	14.413	994.55	0.38	15.402	954.64	0.37	16.636	912.73	0.36
13.604	1033.62	0.39	14.433	994.67	0.38	15.422	954.73	0.37	16.656	912.87	0.36
13.624	1033.68	0.39	14.453	994.74	0.38	15.442	954.85	0.37	16.676	913.01	0.36
13.644	1033.74	0.39	14.472	994.82	0.38	15.462	954.96	0.37	16.696	913.15	0.36
13.664	1033.84	0.39	14.492	994.94	0.38	15.482	955.07	0.37	16.716	913.29	0.36
13.684	1033.94	0.39	14.512	995.07	0.38	15.501	955.19	0.37	16.736	913.43	0.36
13.704	1034.01	0.39	14.532	995.14	0.38	15.521	955.28	0.37	16.756	913.57	0.36
13.724	1034.07	0.39	14.552	995.19	0.38	15.541	955.41	0.37	16.776	913.71	0.36
13.744	1034.13	0.39	14.571	995.32	0.38	15.561	955.54	0.37	16.796	913.85	0.36
13.763	1034.21	0.39	14.591	995.39	0.38	15.581	955.64	0.37	16.815	913.98	0.36
13.783	1034.33	0.39	14.611	995.47	0.38	15.601	955.73	0.37	16.835	914.12	0.36
13.803	1034.40	0.39	14.631	995.59	0.38	15.620	955.88	0.37	16.855	914.27	0.36
13.823	1034.46	0.39	14.651	995.67	0.38	15.640	956.00	0.37	16.875	914.41	0.36
13.843	1034.52	0.39	14.670	995.77	0.38	15.660	956.08	0.37	16.895	914.55	0.36
13.863	1034.58	0.39	14.690	995.87	0.38	15.680	956.18	0.37	16.915	914.69	0.36
13.883	1034.70	0.39	14.710	995.99	0.38	15.700	956.33	0.37	16.935	914.83	0.36
13.903	1034.78	0.39	14.730	996.05	0.38	15.719	956.44	0.37	16.955	914.96	0.36
13.923	1034.85	0.39	14.750	996.13	0.38	15.739	956.51	0.37	16.975	915.10	0.36
13.943	1034.91	0.39	14.770	996.24	0.38	15.759	956.63	0.37	16.995	915.23	0.36
13.963	1034.97	0.39	14.789	996.31	0.38	15.779	956.76	0.37	17.014	915.36	0.36
13.983	1035.03	0.39	14.809	996.38	0.38	15.799	956.88	0.37	17.034	915.49	0.36
14.003	1035.12	0.39	14.829	996.47	0.38	15.819	957.03	0.37	17.054	915.63	0.36
14.022	1035.24	0.39	14.849	996.60	0.38	15.838	957.13	0.37	17.074	915.76	0.36
14.042	1035.30	0.39	14.869	996.68	0.38	15.858	957.19	0.37	17.094	915.89	0.36
14.062	1035.36	0.39	14.888	996.79	0.38	15.878	957.33	0.37	17.114	916.02	0.36
14.082	1035.45	0.39	14.908	996.88	0.38	15.898	957.43	0.37	17.134	916.15	0.36
14.102	1035.57	0.39	14.928	996.94	0.38	15.918	957.54	0.37	17.154	916.29	0.36
14.122	1035.63	0.39	14.948	997.03	0.38	15.937	957.66	0.37	17.174	916.42	0.36
14.142	1035.69	0.39	14.968	997.16	0.38	15.957	957.80	0.37	17.194	916.55	0.36
14.162	1035.75	0.39	14.987	997.23	0.38	15.977	957.92	0.37	17.213	916.68	0.36
14.182	1035.82	0.39	15.007	997.29	0.38	15.997	958.01	0.37	17.233	916.81	0.36
14.202	1035.94	0.39	15.027	997.41	0.38	16.017	958.06	0.37	17.253	916.94	0.36
14.222	1036.01	0.39	15.047	997.50	0.38	16.036	958.18	0.37	17.273	917.09	0.36
14.242	1036.07	0.39	15.067	997.56	0.38	16.056	958.31	0.37	17.293	917.22	0.36
14.262	1036.13	0.39	15.087	997.63	0.38	16.076	958.42	0.37	17.313	917.39	0.36

14.282	1036.19	0.39	15.106	997.78	0.38	16.096	958.52	0.37	17.333	917.53	0.36
14.301	1036.25	0.39	15.126	997.86	0.38	16.116	958.63	0.37	17.353	917.60	0.36
14.321	1036.32	0.39	15.146	997.98	0.38	16.136	958.75	0.37	17.373	917.79	0.36
14.341	1036.44	0.39	15.166	998.08	0.38	16.155	958.88	0.37	17.393	917.93	0.36
14.361	1036.51	0.39	15.186	998.13	0.38	16.175	958.99	0.37	17.413	918.05	0.36
14.381	1036.59	0.39	15.205	998.24	0.38	16.195	959.08	0.37	17.432	918.18	0.36
14.401	1036.70	0.39	15.225	998.33	0.38	16.215	959.15	0.37	17.452	918.31	0.36
14.421	1036.76	0.39	15.245	998.40	0.38	16.235	959.31	0.37	17.472	918.44	0.36
14.441	1036.82	0.39	15.265	998.46	0.38	16.254	959.42	0.37	17.492	918.57	0.36
14.461	1036.88	0.39	15.285	998.58	0.38	16.274	959.50	0.37	17.512	918.69	0.36
14.481	1036.94	0.39	15.304	998.66	0.38	16.294	959.60	0.37	17.532	918.82	0.36
14.501	1037.00	0.39	15.324	998.76	0.38	16.314	959.74	0.37	17.552	918.95	0.36
14.521	1037.10	0.39	15.344	998.87	0.38	16.334	959.81	0.37	17.572	919.08	0.36
14.541	1037.20	0.39	15.364	998.94	0.38	16.354	959.93	0.37	17.592	919.20	0.36
14.560	1037.26	0.39	15.384	999.00	0.38	16.373	960.02	0.37	17.612	919.33	0.36
14.580	1037.32	0.39	15.404	999.10	0.38	16.393	960.16	0.37	17.631	919.45	0.36
14.600	1037.40	0.39	15.423	999.22	0.38	16.413	960.28	0.37	17.651	919.63	0.36
14.620	1037.51	0.39	15.443	999.29	0.38	16.433	960.39	0.37	17.671	919.78	0.36
14.640	1037.57	0.39	15.463	999.35	0.38	16.453	960.48	0.37	17.691	919.90	0.36
14.660	1037.63	0.39	15.483	999.52	0.38	16.472	960.63	0.37	17.711	920.02	0.36
14.680	1037.70	0.39	15.503	999.58	0.38	16.492	960.75	0.37	17.731	920.14	0.36
14.700	1037.81	0.39	15.522	999.65	0.38	16.512	960.82	0.37	17.751	920.26	0.36
14.720	1037.88	0.39	15.542	999.73	0.38	16.532	960.88	0.37	17.771	920.38	0.36
14.740	1037.94	0.39	15.562	999.83	0.38	16.552	960.99	0.37	17.791	920.50	0.36
14.760	1038.00	0.39	15.582	999.93	0.38	16.572	961.17	0.37	17.811	920.62	0.36
14.780	1038.11	0.39	15.602	1000.00	0.38	16.591	961.24	0.37	17.831	920.76	0.36
14.800	1038.19	0.39	15.621	1000.06	0.38	16.611	961.34	0.37	17.850	920.94	0.36
14.819	1038.25	0.39	15.641	1000.17	0.38	16.631	961.49	0.37	17.870	921.07	0.36
14.839	1038.30	0.39	15.661	1000.28	0.38	16.651	961.61	0.37	17.890	921.19	0.36
14.859	1038.39	0.39	15.681	1000.36	0.38	16.671	961.66	0.37	17.910	921.31	0.36
14.879	1038.50	0.39	15.701	1000.41	0.38	16.690	961.76	0.37	17.930	921.42	0.36
14.899	1038.55	0.39	15.721	1000.56	0.38	16.710	961.91	0.37	17.950	921.54	0.36
14.919	1038.61	0.39	15.740	1000.64	0.38	16.730	962.03	0.37	17.970	921.67	0.36
14.939	1038.66	0.39	15.760	1000.71	0.38	16.750	962.13	0.37	17.990	921.79	0.36
14.959	1038.72	0.39	15.780	1000.78	0.38	16.770	962.24	0.37	18.010	921.91	0.36
14.979	1038.81	0.39	15.800	1000.88	0.38	16.790	962.35	0.37	18.030	922.09	0.36
14.999	1038.91	0.39	15.820	1000.99	0.38	16.809	962.42	0.37	18.049	922.23	0.36
15.019	1038.97	0.39	15.839	1001.07	0.38	16.829	962.53	0.37	18.069	922.35	0.36
15.039	1039.02	0.39	15.859	1001.13	0.38	16.849	962.63	0.37	18.089	922.47	0.36
15.059	1039.09	0.39	15.879	1001.20	0.38	16.869	962.72	0.37	18.109	922.58	0.36
15.079	1039.20	0.39	15.899	1001.32	0.38	16.889	962.85	0.37	18.129	922.71	0.36
15.098	1039.27	0.39	15.919	1001.43	0.38	16.908	962.97	0.37	18.149	922.88	0.36
15.118	1039.33	0.39	15.938	1001.50	0.38	16.928	963.09	0.37	18.169	923.00	0.36
15.138	1039.38	0.39	15.958	1001.57	0.38	16.948	963.18	0.37	18.189	923.11	0.36
15.158	1039.49	0.39	15.978	1001.65	0.38	16.968	963.28	0.37	18.209	923.23	0.36

15.178	1039.58	0.39	15.998	1001.76	0.38	16.988	963.39	0.37	18.229	923.34	0.36
15.198	1039.63	0.39	16.018	1001.85	0.38	17.008	963.51	0.37	18.248	923.45	0.36
15.218	1039.69	0.39	16.038	1001.92	0.38	17.027	963.60	0.37	18.268	923.57	0.36
15.238	1039.77	0.39	16.057	1002.01	0.38	17.047	963.70	0.37	18.288	923.74	0.36
15.258	1039.88	0.39	16.077	1002.14	0.38	17.067	963.80	0.37	18.308	923.88	0.36
15.278	1039.94	0.39	16.097	1002.21	0.38	17.087	963.91	0.37	18.328	924.00	0.36
15.298	1039.99	0.39	16.117	1002.27	0.38	17.107	964.03	0.37	18.348	924.12	0.36
15.318	1040.04	0.39	16.137	1002.38	0.38	17.126	964.13	0.37	18.368	924.24	0.36
15.338	1040.10	0.39	16.156	1002.49	0.38	17.146	964.24	0.37	18.388	924.37	0.36
15.357	1040.17	0.39	16.176	1002.56	0.38	17.166	964.34	0.37	18.408	924.50	0.36
15.377	1040.28	0.39	16.196	1002.63	0.38	17.186	964.45	0.37	18.428	924.62	0.36
15.397	1040.34	0.39	16.216	1002.76	0.38	17.206	964.56	0.37	18.448	924.74	0.36
15.417	1040.40	0.39	16.236	1002.82	0.38	17.226	964.67	0.37	18.467	924.87	0.36
15.437	1040.51	0.39	16.255	1002.87	0.38	17.245	964.76	0.37	18.487	924.99	0.36
15.457	1040.58	0.39	16.275	1002.96	0.38	17.265	964.85	0.37	18.507	925.10	0.36
15.477	1040.63	0.39	16.295	1003.10	0.38	17.285	964.96	0.37	18.527	925.22	0.36
15.497	1040.70	0.39	16.315	1003.16	0.38	17.305	965.07	0.37	18.547	925.35	0.36
15.517	1040.75	0.39	16.335	1003.24	0.38	17.325	965.18	0.37	18.567	925.47	0.36
15.537	1040.81	0.39	16.355	1003.36	0.38	17.344	965.27	0.37	18.587	925.58	0.36
15.557	1040.88	0.39	16.374	1003.41	0.38	17.364	965.36	0.37	18.607	925.70	0.36
15.577	1040.95	0.39	16.394	1003.48	0.38	17.384	965.46	0.37	18.627	925.81	0.36
15.597	1041.07	0.39	16.414	1003.58	0.38	17.404	965.58	0.37	18.647	925.93	0.36
15.616	1041.15	0.39	16.434	1003.69	0.38	17.424	965.69	0.37	18.666	926.09	0.36
15.636	1041.22	0.39	16.454	1003.74	0.38	17.444	965.78	0.37	18.686	926.23	0.36
15.656	1041.28	0.39	16.473	1003.84	0.38	17.463	965.87	0.37	18.706	926.35	0.36
15.676	1041.35	0.39	16.493	1003.94	0.38	17.483	965.96	0.37	18.726	926.46	0.36
15.696	1041.41	0.39	16.513	1003.99	0.38	17.503	966.06	0.37	18.746	926.57	0.36
15.716	1041.48	0.39	16.533	1004.09	0.38	17.523	966.17	0.37	18.766	926.68	0.36
15.736	1041.54	0.39	16.553	1004.19	0.38	17.543	966.27	0.37	18.786	926.79	0.36
15.756	1041.61	0.39	16.572	1004.27	0.38	17.562	966.36	0.37	18.806	926.94	0.36
15.776	1041.68	0.39	16.592	1004.36	0.38	17.582	966.44	0.37	18.826	927.10	0.36
15.796	1041.75	0.39	16.612	1004.44	0.38	17.602	966.55	0.37	18.846	927.21	0.36
15.816	1041.82	0.39	16.632	1004.50	0.38	17.622	966.70	0.37	18.865	927.28	0.36
15.836	1041.89	0.39	16.652	1004.56	0.38	17.642	966.80	0.37	18.885	927.38	0.36
15.856	1041.96	0.39	16.672	1004.66	0.38	17.662	966.89	0.37	18.905	927.52	0.36
15.876	1042.04	0.39	16.691	1004.76	0.38	17.681	966.97	0.37	18.925	927.65	0.36
15.895	1042.11	0.39	16.711	1004.83	0.38	17.701	967.05	0.37	18.945	927.78	0.36
15.915	1042.19	0.39	16.731	1004.89	0.38	17.721	967.15	0.37	18.965	927.91	0.36
15.935	1042.27	0.39	16.751	1005.00	0.38	17.741	967.26	0.37	18.985	928.03	0.36
15.955	1042.35	0.39	16.771	1005.09	0.38	17.761	967.41	0.37	19.005	928.14	0.36
15.975	1042.42	0.39	16.790	1005.14	0.38	17.780	967.52	0.37	19.025	928.26	0.36
15.995	1042.50	0.39	16.810	1005.26	0.38	17.800	967.60	0.37	19.045	928.38	0.36
16.015	1042.58	0.39	16.830	1005.36	0.38	17.820	967.70	0.37	19.065	928.50	0.36
16.035	1042.65	0.39	16.850	1005.42	0.38	17.840	967.80	0.37	19.084	928.62	0.36
16.055	1042.72	0.39	16.870	1005.47	0.38	17.860	967.85	0.37	19.104	928.74	0.36

16.075	1042.80	0.39	16.889	1005.59	0.38	17.880	967.96	0.37	19.124	928.86	0.36
16.095	1042.87	0.39	16.909	1005.67	0.38	17.899	968.11	0.37	19.144	928.99	0.36
16.115	1042.95	0.39	16.929	1005.73	0.38	17.919	968.17	0.37	19.164	929.12	0.36
16.135	1043.02	0.39	16.949	1005.79	0.38	17.939	968.23	0.37	19.184	929.24	0.36
16.154	1043.09	0.39	16.969	1005.94	0.38	17.959	968.33	0.37	19.204	929.37	0.36
16.174	1043.16	0.39	16.989	1006.02	0.38	17.979	968.47	0.37	19.224	929.49	0.36
16.194	1043.23	0.39	17.008	1006.10	0.38	17.998	968.57	0.37	19.244	929.61	0.36
16.214	1043.30	0.39	17.028	1006.17	0.38	18.018	968.65	0.37	19.264	929.72	0.36
16.234	1043.37	0.39	17.048	1006.24	0.38	18.038	968.74	0.37	19.283	929.84	0.36
16.254	1043.44	0.39	17.068	1006.31	0.38	18.058	968.88	0.37	19.303	929.96	0.36
16.274	1043.51	0.39	17.088	1006.38	0.38	18.078	968.98	0.37	19.323	930.09	0.36
16.294	1043.57	0.39	17.107	1006.44	0.38	18.098	969.05	0.37	19.343	930.21	0.36
16.314	1043.64	0.39	17.127	1006.52	0.38	18.117	969.13	0.37	19.363	930.33	0.36
16.334	1043.71	0.39	17.147	1006.68	0.38	18.137	969.26	0.37	19.383	930.44	0.36
16.354	1043.78	0.39	17.167	1006.75	0.38	18.157	969.36	0.37	19.403	930.50	0.36
16.374	1043.85	0.39	17.187	1006.82	0.38	18.177	969.46	0.37	19.423	930.67	0.36
16.394	1043.92	0.39	17.206	1006.89	0.38	18.197	969.56	0.37	19.443	930.81	0.36
16.413	1044.01	0.39	17.226	1006.96	0.38	18.216	969.67	0.37	19.463	930.92	0.36
16.433	1044.14	0.39	17.246	1007.02	0.38	18.236	969.79	0.37	19.483	931.03	0.36
16.453	1044.20	0.39	17.266	1007.10	0.38	18.256	969.91	0.37	19.502	931.14	0.36
16.473	1044.20	0.39	17.286	1007.18	0.38	18.276	969.99	0.37	19.522	931.25	0.36
16.493	1044.30	0.39	17.306	1007.35	0.38	18.296	970.08	0.37	19.542	931.36	0.36
16.513	1044.40	0.39	17.325	1007.43	0.38	18.316	970.19	0.37	19.562	931.47	0.36
16.533	1044.47	0.39	17.345	1007.50	0.38	18.335	970.30	0.37	19.582	931.58	0.36
16.553	1044.53	0.39	17.365	1007.59	0.38	18.355	970.41	0.37	19.602	931.69	0.36
16.573	1044.60	0.39	17.385	1007.65	0.38	18.375	970.50	0.37	19.622	931.80	0.36
16.593	1044.67	0.39	17.405	1007.73	0.38	18.395	970.58	0.37	19.642	931.91	0.36
16.613	1044.74	0.39	17.424	1007.81	0.38	18.415	970.64	0.37	19.662	932.02	0.36
16.633	1044.80	0.39	17.444	1007.88	0.38	18.434	970.78	0.37	19.682	932.14	0.36
16.653	1044.87	0.39	17.464	1007.96	0.38	18.454	970.86	0.37	19.701	932.27	0.36
16.673	1044.93	0.39	17.484	1008.05	0.38	18.474	970.93	0.37	19.721	932.40	0.36
16.692	1045.00	0.39	17.504	1008.13	0.38	18.494	971.04	0.37	19.741	932.52	0.36
16.712	1045.06	0.39	17.523	1008.21	0.38	18.514	971.12	0.37	19.761	932.65	0.36
16.732	1045.15	0.39	17.543	1008.29	0.38	18.534	971.25	0.37	19.781	932.74	0.36
16.752	1045.27	0.39	17.563	1008.37	0.38	18.553	971.37	0.37	19.801	932.83	0.36
16.772	1045.34	0.39	17.583	1008.45	0.38	18.573	971.47	0.37	19.821	932.97	0.36
16.792	1045.40	0.39	17.603	1008.52	0.38	18.593	971.53	0.37	19.841	933.11	0.36
16.812	1045.46	0.39	17.623	1008.62	0.38	18.613	971.65	0.37	19.861	933.18	0.36
16.832	1045.52	0.39	17.642	1008.70	0.38	18.633	971.77	0.37	19.881	933.30	0.36
16.852	1045.58	0.39	17.662	1008.77	0.38	18.652	971.87	0.37	19.900	933.44	0.36
16.872	1045.65	0.39	17.682	1008.86	0.38	18.672	971.93	0.37	19.920	933.55	0.36
16.892	1045.71	0.39	17.702	1008.94	0.38	18.692	972.05	0.37	19.940	933.63	0.36
16.912	1045.79	0.39	17.722	1009.03	0.38	18.712	972.17	0.37	19.960	933.77	0.36
16.932	1045.92	0.39	17.741	1009.06	0.38	18.732	972.27	0.37	19.980	933.85	0.36
16.951	1045.91	0.39	17.761	1009.23	0.38	18.752	972.33	0.37	20.000	933.99	0.36

16.971	1046.00	0.39	17.781	1009.25	0.38	18.771	972.43	0.37			
16.991	1046.10	0.39	17.801	1009.37	0.38	18.791	972.57	0.37			
17.011	1046.16	0.39	17.821	1009.39	0.38	18.811	972.68	0.37			
17.031	1046.22	0.39	17.840	1009.49	0.38	18.831	972.74	0.37			
17.051	1046.28	0.39	17.860	1009.59	0.38	18.851	972.80	0.37			
17.071	1046.34	0.39	17.880	1009.67	0.38	18.870	972.89	0.37			
17.091	1046.40	0.39	17.900	1009.76	0.38	18.890	973.02	0.37			
17.111	1046.46	0.39	17.920	1009.83	0.38	18.910	973.12	0.37			
17.131	1046.56	0.39	17.940	1009.94	0.38	18.930	973.21	0.37			
17.151	1046.66	0.39	17.959	1010.03	0.38	18.950	973.31	0.37			
17.171	1046.72	0.39	17.979	1010.11	0.38	18.969	973.44	0.37			
17.191	1046.78	0.39	17.999	1010.20	0.38	18.989	973.48	0.37			
17.210	1046.84	0.39	18.019	1010.24	0.38	19.009	973.58	0.37			
17.230	1046.90	0.39	18.039	1010.34	0.38	19.029	973.69	0.37			
17.250	1046.96	0.39	18.058	1010.43	0.38	19.049	973.78	0.37			
17.270	1047.02	0.39	18.078	1010.53	0.38	19.069	973.88	0.37			
17.290	1047.09	0.39	18.098	1010.60	0.38	19.088	973.99	0.37			
17.310	1047.21	0.39	18.118	1010.70	0.38	19.108	974.09	0.37			
17.330	1047.27	0.39	18.138	1010.72	0.38	19.128	974.16	0.37			
17.350	1047.33	0.39	18.157	1010.83	0.38	19.148	974.28	0.37			
17.370	1047.39	0.39	18.177	1010.92	0.38	19.168	974.39	0.37			
17.390	1047.44	0.39	18.197	1011.02	0.38	19.187	974.48	0.37			
17.410	1047.53	0.39	18.217	1011.09	0.38	19.207	974.55	0.37			
17.430	1047.64	0.39	18.237	1011.19	0.38	19.227	974.66	0.38			
17.450	1047.69	0.39	18.257	1011.27	0.38	19.247	974.77	0.38			
17.470	1047.75	0.39	18.276	1011.29	0.38	19.267	974.85	0.38			
17.489	1047.81	0.39	18.296	1011.40	0.38	19.287	974.95	0.38			
17.509	1047.86	0.39	18.316	1011.50	0.38	19.306	975.05	0.38			
17.529	1047.97	0.39	18.336	1011.59	0.38	19.326	975.19	0.38			
17.549	1048.06	0.39	18.356	1011.64	0.38	19.346	975.28	0.38			
17.569	1048.11	0.39	18.375	1011.76	0.38	19.366	975.36	0.38			
17.589	1048.17	0.39	18.395	1011.79	0.38	19.386	975.42	0.38			
17.609	1048.23	0.39	18.415	1011.90	0.38	19.405	975.46	0.38			
17.629	1048.28	0.39	18.435	1011.98	0.38	19.425	975.57	0.38			
17.649	1048.34	0.39	18.455	1012.08	0.38	19.445	975.68	0.38			
17.669	1048.44	0.39	18.474	1012.17	0.38	19.465	975.77	0.38			
17.689	1048.53	0.39	18.494	1012.27	0.38	19.485	975.85	0.38			
17.709	1048.59	0.39	18.514	1012.33	0.38	19.505	975.93	0.38			
17.729	1048.64	0.39	18.534	1012.38	0.38	19.524	976.05	0.38			
17.748	1048.70	0.39	18.554	1012.49	0.38	19.544	976.17	0.38			
17.768	1048.81	0.39	18.574	1012.54	0.38	19.564	976.28	0.38			
17.788	1048.88	0.39	18.593	1012.62	0.38	19.584	976.33	0.38			
17.808	1048.93	0.39	18.613	1012.71	0.38	19.604	976.41	0.38			
17.828	1048.99	0.39	18.633	1012.80	0.38	19.623	976.53	0.38			
17.848	1049.07	0.39	18.653	1012.90	0.38	19.643	976.61	0.38			

17.868	1049.17	0.39	18.673	1012.97	0.38	19.663	976.70	0.38			
17.888	1049.23	0.39	18.692	1013.07	0.38	19.683	976.80	0.38			
17.908	1049.28	0.39	18.712	1013.15	0.38	19.703	976.91	0.38			
17.928	1049.33	0.39	18.732	1013.22	0.38	19.723	976.99	0.38			
17.948	1049.38	0.39	18.752	1013.32	0.38	19.742	977.08	0.38			
17.968	1049.46	0.39	18.772	1013.38	0.38	19.762	977.18	0.38			
17.988	1049.56	0.39	18.791	1013.46	0.38	19.782	977.27	0.38			
18.007	1049.62	0.39	18.811	1013.55	0.38	19.802	977.35	0.38			
18.027	1049.68	0.39	18.831	1013.66	0.38	19.822	977.41	0.38			
18.047	1049.73	0.39	18.851	1013.76	0.38	19.841	977.56	0.38			
18.067	1049.81	0.39	18.871	1013.80	0.38	19.861	977.68	0.38			
18.087	1049.91	0.39	18.891	1013.86	0.38	19.881	977.72	0.38			
18.107	1049.96	0.39	18.910	1013.99	0.38	19.901	977.79	0.38			
18.127	1050.01	0.39	18.930	1014.06	0.38	19.921	977.91	0.38			
18.147	1050.06	0.39	18.950	1014.13	0.38	19.941	977.98	0.38			
18.167	1050.15	0.39	18.970	1014.21	0.38	19.960	978.10	0.38			
18.187	1050.25	0.39	18.990	1014.27	0.38	19.980	978.19	0.38			
18.207	1050.30	0.39	19.009	1014.33	0.38	20.000	978.28	0.38			
18.227	1050.35	0.39	19.029	1014.41	0.38						
18.247	1050.40	0.39	19.049	1014.48	0.38						
18.267	1050.49	0.39	19.069	1014.56	0.38						
18.286	1050.57	0.39	19.089	1014.70	0.38						
18.306	1050.62	0.39	19.108	1014.76	0.38						
18.326	1050.69	0.39	19.128	1014.81	0.38						
18.346	1050.79	0.39	19.148	1014.88	0.38						
18.366	1050.84	0.39	19.168	1014.97	0.38						
18.386	1050.89	0.39	19.188	1015.07	0.38						
18.406	1050.94	0.39	19.208	1015.16	0.38						
18.426	1051.00	0.39	19.227	1015.22	0.38						
18.446	1051.10	0.39	19.247	1015.28	0.38						
18.466	1051.16	0.39	19.267	1015.39	0.39						
18.486	1051.21	0.39	19.287	1015.48	0.39						
18.506	1051.30	0.39	19.307	1015.54	0.39						
18.526	1051.38	0.39	19.326	1015.58	0.39						
18.545	1051.43	0.39	19.346	1015.68	0.39						
18.565	1051.49	0.39	19.366	1015.77	0.39						
18.585	1051.58	0.39	19.386	1015.84	0.39						
18.605	1051.64	0.39	19.406	1015.93	0.39						
18.625	1051.69	0.39	19.425	1016.01	0.39						
18.645	1051.77	0.39	19.445	1016.07	0.39						
18.665	1051.86	0.39	19.465	1016.12	0.39						
18.685	1051.90	0.39	19.485	1016.18	0.39						
18.705	1051.95	0.39	19.505	1016.30	0.39						
18.725	1051.99	0.39	19.525	1016.37	0.39						
18.745	1052.05	0.39	19.544	1016.43	0.39						

18.765	1052.15	0.39	19.564	1016.53	0.39						
18.785	1052.21	0.39	19.584	1016.61	0.39						
18.805	1052.26	0.39	19.604	1016.67	0.39						
18.824	1052.34	0.39	19.624	1016.73	0.39						
18.844	1052.43	0.39	19.643	1016.80	0.39						
18.864	1052.47	0.39	19.663	1016.87	0.39						
18.884	1052.52	0.39	19.683	1016.94	0.39						
18.904	1052.61	0.39	19.703	1017.04	0.39						
18.924	1052.70	0.39	19.723	1017.17	0.39						
18.944	1052.79	0.39	19.742	1017.23	0.39						
18.964	1052.86	0.39	19.762	1017.29	0.39						
18.984	1052.90	0.39	19.782	1017.37	0.39						
19.004	1052.94	0.39	19.802	1017.43	0.39						
19.024	1052.99	0.39	19.822	1017.49	0.39						
19.044	1053.06	0.39	19.842	1017.57	0.39						
19.064	1053.15	0.39	19.861	1017.66	0.39						
19.083	1053.20	0.39	19.881	1017.79	0.39						
19.103	1053.24	0.39	19.901	1017.87	0.39						
19.123	1053.32	0.39	19.921	1017.92	0.39						
19.143	1053.41	0.39	19.941	1017.97	0.39						
19.163	1053.45	0.39	19.960	1018.05	0.39						
19.183	1053.50	0.39	19.980	1018.13	0.39						
19.203	1053.59	0.39	20.000	1018.19	0.39						
19.223	1053.66	0.39									
19.243	1053.70	0.39									
19.263	1053.76	0.39									
19.283	1053.84	0.39									
19.303	1053.92	0.39									
19.323	1054.01	0.39									
19.342	1054.07	0.39									
19.362	1054.12	0.39									
19.382	1054.18	0.39									
19.402	1054.26	0.39									
19.422	1054.32	0.39									
19.442	1054.36	0.39									
19.462	1054.43	0.39									
19.482	1054.51	0.39									
19.502	1054.57	0.39									
19.522	1054.61	0.39									
19.542	1054.67	0.39									
19.562	1054.75	0.39									
19.582	1054.83	0.39									
19.602	1054.91	0.39									
19.621	1054.97	0.39									
19.641	1055.01	0.39									

19.661	1055.07	0.39									
19.681	1055.15	0.39									
19.701	1055.21	0.40									
19.721	1055.26	0.40									
19.741	1055.30	0.40									
19.761	1055.35	0.40									
19.781	1055.44	0.40									
19.801	1055.53	0.40									
19.821	1055.58	0.40									
19.841	1055.62	0.40									
19.861	1055.67	0.40									
19.880	1055.74	0.40									
19.900	1055.84	0.40									
19.920	1055.89	0.40									
19.940	1055.94	0.40									
19.960	1056.03	0.40									
19.980	1056.11	0.40									
20.000	1056.15	0.40									

Combined standard uncertainties:

$u(T)$= 0.006 K; $u(p)$= 0.0020 MPa for p < 6 MPa; $u(p)$= 0.024 MPa for 6 MPa ≤ p ≤ 70 MPa

$u(x_{\mathrm{CO_2}})$= 0.00025; $u(x_{\mathrm{NO}})$= 0.000014; $u(x_{\mathrm{SO_2}})$= 0.0000023; $u(x_{\mathrm{CO}})$= 0.0000043

Table S1 (continued). $p\rho T$ experimental data for the CO_2 + O_2 + SO_2 + CO (Mix 1) and CO_2 + NO + SO_2 + CO (Mix 2) mixtures. $u(\rho)$: combined standard uncertainty.

**Mix 1: x_{CO_2} = 0.96734; x_{O_2} = 0.030038; x_{SO_2} = 0.0009035; x_{CO} = 0.0017032**											
***T*= 303.09±0.01 K**			***T*= 313.14±0.01 K**			***T*= 333.15±0.01 K**			***T*= 353.15±0.02 K**		
p (MPa)	ρ (kg.m^{-3})	$u(\rho)$ (kg.m^{-3})	p (MPa)	ρ (kg.m^{-3})	$u(\rho)$ (kg.m^{-3})	p (MPa)	ρ (kg.m^{-3})	$u(\rho)$ (kg.m^{-3})	p (MPa)	ρ (kg.m^{-3})	$u(\rho)$ (kg.m^{-3})
0.113	1.64	0.21	0.114	1.60	0.21	0.133	1.77	0.21	0.131	1.69	0.21
0.133	2.01	0.21	0.134	1.97	0.21	0.153	2.10	0.21	0.151	1.98	0.21
0.153	2.40	0.21	0.154	2.32	0.21	0.173	2.44	0.21	0.171	2.23	0.21
0.173	2.73	0.21	0.174	2.62	0.21	0.193	2.77	0.21	0.190	2.49	0.21
0.193	3.05	0.21	0.194	3.00	0.21	0.213	3.11	0.21	0.210	2.82	0.21
0.213	3.48	0.22	0.214	3.37	0.22	0.232	3.46	0.22	0.230	3.11	0.21
0.233	3.77	0.22	0.234	3.69	0.22	0.252	3.76	0.22	0.250	3.43	0.21
0.253	4.12	0.22	0.254	4.04	0.22	0.272	4.04	0.22	0.270	3.76	0.21
0.272	4.53	0.22	0.274	4.40	0.22	0.292	4.39	0.22	0.290	4.06	0.21
0.292	4.91	0.22	0.293	4.72	0.22	0.312	4.74	0.22	0.310	4.34	0.21
0.312	5.28	0.22	0.313	5.04	0.22	0.332	5.09	0.22	0.330	4.63	0.21
0.332	5.61	0.22	0.333	5.41	0.22	0.352	5.37	0.22	0.350	4.92	0.22
0.352	5.95	0.22	0.353	5.77	0.22	0.372	5.62	0.22	0.370	5.20	0.21
0.372	6.28	0.22	0.373	6.13	0.22	0.392	5.95	0.22	0.390	5.51	0.22
0.392	6.65	0.22	0.393	6.45	0.22	0.412	6.32	0.22	0.410	5.85	0.22
0.412	7.03	0.22	0.413	6.78	0.22	0.432	6.60	0.22	0.430	6.18	0.22
0.432	7.38	0.22	0.433	7.11	0.22	0.452	6.97	0.22	0.450	6.49	0.22
0.452	7.75	0.22	0.453	7.45	0.22	0.472	7.31	0.22	0.470	6.77	0.22
0.472	8.08	0.22	0.473	7.85	0.22	0.492	7.61	0.22	0.490	7.05	0.22
0.491	8.40	0.22	0.493	8.22	0.22	0.512	7.91	0.22	0.509	7.36	0.22
0.511	8.78	0.22	0.512	8.54	0.22	0.532	8.25	0.22	0.529	7.68	0.22
0.531	9.15	0.22	0.532	8.89	0.22	0.552	8.57	0.22	0.549	8.01	0.22
0.551	9.49	0.22	0.552	9.22	0.22	0.571	8.90	0.22	0.569	8.35	0.22
0.571	9.89	0.22	0.572	9.58	0.22	0.591	9.23	0.22	0.589	8.60	0.22
0.591	10.24	0.22	0.592	9.95	0.22	0.611	9.56	0.22	0.609	8.89	0.22
0.611	10.58	0.22	0.612	10.31	0.22	0.631	9.89	0.22	0.629	9.22	0.22
0.631	10.97	0.22	0.632	10.63	0.22	0.651	10.24	0.22	0.649	9.56	0.22
0.651	11.36	0.22	0.652	10.96	0.22	0.671	10.51	0.22	0.669	9.90	0.22
0.671	11.74	0.22	0.672	11.35	0.22	0.691	10.82	0.22	0.689	10.17	0.22
0.691	12.09	0.22	0.692	11.71	0.22	0.711	11.18	0.22	0.709	10.43	0.22
0.710	12.44	0.22	0.712	12.05	0.22	0.731	11.55	0.22	0.729	10.79	0.22
0.730	12.84	0.22	0.731	12.41	0.22	0.751	11.90	0.22	0.749	11.09	0.22
0.750	13.21	0.22	0.751	12.80	0.22	0.771	12.22	0.22	0.769	11.40	0.22
0.770	13.58	0.22	0.771	13.17	0.22	0.791	12.54	0.22	0.789	11.72	0.22
0.790	13.97	0.22	0.791	13.50	0.22	0.811	12.86	0.22	0.809	12.01	0.22

0.810	14.36	0.22	0.811	13.83	0.22	0.831	13.17	0.22	0.829	12.31	0.22
0.830	14.72	0.22	0.831	14.18	0.22	0.851	13.52	0.22	0.848	12.64	0.22
0.850	15.06	0.22	0.851	14.55	0.22	0.871	13.82	0.22	0.868	12.97	0.22
0.870	15.41	0.22	0.871	14.88	0.22	0.890	14.18	0.22	0.888	13.29	0.22
0.890	15.84	0.22	0.891	15.29	0.22	0.910	14.53	0.22	0.908	13.63	0.22
0.910	16.22	0.22	0.911	15.64	0.22	0.930	14.84	0.22	0.928	13.97	0.22
0.929	16.57	0.22	0.930	15.99	0.22	0.950	15.19	0.22	0.948	14.25	0.22
0.949	16.94	0.22	0.950	16.35	0.22	0.970	15.52	0.22	0.968	14.54	0.22
0.969	17.33	0.22	0.970	16.71	0.22	0.990	15.86	0.22	0.988	14.82	0.22
0.989	17.71	0.22	0.990	17.11	0.22	1.010	16.18	0.22	1.008	15.11	0.22
1.009	18.11	0.22	1.010	17.45	0.22	1.030	16.52	0.22	1.028	15.44	0.22
1.029	18.45	0.22	1.030	17.78	0.22	1.050	16.86	0.22	1.048	15.76	0.22
1.049	18.84	0.22	1.050	18.18	0.22	1.070	17.19	0.22	1.068	16.07	0.22
1.069	19.26	0.22	1.070	18.55	0.22	1.090	17.54	0.22	1.088	16.39	0.22
1.089	19.64	0.22	1.090	18.89	0.22	1.110	17.91	0.22	1.108	16.73	0.22
1.109	20.00	0.22	1.110	19.23	0.22	1.130	18.27	0.22	1.128	17.08	0.22
1.128	20.45	0.22	1.130	19.63	0.22	1.150	18.61	0.22	1.148	17.40	0.22
1.148	20.83	0.22	1.149	19.99	0.22	1.170	18.95	0.22	1.167	17.72	0.22
1.168	21.17	0.22	1.169	20.35	0.22	1.190	19.27	0.22	1.187	18.02	0.22
1.188	21.54	0.22	1.189	20.75	0.22	1.209	19.56	0.22	1.207	18.31	0.22
1.208	21.94	0.22	1.209	21.14	0.22	1.229	19.94	0.22	1.227	18.59	0.22
1.228	22.30	0.22	1.229	21.51	0.22	1.249	20.31	0.22	1.247	18.96	0.22
1.248	22.71	0.22	1.249	21.87	0.22	1.269	20.66	0.22	1.267	19.27	0.22
1.268	23.11	0.22	1.269	22.25	0.22	1.289	21.00	0.22	1.287	19.58	0.22
1.288	23.49	0.22	1.289	22.61	0.22	1.309	21.34	0.22	1.307	19.92	0.22
1.308	23.88	0.22	1.309	22.94	0.22	1.329	21.66	0.22	1.327	20.20	0.22
1.328	24.28	0.22	1.329	23.35	0.22	1.349	22.00	0.22	1.347	20.50	0.22
1.347	24.70	0.22	1.348	23.75	0.22	1.369	22.37	0.22	1.367	20.84	0.22
1.367	25.10	0.22	1.368	24.13	0.22	1.389	22.68	0.22	1.387	21.19	0.22
1.387	25.44	0.22	1.388	24.50	0.22	1.409	23.02	0.22	1.407	21.48	0.22
1.407	25.84	0.22	1.408	24.88	0.22	1.429	23.37	0.22	1.427	21.81	0.22
1.427	26.24	0.22	1.428	25.20	0.22	1.449	23.73	0.22	1.447	22.14	0.22
1.447	26.67	0.22	1.448	25.59	0.22	1.469	24.12	0.22	1.467	22.47	0.22
1.467	27.08	0.22	1.468	25.98	0.22	1.489	24.45	0.22	1.487	22.79	0.22
1.487	27.46	0.22	1.488	26.37	0.22	1.509	24.79	0.22	1.506	23.10	0.22
1.507	27.89	0.22	1.508	26.77	0.22	1.528	25.15	0.22	1.526	23.41	0.22
1.527	28.30	0.22	1.528	27.19	0.22	1.548	25.42	0.22	1.546	23.76	0.22
1.547	28.68	0.22	1.548	27.55	0.22	1.568	25.80	0.22	1.566	24.10	0.22
1.566	29.04	0.22	1.567	27.91	0.22	1.588	26.15	0.22	1.586	24.44	0.22
1.586	29.43	0.22	1.587	28.28	0.22	1.608	26.51	0.22	1.606	24.76	0.22
1.606	29.84	0.22	1.607	28.66	0.22	1.628	26.89	0.22	1.626	25.04	0.22
1.626	30.25	0.22	1.627	29.00	0.22	1.648	27.27	0.22	1.646	25.33	0.22
1.646	30.66	0.22	1.647	29.42	0.22	1.668	27.59	0.22	1.666	25.66	0.22
1.666	31.06	0.22	1.667	29.81	0.22	1.688	27.95	0.22	1.686	26.02	0.22
1.686	31.48	0.22	1.687	30.20	0.22	1.708	28.32	0.22	1.706	26.36	0.22

1.706	31.92	0.22	1.707	30.58	0.22	1.728	28.69	0.22	1.726	26.71	0.22
1.726	32.30	0.22	1.727	30.97	0.22	1.748	29.05	0.22	1.746	27.04	0.22
1.746	32.73	0.22	1.747	31.37	0.22	1.768	29.40	0.22	1.766	27.37	0.22
1.765	33.16	0.22	1.767	31.76	0.22	1.788	29.73	0.22	1.786	27.70	0.22
1.785	33.58	0.22	1.786	32.15	0.22	1.808	30.08	0.22	1.806	28.00	0.22
1.805	33.96	0.22	1.806	32.56	0.22	1.828	30.44	0.22	1.825	28.30	0.22
1.825	34.37	0.22	1.826	32.98	0.22	1.847	30.81	0.22	1.845	28.66	0.22
1.845	34.77	0.22	1.846	33.36	0.22	1.867	31.18	0.22	1.865	28.97	0.22
1.865	35.20	0.22	1.866	33.77	0.22	1.887	31.55	0.22	1.885	29.30	0.22
1.885	35.64	0.22	1.886	34.14	0.22	1.907	31.92	0.22	1.905	29.63	0.22
1.905	36.03	0.22	1.906	34.54	0.22	1.927	32.28	0.22	1.925	29.94	0.22
1.925	36.49	0.22	1.926	34.98	0.22	1.947	32.63	0.22	1.945	30.26	0.22
1.945	36.91	0.22	1.946	35.37	0.22	1.967	32.96	0.22	1.965	30.62	0.22
1.965	37.34	0.22	1.966	35.75	0.22	1.987	33.30	0.22	1.985	30.97	0.22
1.984	37.78	0.22	1.985	36.13	0.22	2.007	33.68	0.22	2.005	31.30	0.22
2.004	38.21	0.22	2.005	36.53	0.22	2.027	34.05	0.22	2.025	31.63	0.22
2.024	38.64	0.22	2.025	36.95	0.22	2.047	34.41	0.22	2.045	31.96	0.22
2.044	39.06	0.22	2.045	37.33	0.22	2.067	34.78	0.22	2.065	32.28	0.22
2.064	39.47	0.22	2.065	37.73	0.22	2.087	35.16	0.22	2.085	32.60	0.22
2.084	39.88	0.22	2.085	38.18	0.22	2.107	35.53	0.22	2.105	32.95	0.22
2.104	40.35	0.22	2.105	38.58	0.22	2.127	35.89	0.22	2.125	33.31	0.22
2.124	40.77	0.22	2.125	38.96	0.22	2.147	36.25	0.22	2.145	33.61	0.22
2.144	41.18	0.22	2.145	39.38	0.22	2.166	36.62	0.22	2.164	33.96	0.22
2.164	41.64	0.22	2.165	39.76	0.22	2.186	37.02	0.22	2.184	34.31	0.22
2.184	42.07	0.22	2.185	40.15	0.22	2.206	37.37	0.22	2.204	34.65	0.22
2.203	42.49	0.22	2.204	40.56	0.22	2.226	37.72	0.22	2.224	35.00	0.22
2.223	42.93	0.22	2.224	40.98	0.22	2.246	38.08	0.22	2.244	35.34	0.22
2.243	43.38	0.22	2.244	41.42	0.22	2.266	38.45	0.22	2.264	35.67	0.22
2.263	43.82	0.22	2.264	41.81	0.22	2.286	38.82	0.22	2.284	35.99	0.22
2.283	44.26	0.22	2.284	42.24	0.22	2.306	39.22	0.22	2.304	36.30	0.22
2.303	44.69	0.22	2.304	42.63	0.22	2.326	39.60	0.22	2.324	36.65	0.22
2.323	45.13	0.22	2.324	43.03	0.22	2.346	39.97	0.22	2.344	36.99	0.22
2.343	45.63	0.22	2.344	43.46	0.22	2.366	40.33	0.22	2.364	37.32	0.22
2.363	46.04	0.22	2.364	43.89	0.22	2.386	40.69	0.22	2.384	37.70	0.22
2.383	46.49	0.22	2.384	44.31	0.22	2.406	41.05	0.22	2.404	38.00	0.22
2.403	46.94	0.22	2.403	44.73	0.22	2.426	41.41	0.22	2.424	38.34	0.22
2.422	47.38	0.22	2.423	45.15	0.22	2.446	41.82	0.22	2.444	38.69	0.22
2.442	47.85	0.22	2.443	45.53	0.22	2.466	42.21	0.22	2.464	39.04	0.22
2.462	48.26	0.22	2.463	45.96	0.22	2.485	42.60	0.22	2.483	39.38	0.22
2.482	48.72	0.22	2.483	46.39	0.22	2.505	42.97	0.22	2.503	39.72	0.22
2.502	49.19	0.22	2.503	46.80	0.22	2.525	43.33	0.22	2.523	40.06	0.22
2.522	49.65	0.22	2.523	47.22	0.22	2.545	43.69	0.22	2.543	40.41	0.22
2.542	50.07	0.22	2.543	47.71	0.22	2.565	44.07	0.22	2.563	40.79	0.22
2.562	50.50	0.22	2.563	48.13	0.22	2.585	44.47	0.22	2.583	41.11	0.22
2.582	50.98	0.22	2.583	48.55	0.22	2.605	44.84	0.22	2.603	41.43	0.22

2.602	51.42	0.22	2.603	49.00	0.22	2.625	45.21	0.22	2.623	41.78	0.22
2.621	51.87	0.22	2.622	49.40	0.22	2.645	45.61	0.22	2.643	42.14	0.22
2.641	52.32	0.22	2.642	49.80	0.22	2.665	46.00	0.22	2.663	42.48	0.22
2.661	52.84	0.22	2.662	50.22	0.22	2.685	46.36	0.22	2.683	42.84	0.22
2.681	53.27	0.22	2.682	50.68	0.22	2.705	46.77	0.22	2.703	43.23	0.22
2.701	53.72	0.22	2.702	51.13	0.22	2.725	47.18	0.22	2.723	43.56	0.22
2.721	54.21	0.22	2.722	51.53	0.22	2.745	47.56	0.22	2.743	43.89	0.22
2.741	54.64	0.22	2.742	51.96	0.22	2.765	47.90	0.22	2.763	44.24	0.22
2.761	55.10	0.22	2.762	52.41	0.22	2.785	48.30	0.22	2.783	44.55	0.22
2.781	55.59	0.22	2.782	52.86	0.22	2.805	48.68	0.22	2.803	44.89	0.22
2.801	56.07	0.22	2.802	53.30	0.22	2.824	49.07	0.22	2.822	45.27	0.22
2.821	56.55	0.22	2.822	53.74	0.22	2.844	49.48	0.22	2.842	45.66	0.22
2.840	57.04	0.22	2.841	54.17	0.22	2.864	49.84	0.22	2.862	46.00	0.22
2.860	57.45	0.22	2.861	54.61	0.22	2.884	50.24	0.22	2.882	46.34	0.22
2.880	57.93	0.22	2.881	55.07	0.22	2.904	50.65	0.22	2.902	46.67	0.22
2.900	58.46	0.22	2.901	55.54	0.22	2.924	51.06	0.22	2.922	47.03	0.22
2.920	58.94	0.22	2.921	55.93	0.22	2.944	51.46	0.22	2.942	47.39	0.22
2.940	59.42	0.22	2.941	56.37	0.22	2.964	51.82	0.22	2.962	47.75	0.22
2.960	59.87	0.22	2.961	56.82	0.22	2.984	52.17	0.22	2.982	48.09	0.22
2.980	60.33	0.22	2.981	57.27	0.22	3.004	52.57	0.22	3.002	48.41	0.22
3.000	60.84	0.22	3.001	57.73	0.22	3.024	52.98	0.22	3.022	48.82	0.22
3.020	61.32	0.22	3.021	58.17	0.22	3.044	53.41	0.22	3.042	49.18	0.22
3.040	61.81	0.22	3.040	58.66	0.22	3.064	53.83	0.22	3.062	49.52	0.22
3.059	62.29	0.22	3.060	59.12	0.22	3.084	54.23	0.22	3.082	49.86	0.22
3.079	62.77	0.22	3.080	59.54	0.22	3.104	54.61	0.22	3.102	50.26	0.22
3.099	63.28	0.22	3.100	59.97	0.22	3.124	55.01	0.22	3.122	50.61	0.22
3.119	63.78	0.22	3.120	60.41	0.22	3.143	55.41	0.22	3.141	50.93	0.22
3.139	64.28	0.22	3.140	60.90	0.22	3.163	55.77	0.22	3.161	51.26	0.22
3.159	64.78	0.22	3.160	61.38	0.22	3.183	56.18	0.22	3.181	51.61	0.22
3.179	65.28	0.22	3.180	61.82	0.22	3.203	56.58	0.22	3.201	51.98	0.22
3.199	65.76	0.22	3.200	62.26	0.22	3.223	56.96	0.22	3.221	52.35	0.22
3.219	66.23	0.22	3.220	62.75	0.22	3.243	57.37	0.22	3.241	52.71	0.22
3.239	66.77	0.22	3.240	63.20	0.22	3.263	57.74	0.22	3.261	53.07	0.22
3.258	67.29	0.22	3.259	63.66	0.22	3.283	58.16	0.22	3.281	53.41	0.22
3.278	67.78	0.22	3.279	64.15	0.22	3.303	58.59	0.22	3.301	53.78	0.22
3.298	68.30	0.22	3.299	64.63	0.22	3.323	58.97	0.22	3.321	54.18	0.22
3.318	68.79	0.22	3.319	65.08	0.22	3.343	59.39	0.22	3.341	54.53	0.22
3.338	69.32	0.22	3.339	65.54	0.22	3.363	59.81	0.22	3.361	54.87	0.22
3.358	69.86	0.22	3.359	66.00	0.22	3.383	60.23	0.22	3.381	55.23	0.22
3.378	70.33	0.22	3.379	66.46	0.22	3.403	60.65	0.22	3.401	55.63	0.22
3.398	70.84	0.22	3.399	66.97	0.22	3.423	61.06	0.22	3.421	55.98	0.22
3.418	71.40	0.22	3.419	67.42	0.22	3.443	61.47	0.22	3.441	56.33	0.22
3.438	71.91	0.22	3.439	67.87	0.22	3.462	61.88	0.22	3.461	56.70	0.22
3.458	72.43	0.22	3.458	68.37	0.22	3.482	62.29	0.22	3.480	57.10	0.22
3.477	72.94	0.22	3.478	68.88	0.22	3.502	62.69	0.22	3.500	57.41	0.22

3.497	73.48	0.22	3.498	69.34	0.22	3.522	63.08	0.22	3.520	57.80	0.22
3.517	74.02	0.22	3.518	69.79	0.22	3.542	63.49	0.22	3.540	58.20	0.22
3.537	74.51	0.22	3.538	70.28	0.22	3.562	63.94	0.22	3.560	58.57	0.22
3.557	75.07	0.22	3.558	70.80	0.22	3.582	64.30	0.22	3.580	58.95	0.22
3.577	75.58	0.22	3.578	71.28	0.22	3.602	64.73	0.22	3.600	59.26	0.22
3.597	76.09	0.22	3.598	71.72	0.22	3.622	65.17	0.22	3.620	59.60	0.22
3.617	76.65	0.22	3.618	72.22	0.22	3.642	65.59	0.22	3.640	59.97	0.22
3.637	77.18	0.22	3.638	72.72	0.22	3.662	66.02	0.22	3.660	60.34	0.22
3.657	77.71	0.22	3.658	73.22	0.22	3.682	66.45	0.22	3.680	60.77	0.22
3.677	78.25	0.22	3.677	73.71	0.22	3.702	66.83	0.22	3.700	61.13	0.22
3.696	78.79	0.22	3.697	74.18	0.22	3.722	67.22	0.22	3.720	61.48	0.22
3.716	79.38	0.22	3.717	74.67	0.22	3.742	67.67	0.22	3.740	61.84	0.22
3.736	79.91	0.22	3.737	75.18	0.22	3.762	68.09	0.22	3.760	62.26	0.22
3.756	80.46	0.22	3.757	75.66	0.22	3.781	68.53	0.22	3.780	62.63	0.22
3.776	81.03	0.22	3.777	76.12	0.22	3.801	68.96	0.22	3.799	63.00	0.22
3.796	81.55	0.22	3.797	76.65	0.22	3.821	69.38	0.22	3.819	63.36	0.22
3.816	82.10	0.22	3.817	77.15	0.22	3.841	69.81	0.22	3.839	63.73	0.22
3.836	82.67	0.22	3.837	77.65	0.22	3.861	70.22	0.22	3.859	64.09	0.22
3.856	83.22	0.22	3.857	78.16	0.22	3.881	70.63	0.22	3.879	64.46	0.22
3.876	83.78	0.22	3.877	78.66	0.22	3.901	71.06	0.22	3.899	64.83	0.22
3.896	84.34	0.22	3.896	79.17	0.22	3.921	71.53	0.22	3.919	65.21	0.22
3.915	84.89	0.22	3.916	79.67	0.22	3.941	71.94	0.22	3.939	65.59	0.22
3.935	85.48	0.22	3.936	80.18	0.22	3.961	72.35	0.22	3.959	65.96	0.22
3.955	86.06	0.22	3.956	80.70	0.22	3.981	72.77	0.22	3.979	66.36	0.22
3.975	86.63	0.22	3.976	81.22	0.22	4.001	73.21	0.22	3.999	66.76	0.22
3.995	87.22	0.22	3.996	81.73	0.22	4.021	73.64	0.22	4.019	67.15	0.22
4.015	87.80	0.22	4.016	82.24	0.22	4.041	74.07	0.22	4.039	67.54	0.22
4.035	88.39	0.22	4.036	82.76	0.22	4.061	74.50	0.22	4.059	67.91	0.22
4.055	88.98	0.22	4.056	83.25	0.22	4.081	74.93	0.22	4.079	68.29	0.22
4.075	89.55	0.22	4.076	83.77	0.22	4.100	75.36	0.22	4.099	68.66	0.22
4.095	90.12	0.22	4.095	84.33	0.22	4.120	75.79	0.22	4.119	69.03	0.22
4.114	90.70	0.22	4.115	84.83	0.22	4.140	76.23	0.22	4.138	69.39	0.22
4.134	91.30	0.22	4.135	85.36	0.22	4.160	76.67	0.22	4.158	69.77	0.22
4.154	91.91	0.22	4.155	85.88	0.22	4.180	77.12	0.22	4.178	70.17	0.22
4.174	92.49	0.22	4.175	86.38	0.22	4.200	77.55	0.22	4.198	70.58	0.22
4.194	93.08	0.22	4.195	86.90	0.22	4.220	77.98	0.22	4.218	70.94	0.22
4.214	93.69	0.22	4.215	87.42	0.22	4.240	78.41	0.22	4.238	71.32	0.22
4.234	94.30	0.22	4.235	87.99	0.22	4.260	78.86	0.22	4.258	71.69	0.22
4.254	94.91	0.22	4.255	88.52	0.22	4.280	79.34	0.22	4.278	72.08	0.22
4.274	95.51	0.22	4.275	89.05	0.22	4.300	79.76	0.22	4.298	72.50	0.22
4.294	96.11	0.22	4.295	89.63	0.22	4.320	80.19	0.22	4.318	72.85	0.22
4.314	96.73	0.22	4.314	90.15	0.22	4.340	80.63	0.22	4.338	73.27	0.22
4.333	97.38	0.22	4.334	90.71	0.22	4.360	81.10	0.22	4.358	73.66	0.22
4.353	98.01	0.22	4.354	91.23	0.22	4.380	81.59	0.22	4.378	74.03	0.22
4.373	98.63	0.22	4.374	91.74	0.22	4.400	82.02	0.22	4.398	74.43	0.22

4.393	99.23	0.22	4.394	92.29	0.22	4.419	82.45	0.22	4.418	74.84	0.22
4.413	99.84	0.22	4.414	92.82	0.22	4.439	82.92	0.22	4.438	75.22	0.22
4.433	100.48	0.22	4.434	93.39	0.22	4.459	83.38	0.22	4.458	75.58	0.22
4.453	101.14	0.22	4.454	93.97	0.22	4.479	83.83	0.22	4.477	75.96	0.22
4.473	101.80	0.22	4.474	94.52	0.22	4.499	84.28	0.22	4.497	76.38	0.22
4.493	102.42	0.22	4.494	95.03	0.22	4.519	84.74	0.22	4.517	76.77	0.22
4.513	103.06	0.22	4.513	95.60	0.22	4.539	85.20	0.22	4.537	77.15	0.22
4.533	103.73	0.22	4.533	96.17	0.22	4.559	85.63	0.22	4.557	77.54	0.22
4.552	104.35	0.22	4.553	96.73	0.22	4.579	86.06	0.22	4.577	77.93	0.22
4.572	104.97	0.22	4.573	97.29	0.22	4.599	86.55	0.22	4.597	78.32	0.22
4.592	105.64	0.22	4.593	97.84	0.22	4.619	87.03	0.22	4.617	78.70	0.22
4.612	106.32	0.22	4.613	98.40	0.22	4.639	87.48	0.22	4.637	79.11	0.22
4.632	107.02	0.22	4.633	99.00	0.22	4.659	87.92	0.22	4.657	79.54	0.22
4.652	107.67	0.22	4.653	99.58	0.22	4.679	88.39	0.22	4.677	79.91	0.22
4.672	108.32	0.22	4.673	100.13	0.22	4.699	88.87	0.22	4.697	80.34	0.22
4.692	109.00	0.22	4.693	100.72	0.22	4.719	89.33	0.22	4.717	80.72	0.22
4.712	109.68	0.22	4.713	101.32	0.22	4.738	89.80	0.22	4.737	81.12	0.22
4.732	110.38	0.22	4.732	101.90	0.22	4.758	90.27	0.22	4.757	81.54	0.22
4.751	111.06	0.22	4.752	102.45	0.22	4.778	90.73	0.22	4.777	81.91	0.22
4.771	111.73	0.22	4.772	103.02	0.22	4.798	91.21	0.22	4.796	82.33	0.22
4.791	112.41	0.22	4.792	103.60	0.22	4.818	91.67	0.22	4.816	82.74	0.22
4.811	113.09	0.22	4.812	104.18	0.22	4.838	92.10	0.22	4.836	83.14	0.22
4.831	113.76	0.22	4.832	104.75	0.22	4.858	92.58	0.22	4.856	83.55	0.22
4.851	114.45	0.22	4.852	105.34	0.22	4.878	93.06	0.22	4.876	83.96	0.22
4.871	115.17	0.22	4.872	105.97	0.22	4.898	93.54	0.22	4.896	84.34	0.22
4.891	115.88	0.22	4.892	106.52	0.22	4.918	94.01	0.22	4.916	84.70	0.22
4.911	116.58	0.22	4.912	107.13	0.22	4.938	94.49	0.22	4.936	85.10	0.22
4.931	117.33	0.22	4.931	107.75	0.22	4.958	94.96	0.22	4.956	85.56	0.22
4.951	118.08	0.22	4.951	108.35	0.22	4.978	95.45	0.22	4.976	85.99	0.22
4.970	118.79	0.22	4.971	108.91	0.22	4.998	95.98	0.22	4.996	86.39	0.22
4.990	119.50	0.22	4.991	109.54	0.22	5.018	96.43	0.22	5.016	86.77	0.22
5.010	120.23	0.22	5.011	110.14	0.22	5.038	96.89	0.22	5.036	87.17	0.22
5.030	120.97	0.22	5.031	110.73	0.22	5.058	97.37	0.22	5.056	87.59	0.22
5.050	121.71	0.22	5.051	111.35	0.22	5.077	97.87	0.22	5.076	88.03	0.22
5.070	122.44	0.22	5.071	111.92	0.22	5.097	98.33	0.22	5.096	88.41	0.22
5.090	123.18	0.22	5.091	112.57	0.22	5.117	98.81	0.22	5.116	88.80	0.22
5.110	123.93	0.22	5.111	113.20	0.22	5.137	99.32	0.22	5.135	89.20	0.22
5.130	124.70	0.22	5.131	113.83	0.22	5.157	99.81	0.22	5.155	89.61	0.22
5.150	125.47	0.22	5.150	114.46	0.22	5.177	100.30	0.22	5.175	90.04	0.22
5.170	126.24	0.22	5.170	115.07	0.22	5.197	100.79	0.22	5.195	90.45	0.22
5.189	127.03	0.22	5.190	115.69	0.22	5.217	101.28	0.22	5.215	90.85	0.22
5.209	127.82	0.22	5.210	116.31	0.22	5.237	101.77	0.22	5.235	91.28	0.22
5.229	128.63	0.22	5.230	116.95	0.22	5.257	102.24	0.22	5.255	91.66	0.22
5.249	129.42	0.22	5.250	117.63	0.22	5.277	102.72	0.22	5.275	92.08	0.22
5.269	130.18	0.22	5.270	118.25	0.22	5.297	103.20	0.22	5.295	92.51	0.22

5.289	130.96	0.22	5.290	118.84	0.22	5.317	103.74	0.22	5.315	92.94	0.22
5.309	131.74	0.22	5.310	119.47	0.22	5.337	104.21	0.22	5.335	93.36	0.22
5.329	132.56	0.22	5.330	120.12	0.22	5.357	104.69	0.22	5.355	93.80	0.22
5.349	133.39	0.22	5.350	120.77	0.22	5.377	105.22	0.22	5.375	94.23	0.22
5.369	134.23	0.22	5.369	121.42	0.22	5.396	105.68	0.22	5.395	94.65	0.22
5.389	135.09	0.22	5.389	122.07	0.22	5.416	106.19	0.22	5.415	95.06	0.22
5.408	135.90	0.22	5.409	122.75	0.22	5.436	106.71	0.22	5.435	95.48	0.22
5.428	136.71	0.22	5.429	123.42	0.22	5.456	107.21	0.22	5.454	95.89	0.22
5.448	137.54	0.22	5.449	124.09	0.22	5.476	107.72	0.22	5.474	96.30	0.22
5.468	138.37	0.22	5.469	124.78	0.22	5.496	108.24	0.22	5.494	96.71	0.22
5.488	139.23	0.22	5.489	125.41	0.22	5.516	108.75	0.22	5.514	97.13	0.22
5.508	140.08	0.22	5.509	126.09	0.22	5.536	109.25	0.22	5.534	97.58	0.22
5.528	140.99	0.22	5.529	126.79	0.22	5.556	109.76	0.22	5.554	98.03	0.22
5.548	141.90	0.22	5.549	127.48	0.22	5.576	110.26	0.22	5.574	98.44	0.22
5.568	142.79	0.22	5.568	128.11	0.22	5.596	110.76	0.22	5.594	98.81	0.22
5.588	143.69	0.22	5.588	128.79	0.22	5.616	111.24	0.22	5.614	99.25	0.22
5.607	144.60	0.23	5.608	129.47	0.22	5.636	111.75	0.22	5.634	99.68	0.22
5.627	145.50	0.23	5.628	130.16	0.22	5.656	112.29	0.22	5.654	100.10	0.22
5.647	146.39	0.23	5.648	130.86	0.22	5.676	112.82	0.22	5.674	100.53	0.22
5.667	147.28	0.23	5.668	131.51	0.22	5.696	113.35	0.22	5.694	100.94	0.22
5.687	148.19	0.23	5.688	132.21	0.22	5.715	113.87	0.22	5.714	101.44	0.22
5.707	149.11	0.23	5.708	132.92	0.22	5.735	114.38	0.22	5.734	101.88	0.22
5.727	150.09	0.23	5.728	133.62	0.22	5.755	114.89	0.22	5.754	102.29	0.22
5.747	151.07	0.23	5.748	134.34	0.22	5.775	115.42	0.22	5.774	102.70	0.22
5.767	152.04	0.23	5.768	135.05	0.22	5.795	115.94	0.22	5.793	103.11	0.22
5.787	153.02	0.23	5.787	135.74	0.22	5.815	116.47	0.22	5.813	103.56	0.22
5.807	153.98	0.23	5.807	136.48	0.22	5.835	117.03	0.22	5.833	104.01	0.22
5.826	154.97	0.23	5.827	137.21	0.22	5.855	117.55	0.22	5.853	104.42	0.22
5.846	155.98	0.23	5.847	137.93	0.22	5.875	118.08	0.22	5.873	104.89	0.22
5.866	157.01	0.23	5.867	138.70	0.23	5.895	118.64	0.22	5.893	105.34	0.22
5.886	158.04	0.23	5.887	139.40	0.23	5.915	119.14	0.22	5.913	105.75	0.22
5.906	159.08	0.23	5.907	140.12	0.23	5.935	119.66	0.22	5.933	106.14	0.22
5.926	160.11	0.23	5.927	140.85	0.23	5.955	120.19	0.22	5.953	106.65	0.22
5.946	161.17	0.23	5.947	141.59	0.23	5.975	120.69	0.22	5.973	107.08	0.22
5.966	162.27	0.23	5.967	142.35	0.23	5.995	121.23	0.22	5.993	107.51	0.22
5.986	163.37	0.23	5.986	143.10	0.23	6.015	121.78	0.22	6.013	107.94	0.22
6.006	164.44	0.23	6.006	143.81	0.23	6.034	122.36	0.22	6.033	108.37	0.22
6.026	165.54	0.23	6.026	144.59	0.23	6.054	123.01	0.22	6.053	108.86	0.22
6.045	166.67	0.23	6.046	145.37	0.23	6.074	123.56	0.22	6.073	109.29	0.22
6.065	167.81	0.23	6.066	146.12	0.23	6.094	124.04	0.22	6.093	109.71	0.22
6.085	168.95	0.23	6.086	146.88	0.23	6.114	124.59	0.22	6.112	110.14	0.22
6.105	170.12	0.23	6.106	147.62	0.23	6.134	125.24	0.22	6.132	110.62	0.22
6.125	171.31	0.23	6.126	148.40	0.23	6.154	125.78	0.22	6.152	111.06	0.22
6.145	172.51	0.23	6.146	149.21	0.23	6.174	126.30	0.22	6.172	111.50	0.22
6.165	173.71	0.23	6.166	150.01	0.23	6.194	126.84	0.22	6.192	111.96	0.22

6.185	174.94	0.23	6.186	150.81	0.23	6.214	127.42	0.22	6.212	112.37	0.22
6.205	176.19	0.23	6.205	151.60	0.23	6.234	127.95	0.22	6.232	112.83	0.22
6.225	177.47	0.23	6.225	152.39	0.23	6.254	128.50	0.22	6.252	113.30	0.22
6.244	178.76	0.23	6.245	153.16	0.23	6.274	129.08	0.22	6.272	113.75	0.22
6.264	180.00	0.23	6.265	153.95	0.23	6.294	129.67	0.22	6.292	114.22	0.22
6.284	181.36	0.23	6.285	154.74	0.23	6.314	130.20	0.22	6.312	114.64	0.22
6.304	182.71	0.23	6.305	155.57	0.23	6.334	130.75	0.22	6.332	115.06	0.22
6.324	184.07	0.23	6.325	156.42	0.23	6.353	131.31	0.22	6.352	115.51	0.22
6.344	185.43	0.23	6.345	157.21	0.23	6.373	131.86	0.22	6.372	116.00	0.22
6.364	186.79	0.23	6.365	158.04	0.23	6.393	132.46	0.22	6.392	116.44	0.22
6.384	188.14	0.23	6.385	158.86	0.23	6.413	133.03	0.22	6.412	116.88	0.22
6.404	189.50	0.23	6.405	159.69	0.23	6.433	133.60	0.22	6.432	117.33	0.22
6.424	191.10	0.23	6.424	160.55	0.23	6.453	134.19	0.22	6.451	117.82	0.22
6.444	192.70	0.23	6.444	161.39	0.23	6.473	134.72	0.22	6.471	118.25	0.22
6.463	194.30	0.23	6.464	162.24	0.23	6.493	135.32	0.22	6.491	118.72	0.22
6.483	195.90	0.23	6.484	163.10	0.23	6.513	135.89	0.22	6.511	119.20	0.22
6.503	197.50	0.23	6.504	163.99	0.23	6.533	136.43	0.22	6.531	119.67	0.22
6.523	199.08	0.23	6.524	164.85	0.23	6.553	137.03	0.22	6.551	120.08	0.22
6.543	200.67	0.23	6.544	165.73	0.23	6.573	137.61	0.22	6.571	120.56	0.22
6.563	202.25	0.23	6.564	166.62	0.23	6.593	138.20	0.22	6.591	121.04	0.22
6.583	203.84	0.23	6.584	167.51	0.23	6.613	138.77	0.22	6.611	121.51	0.22
6.603	205.45	0.23	6.604	168.34	0.23	6.633	139.34	0.22	6.631	121.97	0.22
6.623	207.20	0.23	6.623	169.23	0.23	6.653	139.92	0.22	6.651	122.40	0.22
6.643	208.95	0.23	6.643	170.17	0.23	6.672	140.52	0.23	6.671	122.86	0.22
6.663	210.70	0.23	6.663	171.05	0.23	6.692	141.16	0.23	6.691	123.33	0.22
6.682	212.45	0.23	6.683	171.98	0.23	6.712	141.72	0.23	6.711	123.78	0.22
6.702	214.22	0.23	6.703	172.92	0.23	6.732	142.33	0.23	6.731	124.28	0.22
6.722	216.11	0.23	6.723	173.85	0.23	6.752	142.95	0.23	6.751	124.73	0.22
6.742	218.00	0.23	6.743	174.79	0.23	6.772	143.53	0.23	6.770	125.19	0.22
6.762	219.90	0.23	6.763	175.73	0.23	6.792	144.13	0.23	6.790	125.67	0.22
6.782	221.79	0.23	6.783	176.73	0.23	6.812	144.71	0.23	6.810	126.10	0.22
6.802	223.70	0.23	6.803	177.81	0.23	6.832	145.28	0.23	6.830	126.60	0.22
6.822	225.79	0.23	6.823	178.58	0.23	6.852	145.86	0.23	6.850	127.05	0.22
6.842	227.88	0.23	6.842	179.48	0.23	6.872	146.46	0.23	6.870	127.51	0.22
6.862	229.97	0.23	6.862	180.51	0.23	6.892	147.08	0.23	6.890	128.00	0.22
6.882	232.06	0.23	6.882	181.71	0.23	6.912	147.70	0.23	6.910	128.50	0.22
6.901	234.17	0.23	6.902	182.71	0.23	6.932	148.31	0.23	6.930	128.99	0.22
6.921	236.56	0.23	6.922	183.72	0.23	6.952	148.93	0.23	6.950	129.48	0.22
6.941	238.95	0.23	6.942	184.75	0.23	6.972	149.54	0.23	6.970	129.94	0.22
6.961	241.34	0.23	6.962	185.72	0.23	6.991	150.14	0.23	6.990	130.36	0.22
6.981	243.72	0.23	6.982	186.73	0.23	7.011	150.77	0.23	7.010	130.84	0.22
7.001	246.14	0.23	7.002	187.76	0.23	7.031	151.36	0.23	7.030	131.32	0.22
7.021	249.02	0.23	7.022	188.79	0.23	7.051	151.97	0.23	7.050	131.81	0.22
7.041	251.91	0.23	7.041	189.83	0.23	7.071	152.60	0.23	7.070	132.34	0.22
7.061	254.80	0.23	7.061	190.87	0.23	7.091	153.26	0.23	7.090	132.81	0.22

7.081	257.68	0.23	7.081	191.92	0.23	7.111	153.91	0.23	7.109	133.28	0.22
7.100	260.58	0.23	7.101	193.01	0.23	7.131	154.52	0.23	7.129	133.76	0.22
7.120	263.86	0.23	7.121	194.08	0.23	7.151	155.14	0.23	7.149	134.22	0.22
7.140	267.15	0.23	7.141	195.16	0.23	7.171	155.78	0.23	7.169	134.70	0.22
7.160	270.43	0.23	7.161	196.28	0.23	7.191	156.36	0.23	7.189	135.20	0.22
7.180	273.72	0.23	7.181	197.33	0.23	7.211	156.99	0.23	7.209	135.67	0.22
7.200	277.00	0.23	7.201	198.44	0.23	7.231	157.64	0.23	7.229	136.13	0.22
7.220	281.18	0.21	7.221	199.57	0.23	7.251	158.29	0.23	7.249	136.63	0.22
7.240	285.36	0.21	7.241	200.66	0.23	7.271	158.94	0.23	7.269	137.12	0.22
7.260	289.54	0.21	7.260	201.79	0.23	7.291	159.59	0.23	7.289	137.62	0.22
7.280	293.72	0.22	7.280	202.96	0.23	7.311	160.23	0.23	7.309	138.12	0.22
7.300	297.90	0.22	7.300	204.14	0.23	7.330	160.87	0.23	7.329	138.62	0.22
7.319	302.67	0.22	7.320	205.28	0.23	7.350	161.51	0.23	7.349	139.11	0.22
7.339	307.44	0.22	7.340	206.45	0.23	7.370	162.15	0.23	7.369	139.61	0.22
7.359	312.22	0.22	7.360	207.65	0.23	7.390	162.79	0.23	7.389	140.12	0.22
7.379	317.00	0.22	7.380	208.84	0.23	7.410	163.44	0.23	7.409	140.61	0.23
7.399	321.78	0.22	7.400	210.07	0.23	7.430	164.13	0.23	7.428	141.10	0.23
7.419	327.88	0.22	7.420	211.29	0.23	7.450	164.77	0.23	7.448	141.58	0.23
7.439	334.05	0.22	7.440	212.47	0.23	7.470	165.40	0.23	7.468	142.06	0.23
7.459	340.23	0.22	7.460	213.78	0.23	7.490	166.06	0.23	7.488	142.56	0.23
7.479	346.40	0.22	7.479	215.04	0.23	7.510	166.73	0.23	7.508	143.05	0.23
7.499	352.57	0.22	7.499	216.27	0.23	7.530	167.40	0.23	7.528	143.54	0.23
7.519	359.29	0.22	7.519	217.51	0.23	7.550	168.07	0.23	7.548	144.08	0.23
7.538	366.06	0.23	7.539	218.82	0.23	7.570	168.73	0.23	7.568	144.58	0.23
7.558	372.83	0.23	7.559	220.14	0.23	7.590	169.39	0.23	7.588	145.06	0.23
7.578	379.60	0.23	7.579	221.45	0.23	7.610	170.12	0.23	7.608	145.54	0.23
7.598	386.37	0.23	7.599	222.81	0.23	7.630	170.81	0.23	7.628	146.08	0.23
7.618	393.32	0.23	7.619	224.16	0.23	7.649	171.45	0.23	7.648	146.57	0.23
7.638	400.28	0.23	7.639	225.52	0.23	7.669	172.16	0.23	7.668	147.06	0.23
7.658	407.25	0.23	7.659	226.84	0.23	7.689	172.83	0.23	7.688	147.59	0.23
7.678	414.22	0.23	7.678	228.26	0.23	7.709	173.52	0.23	7.708	148.13	0.23
7.698	421.19	0.24	7.698	229.67	0.23	7.729	174.22	0.23	7.728	148.62	0.23
7.718	428.68	0.24	7.718	231.04	0.23	7.749	174.93	0.23	7.748	149.11	0.23
7.737	436.25	0.24	7.738	232.50	0.23	7.769	175.63	0.23	7.767	149.63	0.23
7.757	443.81	0.24	7.758	233.99	0.23	7.789	176.33	0.23	7.787	150.17	0.23
7.777	451.37	0.24	7.778	235.49	0.23	7.809	177.02	0.23	7.807	150.69	0.23
7.797	458.94	0.24	7.798	236.96	0.23	7.829	177.72	0.23	7.827	151.19	0.23
7.817	465.99	0.25	7.818	238.49	0.23	7.849	178.42	0.23	7.847	151.66	0.23
7.837	472.96	0.25	7.838	240.00	0.23	7.869	179.12	0.23	7.867	152.18	0.23
7.857	479.92	0.25	7.858	241.51	0.23	7.889	179.81	0.23	7.887	152.69	0.23
7.877	486.89	0.25	7.878	243.07	0.23	7.909	180.51	0.23	7.907	153.18	0.23
7.897	493.86	0.25	7.897	244.64	0.23	7.929	181.24	0.23	7.927	153.68	0.23
7.917	500.99	0.25	7.917	246.22	0.23	7.949	181.97	0.23	7.947	154.25	0.23
7.937	508.16	0.26	7.937	247.82	0.23	7.968	182.66	0.23	7.967	154.76	0.23
7.956	515.33	0.26	7.957	249.45	0.23	7.988	183.35	0.23	7.987	155.28	0.23

7.976	522.49	0.26	7.977	251.11	0.23	8.008	184.07	0.23	8.007	155.81	0.23
7.996	529.66	0.26	7.997	252.79	0.23	8.028	184.80	0.23	8.027	156.31	0.23
8.016	535.37	0.26	8.017	254.52	0.23	8.048	185.54	0.23	8.047	156.87	0.23
8.036	540.74	0.26	8.037	256.23	0.23	8.068	186.29	0.23	8.067	157.38	0.23
8.056	546.12	0.27	8.057	257.94	0.23	8.088	187.02	0.23	8.086	157.89	0.23
8.076	551.49	0.27	8.077	259.67	0.23	8.108	187.70	0.23	8.106	158.42	0.23
8.096	556.87	0.27	8.096	261.48	0.23	8.128	188.44	0.23	8.126	158.91	0.23
8.116	561.61	0.27	8.116	263.31	0.23	8.148	189.17	0.23	8.146	159.45	0.23
8.136	566.19	0.27	8.136	265.15	0.23	8.168	189.89	0.23	8.166	160.01	0.23
8.156	570.77	0.27	8.156	267.01	0.23	8.188	190.63	0.23	8.186	160.55	0.23
8.175	575.35	0.27	8.176	268.89	0.23	8.208	191.41	0.23	8.206	161.08	0.23
8.195	579.93	0.27	8.196	270.79	0.23	8.228	192.18	0.23	8.226	161.57	0.23
8.215	583.90	0.28	8.216	272.74	0.23	8.248	192.91	0.23	8.246	162.09	0.23
8.235	587.68	0.28	8.236	274.72	0.23	8.268	193.60	0.23	8.266	162.64	0.23
8.255	591.46	0.28	8.256	276.71	0.23	8.287	194.36	0.23	8.286	163.19	0.23
8.275	595.24	0.28	8.276	278.71	0.23	8.307	195.15	0.23	8.306	163.72	0.23
8.295	599.03	0.28	8.296	280.70	0.23	8.327	195.88	0.23	8.326	164.26	0.23
8.315	602.22	0.28	8.315	282.90	0.23	8.347	196.65	0.23	8.346	164.80	0.23
8.335	605.20	0.28	8.335	285.06	0.23	8.367	197.40	0.23	8.366	165.35	0.23
8.355	608.19	0.28	8.355	287.21	0.23	8.387	198.16	0.23	8.386	165.87	0.23
8.375	611.18	0.28	8.375	289.36	0.23	8.407	198.94	0.23	8.406	166.40	0.23
8.394	614.16	0.28	8.395	291.61	0.23	8.427	199.72	0.23	8.425	166.93	0.23
8.414	616.86	0.28	8.415	293.85	0.23	8.447	200.50	0.23	8.445	167.47	0.23
8.434	619.45	0.28	8.435	296.10	0.23	8.467	201.29	0.23	8.465	168.06	0.23
8.454	622.04	0.29	8.455	298.38	0.23	8.487	202.09	0.23	8.485	168.61	0.23
8.474	624.62	0.29	8.475	300.72	0.23	8.507	202.87	0.23	8.505	169.14	0.23
8.494	627.21	0.29	8.495	303.07	0.23	8.527	203.64	0.23	8.525	169.67	0.23
8.514	629.52	0.29	8.514	305.44	0.23	8.547	204.41	0.23	8.545	170.23	0.23
8.534	631.71	0.29	8.534	307.84	0.23	8.567	205.17	0.23	8.565	170.76	0.23
8.554	633.90	0.29	8.554	310.29	0.23	8.587	206.03	0.23	8.585	171.31	0.23
8.574	636.09	0.29	8.574	312.79	0.23	8.606	206.81	0.23	8.605	171.89	0.23
8.593	638.28	0.29	8.594	315.37	0.23	8.626	207.61	0.23	8.625	172.41	0.23
8.613	640.20	0.29	8.614	317.92	0.23	8.646	208.44	0.23	8.645	172.96	0.23
8.633	642.00	0.29	8.634	320.40	0.23	8.666	209.22	0.23	8.665	173.53	0.23
8.653	643.79	0.29	8.654	322.93	0.23	8.686	210.03	0.23	8.685	174.07	0.23
8.673	645.58	0.29	8.674	325.74	0.23	8.706	210.86	0.23	8.705	174.60	0.23
8.693	647.37	0.29	8.694	328.54	0.23	8.726	211.70	0.23	8.725	175.16	0.23
8.713	648.90	0.29	8.714	331.39	0.23	8.746	212.52	0.23	8.744	175.72	0.23
8.733	650.30	0.29	8.733	334.26	0.23	8.766	213.31	0.23	8.764	176.27	0.23
8.753	651.69	0.29	8.753	337.20	0.23	8.786	214.10	0.23	8.784	176.81	0.23
8.773	653.08	0.29	8.773	340.24	0.23	8.806	214.92	0.23	8.804	177.41	0.23
8.793	654.48	0.29	8.793	343.32	0.23	8.826	215.80	0.23	8.824	178.00	0.23
8.812	655.87	0.29	8.813	346.39	0.23	8.846	216.64	0.23	8.844	178.57	0.23
8.832	657.26	0.30	8.833	349.49	0.23	8.866	217.46	0.23	8.864	179.25	0.23
8.852	658.66	0.30	8.853	352.83	0.23	8.886	218.28	0.23	8.884	179.70	0.23

8.872	660.05	0.30	8.873	356.11	0.23	8.906	219.14	0.23	8.904	180.27	0.23
8.892	661.45	0.30	8.893	359.39	0.23	8.925	220.00	0.23	8.924	180.92	0.23
8.912	662.72	0.30	8.913	362.69	0.23	8.945	220.86	0.23	8.944	181.54	0.23
8.932	663.91	0.30	8.933	366.02	0.23	8.965	221.67	0.23	8.964	182.08	0.23
8.952	665.11	0.30	8.952	369.39	0.24	8.985	222.55	0.23	8.984	182.61	0.23
8.972	666.30	0.30	8.972	372.67	0.24	9.005	223.42	0.23	9.004	183.17	0.23
8.992	667.50	0.30	8.992	376.06	0.24	9.025	224.29	0.23	9.024	183.78	0.23
9.012	668.66	0.30	9.012	379.57	0.24	9.045	225.17	0.23	9.044	184.38	0.23
9.031	669.79	0.30	9.032	383.07	0.24	9.065	226.03	0.23	9.064	184.94	0.23
9.051	670.92	0.30	9.052	386.48	0.24	9.085	226.89	0.23	9.083	185.49	0.23
9.071	672.05	0.30	9.072	389.84	0.24	9.105	227.74	0.23	9.103	186.07	0.23
9.091	673.19	0.30	9.092	393.34	0.24	9.125	228.63	0.23	9.123	186.67	0.23
9.111	674.32	0.30	9.112	396.84	0.24	9.145	229.49	0.23	9.143	187.23	0.23
9.131	675.30	0.30	9.132	400.31	0.24	9.165	230.38	0.23	9.163	187.81	0.23
9.151	676.54	0.30	9.151	403.82	0.24	9.185	231.29	0.23	9.183	188.40	0.23
9.171	677.66	0.30	9.171	407.29	0.24	9.205	232.12	0.23	9.203	188.98	0.23
9.191	678.85	0.30	9.191	410.73	0.24	9.225	233.03	0.23	9.223	189.58	0.23
9.211	679.98	0.30	9.211	414.13	0.24	9.244	233.94	0.23	9.243	190.17	0.23
9.230	680.92	0.30	9.231	417.53	0.24	9.264	234.84	0.23	9.263	190.76	0.23
9.250	681.77	0.30	9.251	420.88	0.24	9.284	235.75	0.23	9.283	191.33	0.23
9.270	682.65	0.30	9.271	424.14	0.24	9.304	236.67	0.23	9.303	191.91	0.23
9.290	683.65	0.30	9.291	427.29	0.24	9.324	237.57	0.23	9.323	192.49	0.23
9.310	684.68	0.30	9.311	430.39	0.24	9.344	238.49	0.23	9.343	193.09	0.23
9.330	685.65	0.30	9.331	433.21	0.25	9.364	239.46	0.23	9.363	193.68	0.23
9.350	686.69	0.30	9.351	436.00	0.25	9.384	240.37	0.23	9.383	194.27	0.23
9.370	687.67	0.30	9.370	438.95	0.25	9.404	241.31	0.23	9.402	194.85	0.23
9.390	688.52	0.30	9.390	442.11	0.25	9.424	242.24	0.23	9.422	195.42	0.23
9.410	689.26	0.30	9.410	445.39	0.25	9.444	243.15	0.23	9.442	196.07	0.23
9.430	690.13	0.30	9.430	448.66	0.25	9.464	244.12	0.23	9.462	196.68	0.23
9.449	691.08	0.30	9.450	451.98	0.25	9.484	245.03	0.23	9.482	197.25	0.23
9.469	692.05	0.30	9.470	455.25	0.25	9.504	245.94	0.23	9.502	197.81	0.23
9.489	693.00	0.30	9.490	458.43	0.25	9.524	246.87	0.23	9.522	198.45	0.23
9.509	693.93	0.30	9.510	461.44	0.25	9.544	247.89	0.23	9.542	199.08	0.23
9.529	694.82	0.30	9.530	464.25	0.25	9.564	248.87	0.23	9.562	199.69	0.23
9.549	695.66	0.30	9.550	467.09	0.25	9.583	249.82	0.23	9.582	200.27	0.23
9.569	696.51	0.31	9.569	470.06	0.25	9.603	250.75	0.23	9.602	200.84	0.23
9.589	697.37	0.31	9.589	473.20	0.25	9.623	251.70	0.23	9.622	201.43	0.23
9.609	698.20	0.31	9.609	476.39	0.25	9.643	252.70	0.23	9.642	202.05	0.23
9.629	698.98	0.31	9.629	479.42	0.25	9.663	253.70	0.23	9.662	202.69	0.23
9.649	699.66	0.31	9.649	482.23	0.26	9.683	254.69	0.23	9.682	203.29	0.23
9.668	700.32	0.31	9.669	484.94	0.26	9.703	255.63	0.23	9.702	203.88	0.23
9.688	700.93	0.31	9.689	487.82	0.26	9.723	256.62	0.23	9.722	204.51	0.23
9.708	701.73	0.31	9.709	491.08	0.26	9.743	257.63	0.23	9.741	205.13	0.23
9.728	702.61	0.31	9.729	494.25	0.26	9.763	258.65	0.23	9.761	205.75	0.23
9.748	703.41	0.31	9.749	497.47	0.26	9.783	259.62	0.23	9.781	206.38	0.23

9.768	704.20	0.31	9.769	500.57	0.26	9.803	260.63	0.23	9.801	206.99	0.23
9.788	705.02	0.31	9.788	503.53	0.26	9.823	261.65	0.23	9.821	207.61	0.23
9.808	705.84	0.31	9.808	506.45	0.26	9.843	262.65	0.23	9.841	208.23	0.23
9.828	706.58	0.31	9.828	509.34	0.26	9.863	263.64	0.23	9.861	208.84	0.23
9.848	707.27	0.31	9.848	512.08	0.26	9.883	264.66	0.23	9.881	209.46	0.23
9.868	707.96	0.31	9.868	514.72	0.26	9.902	265.70	0.23	9.901	210.07	0.23
9.887	708.71	0.31	9.888	517.46	0.26	9.922	266.76	0.23	9.921	210.68	0.23
9.907	709.50	0.31	9.908	520.00	0.26	9.942	267.82	0.23	9.941	211.29	0.23
9.927	710.24	0.31	9.928	522.54	0.26	9.962	268.85	0.23	9.961	211.89	0.23
9.947	711.03	0.31	9.948	524.97	0.27	9.982	269.83	0.23	9.981	212.49	0.23
9.967	711.74	0.31	9.968	527.31	0.27	10.002	270.87	0.23	10.001	213.10	0.23
9.987	712.43	0.31	9.988	529.57	0.27	10.022	271.94	0.23	10.021	213.75	0.23
10.007	713.19	0.31	10.007	531.86	0.27	10.042	273.02	0.23	10.041	214.39	0.23
10.027	713.86	0.31	10.027	534.10	0.27	10.062	274.06	0.23	10.061	215.02	0.23
10.047	714.53	0.31	10.047	536.23	0.27	10.082	275.14	0.23	10.080	215.68	0.23
10.067	715.19	0.31	10.067	538.30	0.27	10.102	276.22	0.23	10.100	216.29	0.23
10.086	715.81	0.31	10.087	540.36	0.27	10.122	277.30	0.23	10.120	216.93	0.23
10.106	716.43	0.31	10.107	542.25	0.27	10.142	278.31	0.23	10.140	217.56	0.23
10.126	717.07	0.31	10.127	544.11	0.27	10.162	279.36	0.23	10.160	218.17	0.23
10.146	717.76	0.31	10.147	545.83	0.27	10.182	280.44	0.23	10.180	218.79	0.23
10.166	718.43	0.31	10.167	547.53	0.27	10.202	281.54	0.23	10.200	219.42	0.23
10.186	719.10	0.31	10.187	549.06	0.27	10.221	282.62	0.23	10.220	220.08	0.23
10.206	719.78	0.31	10.206	550.61	0.27	10.241	283.71	0.23	10.240	220.75	0.23
10.226	720.38	0.31	10.226	552.24	0.27	10.261	284.79	0.23	10.260	221.34	0.23
10.246	720.96	0.31	10.246	554.20	0.27	10.281	285.85	0.23	10.280	221.99	0.23
10.266	721.55	0.31	10.266	556.12	0.27	10.301	286.94	0.23	10.300	222.66	0.23
10.286	722.20	0.31	10.286	558.07	0.27	10.321	288.07	0.23	10.320	223.32	0.23
10.305	722.81	0.31	10.306	560.01	0.27	10.341	289.18	0.23	10.340	223.96	0.23
10.325	723.47	0.31	10.326	561.86	0.27	10.361	290.32	0.24	10.360	224.59	0.23
10.345	724.11	0.31	10.346	563.73	0.27	10.381	291.48	0.24	10.380	225.23	0.23
10.365	724.72	0.31	10.366	565.49	0.27	10.401	292.56	0.24	10.399	225.90	0.23
10.385	725.42	0.31	10.386	567.19	0.28	10.421	293.68	0.24	10.419	226.53	0.23
10.405	726.02	0.31	10.406	568.88	0.28	10.441	294.80	0.24	10.439	227.18	0.23
10.425	726.61	0.31	10.425	570.57	0.28	10.461	295.88	0.24	10.459	227.82	0.23
10.445	727.20	0.31	10.445	572.19	0.28	10.481	296.99	0.24	10.479	228.47	0.23
10.465	727.77	0.31	10.465	573.70	0.28	10.501	298.10	0.24	10.499	229.17	0.23
10.485	728.37	0.31	10.485	575.12	0.28	10.521	299.24	0.24	10.519	229.84	0.23
10.505	728.98	0.31	10.505	576.52	0.28	10.540	300.40	0.24	10.539	230.48	0.23
10.524	729.56	0.31	10.525	578.21	0.28	10.560	301.57	0.24	10.559	231.12	0.23
10.544	730.10	0.31	10.545	579.89	0.28	10.580	302.74	0.24	10.579	231.77	0.23
10.564	730.62	0.31	10.565	581.54	0.28	10.600	303.86	0.24	10.599	232.45	0.23
10.584	731.09	0.31	10.585	583.27	0.28	10.620	304.98	0.24	10.619	233.13	0.23
10.604	731.69	0.31	10.605	584.93	0.28	10.640	306.11	0.24	10.639	233.78	0.23
10.624	732.30	0.31	10.624	586.62	0.28	10.660	307.23	0.24	10.659	234.44	0.23
10.644	732.88	0.31	10.644	588.21	0.28	10.680	308.35	0.24	10.679	235.11	0.23

10.664	733.44	0.31	10.664	589.76	0.28	10.700	309.50	0.24	10.699	235.79	0.23
10.684	734.01	0.31	10.684	591.26	0.28	10.720	310.66	0.24	10.719	236.42	0.23
10.704	734.57	0.31	10.704	592.73	0.28	10.740	311.84	0.24	10.738	237.11	0.23
10.723	735.12	0.31	10.724	594.20	0.28	10.760	313.06	0.24	10.758	237.80	0.23
10.743	735.66	0.31	10.744	595.66	0.28	10.780	314.29	0.24	10.778	238.47	0.23
10.763	736.23	0.32	10.764	597.07	0.28	10.800	315.51	0.24	10.798	239.09	0.23
10.783	736.76	0.32	10.784	598.37	0.28	10.820	316.68	0.24	10.818	239.78	0.23
10.803	737.27	0.32	10.804	599.70	0.28	10.840	317.87	0.24	10.838	240.47	0.23
10.823	737.81	0.32	10.824	601.03	0.28	10.859	319.08	0.24	10.858	241.15	0.23
10.843	738.31	0.32	10.843	602.31	0.28	10.879	320.28	0.24	10.878	241.78	0.23
10.863	738.79	0.32	10.863	603.55	0.28	10.899	321.49	0.24	10.898	242.45	0.23
10.883	739.34	0.32	10.883	604.78	0.28	10.919	322.68	0.24	10.918	243.16	0.23
10.903	739.79	0.32	10.903	606.01	0.28	10.939	323.87	0.24	10.938	243.90	0.23
10.923	740.35	0.32	10.923	607.22	0.28	10.959	325.06	0.24	10.958	244.57	0.23
10.942	740.91	0.32	10.943	608.40	0.29	10.979	326.28	0.24	10.978	245.23	0.23
10.962	741.41	0.32	10.963	609.44	0.29	10.999	327.57	0.24	10.998	245.90	0.23
10.982	741.92	0.32	10.983	610.45	0.29	11.019	328.81	0.24	11.018	246.59	0.23
11.002	742.45	0.32	11.003	611.41	0.29	11.039	330.05	0.24	11.038	247.23	0.23
11.020	742.97	0.32	11.023	612.72	0.29	11.059	331.32	0.24	11.057	247.87	0.23
11.040	743.49	0.32	11.043	613.93	0.29	11.079	332.62	0.24	11.077	248.56	0.23
11.060	744.00	0.32	11.062	615.10	0.29	11.099	333.89	0.24	11.097	249.32	0.23
11.080	744.50	0.32	11.082	616.29	0.29	11.119	335.11	0.24	11.117	250.01	0.23
11.100	745.00	0.32	11.102	617.47	0.29	11.139	336.36	0.24	11.137	250.69	0.23
11.120	745.48	0.32	11.122	618.65	0.29	11.159	337.67	0.24	11.157	251.37	0.23
11.140	745.96	0.32	11.142	619.83	0.29	11.178	338.90	0.24	11.177	252.05	0.23
11.160	746.43	0.32	11.162	620.94	0.29	11.198	340.15	0.24	11.197	252.74	0.23
11.180	746.92	0.32	11.182	622.04	0.29	11.218	341.43	0.24	11.217	253.49	0.23
11.200	747.38	0.32	11.202	623.10	0.29	11.238	342.73	0.24	11.237	254.21	0.23
11.220	747.80	0.32	11.222	624.13	0.29	11.258	344.01	0.24	11.257	254.88	0.23
11.240	748.22	0.32	11.242	625.22	0.29	11.278	345.29	0.24	11.277	255.56	0.23
11.260	748.64	0.32	11.261	626.27	0.29	11.298	346.57	0.24	11.297	256.30	0.23
11.280	749.09	0.32	11.281	627.31	0.29	11.318	347.84	0.24	11.317	256.99	0.23
11.300	749.59	0.32	11.301	628.34	0.29	11.338	349.10	0.24	11.337	257.67	0.23
11.320	750.08	0.32	11.321	629.27	0.29	11.358	350.42	0.24	11.357	258.39	0.23
11.340	750.54	0.32	11.341	630.14	0.29	11.378	351.73	0.24	11.377	259.11	0.23
11.360	751.03	0.32	11.361	631.05	0.29	11.398	353.03	0.24	11.396	259.81	0.23
11.380	751.50	0.32	11.381	632.15	0.29	11.418	354.33	0.24	11.416	260.51	0.23
11.400	751.97	0.32	11.401	633.21	0.29	11.438	355.58	0.24	11.436	261.24	0.23
11.420	752.43	0.32	11.421	634.26	0.29	11.458	356.87	0.24	11.456	261.91	0.23
11.440	752.86	0.32	11.441	635.28	0.29	11.478	358.19	0.24	11.476	262.64	0.23
11.460	753.29	0.32	11.461	636.23	0.29	11.497	359.48	0.24	11.496	263.37	0.23
11.480	753.71	0.32	11.480	637.24	0.29	11.517	360.77	0.24	11.516	264.06	0.23
11.500	754.17	0.32	11.500	638.27	0.29	11.537	362.07	0.24	11.536	264.77	0.23
11.520	754.59	0.32	11.520	639.24	0.29	11.557	363.38	0.24	11.556	265.51	0.23
11.540	754.94	0.32	11.540	640.20	0.29	11.577	364.69	0.24	11.576	266.24	0.23

11.560	755.38	0.32	11.560	641.18	0.29	11.597	366.00	0.24	11.596	266.95	0.23
11.579	755.84	0.32	11.580	642.16	0.29	11.617	367.31	0.24	11.616	267.71	0.23
11.599	756.31	0.32	11.600	643.13	0.29	11.637	368.62	0.24	11.636	268.42	0.23
11.619	756.79	0.32	11.620	644.06	0.29	11.657	369.90	0.24	11.656	269.13	0.23
11.639	757.25	0.32	11.640	645.04	0.29	11.677	371.15	0.24	11.676	269.85	0.23
11.659	757.65	0.32	11.660	645.98	0.29	11.697	372.42	0.24	11.696	270.57	0.23
11.679	758.11	0.32	11.679	646.81	0.29	11.717	373.71	0.24	11.715	271.27	0.23
11.699	758.58	0.32	11.699	647.69	0.29	11.737	375.03	0.24	11.735	272.00	0.23
11.719	759.00	0.32	11.719	648.60	0.29	11.757	376.30	0.24	11.755	272.78	0.23
11.739	759.41	0.32	11.739	649.50	0.30	11.777	377.55	0.24	11.775	273.49	0.23
11.759	759.85	0.32	11.759	650.33	0.30	11.797	378.78	0.24	11.795	274.19	0.23
11.779	760.31	0.32	11.779	651.17	0.30	11.817	379.98	0.25	11.815	274.92	0.23
11.798	760.74	0.32	11.799	652.03	0.30	11.836	381.22	0.25	11.835	275.68	0.24
11.818	761.15	0.32	11.819	652.88	0.30	11.856	382.54	0.25	11.855	276.43	0.24
11.838	761.56	0.32	11.839	653.72	0.30	11.876	383.95	0.25	11.875	277.15	0.24
11.858	761.95	0.32	11.859	654.51	0.30	11.896	385.23	0.25	11.895	277.87	0.24
11.878	762.35	0.32	11.879	655.34	0.30	11.916	386.58	0.25	11.915	278.61	0.24
11.898	762.77	0.32	11.898	656.23	0.30	11.936	387.91	0.25	11.935	279.37	0.24
11.918	763.16	0.32	11.918	656.97	0.30	11.956	389.15	0.25	11.955	280.11	0.24
11.938	763.52	0.32	11.938	657.73	0.30	11.976	390.53	0.25	11.975	280.85	0.24
11.958	763.85	0.32	11.958	658.50	0.30	11.996	391.84	0.25	11.995	281.54	0.24
11.978	764.20	0.32	11.978	659.25	0.30	12.016	393.15	0.25	12.015	282.32	0.24
11.998	764.66	0.32	11.998	659.99	0.30	12.036	394.50	0.25	12.035	283.06	0.24
12.017	765.03	0.32	12.018	660.68	0.30	12.056	395.80	0.25	12.054	283.75	0.24
12.037	765.46	0.32	12.038	661.37	0.30	12.076	397.13	0.25	12.074	284.50	0.24
12.057	765.89	0.32	12.058	662.04	0.30	12.096	398.46	0.25	12.094	285.25	0.24
12.077	766.30	0.32	12.078	662.77	0.30	12.116	399.75	0.25	12.114	286.00	0.24
12.097	766.71	0.32	12.097	663.60	0.30	12.136	401.02	0.25	12.134	286.74	0.24
12.117	767.08	0.32	12.117	664.36	0.30	12.155	402.26	0.25	12.154	287.47	0.24
12.137	767.43	0.32	12.137	665.13	0.30	12.175	403.47	0.25	12.174	288.23	0.24
12.157	767.89	0.32	12.157	665.89	0.30	12.195	404.83	0.25	12.194	288.94	0.24
12.177	768.28	0.32	12.177	666.58	0.30	12.215	406.14	0.25	12.214	289.69	0.24
12.197	768.66	0.32	12.197	667.33	0.30	12.235	407.45	0.25	12.234	290.50	0.24
12.216	769.06	0.32	12.217	668.04	0.30	12.255	408.80	0.25	12.254	291.22	0.24
12.236	769.43	0.32	12.237	668.78	0.30	12.275	410.14	0.25	12.274	291.94	0.24
12.256	769.78	0.32	12.257	669.53	0.30	12.295	411.47	0.25	12.294	292.69	0.24
12.276	770.15	0.32	12.277	670.23	0.30	12.315	412.81	0.25	12.314	293.45	0.24
12.296	770.53	0.32	12.297	670.96	0.30	12.335	414.13	0.25	12.334	294.22	0.24
12.316	770.91	0.32	12.316	671.66	0.30	12.355	415.43	0.25	12.354	295.02	0.24
12.336	771.26	0.32	12.336	672.41	0.30	12.375	416.78	0.25	12.373	295.76	0.24
12.356	771.57	0.32	12.356	673.12	0.30	12.395	418.06	0.25	12.393	296.52	0.24
12.376	771.91	0.32	12.376	673.79	0.30	12.415	419.33	0.25	12.413	297.30	0.24
12.396	772.27	0.32	12.396	674.47	0.30	12.435	420.61	0.25	12.433	298.04	0.24
12.416	772.65	0.32	12.416	675.18	0.30	12.455	421.93	0.25	12.453	298.83	0.24
12.435	773.03	0.32	12.436	675.89	0.30	12.474	423.24	0.25	12.473	299.58	0.24

12.455	773.39	0.32	12.456	676.57	0.30	12.494	424.53	0.25	12.493	300.33	0.24
12.475	773.76	0.32	12.476	677.23	0.30	12.514	425.80	0.25	12.513	301.12	0.24
12.495	774.17	0.32	12.496	677.87	0.30	12.534	427.06	0.25	12.533	301.89	0.24
12.515	774.52	0.32	12.516	678.50	0.30	12.554	428.32	0.25	12.553	302.64	0.24
12.535	774.86	0.32	12.535	679.02	0.30	12.574	429.59	0.25	12.573	303.39	0.24
12.555	775.21	0.32	12.555	679.64	0.30	12.594	430.89	0.25	12.593	304.17	0.24
12.575	775.60	0.32	12.575	680.34	0.30	12.614	432.16	0.25	12.613	304.94	0.24
12.595	775.98	0.32	12.595	681.02	0.30	12.634	433.41	0.25	12.633	305.74	0.24
12.615	776.35	0.32	12.615	681.68	0.30	12.654	434.60	0.25	12.653	306.51	0.24
12.635	776.66	0.33	12.635	682.35	0.30	12.674	435.85	0.25	12.673	307.23	0.24
12.654	777.00	0.33	12.655	682.95	0.30	12.694	437.14	0.25	12.693	308.00	0.24
12.674	777.36	0.33	12.675	683.57	0.30	12.714	438.31	0.25	12.712	308.75	0.24
12.694	777.70	0.33	12.695	684.21	0.30	12.734	439.44	0.25	12.732	309.53	0.24
12.714	778.04	0.33	12.715	684.83	0.30	12.754	440.55	0.25	12.752	310.37	0.24
12.734	778.38	0.33	12.734	685.48	0.30	12.774	441.82	0.25	12.772	311.13	0.24
12.754	778.74	0.33	12.754	686.09	0.30	12.793	443.06	0.25	12.792	311.92	0.24
12.774	779.12	0.33	12.774	686.76	0.30	12.813	444.33	0.26	12.812	312.71	0.24
12.794	779.45	0.33	12.794	687.39	0.30	12.833	445.62	0.26	12.832	313.47	0.24
12.814	779.76	0.33	12.814	688.00	0.30	12.853	446.89	0.26	12.852	314.19	0.24
12.834	780.11	0.33	12.834	688.63	0.30	12.873	448.19	0.26	12.872	314.93	0.24
12.854	780.45	0.33	12.854	689.21	0.30	12.893	449.43	0.26	12.892	315.71	0.24
12.873	780.75	0.33	12.874	689.84	0.30	12.913	450.65	0.26	12.912	316.51	0.24
12.893	781.05	0.33	12.894	690.45	0.30	12.933	451.87	0.26	12.932	317.30	0.24
12.913	781.37	0.33	12.914	691.03	0.31	12.953	453.08	0.26	12.952	318.07	0.24
12.933	781.69	0.33	12.934	691.60	0.31	12.973	454.31	0.26	12.972	318.89	0.24
12.953	781.96	0.33	12.953	692.21	0.31	12.993	455.56	0.26	12.992	319.68	0.24
12.973	782.27	0.33	12.973	692.81	0.31	13.013	456.83	0.26	13.012	320.42	0.24
12.993	782.62	0.33	12.993	693.34	0.31	13.033	458.03	0.26	13.031	321.21	0.24
13.013	782.99	0.33	13.013	693.89	0.31	13.053	459.20	0.26	13.051	321.95	0.24
13.033	783.30	0.33	13.033	694.44	0.31	13.073	460.38	0.26	13.071	322.77	0.24
13.053	783.62	0.33	13.053	694.95	0.31	13.093	461.59	0.26	13.091	323.58	0.24
13.072	783.94	0.33	13.073	695.47	0.31	13.112	462.79	0.26	13.111	324.38	0.24
13.092	784.26	0.33	13.093	696.01	0.31	13.132	463.96	0.26	13.131	325.25	0.24
13.112	784.60	0.33	13.113	696.57	0.31	13.152	465.10	0.26	13.151	325.97	0.24
13.132	784.98	0.33	13.133	697.16	0.31	13.172	466.24	0.26	13.171	326.74	0.24
13.152	785.32	0.33	13.152	697.72	0.31	13.192	467.38	0.26	13.191	327.52	0.24
13.172	785.63	0.33	13.172	698.32	0.31	13.212	468.52	0.26	13.211	328.28	0.24
13.192	785.94	0.33	13.192	698.92	0.31	13.232	469.70	0.26	13.231	329.10	0.24
13.212	786.25	0.33	13.212	699.48	0.31	13.252	470.88	0.26	13.251	329.93	0.24
13.232	786.60	0.33	13.232	699.99	0.31	13.272	471.93	0.26	13.271	330.73	0.24
13.252	786.93	0.33	13.252	700.53	0.31	13.292	472.94	0.26	13.291	331.51	0.24
13.272	787.24	0.33	13.272	701.12	0.31	13.312	473.90	0.26	13.311	332.30	0.24
13.291	787.58	0.33	13.292	701.69	0.31	13.332	475.01	0.26	13.331	333.11	0.24
13.311	787.92	0.33	13.312	702.20	0.31	13.352	476.27	0.26	13.351	333.90	0.24
13.331	788.24	0.33	13.332	702.69	0.31	13.372	477.44	0.26	13.370	334.68	0.24

13.351	788.57	0.33	13.352	703.21	0.31	13.392	478.56	0.26	13.390	335.46	0.24
13.371	788.85	0.33	13.371	703.77	0.31	13.412	479.74	0.26	13.410	336.30	0.24
13.391	789.14	0.33	13.391	704.28	0.31	13.431	480.91	0.26	13.430	337.09	0.24
13.411	789.50	0.33	13.411	704.84	0.31	13.451	482.10	0.26	13.450	337.86	0.24
13.431	789.82	0.33	13.431	705.38	0.31	13.471	483.26	0.26	13.470	338.64	0.24
13.451	790.11	0.33	13.451	705.91	0.31	13.491	484.40	0.26	13.490	339.45	0.24
13.471	790.42	0.33	13.471	706.45	0.31	13.511	485.52	0.26	13.510	340.23	0.24
13.491	790.72	0.33	13.491	706.99	0.31	13.531	486.59	0.26	13.530	341.02	0.24
13.510	791.02	0.33	13.511	707.51	0.31	13.551	487.75	0.26	13.550	341.83	0.24
13.530	791.30	0.33	13.531	708.03	0.31	13.571	488.88	0.26	13.570	342.66	0.24
13.550	791.59	0.33	13.551	708.49	0.31	13.591	489.96	0.26	13.590	343.47	0.24
13.570	791.91	0.33	13.571	708.98	0.31	13.611	491.06	0.26	13.610	344.27	0.24
13.590	792.20	0.33	13.590	709.51	0.31	13.631	492.09	0.26	13.630	345.07	0.24
13.610	792.43	0.33	13.610	710.06	0.31	13.651	493.14	0.26	13.650	345.82	0.24
13.630	792.69	0.33	13.630	710.53	0.31	13.671	494.28	0.26	13.670	346.57	0.24
13.650	793.00	0.33	13.650	711.03	0.31	13.691	495.35	0.26	13.689	347.37	0.24
13.670	793.26	0.33	13.670	711.54	0.31	13.711	496.41	0.26	13.709	348.22	0.24
13.690	793.59	0.33	13.690	712.05	0.31	13.731	497.50	0.26	13.729	348.96	0.24
13.709	793.93	0.33	13.710	712.53	0.31	13.750	498.56	0.26	13.749	349.77	0.24
13.729	794.22	0.33	13.730	713.01	0.31	13.770	499.58	0.27	13.769	350.60	0.24
13.749	794.50	0.33	13.750	713.49	0.31	13.790	500.56	0.27	13.789	351.40	0.24
13.769	794.82	0.33	13.770	713.96	0.31	13.810	501.62	0.27	13.809	352.17	0.24
13.789	795.09	0.33	13.789	714.43	0.31	13.830	502.66	0.27	13.829	353.00	0.24
13.809	795.36	0.33	13.809	714.90	0.31	13.850	503.66	0.27	13.849	353.82	0.24
13.829	795.68	0.33	13.829	715.36	0.31	13.870	504.67	0.27	13.869	354.62	0.24
13.849	796.01	0.33	13.849	715.79	0.31	13.890	505.66	0.27	13.889	355.41	0.24
13.869	796.30	0.33	13.869	716.23	0.31	13.910	506.61	0.27	13.909	356.24	0.24
13.889	796.64	0.33	13.889	716.67	0.31	13.930	507.53	0.27	13.929	357.02	0.24
13.909	796.91	0.33	13.909	717.09	0.31	13.950	508.44	0.27	13.949	357.83	0.24
13.928	797.22	0.33	13.929	717.52	0.31	13.970	509.52	0.27	13.969	358.66	0.24
13.948	797.52	0.33	13.949	717.92	0.31	13.990	510.47	0.27	13.989	359.47	0.24
13.968	797.81	0.33	13.969	718.39	0.31	14.010	511.49	0.27	14.009	360.25	0.24
13.988	798.12	0.33	13.989	718.84	0.31	14.030	512.54	0.27	14.028	361.08	0.24
14.008	798.38	0.33	14.008	719.30	0.31	14.050	513.56	0.27	14.048	361.87	0.25
14.028	798.69	0.33	14.028	719.81	0.31	14.070	514.56	0.27	14.068	362.65	0.25
14.048	798.98	0.33	14.048	720.32	0.31	14.089	515.59	0.27	14.088	363.45	0.25
14.068	799.25	0.33	14.068	720.81	0.31	14.109	516.61	0.27	14.108	364.28	0.25
14.088	799.56	0.33	14.088	721.28	0.31	14.129	517.61	0.27	14.128	365.11	0.25
14.108	799.87	0.33	14.108	721.74	0.31	14.149	518.59	0.27	14.148	365.91	0.25
14.128	800.13	0.33	14.128	722.16	0.31	14.169	519.58	0.27	14.168	366.67	0.25
14.147	800.39	0.33	14.148	722.59	0.31	14.189	520.52	0.27	14.188	367.47	0.25
14.167	800.69	0.33	14.168	723.03	0.31	14.209	521.40	0.27	14.208	368.29	0.25
14.187	800.97	0.33	14.188	723.46	0.31	14.229	522.35	0.27	14.228	369.10	0.25
14.207	801.25	0.33	14.207	723.96	0.31	14.249	523.34	0.27	14.248	369.85	0.25
14.227	801.52	0.33	14.227	724.39	0.31	14.269	524.35	0.27	14.268	370.67	0.25

14.247	801.81	0.33	14.247	724.82	0.31	14.289	525.28	0.27	14.288	371.46	0.25
14.267	802.09	0.33	14.267	725.26	0.31	14.309	526.20	0.27	14.308	372.22	0.25
14.287	802.36	0.33	14.287	725.74	0.31	14.329	527.12	0.27	14.328	373.04	0.25
14.307	802.68	0.33	14.307	726.15	0.31	14.349	528.05	0.27	14.347	373.88	0.25
14.327	802.97	0.33	14.327	726.54	0.31	14.369	528.98	0.27	14.367	374.68	0.25
14.347	803.24	0.33	14.347	726.96	0.31	14.389	529.89	0.27	14.387	375.42	0.25
14.366	803.50	0.33	14.367	727.38	0.31	14.408	530.72	0.27	14.407	376.25	0.25
14.386	803.76	0.33	14.387	727.80	0.31	14.428	531.55	0.27	14.427	377.06	0.25
14.406	804.02	0.33	14.407	728.26	0.31	14.448	532.36	0.27	14.447	377.85	0.25
14.426	804.27	0.33	14.426	728.68	0.31	14.468	533.25	0.27	14.467	378.60	0.25
14.446	804.54	0.33	14.446	729.09	0.31	14.488	534.24	0.27	14.487	379.38	0.25
14.466	804.85	0.33	14.466	729.48	0.31	14.508	535.18	0.27	14.507	380.17	0.25
14.486	805.12	0.33	14.486	729.87	0.31	14.528	536.10	0.27	14.527	380.95	0.25
14.506	805.36	0.33	14.506	730.29	0.31	14.548	537.03	0.27	14.547	381.78	0.25
14.526	805.66	0.33	14.526	730.65	0.31	14.568	538.02	0.27	14.567	382.60	0.25
14.546	805.93	0.33	14.546	730.96	0.31	14.588	538.85	0.27	14.587	383.40	0.25
14.565	806.17	0.33	14.566	731.37	0.31	14.608	539.75	0.27	14.607	384.19	0.25
14.585	806.40	0.33	14.586	731.81	0.31	14.628	540.66	0.27	14.627	384.98	0.25
14.605	806.63	0.33	14.606	732.19	0.31	14.648	541.54	0.27	14.647	385.80	0.25
14.625	806.90	0.33	14.626	732.60	0.32	14.668	542.38	0.27	14.667	386.59	0.25
14.645	807.17	0.33	14.645	733.05	0.32	14.688	543.27	0.27	14.686	387.38	0.25
14.665	807.33	0.33	14.665	733.42	0.32	14.708	544.14	0.27	14.706	388.15	0.25
14.685	807.58	0.33	14.685	733.85	0.32	14.727	544.98	0.27	14.726	388.96	0.25
14.705	807.87	0.33	14.705	734.24	0.32	14.747	545.82	0.27	14.746	389.73	0.25
14.725	808.16	0.33	14.725	734.64	0.32	14.767	546.67	0.27	14.766	390.53	0.25
14.745	808.43	0.33	14.745	735.04	0.32	14.787	547.54	0.27	14.786	391.36	0.25
14.765	808.65	0.33	14.765	735.45	0.32	14.807	548.40	0.27	14.806	392.13	0.25
14.784	808.93	0.33	14.785	735.90	0.32	14.827	549.27	0.28	14.826	392.86	0.25
14.804	809.20	0.33	14.805	736.33	0.32	14.847	550.11	0.28	14.846	393.63	0.25
14.824	809.47	0.33	14.825	736.69	0.32	14.867	550.91	0.28	14.866	394.49	0.25
14.844	809.75	0.33	14.844	737.05	0.32	14.887	551.75	0.28	14.886	395.28	0.25
14.864	810.02	0.33	14.864	737.47	0.32	14.907	552.56	0.28	14.906	396.08	0.25
14.884	810.29	0.33	14.884	737.87	0.32	14.927	553.28	0.28	14.926	396.83	0.25
14.904	810.55	0.33	14.904	738.27	0.32	14.947	554.08	0.28	14.946	397.64	0.25
14.924	810.81	0.33	14.924	738.66	0.32	14.967	554.90	0.28	14.966	398.48	0.25
14.944	811.07	0.33	14.944	739.00	0.32	14.987	555.72	0.28	14.986	399.29	0.25
14.964	811.33	0.33	14.964	739.38	0.32	15.007	556.55	0.28	15.005	400.09	0.25
14.984	811.59	0.33	14.984	739.80	0.32	15.027	557.43	0.28	15.025	400.84	0.25
15.003	811.84	0.33	15.004	740.17	0.32	15.046	558.23	0.28	15.045	401.59	0.25
15.023	812.09	0.33	15.024	740.53	0.32	15.066	559.08	0.28	15.065	402.39	0.25
15.043	812.36	0.33	15.044	740.90	0.32	15.086	559.90	0.28	15.085	403.20	0.25
15.063	812.65	0.33	15.063	741.24	0.32	15.106	560.70	0.28	15.105	403.95	0.25
15.083	812.90	0.33	15.083	741.58	0.32	15.126	561.53	0.28	15.125	404.74	0.25
15.103	813.14	0.33	15.103	741.92	0.32	15.146	562.34	0.28	15.145	405.56	0.25
15.123	813.37	0.33	15.123	742.30	0.32	15.166	563.15	0.28	15.165	406.34	0.25

15.143	813.60	0.33	15.143	742.67	0.32	15.186	563.95	0.28	15.185	407.13	0.25
15.163	813.89	0.33	15.163	743.06	0.32	15.206	564.73	0.28	15.205	407.89	0.25
15.183	814.14	0.33	15.183	743.45	0.32	15.226	565.52	0.28	15.225	408.66	0.25
15.202	814.35	0.33	15.203	743.82	0.32	15.246	566.30	0.28	15.245	409.45	0.25
15.222	814.63	0.33	15.223	744.22	0.32	15.266	567.07	0.28	15.265	410.25	0.25
15.242	814.87	0.33	15.243	744.55	0.32	15.286	567.85	0.28	15.285	411.03	0.25
15.262	815.08	0.33	15.262	744.94	0.32	15.306	568.65	0.28	15.305	411.80	0.25
15.282	815.34	0.33	15.282	745.33	0.32	15.326	569.42	0.28	15.325	412.58	0.25
15.302	815.61	0.33	15.302	745.70	0.32	15.346	570.16	0.28	15.344	413.36	0.25
15.322	815.88	0.33	15.322	746.03	0.32	15.365	570.94	0.28	15.364	414.13	0.25
15.342	816.09	0.33	15.342	746.41	0.32	15.385	571.70	0.28	15.384	414.88	0.25
15.362	816.32	0.33	15.362	746.79	0.32	15.405	572.46	0.28	15.404	415.64	0.25
15.382	816.58	0.33	15.382	747.18	0.32	15.425	573.20	0.28	15.424	416.40	0.25
15.402	816.84	0.33	15.402	747.54	0.32	15.445	573.93	0.28	15.444	417.17	0.25
15.421	817.08	0.33	15.422	747.84	0.32	15.465	574.65	0.28	15.464	417.97	0.25
15.441	817.28	0.33	15.442	748.21	0.32	15.485	575.37	0.28	15.484	418.70	0.25
15.461	817.50	0.33	15.462	748.59	0.32	15.505	576.08	0.28	15.504	419.47	0.25
15.481	817.74	0.33	15.481	748.95	0.32	15.525	576.79	0.28	15.524	420.25	0.25
15.501	817.98	0.34	15.501	749.31	0.32	15.545	577.51	0.28	15.544	421.03	0.25
15.521	818.23	0.34	15.521	749.66	0.32	15.565	578.22	0.28	15.564	421.83	0.25
15.541	818.47	0.34	15.541	750.01	0.32	15.585	578.90	0.28	15.584	422.56	0.25
15.561	818.71	0.34	15.561	750.36	0.32	15.605	579.62	0.28	15.604	423.33	0.25
15.581	818.95	0.34	15.581	750.70	0.32	15.625	580.34	0.28	15.624	424.12	0.25
15.601	819.18	0.34	15.601	751.04	0.32	15.645	581.00	0.28	15.644	424.91	0.25
15.621	819.41	0.34	15.621	751.38	0.32	15.665	581.67	0.28	15.664	425.61	0.25
15.640	819.64	0.34	15.641	751.70	0.32	15.684	582.35	0.28	15.683	426.37	0.25
15.660	819.85	0.34	15.661	752.07	0.32	15.704	582.99	0.28	15.703	427.14	0.25
15.680	820.02	0.34	15.680	752.43	0.32	15.724	583.59	0.28	15.723	427.91	0.25
15.700	820.27	0.34	15.700	752.74	0.32	15.744	584.23	0.28	15.743	428.68	0.25
15.720	820.50	0.34	15.720	753.05	0.32	15.764	584.93	0.28	15.763	429.44	0.26
15.740	820.74	0.34	15.740	753.36	0.32	15.784	585.69	0.28	15.783	430.20	0.26
15.760	821.00	0.34	15.760	753.65	0.32	15.804	586.40	0.28	15.803	430.93	0.26
15.780	821.20	0.34	15.780	753.96	0.32	15.824	587.08	0.28	15.823	431.67	0.26
15.800	821.48	0.34	15.800	754.31	0.32	15.844	587.76	0.28	15.843	432.45	0.26
15.820	821.69	0.34	15.820	754.66	0.32	15.864	588.42	0.28	15.863	433.24	0.26
15.840	821.95	0.34	15.840	755.01	0.32	15.884	589.07	0.28	15.883	433.97	0.26
15.859	822.19	0.34	15.860	755.35	0.32	15.904	589.80	0.28	15.903	434.68	0.26
15.879	822.40	0.34	15.880	755.67	0.32	15.924	590.47	0.28	15.923	435.39	0.26
15.899	822.61	0.34	15.899	756.00	0.32	15.944	591.15	0.28	15.943	436.11	0.26
15.919	822.88	0.34	15.919	756.35	0.32	15.964	591.84	0.28	15.963	436.86	0.26
15.939	823.14	0.34	15.939	756.73	0.32	15.984	592.52	0.28	15.983	437.60	0.26
15.959	823.36	0.34	15.959	757.06	0.32	16.003	593.22	0.28	16.002	438.38	0.26
15.979	823.59	0.34	15.979	757.34	0.32	16.023	593.88	0.28	16.022	439.14	0.26
15.999	823.84	0.34	15.999	757.65	0.32	16.043	594.53	0.28	16.042	439.85	0.26
16.019	824.04	0.34	16.019	757.99	0.32	16.063	595.19	0.28	16.062	440.57	0.26

16.039	824.30	0.34	16.039	758.33	0.32	16.083	595.87	0.28	16.082	441.31	0.26
16.058	824.51	0.34	16.059	758.66	0.32	16.103	596.52	0.29	16.102	442.05	0.26
16.078	824.74	0.34	16.079	759.00	0.32	16.123	597.16	0.29	16.122	442.79	0.26
16.098	824.99	0.34	16.099	759.34	0.32	16.143	597.79	0.29	16.142	443.57	0.26
16.118	825.25	0.34	16.118	759.67	0.32	16.163	598.42	0.29	16.162	444.29	0.26
16.138	825.44	0.34	16.138	759.99	0.32	16.183	599.08	0.29	16.182	445.00	0.26
16.158	825.68	0.34	16.158	760.31	0.32	16.203	599.74	0.29	16.202	445.73	0.26
16.178	825.90	0.34	16.178	760.63	0.32	16.223	600.36	0.29	16.222	446.45	0.26
16.198	826.09	0.34	16.198	760.95	0.32	16.243	600.99	0.29	16.242	447.21	0.26
16.218	826.33	0.34	16.218	761.26	0.32	16.263	601.61	0.29	16.262	447.93	0.26
16.238	826.57	0.34	16.238	761.57	0.32	16.283	602.21	0.29	16.282	448.63	0.26
16.258	826.81	0.34	16.258	761.87	0.32	16.303	602.82	0.29	16.302	449.34	0.26
16.277	827.04	0.34	16.278	762.22	0.32	16.323	603.44	0.29	16.322	450.06	0.26
16.297	827.27	0.34	16.298	762.56	0.32	16.342	604.05	0.29	16.341	450.79	0.26
16.317	827.49	0.34	16.317	762.86	0.32	16.362	604.69	0.29	16.361	451.51	0.26
16.337	827.72	0.34	16.337	763.20	0.32	16.382	605.33	0.29	16.381	452.24	0.26
16.357	827.94	0.34	16.357	763.50	0.32	16.402	605.91	0.29	16.401	453.00	0.26
16.377	828.17	0.34	16.377	763.78	0.32	16.422	606.51	0.29	16.421	453.78	0.26
16.397	828.39	0.34	16.397	764.12	0.32	16.442	607.13	0.29	16.441	454.48	0.26
16.417	828.60	0.34	16.417	764.43	0.32	16.462	607.69	0.29	16.461	455.16	0.26
16.437	828.82	0.34	16.437	764.74	0.32	16.482	608.26	0.29	16.481	455.87	0.26
16.457	829.04	0.34	16.457	765.05	0.32	16.502	608.82	0.29	16.501	456.61	0.26
16.477	829.25	0.34	16.477	765.35	0.32	16.522	609.37	0.29	16.521	457.39	0.26
16.496	829.46	0.34	16.497	765.65	0.32	16.542	609.90	0.29	16.541	458.08	0.26
16.516	829.67	0.34	16.517	765.88	0.32	16.562	610.48	0.29	16.561	458.75	0.26
16.536	829.88	0.34	16.536	766.12	0.32	16.582	611.10	0.29	16.581	459.45	0.26
16.556	830.08	0.34	16.556	766.46	0.32	16.602	611.69	0.29	16.601	460.20	0.26
16.576	830.28	0.34	16.576	766.74	0.32	16.622	612.26	0.29	16.621	460.88	0.26
16.596	830.51	0.34	16.596	767.06	0.32	16.642	612.87	0.29	16.641	461.61	0.26
16.616	830.75	0.34	16.616	767.39	0.32	16.661	613.43	0.29	16.660	462.32	0.26
16.636	830.94	0.34	16.636	767.71	0.32	16.681	614.04	0.29	16.680	463.00	0.26
16.656	831.14	0.34	16.656	767.98	0.32	16.701	614.66	0.29	16.700	463.72	0.26
16.676	831.36	0.34	16.676	768.30	0.32	16.721	615.22	0.29	16.720	464.45	0.26
16.695	831.57	0.34	16.696	768.58	0.32	16.741	615.79	0.29	16.740	465.10	0.26
16.715	831.74	0.34	16.716	768.88	0.32	16.761	616.38	0.29	16.760	465.73	0.26
16.735	831.92	0.34	16.735	769.20	0.32	16.781	616.95	0.29	16.780	466.48	0.26
16.755	832.11	0.34	16.755	769.52	0.32	16.801	617.49	0.29	16.800	467.19	0.26
16.775	832.32	0.34	16.775	769.77	0.32	16.821	618.08	0.29	16.820	467.88	0.26
16.795	832.58	0.34	16.795	770.07	0.32	16.841	618.68	0.29	16.840	468.57	0.26
16.815	832.78	0.34	16.815	770.37	0.32	16.861	619.24	0.29	16.860	469.28	0.26
16.835	832.98	0.34	16.835	770.67	0.32	16.881	619.76	0.29	16.880	469.98	0.26
16.855	833.18	0.34	16.855	770.97	0.32	16.901	620.37	0.29	16.900	470.67	0.26
16.875	833.39	0.34	16.875	771.26	0.32	16.921	620.94	0.29	16.920	471.41	0.26
16.895	833.65	0.34	16.895	771.55	0.32	16.941	621.47	0.29	16.940	472.12	0.26
16.914	833.85	0.34	16.915	771.84	0.32	16.961	622.02	0.29	16.960	472.79	0.26

16.934	834.06	0.34	16.935	772.12	0.32	16.980	622.56	0.29	16.980	473.42	0.26
16.954	834.26	0.34	16.954	772.40	0.32	17.000	623.09	0.29	16.999	474.18	0.26
16.974	834.45	0.34	16.974	772.68	0.32	17.020	623.63	0.29	17.019	474.86	0.26
16.994	834.65	0.34	16.994	773.01	0.32	17.040	624.15	0.29	17.039	475.50	0.26
17.014	834.85	0.34	17.014	773.30	0.32	17.060	624.67	0.29	17.059	476.15	0.26
17.034	835.10	0.34	17.034	773.57	0.32	17.080	625.22	0.29	17.079	476.84	0.26
17.054	835.32	0.34	17.054	773.84	0.32	17.100	625.76	0.29	17.099	477.57	0.26
17.074	835.52	0.34	17.074	774.08	0.32	17.120	626.27	0.29	17.119	478.27	0.26
17.094	835.77	0.34	17.094	774.29	0.32	17.140	626.77	0.29	17.139	478.92	0.26
17.114	835.96	0.34	17.114	774.58	0.33	17.160	627.27	0.29	17.159	479.56	0.26
17.133	836.14	0.34	17.134	774.92	0.33	17.180	627.72	0.29	17.179	480.17	0.26
17.153	836.33	0.34	17.154	775.23	0.33	17.200	628.23	0.29	17.199	480.81	0.26
17.173	836.57	0.34	17.173	775.51	0.33	17.220	628.80	0.29	17.219	481.50	0.26
17.193	836.76	0.34	17.193	775.78	0.33	17.240	629.37	0.29	17.239	482.20	0.26
17.213	836.97	0.34	17.213	776.05	0.33	17.260	629.91	0.29	17.259	482.93	0.26
17.233	837.19	0.34	17.233	776.33	0.33	17.280	630.44	0.29	17.279	483.59	0.26
17.253	837.36	0.34	17.253	776.60	0.33	17.299	630.96	0.29	17.299	484.26	0.26
17.273	837.58	0.34	17.273	776.88	0.33	17.319	631.48	0.29	17.318	484.94	0.26
17.293	837.79	0.34	17.293	777.20	0.33	17.339	632.00	0.29	17.338	485.63	0.26
17.313	837.96	0.34	17.313	777.51	0.33	17.359	632.53	0.29	17.358	486.24	0.26
17.333	838.17	0.34	17.333	777.77	0.33	17.379	633.04	0.29	17.378	486.94	0.26
17.352	838.39	0.34	17.353	778.03	0.33	17.399	633.57	0.29	17.398	487.60	0.27
17.372	838.61	0.34	17.372	778.29	0.33	17.419	634.10	0.29	17.418	488.24	0.27
17.392	838.83	0.34	17.392	778.59	0.33	17.439	634.63	0.29	17.438	488.90	0.27
17.412	839.02	0.34	17.412	778.88	0.33	17.459	635.16	0.29	17.458	489.59	0.27
17.432	839.17	0.34	17.432	779.13	0.33	17.479	635.64	0.29	17.478	490.21	0.27
17.452	839.37	0.34	17.452	779.39	0.33	17.499	636.11	0.29	17.498	490.84	0.27
17.472	839.57	0.34	17.472	779.70	0.33	17.519	636.61	0.29	17.518	491.51	0.27
17.492	839.76	0.34	17.492	779.98	0.33	17.539	637.16	0.29	17.538	492.16	0.27
17.512	839.96	0.34	17.512	780.23	0.33	17.559	637.63	0.29	17.558	492.80	0.27
17.532	840.15	0.34	17.532	780.48	0.33	17.579	638.15	0.29	17.578	493.44	0.27
17.551	840.34	0.34	17.552	780.79	0.33	17.599	638.67	0.29	17.598	494.12	0.27
17.571	840.53	0.34	17.572	781.05	0.33	17.618	639.13	0.29	17.618	494.77	0.27
17.591	840.76	0.34	17.591	781.29	0.33	17.638	639.65	0.29	17.638	495.41	0.27
17.611	840.97	0.34	17.611	781.54	0.33	17.658	640.14	0.29	17.657	496.02	0.27
17.631	841.14	0.34	17.631	781.85	0.33	17.678	640.59	0.29	17.677	496.61	0.27
17.651	841.31	0.34	17.651	782.12	0.33	17.698	641.08	0.29	17.697	497.24	0.27
17.671	841.45	0.34	17.671	782.37	0.33	17.718	641.52	0.29	17.717	497.89	0.27
17.691	841.63	0.34	17.691	782.66	0.33	17.738	641.97	0.30	17.737	498.54	0.27
17.711	841.82	0.34	17.711	782.89	0.33	17.758	642.51	0.30	17.757	499.20	0.27
17.731	842.02	0.34	17.731	783.14	0.33	17.778	642.96	0.30	17.777	499.81	0.27
17.751	842.27	0.34	17.751	783.41	0.33	17.798	643.45	0.30	17.797	500.44	0.27
17.770	842.47	0.34	17.771	783.69	0.33	17.818	643.96	0.30	17.817	501.08	0.27
17.790	842.65	0.34	17.790	783.96	0.33	17.838	644.46	0.30	17.837	501.70	0.27
17.810	842.83	0.34	17.810	784.24	0.33	17.858	644.93	0.30	17.857	502.33	0.27

17.830	843.01	0.34	17.830	784.45	0.33	17.878	645.38	0.30	17.877	502.96	0.27
17.850	843.24	0.34	17.850	784.67	0.33	17.898	645.83	0.30	17.897	503.60	0.27
17.870	843.45	0.34	17.870	784.93	0.33	17.918	646.35	0.30	17.917	504.21	0.27
17.890	843.63	0.34	17.890	785.20	0.33	17.937	646.87	0.30	17.937	504.81	0.27
17.910	843.81	0.34	17.910	785.47	0.33	17.957	647.33	0.30	17.957	505.47	0.27
17.930	844.00	0.34	17.930	785.73	0.33	17.977	647.83	0.30	17.976	506.13	0.27
17.950	844.19	0.34	17.950	785.98	0.33	17.997	648.28	0.30	17.996	506.75	0.27
17.970	844.38	0.34	17.970	786.22	0.33	18.017	648.73	0.30	18.016	507.35	0.27
17.989	844.57	0.34	17.990	786.48	0.33	18.037	649.19	0.30	18.036	508.00	0.27
18.009	844.76	0.34	18.009	786.73	0.33	18.057	649.69	0.30	18.056	508.59	0.27
18.029	844.94	0.34	18.029	786.98	0.33	18.077	650.15	0.30	18.076	509.21	0.27
18.049	845.14	0.34	18.049	787.24	0.33	18.097	650.62	0.30	18.096	509.85	0.27
18.069	845.37	0.34	18.069	787.50	0.33	18.117	651.07	0.30	18.116	510.41	0.27
18.089	845.55	0.34	18.089	787.77	0.33	18.137	651.50	0.30	18.136	511.04	0.27
18.109	845.72	0.34	18.109	788.03	0.33	18.157	651.95	0.30	18.156	511.69	0.27
18.129	845.89	0.34	18.129	788.30	0.33	18.177	652.44	0.30	18.176	512.29	0.27
18.149	846.11	0.34	18.149	788.56	0.33	18.197	652.90	0.30	18.196	512.84	0.27
18.169	846.30	0.34	18.169	788.82	0.33	18.217	653.35	0.30	18.216	513.41	0.27
18.188	846.46	0.34	18.189	789.06	0.33	18.237	653.85	0.30	18.236	514.04	0.27
18.208	846.67	0.34	18.209	789.30	0.33	18.256	654.27	0.30	18.256	514.57	0.27
18.228	846.85	0.34	18.228	789.55	0.33	18.276	654.77	0.30	18.276	515.18	0.27
18.248	847.00	0.34	18.248	789.79	0.33	18.296	655.32	0.30	18.296	515.79	0.27
18.268	847.20	0.34	18.268	790.02	0.33	18.316	655.76	0.30	18.315	516.36	0.27
18.288	847.41	0.34	18.288	790.29	0.33	18.336	656.16	0.30	18.335	516.96	0.27
18.308	847.61	0.34	18.308	790.57	0.33	18.356	656.57	0.30	18.355	517.57	0.27
18.328	847.77	0.34	18.328	790.81	0.33	18.376	657.06	0.30	18.375	518.17	0.27
18.348	847.92	0.34	18.348	791.04	0.33	18.396	657.50	0.30	18.395	518.75	0.27
18.368	848.11	0.34	18.368	791.25	0.33	18.416	657.93	0.30	18.415	519.32	0.27
18.388	848.30	0.34	18.388	791.47	0.33	18.436	658.39	0.30	18.435	519.96	0.27
18.407	848.49	0.34	18.408	791.76	0.33	18.456	658.81	0.30	18.455	520.56	0.27
18.427	848.67	0.34	18.427	791.97	0.33	18.476	659.20	0.30	18.475	521.14	0.27
18.447	848.85	0.34	18.447	792.25	0.33	18.496	659.57	0.30	18.495	521.72	0.27
18.467	849.03	0.34	18.467	792.49	0.33	18.516	659.99	0.30	18.515	522.32	0.27
18.487	849.22	0.34	18.487	792.75	0.33	18.536	660.43	0.30	18.535	522.88	0.27
18.507	849.40	0.34	18.507	793.03	0.33	18.556	660.88	0.30	18.555	523.44	0.27
18.527	849.53	0.34	18.527	793.30	0.33	18.576	661.32	0.30	18.575	524.01	0.27
18.547	849.75	0.34	18.547	793.57	0.33	18.595	661.71	0.30	18.595	524.63	0.27
18.567	849.97	0.34	18.567	793.83	0.33	18.615	662.13	0.30	18.615	525.25	0.27
18.587	850.15	0.34	18.587	794.02	0.33	18.635	662.58	0.30	18.634	525.80	0.27
18.607	850.32	0.34	18.607	794.26	0.33	18.655	663.02	0.30	18.654	526.34	0.27
18.626	850.50	0.34	18.627	794.52	0.33	18.675	663.46	0.30	18.674	526.93	0.27
18.646	850.68	0.34	18.646	794.79	0.33	18.695	663.89	0.30	18.694	527.51	0.27
18.666	850.86	0.34	18.666	795.04	0.33	18.715	664.32	0.30	18.714	528.08	0.27
18.686	851.03	0.34	18.686	795.30	0.33	18.735	664.75	0.30	18.734	528.65	0.27
18.706	851.19	0.34	18.706	795.51	0.33	18.755	665.18	0.30	18.754	529.22	0.27

18.726	851.36	0.34	18.726	795.73	0.33	18.775	665.59	0.30	18.774	529.77	0.27
18.746	851.54	0.34	18.746	795.99	0.33	18.795	666.00	0.30	18.794	530.33	0.27
18.766	851.73	0.34	18.766	796.25	0.33	18.815	666.40	0.30	18.814	530.90	0.27
18.786	851.91	0.34	18.786	796.50	0.33	18.835	666.80	0.30	18.834	531.41	0.27
18.806	852.09	0.34	18.806	796.72	0.33	18.855	667.19	0.30	18.854	531.97	0.27
18.826	852.27	0.34	18.826	796.93	0.33	18.875	667.58	0.30	18.874	532.55	0.27
18.845	852.45	0.34	18.845	797.18	0.33	18.895	668.03	0.30	18.894	533.09	0.27
18.865	852.63	0.34	18.865	797.42	0.33	18.914	668.43	0.30	18.914	533.66	0.27
18.885	852.80	0.34	18.885	797.67	0.33	18.934	668.82	0.30	18.934	534.22	0.27
18.905	852.98	0.34	18.905	797.91	0.33	18.954	669.23	0.30	18.954	534.80	0.27
18.925	853.18	0.34	18.925	798.16	0.33	18.974	669.64	0.30	18.973	535.35	0.27
18.945	853.40	0.34	18.945	798.34	0.33	18.994	670.05	0.30	18.993	535.89	0.27
18.965	853.56	0.34	18.965	798.56	0.33	19.014	670.42	0.30	19.013	536.45	0.27
18.985	853.73	0.34	18.985	798.80	0.33	19.034	670.77	0.30	19.033	537.06	0.27
19.005	853.89	0.34	19.005	799.04	0.33	19.054	671.11	0.30	19.053	537.63	0.27
19.025	854.04	0.34	19.025	799.27	0.33	19.074	671.56	0.30	19.073	538.17	0.27
19.044	854.22	0.34	19.045	799.51	0.33	19.094	671.97	0.30	19.093	538.70	0.27
19.064	854.43	0.34	19.064	799.74	0.33	19.114	672.35	0.30	19.113	539.24	0.27
19.084	854.58	0.34	19.084	799.98	0.33	19.134	672.73	0.30	19.133	539.79	0.27
19.104	854.74	0.34	19.104	800.21	0.33	19.154	673.13	0.30	19.153	540.35	0.28
19.124	854.91	0.34	19.124	800.44	0.33	19.174	673.54	0.30	19.173	540.92	0.28
19.144	855.10	0.34	19.144	800.66	0.33	19.194	673.93	0.30	19.193	541.45	0.28
19.164	855.28	0.34	19.164	800.89	0.33	19.214	674.36	0.30	19.213	541.98	0.28
19.184	855.45	0.34	19.184	801.11	0.33	19.233	674.77	0.30	19.233	542.52	0.28
19.204	855.58	0.34	19.204	801.33	0.33	19.253	675.13	0.30	19.253	543.06	0.28
19.224	855.81	0.34	19.224	801.55	0.33	19.273	675.53	0.30	19.273	543.62	0.28
19.244	856.00	0.34	19.244	801.77	0.33	19.293	675.91	0.30	19.292	544.14	0.28
19.263	856.17	0.34	19.263	802.00	0.33	19.313	676.28	0.30	19.312	544.65	0.28
19.283	856.34	0.34	19.283	802.22	0.33	19.333	676.67	0.30	19.332	545.20	0.28
19.303	856.51	0.34	19.303	802.42	0.33	19.353	677.04	0.30	19.352	545.75	0.28
19.323	856.67	0.34	19.323	802.65	0.33	19.373	677.43	0.30	19.372	546.28	0.28
19.343	856.83	0.34	19.343	802.91	0.33	19.393	677.84	0.30	19.392	546.80	0.28
19.363	857.03	0.34	19.363	803.12	0.33	19.413	678.18	0.30	19.412	547.28	0.28
19.383	857.24	0.34	19.383	803.38	0.33	19.433	678.55	0.30	19.432	547.78	0.28
19.403	857.39	0.34	19.403	803.59	0.33	19.453	678.93	0.30	19.452	548.31	0.28
19.423	857.55	0.34	19.423	803.79	0.33	19.473	679.27	0.30	19.472	548.88	0.28
19.443	857.70	0.34	19.443	804.00	0.33	19.493	679.69	0.30	19.492	549.41	0.28
19.463	857.90	0.34	19.463	804.27	0.33	19.513	680.04	0.30	19.512	549.92	0.28
19.482	858.08	0.34	19.482	804.46	0.33	19.533	680.43	0.30	19.532	550.42	0.28
19.502	858.23	0.34	19.502	804.67	0.33	19.552	680.81	0.30	19.552	550.93	0.28
19.522	858.41	0.34	19.522	804.91	0.33	19.572	681.18	0.30	19.572	551.47	0.28
19.542	858.60	0.34	19.542	805.15	0.33	19.592	681.60	0.30	19.592	552.02	0.28
19.562	858.74	0.34	19.562	805.36	0.33	19.612	682.01	0.30	19.612	552.51	0.28
19.582	858.90	0.34	19.582	805.55	0.33	19.632	682.36	0.30	19.631	553.02	0.28
19.602	859.08	0.35	19.602	805.81	0.33	19.652	682.74	0.30	19.651	553.53	0.28

19.622	859.22	0.35	19.622	806.02	0.33	19.672	683.14	0.30	19.671	554.00	0.28
19.642	859.30	0.35	19.642	806.23	0.33	19.692	683.52	0.30	19.691	554.46	0.28
19.662	859.49	0.35	19.662	806.48	0.33	19.712	683.90	0.30	19.711	554.93	0.28
19.681	859.66	0.35	19.682	806.72	0.33	19.732	684.28	0.30	19.731	555.45	0.28
19.701	859.83	0.35	19.701	806.95	0.33	19.752	684.67	0.30	19.751	555.99	0.28
19.721	860.02	0.35	19.721	807.16	0.33	19.772	685.04	0.30	19.771	556.48	0.28
19.741	860.17	0.35	19.741	807.34	0.33	19.792	685.39	0.30	19.791	557.00	0.28
19.761	860.36	0.35	19.761	807.57	0.33	19.812	685.74	0.30	19.811	557.55	0.28
19.781	860.50	0.35	19.781	807.78	0.33	19.832	686.11	0.30	19.831	558.06	0.28
19.801	860.67	0.35	19.801	807.97	0.33	19.852	686.46	0.30	19.851	558.55	0.28
19.821	860.86	0.35	19.821	808.21	0.33	19.871	686.77	0.31	19.871	559.04	0.28
19.841	861.05	0.35	19.841	808.40	0.33	19.891	687.15	0.31	19.891	559.55	0.28
19.861	861.17	0.35	19.861	808.61	0.33	19.911	687.56	0.31	19.911	560.09	0.28
19.881	861.36	0.35	19.881	808.86	0.33	19.931	687.94	0.31	19.931	560.57	0.28
19.900	861.51	0.35	19.900	809.09	0.33	19.951	688.31	0.31	19.950	561.07	0.28
19.920	861.65	0.35	19.920	809.29	0.33	19.971	688.66	0.31	19.970	561.57	0.28
19.940	861.84	0.35	19.940	809.49	0.33	19.991	689.02	0.31	19.990	562.04	0.28
19.960	862.02	0.35	19.960	809.73	0.33	20.011	689.37	0.31	20.010	562.57	0.28
19.980	862.17	0.35	19.980	809.95	0.33	20.031	689.71	0.31	20.030	563.06	0.28
20.000	862.31	0.35	20.000	810.14	0.33	20.051	690.10	0.31	20.050	563.50	0.28
						20.071	690.46	0.31	20.070	564.03	0.28
						20.091	690.80	0.31	20.090	564.53	0.28
						20.111	691.11	0.31	20.110	565.02	0.28
						20.131	691.47	0.31	20.130	565.50	0.28
						20.151	691.85	0.31	20.150	565.95	0.28
						20.171	692.17	0.31	20.170	566.39	0.28
						20.190	692.53	0.31	20.190	566.87	0.28
						20.210	692.92	0.31	20.210	567.35	0.28
						20.230	693.22	0.31	20.230	567.84	0.28
						20.250	693.53	0.31	20.250	568.32	0.28
						20.270	693.89	0.31	20.270	568.78	0.28
						20.290	694.24	0.31	20.289	569.23	0.28
						20.310	694.58	0.31	20.309	569.69	0.28
						20.330	694.85	0.31	20.329	570.16	0.28
						20.350	695.16	0.31	20.349	570.63	0.28
						20.370	695.50	0.31	20.369	571.10	0.28
						20.390	695.88	0.31	20.389	571.59	0.28
						20.410	696.19	0.31	20.409	572.11	0.28
						20.430	696.50	0.31	20.429	572.56	0.28
						20.450	696.85	0.31	20.449	573.05	0.28
						20.470	697.21	0.31	20.469	573.50	0.28
						20.490	697.51	0.31	20.489	573.96	0.28
						20.509	697.87	0.31	20.509	574.47	0.28
						20.529	698.22	0.31	20.529	574.91	0.28
						20.549	698.55	0.31	20.549	575.40	0.28

						20.569	698.92	0.31	20.569	575.84	0.28
						20.589	699.22	0.31	20.589	576.27	0.28
						20.609	699.52	0.31	20.608	576.77	0.28
						20.629	699.84	0.31	20.628	577.25	0.28
						20.649	700.21	0.31	20.648	577.71	0.28
						20.669	700.51	0.31	20.668	578.15	0.28
						20.689	700.84	0.31	20.688	578.62	0.28
						20.709	701.19	0.31	20.708	579.11	0.28
						20.729	701.49	0.31	20.728	579.54	0.28
						20.749	701.85	0.31	20.748	579.96	0.28
						20.769	702.19	0.31	20.768	580.46	0.28
						20.789	702.51	0.31	20.788	580.91	0.28
						20.809	702.83	0.31	20.808	581.36	0.28
						20.829	703.16	0.31	20.828	581.82	0.28
						20.848	703.50	0.31	20.848	582.24	0.28
						20.868	703.82	0.31	20.868	582.68	0.28
						20.888	704.14	0.31	20.888	583.13	0.28
						20.908	704.46	0.31	20.908	583.57	0.28
						20.928	704.77	0.31	20.928	584.02	0.28
						20.948	705.09	0.31	20.947	584.47	0.28
						20.968	705.40	0.31	20.967	584.92	0.28
						20.988	705.70	0.31	20.987	585.33	0.28
						21.008	705.99	0.31	21.007	585.79	0.28
						21.028	706.28	0.31	21.027	586.25	0.28
						21.048	706.55	0.31	21.047	586.71	0.28
						21.068	706.87	0.31	21.067	587.21	0.28
						21.088	707.21	0.31	21.087	587.68	0.28
						21.108	707.55	0.31	21.107	588.10	0.28
						21.128	707.87	0.31	21.127	588.53	0.28
						21.148	708.19	0.31	21.147	588.97	0.28
						21.167	708.47	0.31	21.167	589.39	0.28
						21.187	708.77	0.31	21.187	589.83	0.28
						21.207	709.10	0.31	21.207	590.26	0.29
						21.227	709.37	0.31	21.227	590.69	0.29
						21.247	709.71	0.31	21.247	591.08	0.29
						21.267	710.04	0.31	21.267	591.48	0.29
						21.287	710.36	0.31	21.286	591.90	0.29
						21.307	710.68	0.31	21.306	592.35	0.29
						21.327	710.99	0.31	21.326	592.78	0.29
						21.347	711.31	0.31	21.346	593.21	0.29
						21.367	711.61	0.31	21.366	593.65	0.29
						21.387	711.91	0.31	21.386	594.08	0.29
						21.407	712.18	0.31	21.406	594.52	0.29
						21.427	712.49	0.31	21.426	594.93	0.29
						21.447	712.82	0.31	21.446	595.34	0.29

						21.467	713.15	0.31	21.466	595.76	0.29
						21.486	713.44	0.31	21.486	596.23	0.29
						21.506	713.72	0.31	21.506	596.64	0.29
						21.526	713.99	0.31	21.526	597.05	0.29
						21.546	714.27	0.31	21.546	597.46	0.29
						21.566	714.60	0.31	21.566	597.87	0.29
						21.586	714.92	0.31	21.586	598.29	0.29
						21.606	715.22	0.31	21.605	598.70	0.29
						21.626	715.50	0.31	21.625	599.12	0.29
						21.646	715.78	0.31	21.645	599.57	0.29
						21.666	716.07	0.31	21.665	599.96	0.29
						21.686	716.35	0.31	21.685	600.34	0.29
						21.706	716.63	0.31	21.705	600.79	0.29
						21.726	716.93	0.31	21.725	601.19	0.29
						21.746	717.26	0.31	21.745	601.58	0.29
						21.766	717.53	0.31	21.765	602.02	0.29
						21.786	717.81	0.31	21.785	602.38	0.29
						21.805	718.12	0.31	21.805	602.75	0.29
						21.825	718.37	0.31	21.825	603.16	0.29
						21.845	718.61	0.31	21.845	603.59	0.29
						21.865	718.90	0.31	21.865	603.98	0.29
						21.885	719.22	0.31	21.885	604.37	0.29
						21.905	719.50	0.31	21.905	604.78	0.29
						21.925	719.80	0.31	21.925	605.20	0.29
						21.945	720.10	0.31	21.944	605.58	0.29
						21.965	720.39	0.31	21.964	606.01	0.29
						21.985	720.63	0.31	21.984	606.41	0.29
						22.005	720.93	0.31	22.004	606.80	0.29
						22.025	721.24	0.31	22.024	607.20	0.29
						22.045	721.54	0.31	22.044	607.62	0.29
						22.065	721.83	0.31	22.064	608.02	0.29
						22.085	722.11	0.31	22.084	608.41	0.29
						22.105	722.38	0.31	22.104	608.81	0.29
						22.124	722.63	0.31	22.124	609.24	0.29
						22.144	722.94	0.31	22.144	609.64	0.29
						22.164	723.22	0.31	22.164	610.01	0.29
						22.184	723.48	0.31	22.184	610.39	0.29
						22.204	723.75	0.31	22.204	610.80	0.29
						22.224	724.01	0.31	22.224	611.22	0.29
						22.244	724.34	0.31	22.244	611.59	0.29
						22.264	724.66	0.31	22.263	611.97	0.29
						22.284	724.96	0.31	22.283	612.37	0.29
						22.304	725.23	0.31	22.303	612.78	0.29
						22.324	725.50	0.31	22.323	613.15	0.29
						22.344	725.77	0.31	22.343	613.51	0.29

						22.364	726.05	0.31	22.363	613.91	0.29
						22.384	726.32	0.31	22.383	614.32	0.29
						22.404	726.57	0.31	22.403	614.72	0.29
						22.424	726.81	0.31	22.423	615.09	0.29
						22.443	727.08	0.31	22.443	615.45	0.29
						22.463	727.36	0.31	22.463	615.82	0.29
						22.483	727.63	0.31	22.483	616.21	0.29
						22.503	727.89	0.31	22.503	616.61	0.29
						22.523	728.16	0.31	22.523	617.02	0.29
						22.543	728.42	0.31	22.543	617.42	0.29
						22.563	728.70	0.31	22.563	617.78	0.29
						22.583	728.97	0.31	22.583	618.12	0.29
						22.603	729.24	0.31	22.602	618.48	0.29
						22.623	729.49	0.31	22.622	618.87	0.29
						22.643	729.75	0.31	22.642	619.27	0.29
						22.663	730.02	0.31	22.662	619.64	0.29
						22.683	730.30	0.31	22.682	620.00	0.29
						22.703	730.55	0.32	22.702	620.42	0.29
						22.723	730.82	0.32	22.722	620.83	0.29
						22.743	731.06	0.32	22.742	621.23	0.29
						22.762	731.34	0.32	22.762	621.56	0.29
						22.782	731.63	0.32	22.782	621.89	0.29
						22.802	731.91	0.32	22.802	622.21	0.29
						22.822	732.18	0.32	22.822	622.56	0.29
						22.842	732.40	0.32	22.842	622.97	0.29
						22.862	732.67	0.32	22.862	623.33	0.29
						22.882	732.95	0.32	22.882	623.68	0.29
						22.902	733.22	0.32	22.902	624.03	0.29
						22.922	733.52	0.32	22.921	624.38	0.29
						22.942	733.82	0.32	22.941	624.74	0.29
						22.962	734.06	0.32	22.961	625.13	0.29
						22.982	734.31	0.32	22.981	625.51	0.29
						23.002	734.56	0.32	23.001	625.83	0.29
						23.022	734.81	0.32	23.021	626.17	0.29
						23.042	735.08	0.32	23.041	626.53	0.29
						23.062	735.34	0.32	23.061	626.91	0.29
						23.082	735.63	0.32	23.081	627.28	0.29
						23.101	735.89	0.32	23.101	627.64	0.29
						23.121	736.13	0.32	23.121	627.99	0.29
						23.141	736.38	0.32	23.141	628.32	0.29
						23.161	736.67	0.32	23.161	628.67	0.29
						23.181	736.94	0.32	23.181	629.03	0.29
						23.201	737.18	0.32	23.201	629.35	0.29
						23.221	737.43	0.32	23.221	629.69	0.29
						23.241	737.71	0.32	23.241	630.04	0.29

						23.261	737.96	0.32	23.260	630.40	0.29
						23.281	738.17	0.32	23.280	630.75	0.29
						23.301	738.45	0.32	23.300	631.08	0.29
						23.321	738.70	0.32	23.320	631.42	0.29
						23.341	738.94	0.32	23.340	631.73	0.29
						23.361	739.22	0.32	23.360	632.03	0.29
						23.381	739.46	0.32	23.380	632.36	0.29
						23.401	739.68	0.32	23.400	632.69	0.29
						23.420	739.95	0.32	23.420	633.01	0.29
						23.440	740.23	0.32	23.440	633.36	0.29
						23.460	740.49	0.32	23.460	633.74	0.29
						23.480	740.69	0.32	23.480	634.07	0.29
						23.500	740.93	0.32	23.500	634.45	0.29
						23.520	741.19	0.32	23.520	634.80	0.29
						23.540	741.42	0.32	23.540	635.13	0.29
						23.560	741.67	0.32	23.560	635.47	0.29
						23.580	741.93	0.32	23.579	635.85	0.29
						23.600	742.21	0.32	23.599	636.16	0.29
						23.620	742.44	0.32	23.619	636.52	0.29
						23.640	742.65	0.32	23.639	636.87	0.29
						23.660	742.89	0.32	23.659	637.17	0.29
						23.680	743.14	0.32	23.679	637.54	0.29
						23.700	743.39	0.32	23.699	637.87	0.29
						23.720	743.64	0.32	23.719	638.22	0.30
						23.739	743.88	0.32	23.739	638.59	0.30
						23.759	744.04	0.32	23.759	638.93	0.30
						23.779	744.29	0.32	23.779	639.27	0.30
						23.799	744.54	0.32	23.799	639.58	0.30
						23.819	744.80	0.32	23.819	639.88	0.30
						23.839	745.06	0.32	23.839	640.25	0.30
						23.859	745.28	0.32	23.859	640.58	0.30
						23.879	745.55	0.32	23.879	640.90	0.30
						23.899	745.82	0.32	23.899	641.25	0.30
						23.919	746.02	0.32	23.918	641.58	0.30
						23.939	746.25	0.32	23.938	641.93	0.30
						23.959	746.52	0.32	23.958	642.28	0.30
						23.979	746.78	0.32	23.978	642.61	0.30
						23.999	746.99	0.32	23.998	642.89	0.30
						24.019	747.27	0.32	24.018	643.19	0.30
						24.039	747.53	0.32	24.038	643.58	0.30
						24.058	747.79	0.32	24.058	643.91	0.30
						24.078	748.03	0.32	24.078	644.22	0.30
						24.098	748.27	0.32	24.098	644.54	0.30
						24.118	748.51	0.32	24.118	644.87	0.30
						24.138	748.72	0.32	24.138	645.20	0.30

						24.158	748.92	0.32	24.158	645.53	0.30
						24.178	749.18	0.32	24.178	645.84	0.30
						24.198	749.43	0.32	24.198	646.15	0.30
						24.218	749.68	0.32	24.218	646.45	0.30
						24.238	749.92	0.32	24.237	646.76	0.30
						24.258	750.13	0.32	24.257	647.08	0.30
						24.278	750.40	0.32	24.277	647.42	0.30
						24.298	750.61	0.32	24.297	647.74	0.30
						24.318	750.85	0.32	24.317	648.05	0.30
						24.338	751.10	0.32	24.337	648.36	0.30
						24.358	751.34	0.32	24.357	648.66	0.30
						24.377	751.62	0.32	24.377	648.97	0.30
						24.397	751.85	0.32	24.397	649.28	0.30
						24.417	752.08	0.32	24.417	649.60	0.30
						24.437	752.30	0.32	24.437	649.94	0.30
						24.457	752.54	0.32	24.457	650.24	0.30
						24.477	752.78	0.32	24.477	650.55	0.30
						24.497	752.98	0.32	24.497	650.87	0.30
						24.517	753.19	0.32	24.517	651.14	0.30
						24.537	753.41	0.32	24.537	651.35	0.30
						24.557	753.66	0.32	24.557	651.65	0.30
						24.577	753.90	0.32	24.576	652.00	0.30
						24.597	754.11	0.32	24.596	652.29	0.30
						24.617	754.35	0.32	24.616	652.58	0.30
						24.637	754.59	0.32	24.636	652.88	0.30
						24.657	754.79	0.32	24.656	653.22	0.30
						24.677	755.01	0.32	24.676	653.56	0.30
						24.696	755.23	0.32	24.696	653.86	0.30
						24.716	755.44	0.32	24.716	654.21	0.30
						24.736	755.69	0.32	24.736	654.50	0.30
						24.756	755.94	0.32	24.756	654.78	0.30
						24.776	756.15	0.32	24.776	655.12	0.30
						24.796	756.39	0.32	24.796	655.42	0.30
						24.816	756.64	0.32	24.816	655.73	0.30
						24.836	756.83	0.32	24.836	656.06	0.30
						24.856	757.04	0.32	24.856	656.34	0.30
						24.876	757.27	0.32	24.876	656.63	0.30
						24.896	757.48	0.32	24.895	657.00	0.30
						24.916	757.67	0.32	24.915	657.29	0.30
						24.936	757.91	0.32	24.935	657.65	0.30
						24.956	758.14	0.32	24.955	657.93	0.30
						24.976	758.33	0.32	24.975	658.23	0.30
						24.996	758.54	0.32	24.995	658.55	0.30
						25.015	758.78	0.32	25.015	658.88	0.30
						25.035	759.01	0.32	25.035	659.15	0.30

						25.055	759.24	0.32	25.055	659.47	0.30
						25.075	759.47	0.32	25.075	659.76	0.30
						25.095	759.68	0.32	25.095	660.07	0.30
						25.115	759.87	0.32	25.115	660.38	0.30
						25.135	760.07	0.32	25.135	660.64	0.30
						25.155	760.29	0.32	25.155	660.99	0.30
						25.175	760.47	0.32	25.175	661.30	0.30
						25.195	760.65	0.32	25.195	661.58	0.30
						25.215	760.88	0.32	25.215	661.85	0.30
						25.235	761.14	0.32	25.234	662.16	0.30
						25.255	761.35	0.32	25.254	662.49	0.30
						25.275	761.55	0.32	25.274	662.80	0.30
						25.295	761.75	0.32	25.294	663.12	0.30
						25.315	761.98	0.32	25.314	663.41	0.30
						25.335	762.24	0.32	25.334	663.67	0.30
						25.354	762.44	0.32	25.354	664.01	0.30
						25.374	762.65	0.32	25.374	664.27	0.30
						25.394	762.85	0.32	25.394	664.56	0.30
						25.414	763.08	0.32	25.414	664.88	0.30
						25.434	763.33	0.32	25.434	665.20	0.30
						25.454	763.53	0.32	25.454	665.48	0.30
						25.474	763.72	0.32	25.474	665.78	0.30
						25.494	763.96	0.32	25.494	666.08	0.30
						25.514	764.17	0.32	25.514	666.40	0.30
						25.534	764.37	0.32	25.534	666.68	0.30
						25.554	764.56	0.32	25.553	666.94	0.30
						25.574	764.82	0.32	25.573	667.21	0.30
						25.594	765.02	0.32	25.593	667.51	0.30
						25.614	765.24	0.32	25.613	667.82	0.30
						25.634	765.49	0.32	25.633	668.12	0.30
						25.654	765.67	0.32	25.653	668.42	0.30
						25.673	765.86	0.32	25.673	668.69	0.30
						25.693	766.09	0.32	25.693	668.98	0.30
						25.713	766.32	0.32	25.713	669.28	0.30
						25.733	766.50	0.32	25.733	669.59	0.30
						25.753	766.74	0.32	25.753	669.88	0.30
						25.773	766.94	0.32	25.773	670.17	0.30
						25.793	767.13	0.32	25.793	670.46	0.30
						25.813	767.38	0.32	25.813	670.74	0.30
						25.833	767.61	0.32	25.833	671.03	0.30
						25.853	767.84	0.32	25.853	671.31	0.30
						25.873	768.07	0.32	25.873	671.58	0.30
						25.893	768.23	0.32	25.892	671.87	0.30
						25.913	768.44	0.32	25.912	672.15	0.30
						25.933	768.66	0.32	25.932	672.44	0.30

						25.953	768.88	0.32	25.952	672.71	0.30
						25.973	769.07	0.32	25.972	672.98	0.30
						25.992	769.26	0.32	25.992	673.25	0.30
						26.012	769.48	0.32	26.012	673.52	0.30
						26.032	769.69	0.32	26.032	673.85	0.30
						26.052	769.89	0.32	26.052	674.16	0.30
						26.072	770.10	0.32	26.072	674.41	0.30
						26.092	770.30	0.32	26.092	674.67	0.30
						26.112	770.52	0.32	26.112	674.94	0.30
						26.132	770.73	0.32	26.132	675.25	0.30
						26.152	770.90	0.32	26.152	675.56	0.30
						26.172	771.07	0.32	26.172	675.85	0.30
						26.192	771.28	0.32	26.192	676.09	0.30
						26.212	771.50	0.32	26.211	676.34	0.30
						26.232	771.71	0.32	26.231	676.58	0.30
						26.252	771.92	0.32	26.251	676.88	0.30
						26.272	772.13	0.32	26.271	677.17	0.30
						26.292	772.34	0.32	26.291	677.44	0.30
						26.311	772.54	0.32	26.311	677.72	0.30
						26.331	772.73	0.32	26.331	678.01	0.30
						26.351	772.92	0.32	26.351	678.26	0.30
						26.371	773.11	0.32	26.371	678.50	0.30
						26.391	773.30	0.32	26.391	678.80	0.30
						26.411	773.49	0.32	26.411	679.08	0.30
						26.431	773.69	0.32	26.431	679.37	0.30
						26.451	773.92	0.33	26.451	679.65	0.30
						26.471	774.13	0.33	26.471	679.92	0.30
						26.491	774.31	0.33	26.491	680.15	0.30
						26.511	774.49	0.33	26.511	680.39	0.30
						26.531	774.67	0.33	26.531	680.67	0.30
						26.551	774.90	0.33	26.550	680.96	0.30
						26.571	775.11	0.33	26.570	681.24	0.30
						26.591	775.29	0.33	26.590	681.48	0.30
						26.611	775.45	0.33	26.610	681.70	0.30
						26.630	775.60	0.33	26.630	682.01	0.30
						26.650	775.78	0.33	26.650	682.29	0.30
						26.670	775.98	0.33	26.670	682.50	0.30
						26.690	776.19	0.33	26.690	682.74	0.30
						26.710	776.44	0.33	26.710	683.05	0.30
						26.730	776.64	0.33	26.730	683.33	0.30
						26.750	776.83	0.33	26.750	683.59	0.30
						26.770	777.02	0.33	26.770	683.84	0.30
						26.790	777.22	0.33	26.790	684.09	0.31
						26.810	777.39	0.33	26.810	684.33	0.31
						26.830	777.57	0.33	26.830	684.58	0.31

						26.850	777.78	0.33	26.850	684.82	0.31
						26.870	778.02	0.33	26.870	685.07	0.31
						26.890	778.20	0.33	26.889	685.35	0.31
						26.910	778.37	0.33	26.909	685.64	0.31
						26.930	778.55	0.33	26.929	685.89	0.31
						26.949	778.73	0.33	26.949	686.13	0.31
						26.969	778.96	0.33	26.969	686.37	0.31
						26.989	779.15	0.33	26.989	686.61	0.31
						27.009	779.33	0.33	27.009	686.87	0.31
						27.029	779.56	0.33	27.029	687.15	0.31
						27.049	779.74	0.33	27.049	687.39	0.31
						27.069	779.91	0.33	27.069	687.62	0.31
						27.089	780.12	0.33	27.089	687.88	0.31
						27.109	780.33	0.33	27.109	688.14	0.31
						27.129	780.53	0.33	27.129	688.39	0.31
						27.149	780.68	0.33	27.149	688.61	0.31
						27.169	780.86	0.33	27.169	688.81	0.31
						27.189	781.07	0.33	27.189	688.95	0.31
						27.209	781.27	0.33	27.208	689.21	0.31
						27.229	781.46	0.33	27.228	689.47	0.31
						27.249	781.60	0.33	27.248	689.73	0.31
						27.268	781.79	0.33	27.268	690.00	0.31
						27.288	781.99	0.33	27.288	690.24	0.31
						27.308	782.19	0.33	27.308	690.51	0.31
						27.328	782.37	0.33	27.328	690.79	0.31
						27.348	782.52	0.33	27.348	691.03	0.31
						27.368	782.71	0.33	27.368	691.28	0.31
						27.388	782.91	0.33	27.388	691.56	0.31
						27.408	783.11	0.33	27.408	691.81	0.31
						27.428	783.28	0.33	27.428	692.10	0.31
						27.448	783.43	0.33	27.448	692.33	0.31
						27.468	783.62	0.33	27.468	692.61	0.31
						27.488	783.82	0.33	27.488	692.84	0.31
						27.508	784.00	0.33	27.508	693.10	0.31
						27.528	784.18	0.33	27.528	693.32	0.31
						27.548	784.37	0.33	27.547	693.58	0.31
						27.568	784.56	0.33	27.567	693.84	0.31
						27.588	784.74	0.33	27.587	694.09	0.31
						27.607	784.93	0.33	27.607	694.39	0.31
						27.627	785.09	0.33	27.627	694.65	0.31
						27.647	785.27	0.33	27.647	694.92	0.31
						27.667	785.45	0.33	27.667	695.12	0.31
						27.687	785.63	0.33	27.687	695.38	0.31
						27.707	785.85	0.33	27.707	695.65	0.31
						27.727	786.04	0.33	27.727	695.91	0.31

						27.747	786.21	0.33	27.747	696.17	0.31
						27.767	786.38	0.33	27.767	696.42	0.31
						27.787	786.54	0.33	27.787	696.66	0.31
						27.807	786.70	0.33	27.807	696.90	0.31
						27.827	786.87	0.33	27.827	697.15	0.31
						27.847	787.08	0.33	27.847	697.40	0.31
						27.867	787.29	0.33	27.866	697.65	0.31
						27.887	787.46	0.33	27.886	697.89	0.31
						27.907	787.62	0.33	27.906	698.13	0.31
						27.926	787.79	0.33	27.926	698.37	0.31
						27.946	787.96	0.33	27.946	698.61	0.31
						27.966	788.10	0.33	27.966	698.86	0.31
						27.986	788.27	0.33	27.986	699.14	0.31
						28.006	788.46	0.33	28.006	699.36	0.31
						28.026	788.63	0.33	28.026	699.61	0.31
						28.046	788.80	0.33	28.046	699.88	0.31
						28.066	789.01	0.33	28.066	700.10	0.31
						28.086	789.23	0.33	28.086	700.34	0.31
						28.106	789.40	0.33	28.106	700.59	0.31
						28.126	789.57	0.33	28.126	700.85	0.31
						28.146	789.77	0.33	28.146	701.11	0.31
						28.166	789.95	0.33	28.166	701.36	0.31
						28.186	790.12	0.33	28.186	701.60	0.31
						28.206	790.27	0.33	28.205	701.83	0.31
						28.226	790.49	0.33	28.225	702.02	0.31
						28.245	790.68	0.33	28.245	702.23	0.31
						28.265	790.85	0.33	28.265	702.48	0.31
						28.285	791.02	0.33	28.285	702.74	0.31
						28.305	791.18	0.33	28.305	703.01	0.31
						28.325	791.35	0.33	28.325	703.24	0.31
						28.345	791.51	0.33	28.345	703.47	0.31
						28.365	791.75	0.33	28.365	703.70	0.31
						28.385	791.91	0.33	28.385	703.93	0.31
						28.405	792.07	0.33	28.405	704.15	0.31
						28.425	792.23	0.33	28.425	704.39	0.31
						28.445	792.46	0.33	28.445	704.65	0.31
						28.465	792.62	0.33	28.465	704.86	0.31
						28.485	792.77	0.33	28.485	705.06	0.31
						28.505	792.98	0.33	28.505	705.30	0.31
						28.525	793.16	0.33	28.524	705.55	0.31
						28.545	793.34	0.33	28.544	705.77	0.31
						28.564	793.52	0.33	28.564	705.97	0.31
						28.584	793.68	0.33	28.584	706.22	0.31
						28.604	793.90	0.33	28.604	706.47	0.31
						28.624	794.04	0.33	28.624	706.71	0.31

						28.644	794.19	0.33	28.644	706.96	0.31
						28.664	794.43	0.33	28.664	707.15	0.31
						28.684	794.59	0.33	28.684	707.34	0.31
						28.704	794.74	0.33	28.704	707.57	0.31
						28.724	794.95	0.33	28.724	707.80	0.31
						28.744	795.11	0.33	28.744	708.02	0.31
						28.764	795.31	0.33	28.764	708.23	0.31
						28.784	795.48	0.33	28.784	708.47	0.31
						28.804	795.63	0.33	28.804	708.74	0.31
						28.824	795.79	0.33	28.824	708.97	0.31
						28.844	795.99	0.33	28.844	709.19	0.31
						28.864	796.12	0.33	28.863	709.40	0.31
						28.883	796.31	0.33	28.883	709.61	0.31
						28.903	796.48	0.33	28.903	709.82	0.31
						28.923	796.67	0.33	28.923	710.04	0.31
						28.943	796.85	0.33	28.943	710.25	0.31
						28.963	797.01	0.33	28.963	710.49	0.31
						28.983	797.22	0.33	28.983	710.73	0.31
						29.003	797.36	0.33	29.003	710.96	0.31
						29.023	797.56	0.33	29.023	711.16	0.31
						29.043	797.70	0.33	29.043	711.36	0.31
						29.063	797.89	0.33	29.063	711.49	0.31
						29.083	798.06	0.33	29.083	711.63	0.31
						29.103	798.23	0.33	29.103	711.85	0.31
						29.123	798.43	0.33	29.123	712.10	0.31
						29.143	798.61	0.33	29.143	712.34	0.31
						29.163	798.74	0.33	29.163	712.56	0.31
						29.183	798.89	0.33	29.182	712.78	0.31
						29.202	799.08	0.33	29.202	713.00	0.31
						29.222	799.28	0.33	29.222	713.22	0.31
						29.242	799.42	0.33	29.242	713.43	0.31
						29.262	799.61	0.33	29.262	713.64	0.31
						29.282	799.78	0.33	29.282	713.86	0.31
						29.302	799.94	0.33	29.302	714.09	0.31
						29.322	800.13	0.33	29.322	714.31	0.31
						29.342	800.27	0.33	29.342	714.52	0.31
						29.362	800.48	0.33	29.362	714.74	0.31
						29.382	800.63	0.33	29.382	714.94	0.31
						29.402	800.81	0.33	29.402	715.19	0.31
						29.422	800.97	0.33	29.422	715.41	0.31
						29.442	801.11	0.33	29.442	715.62	0.31
						29.462	801.29	0.33	29.462	715.87	0.31
						29.482	801.46	0.33	29.482	716.10	0.31
						29.502	801.62	0.33	29.502	716.31	0.31
						29.521	801.78	0.33	29.521	716.50	0.31

						29.541	801.95	0.33	29.541	716.69	0.31
						29.561	802.12	0.33	29.561	716.93	0.31
						29.581	802.30	0.33	29.581	717.15	0.31
						29.601	802.48	0.33	29.601	717.35	0.31
						29.621	802.66	0.33	29.621	717.56	0.31
						29.641	802.81	0.33	29.641	717.80	0.31
						29.661	802.93	0.33	29.661	717.99	0.31
						29.681	803.10	0.33	29.681	718.21	0.31
						29.701	803.28	0.33	29.701	718.44	0.31
						29.721	803.45	0.33	29.721	718.66	0.31
						29.741	803.64	0.33	29.741	718.84	0.31
						29.761	803.77	0.33	29.761	719.05	0.31
						29.781	803.93	0.33	29.781	719.28	0.31
						29.801	804.10	0.33	29.801	719.51	0.31
						29.821	804.28	0.33	29.821	719.73	0.31
						29.841	804.42	0.33	29.840	719.94	0.31
						29.860	804.58	0.33	29.860	720.15	0.31
						29.880	804.77	0.33	29.880	720.35	0.31
						29.900	804.95	0.33	29.900	720.55	0.31
						29.920	805.13	0.33	29.920	720.74	0.31
						29.940	805.30	0.33	29.940	720.93	0.31
						29.960	805.47	0.33	29.960	721.16	0.31
						29.980	805.64	0.33	29.980	721.33	0.31
						30.000	805.80	0.33	30.000	721.49	0.31

Combined standard uncertainties:

$u(T)$= 0.006 K; $u(p)$= 0.0020 MPa for p < 6 MPa; $u(p)$= 0.024 MPa for 6 MPa ≤ p ≤ 70 MPa

$u(x_{CO_2})$= 0.00024; $u(x_{O_2})$= 0.000030; $u(x_{SO_2})$= 0.0000023; $u(x_{CO})$= 0.0000043

Table S1 (continued). $p\rho T$ experimental data for the $CO_2 + O_2 + SO_2 + CO$ (Mix 1) and $CO_2 + NO + SO_2 + CO$ (Mix 2) mixtures. $u(\rho)$: combined standard uncertainty.

Mix 1: x_{CO_2} = 0.96734; x_{O_2} = 0.030038; x_{SO_2} = 0.0009035; x_{CO} = 0.0017032											
***T*= 373.18±0.01 K**											
p (MPa)	ρ (kg.m^{-3})	$u(\rho)$ (kg.m^{-3})									
0.115	1.22	0.20									
0.135	1.55	0.20									
0.155	1.89	0.21									
0.175	2.13	0.21									
0.195	2.38	0.21									
0.215	2.70	0.21									
0.235	2.96	0.21									
0.255	3.30	0.21									
0.275	3.51	0.21									
0.295	3.80	0.21									
0.315	4.10	0.21									
0.335	4.37	0.21									
0.354	4.66	0.21									
0.374	4.99	0.22									
0.394	5.28	0.22									
0.414	5.52	0.21									
0.434	5.79	0.21									
0.454	6.11	0.22									
0.474	6.42	0.22									
0.494	6.67	0.22									
0.514	6.95	0.22									
0.534	7.26	0.22									
0.554	7.54	0.22									
0.574	7.86	0.22									
0.594	8.17	0.22									
0.614	8.43	0.22									
0.634	8.74	0.22									
0.653	9.02	0.22									
0.673	9.31	0.22									
0.693	9.60	0.22									
0.713	9.85	0.22									
0.733	10.20	0.22									
0.753	10.51	0.22									
0.773	10.80	0.22									
0.793	11.11	0.22									

0.813	11.40	0.22									
0.833	11.67	0.22									
0.853	11.86	0.22									
0.873	12.21	0.22									
0.893	12.44	0.22									
0.913	12.75	0.22									
0.933	13.06	0.22									
0.953	13.35	0.22									
0.972	13.62	0.22									
0.992	13.94	0.22									
1.012	14.23	0.22									
1.032	14.55	0.22									
1.052	14.79	0.22									
1.072	15.09	0.22									
1.092	15.38	0.22									
1.112	15.72	0.22									
1.132	16.02	0.22									
1.152	16.28	0.22									
1.172	16.56	0.22									
1.192	16.88	0.22									
1.212	17.17	0.22									
1.232	17.46	0.22									
1.252	17.77	0.22									
1.272	18.11	0.22									
1.291	18.42	0.22									
1.311	18.71	0.22									
1.331	18.98	0.22									
1.351	19.31	0.22									
1.371	19.62	0.22									
1.391	19.90	0.22									
1.411	20.20	0.22									
1.431	20.51	0.22									
1.451	20.81	0.22									
1.471	21.12	0.22									
1.491	21.44	0.22									
1.511	21.75	0.22									
1.531	22.06	0.22									
1.551	22.37	0.22									
1.571	22.70	0.22									
1.591	23.03	0.22									
1.610	23.31	0.22									
1.630	23.58	0.22									
1.650	23.83	0.22									
1.670	24.09	0.22									
1.690	24.40	0.22									

1.710	24.70	0.22									
1.730	24.99	0.22									
1.750	25.27	0.22									
1.770	25.63	0.22									
1.790	25.90	0.22									
1.810	26.22	0.22									
1.830	26.55	0.22									
1.850	26.87	0.22									
1.870	27.17	0.22									
1.890	27.42	0.22									
1.909	27.78	0.22									
1.929	28.10	0.22									
1.949	28.40	0.22									
1.969	28.70	0.22									
1.989	29.01	0.22									
2.009	29.33	0.22									
2.029	29.63	0.22									
2.049	29.93	0.22									
2.069	30.21	0.22									
2.089	30.55	0.22									
2.109	30.87	0.22									
2.129	31.17	0.22									
2.149	31.42	0.22									
2.169	31.74	0.22									
2.189	32.09	0.22									
2.209	32.41	0.22									
2.228	32.70	0.22									
2.248	33.00	0.22									
2.268	33.33	0.22									
2.288	33.67	0.22									
2.308	34.00	0.22									
2.328	34.24	0.22									
2.348	34.59	0.22									
2.368	34.92	0.22									
2.388	35.22	0.22									
2.408	35.51	0.22									
2.428	35.80	0.22									
2.448	36.12	0.22									
2.468	36.46	0.22									
2.488	36.80	0.22									
2.508	37.12	0.22									
2.528	37.44	0.22									
2.547	37.78	0.22									
2.567	38.08	0.22									
2.587	38.39	0.22									

2.607	38.70	0.22									
2.627	39.00	0.22									
2.647	39.31	0.22									
2.667	39.63	0.22									
2.687	39.97	0.22									
2.707	40.28	0.22									
2.727	40.56	0.22									
2.747	40.89	0.22									
2.767	41.22	0.22									
2.787	41.54	0.22									
2.807	41.85	0.22									
2.827	42.17	0.22									
2.847	42.48	0.22									
2.866	42.81	0.22									
2.886	43.14	0.22									
2.906	43.47	0.22									
2.926	43.81	0.22									
2.946	44.13	0.22									
2.966	44.46	0.22									
2.986	44.78	0.22									
3.006	45.10	0.22									
3.026	45.42	0.22									
3.046	45.75	0.22									
3.066	46.05	0.22									
3.086	46.34	0.22									
3.106	46.67	0.22									
3.126	47.02	0.22									
3.146	47.35	0.22									
3.165	47.66	0.22									
3.185	47.97	0.22									
3.205	48.29	0.22									
3.225	48.66	0.22									
3.245	48.94	0.22									
3.265	49.27	0.22									
3.285	49.62	0.22									
3.305	49.96	0.22									
3.325	50.30	0.22									
3.345	50.60	0.22									
3.365	50.89	0.22									
3.385	51.22	0.22									
3.405	51.53	0.22									
3.425	51.89	0.22									
3.445	52.22	0.22									
3.465	52.55	0.22									
3.484	52.90	0.22									

3.504	53.22	0.22									
3.524	53.56	0.22									
3.544	53.91	0.22									
3.564	54.25	0.22									
3.584	54.54	0.22									
3.604	54.88	0.22									
3.624	55.23	0.22									
3.644	55.54	0.22									
3.664	55.90	0.22									
3.684	56.22	0.22									
3.704	56.55	0.22									
3.724	56.89	0.22									
3.744	57.18	0.22									
3.764	57.57	0.22									
3.784	57.92	0.22									
3.803	58.21	0.22									
3.823	58.54	0.22									
3.843	58.87	0.22									
3.863	59.22	0.22									
3.883	59.60	0.22									
3.903	59.93	0.22									
3.923	60.25	0.22									
3.943	60.57	0.22									
3.963	60.94	0.22									
3.983	61.28	0.22									
4.003	61.59	0.22									
4.023	61.94	0.22									
4.043	62.29	0.22									
4.063	62.64	0.22									
4.083	62.99	0.22									
4.103	63.33	0.22									
4.122	63.68	0.22									
4.142	64.02	0.22									
4.162	64.36	0.22									
4.182	64.70	0.22									
4.202	65.04	0.22									
4.222	65.39	0.22									
4.242	65.69	0.22									
4.262	66.04	0.22									
4.282	66.38	0.22									
4.302	66.73	0.22									
4.322	67.09	0.22									
4.342	67.44	0.22									
4.362	67.78	0.22									
4.382	68.11	0.22									

4.402	68.46	0.22									
4.421	68.83	0.22									
4.441	69.20	0.22									
4.461	69.53	0.22									
4.481	69.90	0.22									
4.501	70.25	0.22									
4.521	70.58	0.22									
4.541	70.95	0.22									
4.561	71.30	0.22									
4.581	71.64	0.22									
4.601	72.00	0.22									
4.621	72.30	0.22									
4.641	72.66	0.22									
4.661	73.06	0.22									
4.681	73.41	0.22									
4.701	73.76	0.22									
4.721	74.10	0.22									
4.740	74.44	0.22									
4.760	74.78	0.22									
4.780	75.15	0.22									
4.800	75.52	0.22									
4.820	75.86	0.22									
4.840	76.23	0.22									
4.860	76.58	0.22									
4.880	76.91	0.22									
4.900	77.29	0.22									
4.920	77.64	0.22									
4.940	77.98	0.22									
4.960	78.33	0.22									
4.980	78.68	0.22									
5.000	79.05	0.22									
5.020	79.45	0.22									
5.040	79.79	0.22									
5.059	80.13	0.22									
5.079	80.49	0.22									
5.099	80.88	0.22									
5.119	81.20	0.22									
5.139	81.57	0.22									
5.159	81.95	0.22									
5.179	82.29	0.22									
5.199	82.66	0.22									
5.219	83.03	0.22									
5.239	83.40	0.22									
5.259	83.76	0.22									
5.279	84.12	0.22									

5.299	84.49	0.22									
5.319	84.85	0.22									
5.339	85.20	0.22									
5.359	85.56	0.22									
5.378	85.91	0.22									
5.398	86.30	0.22									
5.418	86.70	0.22									
5.438	87.10	0.22									
5.458	87.50	0.22									
5.478	87.88	0.22									
5.498	88.23	0.22									
5.518	88.47	0.22									
5.538	88.91	0.22									
5.558	89.34	0.22									
5.578	89.70	0.22									
5.598	90.06	0.22									
5.618	90.42	0.22									
5.638	90.78	0.22									
5.658	91.15	0.22									
5.677	91.56	0.22									
5.697	91.91	0.22									
5.717	92.29	0.22									
5.737	92.67	0.22									
5.757	93.02	0.22									
5.777	93.41	0.22									
5.797	93.80	0.22									
5.817	94.19	0.22									
5.837	94.52	0.22									
5.857	94.92	0.22									
5.877	95.34	0.22									
5.897	95.73	0.22									
5.917	96.11	0.22									
5.937	96.48	0.22									
5.957	96.86	0.22									
5.977	97.23	0.22									
5.996	97.60	0.22									
6.016	97.96	0.22									
6.036	98.37	0.22									
6.056	98.76	0.22									
6.076	99.12	0.22									
6.096	99.49	0.22									
6.116	99.85	0.22									
6.136	100.21	0.22									
6.156	100.57	0.22									
6.176	100.96	0.22									

6.196	101.36	0.22									
6.216	101.77	0.22									
6.236	102.13	0.22									
6.256	102.50	0.22									
6.276	102.90	0.22									
6.296	103.27	0.22									
6.315	103.64	0.22									
6.335	104.02	0.22									
6.355	104.42	0.22									
6.375	104.84	0.22									
6.395	105.21	0.22									
6.415	105.56	0.22									
6.435	105.95	0.22									
6.455	106.35	0.22									
6.475	106.71	0.22									
6.495	107.11	0.22									
6.515	107.53	0.22									
6.535	107.94	0.22									
6.555	108.29	0.22									
6.575	108.69	0.22									
6.595	109.08	0.22									
6.615	109.49	0.22									
6.634	109.89	0.22									
6.654	110.29	0.22									
6.674	110.69	0.22									
6.694	111.08	0.22									
6.714	111.47	0.22									
6.734	111.86	0.22									
6.754	112.24	0.22									
6.774	112.62	0.22									
6.794	113.05	0.22									
6.814	113.46	0.22									
6.834	113.84	0.22									
6.854	114.26	0.22									
6.874	114.64	0.22									
6.894	114.97	0.22									
6.914	115.40	0.22									
6.933	115.82	0.22									
6.953	116.22	0.22									
6.973	116.59	0.22									
6.993	117.01	0.22									
7.013	117.43	0.22									
7.033	117.81	0.22									
7.053	118.18	0.22									
7.073	118.58	0.22									

7.093	118.99	0.22									
7.113	119.40	0.22									
7.133	119.80	0.22									
7.153	120.21	0.22									
7.173	120.61	0.22									
7.193	121.02	0.22									
7.213	121.42	0.22									
7.233	121.84	0.22									
7.252	122.21	0.22									
7.272	122.61	0.22									
7.292	123.03	0.22									
7.312	123.39	0.22									
7.332	123.82	0.22									
7.352	124.25	0.22									
7.372	124.67	0.22									
7.392	125.08	0.22									
7.412	125.49	0.22									
7.432	125.90	0.22									
7.452	126.30	0.22									
7.472	126.71	0.22									
7.492	127.11	0.22									
7.512	127.55	0.22									
7.532	128.00	0.22									
7.552	128.40	0.22									
7.571	128.79	0.22									
7.591	129.18	0.22									
7.611	129.59	0.22									
7.631	130.04	0.22									
7.651	130.45	0.22									
7.671	130.87	0.22									
7.691	131.30	0.22									
7.711	131.70	0.22									
7.731	132.11	0.22									
7.751	132.54	0.22									
7.771	132.98	0.22									
7.791	133.41	0.22									
7.811	133.84	0.22									
7.831	134.28	0.22									
7.851	134.70	0.22									
7.871	135.11	0.22									
7.890	135.47	0.22									
7.910	135.88	0.22									
7.930	136.30	0.22									
7.950	136.71	0.22									
7.970	137.14	0.22									

7.990	137.55	0.22									
8.010	137.99	0.22									
8.030	138.45	0.22									
8.050	138.86	0.22									
8.070	139.27	0.22									
8.090	139.70	0.22									
8.110	140.15	0.22									
8.130	140.57	0.22									
8.150	141.02	0.22									
8.170	141.46	0.22									
8.189	141.86	0.22									
8.209	142.31	0.22									
8.229	142.72	0.22									
8.249	143.13	0.22									
8.269	143.56	0.23									
8.289	143.99	0.23									
8.309	144.41	0.23									
8.329	144.84	0.23									
8.349	145.27	0.23									
8.369	145.70	0.23									
8.389	146.18	0.23									
8.409	146.62	0.23									
8.429	147.04	0.23									
8.449	147.46	0.23									
8.469	147.94	0.23									
8.489	148.37	0.23									
8.508	148.77	0.23									
8.528	149.17	0.23									
8.548	149.64	0.23									
8.568	150.11	0.23									
8.588	150.56	0.23									
8.608	150.95	0.23									
8.628	151.40	0.23									
8.648	151.86	0.23									
8.668	152.32	0.23									
8.688	152.78	0.23									
8.708	153.23	0.23									
8.728	153.65	0.23									
8.748	154.02	0.23									
8.768	154.46	0.23									
8.788	154.91	0.23									
8.808	155.34	0.23									
8.827	155.76	0.23									
8.847	156.21	0.23									
8.867	156.69	0.23									

8.887	157.15	0.23									
8.907	157.57	0.23									
8.927	158.00	0.23									
8.947	158.48	0.23									
8.967	158.93	0.23									
8.987	159.37	0.23									
9.007	159.84	0.23									
9.027	160.32	0.23									
9.047	160.75	0.23									
9.067	161.18	0.23									
9.087	161.64	0.23									
9.107	162.10	0.23									
9.126	162.55	0.23									
9.146	163.01	0.23									
9.166	163.48	0.23									
9.186	163.93	0.23									
9.206	164.38	0.23									
9.226	164.83	0.23									
9.246	165.28	0.23									
9.266	165.73	0.23									
9.286	166.19	0.23									
9.306	166.63	0.23									
9.326	167.08	0.23									
9.346	167.54	0.23									
9.366	168.03	0.23									
9.386	168.47	0.23									
9.406	168.90	0.23									
9.426	169.35	0.23									
9.445	169.83	0.23									
9.465	170.31	0.23									
9.485	170.78	0.23									
9.505	171.23	0.23									
9.525	171.65	0.23									
9.545	172.13	0.23									
9.565	172.60	0.23									
9.585	173.07	0.23									
9.605	173.54	0.23									
9.625	174.00	0.23									
9.645	174.45	0.23									
9.665	174.92	0.23									
9.685	175.45	0.23									
9.705	175.91	0.23									
9.725	176.36	0.23									
9.745	176.82	0.23									
9.764	177.32	0.23									

9.784	177.77	0.23									
9.804	178.24	0.23									
9.824	178.73	0.23									
9.844	179.18	0.23									
9.864	179.66	0.23									
9.884	180.15	0.23									
9.904	180.63	0.23									
9.924	181.08	0.23									
9.944	181.52	0.23									
9.964	182.02	0.23									
9.984	182.48	0.23									
10.004	182.94	0.23									
10.024	183.43	0.23									
10.044	183.92	0.23									
10.064	184.40	0.23									
10.083	184.87	0.23									
10.103	185.35	0.23									
10.123	185.82	0.23									
10.143	186.29	0.23									
10.163	186.77	0.23									
10.183	187.25	0.23									
10.203	187.70	0.23									
10.223	188.21	0.23									
10.243	188.70	0.23									
10.263	189.17	0.23									
10.283	189.63	0.23									
10.303	190.14	0.23									
10.323	190.64	0.23									
10.343	191.09	0.23									
10.363	191.60	0.23									
10.382	192.11	0.23									
10.402	192.61	0.23									
10.422	193.06	0.23									
10.442	193.55	0.23									
10.462	194.05	0.23									
10.482	194.56	0.23									
10.502	195.05	0.23									
10.522	195.51	0.23									
10.542	195.97	0.23									
10.562	196.52	0.23									
10.582	197.02	0.23									
10.602	197.51	0.23									
10.622	198.00	0.23									
10.642	198.49	0.23									
10.662	198.97	0.23									

10.682	199.45	0.23									
10.701	199.93	0.23									
10.721	200.42	0.23									
10.741	200.95	0.23									
10.761	201.41	0.23									
10.781	201.88	0.23									
10.801	202.36	0.23									
10.821	202.90	0.23									
10.841	203.37	0.23									
10.861	203.87	0.23									
10.881	204.40	0.23									
10.901	204.92	0.23									
10.921	205.39	0.23									
10.941	205.87	0.23									
10.961	206.40	0.23									
10.981	206.91	0.23									
11.001	207.41	0.23									
11.020	207.92	0.23									
11.040	208.43	0.23									
11.060	208.94	0.23									
11.080	209.41	0.23									
11.100	209.87	0.23									
11.120	210.44	0.23									
11.140	210.97	0.23									
11.160	211.47	0.23									
11.180	211.97	0.23									
11.200	212.47	0.23									
11.220	212.99	0.23									
11.240	213.53	0.23									
11.260	214.02	0.23									
11.280	214.50	0.23									
11.300	214.98	0.23									
11.320	215.52	0.23									
11.339	216.05	0.23									
11.359	216.57	0.23									
11.379	217.05	0.23									
11.399	217.52	0.23									
11.419	218.02	0.23									
11.439	218.57	0.23									
11.459	219.06	0.23									
11.479	219.55	0.23									
11.499	220.07	0.23									
11.519	220.62	0.23									
11.539	221.11	0.23									
11.559	221.64	0.23									

11.579	222.17	0.23									
11.599	222.69	0.23									
11.619	223.16	0.23									
11.638	223.71	0.23									
11.658	224.25	0.23									
11.678	224.76	0.23									
11.698	225.23	0.23									
11.718	225.77	0.23									
11.738	226.30	0.23									
11.758	226.83	0.23									
11.778	227.35	0.23									
11.798	227.87	0.23									
11.818	228.39	0.23									
11.838	228.92	0.23									
11.858	229.43	0.23									
11.878	230.00	0.23									
11.898	230.54	0.23									
11.918	231.04	0.23									
11.938	231.56	0.23									
11.957	232.05	0.23									
11.977	232.59	0.23									
11.997	233.14	0.23									
12.017	233.63	0.23									
12.037	234.19	0.23									
12.057	234.70	0.23									
12.077	235.22	0.23									
12.097	235.77	0.23									
12.117	236.27	0.23									
12.137	236.82	0.23									
12.157	237.34	0.23									
12.177	237.86	0.23									
12.197	238.41	0.23									
12.217	238.96	0.23									
12.237	239.51	0.23									
12.257	240.05	0.23									
12.276	240.60	0.23									
12.296	241.12	0.23									
12.316	241.65	0.23									
12.336	242.19	0.23									
12.356	242.72	0.23									
12.376	243.28	0.23									
12.396	243.81	0.23									
12.416	244.34	0.23									
12.436	244.86	0.23									
12.456	245.38	0.23									

12.476	245.91	0.23									
12.496	246.47	0.23									
12.516	247.04	0.23									
12.536	247.57	0.23									
12.556	248.08	0.23									
12.576	248.62	0.23									
12.595	249.19	0.23									
12.615	249.75	0.23									
12.635	250.30	0.23									
12.655	250.85	0.23									
12.675	251.34	0.23									
12.695	251.88	0.23									
12.715	252.43	0.23									
12.735	252.99	0.23									
12.755	253.55	0.23									
12.775	254.11	0.23									
12.795	254.67	0.23									
12.815	255.20	0.23									
12.835	255.74	0.23									
12.855	256.28	0.23									
12.875	256.83	0.23									
12.894	257.37	0.23									
12.914	257.92	0.23									
12.934	258.45	0.23									
12.954	259.04	0.23									
12.974	259.62	0.23									
12.994	260.15	0.23									
13.014	260.68	0.23									
13.034	261.23	0.23									
13.054	261.80	0.23									
13.074	262.38	0.23									
13.094	262.93	0.23									
13.114	263.45	0.23									
13.134	264.01	0.23									
13.154	264.57	0.23									
13.174	265.13	0.23									
13.194	265.69	0.23									
13.213	266.24	0.23									
13.233	266.70	0.23									
13.253	267.26	0.23									
13.273	267.81	0.23									
13.293	268.32	0.23									
13.313	268.93	0.23									
13.333	269.49	0.23									
13.353	270.04	0.23									

13.373	270.58	0.23									
13.393	271.13	0.24									
13.413	271.71	0.24									
13.433	272.28	0.24									
13.453	272.83	0.24									
13.473	273.42	0.24									
13.493	273.95	0.24									
13.513	274.53	0.24									
13.532	275.10	0.24									
13.552	275.62	0.24									
13.572	276.19	0.24									
13.592	276.79	0.24									
13.612	277.37	0.24									
13.632	277.89	0.24									
13.652	278.44	0.24									
13.672	279.00	0.24									
13.692	279.58	0.24									
13.712	280.16	0.24									
13.732	280.71	0.24									
13.752	281.27	0.24									
13.772	281.84	0.24									
13.792	282.40	0.24									
13.812	282.99	0.24									
13.832	283.60	0.24									
13.851	284.15	0.24									
13.871	284.70	0.24									
13.891	285.25	0.24									
13.911	285.79	0.24									
13.931	286.38	0.24									
13.951	286.97	0.24									
13.971	287.53	0.24									
13.991	288.07	0.24									
14.011	288.64	0.24									
14.031	289.24	0.24									
14.051	289.83	0.24									
14.071	290.39	0.24									
14.091	290.92	0.24									
14.111	291.45	0.24									
14.131	292.00	0.24									
14.150	292.59	0.24									
14.170	293.21	0.24									
14.190	293.75	0.24									
14.210	294.30	0.24									
14.230	294.89	0.24									
14.250	295.47	0.24									

14.270	296.02	0.24									
14.290	296.61	0.24									
14.310	297.21	0.24									
14.330	297.79	0.24									
14.350	298.31	0.24									
14.370	298.89	0.24									
14.390	299.48	0.24									
14.410	300.06	0.24									
14.430	300.63	0.24									
14.450	301.21	0.24									
14.469	301.79	0.24									
14.489	302.37	0.24									
14.509	302.92	0.24									
14.529	303.53	0.24									
14.549	304.14	0.24									
14.569	304.69	0.24									
14.589	305.25	0.24									
14.609	305.81	0.24									
14.629	306.39	0.24									
14.649	307.01	0.24									
14.669	307.58	0.24									
14.689	308.14	0.24									
14.709	308.75	0.24									
14.729	309.31	0.24									
14.749	309.89	0.24									
14.769	310.50	0.24									
14.788	311.04	0.24									
14.808	311.61	0.24									
14.828	312.19	0.24									
14.848	312.80	0.24									
14.868	313.35	0.24									
14.888	313.88	0.24									
14.908	314.46	0.24									
14.928	315.08	0.24									
14.948	315.70	0.24									
14.968	316.27	0.24									
14.988	316.85	0.24									
15.008	317.42	0.24									
15.028	318.01	0.24									
15.048	318.58	0.24									
15.068	319.14	0.24									
15.088	319.72	0.24									
15.107	320.31	0.24									
15.127	320.91	0.24									
15.147	321.47	0.24									

15.167	322.04	0.24									
15.187	322.64	0.24									
15.207	323.20	0.24									
15.227	323.76	0.24									
15.247	324.35	0.24									
15.267	324.91	0.24									
15.287	325.46	0.24									
15.307	326.03	0.24									
15.327	326.56	0.24									
15.347	327.15	0.24									
15.367	327.75	0.24									
15.387	328.29	0.24									
15.406	328.90	0.24									
15.426	329.48	0.24									
15.446	330.04	0.24									
15.466	330.62	0.24									
15.486	331.22	0.24									
15.506	331.82	0.24									
15.526	332.42	0.24									
15.546	332.99	0.24									
15.566	333.53	0.24									
15.586	334.13	0.24									
15.606	334.73	0.24									
15.626	335.30	0.24									
15.646	335.86	0.24									
15.666	336.49	0.24									
15.686	337.11	0.24									
15.706	337.67	0.24									
15.725	338.23	0.24									
15.745	338.83	0.24									
15.765	339.44	0.24									
15.785	339.98	0.24									
15.805	340.56	0.24									
15.825	341.15	0.24									
15.845	341.70	0.24									
15.865	342.26	0.24									
15.885	342.82	0.24									
15.905	343.38	0.24									
15.925	344.02	0.24									
15.945	344.60	0.24									
15.965	345.17	0.24									
15.985	345.73	0.24									
16.005	346.30	0.24									
16.025	346.91	0.24									
16.044	347.48	0.24									

16.064	348.07	0.24									
16.084	348.67	0.24									
16.104	349.22	0.24									
16.124	349.83	0.24									
16.144	350.42	0.24									
16.164	351.01	0.24									
16.184	351.60	0.24									
16.204	352.18	0.24									
16.224	352.77	0.24									
16.244	353.35	0.24									
16.264	353.93	0.24									
16.284	354.52	0.24									
16.304	355.10	0.24									
16.324	355.68	0.25									
16.344	356.26	0.25									
16.363	356.83	0.25									
16.383	357.39	0.25									
16.403	357.95	0.25									
16.423	358.52	0.25									
16.443	359.11	0.25									
16.463	359.72	0.25									
16.483	360.28	0.25									
16.503	360.87	0.25									
16.523	361.46	0.25									
16.543	362.03	0.25									
16.563	362.59	0.25									
16.583	363.14	0.25									
16.603	363.72	0.25									
16.623	364.30	0.25									
16.643	364.87	0.25									
16.662	365.42	0.25									
16.682	366.00	0.25									
16.702	366.57	0.25									
16.722	367.13	0.25									
16.742	367.75	0.25									
16.762	368.36	0.25									
16.782	368.93	0.25									
16.802	369.49	0.25									
16.822	370.04	0.25									
16.842	370.61	0.25									
16.862	371.17	0.25									
16.882	371.72	0.25									
16.902	372.25	0.25									
16.922	372.79	0.25									
16.942	373.35	0.25									

16.962	373.92	0.25									
16.981	374.52	0.25									
17.001	375.07	0.25									
17.021	375.66	0.25									
17.041	376.22	0.25									
17.061	376.78	0.25									
17.081	377.35	0.25									
17.101	377.94	0.25									
17.121	378.55	0.25									
17.141	379.09	0.25									
17.161	379.65	0.25									
17.181	380.27	0.25									
17.201	380.86	0.25									
17.221	381.42	0.25									
17.241	381.99	0.25									
17.261	382.58	0.25									
17.281	383.11	0.25									
17.300	383.71	0.25									
17.320	384.26	0.25									
17.340	384.82	0.25									
17.360	385.40	0.25									
17.380	385.98	0.25									
17.400	386.51	0.25									
17.420	387.09	0.25									
17.440	387.67	0.25									
17.460	388.25	0.25									
17.480	388.83	0.25									
17.500	389.38	0.25									
17.520	390.00	0.25									
17.540	390.58	0.25									
17.560	391.15	0.25									
17.580	391.72	0.25									
17.600	392.27	0.25									
17.619	392.82	0.25									
17.639	393.47	0.25									
17.659	394.13	0.25									
17.679	394.66	0.25									
17.699	395.14	0.25									
17.719	395.75	0.25									
17.739	396.42	0.25									
17.759	397.00	0.25									
17.779	397.53	0.25									
17.799	398.07	0.25									
17.819	398.64	0.25									
17.839	399.21	0.25									

17.859	399.78	0.25									
17.879	400.35	0.25									
17.899	400.92	0.25									
17.918	401.49	0.25									
17.938	402.04	0.25									
17.958	402.58	0.25									
17.978	403.12	0.25									
17.998	403.67	0.25									
18.018	404.22	0.25									
18.038	404.83	0.25									
18.058	405.38	0.25									
18.078	405.92	0.25									
18.098	406.51	0.25									
18.118	407.07	0.25									
18.138	407.63	0.25									
18.158	408.14	0.25									
18.178	408.69	0.25									
18.198	409.25	0.25									
18.218	409.83	0.25									
18.237	410.39	0.25									
18.257	410.95	0.25									
18.277	411.51	0.25									
18.297	412.06	0.25									
18.317	412.59	0.25									
18.337	413.14	0.25									
18.357	413.70	0.25									
18.377	414.22	0.25									
18.397	414.75	0.25									
18.417	415.31	0.25									
18.437	415.87	0.25									
18.457	416.42	0.25									
18.477	416.95	0.25									
18.497	417.48	0.25									
18.517	418.02	0.25									
18.537	418.55	0.25									
18.556	419.02	0.25									
18.576	419.49	0.25									
18.596	419.95	0.25									
18.616	420.50	0.25									
18.636	421.06	0.25									
18.656	421.60	0.25									
18.676	422.15	0.25									
18.696	422.73	0.25									
18.716	423.27	0.26									
18.736	423.79	0.26									

18.756	424.34	0.26									
18.776	424.88	0.26									
18.796	425.41	0.26									
18.816	425.92	0.26									
18.836	426.49	0.26									
18.856	427.03	0.26									
18.875	427.59	0.26									
18.895	428.13	0.26									
18.915	428.64	0.26									
18.935	429.20	0.26									
18.955	429.76	0.26									
18.975	430.32	0.26									
18.995	430.87	0.26									
19.015	431.40	0.26									
19.035	431.93	0.26									
19.055	432.43	0.26									
19.075	432.93	0.26									
19.095	433.48	0.26									
19.115	434.06	0.26									
19.135	434.60	0.26									
19.155	435.11	0.26									
19.174	435.60	0.26									
19.194	436.15	0.26									
19.214	436.70	0.26									
19.234	437.22	0.26									
19.254	437.72	0.26									
19.274	438.24	0.26									
19.294	438.80	0.26									
19.314	439.34	0.26									
19.334	439.85	0.26									
19.354	440.35	0.26									
19.374	440.88	0.26									
19.394	441.41	0.26									
19.414	441.93	0.26									
19.434	442.45	0.26									
19.454	442.97	0.26									
19.474	443.49	0.26									
19.493	444.00	0.26									
19.513	444.51	0.26									
19.533	445.02	0.26									
19.553	445.52	0.26									
19.573	446.03	0.26									
19.593	446.53	0.26									
19.613	447.08	0.26									
19.633	447.63	0.26									

19.653	448.14	0.26									
19.673	448.64	0.26									
19.693	449.13	0.26									
19.713	449.65	0.26									
19.733	450.17	0.26									
19.753	450.68	0.26									
19.773	451.12	0.26									
19.793	451.48	0.26									
19.812	452.03	0.26									
19.832	452.52	0.26									
19.852	453.03	0.26									
19.872	453.56	0.26									
19.892	454.08	0.26									
19.912	454.58	0.26									
19.932	455.10	0.26									
19.952	455.60	0.26									
19.972	456.11	0.26									
19.992	456.64	0.26									
20.012	457.16	0.26									
20.032	457.68	0.26									
20.052	458.18	0.26									
20.072	458.69	0.26									
20.092	459.19	0.26									
20.112	459.70	0.26									
20.131	460.21	0.26									
20.151	460.73	0.26									
20.171	461.23	0.26									
20.191	461.73	0.26									
20.211	462.23	0.26									
20.231	462.71	0.26									
20.251	463.21	0.26									
20.271	463.69	0.26									
20.291	464.19	0.26									
20.311	464.73	0.26									
20.331	465.20	0.26									
20.351	465.71	0.26									
20.371	466.23	0.26									
20.391	466.75	0.26									
20.411	467.23	0.26									
20.430	467.73	0.26									
20.450	468.23	0.26									
20.470	468.68	0.26									
20.490	469.17	0.26									
20.510	469.69	0.26									
20.530	470.20	0.26									

20.550	470.65	0.26									
20.570	471.12	0.26									
20.590	471.61	0.26									
20.610	472.10	0.26									
20.630	472.60	0.26									
20.650	473.11	0.26									
20.670	473.57	0.26									
20.690	474.04	0.26									
20.710	474.52	0.26									
20.730	475.04	0.26									
20.749	475.54	0.26									
20.769	475.98	0.26									
20.789	476.45	0.26									
20.809	476.92	0.26									
20.829	477.41	0.26									
20.849	477.91	0.26									
20.869	478.41	0.26									
20.889	478.84	0.26									
20.909	479.29	0.26									
20.929	479.78	0.26									
20.949	480.27	0.26									
20.969	480.75	0.26									
20.989	481.21	0.26									
21.009	481.67	0.26									
21.029	482.14	0.26									
21.049	482.60	0.27									
21.068	483.05	0.27									
21.088	483.50	0.27									
21.108	483.96	0.27									
21.128	484.47	0.27									
21.148	484.95	0.27									
21.168	485.38	0.27									
21.188	485.85	0.27									
21.208	486.33	0.27									
21.228	486.77	0.27									
21.248	487.19	0.27									
21.268	487.56	0.27									
21.288	487.94	0.27									
21.308	488.42	0.27									
21.328	488.94	0.27									
21.348	489.39	0.27									
21.368	489.84	0.27									
21.387	490.29	0.27									
21.407	490.75	0.27									
21.427	491.21	0.27									

21.447	491.70	0.27									
21.467	492.14	0.27									
21.487	492.59	0.27									
21.507	493.09	0.27									
21.527	493.52	0.27									
21.547	494.00	0.27									
21.567	494.46	0.27									
21.587	494.89	0.27									
21.607	495.36	0.27									
21.627	495.82	0.27									
21.647	496.28	0.27									
21.667	496.74	0.27									
21.686	497.14	0.27									
21.706	497.58	0.27									
21.726	498.04	0.27									
21.746	498.50	0.27									
21.766	498.95	0.27									
21.786	499.40	0.27									
21.806	499.84	0.27									
21.826	500.28	0.27									
21.846	500.72	0.27									
21.866	501.16	0.27									
21.886	501.58	0.27									
21.906	502.04	0.27									
21.926	502.52	0.27									
21.946	502.95	0.27									
21.966	503.41	0.27									
21.986	503.86	0.27									
22.005	504.21	0.27									
22.025	504.61	0.27									
22.045	505.05	0.27									
22.065	505.51	0.27									
22.085	505.92	0.27									
22.105	506.35	0.27									
22.125	506.81	0.27									
22.145	507.25	0.27									
22.165	507.68	0.27									
22.185	508.15	0.27									
22.205	508.54	0.27									
22.225	509.00	0.27									
22.245	509.46	0.27									
22.265	509.92	0.27									
22.285	510.35	0.27									
22.305	510.73	0.27									
22.324	511.21	0.27									

22.344	511.61	0.27									
22.364	512.02	0.27									
22.384	512.47	0.27									
22.404	512.92	0.27									
22.424	513.34	0.27									
22.444	513.74	0.27									
22.464	514.15	0.27									
22.484	514.61	0.27									
22.504	515.07	0.27									
22.524	515.51	0.27									
22.544	515.93	0.27									
22.564	516.34	0.27									
22.584	516.74	0.27									
22.604	517.18	0.27									
22.624	517.62	0.27									
22.643	518.06	0.27									
22.663	518.48	0.27									
22.683	518.90	0.27									
22.703	519.31	0.27									
22.723	519.72	0.27									
22.743	520.12	0.27									
22.763	520.52	0.27									
22.783	520.97	0.27									
22.803	521.36	0.27									
22.823	521.75	0.27									
22.843	522.20	0.27									
22.863	522.60	0.27									
22.883	523.00	0.27									
22.903	523.42	0.27									
22.923	523.83	0.27									
22.942	524.24	0.27									
22.962	524.68	0.27									
22.982	525.09	0.27									
23.002	525.48	0.27									
23.022	525.89	0.27									
23.042	526.29	0.27									
23.062	526.67	0.27									
23.082	527.10	0.27									
23.102	527.52	0.27									
23.122	527.91	0.27									
23.142	528.28	0.27									
23.162	528.65	0.27									
23.182	528.99	0.27									
23.202	529.37	0.27									
23.222	529.79	0.27									

23.242	530.25	0.27									
23.261	530.63	0.27									
23.281	531.04	0.27									
23.301	531.42	0.27									
23.321	531.80	0.27									
23.341	532.21	0.27									
23.361	532.64	0.27									
23.381	533.04	0.27									
23.401	533.42	0.27									
23.421	533.81	0.27									
23.441	534.25	0.27									
23.461	534.61	0.27									
23.481	534.98	0.27									
23.501	535.34	0.27									
23.521	535.73	0.27									
23.541	536.15	0.27									
23.561	536.58	0.27									
23.580	536.96	0.28									
23.600	537.39	0.28									
23.620	537.79	0.28									
23.640	538.17	0.28									
23.660	538.57	0.28									
23.680	538.95	0.28									
23.700	539.35	0.28									
23.720	539.75	0.28									
23.740	540.11	0.28									
23.760	540.47	0.28									
23.780	540.88	0.28									
23.800	541.26	0.28									
23.820	541.69	0.28									
23.840	542.10	0.28									
23.860	542.48	0.28									
23.880	542.86	0.28									
23.899	543.22	0.28									
23.919	543.58	0.28									
23.939	543.98	0.28									
23.959	544.41	0.28									
23.979	544.79	0.28									
23.999	545.17	0.28									
24.019	545.55	0.28									
24.039	545.94	0.28									
24.059	546.35	0.28									
24.079	546.71	0.28									
24.099	547.07	0.28									
24.119	547.45	0.28									

24.139	547.86	0.28									
24.159	548.20	0.28									
24.179	548.56	0.28									
24.198	548.95	0.28									
24.218	549.37	0.28									
24.238	549.73	0.28									
24.258	550.11	0.28									
24.278	550.49	0.28									
24.298	550.88	0.28									
24.318	551.22	0.28									
24.338	551.54	0.28									
24.358	551.92	0.28									
24.378	552.29	0.28									
24.398	552.67	0.28									
24.418	553.08	0.28									
24.438	553.47	0.28									
24.458	553.83	0.28									
24.478	554.21	0.28									
24.498	554.58	0.28									
24.517	554.93	0.28									
24.537	555.28	0.28									
24.557	555.63	0.28									
24.577	555.98	0.28									
24.597	556.34	0.28									
24.617	556.71	0.28									
24.637	557.11	0.28									
24.657	557.47	0.28									
24.677	557.81	0.28									
24.697	558.16	0.28									
24.717	558.53	0.28									
24.737	558.93	0.28									
24.757	559.28	0.28									
24.777	559.62	0.28									
24.797	560.00	0.28									
24.817	560.37	0.28									
24.836	560.73	0.28									
24.856	561.06	0.28									
24.876	561.37	0.28									
24.896	561.68	0.28									
24.916	562.01	0.28									
24.936	562.39	0.28									
24.956	562.76	0.28									
24.976	563.13	0.28									
24.996	563.49	0.28									
25.016	563.85	0.28									

25.036	564.20	0.28									
25.056	564.55	0.28									
25.076	564.90	0.28									
25.096	565.23	0.28									
25.116	565.58	0.28									
25.136	565.94	0.28									
25.155	566.31	0.28									
25.175	566.66	0.28									
25.195	566.99	0.28									
25.215	567.30	0.28									
25.235	567.60	0.28									
25.255	568.00	0.28									
25.275	568.38	0.28									
25.295	568.72	0.28									
25.315	569.05	0.28									
25.335	569.39	0.28									
25.355	569.79	0.28									
25.375	570.12	0.28									
25.395	570.47	0.28									
25.415	570.81	0.28									
25.435	571.14	0.28									
25.454	571.51	0.28									
25.474	571.88	0.28									
25.494	572.22	0.28									
25.514	572.54	0.28									
25.534	572.91	0.28									
25.554	573.25	0.28									
25.574	573.60	0.28									
25.594	573.93	0.28									
25.614	574.24	0.28									
25.634	574.61	0.28									
25.654	574.97	0.28									
25.674	575.32	0.28									
25.694	575.67	0.28									
25.714	575.96	0.28									
25.734	576.30	0.28									
25.754	576.64	0.28									
25.773	576.96	0.28									
25.793	577.30	0.28									
25.813	577.64	0.28									
25.833	577.99	0.28									
25.853	578.37	0.28									
25.873	578.68	0.28									
25.893	578.96	0.28									
25.913	579.35	0.28									

25.933	579.71	0.28									
25.953	580.03	0.28									
25.973	580.30	0.28									
25.993	580.63	0.28									
26.013	580.98	0.28									
26.033	581.31	0.28									
26.053	581.68	0.28									
26.073	582.03	0.28									
26.092	582.35	0.28									
26.112	582.67	0.28									
26.132	583.00	0.28									
26.152	583.33	0.28									
26.172	583.65	0.28									
26.192	583.96	0.28									
26.212	584.28	0.28									
26.232	584.58	0.28									
26.252	584.91	0.28									
26.272	585.27	0.28									
26.292	585.58	0.28									
26.312	585.91	0.28									
26.332	586.25	0.28									
26.352	586.56	0.28									
26.372	586.86	0.28									
26.392	587.20	0.28									
26.411	587.53	0.28									
26.431	587.86	0.29									
26.451	588.15	0.29									
26.471	588.42	0.29									
26.491	588.69	0.29									
26.511	588.99	0.29									
26.531	589.35	0.29									
26.551	589.69	0.29									
26.571	589.94	0.29									
26.591	590.30	0.29									
26.611	590.65	0.29									
26.631	590.97	0.29									
26.651	591.26	0.29									
26.671	591.55	0.29									
26.691	591.88	0.29									
26.710	592.22	0.29									
26.730	592.53	0.29									
26.750	592.86	0.29									
26.770	593.16	0.29									
26.790	593.43	0.29									
26.810	593.76	0.29									

26.830	594.09	0.29									
26.850	594.45	0.29									
26.870	594.79	0.29									
26.890	595.09	0.29									
26.910	595.39	0.29									
26.930	595.71	0.29									
26.950	595.99	0.29									
26.970	596.28	0.29									
26.990	596.60	0.29									
27.010	596.91	0.29									
27.029	597.24	0.29									
27.049	597.57	0.29									
27.069	597.89	0.29									
27.089	598.20	0.29									
27.109	598.50	0.29									
27.129	598.80	0.29									
27.149	599.10	0.29									
27.169	599.38	0.29									
27.189	599.73	0.29									
27.209	600.03	0.29									
27.229	600.32	0.29									
27.249	600.61	0.29									
27.269	600.96	0.29									
27.289	601.28	0.29									
27.309	601.57	0.29									
27.329	601.84	0.29									
27.348	602.15	0.29									
27.368	602.47	0.29									
27.388	602.80	0.29									
27.408	603.09	0.29									
27.428	603.37	0.29									
27.448	603.69	0.29									
27.468	603.96	0.29									
27.488	604.23	0.29									
27.508	604.55	0.29									
27.528	604.83	0.29									
27.548	605.11	0.29									
27.568	605.43	0.29									
27.588	605.75	0.29									
27.608	606.06	0.29									
27.628	606.33	0.29									
27.647	606.61	0.29									
27.667	606.89	0.29									
27.687	607.20	0.29									
27.707	607.49	0.29									

27.727	607.77	0.29									
27.747	608.08	0.29									
27.767	608.36	0.29									
27.787	608.63	0.29									
27.807	608.94	0.29									
27.827	609.24	0.29									
27.847	609.54	0.29									
27.867	609.84	0.29									
27.887	610.13	0.29									
27.907	610.43	0.29									
27.927	610.71	0.29									
27.947	611.00	0.29									
27.966	611.29	0.29									
27.986	611.57	0.29									
28.006	611.85	0.29									
28.026	612.13	0.29									
28.046	612.40	0.29									
28.066	612.62	0.29									
28.086	612.88	0.29									
28.106	613.18	0.29									
28.126	613.45	0.29									
28.146	613.73	0.29									
28.166	614.01	0.29									
28.186	614.38	0.29									
28.206	614.65	0.29									
28.226	614.92	0.29									
28.246	615.20	0.29									
28.266	615.48	0.29									
28.285	615.75	0.29									
28.305	616.04	0.29									
28.325	616.35	0.29									
28.345	616.63	0.29									
28.365	616.94	0.29									
28.385	617.23	0.29									
28.405	617.49	0.29									
28.425	617.75	0.29									
28.445	618.06	0.29									
28.465	618.33	0.29									
28.485	618.60	0.29									
28.505	618.88	0.29									
28.525	619.19	0.29									
28.545	619.46	0.29									
28.565	619.76	0.29									
28.585	620.05	0.29									
28.604	620.32	0.29									

28.624	620.63	0.29									
28.644	620.87	0.29									
28.664	621.16	0.29									
28.684	621.46	0.29									
28.704	621.76	0.29									
28.724	622.00	0.29									
28.744	622.23	0.29									
28.764	622.51	0.29									
28.784	622.86	0.29									
28.804	623.10	0.29									
28.824	623.36	0.29									
28.844	623.64	0.29									
28.864	623.95	0.29									
28.884	624.19	0.29									
28.903	624.45	0.29									
28.923	624.75	0.29									
28.943	625.04	0.29									
28.963	625.28	0.29									
28.983	625.53	0.29									
29.003	625.82	0.29									
29.023	626.11	0.29									
29.043	626.40	0.29									
29.063	626.68	0.29									
29.083	626.90	0.29									
29.103	627.17	0.29									
29.123	627.45	0.29									
29.143	627.74	0.29									
29.163	628.01	0.29									
29.183	628.28	0.29									
29.203	628.56	0.29									
29.222	628.81	0.29									
29.242	629.08	0.29									
29.262	629.36	0.29									
29.282	629.63	0.29									
29.302	629.89	0.29									
29.322	630.15	0.29									
29.342	630.43	0.29									
29.362	630.70	0.29									
29.382	630.95	0.29									
29.402	631.20	0.29									
29.422	631.47	0.29									
29.442	631.77	0.29									
29.462	632.03	0.29									
29.482	632.27	0.29									
29.502	632.51	0.29									

29.522	632.77	0.29									
29.541	633.06	0.29									
29.561	633.31	0.29									
29.581	633.56	0.29									
29.601	633.79	0.29									
29.621	634.05	0.29									
29.641	634.33	0.29									
29.661	634.58	0.29									
29.681	634.83	0.29									
29.701	635.11	0.29									
29.721	635.37	0.29									
29.741	635.60	0.29									
29.761	635.87	0.29									
29.781	636.15	0.29									
29.801	636.41	0.30									
29.821	636.67	0.30									
29.841	636.93	0.30									
29.860	637.17	0.30									
29.880	637.39	0.30									
29.900	637.63	0.30									
29.920	637.89	0.30									
29.940	638.15	0.30									
29.960	638.41	0.30									
29.980	638.67	0.30									
30.000	638.92	0.30									

Combined standard uncertainties:

$u(T)$= 0.006 K; $u(p)$= 0.0020 MPa for p < 6 MPa; $u(p)$= 0.024 MPa for 6 MPa ≤ p ≤ 70 MPa

$u(x_{CO_2})$= 0.00024; $u(x_{O_2})$= 0.000030; $u(x_{SO_2})$= 0.0000023; $u(x_{CO})$= 0.0000043

Table S1 (continued). $p\rho T$ experimental data for the $CO_2 + O_2 + SO_2 + CO$ (Mix 1) and $CO_2 + NO + SO_2 + CO$ (Mix 2) mixtures. $u(\rho)$: combined standard uncertainty.

**Mix 2: x_{CO_2} = 0.99592; x_{NO} = 0.001410; x_{SO_2} = 0.0009100; x_{CO} = 0.0017002**											
***T*= 303.16±0.02 K**			***T*= 313.13±0.02 K**			***T*= 333.14±0.02 K**			***T*= 353.17±0.02 K**		
p (MPa)	ρ (kg.m^{-3})	$u(\rho)$ (kg.m^{-3})	p (MPa)	ρ (kg.m^{-3})	$u(\rho)$ (kg.m^{-3})	p (MPa)	ρ (kg.m^{-3})	$u(\rho)$ (kg.m^{-3})	p (MPa)	ρ (kg.m^{-3})	$u(\rho)$ (kg.m^{-3})
0.122	2.05	0.21	0.111	1.73	0.22	0.113	1.71	0.22	0.120	1.72	0.20
0.142	2.50	0.21	0.131	2.04	0.22	0.133	2.04	0.22	0.139	2.04	0.21
0.162	2.78	0.21	0.150	2.36	0.22	0.153	2.37	0.22	0.159	2.36	0.21
0.182	3.12	0.21	0.170	2.73	0.22	0.173	2.68	0.22	0.179	2.65	0.21
0.202	3.48	0.22	0.190	3.07	0.22	0.193	2.99	0.22	0.199	2.94	0.21
0.221	3.83	0.22	0.210	3.38	0.22	0.213	3.30	0.22	0.219	3.23	0.21
0.241	4.19	0.22	0.230	3.72	0.22	0.233	3.62	0.22	0.239	3.52	0.21
0.261	4.55	0.22	0.250	4.10	0.22	0.253	3.93	0.22	0.259	3.81	0.21
0.281	4.91	0.22	0.270	4.45	0.22	0.273	4.24	0.22	0.279	4.09	0.22
0.301	5.26	0.22	0.290	4.78	0.22	0.293	4.56	0.22	0.299	4.37	0.22
0.321	5.60	0.22	0.310	5.13	0.22	0.312	4.87	0.22	0.319	4.75	0.22
0.341	5.94	0.22	0.330	5.49	0.22	0.332	5.18	0.22	0.339	5.01	0.22
0.360	6.29	0.22	0.350	5.86	0.22	0.352	5.51	0.22	0.359	5.32	0.22
0.380	6.64	0.22	0.369	6.23	0.22	0.372	5.84	0.22	0.379	5.60	0.22
0.400	6.98	0.22	0.389	6.57	0.22	0.392	6.15	0.22	0.399	5.89	0.22
0.420	7.33	0.22	0.409	6.92	0.22	0.412	6.46	0.22	0.419	6.17	0.22
0.440	7.68	0.22	0.429	7.26	0.22	0.432	6.76	0.22	0.438	6.46	0.22
0.460	8.06	0.22	0.449	7.56	0.22	0.452	7.07	0.22	0.458	6.74	0.22
0.480	8.41	0.22	0.469	7.88	0.22	0.472	7.38	0.22	0.478	7.03	0.22
0.499	8.75	0.22	0.489	8.28	0.22	0.492	7.69	0.22	0.498	7.32	0.22
0.519	9.14	0.22	0.509	8.64	0.22	0.512	8.00	0.22	0.518	7.62	0.22
0.539	9.50	0.22	0.529	8.97	0.22	0.532	8.34	0.22	0.538	7.89	0.22
0.559	9.86	0.22	0.549	9.32	0.22	0.552	8.63	0.22	0.558	8.22	0.22
0.579	10.17	0.22	0.569	9.70	0.22	0.572	8.98	0.22	0.578	8.56	0.22
0.599	10.55	0.22	0.588	10.04	0.22	0.592	9.30	0.22	0.598	8.87	0.22
0.618	10.90	0.22	0.608	10.44	0.22	0.612	9.61	0.22	0.618	9.17	0.22
0.638	11.28	0.22	0.628	10.81	0.22	0.631	9.95	0.22	0.638	9.48	0.22
0.658	11.64	0.22	0.648	11.15	0.22	0.651	10.31	0.22	0.658	9.78	0.22
0.678	12.01	0.22	0.668	11.48	0.22	0.671	10.65	0.22	0.678	10.05	0.22
0.698	12.37	0.22	0.688	11.82	0.22	0.691	10.97	0.22	0.698	10.38	0.22
0.718	12.74	0.22	0.708	12.17	0.22	0.711	11.30	0.22	0.718	10.70	0.22
0.738	13.10	0.22	0.728	12.54	0.22	0.731	11.63	0.22	0.737	11.00	0.22
0.757	13.45	0.22	0.748	12.91	0.22	0.751	11.99	0.22	0.757	11.29	0.22
0.777	13.90	0.22	0.768	13.23	0.22	0.771	12.30	0.22	0.777	11.58	0.22
0.797	14.27	0.22	0.788	13.61	0.22	0.791	12.66	0.22	0.797	11.91	0.22

0.817	14.63	0.22	0.807	13.99	0.22	0.811	12.99	0.22	0.817	12.23	0.22
0.837	15.00	0.22	0.827	14.36	0.22	0.831	13.30	0.22	0.837	12.53	0.22
0.857	15.37	0.22	0.847	14.72	0.22	0.851	13.64	0.22	0.857	12.87	0.22
0.877	15.73	0.22	0.867	15.05	0.22	0.871	13.97	0.22	0.877	13.22	0.22
0.896	16.18	0.22	0.887	15.44	0.22	0.891	14.32	0.22	0.897	13.55	0.22
0.916	16.55	0.22	0.907	15.78	0.22	0.911	14.66	0.22	0.917	13.87	0.22
0.936	16.92	0.22	0.927	16.14	0.22	0.931	15.00	0.22	0.937	14.18	0.22
0.956	17.29	0.22	0.947	16.52	0.22	0.950	15.33	0.22	0.957	14.48	0.22
0.976	17.73	0.22	0.967	16.89	0.22	0.970	15.67	0.22	0.977	14.77	0.22
0.996	18.05	0.22	0.987	17.29	0.22	0.990	16.00	0.22	0.997	15.07	0.22
1.016	18.47	0.22	1.007	17.66	0.22	1.010	16.30	0.22	1.017	15.41	0.22
1.035	18.79	0.22	1.026	18.04	0.22	1.030	16.63	0.22	1.036	15.74	0.22
1.055	19.20	0.22	1.046	18.42	0.22	1.050	17.05	0.22	1.056	16.04	0.22
1.075	19.61	0.22	1.066	18.77	0.22	1.070	17.39	0.22	1.076	16.36	0.22
1.095	20.00	0.22	1.086	19.10	0.22	1.090	17.73	0.22	1.096	16.68	0.22
1.115	20.44	0.22	1.106	19.50	0.22	1.110	18.09	0.22	1.116	17.03	0.22
1.135	20.81	0.22	1.126	19.89	0.22	1.130	18.45	0.22	1.136	17.39	0.22
1.155	21.19	0.22	1.146	20.24	0.22	1.150	18.75	0.22	1.156	17.70	0.22
1.174	21.57	0.22	1.166	20.60	0.22	1.170	19.09	0.22	1.176	17.97	0.22
1.194	22.02	0.22	1.186	21.00	0.22	1.190	19.45	0.22	1.196	18.28	0.22
1.214	22.40	0.22	1.206	21.36	0.22	1.210	19.81	0.22	1.216	18.62	0.22
1.234	22.78	0.22	1.226	21.71	0.22	1.230	20.13	0.22	1.236	18.93	0.22
1.254	23.17	0.22	1.245	22.08	0.22	1.250	20.47	0.22	1.256	19.23	0.22
1.274	23.54	0.22	1.265	22.48	0.22	1.270	20.84	0.22	1.276	19.59	0.22
1.293	23.93	0.22	1.285	22.88	0.22	1.289	21.19	0.22	1.296	19.91	0.22
1.313	24.38	0.22	1.305	23.26	0.22	1.309	21.53	0.22	1.316	20.19	0.22
1.333	24.78	0.22	1.325	23.63	0.22	1.329	21.90	0.22	1.335	20.55	0.22
1.353	25.18	0.22	1.345	24.00	0.22	1.349	22.24	0.22	1.355	20.87	0.22
1.373	25.56	0.22	1.365	24.39	0.22	1.369	22.56	0.22	1.375	21.18	0.22
1.393	25.95	0.22	1.385	24.78	0.22	1.389	22.93	0.22	1.395	21.54	0.22
1.413	26.38	0.22	1.405	25.16	0.22	1.409	23.31	0.22	1.415	21.83	0.22
1.432	26.80	0.22	1.425	25.54	0.22	1.429	23.63	0.22	1.435	22.18	0.22
1.452	27.19	0.22	1.445	25.93	0.22	1.449	23.96	0.22	1.455	22.48	0.22
1.472	27.59	0.22	1.464	26.32	0.22	1.469	24.33	0.22	1.475	22.81	0.22
1.492	27.99	0.22	1.484	26.70	0.22	1.489	24.69	0.22	1.495	23.16	0.22
1.512	28.38	0.22	1.504	27.09	0.22	1.509	25.06	0.22	1.515	23.48	0.22
1.532	28.77	0.22	1.524	27.50	0.22	1.529	25.40	0.22	1.535	23.79	0.22
1.552	29.24	0.22	1.544	27.86	0.22	1.549	25.72	0.22	1.555	24.11	0.22
1.571	29.64	0.22	1.564	28.22	0.22	1.569	26.11	0.22	1.575	24.46	0.22
1.591	30.04	0.22	1.584	28.62	0.22	1.589	26.51	0.22	1.595	24.79	0.22
1.611	30.44	0.22	1.604	29.04	0.22	1.608	26.85	0.22	1.615	25.12	0.22
1.631	30.85	0.22	1.624	29.43	0.22	1.628	27.20	0.22	1.634	25.47	0.22
1.651	31.25	0.22	1.644	29.80	0.22	1.648	27.57	0.22	1.654	25.77	0.22
1.671	31.65	0.22	1.664	30.20	0.22	1.668	27.92	0.22	1.674	26.11	0.22
1.691	32.11	0.22	1.683	30.60	0.22	1.688	28.25	0.22	1.694	26.46	0.22

1.710	32.50	0.22	1.703	30.98	0.22	1.708	28.61	0.22	1.714	26.78	0.22
1.730	32.90	0.22	1.723	31.35	0.22	1.728	28.99	0.22	1.734	27.11	0.22
1.750	33.34	0.22	1.743	31.79	0.22	1.748	29.37	0.22	1.754	27.46	0.22
1.770	33.75	0.22	1.763	32.19	0.22	1.768	29.69	0.22	1.774	27.79	0.22
1.790	34.14	0.22	1.783	32.58	0.22	1.788	30.05	0.22	1.794	28.11	0.22
1.810	34.56	0.22	1.803	33.00	0.22	1.808	30.42	0.22	1.814	28.43	0.22
1.830	34.99	0.22	1.823	33.39	0.22	1.828	30.80	0.22	1.834	28.74	0.22
1.849	35.41	0.22	1.843	33.78	0.22	1.848	31.12	0.22	1.854	29.10	0.22
1.869	35.81	0.22	1.863	34.18	0.22	1.868	31.47	0.22	1.874	29.42	0.22
1.889	36.26	0.22	1.883	34.59	0.22	1.888	31.86	0.22	1.894	29.74	0.22
1.909	36.67	0.22	1.902	35.01	0.22	1.908	32.23	0.22	1.914	30.08	0.22
1.929	37.12	0.22	1.922	35.39	0.22	1.927	32.57	0.22	1.933	30.45	0.22
1.949	37.54	0.22	1.942	35.76	0.22	1.947	32.98	0.22	1.953	30.76	0.22
1.968	37.94	0.22	1.962	36.18	0.22	1.967	33.33	0.22	1.973	31.11	0.22
1.988	38.40	0.22	1.982	36.60	0.22	1.987	33.71	0.22	1.993	31.45	0.22
2.008	38.82	0.22	2.002	36.99	0.22	2.007	34.08	0.22	2.013	31.77	0.22
2.028	39.23	0.22	2.022	37.40	0.22	2.027	34.44	0.22	2.033	32.12	0.22
2.048	39.66	0.22	2.042	37.85	0.22	2.047	34.80	0.22	2.053	32.42	0.22
2.068	40.11	0.22	2.062	38.26	0.22	2.067	35.16	0.22	2.073	32.72	0.22
2.088	40.59	0.22	2.082	38.68	0.22	2.087	35.52	0.22	2.093	33.03	0.22
2.107	40.99	0.22	2.102	39.09	0.22	2.107	35.91	0.22	2.113	33.44	0.22
2.127	41.41	0.22	2.121	39.46	0.22	2.127	36.25	0.22	2.133	33.82	0.22
2.147	41.86	0.22	2.141	39.88	0.22	2.147	36.64	0.22	2.153	34.15	0.22
2.167	42.31	0.22	2.161	40.30	0.22	2.167	37.04	0.22	2.173	34.46	0.22
2.187	42.76	0.22	2.181	40.72	0.22	2.187	37.41	0.22	2.193	34.80	0.22
2.207	43.20	0.22	2.201	41.13	0.22	2.207	37.76	0.22	2.213	35.18	0.22
2.227	43.66	0.22	2.221	41.55	0.22	2.227	38.16	0.22	2.232	35.54	0.22
2.246	44.11	0.22	2.241	41.96	0.22	2.246	38.54	0.22	2.252	35.87	0.22
2.266	44.56	0.22	2.261	42.38	0.22	2.266	38.88	0.22	2.272	36.22	0.22
2.286	45.01	0.22	2.281	42.82	0.22	2.286	39.28	0.22	2.292	36.57	0.22
2.306	45.46	0.22	2.301	43.27	0.22	2.306	39.69	0.22	2.312	36.88	0.22
2.326	45.91	0.22	2.321	43.64	0.22	2.326	40.07	0.22	2.332	37.23	0.22
2.346	46.37	0.22	2.340	44.06	0.22	2.346	40.46	0.22	2.352	37.56	0.22
2.366	46.81	0.22	2.360	44.50	0.22	2.366	40.80	0.22	2.372	37.89	0.22
2.385	47.26	0.22	2.380	44.95	0.22	2.386	41.18	0.22	2.392	38.26	0.22
2.405	47.72	0.22	2.400	45.40	0.22	2.406	41.56	0.22	2.412	38.63	0.22
2.425	48.17	0.22	2.420	45.78	0.22	2.426	41.94	0.22	2.432	38.95	0.22
2.445	48.62	0.22	2.440	46.19	0.22	2.446	42.31	0.22	2.452	39.28	0.22
2.465	49.09	0.22	2.460	46.62	0.22	2.466	42.69	0.22	2.472	39.64	0.22
2.485	49.55	0.22	2.480	47.05	0.22	2.486	43.07	0.22	2.492	40.01	0.22
2.505	50.01	0.22	2.500	47.49	0.22	2.506	43.44	0.22	2.512	40.32	0.22
2.524	50.47	0.22	2.520	47.92	0.22	2.526	43.85	0.22	2.531	40.67	0.22
2.544	50.94	0.22	2.540	48.36	0.22	2.546	44.29	0.22	2.551	41.03	0.22
2.564	51.40	0.22	2.559	48.82	0.22	2.565	44.66	0.22	2.571	41.38	0.22
2.584	51.87	0.22	2.579	49.27	0.22	2.585	45.02	0.22	2.591	41.74	0.22

2.604	52.34	0.22	2.599	49.66	0.22	2.605	45.41	0.22	2.611	42.10	0.22
2.624	52.81	0.22	2.619	50.10	0.22	2.625	45.80	0.22	2.631	42.43	0.22
2.643	53.29	0.22	2.639	50.55	0.22	2.645	46.14	0.22	2.651	42.76	0.22
2.663	53.76	0.22	2.659	51.00	0.22	2.665	46.51	0.22	2.671	43.12	0.22
2.683	54.23	0.22	2.679	51.44	0.22	2.685	46.92	0.22	2.691	43.48	0.22
2.703	54.69	0.22	2.699	51.86	0.22	2.705	47.36	0.22	2.711	43.82	0.22
2.723	55.15	0.22	2.719	52.33	0.22	2.725	47.78	0.22	2.731	44.16	0.22
2.743	55.64	0.22	2.739	52.80	0.22	2.745	48.15	0.22	2.751	44.52	0.22
2.763	56.13	0.22	2.759	53.22	0.22	2.765	48.53	0.22	2.771	44.89	0.22
2.782	56.61	0.22	2.778	53.65	0.22	2.785	48.93	0.22	2.791	45.24	0.22
2.802	57.09	0.22	2.798	54.11	0.22	2.805	49.33	0.22	2.811	45.61	0.22
2.822	57.58	0.22	2.818	54.57	0.22	2.825	49.70	0.22	2.830	45.96	0.22
2.842	58.06	0.22	2.838	55.02	0.22	2.845	50.11	0.22	2.850	46.29	0.22
2.862	58.55	0.22	2.858	55.49	0.22	2.865	50.51	0.22	2.870	46.67	0.22
2.882	59.04	0.22	2.878	55.94	0.22	2.884	50.91	0.22	2.890	47.01	0.22
2.902	59.53	0.22	2.898	56.39	0.22	2.904	51.31	0.22	2.910	47.36	0.22
2.921	60.02	0.22	2.918	56.84	0.22	2.924	51.73	0.22	2.930	47.74	0.22
2.941	60.49	0.22	2.938	57.28	0.22	2.944	52.09	0.22	2.950	48.10	0.22
2.961	60.97	0.22	2.958	57.75	0.22	2.964	52.48	0.22	2.970	48.41	0.22
2.981	61.49	0.22	2.978	58.19	0.22	2.984	52.89	0.22	2.990	48.82	0.22
3.001	61.99	0.22	2.997	58.66	0.22	3.004	53.32	0.22	3.010	49.20	0.22
3.021	62.46	0.22	3.017	59.12	0.22	3.024	53.70	0.22	3.030	49.54	0.22
3.041	62.97	0.22	3.037	59.57	0.22	3.044	54.09	0.22	3.050	49.84	0.22
3.060	63.47	0.22	3.057	60.04	0.22	3.064	54.51	0.22	3.070	50.24	0.22
3.080	63.97	0.22	3.077	60.50	0.22	3.084	54.93	0.22	3.090	50.61	0.22
3.100	64.48	0.22	3.097	60.96	0.22	3.104	55.32	0.22	3.110	50.96	0.22
3.120	64.98	0.22	3.117	61.42	0.22	3.124	55.74	0.22	3.129	51.31	0.22
3.140	65.49	0.22	3.137	61.89	0.22	3.144	56.13	0.22	3.149	51.68	0.22
3.160	66.01	0.22	3.157	62.36	0.22	3.164	56.54	0.22	3.169	52.05	0.22
3.180	66.51	0.22	3.177	62.88	0.22	3.184	56.97	0.22	3.189	52.41	0.22
3.199	67.04	0.22	3.197	63.33	0.22	3.203	57.39	0.22	3.209	52.76	0.22
3.219	67.58	0.22	3.216	63.77	0.22	3.223	57.76	0.22	3.229	53.13	0.22
3.239	68.08	0.23	3.236	64.26	0.22	3.243	58.17	0.22	3.249	53.50	0.22
3.259	68.59	0.22	3.256	64.75	0.22	3.263	58.60	0.22	3.269	53.86	0.22
3.279	69.09	0.23	3.276	65.20	0.22	3.283	59.03	0.22	3.289	54.21	0.22
3.299	69.62	0.22	3.296	65.65	0.22	3.303	59.47	0.22	3.309	54.59	0.22
3.318	70.15	0.23	3.316	66.15	0.22	3.323	59.89	0.22	3.329	54.98	0.22
3.338	70.67	0.23	3.336	66.66	0.22	3.343	60.31	0.22	3.349	55.36	0.22
3.358	71.20	0.23	3.356	67.14	0.22	3.363	60.69	0.22	3.369	55.72	0.22
3.378	71.72	0.23	3.376	67.62	0.22	3.383	61.07	0.22	3.389	56.13	0.22
3.398	72.25	0.23	3.396	68.11	0.22	3.403	61.54	0.22	3.409	56.48	0.22
3.418	72.79	0.23	3.416	68.55	0.22	3.423	61.94	0.22	3.428	56.82	0.22
3.438	73.33	0.23	3.435	69.05	0.22	3.443	62.34	0.22	3.448	57.18	0.22
3.457	73.85	0.23	3.455	69.58	0.23	3.463	62.78	0.22	3.468	57.60	0.22
3.477	74.39	0.23	3.475	70.07	0.23	3.483	63.22	0.22	3.488	57.97	0.22

3.497	74.95	0.23	3.495	70.50	0.23	3.503	63.60	0.22	3.508	58.33	0.22
3.517	75.48	0.23	3.515	71.02	0.23	3.522	64.01	0.22	3.528	58.70	0.22
3.537	76.04	0.23	3.535	71.51	0.23	3.542	64.45	0.22	3.548	59.11	0.22
3.557	76.57	0.23	3.555	72.02	0.23	3.562	64.89	0.22	3.568	59.46	0.22
3.577	77.12	0.23	3.575	72.54	0.23	3.582	65.29	0.22	3.588	59.83	0.22
3.596	77.69	0.23	3.595	73.04	0.23	3.602	65.75	0.22	3.608	60.23	0.22
3.616	78.24	0.23	3.615	73.49	0.23	3.622	66.19	0.22	3.628	60.59	0.22
3.636	78.81	0.23	3.635	74.00	0.23	3.642	66.60	0.22	3.648	60.94	0.22
3.656	79.35	0.23	3.654	74.52	0.23	3.662	67.01	0.22	3.668	61.33	0.22
3.676	79.90	0.23	3.674	75.04	0.23	3.682	67.44	0.22	3.688	61.74	0.22
3.696	80.47	0.23	3.694	75.54	0.23	3.702	67.84	0.22	3.708	62.08	0.22
3.716	81.04	0.23	3.714	76.06	0.23	3.722	68.30	0.22	3.727	62.47	0.22
3.735	81.61	0.23	3.734	76.57	0.23	3.742	68.74	0.22	3.747	62.85	0.22
3.755	82.14	0.23	3.754	77.07	0.23	3.762	69.16	0.22	3.767	63.21	0.22
3.775	82.68	0.23	3.774	77.58	0.23	3.782	69.60	0.22	3.787	63.59	0.22
3.795	83.26	0.23	3.794	78.10	0.23	3.802	70.05	0.22	3.807	63.97	0.22
3.815	83.83	0.23	3.814	78.61	0.23	3.822	70.46	0.22	3.827	64.36	0.22
3.835	84.43	0.23	3.834	79.15	0.23	3.841	70.92	0.22	3.847	64.76	0.22
3.855	85.03	0.23	3.854	79.67	0.23	3.861	71.38	0.22	3.867	65.13	0.22
3.874	85.59	0.23	3.873	80.18	0.23	3.881	71.79	0.22	3.887	65.50	0.22
3.894	86.18	0.23	3.893	80.70	0.23	3.901	72.23	0.22	3.907	65.87	0.22
3.914	86.79	0.23	3.913	81.23	0.23	3.921	72.69	0.22	3.927	66.24	0.22
3.934	87.35	0.23	3.933	81.75	0.23	3.941	73.13	0.22	3.947	66.62	0.22
3.954	87.92	0.23	3.953	82.28	0.23	3.961	73.57	0.22	3.967	67.04	0.22
3.974	88.51	0.23	3.973	82.79	0.23	3.981	73.99	0.22	3.987	67.43	0.22
3.993	89.12	0.23	3.993	83.30	0.23	4.001	74.43	0.22	4.007	67.82	0.22
4.013	89.70	0.23	4.013	83.83	0.23	4.021	74.82	0.22	4.026	68.22	0.22
4.033	90.32	0.23	4.033	84.43	0.23	4.041	75.28	0.22	4.046	68.59	0.22
4.053	90.93	0.23	4.053	84.95	0.23	4.061	75.73	0.22	4.066	68.97	0.22
4.073	91.53	0.23	4.073	85.46	0.23	4.081	76.17	0.22	4.086	69.35	0.22
4.093	92.15	0.23	4.092	86.00	0.23	4.101	76.62	0.22	4.106	69.73	0.22
4.113	92.74	0.23	4.112	86.55	0.23	4.121	77.07	0.22	4.126	70.13	0.22
4.132	93.34	0.23	4.132	87.07	0.23	4.141	77.52	0.22	4.146	70.56	0.22
4.152	93.96	0.23	4.152	87.64	0.23	4.160	77.93	0.22	4.166	70.93	0.22
4.172	94.58	0.23	4.172	88.20	0.23	4.180	78.36	0.22	4.186	71.31	0.22
4.192	95.21	0.23	4.192	88.73	0.23	4.200	78.82	0.22	4.206	71.71	0.22
4.212	95.81	0.23	4.212	89.26	0.23	4.220	79.27	0.22	4.226	72.11	0.22
4.232	96.43	0.23	4.232	89.81	0.23	4.240	79.72	0.22	4.246	72.47	0.22
4.252	97.04	0.23	4.252	90.39	0.23	4.260	80.18	0.22	4.266	72.89	0.22
4.271	97.67	0.23	4.272	90.95	0.23	4.280	80.65	0.22	4.286	73.30	0.22
4.291	98.33	0.23	4.292	91.50	0.23	4.300	81.13	0.22	4.306	73.70	0.22
4.311	98.97	0.23	4.312	92.06	0.23	4.320	81.60	0.22	4.325	74.06	0.22
4.331	99.62	0.23	4.331	92.62	0.23	4.340	82.04	0.22	4.345	74.43	0.22
4.351	100.26	0.23	4.351	93.21	0.23	4.360	82.48	0.22	4.365	74.84	0.22
4.371	100.92	0.23	4.371	93.78	0.23	4.380	82.94	0.22	4.385	75.26	0.22

4.391	101.57	0.23	4.391	94.33	0.23	4.400	83.41	0.22	4.405	75.68	0.22
4.410	102.22	0.23	4.411	94.89	0.23	4.420	83.87	0.22	4.425	76.09	0.22
4.430	102.88	0.23	4.431	95.43	0.23	4.440	84.34	0.22	4.445	76.47	0.22
4.450	103.54	0.23	4.451	96.02	0.23	4.460	84.81	0.22	4.465	76.86	0.22
4.470	104.20	0.23	4.471	96.62	0.23	4.480	85.29	0.22	4.485	77.27	0.22
4.490	104.87	0.23	4.491	97.21	0.23	4.499	85.76	0.22	4.505	77.67	0.22
4.510	105.52	0.23	4.511	97.76	0.23	4.519	86.20	0.22	4.525	78.07	0.22
4.530	106.18	0.23	4.531	98.36	0.23	4.539	86.67	0.22	4.545	78.45	0.22
4.549	106.85	0.23	4.550	98.97	0.23	4.559	87.14	0.22	4.565	78.83	0.22
4.569	107.54	0.23	4.570	99.57	0.23	4.579	87.62	0.22	4.585	79.27	0.22
4.589	108.22	0.23	4.590	100.09	0.23	4.599	88.11	0.22	4.605	79.70	0.22
4.609	108.90	0.23	4.610	100.70	0.23	4.619	88.59	0.22	4.625	80.09	0.22
4.629	109.60	0.23	4.630	101.31	0.23	4.639	89.06	0.22	4.644	80.48	0.22
4.649	110.29	0.23	4.650	101.93	0.23	4.659	89.52	0.22	4.664	80.90	0.22
4.668	110.98	0.23	4.670	102.53	0.23	4.679	90.00	0.22	4.684	81.33	0.22
4.688	111.66	0.23	4.690	103.09	0.23	4.699	90.47	0.22	4.704	81.71	0.22
4.708	112.35	0.23	4.710	103.67	0.23	4.719	90.94	0.22	4.724	82.10	0.22
4.728	113.08	0.23	4.730	104.27	0.23	4.739	91.42	0.22	4.744	82.51	0.22
4.748	113.79	0.23	4.750	104.88	0.23	4.759	91.89	0.23	4.764	82.92	0.22
4.768	114.49	0.23	4.769	105.50	0.23	4.779	92.40	0.23	4.784	83.33	0.22
4.788	115.21	0.23	4.789	106.16	0.23	4.799	92.90	0.23	4.804	83.74	0.22
4.807	115.90	0.23	4.809	106.79	0.23	4.818	93.39	0.23	4.824	84.14	0.22
4.827	116.63	0.23	4.829	107.40	0.23	4.838	93.85	0.23	4.844	84.56	0.22
4.847	117.39	0.23	4.849	108.00	0.23	4.858	94.31	0.23	4.864	84.98	0.22
4.867	118.14	0.23	4.869	108.59	0.23	4.878	94.79	0.23	4.884	85.40	0.22
4.887	118.85	0.23	4.889	109.23	0.23	4.898	95.29	0.23	4.904	85.82	0.22
4.907	119.60	0.23	4.909	109.89	0.23	4.918	95.78	0.23	4.924	86.25	0.22
4.927	120.34	0.23	4.929	110.50	0.23	4.938	96.29	0.23	4.943	86.65	0.22
4.946	121.04	0.23	4.949	111.15	0.23	4.958	96.80	0.23	4.963	87.07	0.22
4.966	121.81	0.23	4.969	111.80	0.23	4.978	97.27	0.23	4.983	87.48	0.22
4.986	122.59	0.23	4.988	112.43	0.23	4.998	97.74	0.23	5.003	87.90	0.22
5.006	123.36	0.23	5.008	113.01	0.23	5.018	98.25	0.23	5.023	88.31	0.22
5.026	124.12	0.23	5.028	113.67	0.23	5.038	98.74	0.23	5.043	88.72	0.22
5.046	124.87	0.23	5.048	114.32	0.23	5.058	99.24	0.23	5.063	89.15	0.22
5.066	125.62	0.23	5.068	114.97	0.23	5.078	99.74	0.23	5.083	89.60	0.22
5.085	126.40	0.23	5.088	115.66	0.23	5.098	100.26	0.23	5.103	90.00	0.22
5.105	127.22	0.23	5.108	116.31	0.23	5.118	100.77	0.23	5.123	90.41	0.22
5.125	128.01	0.23	5.128	116.95	0.23	5.137	101.27	0.23	5.143	90.82	0.22
5.145	128.81	0.23	5.148	117.59	0.23	5.157	101.76	0.23	5.163	91.26	0.22
5.165	129.59	0.23	5.168	118.28	0.23	5.177	102.26	0.23	5.183	91.67	0.22
5.185	130.39	0.23	5.188	118.96	0.23	5.197	102.78	0.23	5.203	92.08	0.22
5.205	131.19	0.23	5.207	119.63	0.23	5.217	103.28	0.23	5.223	92.50	0.22
5.224	132.02	0.23	5.227	120.27	0.23	5.237	103.77	0.23	5.242	92.92	0.22
5.244	132.83	0.23	5.247	120.95	0.23	5.257	104.27	0.23	5.262	93.34	0.22
5.264	133.67	0.23	5.267	121.64	0.23	5.277	104.79	0.23	5.282	93.76	0.22

5.284	134.49	0.23	5.287	122.32	0.23	5.297	105.33	0.23	5.302	94.19	0.22
5.304	135.33	0.23	5.307	123.01	0.23	5.317	105.85	0.23	5.322	94.67	0.22
5.324	136.15	0.23	5.327	123.73	0.23	5.337	106.36	0.23	5.342	95.10	0.22
5.343	137.00	0.23	5.347	124.42	0.23	5.357	106.88	0.23	5.362	95.51	0.22
5.363	137.84	0.23	5.367	125.10	0.23	5.377	107.39	0.23	5.382	95.93	0.22
5.383	138.71	0.23	5.387	125.77	0.23	5.397	107.90	0.23	5.402	96.40	0.22
5.403	139.61	0.23	5.407	126.49	0.23	5.417	108.43	0.23	5.422	96.82	0.22
5.423	140.48	0.23	5.426	127.22	0.23	5.437	108.94	0.23	5.442	97.24	0.22
5.443	141.37	0.23	5.446	127.93	0.23	5.456	109.45	0.23	5.462	97.69	0.22
5.463	142.26	0.23	5.466	128.62	0.23	5.476	109.98	0.23	5.482	98.09	0.22
5.482	143.11	0.23	5.486	129.31	0.23	5.496	110.51	0.23	5.502	98.50	0.22
5.502	144.01	0.23	5.506	130.04	0.23	5.516	111.05	0.23	5.522	98.96	0.22
5.522	144.93	0.23	5.526	130.76	0.23	5.536	111.57	0.23	5.541	99.44	0.23
5.542	145.85	0.23	5.546	131.48	0.23	5.556	112.07	0.23	5.561	99.85	0.23
5.562	146.74	0.23	5.566	132.21	0.23	5.576	112.59	0.23	5.581	100.27	0.23
5.582	147.67	0.23	5.586	132.92	0.23	5.596	113.16	0.23	5.601	100.72	0.23
5.602	148.58	0.23	5.606	133.68	0.23	5.616	113.72	0.23	5.621	101.17	0.23
5.621	149.55	0.23	5.626	134.46	0.23	5.636	114.24	0.23	5.641	101.59	0.23
5.641	150.47	0.23	5.645	135.19	0.23	5.656	114.77	0.23	5.661	102.05	0.23
5.661	151.46	0.23	5.665	135.92	0.23	5.676	115.29	0.23	5.681	102.49	0.23
5.681	152.43	0.23	5.685	136.68	0.23	5.696	115.82	0.23	5.701	102.91	0.23
5.701	153.41	0.23	5.705	137.46	0.23	5.716	116.36	0.23	5.721	103.37	0.23
5.721	154.36	0.23	5.725	138.19	0.23	5.736	116.90	0.23	5.741	103.82	0.23
5.741	155.34	0.23	5.745	138.93	0.23	5.756	117.46	0.23	5.761	104.26	0.23
5.760	156.32	0.23	5.765	139.69	0.23	5.775	118.02	0.23	5.781	104.67	0.23
5.780	157.32	0.23	5.785	140.49	0.23	5.795	118.54	0.23	5.801	105.12	0.23
5.800	158.34	0.23	5.805	141.25	0.23	5.815	119.07	0.23	5.821	105.59	0.23
5.820	159.37	0.23	5.825	142.03	0.23	5.835	119.64	0.23	5.840	106.06	0.23
5.840	160.38	0.23	5.845	142.82	0.23	5.855	120.19	0.23	5.860	106.46	0.23
5.860	161.43	0.23	5.864	143.59	0.23	5.875	120.75	0.23	5.880	106.94	0.23
5.880	162.53	0.23	5.884	144.35	0.23	5.895	121.30	0.23	5.900	107.43	0.23
5.899	163.56	0.23	5.904	145.12	0.23	5.915	121.86	0.23	5.920	107.88	0.23
5.919	164.62	0.23	5.924	145.87	0.23	5.935	122.42	0.23	5.940	108.31	0.23
5.939	165.71	0.23	5.944	146.66	0.23	5.955	122.97	0.23	5.960	108.76	0.23
5.959	166.81	0.23	5.964	147.47	0.23	5.975	123.54	0.23	5.980	109.21	0.23
5.979	167.90	0.23	5.984	148.28	0.23	5.995	124.10	0.23	6.000	109.65	0.23
5.999	168.97	0.23	6.004	149.10	0.23	6.015	124.65	0.23	6.020	110.12	0.23
6.018	170.11	0.23	6.024	149.92	0.23	6.035	125.21	0.23	6.040	110.56	0.23
6.038	171.26	0.23	6.044	150.73	0.23	6.055	125.78	0.23	6.060	110.99	0.23
6.058	172.40	0.23	6.064	151.57	0.23	6.075	126.33	0.23	6.080	111.46	0.23
6.078	173.53	0.23	6.083	152.38	0.23	6.094	126.91	0.23	6.100	111.88	0.23
6.098	174.71	0.23	6.103	153.17	0.23	6.114	127.50	0.23	6.120	112.34	0.23
6.118	175.91	0.23	6.123	154.01	0.23	6.134	128.07	0.23	6.139	112.82	0.23
6.138	177.10	0.23	6.143	154.83	0.23	6.154	128.63	0.23	6.159	113.30	0.23
6.157	178.28	0.23	6.163	155.71	0.23	6.174	129.19	0.23	6.179	113.72	0.23

6.177	179.51	0.23	6.183	156.51	0.23	6.194	129.77	0.23	6.199	114.20	0.23
6.197	180.72	0.23	6.203	157.38	0.23	6.214	130.36	0.23	6.219	114.69	0.23
6.217	182.00	0.23	6.223	158.29	0.23	6.234	130.94	0.23	6.239	115.16	0.23
6.237	183.27	0.23	6.243	159.10	0.23	6.254	131.54	0.23	6.259	115.59	0.23
6.257	184.50	0.23	6.263	160.01	0.23	6.274	132.13	0.23	6.279	116.08	0.23
6.277	185.76	0.23	6.283	160.90	0.23	6.294	132.70	0.23	6.299	116.56	0.23
6.296	187.04	0.23	6.302	161.82	0.23	6.314	133.27	0.23	6.319	117.01	0.23
6.316	188.38	0.23	6.322	162.71	0.23	6.334	133.85	0.23	6.339	117.43	0.23
6.336	189.80	0.23	6.342	163.63	0.23	6.354	134.45	0.23	6.359	117.92	0.23
6.356	191.15	0.23	6.362	164.53	0.23	6.374	135.03	0.23	6.379	118.39	0.23
6.376	192.49	0.23	6.382	165.42	0.23	6.394	135.62	0.23	6.399	118.86	0.23
6.396	193.89	0.23	6.402	166.34	0.23	6.413	136.21	0.23	6.419	119.33	0.23
6.416	195.28	0.23	6.422	167.29	0.23	6.433	136.81	0.23	6.438	119.81	0.23
6.435	196.74	0.23	6.442	168.23	0.23	6.453	137.42	0.23	6.458	120.29	0.23
6.455	198.24	0.23	6.462	169.19	0.23	6.473	138.04	0.23	6.478	120.75	0.23
6.475	199.77	0.23	6.482	170.06	0.23	6.493	138.65	0.23	6.498	121.21	0.23
6.495	201.21	0.23	6.502	171.01	0.23	6.513	139.21	0.23	6.518	121.68	0.23
6.515	202.72	0.23	6.521	171.98	0.23	6.533	139.81	0.23	6.538	122.17	0.23
6.535	204.27	0.23	6.541	172.93	0.23	6.553	140.43	0.23	6.558	122.66	0.23
6.555	205.85	0.23	6.561	173.96	0.23	6.573	141.03	0.23	6.578	123.12	0.23
6.574	207.43	0.23	6.581	174.82	0.23	6.593	141.66	0.23	6.598	123.63	0.23
6.594	209.06	0.23	6.601	175.88	0.23	6.613	142.29	0.23	6.618	124.11	0.23
6.614	210.76	0.23	6.621	176.91	0.23	6.633	142.91	0.23	6.638	124.57	0.23
6.634	212.41	0.23	6.641	177.90	0.23	6.653	143.53	0.23	6.658	125.08	0.23
6.654	214.11	0.23	6.661	178.91	0.23	6.673	144.11	0.23	6.678	125.55	0.23
6.674	215.86	0.23	6.681	179.88	0.23	6.693	144.70	0.23	6.698	126.02	0.23
6.693	217.70	0.23	6.701	180.89	0.23	6.713	145.33	0.23	6.718	126.53	0.23
8.014	698.65	0.31	6.721	182.00	0.23	6.732	145.99	0.23	6.737	127.02	0.23
8.032	700.01	0.31	6.740	182.96	0.23	6.752	146.64	0.23	6.757	127.52	0.23
8.050	701.37	0.31	6.760	184.14	0.23	6.772	147.24	0.23	6.777	128.01	0.23
8.068	702.73	0.31	6.780	185.19	0.23	6.792	147.84	0.23	6.797	128.45	0.23
8.086	704.09	0.31	6.800	186.32	0.23	6.812	148.48	0.23	6.817	128.92	0.23
8.104	705.21	0.31	6.820	187.38	0.23	6.832	149.12	0.23	6.837	129.41	0.23
8.122	706.14	0.31	6.840	188.48	0.23	6.852	149.74	0.23	6.857	129.91	0.23
8.140	707.07	0.31	6.860	189.54	0.23	6.872	150.37	0.23	6.877	130.44	0.23
8.158	708.01	0.31	6.880	190.67	0.23	6.892	151.00	0.23	6.897	130.95	0.23
8.176	708.94	0.31	6.900	191.82	0.23	6.912	151.64	0.23	6.917	131.43	0.23
8.194	709.87	0.31	6.920	193.00	0.23	6.932	152.30	0.23	6.937	131.91	0.23
8.212	710.81	0.31	6.940	194.16	0.23	6.952	152.95	0.23	6.957	132.40	0.23
8.230	711.75	0.31	6.959	195.37	0.23	6.972	153.60	0.23	6.977	132.88	0.23
8.248	712.69	0.31	6.979	196.62	0.23	6.992	154.25	0.23	6.997	133.38	0.23
8.266	713.64	0.32	6.999	197.88	0.23	7.012	154.88	0.23	7.017	133.88	0.23
8.284	714.58	0.32	7.019	198.97	0.23	7.032	155.54	0.23	7.036	134.37	0.23
8.302	715.43	0.32	7.039	200.07	0.23	7.051	156.20	0.23	7.056	134.89	0.23
8.320	716.20	0.32	7.059	201.16	0.23	7.071	156.86	0.23	7.076	135.37	0.23

8.338	716.96	0.32	7.079	202.26	0.23	7.091	157.54	0.23	7.096	135.85	0.23
8.356	717.73	0.32	7.099	203.37	0.23	7.111	158.19	0.23	7.116	136.35	0.23
8.374	718.49	0.32	7.119	204.97	0.23	7.131	158.84	0.23	7.136	136.84	0.23
8.392	719.26	0.32	7.139	206.60	0.23	7.151	159.51	0.23	7.156	137.34	0.23
8.410	720.05	0.32	7.159	207.87	0.23	7.171	160.20	0.23	7.176	137.86	0.23
8.428	720.84	0.32	7.178	209.14	0.23	7.191	160.88	0.23	7.196	138.38	0.23
8.446	721.64	0.32	7.198	210.43	0.23	7.211	161.56	0.23	7.216	138.87	0.23
8.464	722.43	0.32	7.218	211.72	0.23	7.231	162.23	0.23	7.236	139.37	0.23
8.482	723.23	0.32	7.238	213.03	0.23	7.251	162.89	0.23	7.256	139.90	0.23
8.500	723.98	0.32	7.258	214.36	0.23	7.271	163.60	0.23	7.276	140.41	0.23
8.518	724.67	0.32	7.278	215.71	0.24	7.291	164.29	0.23	7.296	140.92	0.23
8.536	725.37	0.32	7.298	217.08	0.24	7.311	164.96	0.23	7.316	141.46	0.23
8.553	726.06	0.32	7.318	218.44	0.24	7.331	165.67	0.23	7.335	141.94	0.23
8.571	726.76	0.32	7.338	219.83	0.24	7.351	166.38	0.23	7.355	142.46	0.23
8.589	727.45	0.32	7.358	221.27	0.24	7.370	167.09	0.23	7.375	142.99	0.23
8.607	728.14	0.32	7.378	222.67	0.24	7.390	167.81	0.23	7.395	143.52	0.23
8.625	728.82	0.32	7.397	224.05	0.24	7.410	168.54	0.23	7.415	144.00	0.23
8.643	729.51	0.32	7.417	225.26	0.24	7.430	169.25	0.23	7.435	144.51	0.23
8.661	730.19	0.32	7.437	226.59	0.24	7.450	169.96	0.23	7.455	145.04	0.23
8.679	730.88	0.32	7.457	228.11	0.24	7.470	170.65	0.23	7.475	145.56	0.23
8.697	731.58	0.32	7.477	229.43	0.24	7.490	171.35	0.23	7.495	146.08	0.23
8.715	732.28	0.32	7.497	230.75	0.24	7.510	172.04	0.23	7.515	146.64	0.23
8.733	732.99	0.32	7.517	232.28	0.24	7.530	173.09	0.23	7.535	147.20	0.23
8.751	733.70	0.32	7.537	233.86	0.24	7.550	173.80	0.23	7.555	147.72	0.23
8.769	734.41	0.32	7.557	235.44	0.24	7.570	174.50	0.23	7.575	148.24	0.23
8.787	735.12	0.32	7.577	237.03	0.24	7.590	175.22	0.23	7.595	148.77	0.23
8.805	735.72	0.32	7.597	238.62	0.24	7.610	175.94	0.23	7.615	149.28	0.23
8.823	736.30	0.32	7.616	240.25	0.24	7.630	176.67	0.23	7.634	149.80	0.23
8.841	736.87	0.32	7.636	241.88	0.24	7.650	177.38	0.23	7.654	150.30	0.23
8.859	737.44	0.32	7.656	243.54	0.24	7.670	178.09	0.23	7.674	150.83	0.23
8.877	738.01	0.32	7.676	245.25	0.24	7.690	178.81	0.23	7.694	151.38	0.23
8.895	738.58	0.32	7.696	246.96	0.24	7.709	179.54	0.23	7.714	151.90	0.23
8.913	739.16	0.32	7.716	248.74	0.24	7.729	180.27	0.23	7.734	152.44	0.23
8.931	739.73	0.32	7.736	250.53	0.24	7.749	181.02	0.23	7.754	152.99	0.23
8.949	740.30	0.32	7.756	252.32	0.24	7.769	181.77	0.23	7.774	153.50	0.23
8.967	740.87	0.32	7.776	254.11	0.24	7.789	182.51	0.23	7.794	154.04	0.23
8.985	741.44	0.32	7.796	255.91	0.24	7.809	183.24	0.23	7.814	154.59	0.23
9.003	742.01	0.32	7.816	257.85	0.24	7.829	183.99	0.23	7.834	155.13	0.23
9.021	742.58	0.32	7.835	259.84	0.24	7.849	184.76	0.23	7.854	155.68	0.23
9.039	743.14	0.32	7.855	261.86	0.24	7.869	185.54	0.23	7.874	156.23	0.23
9.057	743.70	0.32	7.875	263.93	0.24	7.889	186.30	0.23	7.894	156.77	0.23
9.075	744.26	0.32	7.895	266.00	0.24	7.909	187.05	0.23	7.914	157.31	0.23
9.093	744.82	0.32	7.915	268.10	0.24	7.929	187.83	0.23	7.933	157.86	0.23
9.111	745.38	0.32	7.935	270.21	0.24	7.949	188.62	0.23	7.953	158.41	0.23
9.129	745.95	0.32	7.955	272.33	0.24	7.969	189.41	0.23	7.973	158.95	0.23

9.146	746.52	0.32	7.975	274.48	0.24	7.989	190.17	0.23	7.993	159.48	0.23
9.164	747.09	0.32	7.995	276.63	0.24	8.009	190.96	0.23	8.013	160.01	0.23
9.182	747.66	0.32	8.015	278.90	0.24	8.028	191.77	0.23	8.033	160.56	0.23
9.200	748.23	0.32	8.035	281.21	0.24	8.048	192.55	0.23	8.053	161.08	0.23
9.218	748.79	0.32	8.054	283.53	0.24	8.068	193.34	0.23	8.073	161.64	0.23
9.236	749.33	0.32	8.074	285.92	0.24	8.088	194.15	0.23	8.093	162.21	0.23
9.254	749.88	0.32	8.094	288.31	0.24	8.108	194.92	0.23	8.113	162.78	0.23
9.272	750.42	0.32	8.114	290.78	0.24	8.128	195.71	0.23	8.133	163.34	0.23
9.290	750.97	0.32	8.134	293.29	0.24	8.148	196.51	0.23	8.153	163.85	0.23
9.308	751.51	0.32	8.154	295.86	0.24	8.168	197.30	0.23	8.173	164.40	0.23
9.326	752.01	0.32	8.174	298.65	0.24	8.188	198.11	0.23	8.193	164.97	0.23
9.344	752.50	0.32	8.194	301.43	0.24	8.208	198.95	0.23	8.213	165.54	0.23
9.362	753.00	0.32	8.214	304.22	0.24	8.228	199.77	0.23	8.232	166.07	0.23
9.380	753.49	0.32	8.234	307.01	0.24	8.248	200.59	0.23	8.252	166.64	0.23
9.398	753.99	0.32	8.254	309.84	0.24	8.268	201.40	0.23	8.272	167.19	0.23
9.416	754.49	0.32	8.273	312.91	0.25	8.288	202.23	0.23	8.292	167.76	0.23
9.434	755.00	0.32	8.293	315.98	0.25	8.308	203.07	0.23	8.312	168.36	0.23
9.452	755.51	0.32	8.313	319.12	0.25	8.328	203.91	0.23	8.332	168.92	0.23
9.470	756.02	0.32	8.333	322.31	0.25	8.347	204.72	0.23	8.352	169.48	0.23
9.488	756.53	0.32	8.353	325.53	0.25	8.367	205.52	0.23	8.372	170.06	0.23
9.506	757.04	0.32	8.373	328.95	0.25	8.387	206.31	0.23	8.392	170.64	0.23
9.524	757.52	0.32	8.393	332.38	0.25	8.407	207.15	0.23	8.412	171.18	0.23
9.542	757.99	0.32	8.413	336.01	0.25	8.427	208.01	0.23	8.432	171.78	0.23
9.560	758.47	0.32	8.433	339.75	0.25	8.447	208.88	0.23	8.452	172.37	0.23
9.578	758.95	0.32	8.453	343.54	0.25	8.467	209.75	0.23	8.472	172.95	0.23
9.596	759.42	0.32	8.473	347.67	0.25	8.487	210.60	0.23	8.492	173.50	0.23
9.614	759.89	0.33	8.492	351.79	0.25	8.507	211.48	0.23	8.512	174.07	0.23
9.632	760.34	0.33	8.512	355.92	0.25	8.527	212.37	0.23	8.531	174.67	0.23
9.650	760.80	0.33	8.532	360.04	0.25	8.547	213.26	0.23	8.551	175.26	0.23
9.668	761.25	0.33	8.552	364.44	0.25	8.567	214.18	0.23	8.571	175.85	0.23
9.686	761.70	0.33	8.572	368.71	0.25	8.587	215.11	0.23	8.591	176.44	0.23
9.704	762.15	0.33	8.592	373.26	0.25	8.607	215.98	0.23	8.611	177.03	0.23
9.721	762.59	0.33	8.612	377.69	0.25	8.627	216.86	0.23	8.631	177.61	0.23
9.739	763.03	0.33	8.632	382.25	0.25	8.647	217.76	0.23	8.651	178.17	0.23
9.757	763.47	0.33	8.652	387.77	0.26	8.666	218.64	0.23	8.671	178.73	0.23
9.775	763.91	0.33	8.672	393.30	0.26	8.686	219.51	0.23	8.691	179.31	0.23
9.793	764.35	0.33	8.692	398.82	0.26	8.706	220.41	0.23	8.711	179.91	0.23
9.811	764.79	0.33	8.711	404.34	0.26	8.726	221.34	0.23	8.731	180.54	0.23
9.829	765.22	0.33	8.731	409.87	0.26	8.746	222.26	0.23	8.751	181.12	0.23
9.847	765.66	0.33	8.751	415.39	0.26	8.766	223.20	0.23	8.771	181.71	0.23
9.865	766.09	0.33	8.771	420.91	0.26	8.786	224.16	0.23	8.791	182.32	0.23
9.883	766.53	0.33	8.791	426.43	0.26	8.806	225.14	0.23	8.811	182.88	0.23
9.901	766.96	0.33	8.811	431.96	0.26	8.826	226.07	0.23	8.830	183.47	0.23
9.919	767.36	0.33	8.831	437.48	0.26	8.846	227.05	0.23	8.850	184.09	0.23
9.937	767.75	0.33	8.851	443.00	0.26	8.866	228.01	0.23	8.870	184.69	0.23

9.955	768.14	0.33	8.871	448.41	0.26	8.886	228.95	0.24	8.890	185.31	0.23
9.973	768.53	0.33	8.891	453.26	0.27	8.906	229.93	0.24	8.910	185.92	0.23
9.991	768.92	0.33	8.911	458.80	0.27	8.926	230.92	0.24	8.930	186.52	0.23
10.009	769.30	0.33	8.930	465.04	0.27	8.946	231.89	0.24	8.950	187.12	0.23
10.027	769.69	0.33	8.950	470.00	0.27	8.966	232.89	0.24	8.970	187.72	0.23
10.045	770.07	0.33	8.970	475.32	0.27	8.985	233.90	0.24	8.990	188.32	0.23
10.063	770.45	0.33	8.990	479.67	0.27	9.005	234.88	0.24	9.010	188.92	0.23
10.081	770.84	0.33	9.010	484.03	0.27	9.025	235.64	0.24	9.030	189.55	0.23
10.099	771.22	0.33	9.030	488.39	0.27	9.045	236.64	0.24	9.050	190.19	0.23
10.117	771.62	0.33	9.050	492.75	0.27	9.065	237.65	0.24	9.070	190.78	0.23
10.135	772.04	0.33	9.070	497.10	0.27	9.085	238.69	0.24	9.090	191.39	0.23
10.153	772.46	0.33	9.090	501.46	0.27	9.105	239.72	0.24	9.110	192.01	0.23
10.171	772.88	0.33	9.110	505.82	0.27	9.125	240.72	0.24	9.130	192.60	0.23
10.189	773.29	0.33	9.130	510.18	0.27	9.145	241.72	0.24	9.149	193.21	0.23
10.207	773.71	0.33	9.149	514.53	0.27	9.165	242.75	0.24	9.169	193.84	0.23
10.225	774.11	0.33	9.169	518.89	0.28	9.185	243.82	0.24	9.189	194.48	0.23
10.243	774.51	0.33	9.189	523.25	0.28	9.205	244.87	0.24	9.209	195.10	0.23
10.261	774.91	0.33	9.209	527.61	0.28	9.225	245.85	0.24	9.229	195.72	0.23
10.279	775.31	0.33	9.229	531.96	0.28	9.245	246.83	0.24	9.249	196.35	0.23
10.297	775.71	0.33	9.249	536.32	0.28	9.265	247.81	0.24	9.269	196.97	0.23
10.314	776.10	0.33	9.269	540.68	0.28	9.285	248.87	0.24	9.289	197.58	0.23
10.332	776.48	0.33	9.289	545.04	0.28	9.304	249.94	0.24	9.309	198.21	0.23
10.350	776.86	0.33	9.309	549.40	0.28	9.324	251.01	0.24	9.329	198.85	0.23
10.368	777.24	0.33	9.329	553.08	0.28	9.344	252.04	0.24	9.349	199.50	0.23
10.386	777.62	0.33	9.349	555.93	0.28	9.364	253.07	0.24	9.369	200.12	0.23
10.404	778.00	0.33	9.368	558.54	0.28	9.384	254.10	0.24	9.389	200.75	0.23
10.422	778.39	0.33	9.388	561.29	0.28	9.404	255.04	0.24	9.409	201.39	0.23
10.440	778.79	0.33	9.408	564.18	0.28	9.424	255.99	0.24	9.429	202.05	0.23
10.458	779.19	0.33	9.428	567.48	0.29	9.444	256.94	0.24	9.448	202.70	0.23
10.476	779.59	0.33	9.448	570.69	0.29	9.464	258.16	0.24	9.468	203.34	0.23
10.494	779.98	0.33	9.468	573.50	0.29	9.484	259.37	0.24	9.488	203.99	0.23
10.512	780.35	0.33	9.488	576.41	0.29	9.504	260.58	0.24	9.508	204.64	0.23
10.530	780.71	0.33	9.508	579.00	0.29	9.524	261.75	0.24	9.528	205.28	0.23
10.548	781.08	0.33	9.528	581.48	0.29	9.544	262.91	0.24	9.548	205.88	0.23
10.566	781.44	0.33	9.548	584.02	0.29	9.564	264.07	0.24	9.568	206.47	0.23
10.584	781.79	0.33	9.568	586.22	0.29	9.584	265.08	0.24	9.588	207.11	0.23
10.602	782.14	0.33	9.587	588.94	0.29	9.604	266.08	0.24	9.608	207.75	0.23
10.620	782.50	0.33	9.607	591.13	0.29	9.623	267.09	0.24	9.628	208.38	0.23
10.638	782.87	0.33	9.627	593.21	0.29	9.643	268.23	0.24	9.648	209.00	0.23
10.656	783.23	0.33	9.647	595.32	0.29	9.663	269.37	0.24	9.668	209.64	0.23
10.674	783.58	0.33	9.667	597.52	0.29	9.683	270.51	0.24	9.688	210.34	0.23
10.692	783.92	0.33	9.687	599.57	0.29	9.703	271.70	0.24	9.708	210.98	0.23
10.710	784.23	0.33	9.707	601.57	0.29	9.723	272.88	0.24	9.728	211.64	0.23
10.728	784.58	0.33	9.727	603.55	0.29	9.743	274.07	0.24	9.747	212.32	0.23
10.746	784.95	0.33	9.747	605.58	0.29	9.763	275.21	0.24	9.767	212.99	0.23

10.764	785.30	0.33	9.767	607.46	0.29	9.783	276.35	0.24	9.787	213.66	0.23
10.782	785.63	0.33	9.787	609.28	0.29	9.803	277.49	0.24	9.807	214.44	0.23
10.800	785.93	0.33	9.806	611.17	0.29	9.823	278.67	0.24	9.827	215.17	0.23
10.818	786.24	0.33	9.826	612.88	0.29	9.843	279.84	0.24	9.847	215.78	0.23
10.836	786.56	0.33	9.846	614.53	0.29	9.863	281.02	0.24	9.867	216.28	0.23
10.854	786.86	0.33	9.866	616.15	0.29	9.883	282.11	0.24	9.887	216.99	0.23
10.872	787.16	0.33	9.886	617.72	0.30	9.903	283.21	0.24	9.907	217.75	0.23
10.890	787.49	0.33	9.906	619.24	0.30	9.923	284.31	0.24	9.927	218.48	0.23
10.907	787.83	0.33	9.926	620.77	0.30	9.942	285.51	0.24	9.947	219.13	0.23
10.925	788.18	0.33	9.946	622.27	0.30	9.962	286.71	0.24	9.967	219.83	0.23
10.943	788.53	0.33	9.966	623.84	0.30	9.982	287.91	0.24	9.987	220.54	0.23
10.961	788.86	0.33	9.986	625.40	0.30	10.002	289.11	0.24	10.007	221.22	0.23
10.979	789.19	0.33	10.006	626.96	0.30	10.022	290.30	0.24	10.027	221.88	0.23
10.997	789.53	0.33	10.025	628.53	0.30	10.042	291.50	0.24	10.046	222.56	0.23
11.015	789.84	0.33	10.045	629.91	0.30	10.062	292.77	0.24	10.066	223.23	0.23
11.033	790.17	0.33	10.065	631.42	0.30	10.082	294.04	0.24	10.086	223.91	0.23
11.051	790.52	0.33	10.085	633.02	0.30	10.102	295.31	0.24	10.106	224.60	0.23
11.069	790.86	0.33	10.105	634.38	0.30	10.122	296.55	0.24	10.126	225.32	0.23
11.087	791.17	0.33	10.125	635.81	0.30	10.142	297.79	0.24	10.146	226.03	0.23
11.105	791.47	0.33	10.145	637.16	0.30	10.162	299.02	0.24	10.166	226.72	0.23
11.123	791.76	0.33	10.165	638.38	0.30	10.182	300.16	0.24	10.186	227.42	0.23
11.141	792.07	0.33	10.185	639.69	0.30	10.202	301.30	0.24	10.206	228.11	0.23
11.159	792.39	0.33	10.205	640.90	0.30	10.222	302.54	0.24	10.226	228.78	0.23
11.177	792.70	0.33	10.225	642.20	0.30	10.242	303.77	0.24	10.246	229.48	0.23
11.195	793.00	0.33	10.244	643.32	0.30	10.261	305.01	0.24	10.266	230.08	0.23
11.213	793.30	0.33	10.264	644.58	0.30	10.281	306.41	0.24	10.286	230.64	0.23
11.231	793.63	0.33	10.284	645.83	0.30	10.301	307.81	0.24	10.306	231.32	0.23
11.249	793.97	0.33	10.304	647.02	0.30	10.321	309.13	0.24	10.326	232.05	0.24
11.267	794.28	0.33	10.324	648.07	0.30	10.341	310.44	0.24	10.345	232.71	0.24
11.285	794.60	0.33	10.344	649.22	0.30	10.361	311.76	0.24	10.365	233.40	0.24
11.303	794.95	0.33	10.364	650.34	0.30	10.381	313.03	0.24	10.385	234.14	0.24
11.321	795.26	0.33	10.384	651.48	0.30	10.401	314.31	0.24	10.405	234.86	0.24
11.339	795.56	0.33	10.404	652.65	0.30	10.421	315.66	0.24	10.425	235.59	0.24
11.357	795.84	0.33	10.424	653.84	0.30	10.441	317.01	0.25	10.445	236.32	0.24
11.375	796.15	0.33	10.444	654.98	0.30	10.461	318.34	0.25	10.465	237.04	0.24
11.393	796.46	0.33	10.463	656.06	0.30	10.481	319.68	0.25	10.485	237.74	0.24
11.411	796.77	0.33	10.483	657.13	0.30	10.501	321.01	0.25	10.505	238.41	0.24
11.429	797.08	0.33	10.503	658.21	0.30	10.521	322.34	0.25	10.525	239.13	0.24
11.447	797.38	0.33	10.523	659.32	0.30	10.541	323.68	0.25	10.545	239.89	0.24
11.465	797.68	0.33	10.543	660.47	0.30	10.561	325.01	0.25	10.565	240.62	0.24
11.483	797.97	0.33	10.563	661.58	0.30	10.580	326.26	0.25	10.585	241.37	0.24
11.500	798.27	0.33	10.583	662.68	0.30	10.600	327.51	0.25	10.605	242.18	0.24
11.518	798.58	0.33	10.603	663.76	0.30	10.620	328.91	0.25	10.625	242.98	0.24
11.536	798.89	0.33	10.623	664.80	0.30	10.640	330.31	0.25	10.644	243.68	0.24
11.554	799.17	0.33	10.643	665.82	0.30	10.660	331.61	0.25	10.664	244.29	0.24

11.572	799.46	0.33	10.663	666.84	0.31	10.680	332.91	0.25	10.684	244.93	0.24
11.590	799.76	0.33	10.682	667.86	0.31	10.700	334.21	0.25	10.704	245.63	0.24
11.608	800.03	0.33	10.702	668.85	0.31	10.720	335.59	0.25	10.724	246.38	0.24
11.626	800.29	0.33	10.722	669.83	0.31	10.740	337.00	0.25	10.744	247.11	0.24
11.644	800.56	0.33	10.742	670.80	0.31	10.760	338.42	0.25	10.764	247.86	0.24
11.662	800.85	0.33	10.762	671.75	0.31	10.780	339.85	0.25	10.784	248.61	0.24
11.680	801.13	0.33	10.782	672.66	0.31	10.800	341.28	0.25	10.804	249.35	0.24
11.698	801.41	0.33	10.802	673.56	0.31	10.820	342.70	0.25	10.824	250.06	0.24
11.716	801.68	0.33	10.822	674.47	0.31	10.840	344.15	0.25	10.844	250.78	0.24
11.734	801.96	0.33	10.842	675.36	0.31	10.860	345.58	0.25	10.864	251.52	0.24
11.752	802.21	0.33	10.862	676.24	0.31	10.880	347.00	0.25	10.884	252.27	0.24
11.770	802.44	0.33	10.882	677.11	0.31	10.900	348.46	0.25	10.904	253.03	0.24
11.788	802.71	0.33	10.901	677.95	0.31	10.919	349.88	0.25	10.924	253.83	0.24
11.806	802.99	0.33	10.921	678.78	0.31	10.939	351.29	0.25	10.943	254.57	0.24
11.824	803.26	0.33	10.941	679.58	0.31	10.959	352.69	0.25	10.963	255.32	0.24
11.842	803.53	0.33	10.961	680.38	0.31	10.979	353.95	0.25	10.983	256.08	0.24
11.860	803.80	0.33	10.981	681.17	0.31	10.999	355.39	0.25	11.003	256.85	0.24
11.878	804.11	0.33	11.001	681.97	0.31	11.019	356.94	0.25	11.023	257.62	0.24
11.896	804.42	0.33	11.021	682.81	0.31	11.039	358.42	0.25	11.043	258.38	0.24
11.914	804.71	0.33	11.041	683.66	0.31	11.059	359.89	0.25	11.063	259.13	0.24
11.932	805.01	0.33	11.061	684.50	0.31	11.079	361.37	0.25	11.083	259.88	0.24
11.950	805.28	0.33	11.081	685.35	0.31	11.099	362.88	0.25	11.103	260.68	0.24
11.968	805.52	0.34	11.101	686.19	0.31	11.119	364.36	0.25	11.123	261.47	0.24
11.986	805.83	0.34	11.120	686.95	0.31	11.139	365.85	0.25	11.143	262.22	0.24
12.004	806.13	0.34	11.140	687.71	0.31	11.159	367.44	0.25	11.163	262.95	0.24
12.022	806.41	0.34	11.160	688.47	0.31	11.179	368.99	0.25	11.183	263.70	0.24
12.040	806.68	0.34	11.180	689.23	0.31	11.199	370.49	0.25	11.203	264.47	0.24
12.058	806.95	0.34	11.200	689.99	0.31	11.219	372.00	0.25	11.223	265.23	0.24
12.075	807.21	0.34	11.220	690.75	0.31	11.238	373.51	0.25	11.242	266.00	0.24
12.093	807.46	0.34	11.240	691.51	0.31	11.258	375.00	0.25	11.262	266.78	0.24
12.111	807.72	0.34	11.260	692.28	0.31	11.278	376.50	0.25	11.282	267.58	0.24
12.129	807.98	0.34	11.280	693.04	0.31	11.298	378.00	0.25	11.302	268.39	0.24
12.147	808.27	0.34	11.300	693.80	0.31	11.318	379.49	0.25	11.322	269.15	0.24
12.165	808.57	0.34	11.320	694.55	0.31	11.338	380.93	0.25	11.342	269.87	0.24
12.183	808.83	0.34	11.339	695.25	0.31	11.358	382.37	0.25	11.362	270.66	0.24
12.201	809.08	0.34	11.359	695.95	0.31	11.378	383.85	0.25	11.382	271.45	0.24
12.219	809.33	0.34	11.379	696.67	0.31	11.398	385.47	0.25	11.402	272.22	0.24
12.237	809.57	0.34	11.399	697.44	0.31	11.418	387.00	0.25	11.422	273.03	0.24
12.255	809.83	0.34	11.419	697.99	0.31	11.438	388.51	0.25	11.442	273.81	0.24
12.273	810.07	0.34	11.439	698.55	0.31	11.458	390.03	0.25	11.462	274.53	0.24
12.291	810.36	0.34	11.459	699.11	0.31	11.478	391.54	0.25	11.482	275.23	0.24
12.309	810.64	0.34	11.479	699.73	0.31	11.498	393.05	0.26	11.502	275.95	0.24
12.327	810.90	0.34	11.499	700.44	0.31	11.518	394.55	0.26	11.522	276.74	0.24
12.345	811.15	0.34	11.519	701.16	0.31	11.538	395.95	0.26	11.541	277.54	0.24
12.363	811.43	0.34	11.539	701.87	0.31	11.557	397.31	0.26	11.561	278.31	0.24

12.381	811.70	0.34	11.558	702.57	0.31	11.577	398.80	0.26	11.581	279.08	0.24
12.399	811.95	0.34	11.578	703.26	0.31	11.597	400.32	0.26	11.601	279.93	0.24
12.417	812.19	0.34	11.598	703.95	0.31	11.617	401.83	0.26	11.621	280.72	0.24
12.435	812.46	0.34	11.618	704.64	0.31	11.637	403.35	0.26	11.641	281.51	0.24
12.453	812.75	0.34	11.638	705.31	0.31	11.657	404.89	0.26	11.661	282.30	0.24
12.471	813.03	0.34	11.658	705.97	0.31	11.677	406.40	0.26	11.681	283.11	0.24
12.489	813.26	0.34	11.678	706.63	0.31	11.697	407.95	0.26	11.701	283.94	0.24
12.507	813.51	0.34	11.698	707.28	0.31	11.717	409.46	0.26	11.721	284.80	0.24
12.525	813.78	0.34	11.718	707.93	0.31	11.737	411.00	0.26	11.741	285.59	0.24
12.543	814.06	0.34	11.738	708.58	0.31	11.757	412.55	0.26	11.761	286.41	0.24
12.561	814.30	0.34	11.758	709.22	0.31	11.777	414.10	0.26	11.781	287.23	0.24
12.579	814.53	0.34	11.777	709.85	0.31	11.797	415.65	0.26	11.801	288.05	0.24
12.597	814.77	0.34	11.797	710.48	0.31	11.817	417.16	0.26	11.821	288.86	0.24
12.615	815.03	0.34	11.817	711.10	0.31	11.837	418.70	0.26	11.840	289.68	0.24
12.633	815.29	0.34	11.837	711.72	0.31	11.857	420.25	0.26	11.860	290.49	0.24
12.651	815.54	0.34	11.857	712.32	0.31	11.876	421.71	0.26	11.880	291.30	0.24
12.668	815.79	0.34	11.877	712.93	0.31	11.896	423.17	0.26	11.900	292.11	0.24
12.686	816.06	0.34	11.897	713.52	0.31	11.916	424.62	0.26	11.920	292.92	0.24
12.704	816.31	0.34	11.917	714.10	0.32	11.936	426.03	0.26	11.940	293.64	0.24
12.722	816.56	0.34	11.937	714.68	0.32	11.956	427.44	0.26	11.960	294.47	0.24
12.740	816.81	0.34	11.957	715.26	0.32	11.976	428.89	0.26	11.980	295.38	0.24
12.758	817.04	0.34	11.977	715.84	0.32	11.996	430.45	0.26	12.000	296.19	0.24
12.776	817.25	0.34	11.996	716.42	0.32	12.016	431.99	0.26	12.020	297.00	0.24
12.794	817.49	0.34	12.016	716.97	0.32	12.036	433.55	0.26	12.040	297.83	0.24
12.812	817.76	0.34	12.036	717.52	0.32	12.056	435.10	0.26	12.060	298.67	0.24
12.830	818.00	0.34	12.056	718.08	0.32	12.076	436.64	0.26	12.080	299.47	0.24
12.848	818.23	0.34	12.076	718.64	0.32	12.096	438.20	0.26	12.100	300.29	0.24
12.866	818.46	0.34	12.096	719.21	0.32	12.116	439.71	0.26	12.120	301.16	0.24
12.884	818.69	0.34	12.116	719.77	0.32	12.136	441.20	0.26	12.139	302.05	0.24
12.902	818.96	0.34	12.136	720.31	0.32	12.156	442.64	0.26	12.159	302.85	0.24
12.920	819.21	0.34	12.156	720.83	0.32	12.176	444.07	0.26	12.179	303.69	0.24
12.938	819.44	0.34	12.176	721.36	0.32	12.195	445.53	0.26	12.199	304.54	0.24
12.956	819.69	0.34	12.196	721.89	0.32	12.215	446.99	0.26	12.219	305.38	0.24
12.974	819.94	0.34	12.215	722.41	0.32	12.235	448.45	0.26	12.239	306.19	0.24
12.992	820.16	0.34	12.235	722.94	0.32	12.255	449.90	0.26	12.259	306.95	0.24
13.010	820.42	0.34	12.255	723.47	0.32	12.275	451.36	0.26	12.279	307.71	0.24
13.028	820.68	0.34	12.275	723.99	0.32	12.295	452.79	0.26	12.299	308.61	0.24
13.046	820.94	0.34	12.295	724.50	0.32	12.315	454.22	0.26	12.319	309.44	0.24
13.064	821.15	0.34	12.315	725.01	0.32	12.335	455.64	0.26	12.339	310.30	0.24
13.082	821.36	0.34	12.335	725.52	0.32	12.355	457.06	0.26	12.359	311.15	0.24
13.100	821.60	0.34	12.355	726.02	0.32	12.375	458.51	0.27	12.379	311.96	0.24
13.118	821.84	0.34	12.375	726.52	0.32	12.395	459.98	0.27	12.399	312.84	0.24
13.136	822.08	0.34	12.395	727.02	0.32	12.415	461.37	0.27	12.419	313.68	0.24
13.154	822.32	0.34	12.415	727.51	0.32	12.435	462.80	0.27	12.438	314.49	0.24
13.172	822.56	0.34	12.434	728.00	0.32	12.455	464.25	0.27	12.458	315.27	0.24

13.190	822.75	0.34	12.454	728.47	0.32	12.475	465.64	0.27	12.478	316.09	0.24
13.208	822.95	0.34	12.474	728.95	0.32	12.495	467.05	0.27	12.498	316.94	0.24
13.226	823.18	0.34	12.494	729.44	0.32	12.514	468.43	0.27	12.518	317.83	0.24
13.244	823.42	0.34	12.514	729.91	0.32	12.534	469.87	0.27	12.538	318.72	0.25
13.261	823.68	0.34	12.534	730.37	0.32	12.554	471.31	0.27	12.558	319.59	0.25
13.279	823.94	0.34	12.554	730.84	0.32	12.574	472.74	0.27	12.578	320.45	0.25
13.297	824.17	0.34	12.574	731.31	0.32	12.594	474.18	0.27	12.598	321.32	0.25
13.315	824.39	0.34	12.594	731.77	0.32	12.614	475.61	0.27	12.618	322.25	0.25
13.333	824.61	0.34	12.614	732.23	0.32	12.634	477.06	0.27	12.638	323.02	0.25
13.351	824.83	0.34	12.634	732.68	0.32	12.654	478.51	0.27	12.658	323.76	0.25
13.369	825.05	0.34	12.653	733.11	0.32	12.674	479.77	0.27	12.678	324.64	0.25
13.387	825.27	0.34	12.673	733.54	0.32	12.694	481.04	0.27	12.698	325.50	0.25
13.405	825.55	0.34	12.693	733.97	0.32	12.714	482.31	0.27	12.718	326.34	0.25
13.423	825.77	0.34	12.713	734.42	0.32	12.734	483.91	0.27	12.737	327.20	0.25
13.441	826.00	0.34	12.733	734.88	0.32	12.754	485.51	0.27	12.757	328.08	0.25
13.459	826.25	0.34	12.753	735.35	0.32	12.774	486.97	0.27	12.777	328.96	0.25
13.477	826.47	0.34	12.773	735.81	0.32	12.794	488.44	0.27	12.797	329.83	0.25
13.495	826.68	0.34	12.793	736.27	0.32	12.814	489.91	0.27	12.817	330.70	0.25
13.513	826.91	0.34	12.813	736.73	0.32	12.833	490.96	0.27	12.837	331.55	0.25
13.531	827.14	0.34	12.833	737.20	0.32	12.853	492.01	0.27	12.857	332.39	0.25
13.549	827.34	0.34	12.853	737.66	0.32	12.873	493.34	0.27	12.877	333.20	0.25
13.567	827.55	0.34	12.872	738.12	0.32	12.893	494.67	0.27	12.897	333.97	0.25
13.585	827.78	0.34	12.892	738.58	0.32	12.913	496.01	0.27	12.917	334.83	0.25
13.603	828.02	0.34	12.912	739.05	0.32	12.933	497.01	0.27	12.937	335.69	0.25
13.621	828.24	0.34	12.932	739.51	0.32	12.953	498.01	0.27	12.957	336.60	0.25
13.639	828.45	0.34	12.952	739.97	0.32	12.973	499.51	0.27	12.977	337.51	0.25
13.657	828.66	0.34	12.972	740.43	0.32	12.993	501.01	0.27	12.997	338.39	0.25
13.675	828.89	0.34	12.992	740.90	0.32	13.013	502.34	0.27	13.017	339.30	0.25
13.693	829.11	0.34	13.012	741.36	0.32	13.033	503.67	0.27	13.036	340.20	0.25
13.711	829.30	0.34	13.032	741.76	0.32	13.053	505.01	0.27	13.056	341.08	0.25
13.729	829.49	0.34	13.052	742.19	0.32	13.073	506.26	0.27	13.076	341.95	0.25
13.747	829.70	0.34	13.072	742.62	0.32	13.093	507.51	0.27	13.096	342.82	0.25
13.765	829.94	0.34	13.091	743.04	0.32	13.113	508.67	0.27	13.116	343.70	0.25
13.783	830.18	0.34	13.111	743.47	0.32	13.133	509.84	0.27	13.136	344.58	0.25
13.801	830.41	0.34	13.131	743.89	0.32	13.152	511.01	0.27	13.156	345.49	0.25
13.819	830.64	0.34	13.151	744.31	0.32	13.172	512.34	0.27	13.176	346.38	0.25
13.836	830.87	0.34	13.171	744.73	0.32	13.192	513.67	0.27	13.196	347.29	0.25
13.854	831.09	0.34	13.191	745.15	0.32	13.212	515.01	0.28	13.216	348.19	0.25
13.872	831.32	0.34	13.211	745.57	0.32	13.232	516.01	0.28	13.236	349.09	0.25
13.890	831.52	0.34	13.231	745.99	0.32	13.252	517.01	0.28	13.256	349.97	0.25
13.908	831.69	0.34	13.251	746.42	0.32	13.272	518.17	0.28	13.276	350.83	0.25
13.926	831.89	0.34	13.271	746.83	0.32	13.292	519.34	0.28	13.296	351.75	0.25
13.944	832.12	0.34	13.291	747.24	0.32	13.312	520.51	0.28	13.316	352.64	0.25
13.962	832.37	0.34	13.310	747.64	0.32	13.332	521.76	0.28	13.335	353.46	0.25
13.980	832.60	0.34	13.330	748.04	0.32	13.352	523.01	0.28	13.355	354.28	0.25

13.998	832.81	0.34	13.350	748.44	0.32	13.372	524.04	0.28	13.375	355.10	0.25
14.016	833.01	0.34	13.370	748.89	0.32	13.392	525.07	0.28	13.395	355.93	0.25
14.034	833.21	0.34	13.390	749.33	0.32	13.412	526.11	0.28	13.415	356.84	0.25
14.052	833.41	0.34	13.410	749.77	0.32	13.432	527.41	0.28	13.435	357.76	0.25
14.070	833.62	0.34	13.430	750.21	0.32	13.452	528.71	0.28	13.455	358.67	0.25
14.088	833.85	0.34	13.450	750.65	0.32	13.471	530.01	0.28	13.475	359.59	0.25
14.106	834.04	0.34	13.470	751.09	0.32	13.491	531.31	0.28	13.495	360.50	0.25
14.124	834.22	0.34	13.490	751.53	0.32	13.511	532.44	0.28	13.515	361.36	0.25
14.142	834.45	0.34	13.510	751.98	0.32	13.531	533.57	0.28	13.535	362.21	0.25
14.160	834.66	0.34	13.529	752.42	0.32	13.551	534.71	0.28	13.555	363.06	0.25
14.178	834.84	0.34	13.549	752.86	0.32	13.571	535.81	0.28	13.575	363.91	0.25
14.196	835.03	0.34	13.569	753.30	0.32	13.591	536.91	0.28	13.595	364.76	0.25
14.214	835.23	0.34	13.589	753.74	0.32	13.611	538.01	0.28	13.615	365.66	0.25
14.232	835.44	0.34	13.609	754.18	0.32	13.631	539.01	0.28	13.635	366.55	0.25
14.250	835.64	0.34	13.629	754.59	0.32	13.651	540.01	0.28	13.654	367.45	0.25
14.268	835.83	0.34	13.649	754.92	0.32	13.671	541.01	0.28	13.674	368.34	0.25
14.286	836.04	0.34	13.669	755.30	0.32	13.691	542.01	0.28	13.694	369.24	0.25
14.304	836.26	0.34	13.689	755.68	0.32	13.711	543.01	0.28	13.714	370.12	0.25
14.322	836.48	0.34	13.709	756.08	0.32	13.731	543.96	0.28	13.734	371.01	0.25
14.340	836.74	0.34	13.729	756.46	0.32	13.751	544.91	0.28	13.754	371.89	0.25
14.358	836.96	0.34	13.748	756.89	0.32	13.771	546.21	0.28	13.774	372.78	0.25
14.376	837.13	0.34	13.768	757.29	0.32	13.790	547.50	0.28	13.794	373.66	0.25
14.394	837.29	0.34	13.788	757.68	0.32	13.810	548.41	0.28	13.814	374.53	0.25
14.412	837.50	0.34	13.808	758.10	0.32	13.830	549.31	0.28	13.834	375.40	0.25
14.429	837.74	0.34	13.828	758.53	0.32	13.850	550.21	0.28	13.854	376.27	0.25
14.447	837.98	0.34	13.848	758.90	0.32	13.870	551.26	0.28	13.874	377.15	0.25
14.465	838.19	0.34	13.868	759.30	0.32	13.890	552.31	0.28	13.894	378.01	0.25
14.483	838.40	0.34	13.888	759.71	0.32	13.910	553.27	0.28	13.914	378.84	0.25
14.501	838.57	0.34	13.908	760.08	0.32	13.930	554.24	0.28	13.934	379.67	0.25
14.519	838.74	0.34	13.928	760.49	0.33	13.950	555.20	0.28	13.953	380.50	0.25
14.537	838.94	0.34	13.948	760.83	0.33	13.970	556.14	0.28	13.973	381.32	0.25
14.555	839.16	0.34	13.967	761.22	0.33	13.990	557.07	0.28	13.993	382.15	0.25
14.573	839.36	0.34	13.987	761.60	0.33	14.010	558.01	0.28	14.013	382.96	0.25
14.591	839.55	0.34	14.007	761.96	0.33	14.030	559.01	0.28	14.033	383.78	0.25
14.609	839.75	0.34	14.027	762.32	0.33	14.050	560.01	0.28	14.053	384.59	0.25
14.627	839.97	0.34	14.047	762.73	0.33	14.070	561.01	0.28	14.073	385.41	0.25
14.645	840.15	0.34	14.067	763.10	0.33	14.090	562.01	0.28	14.093	386.31	0.25
14.663	840.36	0.34	14.087	763.44	0.33	14.110	563.01	0.28	14.113	387.16	0.25
14.681	840.56	0.34	14.107	763.77	0.33	14.129	564.01	0.28	14.133	388.10	0.25
14.699	840.73	0.34	14.127	764.15	0.33	14.149	565.01	0.28	14.153	389.04	0.25
14.717	840.94	0.34	14.147	764.53	0.33	14.169	566.01	0.28	14.173	389.98	0.25
14.735	841.15	0.34	14.167	764.90	0.33	14.189	567.01	0.28	14.193	390.91	0.25
14.753	841.35	0.34	14.186	765.22	0.33	14.209	568.01	0.28	14.213	391.78	0.26
14.771	841.53	0.34	14.206	765.54	0.33	14.229	569.01	0.29	14.233	392.64	0.26
14.789	841.71	0.34	14.226	765.90	0.33	14.249	570.00	0.29	14.252	393.50	0.26

14.807	841.91	0.34	14.246	766.25	0.33	14.269	570.84	0.29	14.272	394.36	0.26
14.825	842.11	0.34	14.266	766.60	0.33	14.289	571.67	0.29	14.292	395.22	0.26
14.843	842.32	0.34	14.286	766.93	0.33	14.309	572.50	0.29	14.312	396.10	0.26
14.861	842.53	0.34	14.306	767.27	0.33	14.329	573.25	0.29	14.332	396.98	0.26
14.879	842.71	0.34	14.326	767.63	0.33	14.349	574.00	0.29	14.352	397.85	0.26
14.897	842.87	0.34	14.346	767.98	0.33	14.369	574.84	0.29	14.372	398.73	0.26
14.915	843.07	0.34	14.366	768.28	0.33	14.389	575.67	0.29	14.392	399.61	0.26
14.933	843.26	0.34	14.386	768.59	0.33	14.409	576.51	0.29	14.412	400.50	0.26
14.951	843.46	0.34	14.406	768.90	0.33	14.429	577.40	0.29	14.432	401.39	0.26
14.969	843.68	0.34	14.425	769.24	0.33	14.448	578.30	0.29	14.452	402.27	0.26
14.987	843.90	0.34	14.445	769.59	0.33	14.468	579.14	0.29	14.472	403.16	0.26
15.005	844.10	0.34	14.465	769.89	0.33	14.488	579.97	0.29	14.492	404.04	0.26
15.022	844.29	0.34	14.485	770.19	0.33	14.508	580.80	0.29	14.512	404.85	0.26
15.040	844.48	0.34	14.505	770.51	0.33	14.528	581.66	0.29	14.532	405.66	0.26
15.058	844.66	0.34	14.525	770.84	0.33	14.548	582.52	0.29	14.551	406.46	0.26
15.076	844.84	0.34	14.545	771.15	0.33	14.568	583.38	0.29	14.571	407.27	0.26
15.094	845.02	0.34	14.565	771.45	0.33	14.588	584.24	0.29	14.591	408.08	0.26
15.112	845.19	0.34	14.585	771.71	0.33	14.608	585.10	0.29	14.611	408.87	0.26
15.130	845.37	0.34	14.605	771.97	0.33	14.628	585.98	0.29	14.631	409.65	0.26
15.148	845.54	0.34	14.625	772.33	0.33	14.648	586.86	0.29	14.651	410.45	0.26
15.166	845.74	0.34	14.644	772.70	0.33	14.668	587.74	0.29	14.671	411.32	0.26
15.184	845.94	0.34	14.664	772.99	0.33	14.688	588.62	0.29	14.691	412.03	0.26
15.202	846.11	0.34	14.684	773.34	0.33	14.708	589.50	0.29	14.711	412.98	0.26
15.220	846.28	0.34	14.704	773.70	0.33	14.728	590.30	0.29	14.731	413.84	0.26
15.238	846.44	0.34	14.724	774.02	0.33	14.748	591.10	0.29	14.751	414.61	0.26
15.256	846.61	0.34	14.744	774.32	0.33	14.767	591.90	0.29	14.771	415.48	0.26
15.274	846.78	0.34	14.764	774.67	0.33	14.787	592.70	0.29	14.791	416.35	0.26
15.292	846.96	0.34	14.784	775.02	0.33	14.807	593.50	0.29	14.811	417.21	0.26
15.310	847.14	0.34	14.804	775.35	0.33	14.827	594.30	0.29	14.831	418.03	0.26
15.328	847.32	0.34	14.824	775.68	0.33	14.847	595.10	0.29	14.850	418.85	0.26
15.346	847.52	0.34	14.844	776.01	0.33	14.867	595.90	0.29	14.870	419.67	0.26
15.364	847.74	0.34	14.863	776.33	0.33	14.887	596.70	0.29	14.890	420.49	0.26
15.382	847.93	0.34	14.883	776.65	0.33	14.907	597.50	0.29	14.910	421.31	0.26
15.400	848.10	0.34	14.903	776.96	0.33	14.927	598.24	0.29	14.930	422.11	0.26
15.418	848.27	0.34	14.923	777.27	0.33	14.947	598.98	0.29	14.950	422.91	0.26
15.436	848.44	0.34	14.943	777.58	0.33	14.967	599.72	0.29	14.970	423.72	0.26
15.454	848.65	0.34	14.963	777.88	0.33	14.987	600.46	0.29	14.990	424.52	0.26
15.472	848.84	0.34	14.983	778.25	0.33	15.007	601.20	0.29	15.010	425.32	0.26
15.490	849.01	0.34	15.003	778.56	0.33	15.027	601.98	0.29	15.030	426.18	0.26
15.508	849.19	0.34	15.023	778.86	0.33	15.047	602.76	0.29	15.050	427.04	0.26
15.526	849.39	0.34	15.043	779.19	0.33	15.067	603.54	0.29	15.070	427.89	0.26
15.544	849.59	0.34	15.063	779.48	0.33	15.086	604.32	0.29	15.090	428.75	0.26
15.562	849.77	0.35	15.082	779.76	0.33	15.106	605.10	0.29	15.110	429.61	0.26
15.580	849.91	0.35	15.102	780.09	0.33	15.126	605.86	0.29	15.130	430.46	0.26
15.597	850.09	0.35	15.122	780.36	0.33	15.146	606.62	0.29	15.149	431.31	0.26

15.615	850.28	0.35	15.142	780.64	0.33	15.166	607.38	0.29	15.169	432.16	0.26
15.633	850.47	0.35	15.162	780.96	0.33	15.186	608.14	0.29	15.189	433.01	0.26
15.651	850.66	0.35	15.182	781.28	0.33	15.206	608.90	0.29	15.209	433.86	0.26
15.669	850.85	0.35	15.202	781.58	0.33	15.226	609.58	0.29	15.229	434.67	0.26
15.687	851.01	0.35	15.222	781.85	0.33	15.246	610.26	0.29	15.249	435.48	0.26
15.705	851.17	0.35	15.242	782.14	0.33	15.266	610.94	0.29	15.269	436.29	0.26
15.723	851.36	0.35	15.262	782.44	0.33	15.286	611.62	0.29	15.289	437.10	0.26
15.741	851.57	0.35	15.282	782.74	0.33	15.306	612.30	0.29	15.309	437.91	0.26
15.759	851.72	0.35	15.301	783.03	0.33	15.326	612.96	0.29	15.329	438.72	0.26
15.777	851.86	0.35	15.321	783.32	0.33	15.346	613.62	0.29	15.349	439.52	0.26
15.795	852.03	0.35	15.341	783.61	0.33	15.366	614.28	0.29	15.369	440.33	0.26
15.813	852.20	0.35	15.361	783.89	0.33	15.386	614.94	0.29	15.389	441.14	0.26
15.831	852.35	0.35	15.381	784.22	0.33	15.405	615.60	0.29	15.409	441.95	0.26
15.849	852.52	0.35	15.401	784.53	0.33	15.425	616.24	0.29	15.429	442.77	0.26
15.867	852.70	0.35	15.421	784.80	0.33	15.445	616.88	0.29	15.448	443.60	0.26
15.885	852.88	0.35	15.441	785.05	0.33	15.465	617.52	0.29	15.468	444.43	0.26
15.903	853.07	0.35	15.461	785.26	0.33	15.485	618.16	0.29	15.488	445.25	0.26
15.921	853.26	0.35	15.481	785.53	0.33	15.505	618.80	0.29	15.508	446.08	0.26
15.939	853.44	0.35	15.501	785.83	0.33	15.525	619.52	0.29	15.528	446.89	0.26
15.957	853.62	0.35	15.520	786.09	0.33	15.545	620.24	0.30	15.548	447.70	0.26
15.975	853.79	0.35	15.540	786.41	0.33	15.565	620.96	0.30	15.568	448.52	0.26
15.993	853.96	0.35	15.560	786.69	0.33	15.585	621.68	0.30	15.588	449.33	0.26
16.011	854.15	0.35	15.580	786.98	0.33	15.605	622.40	0.30	15.608	450.15	0.26
16.029	854.36	0.35	15.600	787.29	0.33	15.625	623.04	0.30	15.628	451.02	0.26
16.047	854.54	0.35	15.620	787.54	0.33	15.645	623.68	0.30	15.648	451.90	0.26
16.065	854.70	0.35	15.640	787.81	0.33	15.665	624.32	0.30	15.668	452.77	0.26
16.083	854.87	0.35	15.660	788.12	0.33	15.685	624.96	0.30	15.688	453.55	0.26
16.101	855.05	0.35	15.680	788.41	0.33	15.705	625.60	0.30	15.708	454.42	0.26
16.119	855.25	0.35	15.700	788.67	0.33	15.724	626.28	0.30	15.728	455.26	0.26
16.137	855.40	0.35	15.720	788.99	0.33	15.744	626.96	0.30	15.747	456.00	0.26
16.155	855.56	0.35	15.739	789.24	0.33	15.764	627.64	0.30	15.767	456.74	0.26
16.173	855.75	0.35	15.759	789.53	0.33	15.784	628.32	0.30	15.787	457.48	0.27
16.190	855.91	0.35	15.779	789.84	0.33	15.804	629.00	0.30	15.807	458.23	0.27
16.208	856.06	0.35	15.799	790.16	0.33	15.824	629.58	0.30	15.827	459.14	0.27
16.226	856.23	0.35	15.819	790.41	0.33	15.844	630.16	0.30	15.847	459.88	0.27
16.244	856.41	0.35	15.839	790.69	0.33	15.864	630.74	0.30	15.867	460.71	0.27
16.262	856.58	0.35	15.859	790.98	0.33	15.884	631.32	0.30	15.887	461.54	0.27
16.280	856.75	0.35	15.879	791.28	0.33	15.904	631.90	0.30	15.907	462.37	0.27
16.298	856.92	0.35	15.899	791.53	0.33	15.924	632.52	0.30	15.927	463.22	0.27
16.316	857.09	0.35	15.919	791.79	0.33	15.944	633.15	0.30	15.947	464.06	0.27
16.334	857.26	0.35	15.939	792.09	0.33	15.964	633.77	0.30	15.967	464.90	0.27
16.352	857.42	0.35	15.958	792.34	0.33	15.984	634.39	0.30	15.987	465.74	0.27
16.370	857.55	0.35	15.978	792.62	0.33	16.004	635.01	0.30	16.007	466.58	0.27
16.388	857.71	0.35	15.998	792.90	0.33	16.024	635.54	0.30	16.027	467.33	0.27
16.406	857.86	0.35	16.018	793.16	0.33	16.043	636.07	0.30	16.046	468.07	0.27

16.424	858.02	0.35	16.038	793.43	0.33	16.063	636.72	0.30	16.066	468.81	0.27
16.442	858.20	0.35	16.058	793.72	0.33	16.083	637.36	0.30	16.086	469.56	0.27
16.460	858.40	0.35	16.078	794.06	0.33	16.103	638.01	0.30	16.106	470.30	0.27
16.478	858.57	0.35	16.098	794.32	0.33	16.123	638.54	0.30	16.126	471.07	0.27
16.496	858.70	0.35	16.118	794.56	0.33	16.143	639.08	0.30	16.146	471.83	0.27
16.514	858.88	0.35	16.138	794.81	0.33	16.163	639.62	0.30	16.166	472.60	0.27
16.532	859.07	0.35	16.158	795.06	0.33	16.183	640.17	0.30	16.186	473.36	0.27
16.550	859.25	0.35	16.177	795.37	0.33	16.203	640.72	0.30	16.206	474.13	0.27
16.568	859.43	0.35	16.197	795.66	0.33	16.223	641.27	0.30	16.226	474.90	0.27
16.586	859.60	0.35	16.217	795.94	0.33	16.243	641.83	0.30	16.246	475.68	0.27
16.604	859.74	0.35	16.237	796.17	0.33	16.263	642.40	0.30	16.266	476.46	0.27
16.622	859.88	0.35	16.257	796.45	0.33	16.283	642.96	0.30	16.286	477.23	0.27
16.640	860.05	0.35	16.277	796.74	0.33	16.303	643.53	0.30	16.306	478.01	0.27
16.658	860.22	0.35	16.297	797.02	0.33	16.323	644.09	0.30	16.326	478.77	0.27
16.676	860.39	0.35	16.317	797.25	0.33	16.343	644.66	0.30	16.345	479.53	0.27
16.694	860.55	0.35	16.337	797.51	0.33	16.362	645.21	0.30	16.365	480.30	0.27
16.712	860.76	0.35	16.357	797.78	0.33	16.382	645.76	0.30	16.385	481.06	0.27
16.730	860.94	0.35	16.377	798.05	0.33	16.402	646.32	0.30	16.405	481.82	0.27
16.748	861.10	0.35	16.396	798.31	0.33	16.422	646.89	0.30	16.425	482.56	0.27
16.766	861.25	0.35	16.416	798.58	0.33	16.442	647.46	0.30	16.445	483.30	0.27
16.783	861.42	0.35	16.436	798.83	0.33	16.462	648.03	0.30	16.465	484.03	0.27
16.801	861.61	0.35	16.456	799.09	0.33	16.482	648.55	0.30	16.485	484.77	0.27
16.819	861.78	0.35	16.476	799.33	0.33	16.502	649.08	0.30	16.505	485.50	0.27
16.837	861.92	0.35	16.496	799.57	0.33	16.522	649.61	0.30	16.525	486.28	0.27
16.855	862.07	0.35	16.516	799.81	0.33	16.542	650.13	0.30	16.545	487.06	0.27
16.873	862.21	0.35	16.536	800.07	0.33	16.562	650.64	0.30	16.565	487.83	0.27
16.891	862.39	0.35	16.556	800.33	0.33	16.582	651.16	0.30	16.585	488.61	0.27
16.909	862.56	0.35	16.576	800.60	0.33	16.602	651.70	0.30	16.605	489.38	0.27
16.927	862.73	0.35	16.596	800.84	0.33	16.622	652.25	0.30	16.625	490.13	0.27
16.945	862.90	0.35	16.615	801.05	0.33	16.642	652.80	0.30	16.644	490.89	0.27
16.963	863.06	0.35	16.635	801.28	0.33	16.662	653.32	0.30	16.664	491.64	0.27
16.981	863.21	0.35	16.655	801.54	0.33	16.681	653.84	0.30	16.684	492.38	0.27
16.999	863.33	0.35	16.675	801.79	0.33	16.701	654.36	0.30	16.704	493.14	0.27
17.017	863.46	0.35	16.695	802.05	0.33	16.721	654.91	0.30	16.724	493.90	0.27
17.035	863.62	0.35	16.715	802.24	0.33	16.741	655.46	0.30	16.744	494.67	0.27
17.053	863.80	0.35	16.735	802.41	0.33	16.761	656.01	0.30	16.764	495.44	0.27
17.071	863.98	0.35	16.755	802.68	0.33	16.781	656.47	0.30	16.784	496.21	0.27
17.089	864.14	0.35	16.775	802.93	0.33	16.801	656.93	0.30	16.804	496.98	0.27
17.107	864.26	0.35	16.795	803.19	0.33	16.821	657.39	0.30	16.824	497.68	0.27
17.125	864.40	0.35	16.815	803.44	0.33	16.841	657.93	0.30	16.844	498.39	0.27
17.143	864.56	0.35	16.834	803.70	0.33	16.861	658.47	0.30	16.864	499.09	0.27
17.161	864.71	0.35	16.854	803.94	0.33	16.881	659.00	0.30	16.884	499.79	0.27
17.179	864.89	0.35	16.874	804.23	0.33	16.901	659.47	0.30	16.904	500.49	0.27
17.197	865.09	0.35	16.894	804.49	0.33	16.921	659.94	0.30	16.924	501.20	0.27
17.215	865.25	0.35	16.914	804.68	0.33	16.941	660.41	0.30	16.943	501.91	0.27

17.233	865.39	0.35	16.934	804.98	0.33	16.961	660.88	0.30	16.963	502.62	0.27
17.251	865.53	0.35	16.954	805.24	0.33	16.981	661.36	0.30	16.983	503.32	0.27
17.269	865.68	0.35	16.974	805.48	0.33	17.000	661.84	0.30	17.003	504.03	0.27
17.287	865.82	0.35	16.994	805.72	0.34	17.020	662.30	0.30	17.023	504.73	0.27
17.305	865.99	0.35	17.014	805.95	0.34	17.040	662.77	0.30	17.043	505.43	0.27
17.323	866.16	0.35	17.034	806.18	0.34	17.060	663.23	0.30	17.063	506.13	0.27
17.341	866.31	0.35	17.053	806.41	0.34	17.080	663.70	0.30	17.083	506.82	0.27
17.359	866.43	0.35	17.073	806.69	0.34	17.100	664.16	0.30	17.103	507.52	0.27
17.376	866.56	0.35	17.093	806.94	0.34	17.120	664.62	0.30	17.123	508.26	0.27
17.394	866.71	0.35	17.113	807.17	0.34	17.140	665.11	0.30	17.143	509.00	0.27
17.412	866.91	0.35	17.133	807.39	0.34	17.160	665.59	0.30	17.163	509.73	0.27
17.430	867.10	0.35	17.153	807.62	0.34	17.180	666.08	0.30	17.183	510.47	0.27
17.448	867.23	0.35	17.173	807.85	0.34	17.200	666.56	0.30	17.203	511.20	0.27
17.466	867.36	0.35	17.193	808.08	0.34	17.220	667.04	0.30	17.223	511.86	0.27
17.484	867.52	0.35	17.213	808.37	0.34	17.240	667.52	0.30	17.242	512.52	0.27
17.502	867.70	0.35	17.233	808.59	0.34	17.260	667.96	0.30	17.262	513.17	0.27
17.520	867.87	0.35	17.253	808.80	0.34	17.280	668.39	0.30	17.282	513.83	0.27
17.538	868.02	0.35	17.272	809.07	0.34	17.300	668.83	0.30	17.302	514.49	0.27
17.556	868.16	0.35	17.292	809.29	0.34	17.320	669.36	0.30	17.322	515.19	0.28
17.574	868.34	0.35	17.312	809.53	0.34	17.339	669.89	0.31	17.342	515.89	0.28
17.592	868.51	0.35	17.332	809.80	0.34	17.359	670.42	0.31	17.362	516.60	0.28
17.610	868.67	0.35	17.352	810.07	0.34	17.379	670.88	0.31	17.382	517.29	0.28
17.628	868.82	0.35	17.372	810.28	0.34	17.399	671.34	0.31	17.402	517.83	0.28
17.646	868.93	0.35	17.392	810.48	0.34	17.419	671.81	0.31	17.422	518.59	0.28
17.664	869.08	0.35	17.412	810.74	0.34	17.439	672.24	0.31	17.442	519.28	0.28
17.682	869.24	0.35	17.432	810.99	0.34	17.459	672.67	0.31	17.462	520.00	0.28
17.700	869.40	0.35	17.452	811.24	0.34	17.479	673.10	0.31	17.482	520.68	0.28
17.718	869.56	0.35	17.472	811.48	0.34	17.499	673.58	0.31	17.502	521.38	0.28
17.736	869.72	0.35	17.491	811.72	0.34	17.519	674.05	0.31	17.522	522.04	0.28
17.754	869.87	0.35	17.511	811.93	0.34	17.539	674.52	0.31	17.541	522.71	0.28
17.772	870.03	0.35	17.531	812.12	0.34	17.559	674.95	0.31	17.561	523.38	0.28
17.790	870.22	0.35	17.551	812.37	0.34	17.579	675.38	0.31	17.581	524.04	0.28
17.808	870.37	0.35	17.571	812.62	0.34	17.599	675.81	0.31	17.601	524.71	0.28
17.826	870.50	0.35	17.591	812.86	0.34	17.619	676.32	0.31	17.621	525.38	0.28
17.844	870.63	0.35	17.611	813.09	0.34	17.639	676.83	0.31	17.641	526.04	0.28
17.862	870.77	0.35	17.631	813.32	0.34	17.658	677.34	0.31	17.661	526.70	0.28
17.880	870.93	0.35	17.651	813.55	0.34	17.678	677.70	0.31	17.681	527.35	0.28
17.898	871.08	0.35	17.671	813.77	0.34	17.698	678.07	0.31	17.701	528.01	0.28
17.916	871.21	0.35	17.691	813.99	0.34	17.718	678.43	0.31	17.721	528.69	0.28
17.934	871.34	0.35	17.710	814.21	0.34	17.738	678.89	0.31	17.741	529.36	0.28
17.951	871.47	0.35	17.730	814.45	0.34	17.758	679.35	0.31	17.761	530.04	0.28
17.969	871.60	0.35	17.750	814.71	0.34	17.778	679.81	0.31	17.781	530.68	0.28
17.987	871.76	0.35	17.770	814.93	0.34	17.798	680.26	0.31	17.801	531.32	0.28
18.005	871.95	0.35	17.790	815.15	0.34	17.818	680.72	0.31	17.821	531.96	0.28
18.023	872.12	0.35	17.810	815.40	0.34	17.838	681.18	0.31	17.840	532.57	0.28

18.041	872.26	0.35	17.830	815.61	0.34	17.858	681.61	0.31	17.860	533.19	0.28
18.059	872.42	0.35	17.850	815.80	0.34	17.878	682.04	0.31	17.880	533.81	0.28
18.077	872.57	0.35	17.870	816.04	0.34	17.898	682.48	0.31	17.900	534.44	0.28
18.095	872.69	0.35	17.890	816.27	0.34	17.918	682.92	0.31	17.920	535.06	0.28
18.113	872.83	0.35	17.910	816.47	0.34	17.938	683.37	0.31	17.940	535.69	0.28
18.131	872.99	0.35	17.929	816.68	0.34	17.958	683.82	0.31	17.960	536.31	0.28
18.149	873.15	0.35	17.949	816.90	0.34	17.977	684.22	0.31	17.980	536.93	0.28
18.167	873.30	0.35	17.969	817.13	0.34	17.997	684.61	0.31	18.000	537.55	0.28
18.185	873.45	0.35	17.989	817.36	0.34	18.017	685.01	0.31	18.020	538.18	0.28
18.203	873.60	0.35	18.009	817.55	0.34	18.037	685.45	0.31	18.040	538.83	0.28
18.221	873.74	0.35	18.029	817.73	0.34	18.057	685.89	0.31	18.060	539.46	0.28
18.239	873.88	0.35	18.049	817.89	0.34	18.077	686.33	0.31	18.080	540.04	0.28
18.257	874.04	0.35	18.069	818.13	0.34	18.097	686.75	0.31	18.100	540.65	0.28
18.275	874.21	0.35	18.089	818.38	0.34	18.117	687.18	0.31	18.120	541.25	0.28
18.293	874.35	0.35	18.109	818.61	0.34	18.137	687.60	0.31	18.139	541.90	0.28
18.311	874.48	0.35	18.129	818.86	0.34	18.157	688.01	0.31	18.159	542.51	0.28
18.329	874.60	0.35	18.148	819.00	0.34	18.177	688.42	0.31	18.179	543.13	0.28
18.347	874.73	0.35	18.168	819.30	0.34	18.197	688.82	0.31	18.199	543.75	0.28
18.365	874.87	0.35	18.188	819.54	0.34	18.217	689.22	0.31	18.219	544.34	0.28
18.383	875.01	0.35	18.208	819.76	0.34	18.237	689.61	0.31	18.239	544.91	0.28
18.401	875.14	0.35	18.228	820.01	0.34	18.257	690.01	0.31	18.259	545.56	0.28
18.419	875.29	0.35	18.248	820.19	0.34	18.277	690.38	0.31	18.279	546.20	0.28
18.437	875.46	0.35	18.268	820.42	0.34	18.296	690.75	0.31	18.299	546.82	0.28
18.455	875.61	0.35	18.288	820.64	0.34	18.316	691.12	0.31	18.319	547.41	0.28
18.473	875.73	0.35	18.308	820.83	0.34	18.336	691.57	0.31	18.339	547.97	0.28
18.491	875.84	0.35	18.328	821.07	0.34	18.356	692.02	0.31	18.359	548.58	0.28
18.509	875.99	0.35	18.348	821.28	0.34	18.376	692.47	0.31	18.379	549.16	0.28
18.527	876.13	0.35	18.367	821.50	0.34	18.396	692.83	0.31	18.399	549.72	0.28
18.544	876.28	0.35	18.387	821.73	0.34	18.416	693.19	0.31	18.419	550.31	0.28
18.562	876.42	0.35	18.407	821.91	0.34	18.436	693.55	0.31	18.439	550.91	0.28
18.580	876.55	0.35	18.427	822.15	0.34	18.456	693.96	0.31	18.458	551.50	0.28
18.598	876.69	0.35	18.447	822.35	0.34	18.476	694.38	0.31	18.478	552.10	0.28
18.616	876.83	0.35	18.467	822.53	0.34	18.496	694.79	0.31	18.498	552.68	0.28
18.634	876.98	0.35	18.487	822.77	0.34	18.516	695.14	0.31	18.518	553.28	0.28
18.652	877.12	0.35	18.507	823.00	0.34	18.536	695.54	0.31	18.538	553.88	0.28
18.670	877.26	0.35	18.527	823.21	0.34	18.556	695.95	0.31	18.558	554.45	0.28
18.688	877.43	0.35	18.547	823.39	0.34	18.576	696.36	0.31	18.578	554.98	0.28
18.706	877.55	0.35	18.567	823.61	0.34	18.596	696.73	0.31	18.598	555.57	0.28
18.724	877.69	0.35	18.586	823.83	0.34	18.615	697.10	0.31	18.618	556.17	0.28
18.742	877.85	0.35	18.606	824.05	0.34	18.635	697.49	0.31	18.638	556.72	0.28
18.760	878.01	0.35	18.626	824.26	0.34	18.655	697.89	0.31	18.658	557.27	0.28
18.778	878.14	0.35	18.646	824.48	0.34	18.675	698.28	0.31	18.678	557.82	0.28
18.796	878.27	0.35	18.666	824.69	0.34	18.695	698.63	0.31	18.698	558.38	0.28
18.814	878.41	0.35	18.686	824.89	0.34	18.715	698.99	0.31	18.718	558.97	0.28
18.832	878.55	0.35	18.706	825.10	0.34	18.735	699.36	0.31	18.738	559.52	0.28

18.850	878.67	0.35	18.726	825.30	0.34	18.755	699.73	0.31	18.757	560.04	0.28
18.868	878.80	0.35	18.746	825.49	0.34	18.775	700.10	0.31	18.777	560.60	0.28
18.886	878.94	0.35	18.766	825.68	0.34	18.795	700.47	0.31	18.797	561.15	0.28
18.904	879.10	0.35	18.786	825.87	0.34	18.815	700.83	0.31	18.817	561.67	0.28
18.922	879.26	0.35	18.805	826.06	0.34	18.835	701.21	0.31	18.837	562.21	0.28
18.940	879.38	0.35	18.825	826.30	0.34	18.855	701.59	0.31	18.857	562.77	0.28
18.958	879.49	0.35	18.845	826.51	0.34	18.875	701.96	0.31	18.877	563.32	0.28
18.976	879.64	0.35	18.865	826.69	0.34	18.895	702.33	0.31	18.897	563.86	0.28
18.994	879.80	0.35	18.885	826.85	0.34	18.915	702.69	0.31	18.917	564.40	0.28
19.012	879.97	0.35	18.905	827.01	0.34	18.934	703.05	0.31	18.937	564.91	0.28
19.030	880.10	0.35	18.925	827.21	0.34	18.954	703.40	0.31	18.957	565.39	0.28
19.048	880.22	0.35	18.945	827.41	0.34	18.974	703.76	0.31	18.977	565.82	0.28
19.066	880.38	0.35	18.965	827.61	0.34	18.994	704.12	0.31	18.997	566.36	0.28
19.084	880.53	0.35	18.985	827.81	0.34	19.014	704.48	0.31	19.017	566.94	0.28
19.102	880.68	0.35	19.005	828.02	0.34	19.034	704.83	0.31	19.037	567.48	0.28
19.120	880.82	0.35	19.024	828.26	0.34	19.054	705.18	0.31	19.056	568.01	0.28
19.137	880.95	0.35	19.044	828.46	0.34	19.074	705.58	0.31	19.076	568.58	0.28
19.155	881.08	0.35	19.064	828.66	0.34	19.094	705.94	0.31	19.096	569.16	0.28
19.173	881.21	0.35	19.084	828.87	0.34	19.114	706.28	0.31	19.116	569.72	0.29
19.191	881.33	0.35	19.104	829.06	0.34	19.134	706.61	0.31	19.136	570.26	0.29
19.209	881.45	0.35	19.124	829.26	0.34	19.154	706.99	0.31	19.156	570.76	0.29
19.227	881.58	0.35	19.144	829.46	0.34	19.174	707.35	0.31	19.176	571.30	0.29
19.245	881.71	0.35	19.164	829.66	0.34	19.194	707.68	0.31	19.196	571.85	0.29
19.263	881.85	0.35	19.184	829.85	0.34	19.214	708.06	0.31	19.216	572.40	0.29
19.281	882.00	0.35	19.204	830.05	0.34	19.234	708.41	0.31	19.236	572.94	0.29
19.299	882.14	0.35	19.224	830.32	0.34	19.253	708.73	0.31	19.256	573.47	0.29
19.317	882.28	0.35	19.243	830.50	0.34	19.273	709.04	0.31	19.276	573.99	0.29
19.335	882.42	0.35	19.263	830.69	0.34	19.293	709.37	0.31	19.296	574.53	0.29
19.353	882.56	0.35	19.283	830.88	0.34	19.313	709.73	0.31	19.316	575.05	0.29
19.371	882.69	0.35	19.303	831.06	0.34	19.333	710.05	0.31	19.336	575.57	0.29
19.389	882.82	0.35	19.323	831.29	0.34	19.353	710.36	0.31	19.355	576.08	0.29
19.407	882.95	0.35	19.343	831.49	0.34	19.373	710.66	0.31	19.375	576.60	0.29
19.425	883.07	0.35	19.363	831.68	0.34	19.393	711.00	0.31	19.395	577.15	0.29
19.443	883.19	0.35	19.383	831.90	0.34	19.413	711.34	0.31	19.415	577.68	0.29
19.461	883.36	0.35	19.403	832.09	0.34	19.433	711.68	0.31	19.435	578.19	0.29
19.479	883.51	0.35	19.423	832.28	0.34	19.453	712.03	0.31	19.455	578.70	0.29
19.497	883.63	0.35	19.443	832.50	0.34	19.473	712.36	0.31	19.475	579.21	0.29
19.515	883.75	0.35	19.462	832.68	0.34	19.493	712.69	0.31	19.495	579.76	0.29
19.533	883.90	0.35	19.482	832.85	0.34	19.513	713.03	0.31	19.515	580.28	0.29
19.551	884.05	0.35	19.502	833.02	0.34	19.533	713.38	0.31	19.535	580.77	0.29
19.569	884.20	0.35	19.522	833.24	0.34	19.553	713.71	0.31	19.555	581.31	0.29
19.587	884.34	0.35	19.542	833.43	0.34	19.572	714.07	0.31	19.575	581.80	0.29
19.605	884.47	0.35	19.562	833.61	0.34	19.592	714.45	0.31	19.595	582.31	0.29
19.623	884.59	0.35	19.582	833.83	0.34	19.612	714.77	0.31	19.615	582.83	0.29
19.641	884.71	0.35	19.602	834.02	0.34	19.632	715.09	0.31	19.635	583.30	0.29

19.659	884.81	0.35	19.622	834.20	0.34	19.652	715.42	0.31	19.654	583.81	0.29
19.677	884.92	0.35	19.642	834.42	0.34	19.672	715.74	0.31	19.674	584.32	0.29
19.695	885.05	0.35	19.662	834.59	0.34	19.692	716.07	0.31	19.694	584.80	0.29
19.712	885.19	0.35	19.681	834.75	0.34	19.712	716.38	0.31	19.714	585.31	0.29
19.730	885.33	0.35	19.701	834.95	0.34	19.732	716.74	0.31	19.734	585.83	0.29
19.748	885.46	0.35	19.721	835.15	0.34	19.752	717.09	0.32	19.754	586.35	0.29
19.766	885.59	0.35	19.741	835.35	0.34	19.772	717.40	0.32	19.774	586.86	0.29
19.784	885.73	0.35	19.761	835.54	0.34	19.792	717.70	0.32	19.794	587.31	0.29
19.802	885.87	0.35	19.781	835.72	0.34	19.812	718.05	0.32	19.814	587.78	0.29
19.820	886.00	0.35	19.801	835.87	0.34	19.832	718.38	0.32	19.834	588.27	0.29
19.838	886.17	0.35	19.821	836.06	0.34	19.852	718.69	0.32	19.854	588.78	0.29
19.856	886.33	0.35	19.841	836.30	0.34	19.872	719.00	0.32	19.874	589.27	0.29
19.874	886.46	0.35	19.861	836.46	0.34	19.891	719.35	0.32	19.894	589.75	0.29
19.892	886.57	0.35	19.881	836.61	0.34	19.911	719.68	0.32	19.914	590.19	0.29
19.910	886.70	0.35	19.900	836.83	0.34	19.931	719.98	0.32	19.934	590.66	0.29
19.928	886.84	0.35	19.920	837.05	0.34	19.951	720.28	0.32	19.953	591.14	0.29
19.946	886.98	0.35	19.940	837.22	0.34	19.971	720.62	0.32	19.973	591.62	0.29
19.964	887.11	0.35	19.960	837.40	0.34	19.991	720.97	0.32	19.993	592.12	0.29
19.982	887.23	0.35	19.980	837.57	0.34	20.011	721.30	0.32	20.013	592.64	0.29
20.000	887.35	0.35	20.000	837.74	0.34	20.031	721.60	0.32	20.033	593.11	0.29
						20.051	721.91	0.32	20.053	593.57	0.29
						20.071	722.24	0.32	20.073	594.02	0.29
						20.091	722.52	0.32	20.093	594.48	0.29
						20.111	722.84	0.32	20.113	594.95	0.29
						20.131	723.16	0.32	20.133	595.45	0.29
						20.151	723.43	0.32	20.153	595.92	0.29
						20.171	723.74	0.32	20.173	596.37	0.29
						20.191	724.06	0.32	20.193	596.84	0.29
						20.211	724.38	0.32	20.213	597.29	0.29
						20.230	724.71	0.32	20.233	597.72	0.29
						20.250	725.02	0.32	20.252	598.15	0.29
						20.270	725.32	0.32	20.272	598.59	0.29
						20.290	725.60	0.32	20.292	599.06	0.29
						20.310	725.86	0.32	20.312	599.53	0.29
						20.330	726.17	0.32	20.332	599.99	0.29
						20.350	726.47	0.32	20.352	600.43	0.29
						20.370	726.77	0.32	20.372	600.85	0.29
						20.390	727.05	0.32	20.392	601.29	0.29
						20.410	727.32	0.32	20.412	601.74	0.29
						20.430	727.61	0.32	20.432	602.18	0.29
						20.450	727.95	0.32	20.452	602.62	0.29
						20.470	728.22	0.32	20.472	603.06	0.29
						20.490	728.55	0.32	20.492	603.50	0.29
						20.510	728.85	0.32	20.512	603.94	0.29
						20.530	729.15	0.32	20.532	604.37	0.29

						20.549	729.47	0.32	20.551	604.81	0.29
						20.569	729.74	0.32	20.571	605.18	0.29
						20.589	730.06	0.32	20.591	605.50	0.29
						20.609	730.35	0.32	20.611	605.93	0.29
						20.629	730.66	0.32	20.631	606.38	0.29
						20.649	730.99	0.32	20.651	606.82	0.29
						20.669	731.30	0.32	20.671	607.26	0.29
						20.689	731.55	0.32	20.691	607.65	0.29
						20.709	731.85	0.32	20.711	608.09	0.29
						20.729	732.13	0.32	20.731	608.53	0.29
						20.749	732.41	0.32	20.751	608.96	0.29
						20.769	732.73	0.32	20.771	609.39	0.29
						20.789	733.05	0.32	20.791	609.81	0.29
						20.809	733.32	0.32	20.811	610.27	0.29
						20.829	733.60	0.32	20.831	610.74	0.29
						20.849	733.91	0.32	20.850	611.16	0.29
						20.868	734.24	0.32	20.870	611.58	0.29
						20.888	734.55	0.32	20.890	611.99	0.29
						20.908	734.82	0.32	20.910	612.39	0.29
						20.928	735.08	0.32	20.930	612.81	0.29
						20.948	735.39	0.32	20.950	613.20	0.29
						20.968	735.68	0.32	20.970	613.57	0.29
						20.988	735.97	0.32	20.990	613.97	0.29
						21.008	736.25	0.32	21.010	614.39	0.29
						21.028	736.50	0.32	21.030	614.84	0.29
						21.048	736.80	0.32	21.050	615.26	0.29
						21.068	737.10	0.32	21.070	615.70	0.29
						21.088	737.39	0.32	21.090	616.10	0.29
						21.108	737.67	0.32	21.110	616.55	0.29
						21.128	737.96	0.32	21.130	616.93	0.29
						21.148	738.26	0.32	21.149	617.38	0.29
						21.168	738.54	0.32	21.169	617.84	0.29
						21.187	738.82	0.32	21.189	618.22	0.29
						21.207	739.10	0.32	21.209	618.65	0.29
						21.227	739.38	0.32	21.229	619.10	0.29
						21.247	739.65	0.32	21.249	619.51	0.29
						21.267	739.93	0.32	21.269	619.93	0.29
						21.287	740.21	0.32	21.289	620.36	0.29
						21.307	740.47	0.32	21.309	620.75	0.29
						21.327	740.74	0.32	21.329	621.12	0.29
						21.347	741.00	0.32	21.349	621.56	0.30
						21.367	741.27	0.32	21.369	621.99	0.30
						21.387	741.56	0.32	21.389	622.40	0.30
						21.407	741.87	0.32	21.409	622.76	0.30
						21.427	742.13	0.32	21.429	623.18	0.30

						21.447	742.39	0.32	21.448	623.60	0.30
						21.467	742.65	0.32	21.468	624.02	0.30
						21.487	742.91	0.32	21.488	624.44	0.30
						21.506	743.17	0.32	21.508	624.86	0.30
						21.526	743.43	0.32	21.528	625.27	0.30
						21.546	743.72	0.32	21.548	625.68	0.30
						21.566	744.02	0.32	21.568	626.09	0.30
						21.586	744.27	0.32	21.588	626.49	0.30
						21.606	744.53	0.32	21.608	626.89	0.30
						21.626	744.78	0.32	21.628	627.29	0.30
						21.646	745.03	0.32	21.648	627.69	0.30
						21.666	745.30	0.32	21.668	628.09	0.30
						21.686	745.59	0.32	21.688	628.48	0.30
						21.706	745.85	0.32	21.708	628.87	0.30
						21.726	746.10	0.32	21.728	629.26	0.30
						21.746	746.38	0.32	21.747	629.65	0.30
						21.766	746.64	0.32	21.767	630.03	0.30
						21.786	746.88	0.32	21.787	630.48	0.30
						21.806	747.14	0.32	21.807	630.89	0.30
						21.825	747.41	0.32	21.827	631.27	0.30
						21.845	747.70	0.32	21.847	631.65	0.30
						21.865	747.97	0.32	21.867	632.03	0.30
						21.885	748.19	0.32	21.887	632.40	0.30
						21.905	748.41	0.32	21.907	632.82	0.30
						21.925	748.64	0.32	21.927	633.24	0.30
						21.945	748.88	0.32	21.947	633.61	0.30
						21.965	749.14	0.32	21.967	633.97	0.30
						21.985	749.40	0.32	21.987	634.34	0.30
						22.005	749.66	0.32	22.007	634.73	0.30
						22.025	749.91	0.32	22.027	635.15	0.30
						22.045	750.17	0.32	22.046	635.51	0.30
						22.065	750.42	0.32	22.066	635.90	0.30
						22.085	750.67	0.32	22.086	636.29	0.30
						22.105	750.92	0.32	22.106	636.64	0.30
						22.125	751.17	0.32	22.126	637.06	0.30
						22.144	751.43	0.32	22.146	637.44	0.30
						22.164	751.73	0.32	22.166	637.79	0.30
						22.184	752.00	0.32	22.186	638.18	0.30
						22.204	752.25	0.32	22.206	638.58	0.30
						22.224	752.51	0.32	22.226	638.95	0.30
						22.244	752.74	0.32	22.246	639.30	0.30
						22.264	752.98	0.32	22.266	639.68	0.30
						22.284	753.22	0.32	22.286	640.07	0.30
						22.304	753.46	0.32	22.306	640.46	0.30
						22.324	753.75	0.32	22.326	640.80	0.30

						22.344	754.01	0.32	22.345	641.16	0.30
						22.364	754.24	0.32	22.365	641.55	0.30
						22.384	754.47	0.32	22.385	641.92	0.30
						22.404	754.74	0.32	22.405	642.26	0.30
						22.424	755.00	0.32	22.425	642.60	0.30
						22.444	755.24	0.32	22.445	642.97	0.30
						22.463	755.51	0.32	22.465	643.35	0.30
						22.483	755.76	0.32	22.485	643.74	0.30
						22.503	755.99	0.32	22.505	644.11	0.30
						22.523	756.21	0.32	22.525	644.47	0.30
						22.543	756.46	0.32	22.545	644.83	0.30
						22.563	756.73	0.32	22.565	645.19	0.30
						22.583	756.95	0.32	22.585	645.55	0.30
						22.603	757.21	0.32	22.605	645.92	0.30
						22.623	757.47	0.32	22.625	646.28	0.30
						22.643	757.68	0.32	22.644	646.64	0.30
						22.663	757.93	0.32	22.664	646.99	0.30
						22.683	758.18	0.32	22.684	647.35	0.30
						22.703	758.40	0.32	22.704	647.69	0.30
						22.723	758.65	0.32	22.724	648.04	0.30
						22.743	758.91	0.32	22.744	648.38	0.30
						22.763	759.12	0.32	22.764	648.73	0.30
						22.782	759.35	0.32	22.784	649.07	0.30
						22.802	759.60	0.32	22.804	649.41	0.30
						22.822	759.84	0.32	22.824	649.76	0.30
						22.842	760.07	0.32	22.844	650.13	0.30
						22.862	760.28	0.32	22.864	650.51	0.30
						22.882	760.51	0.32	22.884	650.85	0.30
						22.902	760.76	0.32	22.904	651.18	0.30
						22.922	761.00	0.32	22.924	651.51	0.30
						22.942	761.24	0.32	22.944	651.86	0.30
						22.962	761.49	0.32	22.963	652.23	0.30
						22.982	761.73	0.32	22.983	652.57	0.30
						23.002	761.97	0.32	23.003	652.90	0.30
						23.022	762.16	0.32	23.023	653.22	0.30
						23.042	762.35	0.32	23.043	653.56	0.30
						23.062	762.53	0.32	23.063	653.92	0.30
						23.082	762.81	0.32	23.083	654.28	0.30
						23.101	763.02	0.32	23.103	654.63	0.30
						23.121	763.27	0.32	23.123	654.95	0.30
						23.141	763.51	0.32	23.143	655.27	0.30
						23.161	763.74	0.33	23.163	655.61	0.30
						23.181	764.01	0.33	23.183	655.95	0.30
						23.201	764.26	0.33	23.203	656.30	0.30
						23.221	764.47	0.33	23.223	656.64	0.30

						23.241	764.69	0.33	23.243	656.93	0.30
						23.261	764.95	0.33	23.262	657.25	0.30
						23.281	765.20	0.33	23.282	657.59	0.30
						23.301	765.39	0.33	23.302	657.93	0.30
						23.321	765.59	0.33	23.322	658.25	0.30
						23.341	765.82	0.33	23.342	658.59	0.30
						23.361	766.09	0.33	23.362	658.92	0.30
						23.381	766.33	0.33	23.382	659.26	0.30
						23.401	766.58	0.33	23.402	659.58	0.30
						23.421	766.78	0.33	23.422	659.88	0.30
						23.440	766.99	0.33	23.442	660.16	0.30
						23.460	767.24	0.33	23.462	660.48	0.30
						23.480	767.48	0.33	23.482	660.81	0.30
						23.500	767.72	0.33	23.502	661.13	0.30
						23.520	767.93	0.33	23.522	661.44	0.30
						23.540	768.11	0.33	23.542	661.76	0.30
						23.560	768.35	0.33	23.561	662.07	0.30
						23.580	768.58	0.33	23.581	662.40	0.30
						23.600	768.82	0.33	23.601	662.74	0.30
						23.620	769.06	0.33	23.621	663.06	0.30
						23.640	769.24	0.33	23.641	663.36	0.30
						23.660	769.45	0.33	23.661	663.66	0.30
						23.680	769.69	0.33	23.681	663.96	0.30
						23.700	769.92	0.33	23.701	664.27	0.30
						23.720	770.16	0.33	23.721	664.60	0.30
						23.740	770.39	0.33	23.741	664.93	0.30
						23.759	770.62	0.33	23.761	665.21	0.30
						23.779	770.85	0.33	23.781	665.50	0.30
						23.799	771.07	0.33	23.801	665.81	0.30
						23.819	771.30	0.33	23.821	666.12	0.30
						23.839	771.51	0.33	23.841	666.41	0.30
						23.859	771.73	0.33	23.860	666.70	0.30
						23.879	771.95	0.33	23.880	666.98	0.30
						23.899	772.11	0.33	23.900	667.25	0.30
						23.919	772.34	0.33	23.920	667.52	0.30
						23.939	772.60	0.33	23.940	667.78	0.30
						23.959	772.81	0.33	23.960	668.10	0.30
						23.979	773.02	0.33	23.980	668.42	0.30
						23.999	773.23	0.33	24.000	668.75	0.30
						24.019	773.45	0.33	24.020	669.08	0.30
						24.039	773.66	0.33	24.040	669.37	0.30
						24.059	773.87	0.33	24.060	669.70	0.30
						24.078	774.08	0.33	24.080	670.02	0.30
						24.098	774.28	0.33	24.100	670.34	0.30
						24.118	774.49	0.33	24.120	670.65	0.30

						24.138	774.70	0.33	24.140	670.97	0.30
						24.158	774.92	0.33	24.159	671.28	0.31
						24.178	775.17	0.33	24.179	671.58	0.31
						24.198	775.38	0.33	24.199	671.88	0.31
						24.218	775.58	0.33	24.219	672.18	0.31
						24.238	775.78	0.33	24.239	672.50	0.31
						24.258	775.98	0.33	24.259	672.81	0.31
						24.278	776.17	0.33	24.279	673.10	0.31
						24.298	776.40	0.33	24.299	673.40	0.31
						24.318	776.64	0.33	24.319	673.69	0.31
						24.338	776.82	0.33	24.339	673.99	0.31
						24.358	777.00	0.33	24.359	674.30	0.31
						24.378	777.16	0.33	24.379	674.66	0.31
						24.397	777.36	0.33	24.399	674.96	0.31
						24.417	777.57	0.33	24.419	675.25	0.31
						24.437	777.78	0.33	24.439	675.54	0.31
						24.457	777.99	0.33	24.458	675.83	0.31
						24.477	778.21	0.33	24.478	676.15	0.31
						24.497	778.41	0.33	24.498	676.48	0.31
						24.517	778.62	0.33	24.518	676.76	0.31
						24.537	778.88	0.33	24.538	677.04	0.31
						24.557	779.10	0.33	24.558	677.31	0.31
						24.577	779.30	0.33	24.578	677.60	0.31
						24.597	779.51	0.33	24.598	677.93	0.31
						24.617	779.72	0.33	24.618	678.23	0.31
						24.637	779.91	0.33	24.638	678.52	0.31
						24.657	780.11	0.33	24.658	678.84	0.31
						24.677	780.30	0.33	24.678	679.14	0.31
						24.697	780.53	0.33	24.698	679.42	0.31
						24.716	780.78	0.33	24.718	679.69	0.31
						24.736	780.97	0.33	24.738	679.96	0.31
						24.756	781.16	0.33	24.757	680.25	0.31
						24.776	781.40	0.33	24.777	680.58	0.31
						24.796	781.63	0.33	24.797	680.86	0.31
						24.816	781.82	0.33	24.817	681.13	0.31
						24.836	782.01	0.33	24.837	681.44	0.31
						24.856	782.20	0.33	24.857	681.72	0.31
						24.876	782.42	0.33	24.877	681.99	0.31
						24.896	782.65	0.33	24.897	682.30	0.31
						24.916	782.84	0.33	24.917	682.62	0.31
						24.936	783.07	0.33	24.937	682.93	0.31
						24.956	783.28	0.33	24.957	683.19	0.31
						24.976	783.47	0.33	24.977	683.47	0.31
						24.996	783.66	0.33	24.997	683.77	0.31
						25.016	783.89	0.33	25.017	684.03	0.31

						25.035	784.09	0.33	25.037	684.30	0.31
						25.055	784.27	0.33	25.056	684.59	0.31
						25.075	784.51	0.33	25.076	684.89	0.31
						25.095	784.69	0.33	25.096	685.18	0.31
						25.115	784.87	0.33	25.116	685.47	0.31
						25.135	785.05	0.33	25.136	685.76	0.31
						25.155	785.29	0.33	25.156	686.03	0.31
						25.175	785.47	0.33	25.176	686.29	0.31
						25.195	785.65	0.33	25.196	686.55	0.31
						25.215	785.88	0.33	25.216	686.81	0.31
						25.235	786.07	0.33	25.236	687.07	0.31
						25.255	786.27	0.33	25.256	687.34	0.31
						25.275	786.51	0.33	25.276	687.61	0.31
						25.295	786.67	0.33	25.296	687.88	0.31
						25.315	786.87	0.33	25.316	688.14	0.31
						25.335	787.10	0.33	25.336	688.43	0.31
						25.354	787.31	0.33	25.355	688.75	0.31
						25.374	787.49	0.33	25.375	689.01	0.31
						25.394	787.66	0.33	25.395	689.32	0.31
						25.414	787.87	0.33	25.415	689.60	0.31
						25.434	788.05	0.33	25.435	689.86	0.31
						25.454	788.22	0.33	25.455	690.12	0.31
						25.474	788.42	0.33	25.475	690.37	0.31
						25.494	788.63	0.33	25.495	690.68	0.31
						25.514	788.84	0.33	25.515	690.96	0.31
						25.534	789.05	0.33	25.535	691.24	0.31
						25.554	789.24	0.33	25.555	691.54	0.31
						25.574	789.39	0.33	25.575	691.79	0.31
						25.594	789.58	0.33	25.595	692.07	0.31
						25.614	789.79	0.33	25.615	692.38	0.31
						25.634	790.00	0.33	25.635	692.67	0.31
						25.654	790.21	0.33	25.654	692.91	0.31
						25.673	790.41	0.33	25.674	693.15	0.31
						25.693	790.56	0.33	25.694	693.42	0.31
						25.713	790.74	0.33	25.714	693.71	0.31
						25.733	790.95	0.33	25.734	694.01	0.31
						25.753	791.14	0.33	25.754	694.30	0.31
						25.773	791.33	0.33	25.774	694.57	0.31
						25.793	791.53	0.33	25.794	694.81	0.31
						25.813	791.69	0.33	25.814	695.05	0.31
						25.833	791.84	0.33	25.834	695.32	0.31
						25.853	792.07	0.33	25.854	695.60	0.31
						25.873	792.29	0.33	25.874	695.88	0.31
						25.893	792.47	0.33	25.894	696.13	0.31
						25.913	792.62	0.33	25.914	696.38	0.31

						25.933	792.73	0.33	25.934	696.67	0.31
						25.953	792.94	0.33	25.953	696.96	0.31
						25.973	793.11	0.33	25.973	697.21	0.31
						25.992	793.33	0.33	25.993	697.45	0.31
						26.012	793.54	0.33	26.013	697.73	0.31
						26.032	793.72	0.33	26.033	698.01	0.31
						26.052	793.88	0.33	26.053	698.29	0.31
						26.072	794.08	0.33	26.073	698.56	0.31
						26.092	794.27	0.33	26.093	698.79	0.31
						26.112	794.44	0.33	26.113	699.03	0.31
						26.132	794.66	0.33	26.133	699.31	0.31
						26.152	794.87	0.33	26.153	699.57	0.31
						26.172	795.03	0.33	26.173	699.84	0.31
						26.192	795.24	0.33	26.193	700.11	0.31
						26.212	795.45	0.33	26.213	700.33	0.31
						26.232	795.60	0.33	26.233	700.57	0.31
						26.252	795.79	0.33	26.252	700.82	0.31
						26.272	795.99	0.33	26.272	701.08	0.31
						26.292	796.18	0.33	26.292	701.34	0.31
						26.311	796.34	0.33	26.312	701.61	0.31
						26.331	796.55	0.33	26.332	701.87	0.31
						26.351	796.75	0.33	26.352	702.13	0.31
						26.371	796.96	0.33	26.372	702.39	0.31
						26.391	797.16	0.33	26.392	702.65	0.31
						26.411	797.36	0.33	26.412	702.91	0.31
						26.431	797.55	0.33	26.432	703.16	0.31
						26.451	797.69	0.33	26.452	703.41	0.31
						26.471	797.88	0.33	26.472	703.66	0.31
						26.491	798.08	0.33	26.492	703.91	0.31
						26.511	798.24	0.33	26.512	704.15	0.31
						26.531	798.40	0.33	26.532	704.39	0.31
						26.551	798.59	0.33	26.551	704.63	0.31
						26.571	798.80	0.33	26.571	704.88	0.31
						26.591	798.99	0.33	26.591	705.20	0.31
						26.611	799.18	0.33	26.611	705.49	0.31
						26.631	799.38	0.33	26.631	705.65	0.31
						26.650	799.57	0.33	26.651	705.92	0.31
						26.670	799.76	0.33	26.671	706.21	0.31
						26.690	799.90	0.33	26.691	706.45	0.31
						26.710	800.08	0.33	26.711	706.64	0.31
						26.730	800.27	0.33	26.731	706.88	0.31
						26.750	800.46	0.33	26.751	707.13	0.31
						26.770	800.64	0.33	26.771	707.38	0.31
						26.790	800.82	0.33	26.791	707.64	0.31
						26.810	801.00	0.33	26.811	707.94	0.31

						26.830	801.19	0.33	26.831	708.20	0.31
						26.850	801.37	0.33	26.850	708.45	0.31
						26.870	801.56	0.33	26.870	708.69	0.31
						26.890	801.75	0.33	26.890	708.93	0.31
						26.910	801.94	0.33	26.910	709.17	0.31
						26.930	802.13	0.33	26.930	709.40	0.31
						26.950	802.31	0.33	26.950	709.64	0.31
						26.969	802.50	0.33	26.970	709.88	0.31
						26.989	802.68	0.33	26.990	710.14	0.31
						27.009	802.86	0.33	27.010	710.42	0.31
						27.029	803.03	0.33	27.030	710.65	0.31
						27.049	803.21	0.33	27.050	710.87	0.31
						27.069	803.39	0.33	27.070	711.10	0.31
						27.089	803.56	0.33	27.090	711.34	0.31
						27.109	803.74	0.33	27.110	711.57	0.31
						27.129	803.91	0.33	27.130	711.84	0.31
						27.149	804.09	0.33	27.149	712.11	0.31
						27.169	804.25	0.33	27.169	712.33	0.31
						27.189	804.41	0.33	27.189	712.55	0.31
						27.209	804.59	0.33	27.209	712.79	0.31
						27.229	804.81	0.33	27.229	713.06	0.31
						27.249	805.00	0.33	27.249	713.29	0.31
						27.269	805.16	0.33	27.269	713.51	0.31
						27.288	805.33	0.33	27.289	713.73	0.31
						27.308	805.49	0.33	27.309	713.99	0.31
						27.328	805.66	0.33	27.329	714.23	0.31
						27.348	805.82	0.33	27.349	714.44	0.31
						27.368	805.99	0.33	27.369	714.70	0.31
						27.388	806.17	0.33	27.389	714.94	0.31
						27.408	806.38	0.33	27.409	715.16	0.31
						27.428	806.55	0.33	27.429	715.40	0.31
						27.448	806.71	0.33	27.449	715.65	0.31
						27.468	806.87	0.33	27.468	715.86	0.31
						27.488	807.07	0.33	27.488	716.10	0.31
						27.508	807.27	0.33	27.508	716.34	0.31
						27.528	807.42	0.33	27.528	716.54	0.31
						27.548	807.58	0.33	27.548	716.75	0.31
						27.568	807.75	0.33	27.568	716.96	0.31
						27.588	807.95	0.33	27.588	717.17	0.31
						27.607	808.13	0.33	27.608	717.43	0.31
						27.627	808.28	0.33	27.628	717.70	0.31
						27.647	808.43	0.33	27.648	717.93	0.31
						27.667	808.61	0.34	27.668	718.13	0.31
						27.687	808.81	0.34	27.688	718.38	0.31
						27.707	808.97	0.34	27.708	718.59	0.31

						27.727	809.12	0.34	27.728	718.83	0.32
						27.747	809.32	0.34	27.748	719.07	0.32
						27.767	809.50	0.34	27.767	719.29	0.32
						27.787	809.65	0.34	27.787	719.55	0.32
						27.807	809.80	0.34	27.807	719.80	0.32
						27.827	809.96	0.34	27.827	720.01	0.32
						27.847	810.15	0.34	27.847	720.23	0.32
						27.867	810.31	0.34	27.867	720.48	0.32
						27.887	810.46	0.34	27.887	720.67	0.32
						27.907	810.64	0.34	27.907	720.90	0.32
						27.926	810.83	0.34	27.927	721.15	0.32
						27.946	811.03	0.34	27.947	721.40	0.32
						27.966	811.19	0.34	27.967	721.65	0.32
						27.986	811.33	0.34	27.987	721.83	0.32
						28.006	811.50	0.34	28.007	722.06	0.32
						28.026	811.68	0.34	28.027	722.31	0.32
						28.046	811.82	0.34	28.047	722.54	0.32
						28.066	811.96	0.34	28.066	722.72	0.32
						28.086	812.14	0.34	28.086	722.95	0.32
						28.106	812.32	0.34	28.106	723.19	0.32
						28.126	812.49	0.34	28.126	723.43	0.32
						28.146	812.67	0.34	28.146	723.67	0.32
						28.166	812.85	0.34	28.166	723.91	0.32
						28.186	812.99	0.34	28.186	724.15	0.32
						28.206	813.13	0.34	28.206	724.39	0.32
						28.226	813.30	0.34	28.226	724.56	0.32
						28.245	813.48	0.34	28.246	724.77	0.32
						28.265	813.65	0.34	28.266	725.01	0.32
						28.285	813.82	0.34	28.286	725.24	0.32
						28.305	813.99	0.34	28.306	725.47	0.32
						28.325	814.16	0.34	28.326	725.69	0.32
						28.345	814.32	0.34	28.346	725.92	0.32
						28.365	814.48	0.34	28.365	726.15	0.32
						28.385	814.60	0.34	28.385	726.37	0.32
						28.405	814.74	0.34	28.405	726.59	0.32
						28.425	814.91	0.34	28.425	726.82	0.32
						28.445	815.07	0.34	28.445	727.03	0.32
						28.465	815.24	0.34	28.465	727.24	0.32
						28.485	815.40	0.34	28.485	727.46	0.32
						28.505	815.56	0.34	28.505	727.68	0.32
						28.525	815.72	0.34	28.525	727.89	0.32
						28.545	815.88	0.34	28.545	728.11	0.32
						28.564	816.04	0.34	28.565	728.32	0.32
						28.584	816.20	0.34	28.585	728.52	0.32
						28.604	816.35	0.34	28.605	728.68	0.32

						28.624	816.51	0.34	28.625	728.92	0.32
						28.644	816.66	0.34	28.645	729.15	0.32
						28.664	816.81	0.34	28.664	729.34	0.32
						28.684	816.96	0.34	28.684	729.54	0.32
						28.704	817.11	0.34	28.704	729.81	0.32
						28.724	817.27	0.34	28.724	730.02	0.32
						28.744	817.42	0.34	28.744	730.21	0.32
						28.764	817.57	0.34	28.764	730.42	0.32
						28.784	817.76	0.34	28.784	730.67	0.32
						28.804	817.94	0.34	28.804	730.86	0.32
						28.824	818.10	0.34	28.824	731.11	0.32
						28.844	818.24	0.34	28.844	731.32	0.32
						28.864	818.36	0.34	28.864	731.53	0.32
						28.883	818.47	0.34	28.884	731.79	0.32
						28.903	818.66	0.34	28.904	731.97	0.32
						28.923	818.81	0.34	28.924	732.20	0.32
						28.943	818.94	0.34	28.944	732.42	0.32
						28.963	819.07	0.34	28.963	732.60	0.32
						28.983	819.26	0.34	28.983	732.85	0.32
						29.003	819.45	0.34	29.003	733.05	0.32
						29.023	819.60	0.34	29.023	733.26	0.32
						29.043	819.73	0.34	29.043	733.49	0.32
						29.063	819.87	0.34	29.063	733.67	0.32
						29.083	820.07	0.34	29.083	733.90	0.32
						29.103	820.21	0.34	29.103	734.11	0.32
						29.123	820.36	0.34	29.123	734.29	0.32
						29.143	820.53	0.34	29.143	734.53	0.32
						29.163	820.66	0.34	29.163	734.72	0.32
						29.183	820.84	0.34	29.183	734.92	0.32
						29.202	820.98	0.34	29.203	735.16	0.32
						29.222	821.13	0.34	29.223	735.34	0.32
						29.242	821.31	0.34	29.243	735.55	0.32
						29.262	821.49	0.34	29.262	735.78	0.32
						29.282	821.63	0.34	29.282	736.01	0.32
						29.302	821.76	0.34	29.302	736.19	0.32
						29.322	821.94	0.34	29.322	736.39	0.32
						29.342	822.07	0.34	29.342	736.63	0.32
						29.362	822.21	0.34	29.362	736.85	0.32
						29.382	822.39	0.34	29.382	737.04	0.32
						29.402	822.56	0.34	29.402	737.21	0.32
						29.422	822.73	0.34	29.422	737.43	0.32
						29.442	822.88	0.34	29.442	737.66	0.32
						29.462	823.00	0.34	29.462	737.88	0.32
						29.482	823.16	0.34	29.482	738.10	0.32
						29.502	823.32	0.34	29.502	738.28	0.32

						29.521	823.49	0.34	29.522	738.46	0.32
						29.541	823.66	0.34	29.542	738.67	0.32
						29.561	823.80	0.34	29.561	738.89	0.32
						29.581	823.91	0.34	29.581	739.10	0.32
						29.601	824.08	0.34	29.601	739.31	0.32
						29.621	824.24	0.34	29.621	739.48	0.32
						29.641	824.40	0.34	29.641	739.66	0.32
						29.661	824.56	0.34	29.661	739.86	0.32
						29.681	824.72	0.34	29.681	740.07	0.32
						29.701	824.88	0.34	29.701	740.28	0.32
						29.721	824.98	0.34	29.721	740.48	0.32
						29.741	825.11	0.34	29.741	740.68	0.32
						29.761	825.27	0.34	29.761	740.88	0.32
						29.781	825.42	0.34	29.781	741.07	0.32
						29.801	825.58	0.34	29.801	741.26	0.32
						29.821	825.76	0.34	29.821	741.42	0.32
						29.841	825.89	0.34	29.841	741.67	0.32
						29.860	826.02	0.34	29.860	741.89	0.32
						29.880	826.18	0.34	29.880	742.08	0.32
						29.900	826.36	0.34	29.900	742.27	0.32
						29.920	826.48	0.34	29.920	742.46	0.32
						29.940	826.61	0.34	29.940	742.65	0.32
						29.960	826.77	0.34	29.960	742.85	0.32
						29.980	826.94	0.34	29.980	743.04	0.32
						30.000	827.06	0.34	30.000	743.23	0.32

Combined standard uncertainties:

$u(T)$= 0.006 K; $u(p)$= 0.0020 MPa for p < 6 MPa; $u(p)$= 0.024 MPa for 6 MPa ≤ p ≤ 70 MPa

$u(x_{CO_2})$= 0.00025; $u(x_{NO})$= 0.000014; $u(x_{SO_2})$= 0.0000023; $u(x_{CO})$= 0.0000043

Table S1 (continued). $p\rho T$ experimental data for the CO_2 + O_2 + SO_2 + CO (Mix 1) and CO_2 + NO + SO_2 + CO (Mix 2) mixtures. $u(\rho)$: combined standard uncertainty.

**Mix 2: x_{CO_2} = 0.99592; x_{NO} = 0.001410; x_{SO_2} = 0.0009100; x_{CO} = 0.0017002**											
***T*= 373.17±0.03 K**											
***p* (MPa)**	**ρ (kg.m^{-3})**	**$u(\rho)$ (kg.m^{-3})**									
0.105	1.42	0.22									
0.125	1.73	0.23									
0.145	2.03	0.22									
0.165	2.33	0.22									
0.185	2.64	0.22									
0.205	2.92	0.22									
0.225	3.21	0.22									
0.245	3.49	0.22									
0.265	3.78	0.22									
0.285	4.04	0.22									
0.305	4.34	0.22									
0.325	4.64	0.22									
0.345	4.95	0.22									
0.365	5.25	0.22									
0.385	5.52	0.22									
0.405	5.84	0.22									
0.425	6.09	0.22									
0.444	6.37	0.22									
0.464	6.67	0.22									
0.484	6.95	0.22									
0.504	7.25	0.22									
0.524	7.56	0.22									
0.544	7.87	0.22									
0.564	8.17	0.22									
0.584	8.45	0.22									
0.604	8.70	0.22									
0.624	9.01	0.22									
0.644	9.31	0.22									
0.664	9.57	0.22									
0.684	9.88	0.22									
0.704	10.20	0.22									
0.724	10.46	0.22									
0.744	10.73	0.22									
0.764	11.06	0.22									
0.784	11.33	0.22									

0.803	11.63	0.22									
0.823	11.93	0.22									
0.843	12.23	0.22									
0.863	12.56	0.22									
0.883	12.82	0.22									
0.903	13.10	0.22									
0.923	13.38	0.22									
0.943	13.71	0.22									
0.963	14.01	0.22									
0.983	14.31	0.22									
1.003	14.64	0.22									
1.023	14.91	0.22									
1.043	15.19	0.22									
1.063	15.53	0.22									
1.083	15.84	0.22									
1.103	16.13	0.22									
1.123	16.42	0.22									
1.143	16.68	0.22									
1.162	17.01	0.22									
1.182	17.31	0.22									
1.202	17.63	0.22									
1.222	17.93	0.22									
1.242	18.22	0.22									
1.262	18.52	0.22									
1.282	18.76	0.22									
1.302	19.11	0.22									
1.322	19.38	0.22									
1.342	19.70	0.22									
1.362	20.02	0.22									
1.382	20.32	0.22									
1.402	20.64	0.22									
1.422	20.91	0.22									
1.442	21.24	0.22									
1.462	21.55	0.22									
1.482	21.84	0.22									
1.501	22.14	0.22									
1.521	22.46	0.22									
1.541	22.78	0.22									
1.561	23.05	0.22									
1.581	23.33	0.22									
1.601	23.67	0.22									
1.621	24.00	0.22									
1.641	24.28	0.22									
1.661	24.58	0.22									
1.681	24.94	0.22									

1.701	25.24	0.22									
1.721	25.53	0.22									
1.741	25.82	0.22									
1.761	26.11	0.22									
1.781	26.43	0.22									
1.801	26.78	0.22									
1.821	27.11	0.22									
1.841	27.41	0.22									
1.860	27.70	0.22									
1.880	27.99	0.22									
1.900	28.29	0.22									
1.920	28.61	0.22									
1.940	28.94	0.22									
1.960	29.20	0.22									
1.980	29.53	0.22									
2.000	29.88	0.22									
2.020	30.18	0.22									
2.040	30.46	0.22									
2.060	30.77	0.22									
2.080	31.10	0.22									
2.100	31.43	0.22									
2.120	31.76	0.22									
2.140	32.07	0.22									
2.160	32.37	0.22									
2.180	32.68	0.22									
2.199	32.98	0.22									
2.219	33.34	0.22									
2.239	33.63	0.22									
2.259	33.93	0.22									
2.279	34.25	0.22									
2.299	34.57	0.22									
2.319	34.88	0.22									
2.339	35.19	0.22									
2.359	35.52	0.22									
2.379	35.87	0.22									
2.399	36.16	0.22									
2.419	36.50	0.22									
2.439	36.80	0.22									
2.459	37.09	0.22									
2.479	37.41	0.22									
2.499	37.73	0.22									
2.519	38.05	0.22									
2.539	38.37	0.22									
2.558	38.71	0.22									
2.578	39.05	0.22									

2.598	39.35	0.22									
2.618	39.64	0.22									
2.638	39.97	0.22									
2.658	40.31	0.22									
2.678	40.62	0.22									
2.698	40.94	0.22									
2.718	41.26	0.22									
2.738	41.57	0.22									
2.758	41.90	0.22									
2.778	42.25	0.22									
2.798	42.55	0.22									
2.818	42.89	0.22									
2.838	43.21	0.22									
2.858	43.51	0.22									
2.878	43.84	0.22									
2.897	44.17	0.22									
2.917	44.49	0.22									
2.937	44.81	0.22									
2.957	45.19	0.22									
2.977	45.51	0.22									
2.997	45.82	0.22									
3.017	46.13	0.22									
3.037	46.45	0.22									
3.057	46.79	0.22									
3.077	47.11	0.22									
3.097	47.44	0.22									
3.117	47.77	0.22									
3.137	48.09	0.22									
3.157	48.41	0.22									
3.177	48.72	0.22									
3.197	49.08	0.22									
3.217	49.41	0.22									
3.237	49.70	0.22									
3.256	50.03	0.22									
3.276	50.41	0.22									
3.296	50.74	0.22									
3.316	51.05	0.22									
3.336	51.38	0.22									
3.356	51.75	0.22									
3.376	52.05	0.22									
3.396	52.40	0.22									
3.416	52.76	0.22									
3.436	53.10	0.22									
3.456	53.39	0.22									
3.476	53.72	0.22									

3.496	54.05	0.22									
3.516	54.37	0.22									
3.536	54.69	0.22									
3.556	55.06	0.22									
3.576	55.39	0.22									
3.595	55.73	0.22									
3.615	56.09	0.22									
3.635	56.39	0.22									
3.655	56.74	0.22									
3.675	57.10	0.22									
3.695	57.45	0.22									
3.715	57.79	0.22									
3.735	58.11	0.22									
3.755	58.46	0.22									
3.775	58.81	0.22									
3.795	59.13	0.22									
3.815	59.49	0.22									
3.835	59.79	0.22									
3.855	60.13	0.22									
3.875	60.49	0.22									
3.895	60.84	0.22									
3.915	61.19	0.22									
3.935	61.54	0.22									
3.954	61.89	0.22									
3.974	62.23	0.22									
3.994	62.56	0.22									
4.014	62.90	0.22									
4.034	63.24	0.22									
4.054	63.60	0.22									
4.074	63.93	0.22									
4.094	64.29	0.22									
4.114	64.65	0.22									
4.134	64.97	0.22									
4.154	65.34	0.22									
4.174	65.65	0.22									
4.194	65.99	0.22									
4.214	66.35	0.22									
4.234	66.70	0.22									
4.254	67.06	0.22									
4.274	67.42	0.22									
4.293	67.77	0.22									
4.313	68.11	0.22									
4.333	68.47	0.22									
4.353	68.80	0.22									
4.373	69.17	0.22									

4.393	69.62	0.22									
4.413	69.92	0.22									
4.433	70.25	0.22									
4.453	70.63	0.22									
4.473	71.06	0.22									
4.493	71.40	0.22									
4.513	71.71	0.22									
4.533	72.10	0.22									
4.553	72.47	0.22									
4.573	72.80	0.22									
4.593	73.15	0.22									
4.613	73.51	0.22									
4.633	73.89	0.22									
4.652	74.25	0.22									
4.672	74.62	0.22									
4.692	74.98	0.22									
4.712	75.34	0.22									
4.732	75.69	0.22									
4.752	76.04	0.22									
4.772	76.38	0.22									
4.792	76.74	0.22									
4.812	77.13	0.22									
4.832	77.49	0.22									
4.852	77.85	0.22									
4.872	78.25	0.22									
4.892	78.58	0.22									
4.912	78.95	0.22									
4.932	79.33	0.22									
4.952	79.71	0.22									
4.972	80.09	0.22									
4.991	80.46	0.22									
5.011	80.83	0.22									
5.031	81.19	0.22									
5.051	81.52	0.22									
5.071	81.85	0.22									
5.091	82.22	0.22									
5.111	82.58	0.22									
5.131	82.98	0.22									
5.151	83.39	0.22									
5.171	83.75	0.22									
5.191	84.11	0.22									
5.211	84.46	0.22									
5.231	84.83	0.22									
5.251	85.24	0.22									
5.271	85.59	0.22									

5.291	85.98	0.22									
5.311	86.36	0.22									
5.331	86.70	0.22									
5.350	87.09	0.22									
5.370	87.49	0.22									
5.390	87.87	0.22									
5.410	88.21	0.22									
5.430	88.57	0.22									
5.450	88.95	0.22									
5.470	89.33	0.22									
5.490	89.70	0.22									
5.510	90.08	0.22									
5.530	90.45	0.22									
5.550	90.87	0.22									
5.570	91.26	0.22									
5.590	91.64	0.22									
5.610	91.99	0.22									
5.630	92.37	0.22									
5.650	92.78	0.22									
5.670	93.13	0.22									
5.690	93.50	0.22									
5.709	93.91	0.22									
5.729	94.31	0.22									
5.749	94.69	0.22									
5.769	95.02	0.22									
5.789	95.39	0.22									
5.809	95.79	0.22									
5.829	96.19	0.22									
5.849	96.53	0.22									
5.869	96.95	0.22									
5.889	97.32	0.22									
5.909	97.69	0.22									
5.929	98.09	0.22									
5.949	98.51	0.22									
5.969	98.92	0.23									
5.989	99.32	0.23									
6.009	99.72	0.23									
6.029	100.11	0.23									
6.048	100.50	0.23									
6.068	100.88	0.23									
6.088	101.27	0.23									
6.108	101.66	0.23									
6.128	102.05	0.23									
6.148	102.43	0.23									
6.168	102.85	0.23									

6.188	103.25	0.23									
6.208	103.63	0.23									
6.228	103.98	0.23									
6.248	104.35	0.23									
6.268	104.73	0.23									
6.288	105.13	0.23									
6.308	105.56	0.23									
6.328	105.94	0.23									
6.348	106.32	0.23									
6.368	106.72	0.23									
6.388	107.15	0.23									
6.407	107.51	0.23									
6.427	107.92	0.23									
6.447	108.34	0.23									
6.467	108.75	0.23									
6.487	109.12	0.23									
6.507	109.54	0.23									
6.527	109.95	0.23									
6.547	110.35	0.23									
6.567	110.77	0.23									
6.587	111.17	0.23									
6.607	111.58	0.23									
6.627	111.99	0.23									
6.647	112.40	0.23									
6.667	112.79	0.23									
6.687	113.19	0.23									
6.707	113.58	0.23									
6.727	113.97	0.23									
6.746	114.40	0.23									
6.766	114.83	0.23									
6.786	115.22	0.23									
6.806	115.65	0.23									
6.826	116.06	0.23									
6.846	116.45	0.23									
6.866	116.88	0.23									
6.886	117.31	0.23									
6.906	117.73	0.23									
6.926	118.09	0.23									
6.946	118.49	0.23									
6.966	118.90	0.23									
6.986	119.29	0.23									
7.006	119.73	0.23									
7.026	120.11	0.23									
7.046	120.52	0.23									
7.066	120.96	0.23									

7.086	121.39	0.23									
7.105	121.82	0.23									
7.125	122.26	0.23									
7.145	122.66	0.23									
7.165	123.04	0.23									
7.185	123.47	0.23									
7.205	123.89	0.23									
7.225	124.31	0.23									
7.245	124.72	0.23									
7.265	125.14	0.23									
7.285	125.55	0.23									
7.305	126.00	0.23									
7.325	126.44	0.23									
7.345	126.84	0.23									
7.365	127.26	0.23									
7.385	127.66	0.23									
7.405	128.08	0.23									
7.425	128.54	0.23									
7.444	128.94	0.23									
7.464	129.37	0.23									
7.484	129.81	0.23									
7.504	130.27	0.23									
7.524	130.67	0.23									
7.544	131.07	0.23									
7.564	131.49	0.23									
7.584	131.93	0.23									
7.604	132.37	0.23									
7.624	132.81	0.23									
7.644	133.26	0.23									
7.664	133.70	0.23									
7.684	134.13	0.23									
7.704	134.56	0.23									
7.724	134.98	0.23									
7.744	135.41	0.23									
7.764	135.89	0.23									
7.784	136.33	0.23									
7.803	136.74	0.23									
7.823	137.16	0.23									
7.843	137.61	0.23									
7.863	138.06	0.23									
7.883	138.48	0.23									
7.903	138.92	0.23									
7.923	139.37	0.23									
7.943	139.83	0.23									
7.963	140.25	0.23									

7.983	140.64	0.23									
8.003	141.10	0.23									
8.023	141.54	0.23									
8.043	141.97	0.23									
8.063	142.40	0.23									
8.083	142.83	0.23									
8.103	143.29	0.23									
8.123	143.71	0.23									
8.142	144.13	0.23									
8.162	144.62	0.23									
8.182	145.06	0.23									
8.202	145.48	0.23									
8.222	145.91	0.23									
8.242	146.38	0.23									
8.262	146.81	0.23									
8.282	147.25	0.23									
8.302	147.73	0.23									
8.322	148.20	0.23									
8.342	148.63	0.23									
8.362	149.06	0.23									
8.382	149.52	0.23									
8.402	149.97	0.23									
8.422	150.43	0.23									
8.442	150.90	0.23									
8.462	151.36	0.23									
8.482	151.82	0.23									
8.501	152.28	0.23									
8.521	152.73	0.23									
8.541	153.17	0.23									
8.561	153.62	0.23									
8.581	154.07	0.23									
8.601	154.52	0.23									
8.621	155.02	0.23									
8.641	155.48	0.23									
8.661	155.93	0.23									
8.681	156.37	0.23									
8.701	156.86	0.23									
8.721	157.32	0.23									
8.741	157.74	0.23									
8.761	158.23	0.23									
8.781	158.72	0.23									
8.801	159.20	0.23									
8.821	159.62	0.23									
8.840	160.07	0.23									
8.860	160.56	0.23									

8.880	161.02	0.23									
8.900	161.49	0.23									
8.920	161.97	0.23									
8.940	162.43	0.23									
8.960	162.90	0.23									
8.980	163.37	0.23									
9.000	163.83	0.23									
9.020	164.32	0.23									
9.040	164.83	0.23									
9.060	165.29	0.23									
9.080	165.75	0.23									
9.100	166.20	0.23									
9.120	166.69	0.23									
9.140	167.18	0.23									
9.160	167.63	0.23									
9.180	168.12	0.23									
9.199	168.62	0.23									
9.219	169.09	0.23									
9.239	169.55	0.23									
9.259	170.01	0.23									
9.279	170.50	0.23									
9.299	170.99	0.23									
9.319	171.48	0.23									
9.339	171.89	0.23									
9.359	172.31	0.23									
9.379	172.80	0.23									
9.399	173.28	0.23									
9.419	173.77	0.23									
9.439	174.25	0.23									
9.459	174.73	0.23									
9.479	175.20	0.23									
9.499	175.67	0.23									
9.519	176.16	0.23									
9.538	176.64	0.23									
9.558	177.12	0.23									
9.578	177.59	0.23									
9.598	178.08	0.23									
9.618	178.58	0.23									
9.638	179.09	0.23									
9.658	179.59	0.23									
9.678	180.10	0.23									
9.698	180.60	0.23									
9.718	181.12	0.23									
9.738	181.64	0.23									
9.758	182.16	0.23									

9.778	182.68	0.23									
9.798	183.20	0.23									
9.818	183.70	0.23									
9.838	184.20	0.23									
9.858	184.70	0.23									
9.878	185.20	0.23									
9.897	185.70	0.23									
9.917	186.22	0.23									
9.937	186.75	0.23									
9.957	187.27	0.23									
9.977	187.80	0.23									
9.997	188.32	0.23									
10.017	188.82	0.23									
10.037	189.31	0.23									
10.057	189.80	0.23									
10.077	190.29	0.23									
10.097	190.78	0.23									
10.117	191.29	0.23									
10.137	191.81	0.23									
10.157	192.32	0.23									
10.177	192.83	0.23									
10.197	193.34	0.23									
10.217	193.85	0.23									
10.237	194.36	0.23									
10.256	194.87	0.23									
10.276	195.37	0.23									
10.296	195.87	0.23									
10.316	196.38	0.23									
10.336	196.89	0.23									
10.356	197.41	0.23									
10.376	197.93	0.23									
10.396	198.45	0.23									
10.416	198.97	0.23									
10.436	199.47	0.23									
10.456	199.96	0.23									
10.476	200.46	0.23									
10.496	201.00	0.23									
10.516	201.54	0.23									
10.536	202.06	0.23									
10.556	202.57	0.23									
10.576	203.07	0.23									
10.595	203.60	0.23									
10.615	204.13	0.23									
10.635	204.65	0.23									
10.655	205.17	0.23									

10.675	205.68	0.23									
10.695	206.20	0.23									
10.715	206.73	0.23									
10.735	207.25	0.23									
10.755	207.77	0.23									
10.775	208.29	0.23									
10.795	208.80	0.23									
10.815	209.29	0.23									
10.835	209.81	0.23									
10.855	210.35	0.23									
10.875	210.88	0.23									
10.895	211.39	0.23									
10.915	211.90	0.23									
10.935	212.41	0.23									
10.954	212.92	0.23									
10.974	213.46	0.23									
10.994	214.01	0.23									
11.014	214.52	0.23									
11.034	215.04	0.23									
11.054	215.60	0.23									
11.074	216.17	0.23									
11.094	216.67	0.23									
11.114	217.17	0.23									
11.134	217.75	0.23									
11.154	218.32	0.23									
11.174	218.82	0.23									
11.194	219.32	0.23									
11.214	219.85	0.23									
11.234	220.38	0.23									
11.254	220.92	0.23									
11.274	221.45	0.23									
11.293	221.98	0.23									
11.313	222.55	0.23									
11.333	223.13	0.23									
11.353	223.68	0.23									
11.373	224.21	0.23									
11.393	224.71	0.23									
11.413	225.28	0.23									
11.433	225.86	0.23									
11.453	226.40	0.23									
11.473	226.94	0.23									
11.493	227.48	0.23									
11.513	228.02	0.23									
11.533	228.54	0.23									
11.553	229.09	0.23									

11.573	229.65	0.23									
11.593	230.20	0.23									
11.613	230.76	0.23									
11.633	231.31	0.23									
11.652	231.87	0.23									
11.672	232.43	0.23									
11.692	232.96	0.23									
11.712	233.51	0.23									
11.732	234.07	0.24									
11.752	234.64	0.24									
11.772	235.22	0.24									
11.792	235.79	0.24									
11.812	236.36	0.24									
11.832	236.91	0.24									
11.852	237.46	0.24									
11.872	238.00	0.24									
11.892	238.56	0.24									
11.912	239.12	0.24									
11.932	239.72	0.24									
11.952	240.30	0.24									
11.972	240.85	0.24									
11.991	241.39	0.24									
12.011	241.93	0.24									
12.031	242.49	0.24									
12.051	243.07	0.24									
12.071	243.68	0.24									
12.091	244.25	0.24									
12.111	244.80	0.24									
12.131	245.36	0.24									
12.151	245.92	0.24									
12.171	246.51	0.24									
12.191	247.09	0.24									
12.211	247.66	0.24									
12.231	248.24	0.24									
12.251	248.83	0.24									
12.271	249.42	0.24									
12.291	250.01	0.24									
12.311	250.58	0.24									
12.331	251.16	0.24									
12.350	251.75	0.24									
12.370	252.34	0.24									
12.390	252.92	0.24									
12.410	253.50	0.24									
12.430	254.08	0.24									
12.450	254.66	0.24									

12.470	255.23	0.24									
12.490	255.83	0.24									
12.510	256.43	0.24									
12.530	257.03	0.24									
12.550	257.62	0.24									
12.570	258.21	0.24									
12.590	258.80	0.24									
12.610	259.37	0.24									
12.630	259.93	0.24									
12.650	260.52	0.24									
12.670	261.12	0.24									
12.689	261.72	0.24									
12.709	262.31	0.24									
12.729	262.89	0.24									
12.749	263.48	0.24									
12.769	264.06	0.24									
12.789	264.65	0.24									
12.809	265.24	0.24									
12.829	265.85	0.24									
12.849	266.46	0.24									
12.869	267.05	0.24									
12.889	267.64	0.24									
12.909	268.23	0.24									
12.929	268.83	0.24									
12.949	269.42	0.24									
12.969	270.01	0.24									
12.989	270.61	0.24									
13.009	271.20	0.24									
13.029	271.80	0.24									
13.048	272.40	0.24									
13.068	273.00	0.24									
13.088	273.60	0.24									
13.108	274.20	0.24									
13.128	274.79	0.24									
13.148	275.39	0.24									
13.168	275.99	0.24									
13.188	276.59	0.24									
13.208	277.18	0.24									
13.228	277.74	0.24									
13.248	278.30	0.24									
13.268	278.86	0.24									
13.288	279.42	0.24									
13.308	279.98	0.24									
13.328	280.58	0.24									
13.348	281.19	0.24									

13.368	281.80	0.24									
13.387	282.41	0.24									
13.407	283.02	0.24									
13.427	283.65	0.24									
13.447	284.27	0.24									
13.467	284.90	0.24									
13.487	285.52	0.24									
13.507	286.15	0.24									
13.527	286.72	0.24									
13.547	287.30	0.24									
13.567	287.87	0.24									
13.587	288.44	0.24									
13.607	289.02	0.24									
13.627	289.58	0.24									
13.647	290.15	0.24									
13.667	290.72	0.24									
13.687	291.29	0.24									
13.707	291.85	0.24									
13.727	292.46	0.24									
13.746	293.07	0.24									
13.766	293.68	0.24									
13.786	294.28	0.24									
13.806	294.89	0.24									
13.826	295.49	0.24									
13.846	296.08	0.24									
13.866	296.68	0.24									
13.886	297.28	0.24									
13.906	297.87	0.24									
13.926	298.46	0.24									
13.946	299.05	0.24									
13.966	299.64	0.24									
13.986	300.23	0.24									
14.006	300.82	0.24									
14.026	301.43	0.24									
14.046	302.04	0.24									
14.066	302.66	0.24									
14.086	303.27	0.24									
14.105	303.88	0.24									
14.125	304.47	0.24									
14.145	305.05	0.24									
14.165	305.64	0.24									
14.185	306.23	0.24									
14.205	306.82	0.24									
14.225	307.45	0.24									
14.245	308.08	0.24									

14.265	308.67	0.24									
14.285	309.31	0.24									
14.305	309.93	0.24									
14.325	310.52	0.24									
14.345	311.14	0.24									
14.365	311.78	0.24									
14.385	312.42	0.24									
14.405	313.04	0.24									
14.425	313.68	0.24									
14.444	314.31	0.24									
14.464	314.91	0.24									
14.484	315.53	0.24									
14.504	316.18	0.24									
14.524	316.84	0.24									
14.544	317.50	0.24									
14.564	318.07	0.24									
14.584	318.65	0.24									
14.604	319.19	0.24									
14.624	319.73	0.24									
14.644	320.29	0.24									
14.664	320.86	0.24									
14.684	321.46	0.24									
14.704	322.04	0.24									
14.724	322.74	0.25									
14.744	323.31	0.25									
14.764	324.00	0.25									
14.784	324.65	0.25									
14.803	325.28	0.25									
14.823	325.95	0.25									
14.843	326.54	0.25									
14.863	327.18	0.25									
14.883	327.83	0.25									
14.903	328.45	0.25									
14.923	329.09	0.25									
14.943	329.71	0.25									
14.963	330.32	0.25									
14.983	330.97	0.25									
15.003	331.59	0.25									
15.023	332.22	0.25									
15.043	332.84	0.25									
15.063	333.46	0.25									
15.083	334.08	0.25									
15.103	334.70	0.25									
15.123	335.32	0.25									
15.142	335.94	0.25									

15.162	336.56	0.25									
15.182	337.18	0.25									
15.202	337.80	0.25									
15.222	338.42	0.25									
15.242	339.04	0.25									
15.262	339.66	0.25									
15.282	340.28	0.25									
15.302	340.90	0.25									
15.322	341.52	0.25									
15.342	342.14	0.25									
15.362	342.76	0.25									
15.382	343.38	0.25									
15.402	344.00	0.25									
15.422	344.61	0.25									
15.442	345.21	0.25									
15.462	345.82	0.25									
15.482	346.43	0.25									
15.501	347.04	0.25									
15.521	347.66	0.25									
15.541	348.33	0.25									
15.561	349.01	0.25									
15.581	349.61	0.25									
15.601	350.22	0.25									
15.621	350.83	0.25									
15.641	351.43	0.25									
15.661	352.04	0.25									
15.681	352.64	0.25									
15.701	353.25	0.25									
15.721	353.84	0.25									
15.741	354.43	0.25									
15.761	355.02	0.25									
15.781	355.61	0.25									
15.801	356.20	0.25									
15.821	356.82	0.25									
15.840	357.44	0.25									
15.860	358.06	0.25									
15.880	358.68	0.25									
15.900	359.30	0.25									
15.920	359.92	0.25									
15.940	360.54	0.25									
15.960	361.16	0.25									
15.980	361.78	0.25									
16.000	362.40	0.25									
16.020	362.96	0.25									
16.040	363.52	0.25									

16.060	364.08	0.25									
16.080	364.64	0.25									
16.100	365.20	0.25									
16.120	365.86	0.25									
16.140	366.52	0.25									
16.160	367.18	0.25									
16.180	367.84	0.25									
16.199	368.50	0.25									
16.219	369.14	0.25									
16.239	369.78	0.25									
16.259	370.42	0.25									
16.279	371.06	0.25									
16.299	371.70	0.25									
16.319	372.36	0.25									
16.339	373.02	0.25									
16.359	373.68	0.25									
16.379	374.34	0.25									
16.399	375.00	0.26									
16.419	375.56	0.26									
16.439	376.12	0.26									
16.459	376.68	0.26									
16.479	377.24	0.26									
16.499	377.80	0.26									
16.519	378.38	0.26									
16.538	378.96	0.26									
16.558	379.54	0.26									
16.578	380.12	0.26									
16.598	380.70	0.26									
16.618	381.32	0.26									
16.638	381.94	0.26									
16.658	382.56	0.26									
16.678	383.18	0.26									
16.698	383.80	0.26									
16.718	384.36	0.26									
16.738	384.92	0.26									
16.758	385.48	0.26									
16.778	386.04	0.26									
16.798	386.60	0.26									
16.818	387.28	0.26									
16.838	387.96	0.26									
16.858	388.64	0.26									
16.878	389.32	0.26									
16.897	390.00	0.26									
16.917	390.61	0.26									
16.937	391.23	0.26									

16.957	391.84	0.26									
16.977	392.39	0.26									
16.997	392.93	0.26									
17.017	393.48	0.26									
17.037	394.11	0.26									
17.057	394.74	0.26									
17.077	395.37	0.26									
17.097	396.00	0.26									
17.117	396.60	0.26									
17.137	397.20	0.26									
17.157	397.80	0.26									
17.177	398.40	0.26									
17.197	399.00	0.26									
17.217	399.54	0.26									
17.236	400.08	0.26									
17.256	400.62	0.26									
17.276	401.16	0.26									
17.296	401.70	0.26									
17.316	402.26	0.26									
17.336	402.82	0.26									
17.356	403.38	0.26									
17.376	403.94	0.26									
17.396	404.50	0.26									
17.416	405.13	0.26									
17.436	405.76	0.26									
17.456	406.39	0.26									
17.476	407.02	0.26									
17.496	407.65	0.26									
17.516	408.23	0.26									
17.536	408.81	0.26									
17.556	409.39	0.26									
17.576	409.97	0.26									
17.595	410.55	0.26									
17.615	411.06	0.26									
17.635	411.57	0.26									
17.655	412.08	0.26									
17.675	412.59	0.26									
17.695	413.10	0.26									
17.715	413.69	0.26									
17.735	414.28	0.26									
17.755	414.87	0.26									
17.775	415.46	0.26									
17.795	416.05	0.26									
17.815	416.64	0.26									
17.835	417.23	0.26									

17.855	417.82	0.26									
17.875	418.41	0.26									
17.895	419.00	0.26									
17.915	419.60	0.26									
17.934	420.20	0.26									
17.954	420.80	0.26									
17.974	421.40	0.26									
17.994	422.00	0.26									
18.014	422.54	0.26									
18.034	423.08	0.26									
18.054	423.62	0.26									
18.074	424.16	0.26									
18.094	424.70	0.26									
18.114	425.30	0.26									
18.134	425.90	0.26									
18.154	426.50	0.26									
18.174	427.10	0.26									
18.194	427.70	0.26									
18.214	428.31	0.26									
18.234	428.92	0.26									
18.254	429.53	0.26									
18.274	430.14	0.26									
18.293	430.75	0.26									
18.313	431.29	0.26									
18.333	431.83	0.26									
18.353	432.37	0.26									
18.373	432.91	0.26									
18.393	433.45	0.26									
18.413	434.06	0.26									
18.433	434.67	0.26									
18.453	435.28	0.26									
18.473	435.89	0.26									
18.493	436.50	0.26									
18.513	437.00	0.26									
18.533	437.50	0.26									
18.553	438.00	0.26									
18.573	438.50	0.26									
18.593	439.00	0.26									
18.613	439.60	0.26									
18.633	440.20	0.26									
18.652	440.80	0.26									
18.672	441.40	0.26									
18.692	442.00	0.26									
18.712	442.50	0.26									
18.732	443.00	0.26									

18.752	443.50	0.26									
18.772	444.00	0.26									
18.792	444.50	0.26									
18.812	445.10	0.26									
18.832	445.70	0.26									
18.852	446.30	0.26									
18.872	446.90	0.26									
18.892	447.50	0.26									
18.912	448.06	0.26									
18.932	448.62	0.26									
18.952	449.18	0.26									
18.972	449.74	0.26									
18.991	450.30	0.26									
19.011	450.84	0.26									
19.031	451.38	0.26									
19.051	451.92	0.26									
19.071	452.46	0.26									
19.091	453.00	0.26									
19.111	453.58	0.26									
19.131	454.16	0.26									
19.151	454.74	0.26									
19.171	455.31	0.26									
19.191	455.89	0.26									
19.211	456.47	0.26									
19.231	457.05	0.26									
19.251	457.57	0.26									
19.271	458.10	0.26									
19.291	458.62	0.26									
19.311	459.14	0.26									
19.331	459.66	0.26									
19.350	460.18	0.26									
19.370	460.71	0.27									
19.390	461.23	0.27									
19.410	461.75	0.27									
19.430	462.27	0.27									
19.450	462.80	0.27									
19.470	463.32	0.27									
19.490	463.84	0.27									
19.510	464.36	0.27									
19.530	464.88	0.27									
19.550	465.41	0.27									
19.570	465.93	0.27									
19.590	466.45	0.27									
19.610	466.97	0.27									
19.630	467.50	0.27									

19.650	468.02	0.27									
19.670	468.54	0.27									
19.689	469.06	0.27									
19.709	469.58	0.27									
19.729	470.11	0.27									
19.749	470.63	0.27									
19.769	471.15	0.27									
19.789	471.67	0.27									
19.809	472.20	0.27									
19.829	472.72	0.27									
19.849	473.24	0.27									
19.869	473.76	0.27									
19.889	474.29	0.27									
19.909	474.81	0.27									
19.929	475.33	0.27									
19.949	475.85	0.27									
19.969	476.37	0.27									
19.989	476.90	0.27									
20.009	477.42	0.27									
20.029	477.94	0.27									
20.048	478.46	0.27									
20.068	478.99	0.27									
20.088	479.51	0.27									
20.108	480.03	0.27									
20.128	480.55	0.27									
20.148	481.08	0.27									
20.168	481.60	0.27									
20.188	482.12	0.27									
20.208	482.64	0.27									
20.228	483.16	0.27									
20.248	483.69	0.27									
20.268	484.21	0.27									
20.288	484.73	0.27									
20.308	485.25	0.27									
20.328	485.78	0.27									
20.348	486.30	0.27									
20.368	486.82	0.27									
20.387	487.34	0.27									
20.407	487.86	0.27									
20.427	488.39	0.27									
20.447	488.91	0.27									
20.467	489.43	0.27									
20.487	489.95	0.27									
20.507	490.48	0.27									
20.527	491.00	0.27									

20.547	491.52	0.27									
20.567	492.04	0.27									
20.587	492.56	0.27									
20.607	493.09	0.27									
20.627	493.61	0.27									
20.647	494.13	0.27									
20.667	494.65	0.27									
20.687	495.18	0.27									
20.707	495.70	0.27									
20.727	496.22	0.27									
20.746	496.74	0.27									
20.766	497.26	0.27									
20.786	497.79	0.27									
20.806	498.31	0.27									
20.826	498.83	0.27									
20.846	499.35	0.27									
20.866	499.88	0.27									
20.886	500.40	0.27									
20.906	500.92	0.27									
20.926	501.44	0.27									
20.946	501.97	0.27									
20.966	502.49	0.27									
20.986	503.01	0.27									
21.006	503.53	0.27									
21.026	504.05	0.27									
21.046	504.58	0.27									
21.066	505.10	0.27									
21.085	505.62	0.27									
21.105	506.14	0.27									
21.125	506.67	0.27									
21.145	507.19	0.27									
21.165	507.71	0.27									
21.185	508.23	0.27									
21.205	508.75	0.27									
21.225	509.28	0.27									
21.245	509.75	0.27									
21.265	510.27	0.27									
21.285	510.78	0.27									
21.305	511.35	0.27									
21.325	511.86	0.27									
21.345	512.37	0.27									
21.365	512.82	0.27									
21.385	513.27	0.27									
21.405	513.73	0.27									
21.425	514.25	0.27									

21.444	514.74	0.27									
21.464	515.21	0.27									
21.484	515.65	0.27									
21.504	516.12	0.27									
21.524	516.61	0.28									
21.544	517.07	0.28									
21.564	517.52	0.28									
21.584	517.95	0.28									
21.604	518.39	0.28									
21.624	518.86	0.28									
21.644	519.32	0.28									
21.664	519.76	0.28									
21.684	520.21	0.28									
21.704	520.66	0.28									
21.724	521.07	0.28									
21.744	521.43	0.28									
21.764	521.79	0.28									
21.783	522.21	0.28									
21.803	522.68	0.28									
21.823	523.13	0.28									
21.843	523.58	0.28									
21.863	524.08	0.28									
21.883	524.53	0.28									
21.903	524.96	0.28									
21.923	525.44	0.28									
21.943	525.91	0.28									
21.963	526.37	0.28									
21.983	526.80	0.28									
22.003	527.25	0.28									
22.023	527.69	0.28									
22.043	528.10	0.28									
22.063	528.57	0.28									
22.083	529.05	0.28									
22.103	529.57	0.28									
22.123	530.08	0.28									
22.142	530.53	0.28									
22.162	530.96	0.28									
22.182	531.39	0.28									
22.202	531.85	0.28									
22.222	532.27	0.28									
22.242	532.71	0.28									
22.262	533.18	0.28									
22.282	533.59	0.28									
22.302	534.00	0.28									
22.322	534.45	0.28									

22.342	534.90	0.28									
22.362	535.36	0.28									
22.382	535.77	0.28									
22.402	536.20	0.28									
22.422	536.65	0.28									
22.442	537.07	0.28									
22.462	537.49	0.28									
22.481	537.90	0.28									
22.501	538.32	0.28									
22.521	538.75	0.28									
22.541	539.19	0.28									
22.561	539.55	0.28									
22.581	539.89	0.28									
22.601	540.20	0.28									
22.621	540.55	0.28									
22.641	540.98	0.28									
22.661	541.37	0.28									
22.681	541.82	0.28									
22.701	542.28	0.28									
22.721	542.75	0.28									
22.741	543.11	0.28									
22.761	543.55	0.28									
22.781	543.99	0.28									
22.801	544.40	0.28									
22.821	544.83	0.28									
22.840	545.25	0.28									
22.860	545.62	0.28									
22.880	546.05	0.28									
22.900	546.54	0.28									
22.920	546.95	0.28									
22.940	547.36	0.28									
22.960	547.78	0.28									
22.980	548.21	0.28									
23.000	548.62	0.28									
23.020	549.02	0.28									
23.040	549.41	0.28									
23.060	549.83	0.28									
23.080	550.25	0.28									
23.100	550.68	0.28									
23.120	551.13	0.28									
23.140	551.52	0.28									
23.160	551.93	0.28									
23.180	552.37	0.28									
23.199	552.79	0.28									
23.219	553.19	0.28									

23.239	553.56	0.28									
23.259	553.95	0.28									
23.279	554.35	0.28									
23.299	554.75	0.28									
23.319	555.15	0.28									
23.339	555.56	0.28									
23.359	555.97	0.28									
23.379	556.33	0.28									
23.399	556.67	0.28									
23.419	556.93	0.28									
23.439	557.25	0.28									
23.459	557.67	0.28									
23.479	558.07	0.28									
23.499	558.46	0.28									
23.519	558.88	0.28									
23.538	559.26	0.28									
23.558	559.67	0.28									
23.578	560.03	0.28									
23.598	560.47	0.28									
23.618	560.84	0.28									
23.638	561.24	0.28									
23.658	561.66	0.28									
23.678	562.05	0.28									
23.698	562.46	0.28									
23.718	562.87	0.28									
23.738	563.24	0.28									
23.758	563.64	0.28									
23.778	564.06	0.28									
23.798	564.41	0.28									
23.818	564.81	0.28									
23.838	565.24	0.28									
23.858	565.63	0.28									
23.878	566.02	0.28									
23.897	566.40	0.28									
23.917	566.76	0.28									
23.937	567.14	0.28									
23.957	567.53	0.28									
23.977	567.95	0.28									
23.997	568.36	0.28									
24.017	568.73	0.28									
24.037	569.13	0.28									
24.057	569.52	0.28									
24.077	569.90	0.28									
24.097	570.22	0.28									
24.117	570.60	0.28									

24.137	571.00	0.28									
24.157	571.38	0.29									
24.177	571.75	0.29									
24.197	572.11	0.29									
24.217	572.51	0.29									
24.236	572.91	0.29									
24.256	573.31	0.29									
24.276	573.67	0.29									
24.296	574.03	0.29									
24.316	574.38	0.29									
24.336	574.76	0.29									
24.356	575.16	0.29									
24.376	575.52	0.29									
24.396	575.85	0.29									
24.416	576.22	0.29									
24.436	576.61	0.29									
24.456	576.96	0.29									
24.476	577.29	0.29									
24.496	577.57	0.29									
24.516	577.80	0.29									
24.536	578.13	0.29									
24.556	578.50	0.29									
24.576	578.88	0.29									
24.595	579.27	0.29									
24.615	579.64	0.29									
24.635	580.05	0.29									
24.655	580.38	0.29									
24.675	580.76	0.29									
24.695	581.10	0.29									
24.715	581.44	0.29									
24.735	581.82	0.29									
24.755	582.20	0.29									
24.775	582.56	0.29									
24.795	582.91	0.29									
24.815	583.25	0.29									
24.835	583.60	0.29									
24.855	583.99	0.29									
24.875	584.37	0.29									
24.895	584.72	0.29									
24.915	585.09	0.29									
24.934	585.44	0.29									
24.954	585.76	0.29									
24.974	586.14	0.29									
24.994	586.49	0.29									
25.014	586.82	0.29									

25.034	587.18	0.29									
25.054	587.54	0.29									
25.074	587.89	0.29									
25.094	588.24	0.29									
25.114	588.59	0.29									
25.134	588.93	0.29									
25.154	589.28	0.29									
25.174	589.63	0.29									
25.194	589.98	0.29									
25.214	590.32	0.29									
25.234	590.66	0.29									
25.254	591.01	0.29									
25.274	591.39	0.29									
25.293	591.74	0.29									
25.313	592.06	0.29									
25.333	592.34	0.29									
25.353	592.57	0.29									
25.373	592.83	0.29									
25.393	593.19	0.29									
25.413	593.56	0.29									
25.433	593.91	0.29									
25.453	594.25	0.29									
25.473	594.58	0.29									
25.493	594.93	0.29									
25.513	595.27	0.29									
25.533	595.62	0.29									
25.553	595.97	0.29									
25.573	596.31	0.29									
25.593	596.63	0.29									
25.613	596.95	0.29									
25.632	597.26	0.29									
25.652	597.59	0.29									
25.672	597.94	0.29									
25.692	598.27	0.29									
25.712	598.60	0.29									
25.732	598.92	0.29									
25.752	599.28	0.29									
25.772	599.67	0.29									
25.792	599.96	0.29									
25.812	600.32	0.29									
25.832	600.66	0.29									
25.852	600.97	0.29									
25.872	601.33	0.29									
25.892	601.67	0.29									
25.912	602.01	0.29									

25.932	602.34	0.29									
25.952	602.65	0.29									
25.972	602.96	0.29									
25.991	603.27	0.29									
26.011	603.62	0.29									
26.031	603.96	0.29									
26.051	604.27	0.29									
26.071	604.58	0.29									
26.091	604.90	0.29									
26.111	605.21	0.29									
26.131	605.51	0.29									
26.151	605.77	0.29									
26.171	606.05	0.29									
26.191	606.40	0.29									
26.211	606.75	0.29									
26.231	607.08	0.29									
26.251	607.39	0.29									
26.271	607.71	0.29									
26.291	608.02	0.29									
26.311	608.33	0.29									
26.330	608.64	0.29									
26.350	608.97	0.29									
26.370	609.35	0.29									
26.390	609.66	0.29									
26.410	609.94	0.29									
26.430	610.21	0.29									
26.450	610.56	0.29									
26.470	610.90	0.29									
26.490	611.22	0.29									
26.510	611.54	0.29									
26.530	611.86	0.29									
26.550	612.17	0.29									
26.570	612.48	0.29									
26.590	612.78	0.29									
26.610	613.07	0.29									
26.630	613.36	0.29									
26.650	613.66	0.29									
26.670	613.94	0.29									
26.689	614.22	0.29									
26.709	614.52	0.29									
26.729	614.80	0.29									
26.749	615.09	0.29									
26.769	615.43	0.29									
26.789	615.78	0.29									
26.809	616.08	0.29									

26.829	616.42	0.29									
26.849	616.70	0.29									
26.869	616.97	0.29									
26.889	617.30	0.29									
26.909	617.62	0.29									
26.929	617.93	0.29									
26.949	618.26	0.29									
26.969	618.53	0.29									
26.989	618.83	0.29									
27.009	619.14	0.29									
27.028	619.42	0.29									
27.048	619.78	0.29									
27.068	620.11	0.29									
27.088	620.40	0.29									
27.108	620.68	0.29									
27.128	620.99	0.29									
27.148	621.27	0.29									
27.168	621.58	0.29									
27.188	621.91	0.29									
27.208	622.23	0.29									
27.228	622.54	0.29									
27.248	622.85	0.29									
27.268	623.10	0.30									
27.288	623.39	0.30									
27.308	623.70	0.30									
27.328	623.99	0.30									
27.348	624.31	0.30									
27.368	624.63	0.30									
27.387	624.93	0.30									
27.407	625.21	0.30									
27.427	625.45	0.30									
27.447	625.82	0.30									
27.467	626.12	0.30									
27.487	626.39	0.30									
27.507	626.66	0.30									
27.527	626.99	0.30									
27.547	627.32	0.30									
27.567	627.60	0.30									
27.587	627.87	0.30									
27.607	628.16	0.30									
27.627	628.46	0.30									
27.647	628.73	0.30									
27.667	629.02	0.30									
27.687	629.31	0.30									
27.707	629.59	0.30									

27.727	629.88	0.30									
27.746	630.16	0.30									
27.766	630.45	0.30									
27.786	630.78	0.30									
27.806	631.07	0.30									
27.826	631.33	0.30									
27.846	631.60	0.30									
27.866	631.90	0.30									
27.886	632.21	0.30									
27.906	632.51	0.30									
27.926	632.79	0.30									
27.946	633.05	0.30									
27.966	633.35	0.30									
27.986	633.64	0.30									
28.006	633.90	0.30									
28.026	634.21	0.30									
28.046	634.50	0.30									
28.066	634.75	0.30									
28.085	635.03	0.30									
28.105	635.32	0.30									
28.125	635.61	0.30									
28.145	635.89	0.30									
28.165	636.17	0.30									
28.185	636.42	0.30									
28.205	636.67	0.30									
28.225	636.97	0.30									
28.245	637.25	0.30									
28.265	637.54	0.30									
28.285	637.83	0.30									
28.305	638.12	0.30									
28.325	638.39	0.30									
28.345	638.67	0.30									
28.365	638.96	0.30									
28.385	639.24	0.30									
28.405	639.49	0.30									
28.425	639.76	0.30									
28.444	640.04	0.30									
28.464	640.31	0.30									
28.484	640.58	0.30									
28.504	640.84	0.30									
28.524	641.08	0.30									
28.544	641.34	0.30									
28.564	641.65	0.30									
28.584	641.95	0.30									
28.604	642.24	0.30									

28.624	642.51	0.30									
28.644	642.75	0.30									
28.664	642.98	0.30									
28.684	643.23	0.30									
28.704	643.47	0.30									
28.724	643.75	0.30									
28.744	644.03	0.30									
28.764	644.31	0.30									
28.783	644.58	0.30									
28.803	644.84	0.30									
28.823	645.07	0.30									
28.843	645.30	0.30									
28.863	645.48	0.30									
28.883	645.66	0.30									
28.903	645.94	0.30									
28.923	646.20	0.30									
28.943	646.51	0.30									
28.963	646.79	0.30									
28.983	647.05	0.30									
29.003	647.33	0.30									
29.023	647.61	0.30									
29.043	647.87	0.30									
29.063	648.13	0.30									
29.083	648.39	0.30									
29.103	648.65	0.30									
29.123	648.92	0.30									
29.142	649.19	0.30									
29.162	649.44	0.30									
29.182	649.65	0.30									
29.202	649.93	0.30									
29.222	650.26	0.30									
29.242	650.51	0.30									
29.262	650.75	0.30									
29.282	650.99	0.30									
29.302	651.23	0.30									
29.322	651.52	0.30									
29.342	651.80	0.30									
29.362	652.02	0.30									
29.382	652.30	0.30									
29.402	652.56	0.30									
29.422	652.80	0.30									
29.442	653.08	0.30									
29.462	653.32	0.30									
29.481	653.57	0.30									
29.501	653.86	0.30									

29.521	654.09	0.30									
29.541	654.35	0.30									
29.561	654.62	0.30									
29.581	654.89	0.30									
29.601	655.12	0.30									
29.621	655.35	0.30									
29.641	655.62	0.30									
29.661	655.90	0.30									
29.681	656.17	0.30									
29.701	656.41	0.30									
29.721	656.66	0.30									
29.741	656.90	0.30									
29.761	657.15	0.30									
29.781	657.39	0.30									
29.801	657.63	0.30									
29.821	657.88	0.30									
29.840	658.15	0.30									
29.860	658.38	0.30									
29.880	658.60	0.30									
29.900	658.86	0.30									
29.920	659.12	0.30									
29.940	659.38	0.30									
29.960	659.62	0.30									
29.980	659.83	0.30									
30.000	660.06	0.30									

Combined standard uncertainties:

$u(T)$= 0.006 K; $u(p)$= 0.0020 MPa for p < 6 MPa; $u(p)$= 0.024 MPa for 6 MPa ≤ p ≤ 70 MPa

$u(x_{CO_2})$= 0.00025; $u(x_{NO})$= 0.000014; $u(x_{SO_2})$= 0.0000023; $u(x_{CO})$= 0.0000043

Table S2. Experimental dew and bubble pressures, p_{dew} and p_{bubble} , respectively, and densities of the vapor, ρ_V, and liquid, ρ_L, phases in the VLE for the $CO_2 + O_2 + SO_2 + CO$ (Mix 1) and $CO_2 + NO + SO_2 + CO$ (Mix 2) mixtures and the corresponding combined standard uncertainties. Pure CO_2 data are included for comparison; p_{sat}: saturation pressure.

T **(K)**	p_{dew} **(MPa)**	$u(p_{dew})$ **(MPa)**	ρ_V **(kg·m⁻³)**	$u(\rho_V)$ **(kg·m⁻³)**	***T*** **(K)**	p_{bubble} **(MPa)**	$u(p_{bubble})$ **(MPa)**	ρ_L **(kg·m⁻³)**	$u(\rho_L)$ **(kg·m⁻³)**
**Mix 1: x_{CO_2} = 0.96734; x_{O_2} = 0.030038; x_{SO_2} = 0.0009035; x_{CO} = 0.0017032**									
**263.18**	2.744	0.0270	72.64	1.10	**263.14**	4.082	0.101	960.94	0.79
**273.19**	3.629	0.0183	99.63	1.09	**273.10**	4.849	0.099	903.20	1.32
**283.20**	4.716	0.0126	138.27	0.29	**283.22**	5.802	0.062	833.34	1.10
**293.13**	6.041	0.0120	198.28	1.48	**293.13**	6.879	0.057	738.77	1.79
**Mix 2: x_{CO_2} = 0.99592; x_{NO} = 0.001410; x_{SO_2} = 0.0009100; x_{CO} = 0.0017002**									
**263.17**	2.660	0.0061	71.29	0.50	**263.15**	2.755	0.054	981.09	0.81
**273.15**	3.500	0.0083	97.70	0.71	**273.17**	3.562	0.036	925.65	0.55
**283.15**	4.532	0.0072	135.44	0.86	**283.18**	4.573	0.044	859.16	0.93
**293.14**	5.794	0.0071	195.69	1.25	**293.17**	5.809	0.044	770.74	1.22
**Pure CO_2 [a]**									
	p_{sat} **(MPa)**	ρ_V **(kg·m⁻³)**	ρ_L **(kg·m⁻³)**						
**263.15**	2.649	71.19	982.93						
**273.15**	3.485	97.65	927.43						
**283.15**	4.502	135.16	861.12						
**293.15**	5.729	194.20	773.39						

(a) Span, R.; Wagner, W. A New Equation of State for Carbon Dioxide Covering the Fluid Region from the Triple-Point Temperature to 1100 K at Pressures up to 800 MPa, *J. Phys. Chem. Ref. Data* **1996**, *25*, 1509-1596. DOI: 10.1063/1.555991

Table S3. Mole fraction composition, x_i, and the standard uncertainty of the mole fraction composition, $u(x_i)$, of the mixtures prepared for the uncertainty study.

**Component**	**CO_2 + CH_3OH + O_2 + SO_2 + CO (doped Mix 1 for uncertainty)**		**CO_2 + CH_3OH + NO + SO_2 + CO (doped Mix 2 for uncertainty)**	
	$\boldsymbol{x_i}$	$\boldsymbol{u(x_i)}$	$\boldsymbol{x_i}$	$\boldsymbol{u(x_i)}$
**CO_2**	0.95767	0.00058	0.98584	0.00016
**CH_3OH**	0.01001	0.00011	0.01018	0.00012
**O_2**	0.02974	0.00048		
**NO**			0.001396	0.000026
**SO_2**	0.000893	0.000015	0.000899	0.000015
**CO**	0.001686	0.000028	0.001683	0.000027

Table S4. pcT experimental data for the CO_2 + CH_3OH + O_2 + SO_2 + CO and CO_2 + CH_3OH + NO + SO_2 + CO (doped Mix 1 and doped Mix 2) mixtures used for the uncertainty study.

**p (MPa)**	**c (m·s^{-1})**	**p (MPa)**	**c (m·s^{-1})**	**p (MPa)**	**c (m·s^{-1})**
**Doped Mix 1 for uncertainty:** $x_{CO_2} = 0.95769; x_{CH_3OH} = 0.00999; x_{O_2} = 0.02974; x_{SO_2} = 0.000893; x_{CO} = 0.001686$					
**T = 263.15±0.01 K**		**T = 293.15±0.01 K**		**T = 313.15±0.01 K**	
194.96	1406.36	194.60	1329.85	189.96	1272.62
189.96	1395.26	189.96	1318.72	179.96	1247.99
179.96	1372.76	179.96	1294.76	169.96	1222.43
169.96	1349.32	169.96	1270.03	159.96	1195.78
159.96	1325.10	159.96	1244.29	149.96	1167.95
149.96	1299.87	149.96	1217.42	139.96	1138.78
139.96	1273.62	139.96	1189.35	129.96	1108.19
129.96	1246.16	129.96	1159.76	119.96	1075.85
119.96	1217.32	119.96	1128.75	109.96	1041.50
109.96	1186.96	109.96	1095.83	99.96	1004.85
99.96	1154.91	99.96	1060.83	94.96	985.58
94.96	1138.14	94.96	1042.43	89.96	965.48
89.96	1120.82	89.96	1023.33	84.96	944.63
84.96	1102.91	84.96	1003.50	79.96	922.80
79.96	1084.35	79.96	982.88	74.96	899.97
74.96	1065.05	74.96	961.38	69.96	875.80
69.96	1044.98	69.96	938.76	64.96	850.62
64.96	1024.03	64.96	914.91	59.96	823.84
59.96	1002.14	59.96	889.87	54.96	795.22
54.96	979.12	54.96	863.35	49.96	764.46
49.97	954.75	49.96	835.09	44.96	731.24
44.98	929.11	44.96	804.62		
39.98	901.79	39.96	771.66		
34.98	872.24	35.00	735.77		
29.97	840.29	32.96	719.74		
24.97	805.34				

19.96	766.29				
14.97	721.98				
10.01	670.08				
***T* = 263.15±0.01 K**		***T* = 293.15±0.00 K**		***T* = 313.16±0.01 K**	
194.97	1407.06	169.30	1268.76	189.97	1272.91
189.96	1396.01	159.96	1244.64	179.96	1248.30
179.96	1373.48	149.96	1217.74	169.96	1222.76
169.96	1350.10	139.96	1189.66	159.96	1196.14
159.96	1325.87	129.96	1160.16	149.96	1168.32
149.96	1300.66	119.96	1129.08	139.96	1139.18
139.96	1274.41	109.96	1096.2	129.96	1108.59
129.96	1246.95	99.96	1061.24	119.96	1076.17
119.96	1218.20	94.96	1042.82	109.96	1041.85
109.96	1187.89	89.96	1023.76	99.96	1005.21
99.96	1155.87	84.96	1003.95	94.96	985.89
89.96	1121.83	79.96	983.33	89.96	965.85
79.96	1085.36	74.96	961.78	84.96	945.02
69.96	1046.12	69.96	939.24	79.96	923.13
59.96	1003.35	64.96	915.52	74.96	900.37
49.98	956.12	59.96	890.53	69.96	876.37
39.98	903.15	54.96	863.98	64.96	851.09
29.98	842.08	49.96	835.74	59.96	824.01
19.97	768.52	44.98	805.31	54.96	795.58
10.04	673.23	39.97	772.33	49.96	764.89
		34.97	736.2	44.96	731.66
		29.96	695.79		
		27.78	676.46		

Combined standard uncertainties:

$u(T) = 0.015$ K; $u(p) = 0.02$ MPa; $u^{*}(c) = 8.9 \times 10^{-4}c$; $u(c) = 8.9 \times 10^{-4}c$;

$u\left(x_{\mathrm{CO_2}}\right)$= 0.00058; $u\left(x_{\mathrm{CH_3OH}}\right)$= 0.00011; $u\left(x_{\mathrm{O_2}}\right) = 0.00047$; $u\left(x_{\mathrm{SO_2}}\right) =$

0.000015; $u(x_{\mathrm{CO}}) = 0.000028$.

Table S4 (continued). pcT experimental data for the CO_2 + CH_3OH + O_2 + SO_2 + CO and CO_2 + CH_3OH + NO + SO_2 + CO (doped Mix 1 and doped Mix 2) mixtures used for the uncertainty study.

p (MPa)	*c* (m·s^{-1})	*p* (MPa)	*c* (m·s^{-1})	*p* (MPa)	*c* (m·s^{-1})
**Doped Mix 1 for uncertainty:** $x_{CO_2} = 0.95767; x_{CH_3OH} = 0.01001; x_{O_2} = 0.02974; x_{SO_2} = 0.000893;\ x_{CO} = 0.001686$					
***T* = 263.15±0.01 K**		***T* = 293.15±0.01 K**		***T* = 313.14±0.00 K**	
183.46	1380.23	175.15	1283.19	184.42	1258.25
179.96	1372.28	169.95	1270.16	179.96	1247.18
169.96	1348.94	159.96	1244.48	169.96	1221.64
159.96	1324.69	149.96	1217.58	159.96	1194.98
149.96	1299.50	139.96	1189.49	149.96	1167.17
139.96	1273.23	129.96	1159.97	139.96	1138.00
129.96	1245.65	119.96	1128.91	129.96	1107.40
119.96	1216.94	109.96	1096.06	119.96	1075.01
109.96	1186.53	99.96	1061.08	109.96	1040.67
99.96	1154.45	94.96	1042.73	104.96	1022.64
94.96	1137.60	89.96	1023.65	99.96	1004.02
89.96	1120.32	84.96	1003.76	94.96	984.70
84.96	1102.27	79.96	983.17	89.96	964.65
79.96	1083.71	74.96	961.76	84.96	943.75
74.96	1064.40	69.96	939.14	79.96	921.95
69.96	1044.11	64.96	915.37	74.96	899.14
64.96	1023.27	59.96	890.44	69.96	875.05
59.96	1001.23	54.96	863.88	67.47	862.56
54.96	978.16	49.96	835.59		
49.96	953.73	45.00	805.44		
44.96	927.76	44.97	805.13		
39.96	900.41	39.97	772.16		
34.97	870.80	35.98	743.61		
29.97	838.61				
24.97	803.42				

19.98	764.18				
15.03	719.62				
12.49	693.77				
		**T = 293.15±0.01 K**		**T = 313.14±0.00 K**	
		136.10	1178.77	184.46	1258.39
		129.96	1160.51	179.96	1247.25
		119.96	1129.48	169.96	1221.68
		109.96	1096.64	159.96	1195.04
		99.96	1061.64	149.96	1167.21
		94.96	1043.29	139.96	1138.07
		89.96	1024.29	129.96	1107.45
		84.96	1004.5	119.96	1075.06
		79.96	983.86	109.96	1040.72
		74.96	962.32	104.96	1022.75
		69.96	939.8	99.96	1004.07
		64.96	916.14	94.96	984.78
		59.96	891.11	89.96	964.72
		54.96	864.58	84.96	943.83
		49.96	836.34	79.96	922.02
		44.96	805.92	74.96	899.18
		39.96	772.97	69.96	875.17
		36.59	749.03	64.96	849.88
				59.96	823.03
				54.96	794.30
				53.96	788.41

Combined standard uncertainties:

$u(T) = 0.015$ K; $u(p) = 0.02$ MPa; $u^*(c) = 8.9 \times 10^{-4} c$; $u(c) = 8.9 \times 10^{-4} c$; $u(x_{CO_2})$= 0.00058; $u(x_{CH_3OH})$= 0.00011; $u(x_{O_2}) = 0.00048$; $u(x_{SO_2}) = 0.000015$; $u(x_{CO}) = 0.000028$.

Table S4 (continued). pcT experimental data for the CO_2 + CH_3OH + O_2 + SO_2 + CO and CO_2 + CH_3OH + NO + SO_2 + CO (doped Mix 1 and doped Mix 2) mixtures used for the uncertainty study.

p (MPa)	*c* (m·s^{-1})	*p* (MPa)	*c* (m·s^{-1})	*p* (MPa)	*c* (m·s^{-1})
**Doped Mix 2 for uncertainty:** $x_{CO_2} = 0.98584; x_{CH_3OH} = 0.01018; x_{NO} = 0.001396; x_{SO_2} = 0.000899; x_{CO} = 0.001683$					
***T* = 263.15±0.01 K**		***T* = 293.15±0.01 K**		***T* = 313.15±0.01 K**	
194.96	1423.19	189.96	1333.27	189.96	1284.40
189.96	1412.32	179.96	1309.64	179.96	1259.96
179.96	1390.05	169.96	1285.14	169.96	1234.57
169.96	1366.91	159.96	1259.66	159.96	1208.12
159.96	1343.03	149.96	1233.08	149.96	1180.50
149.96	1318.13	139.96	1205.25	139.96	1151.58
139.96	1292.22	129.96	1176.13	129.96	1121.27
129.96	1265.09	119.96	1145.44	119.96	1089.20
119.96	1236.75	109.96	1113.03	109.96	1055.25
109.96	1206.96	99.96	1078.60	99.96	1019.00
99.96	1175.46	89.96	1041.71	94.96	999.91
89.96	1142.06	84.96	1022.23	89.96	980.11
79.96	1106.35	79.96	1001.99	84.96	959.47
69.96	1067.93	74.96	980.90	79.96	937.85
64.96	1047.48	69.96	958.77	74.96	915.36
59.96	1026.13	64.96	935.60	69.96	891.74
54.96	1003.80	59.96	911.17	64.96	866.86
49.98	980.27	54.96	885.26	59.96	840.51
44.97	955.44	49.96	857.77	54.97	812.53
39.97	929.07	44.96	828.24	49.97	782.40
34.96	900.69	39.96	796.42		
29.96	870.29	34.96	761.62		
24.97	837.17	29.96	722.96		
19.97	800.47				
14.97	759.38				

9.99	711.71				

T = 263.14±0.01 K		*T* = 293.15±0.00 K		T = 313.16±0.01 K	
189.96	1412.17	189.96	1333.03	189.96	1284.38
179.96	1389.94	179.96	1309.38	179.96	1259.97
169.96	1366.85	169.96	1284.89	169.96	1234.56
159.96	1342.92	159.96	1259.40	159.96	1208.12
149.96	1318.01	149.96	1232.81	149.96	1180.49
139.96	1292.13	139.96	1204.97	139.96	1151.60
129.96	1265.08	129.96	1175.81	129.96	1121.24
119.96	1236.71	119.96	1145.13	119.96	1089.20
109.96	1206.81	109.96	1112.72	109.96	1055.23
99.96	1175.32	99.96	1078.25	99.96	1019.02
89.96	1141.94	89.96	1041.35	94.96	999.91
79.96	1106.27	84.96	1021.88	89.96	980.08
69.96	1067.74	79.96	1001.65	84.96	959.47
64.96	1047.33	74.96	980.53	79.96	937.97
59.96	1026.06	69.96	958.36	74.96	915.45
54.96	1003.71	64.96	935.23	69.96	891.85
49.98	980.20	59.96	910.75	64.96	866.94
44.96	955.37	54.96	884.90	59.96	840.61
39.96	928.84	49.96	857.35	54.96	812.47
34.95	900.66	44.97	827.81	49.96	782.49
29.96	870.15	39.98	795.98		
24.96	836.95	34.97	761.14		
19.96	800.38	29.97	722.44		
14.95	758.96				
9.96	711.24				

Combined standard uncertainties:

$u(T) = 0.015$ K; $u(p) = 0.02$ MPa; $u^{*}(c) = 4.6 \times 10^{-4}c;$ $u(c) = 4.6 \times 10^{-4}c;$ $u(x_{CO_2}) = 0.00016;$ $u(x_{CH_3OH}) = 0.00012;$ $u(x_{NO}) = 0.000026;$ $u(x_{SO_2}) = 0.000015;$ $u(x_{CO}) = 0.000027.$

Table S4 (continued). pcT experimental data for the CO_2 + CH_3OH + O_2 + SO_2+ CO and CO_2 + CH_3OH + NO + SO_2+ CO (doped Mix 1 and doped Mix 2) mixtures used for the uncertainty study.

**p (MPa)**	**c (m·s⁻¹)**	**p (MPa)**	**c (m·s⁻¹)**	**p (MPa)**	**c (m·s⁻¹)**
**Doped Mix 2 for uncertainty:** $x_{CO_2} = 0.98586; x_{CH_3OH} = 0.01016; x_{NO} = 0.001396; x_{SO_2} = 0.000899; x_{CO} = 0.001683$					
***T* = 263.14±0.01 K**		***T* = 293.15±0.01 K**		***T* = 313.15±0.01 K**	
194.96	1422.51	189.96	1333.30	189.96	1284.71
189.96	1411.65	179.96	1309.66	179.96	1260.25
179.96	1389.38	169.96	1285.20	169.96	1234.85
169.96	1366.21	159.96	1259.65	159.96	1208.41
159.96	1342.30	149.96	1233.11	149.96	1180.81
149.96	1317.40	139.96	1205.31	139.96	1151.92
139.96	1291.45	129.96	1176.08	129.96	1121.62
129.96	1264.34	119.96	1145.42	119.96	1089.55
119.96	1236.00	109.96	1113.01	109.96	1055.72
109.96	1206.14	99.96	1078.56	99.96	1019.47
99.96	1174.62	89.96	1041.70	94.96	1000.37
89.96	1141.17	84.96	1022.23	89.96	980.54
79.96	1105.45	79.96	1001.99	84.96	959.90
69.96	1067.01	74.96	980.91	79.96	938.44
64.96	1046.58	69.96	958.79	74.96	915.91
59.96	1025.22	64.96	935.60	69.96	892.30
54.96	1002.87	59.96	911.16	64.96	867.41
49.97	979.32	54.96	885.34	59.96	841.11
44.96	954.39	49.97	857.74	54.96	812.94
39.96	927.99	44.97	828.34	49.96	782.94
34.96	899.67	39.97	796.47	44.96	750.42
29.97	869.21	34.96	761.62		
24.96	835.95	29.97	723.00		
19.96	799.24				
14.95	757.83				
9.96	710.03				

T = 263.15±0.01 K		T = 293.15±0.00 K		T = 313.16±0.01 K	
194.96	1422.84	184.66	1320.67	194.96	1296.57
189.96	1411.93	179.96	1309.43	189.96	1284.70
179.96	1389.66	169.96	1284.93	179.96	1260.27
169.96	1366.53	159.96	1259.40	169.96	1234.88
159.96	1342.61	149.96	1232.81	159.96	1208.45
149.96	1317.71	139.96	1205.00	149.96	1180.86
139.96	1291.83	129.96	1175.83	139.96	1151.94
129.96	1264.75	119.96	1145.12	129.96	1121.62
119.96	1236.38	109.96	1112.70	119.96	1089.56
109.96	1206.58	99.96	1078.25	109.96	1055.60
99.96	1174.96	94.96	1060.13	99.96	1019.42
89.96	1141.53	89.96	1041.39	94.96	1000.29
79.96	1105.87	84.96	1021.92	89.96	980.49
69.96	1067.39	79.96	1001.69	84.96	959.89
64.96	1047.00	74.96	980.54	79.96	938.41
59.96	1025.63	69.96	958.44	74.96	915.89
54.96	1003.26	64.96	935.25	69.96	892.30
49.96	979.63	59.96	910.85	64.96	867.44
44.97	954.82	54.96	884.91	59.96	841.01
39.96	928.33	49.98	857.36	54.96	813.02
34.96	899.98	44.98	827.86	49.96	782.94
29.96	869.51	39.97	795.98	46.97	763.75
24.94	836.12	34.97	761.13		
19.96	799.54				
14.96	758.19				
9.95	710.29				

Combined standard uncertainties:

$u(T)$ = 0.015 K; $u(p)$ = 0.02 MPa; $u^*(c) = 4.6 \times 10^{-4}c$; $u(c) = 4.6 \times 10^{-4}c$; $u(x_{CO_2}) = 0.00016$; $u(x_{CH_3OH}) = 0.00011$; $u(x_{NO}) = 0.000026$; $u(x_{SO_2}) = 0.000015$; $u(x_{CO}) = 0.000027$.

Table S5. Reduced $p\rho T$ experimental data for the CO_2 + O_2 + SO_2 + CO (Mix 1) and CO_2 + NO + SO_2 + CO (Mix 2) mixtures. Z: compressibility factor; $u(\rho)$, $u(Z)$: combined standard uncertainties.

**Mix 1: x_{CO_2} = 0.96734; x_{O_2} = 0.030038; x_{SO_2} = 0.0009035; x_{CO} = 0.0017032**									
**p (MPa)**	**ρ (kg.m^{-3})**	**$u(\rho)$ (kg.m^{-3})**	**Z**	**$u(Z)$**	**p (MPa)**	**ρ (kg.m^{-3})**	**$u(\rho)$ (kg.m^{-3})**	**Z**	**$u(Z)$**
***T*= 263.18±0.02 K**					***T*= 273.19±0.01 K**				
0.100	1.95	0.22	1.02	0.12	0.100	1.84	0.22	1.04	0.13
0.389	7.93	0.22	0.978	0.028	0.392	7.62	0.22	0.989	0.029
0.678	14.25	0.22	0.949	0.015	0.684	13.70	0.22	0.960	0.016
0.967	20.78	0.22	0.928	0.010	0.976	19.93	0.22	0.941	0.011
1.256	27.71	0.22	0.9038	0.0074	1.268	26.54	0.22	0.9181	0.0079
1.545	34.98	0.22	0.8807	0.0057	1.560	33.44	0.22	0.8964	0.0061
1.834	42.88	0.22	0.8527	0.0045	1.852	40.76	0.22	0.8731	0.0049
2.123	51.39	0.22	0.8237	0.0037	2.144	48.57	0.22	0.8482	0.0040
2.412	60.75	0.22	0.7917	0.0030	2.436	56.86	0.22	0.8232	0.0033
2.689	70.64	0.22	0.7592	0.0024	2.729	66.00	0.22	0.7943	0.0028
2.734	72.37	0.22	0.7533	0.0023	3.021	75.80	0.22	0.7656	0.0023
					3.313	86.59	0.22	0.7350	0.0020
					3.618	99.55	0.22	0.6983	0.0016
***T*= 263.14±0.02 K**					***T*= 273.10±0.01 K**				
4.103	961.17	0.37	0.08514	0.00050	4.910	903.43	0.36	0.10444	0.00051
4.417	963.29	0.37	0.09146	0.00050	5.321	907.56	0.36	0.11267	0.00051
4.815	965.98	0.37	0.09942	0.00050	5.732	911.22	0.36	0.12089	0.00051
5.213	968.81	0.37	0.10733	0.00050	6.143	915.34	0.36	0.12898	0.00051
5.611	971.46	0.37	0.11520	0.00050	6.554	919.08	0.36	0.13705	0.00051
6.009	974.00	0.37	0.12305	0.00049	6.966	922.68	0.36	0.14508	0.00050
6.407	976.47	0.37	0.13087	0.00049	7.377	926.09	0.36	0.15308	0.00050
6.805	978.84	0.37	0.13866	0.00049	7.788	929.32	0.36	0.16105	0.00050
7.203	981.24	0.38	0.14641	0.00049	8.199	932.55	0.36	0.16897	0.00050
7.601	983.48	0.38	0.15415	0.00049	8.610	935.70	0.36	0.17684	0.00050
7.999	985.67	0.38	0.16186	0.00049	9.021	938.72	0.36	0.18469	0.00050
8.397	987.87	0.38	0.16953	0.00049	9.433	941.65	0.37	0.19251	0.00050
8.795	989.99	0.38	0.17719	0.00049	9.844	944.49	0.37	0.20030	0.00050
9.193	992.05	0.38	0.18482	0.00049	10.255	947.24	0.37	0.20806	0.00050
9.590	994.13	0.38	0.19242	0.00049	10.461	948.64	0.37	0.21192	0.00050
9.988	996.13	0.38	0.20000	0.00049	10.666	949.96	0.37	0.21578	0.00050
10.386	998.10	0.38	0.20756	0.00049	11.077	952.61	0.37	0.22348	0.00049
10.784	1000.06	0.38	0.21509	0.00049	11.489	955.14	0.37	0.23116	0.00049
11.182	1001.99	0.38	0.22260	0.00049	11.900	957.70	0.37	0.23879	0.00049
11.580	1003.86	0.38	0.23009	0.00049	12.311	960.12	0.37	0.24642	0.00049
11.978	1005.74	0.38	0.23755	0.00049	12.722	962.51	0.37	0.25402	0.00049

12.376	1007.53	0.38	0.24501	0.00049	13.133	964.85	0.37	0.26159	0.00049
12.774	1009.30	0.38	0.25244	0.00049	13.544	967.21	0.37	0.26912	0.00049
13.172	1011.07	0.38	0.25985	0.00049	13.956	969.42	0.37	0.27666	0.00049
13.570	1012.78	0.38	0.26725	0.00049	14.367	971.62	0.37	0.28417	0.00049
13.968	1014.45	0.38	0.27463	0.00049	14.778	973.85	0.37	0.29163	0.00049
14.366	1016.13	0.38	0.28199	0.00049	15.189	975.93	0.37	0.29911	0.00049
14.764	1017.85	0.38	0.28931	0.00049	15.600	978.01	0.37	0.30655	0.00049
15.162	1019.50	0.38	0.29663	0.00049	16.012	979.98	0.37	0.31400	0.00049
15.560	1021.06	0.39	0.30395	0.00049	16.423	982.01	0.38	0.32140	0.00049
15.958	1022.59	0.39	0.31126	0.00049	16.834	983.96	0.38	0.32879	0.00049
16.356	1024.24	0.39	0.31851	0.00049	17.245	985.92	0.38	0.33615	0.00049
16.754	1025.76	0.39	0.32577	0.00049	17.656	987.85	0.38	0.34350	0.00049
17.152	1027.30	0.39	0.33301	0.00049	18.067	989.75	0.38	0.35082	0.00049
17.549	1028.79	0.39	0.34024	0.00049	18.479	991.61	0.38	0.35813	0.00049
17.947	1030.28	0.39	0.34746	0.00049	18.890	993.43	0.38	0.36543	0.00049
18.345	1031.80	0.39	0.35464	0.00049	19.301	995.20	0.38	0.37272	0.00049
18.743	1033.22	0.39	0.36183	0.00049	19.712	996.97	0.38	0.37998	0.00049
19.162	1034.77	0.39	0.36936	0.00049	20.000	998.22	0.38	0.38505	0.00049
19.581	1036.30	0.39	0.37688	0.00049					
20.000	1037.77	0.39	0.38440	0.00049					
***T*= 283.20±0.02 K**					***T*= 293.13±0.02 K**				
0.100	1.82	0.22	1.02	0.12	0.100	1.74	0.22	1.03	0.13
0.429	8.10	0.22	0.983	0.027	0.466	8.44	0.22	0.988	0.026
0.774	15.01	0.22	0.956	0.014	0.831	15.47	0.22	0.962	0.014
1.118	22.14	0.22	0.9360	0.0096	1.213	23.09	0.22	0.9410	0.0092
1.462	29.64	0.22	0.9144	0.0070	1.579	30.68	0.22	0.9213	0.0068
1.792	37.22	0.22	0.8921	0.0055	1.795	35.40	0.22	0.9077	0.0058
2.121	45.24	0.22	0.8689	0.0044	2.160	43.84	0.22	0.8822	0.0046
2.450	53.74	0.22	0.8450	0.0036	2.542	53.05	0.22	0.8582	0.0037
2.780	62.86	0.22	0.8196	0.0030	3.074	66.85	0.22	0.8233	0.0028
3.109	72.67	0.22	0.7929	0.0025	3.456	77.60	0.22	0.7975	0.0023
3.438	83.26	0.22	0.7653	0.0021	3.822	88.77	0.22	0.7709	0.0020
3.768	94.90	0.22	0.7358	0.0018	4.204	101.55	0.22	0.7413	0.0017
4.396	121.61	0.22	0.6700	0.0013	4.570	115.11	0.22	0.7108	0.0014
4.696	137.52	0.21	0.6328	0.0010	5.118	138.94	0.22	0.6595	0.0011
					5.483	158.37	0.22	0.61993	0.00091
					6.032	197.81	0.21	0.54596	0.00063
***T*= 283.22±0.02 K**					***T*= 293.13±0.02 K**				
5.869	834.26	0.34	0.13037	0.00054	7.056	745.06	0.32	0.16957	0.00058
6.279	840.59	0.34	0.13843	0.00053	7.421	758.36	0.32	0.17521	0.00057
6.689	846.90	0.34	0.14638	0.00053	7.806	770.01	0.32	0.18151	0.00056
7.100	853.00	0.34	0.15424	0.00053	8.191	780.15	0.33	0.18799	0.00056
7.510	858.65	0.34	0.16208	0.00052	8.555	788.50	0.33	0.19427	0.00055
7.920	863.98	0.35	0.16988	0.00052	8.920	796.07	0.33	0.20063	0.00055
8.330	869.02	0.35	0.17764	0.00052	9.305	803.46	0.33	0.20736	0.00054

8.740	873.80	0.35	0.18537	0.00052	9.690	810.45	0.33	0.21408	0.00054
9.150	878.36	0.35	0.19306	0.00051	10.054	816.50	0.33	0.22048	0.00054
9.561	882.71	0.35	0.20072	0.00051	10.439	822.18	0.34	0.22733	0.00053
9.971	886.76	0.35	0.20837	0.00051	10.804	827.73	0.34	0.23371	0.00053
10.381	890.69	0.35	0.21599	0.00051	11.189	833.07	0.34	0.24048	0.00053
10.791	894.51	0.35	0.22357	0.00051	11.553	837.76	0.34	0.24692	0.00053
11.201	898.21	0.35	0.23111	0.00051	11.938	842.26	0.34	0.25378	0.00052
11.612	901.73	0.36	0.23863	0.00051	12.323	846.92	0.34	0.26052	0.00052
12.022	905.14	0.36	0.24613	0.00050	12.708	851.20	0.34	0.26731	0.00052
12.432	908.48	0.36	0.25360	0.00050	13.072	855.35	0.34	0.27363	0.00052
12.842	911.69	0.36	0.26104	0.00050	13.437	859.32	0.35	0.27998	0.00052
13.252	914.78	0.36	0.26847	0.00050	13.822	863.31	0.35	0.28667	0.00052
13.663	917.79	0.36	0.27587	0.00050	14.207	867.25	0.35	0.29331	0.00051
14.073	920.73	0.36	0.28325	0.00050	14.571	870.73	0.35	0.29963	0.00051
14.483	923.57	0.36	0.29061	0.00050	14.936	874.05	0.35	0.30596	0.00051
14.893	926.43	0.36	0.29791	0.00050	15.321	877.45	0.35	0.31264	0.00051
15.303	929.09	0.36	0.30524	0.00050	15.685	880.55	0.35	0.31894	0.00051
15.714	931.79	0.36	0.31252	0.00050	16.070	883.18	0.35	0.32580	0.00051
16.124	934.41	0.36	0.31978	0.00050	16.455	886.29	0.35	0.33243	0.00051
16.534	936.97	0.36	0.32701	0.00050	16.820	889.17	0.35	0.33870	0.00051
16.944	939.43	0.36	0.33425	0.00050	17.205	892.23	0.35	0.34527	0.00051
17.354	941.88	0.37	0.34145	0.00050	17.569	894.95	0.35	0.35150	0.00051
17.764	944.30	0.37	0.34863	0.00050	17.954	897.81	0.35	0.35806	0.00051
18.175	946.70	0.37	0.35577	0.00050	18.339	900.59	0.36	0.36461	0.00051
18.585	948.96	0.37	0.36294	0.00050	18.724	903.26	0.36	0.37116	0.00051
18.995	951.15	0.37	0.37009	0.00050	19.109	905.88	0.36	0.37770	0.00051
19.405	953.39	0.37	0.37720	0.00050	19.473	908.28	0.36	0.38387	0.00051
19.820	955.55	0.37	0.38439	0.00050	19.818	910.53	0.36	0.38971	0.00051
20.000	956.56	0.37	0.38747	0.00050	20.000	911.67	0.36	0.39280	0.00051
***T*= 303.09±0.02 K**					***T*= 313.14±0.02 K**				
0.113	1.64	0.21	1.20	0.30	0.114	1.60	0.21	1.20	0.30
0.491	8.40	0.22	1.014	0.056	0.493	8.22	0.22	1.004	0.056
0.890	15.84	0.22	0.972	0.030	0.891	15.29	0.22	0.976	0.030
1.288	23.49	0.22	0.949	0.020	1.289	22.61	0.22	0.955	0.020
1.686	31.48	0.22	0.927	0.015	1.687	30.20	0.22	0.936	0.015
2.084	39.88	0.22	0.905	0.012	2.085	38.18	0.22	0.915	0.012
2.482	48.72	0.22	0.8822	0.0094	2.483	46.39	0.22	0.8972	0.0097
2.880	57.93	0.22	0.8610	0.0079	2.881	55.07	0.22	0.8770	0.0081
3.278	67.78	0.22	0.8375	0.0067	3.279	64.15	0.22	0.8568	0.0069
3.677	78.25	0.22	0.8136	0.0058	3.677	73.71	0.22	0.8362	0.0060
4.075	89.55	0.22	0.7879	0.0050	4.076	83.77	0.22	0.8155	0.0053
4.473	101.80	0.22	0.7608	0.0044	4.474	94.52	0.22	0.7933	0.0047
4.871	115.17	0.22	0.7324	0.0039	4.872	105.97	0.22	0.7706	0.0041
5.269	130.18	0.22	0.7009	0.0034	5.270	118.25	0.22	0.7470	0.0037

5.667	147.28	0.23	0.6663	0.0030	5.668	131.51	0.22	0.7224	0.0033
6.065	167.81	0.23	0.6259	0.0026	6.066	146.12	0.23	0.6958	0.0030
6.463	194.30	0.23	0.5760	0.0022	6.464	162.24	0.23	0.6678	0.0027
6.862	229.97	0.23	0.5167	0.0019	6.862	180.51	0.23	0.6372	0.0024
7.260	289.54	0.21	0.4342	0.0015	7.260	201.79	0.23	0.6031	0.0021
7.658	407.25	0.23	0.3256	0.0010	8.057	257.94	0.23	0.5235	0.0016
8.056	546.12	0.27	0.25544	0.00077	8.455	298.38	0.23	0.4749	0.0014
8.454	622.04	0.29	0.23535	0.00068	8.853	352.83	0.23	0.4205	0.0012
8.852	658.66	0.30	0.23273	0.00064	9.251	420.88	0.24	0.3684	0.0010
9.250	681.77	0.30	0.23495	0.00062	9.649	482.23	0.26	0.33537	0.00086
9.649	699.66	0.31	0.23880	0.00061	10.047	536.23	0.27	0.31405	0.00077
10.047	714.53	0.31	0.24348	0.00059	10.445	572.19	0.28	0.30597	0.00072
10.445	727.20	0.31	0.24872	0.00058	10.843	602.31	0.28	0.30175	0.00069
10.843	738.31	0.32	0.25431	0.00058	11.242	625.22	0.29	0.30136	0.00066
11.240	748.22	0.32	0.26013	0.00057	11.640	645.04	0.29	0.30245	0.00064
11.639	757.25	0.32	0.26616	0.00056	12.038	661.37	0.30	0.30507	0.00063
12.037	765.46	0.32	0.27231	0.00056	12.436	675.89	0.30	0.30839	0.00062
12.435	773.03	0.32	0.27856	0.00055	12.834	688.63	0.30	0.31237	0.00061
12.834	780.11	0.33	0.28487	0.00055	13.232	699.99	0.31	0.31683	0.00060
13.232	786.60	0.33	0.29129	0.00055	13.630	710.53	0.31	0.32153	0.00059
13.630	792.69	0.33	0.29775	0.00054	14.028	719.81	0.31	0.32665	0.00058
14.028	798.69	0.33	0.30414	0.00054	14.426	728.68	0.31	0.33183	0.00058
14.426	804.27	0.33	0.31060	0.00054	14.825	736.69	0.32	0.33728	0.00057
14.824	809.47	0.33	0.31712	0.00054	15.223	744.22	0.32	0.34284	0.00057
15.222	814.63	0.33	0.32358	0.00053	15.621	751.38	0.32	0.34845	0.00056
15.621	819.41	0.34	0.33010	0.00053	16.019	757.99	0.32	0.35421	0.00056
16.019	824.04	0.34	0.33661	0.00053	16.417	764.43	0.32	0.35996	0.00055
16.417	828.60	0.34	0.34308	0.00053	16.815	770.37	0.32	0.36584	0.00055
16.815	832.78	0.34	0.34964	0.00053	17.213	776.05	0.33	0.37177	0.00055
17.213	836.97	0.34	0.35613	0.00052	17.611	781.54	0.33	0.37769	0.00055
17.611	840.97	0.34	0.36263	0.00052	18.009	786.73	0.33	0.38368	0.00054
18.009	844.76	0.34	0.36917	0.00052	18.408	791.76	0.33	0.38967	0.00054
18.407	848.49	0.34	0.37567	0.00052	18.806	796.72	0.33	0.39562	0.00054
18.806	852.09	0.34	0.38217	0.00052	19.204	801.33	0.33	0.40167	0.00054
19.204	855.58	0.34	0.38867	0.00052	19.602	805.81	0.33	0.40772	0.00054
19.602	859.08	0.35	0.39511	0.00052	20.000	810.14	0.33	0.41378	0.00053
20.000	862.31	0.35	0.40163	0.00052					
***T*= 333.15±0.01 K**					***T*= 353.15±0.02 K**				
0.133	1.77	0.21	1.18	0.25	0.131	1.69	0.21	1.12	0.25
0.691	10.82	0.22	1.006	0.040	0.689	10.17	0.22	0.987	0.040
1.289	21.00	0.22	0.967	0.021	1.287	19.58	0.22	0.957	0.021
1.887	31.55	0.22	0.942	0.014	1.885	29.30	0.22	0.937	0.014
2.485	42.60	0.22	0.919	0.010	2.483	39.38	0.22	0.919	0.010
3.084	54.23	0.22	0.8959	0.0079	3.082	49.86	0.22	0.9004	0.0081

3.682	66.45	0.22	0.8729	0.0064	3.680	60.77	0.22	0.8822	0.0066
4.280	79.34	0.22	0.8499	0.0053	4.278	72.08	0.22	0.8646	0.0055
4.878	93.06	0.22	0.8258	0.0045	4.876	83.96	0.22	0.8460	0.0047
5.476	107.72	0.22	0.8009	0.0039	5.474	96.30	0.22	0.8281	0.0041
6.074	123.56	0.22	0.7745	0.0034	6.073	109.29	0.22	0.8094	0.0036
6.672	140.52	0.23	0.7481	0.0030	6.671	122.86	0.22	0.7910	0.0032
7.271	158.94	0.23	0.7207	0.0026	7.269	137.12	0.22	0.7722	0.0029
7.869	179.12	0.23	0.6921	0.0023	7.867	152.18	0.23	0.7531	0.0026
8.467	201.29	0.23	0.6627	0.0020	8.465	168.06	0.23	0.7338	0.0023
9.065	226.03	0.23	0.6318	0.0018	9.064	184.94	0.23	0.7139	0.0021
9.663	253.70	0.23	0.6001	0.0016	9.662	202.69	0.23	0.6944	0.0019
10.261	284.79	0.23	0.5676	0.0014	10.260	221.34	0.23	0.6753	0.0017
10.859	319.08	0.24	0.5362	0.0013	10.858	241.15	0.23	0.6559	0.0016
11.458	356.87	0.24	0.5058	0.0011	11.456	261.91	0.23	0.6372	0.0015
12.056	395.80	0.25	0.4799	0.0010	12.054	283.75	0.24	0.6189	0.0013
12.654	434.60	0.25	0.45870	0.00092	12.653	306.51	0.24	0.6013	0.0012
13.252	470.88	0.26	0.44338	0.00085	13.251	329.93	0.24	0.5851	0.0012
13.850	503.66	0.27	0.43323	0.00079	13.849	353.82	0.24	0.5702	0.0011
14.448	532.36	0.27	0.42757	0.00075	14.447	377.85	0.25	0.5570	0.0010
15.046	558.23	0.28	0.42464	0.00072	15.045	401.59	0.25	0.54575	0.00094
15.645	581.00	0.28	0.42422	0.00069	15.644	424.91	0.25	0.53631	0.00089
16.243	600.99	0.29	0.42579	0.00067	16.242	447.21	0.26	0.52905	0.00085
16.841	618.68	0.29	0.42885	0.00065	16.840	468.57	0.26	0.52353	0.00081
17.439	634.63	0.29	0.43292	0.00064	17.438	488.90	0.27	0.51959	0.00078
18.037	649.19	0.30	0.43772	0.00063	18.036	508.00	0.27	0.51721	0.00075
18.635	662.58	0.30	0.44310	0.00061	18.634	525.80	0.27	0.51627	0.00073
19.233	674.77	0.30	0.44905	0.00061	19.233	542.52	0.28	0.51643	0.00071
19.832	686.11	0.30	0.45537	0.00060	19.831	558.06	0.28	0.51766	0.00069
20.430	696.50	0.31	0.46211	0.00059	20.429	572.56	0.28	0.51977	0.00067
21.028	706.28	0.31	0.46905	0.00058	21.027	586.25	0.28	0.52249	0.00066
21.626	715.50	0.31	0.47618	0.00058	21.625	599.12	0.29	0.52582	0.00065
22.224	724.01	0.31	0.48359	0.00058	22.224	611.22	0.29	0.52966	0.00064
22.822	732.18	0.32	0.49107	0.00057	22.822	622.56	0.29	0.53401	0.00063
23.420	739.95	0.32	0.49864	0.00057	23.420	633.01	0.29	0.53896	0.00062
24.019	747.27	0.32	0.50637	0.00056	24.018	643.19	0.30	0.54398	0.00061
24.617	754.35	0.32	0.51411	0.00056	24.616	652.58	0.30	0.54951	0.00061
25.215	760.88	0.32	0.52209	0.00056	25.215	661.85	0.30	0.55497	0.00060
25.813	767.38	0.32	0.52994	0.00056	25.813	670.74	0.30	0.56061	0.00060
26.411	773.49	0.32	0.53794	0.00055	26.411	679.08	0.30	0.56655	0.00059
27.009	779.33	0.33	0.54600	0.00055	27.009	686.87	0.31	0.57282	0.00059
27.607	784.93	0.33	0.55411	0.00055	27.607	694.39	0.31	0.57917	0.00058
28.206	790.27	0.33	0.56229	0.00055	28.205	701.83	0.31	0.58544	0.00058
28.804	795.63	0.33	0.57035	0.00055	28.804	708.74	0.31	0.59203	0.00058
29.402	800.81	0.33	0.57842	0.00055	29.402	715.19	0.31	0.59888	0.00057
30.000	805.80	0.33	0.58654	0.00055	30.000	721.49	0.31	0.60572	0.00057

***T*= 373.18±0.02 K**									
0.115	1.22	0.20	1.33	0.35					
0.693	9.60	0.22	1.016	0.042					
1.291	18.42	0.22	0.986	0.022					
1.890	27.42	0.22	0.969	0.015					
2.488	36.80	0.22	0.951	0.011					
3.086	46.34	0.22	0.9364	0.0085					
3.684	56.22	0.22	0.9215	0.0070					
4.282	66.38	0.22	0.9072	0.0059					
4.880	76.91	0.22	0.8924	0.0051					
5.478	87.88	0.22	0.8767	0.0044					
6.076	99.12	0.22	0.8621	0.0039					
6.674	110.69	0.22	0.8481	0.0035					
7.272	122.61	0.22	0.8342	0.0032					
7.871	135.11	0.22	0.8193	0.0029					
8.469	147.94	0.23	0.8051	0.0026					
9.067	161.18	0.23	0.7912	0.0024					
9.665	174.92	0.23	0.7771	0.0022					
10.263	189.17	0.23	0.7630	0.0020					
10.861	203.87	0.23	0.7492	0.0019					
11.459	219.06	0.23	0.7357	0.0017					
12.057	234.70	0.23	0.7225	0.0016					
12.655	250.85	0.23	0.7095	0.0015					
13.253	267.26	0.23	0.6974	0.0014					
13.851	284.15	0.24	0.6856	0.0013					
14.450	301.21	0.24	0.6747	0.0013					
15.048	318.58	0.24	0.6643	0.0012					
15.646	335.86	0.24	0.6552	0.0011					
16.244	353.35	0.24	0.6465	0.0011					
16.842	370.61	0.25	0.6391	0.0010					
17.440	387.67	0.25	0.6327	0.0010					
18.038	404.83	0.25	0.62667	0.00093					
18.636	421.06	0.25	0.62248	0.00090					
19.234	437.22	0.26	0.61872	0.00087					
19.832	452.52	0.26	0.61639	0.00084					
20.430	467.73	0.26	0.61433	0.00081					
21.029	482.14	0.26	0.61341	0.00079					
21.627	495.82	0.27	0.61345	0.00077					
22.225	509.00	0.27	0.61409	0.00075					
22.823	521.75	0.27	0.61521	0.00074					
23.421	533.81	0.27	0.61707	0.00072					
24.019	545.55	0.28	0.61921	0.00071					
24.617	556.71	0.28	0.62190	0.00070					
25.215	567.30	0.28	0.62513	0.00069					

25.813	577.64	0.28	0.62849	0.00068					
26.411	587.53	0.28	0.63223	0.00067					
27.010	596.91	0.29	0.63639	0.00066					
27.608	606.06	0.29	0.64066	0.00065					
28.206	614.65	0.29	0.64539	0.00065					
28.804	623.10	0.29	0.65014	0.00064					
29.402	631.20	0.29	0.65512	0.00064					
30.000	638.92	0.30	0.66037	0.00063					

Combined standard uncertainties:

$u(T)$= 0.006 K; $u(p)$= 0.0020 MPa for p < 6 MPa; $u(p)$= 0.024 MPa for 6 MPa ≤ p ≤ 70 MPa

$u(x_{CO_2})$= 0.00024; $u(x_{O_2})$= 0.000030; $u(x_{SO_2})$= 0.0000023; $u(x_{CO})$= 0.0000043.

Table S5 (continued). Reduced $p\rho T$ experimental data for the CO_2 + O_2 + SO_2 + CO (Mix 1) and CO_2 + NO + SO_2 + CO (Mix 2) mixtures. Z: compressibility factor; $u(\rho)$, $u(Z)$: combined standard uncertainties.

**Mix 2: x_{CO_2} = 0.99592; x_{NO} = 0.001410; x_{SO_2} = 0.0009100; x_{CO} = 0.0017002**									
**p (MPa)**	**ρ (kg.m^{-3})**	**$u(\rho)$ (kg.m^{-3})**	**Z**	**$u(Z)$**	**p (MPa)**	**ρ (kg.m^{-3})**	**$u(\rho)$ (kg.m^{-3})**	**Z**	**$u(Z)$**
***T*= 263.18±0.05 K**					***T*= 273.15±0.01 K**				
0.100	1.79	0.21	1.12	0.14	0.100	1.74	0.21	1.11	0.13836
0.326	6.52	0.22	1.006	0.035	0.394	7.79	0.22	0.981	0.02865
0.552	11.46	0.22	0.968	0.019	0.689	13.93	0.22	0.958	0.01567
0.778	16.54	0.22	0.946	0.013	0.983	20.39	0.22	0.934	0.01047
1.023	22.29	0.22	0.9229	0.0095	1.277	27.12	0.22	0.9122	0.00770
1.268	28.25	0.22	0.9023	0.0073	1.572	34.24	0.22	0.8889	0.00595
1.513	34.64	0.23	0.8780	0.0058	1.866	41.83	0.23	0.8639	0.00475
1.739	40.86	0.23	0.8555	0.0048	2.160	49.99	0.23	0.8370	0.00386
1.966	47.53	0.23	0.8311	0.0040	2.455	58.69	0.23	0.8099	0.00319
2.192	54.65	0.23	0.8061	0.0034	2.749	68.14	0.23	0.7812	0.00266
2.437	62.98	0.23	0.7776	0.0029	3.044	78.65	0.23	0.7493	0.00222
2.625	70.01	0.23	0.7536	0.0025	3.338	90.46	0.23	0.7145	0.00185
***T*= 263.15±0.01 K**					3.467	96.27	0.23	0.6973	0.00170
2.825	981.54	0.38	0.05784	0.00049	***T*= 273.17±0.01 K**				
3.283	984.20	0.38	0.06705	0.00049	3.714	927.02	0.36	0.07758	0.00050
3.741	986.85	0.38	0.07620	0.00049	4.170	931.24	0.36	0.08670	0.00050
4.199	989.50	0.38	0.08530	0.00049	4.626	935.08	0.37	0.09578	0.00050
4.658	992.20	0.38	0.09436	0.00049	5.081	938.59	0.37	0.10483	0.00050
5.116	994.73	0.38	0.10338	0.00049	5.537	942.10	0.37	0.11380	0.00050
5.574	997.26	0.38	0.11235	0.00049	5.993	945.70	0.37	0.12270	0.00049
6.033	999.66	0.38	0.12130	0.00049	6.448	948.98	0.37	0.13157	0.00049
6.491	1002.06	0.38	0.13020	0.00049	6.904	952.17	0.37	0.14040	0.00049
6.949	1004.35	0.38	0.13907	0.00048	7.360	955.30	0.37	0.14917	0.00049
7.407	1006.62	0.38	0.14791	0.00048	7.815	958.34	0.37	0.15791	0.00049
7.866	1008.86	0.38	0.15671	0.00048	8.271	961.31	0.37	0.16660	0.00049
8.324	1011.03	0.38	0.16549	0.00048	8.727	964.18	0.37	0.17525	0.00049
8.782	1013.24	0.38	0.17422	0.00048	9.182	966.95	0.37	0.18387	0.00049
9.240	1015.34	0.39	0.18293	0.00048	9.638	969.66	0.37	0.19246	0.00049
9.699	1017.43	0.39	0.19161	0.00048	10.094	972.23	0.37	0.20102	0.00049
10.157	1019.45	0.39	0.20026	0.00048	10.550	974.85	0.38	0.20954	0.00049
10.615	1021.43	0.39	0.20889	0.00048	11.005	977.38	0.38	0.21802	0.00049
11.074	1023.42	0.39	0.21749	0.00048	11.461	979.87	0.38	0.22647	0.00049
11.532	1025.31	0.39	0.22607	0.00048	11.917	982.27	0.38	0.23490	0.00049

11.990	1027.23	0.39	0.23461	0.00048	12.372	984.62	0.38	0.24330	0.00048
12.448	1029.05	0.39	0.24315	0.00048	12.828	986.90	0.38	0.25168	0.00048
12.907	1030.89	0.39	0.25165	0.00048	13.284	989.15	0.38	0.26003	0.00048
13.365	1032.69	0.39	0.26014	0.00048	13.739	991.39	0.38	0.26834	0.00048
13.823	1034.46	0.39	0.26860	0.00048	14.195	993.53	0.38	0.27664	0.00048
14.282	1036.19	0.39	0.27704	0.00048	14.651	995.67	0.38	0.28491	0.00048
14.740	1037.94	0.39	0.28544	0.00048	15.106	997.78	0.38	0.29315	0.00048
15.198	1039.63	0.39	0.29384	0.00048	15.562	999.83	0.38	0.30137	0.00048
15.656	1041.28	0.39	0.30222	0.00048	16.018	1001.85	0.38	0.30957	0.00048
16.115	1042.95	0.39	0.31057	0.00048	16.473	1003.84	0.38	0.31775	0.00049
16.573	1044.60	0.39	0.31890	0.00048	16.929	1005.73	0.38	0.32592	0.00049
17.031	1046.22	0.39	0.32721	0.00048	17.385	1007.65	0.38	0.33406	0.00049
17.489	1047.81	0.39	0.33550	0.00048	17.840	1009.49	0.38	0.34219	0.00049
17.948	1049.38	0.39	0.34378	0.00049	18.296	1011.40	0.38	0.35027	0.00049
18.406	1050.94	0.39	0.35203	0.00049	18.752	1013.32	0.38	0.35831	0.00049
18.864	1052.47	0.39	0.36027	0.00049	19.208	1015.16	0.38	0.36635	0.00049
19.323	1054.01	0.39	0.36849	0.00049	19.663	1016.87	0.39	0.37441	0.00049
19.781	1055.44	0.40	0.37671	0.00049	20.000	1018.19	0.39	0.38033	0.00018
20.000	1056.15	0.40	0.38063	0.00049					
***T*= 283.20±0.01 K**					***T*= 293.14±0.01 K**				
0.100	1.84	0.22	1.02	0.12	0.100	1.76	0.22	1.03	0.13
0.437	8.31	0.22	0.982	0.027	0.474	8.67	0.22	0.986	0.026
0.774	15.09	0.22	0.958	0.014	0.847	15.94	0.22	0.959	0.014
1.111	22.16	0.22	0.9367	0.0096	1.221	23.49	0.22	0.9375	0.0091
1.448	29.62	0.22	0.9132	0.0070	1.594	31.47	0.22	0.9139	0.0066
1.785	37.50	0.22	0.8892	0.0054	1.968	39.87	0.22	0.8905	0.0051
2.122	46.03	0.23	0.8612	0.0043	2.341	48.75	0.22	0.8665	0.0041
2.459	54.96	0.23	0.8358	0.0035	2.715	58.22	0.22	0.8413	0.0033
2.796	64.65	0.23	0.8079	0.0029	3.088	68.40	0.23	0.8147	0.0027
3.133	75.09	0.23	0.7794	0.0024	3.462	79.43	0.23	0.7864	0.0023
3.470	86.60	0.23	0.7485	0.0020	3.836	91.42	0.23	0.7570	0.0019
3.807	99.55	0.23	0.7144	0.0017	4.209	104.69	0.23	0.7255	0.0016
4.144	114.44	0.23	0.6764	0.0014	4.583	119.64	0.23	0.6911	0.0014
4.507	134.14	0.23	0.6276	0.0011	4.956	136.99	0.23	0.6528	0.0011
***T*= 283.18±0.01 K**					5.349	159.38	0.23	0.60561	0.00091
4.701	861.34	0.35	0.10194	0.00052	5.743	190.17	0.21	0.54485	0.00065
5.137	868.21	0.35	0.11051	0.00052	***T*= 293.17±0.01 K**				
5.573	874.28	0.35	0.11906	0.00052	5.888	773.29	0.33	0.13738	0.00056
6.009	879.88	0.35	0.12756	0.00051	6.306	787.25	0.33	0.14452	0.00055
6.445	885.09	0.35	0.13601	0.00051	6.724	798.94	0.33	0.15185	0.00055
6.881	889.95	0.36	0.14441	0.00051	7.142	808.50	0.34	0.15938	0.00054
7.317	894.52	0.36	0.15278	0.00051	7.560	816.67	0.34	0.16702	0.00054
7.753	898.88	0.36	0.16110	0.00050	7.978	823.98	0.34	0.17469	0.00053
8.189	903.04	0.36	0.16938	0.00050	8.396	830.82	0.34	0.18233	0.00053
8.625	907.06	0.36	0.17760	0.00050	8.814	837.18	0.34	0.18995	0.00053

9.061	910.92	0.36	0.18579	0.00050	9.232	843.03	0.34	0.19758	0.00052
9.497	914.55	0.36	0.19396	0.00050	9.650	848.53	0.34	0.20518	0.00052
9.933	918.16	0.36	0.20206	0.00050	10.068	853.70	0.35	0.21277	0.00052
10.369	921.57	0.36	0.21015	0.00050	10.486	858.64	0.35	0.22033	0.00052
10.805	924.79	0.36	0.21823	0.00050	10.904	863.35	0.35	0.22786	0.00051
11.241	928.04	0.36	0.22624	0.00049	11.322	868.00	0.35	0.23533	0.00051
11.677	931.21	0.36	0.23421	0.00049	11.740	872.35	0.35	0.24280	0.00051
12.113	934.24	0.37	0.24217	0.00049	12.158	876.44	0.35	0.25027	0.00051
12.549	937.10	0.37	0.25012	0.00049	12.576	880.28	0.35	0.25775	0.00051
12.985	939.96	0.37	0.25802	0.00049	12.994	884.12	0.35	0.26516	0.00051
13.421	942.74	0.37	0.26590	0.00049	13.412	887.64	0.35	0.27260	0.00050
13.857	945.47	0.37	0.27374	0.00049	13.830	891.11	0.35	0.28000	0.00050
14.293	948.10	0.37	0.28157	0.00049	14.248	894.60	0.36	0.28734	0.00050
14.729	950.71	0.37	0.28937	0.00049	14.666	897.95	0.36	0.29467	0.00050
15.165	953.33	0.37	0.29712	0.00049	15.084	901.23	0.36	0.30196	0.00050
15.601	955.73	0.37	0.30489	0.00049	15.502	904.46	0.36	0.30922	0.00050
16.036	958.18	0.37	0.31261	0.00049	15.920	907.59	0.36	0.31646	0.00050
16.472	960.63	0.37	0.32029	0.00049	16.338	910.59	0.36	0.32370	0.00050
16.908	962.97	0.37	0.32797	0.00049	16.756	913.57	0.36	0.33090	0.00050
17.344	965.27	0.37	0.33562	0.00049	17.174	916.42	0.36	0.33810	0.00050
17.780	967.52	0.37	0.34326	0.00049	17.592	919.20	0.36	0.34528	0.00050
18.216	969.67	0.37	0.35089	0.00049	18.010	921.91	0.36	0.35244	0.00050
18.652	971.87	0.37	0.35848	0.00049	18.428	924.62	0.36	0.35957	0.00050
19.088	973.99	0.37	0.36606	0.00049	18.846	927.21	0.36	0.36670	0.00050
19.544	976.17	0.38	0.37397	0.00049	19.264	929.72	0.36	0.37382	0.00050
20.000	978.28	0.38	0.38186	0.00018	19.682	932.14	0.36	0.38093	0.00050
					20.000	933.99	0.36	0.38633	0.00050
***T*= 303.19±0.02 K**					***T*= 313.14±0.02 K**				
0.122	2.05	0.21	1.04	0.23	0.111	1.73	0.22	1.08	0.27
0.499	8.75	0.22	0.995	0.054	0.489	8.28	0.22	0.998	0.056
0.896	16.18	0.22	0.967	0.029	0.887	15.44	0.22	0.970	0.030
1.293	23.93	0.22	0.943	0.020	1.285	22.88	0.22	0.949	0.020
1.691	32.11	0.22	0.919	0.015	1.683	30.60	0.22	0.929	0.015
2.088	40.59	0.22	0.897	0.011	2.082	38.68	0.22	0.909	0.012
2.485	49.55	0.22	0.8749	0.0093	2.480	47.05	0.22	0.890	0.010
2.882	59.04	0.22	0.8516	0.0078	2.878	55.94	0.22	0.8691	0.0080
3.279	69.09	0.23	0.8280	0.0066	3.276	65.20	0.22	0.8488	0.0069
3.676	79.90	0.23	0.8027	0.0057	3.674	75.04	0.23	0.8272	0.0059
4.073	91.53	0.23	0.7764	0.0050	4.073	85.46	0.23	0.8050	0.0052
4.470	104.20	0.23	0.7484	0.0043	4.471	96.62	0.23	0.7816	0.0046
4.867	118.14	0.23	0.7188	0.0038	4.869	108.59	0.23	0.7574	0.0041
5.264	133.67	0.23	0.6871	0.0033	5.267	121.64	0.23	0.7314	0.0036
5.661	151.46	0.23	0.6521	0.0029	5.665	135.92	0.23	0.7040	0.0032
6.058	172.40	0.23	0.6131	0.0026	6.064	151.57	0.23	0.6757	0.0029
6.455	198.24	0.23	0.5681	0.0022	6.462	169.19	0.23	0.6451	0.0026

6.693	217.70	0.23	0.5364	0.0020	6.860	189.54	0.23	0.6113	0.0023
8.014	698.65	0.31	0.20014	0.00061	7.258	214.36	0.23	0.5719	0.0020
8.140	707.07	0.31	0.20086	0.00060	7.656	243.54	0.24	0.5310	0.0017
8.500	723.98	0.32	0.20483	0.00059	8.054	283.53	0.24	0.4798	0.0015
8.859	737.44	0.32	0.20960	0.00058	8.453	343.54	0.25	0.4156	0.0012
9.218	748.79	0.32	0.21479	0.00057	8.851	443.00	0.26	0.33748	0.00094
9.578	758.95	0.32	0.22018	0.00056	9.249	536.32	0.28	0.29130	0.00077
9.937	767.75	0.33	0.22582	0.00056	9.647	595.32	0.29	0.27373	0.00070
10.297	775.71	0.33	0.23159	0.00055	10.045	629.91	0.30	0.26937	0.00066
10.656	783.23	0.33	0.23737	0.00055	10.444	654.98	0.30	0.26933	0.00063
11.015	789.84	0.33	0.24332	0.00054	10.842	675.36	0.31	0.27116	0.00062
11.375	796.15	0.33	0.24927	0.00054	11.240	691.51	0.31	0.27456	0.00060
11.734	801.96	0.33	0.25528	0.00054	11.638	705.31	0.31	0.27872	0.00059
12.093	807.46	0.34	0.26131	0.00053	12.036	717.52	0.32	0.28335	0.00058
12.453	812.75	0.34	0.26733	0.00053	12.434	728.00	0.32	0.28851	0.00058
12.812	817.76	0.34	0.27335	0.00053	12.833	737.20	0.32	0.29404	0.00057
13.172	822.56	0.34	0.27938	0.00053	13.231	745.99	0.32	0.29959	0.00056
13.531	827.14	0.34	0.28542	0.00052	13.629	754.59	0.32	0.30508	0.00056
14.250	835.64	0.34	0.29752	0.00052	14.027	762.32	0.33	0.31081	0.00055
14.609	839.75	0.34	0.30353	0.00052	14.425	769.24	0.33	0.31676	0.00055
14.969	843.68	0.34	0.30955	0.00052	14.824	775.68	0.33	0.32280	0.00055
15.328	847.32	0.34	0.31562	0.00052	15.222	781.85	0.33	0.32886	0.00054
15.687	851.01	0.35	0.32162	0.00052	15.620	787.54	0.33	0.33502	0.00054
16.047	854.54	0.35	0.32763	0.00051	16.018	793.16	0.33	0.34113	0.00054
16.406	857.86	0.35	0.33367	0.00051	16.416	798.58	0.33	0.34724	0.00054
16.766	861.25	0.35	0.33964	0.00051	16.815	803.44	0.33	0.35351	0.00053
17.125	864.40	0.35	0.34565	0.00051	17.213	808.37	0.34	0.35967	0.00053
17.484	867.52	0.35	0.35164	0.00051	17.611	813.09	0.34	0.36586	0.00053
17.844	870.63	0.35	0.35758	0.00051	18.009	817.55	0.34	0.37209	0.00053
18.203	873.60	0.35	0.36355	0.00051	18.407	821.91	0.34	0.37830	0.00053
18.562	876.42	0.35	0.36953	0.00051	18.805	826.06	0.34	0.38454	0.00052
18.922	879.26	0.35	0.37547	0.00051	19.204	830.05	0.34	0.39079	0.00052
19.281	882.00	0.35	0.38141	0.00051	19.602	834.02	0.34	0.39700	0.00052
19.641	884.71	0.35	0.38733	0.00051	20.000	837.74	0.34	0.40326	0.00052
20.000	887.35	0.35	0.39324	0.00051					
***T*= 333.15±0.01 K**					***T*= 353.15±0.02 K**				
0.113	1.71	0.22	1.05	0.26	0.120	1.72	0.20	1.04	0.24
0.691	10.97	0.22	1.001	0.040	0.698	10.38	0.22	1.006	0.041
1.289	21.19	0.22	0.966	0.021	1.296	19.91	0.22	0.975	0.021
1.888	31.86	0.22	0.941	0.014	1.894	29.74	0.22	0.954	0.014
2.486	43.07	0.22	0.916	0.010	2.492	40.01	0.22	0.933	0.010
3.084	54.93	0.22	0.8913	0.0078	3.090	50.61	0.22	0.9143	0.0082
3.682	67.44	0.22	0.8668	0.0063	3.688	61.74	0.22	0.8946	0.0067
4.280	80.65	0.22	0.8426	0.0053	4.286	73.30	0.22	0.8756	0.0056
4.878	94.79	0.23	0.8171	0.0045	4.884	85.40	0.22	0.8565	0.0048

5.476	109.98	0.23	0.7906	0.0038	5.482	98.09	0.22	0.8370	0.0041
6.075	126.33	0.23	0.7634	0.0033	6.080	111.46	0.23	0.8169	0.0036
6.673	144.11	0.23	0.7351	0.0029	6.678	125.55	0.23	0.7965	0.0032
7.271	163.60	0.23	0.7056	0.0025	7.276	140.41	0.23	0.7760	0.0029
7.869	185.54	0.23	0.6734	0.0022	7.874	156.23	0.23	0.7548	0.0026
8.467	209.75	0.23	0.6409	0.0020	8.472	172.95	0.23	0.7336	0.0023
9.065	237.65	0.24	0.6056	0.0017	9.070	190.78	0.23	0.7120	0.0021
9.663	269.37	0.24	0.5696	0.0015	9.668	209.64	0.23	0.6907	0.0019
10.261	305.01	0.24	0.5341	0.0013	10.266	230.08	0.23	0.6682	0.0017
10.860	345.58	0.25	0.4989	0.0012	10.864	251.52	0.24	0.6469	0.0016
11.458	390.03	0.25	0.4664	0.0010	11.462	274.53	0.24	0.6253	0.0014
12.056	435.10	0.26	0.43992	0.00092	12.060	298.67	0.24	0.6047	0.0013
12.654	478.51	0.27	0.41986	0.00084	12.658	323.76	0.25	0.5855	0.0012
13.252	517.01	0.28	0.40696	0.00077	13.256	349.97	0.25	0.5673	0.0011
13.850	550.21	0.28	0.39967	0.00073	13.854	376.27	0.25	0.5514	0.0010
14.448	578.30	0.29	0.39667	0.00069	14.452	402.27	0.26	0.5380	0.0010
15.047	602.76	0.29	0.39633	0.00067	15.050	427.04	0.26	0.52781	0.00091
15.645	623.68	0.30	0.39826	0.00065	15.648	451.90	0.26	0.51859	0.00086
16.243	641.83	0.30	0.40180	0.00063	16.246	475.68	0.27	0.51149	0.00082
16.841	657.93	0.30	0.40640	0.00062	16.844	498.39	0.27	0.50616	0.00078
17.439	672.24	0.31	0.41187	0.00061	17.442	519.28	0.28	0.50304	0.00075
18.037	685.45	0.31	0.41779	0.00060	18.040	538.83	0.28	0.50141	0.00073
18.635	697.49	0.31	0.42419	0.00059	18.638	556.72	0.28	0.50139	0.00071
19.234	708.41	0.31	0.43106	0.00058	19.236	572.94	0.29	0.50282	0.00069
19.832	718.38	0.32	0.43830	0.00057	19.834	588.27	0.29	0.50494	0.00067
20.430	727.61	0.32	0.44579	0.00057	20.432	602.18	0.29	0.50816	0.00066
21.028	736.50	0.32	0.45330	0.00056	21.030	614.84	0.29	0.51226	0.00065
21.626	744.78	0.32	0.46101	0.00056	21.628	627.29	0.30	0.51637	0.00064
22.224	752.51	0.32	0.46890	0.00056	22.226	638.95	0.30	0.52096	0.00063
22.822	759.84	0.32	0.47687	0.00055	22.824	649.76	0.30	0.52608	0.00062
23.421	766.78	0.33	0.48494	0.00055	23.422	659.88	0.30	0.53158	0.00061
24.019	773.45	0.33	0.49304	0.00055	24.020	669.08	0.30	0.53766	0.00061
24.617	779.72	0.33	0.50126	0.00055	24.618	678.23	0.31	0.54361	0.00060
25.215	785.88	0.33	0.50941	0.00055	25.216	686.81	0.31	0.54986	0.00059
25.813	791.69	0.33	0.51767	0.00054	25.814	695.05	0.31	0.55623	0.00059
26.411	797.36	0.33	0.52590	0.00054	26.412	702.91	0.31	0.56275	0.00059
27.009	802.86	0.33	0.53412	0.00054	27.010	710.42	0.31	0.56941	0.00058
27.607	808.13	0.33	0.54239	0.00054	27.608	717.43	0.31	0.57632	0.00058
28.206	813.13	0.34	0.55073	0.00054	28.206	724.39	0.32	0.58316	0.00058
28.804	817.94	0.34	0.55910	0.00054	28.804	730.86	0.32	0.59024	0.00057
29.402	822.56	0.34	0.56751	0.00054	29.402	737.21	0.32	0.59731	0.00057
30.000	827.06	0.34	0.57590	0.00054	30.000	743.23	0.32	0.60452	0.00057
***T*= 373.15±0.02 K**									
0.105	1.42	0.22	1.05	0.29					
0.684	9.88	0.22	0.981	0.041					

1.282	18.76	0.22	0.968	0.021					
1.880	27.99	0.22	0.952	0.014					
2.479	37.41	0.22	0.939	0.011					
3.077	47.11	0.22	0.9257	0.0085					
3.675	57.10	0.22	0.9124	0.0069					
4.274	67.42	0.22	0.8985	0.0059					
4.872	78.25	0.22	0.8825	0.0050					
5.470	89.33	0.22	0.8680	0.0044					
6.068	100.88	0.23	0.8526	0.0039					
6.667	112.79	0.23	0.8378	0.0035					
7.265	125.14	0.23	0.8229	0.0031					
7.863	138.06	0.23	0.8073	0.0028					
8.462	151.36	0.23	0.7924	0.0026					
9.060	165.29	0.23	0.7769	0.0023					
9.658	179.59	0.23	0.7623	0.0021					
10.256	194.87	0.23	0.7460	0.0020					
10.855	210.35	0.23	0.7314	0.0018					
11.453	226.40	0.23	0.7170	0.0017					
12.051	243.07	0.24	0.7027	0.0016					
12.650	260.52	0.24	0.6882	0.0015					
13.248	278.30	0.24	0.6747	0.0014					
13.846	296.08	0.24	0.6628	0.0013					
14.444	314.31	0.24	0.6514	0.0012					
15.043	332.84	0.25	0.6406	0.0011					
15.641	351.43	0.25	0.6308	0.0011					
16.239	369.78	0.25	0.6225	0.0010					
16.838	387.96	0.26	0.6152	0.0010					
17.436	405.76	0.26	0.60907	0.00094					
18.034	423.08	0.26	0.60418	0.00090					
18.633	440.20	0.26	0.59995	0.00086					
19.231	457.05	0.26	0.59639	0.00083					
19.829	472.72	0.27	0.59456	0.00081					
20.427	488.39	0.27	0.59285	0.00078					
21.026	504.05	0.27	0.59124	0.00076					
21.624	518.86	0.28	0.59071	0.00074					
22.222	532.27	0.28	0.59177	0.00073					
22.821	544.83	0.28	0.59369	0.00071					
23.419	556.93	0.28	0.59602	0.00070					
24.017	568.73	0.28	0.59856	0.00069					
24.615	579.64	0.29	0.60193	0.00067					
25.214	590.32	0.29	0.60540	0.00067					
25.812	600.32	0.29	0.60944	0.00066					
26.410	609.94	0.29	0.61373	0.00065					
27.009	619.14	0.29	0.61831	0.00064					
27.607	628.16	0.30	0.62293	0.00064					

28.205	636.67	0.30	0.62792	0.00063					
28.803	644.84	0.30	0.63312	0.00062					
29.402	652.56	0.30	0.63863	0.00062					
30.000	660.06	0.30	0.64422	0.00062					

Combined standard uncertainties:

$u(T)$= 0.006 K; $u(p)$= 0.0020 MPa for p < 6 MPa; $u(p)$= 0.024 MPa for 6 MPa ≤ p ≤ 70 MPa

$u(x_{CO_2})$= 0.00025; $u(x_{NO})$= 0.000014; $u(x_{SO_2})$= 0.0000023; $u(x_{CO})$= 0.0000043.

Table S6. pcT experimental data for the CO_2 + CH_3OH + O_2 + SO_2 + CO and CO_2 + CH_3OH + NO + SO_2 + CO (doped Mix 1 and doped Mix 2) mixtures.

***p* (MPa)**	***c* (m.s^{-1})**	***p* (MPa)**	***c* (m.s^{-1})**	***p* (MPa)**	***c* (m.s^{-1})**	***p* (MPa)**	***c* (m.s^{-1})**
$x_{CO_2} = 0.95769; x_{CH_3OH} = 0.00999;\ x_{O_2} = 0.02974; x_{SO_2} = 0.000893;\ x_{CO} = 0.001686$							
***T*= 263.15±0.01 K**		***T*= 273.15±0.02 K**		***T*= 283.16±0.01 K**		***T*= 293.15±0.01 K**	
194.97	1407.06	174.46	1332.06	181.26	1322.09	194.60	1329.85
189.96	1396.01	169.96	1321.3	179.96	1318.99	189.96	1318.72
179.96	1373.48	167.06	1313.85	169.96	1294.66	179.96	1294.76
169.96	1350.10	159.96	1296.14	159.96	1269.44	169.96	1270.03
159.96	1325.87	149.96	1270.36	149.96	1243.06	159.96	1244.29
149.96	1300.66	139.96	1243.47	139.96	1215.54	149.96	1217.42
139.96	1274.41	129.96	1215.22	129.96	1186.67	139.96	1189.35
129.96	1246.95	119.96	1185.6	119.96	1156.24	129.96	1159.76
119.96	1218.20	109.96	1154.41	109.96	1124.22	119.96	1128.75
109.96	1187.89	99.96	1121.22	99.96	1090.13	109.96	1095.83
99.96	1155.87	94.96	1103.83	94.96	1072.21	99.96	1060.83
89.96	1121.83	89.96	1085.89	89.96	1053.60	94.96	1042.43
79.96	1085.36	84.96	1067.28	84.96	1034.39	89.96	1023.33
69.96	1046.12	79.96	1047.94	79.96	1014.41	84.96	1003.50
59.96	1003.35	74.96	1027.77	74.96	993.54	79.96	982.88
49.98	956.12	69.96	1006.75	69.96	971.70	74.96	961.38
39.98	903.15	64.96	984.82	64.96	948.90	69.96	938.76
29.98	842.08	59.96	961.81	59.96	924.81	64.96	914.91
19.97	768.52	54.96	937.48	54.96	899.34	59.96	889.87
10.04	673.23	49.96	911.7	49.96	872.32	54.96	863.35
		44.96	884.33	45.02	843.59	49.96	835.09
		40.04	855.15	39.99	812.25	44.96	804.62
		35.01	823.41	34.95	777.86	39.96	771.66
		29.97	788.51	31.05	748.64	35.00	735.77
						32.96	719.74

Standard uncertainties:

$u(T) = 0.015$ K; $u(p) = 0.02$ MPa; $u^*(c) = 8.9 \times 10^{-4}c$; $u(c) = 8.9 \times 10^{-4}c$;
$u(x_{CO_2}) =$ 0.00058; $u(x_{CH_3OH}) =$ 0.00011; $u(x_{O_2}) = 0.00047$; $u(x_{SO_2}) = 0.000015$;
$u(x_{CO}) = 0.000048$.

Table S6 (continued). pcT experimental data for the CO_2 + CH_3OH + O_2 + SO_2 + CO and CO_2 + CH_3OH + NO + SO_2 + CO (doped Mix 1 and doped Mix 2) mixtures.

p **(MPa)**	***c*** **($m.s^{-1}$)**	***p*** **(MPa)**	***c*** **($m.s^{-1}$)**	***p*** **(MPa)**	***c*** **($m.s^{-1}$)**	***p*** **(MPa)**	***c*** **($m.s^{-1}$)**
**Doped Mix 1:** $x_{CO_2} = 0.95769; x_{CH_3OH} = 0.00999;\ x_{O_2} = 0.02974; x_{SO_2} = 0.000893;\ x_{CO} = 0.001686$							
***T*= 303.15±0.01 K**		***T*= 313.16±0.01 K**		***T*= 333.15±0.01 K**		***T*= 353.15±0.00 K**	
189.96	1293.98	189.97	1272.91	194.96	1241.85	169.96	1136.80
179.96	1269.81	179.96	1248.30	189.96	1229.57	159.96	1108.82
169.96	1244.60	169.96	1222.76	179.96	1204.33	149.96	1079.52
159.96	1218.38	159.96	1196.14	169.96	1178.06	144.97	1064.31
149.96	1191.04	149.96	1168.32	159.96	1150.70	139.96	1048.76
139.96	1162.35	139.96	1139.18	149.96	1122.05	134.96	1032.79
129.96	1132.24	129.96	1108.59	139.96	1092.02	129.96	1016.35
119.96	1100.50	119.96	1076.17	134.96	1076.38	124.96	999.41
109.96	1066.88	109.96	1041.85	129.96	1060.45	119.96	981.95
99.96	1030.92	99.96	1005.21	124.96	1043.88	114.96	963.99
89.96	992.46	94.96	985.89	119.96	1026.96	109.96	945.46
94.96	1011.98	89.96	965.85	114.96	1009.48	104.96	926.21
84.96	972.10	84.96	945.02	109.96	991.36	99.96	906.39
79.96	950.80	79.96	923.13	104.96	972.76	94.96	885.65
74.96	928.60	74.96	900.37	99.96	953.34	89.96	864.09
69.96	905.35	69.96	876.37	94.96	933.30	84.96	841.61
64.96	880.69	64.96	851.09	89.96	912.41	79.96	818.10
59.96	854.61	59.96	824.01	84.96	890.58	74.97	793.37
54.96	827.10	54.96	795.58	79.96	867.74		
49.96	797.55	49.96	764.89	74.96	843.92		
		44.96	731.66	69.96	818.72		
				64.96	792.12		
				59.96	763.61		
				57.97	751.81		

Standard uncertainties:

$u(T) = 0.015$ K, $u(p) = 0.02$ MPa, $u^*(c) = 8.9 \times 10^{-4}c$, $u(c) = 8.9 \times 10^{-4}c$.

$u(x_{CO_2}) = 0.00058$; $u(x_{CH_3OH}) = 0.00011$; $u(x_{O_2}) = 0.00047$; $u(x_{SO_2}) = 0.000015$; $u(x_{CO}) = 0.000048$.

Table S6 (Continued). pcT experimental data for the CO_2 + CH_3OH + O_2 + SO_2 + CO and CO_2 + CH_3OH + NO + SO_2 + CO (doped Mix 1 and doped Mix 2) mixtures.

p **(MPa)**	***c*** **(m.s^{-1})**						
**Doped Mix 1:** $\boldsymbol{x_{CO_2} = 0.95769; x_{CH_3OH} = 0.00999;\ x_{O_2} = 0.02974; x_{SO_2} = 0.000893;\ x_{CO} = 0.001686}$							
T **= 373.14±0.01 K**							
189.97	1152.72						
179.96	1126.53						
169.96	1099.24						
159.96	1070.72						
149.96	1040.95						
144.96	1025.44						
139.96	1009.59						
134.96	993.32						
129.96	976.52						
124.96	959.29						
119.96	941.59						
114.96	923.26						
109.96	904.37						
104.96	884.71						
99.96	864.37						
95.96	847.54						

Standard uncertainties:

$u(T) = 0.015$ K, $u(p) = 0.02$ MPa, $u^*(c) = 8.9 \times 10^{-4}c,\ u(c) = 8.9 \times 10^{-4}c$.

$u(x_{CO_2}) =\ 0.00058$; $u(x_{CH_3OH})$= 0.00011; $u(x_{O_2}) = 0.00047$; $u(x_{SO_2}) = 0.000015$;

$u(x_{CO}) = 0.000048$.

Table S6 (continued). pcT experimental data for the CO_2 + CH_3OH + O_2 + SO_2 + CO and CO_2 + CH_3OH + NO + SO_2 + CO (doped Mix 1 and doped Mix 2) mixtures.

p **(MPa)**	***c*** **(m.s^{-1})**	***p*** **(MPa)**	***c*** **(m.s^{-1})**	***p*** **(MPa)**	***c*** **(m.s^{-1})**	***p*** **(MPa)**	***c*** **(m.s^{-1})**
**Doped Mix 2:** $x_{CO_2} = 0.98586; x_{CH_3OH} = 0.01016; x_{NO} = 0.001396; x_{SO_2} = 0.000899; x_{CO} = 0.001683$							
***T*= 263.14±0.01 K**		***T*= 273.16±0.01 K**		***T*= 283.15±0.01 K**		***T*= 293.15±0.01 K**	
194.96	1422.51	194.96	1396.18	194.96	1370.17	189.96	1333.30
189.96	1411.65	189.96	1385.06	189.96	1358.89	179.96	1309.66
179.96	1389.38	179.96	1362.30	179.96	1335.70	169.96	1285.20
169.96	1366.21	169.96	1338.77	169.96	1311.61	159.96	1259.65
159.96	1342.30	159.96	1314.29	159.96	1286.66	149.96	1233.11
149.96	1317.40	149.96	1288.76	149.96	1260.58	139.96	1205.31
139.96	1291.45	139.96	1262.21	139.96	1233.37	129.96	1176.08
129.96	1264.34	129.96	1234.41	129.96	1204.92	119.96	1145.42
119.96	1236.00	119.96	1205.25	119.96	1174.93	109.96	1113.01
109.96	1206.14	109.96	1174.49	109.96	1143.33	99.96	1078.56
99.96	1174.62	99.96	1141.98	99.96	1109.77	89.96	1041.70
89.96	1141.17	94.96	1124.93	94.96	1092.23	84.96	1022.23
79.96	1105.45	89.96	1107.31	89.96	1074.11	79.96	1001.99
69.96	1067.01	84.96	1089.15	84.96	1055.20	74.96	980.91
64.96	1046.58	79.96	1070.26	79.96	1035.61	69.96	958.79
59.96	1025.22	74.96	1050.72	74.96	1015.24	64.96	935.60
54.96	1002.87	69.96	1030.22	69.96	993.96	59.96	911.16
49.97	979.32	64.96	1008.86	64.96	971.69	54.96	885.34
44.96	954.39	59.96	986.47	59.96	948.27	49.97	857.74
39.96	927.99	54.96	962.80	54.96	923.64	44.97	828.34
34.96	899.67	49.96	938.01	49.97	897.29	39.97	796.47
29.97	869.21	44.96	911.62	44.97	869.42	34.96	761.62
24.96	835.95	39.96	883.46	39.97	839.41	29.97	723.00
19.96	799.24	34.96	853.13	34.96	806.80		
14.95	757.83	29.96	820.05	29.97	771.13		
9.96	710.03	24.96	783.70	24.96	731.21		

		19.96	742.83				
		14.97	696.00				

Combined standard uncertainties:

$u(T) = 0.015$ K; $u(p) = 0.02$ MPa; $u^{*}(c) = 4.6 \times 10^{-4}c;$ $u(c) = 4.6 \times 10^{-4}c;$
$u(x_{CO_2}) = 0.00016;$ $u(x_{CH_3OH}) = 0.00011;$ $u(x_{NO}) = 0.000026;$ $u(x_{SO_2}) = 0.000015;$
$u(x_{CO}) = 0.000027.$

Table S6 (continued). pcT experimental data for the CO_2 + CH_3OH + O_2 + SO_2 + CO and CO_2 + CH_3OH + NO + SO_2 + CO (doped Mix 1 and doped Mix 2) mixtures.

p **(MPa)**	***c*** **($m.s^{-1}$)**	***p*** **(MPa)**	***c*** **($m.s^{-1}$)**	***p*** **(MPa)**	***c*** **($m.s^{-1}$)**	***p*** **(MPa)**	***c*** **($m.s^{-1}$)**
**Doped Mix 2:** $x_{CO_2} = 0.98586; x_{CH_3OH} = 0.01016; x_{NO} = 0.001396; x_{SO_2} = 0.000899; x_{CO} = 0.001683$							
***T*= 303.15±0.01 K**		***T*= 313.15±0.01 K**		***T*= 333.15±0.01 K**		***T*= 353.14±0.01 K**	
194.96	1320.27	189.96	1284.71	194.96	1251.87	194.96	1210.87
189.96	1308.58	179.96	1260.25	189.96	1239.69	189.96	1198.41
179.96	1284.58	169.96	1234.85	179.96	1214.62	179.96	1172.74
169.96	1259.65	159.96	1208.41	169.96	1188.45	169.96	1146.02
159.96	1233.62	149.96	1180.81	159.96	1161.23	159.96	1118.20
149.96	1206.47	139.96	1151.92	149.96	1132.77	149.96	1089.01
139.96	1178.12	129.96	1121.62	144.96	1118.04	144.96	1073.86
129.96	1148.36	119.96	1089.55	139.96	1102.94	139.96	1058.35
119.96	1116.96	109.96	1055.72	134.96	1087.38	134.96	1042.46
109.96	1083.73	99.96	1019.47	129.96	1071.52	129.96	1026.08
99.96	1048.39	94.96	1000.37	124.96	1055.15	124.96	1009.26
94.96	1029.77	89.96	980.54	119.96	1038.32	119.96	991.94
89.96	1010.49	84.96	959.90	114.96	1020.97	114.96	974.11
84.96	990.44	79.96	938.44	109.96	1003.03	109.96	955.64
79.96	969.54	74.96	915.91	104.96	984.51	104.96	936.50
74.96	947.70	69.96	892.30	99.96	965.31	99.96	916.70
69.96	924.81	64.96	867.41	94.96	945.39	94.96	896.16
64.96	900.78	59.96	841.11	89.96	924.72	89.96	874.69
59.96	875.37	54.96	812.94	84.96	903.15	84.96	852.38
54.96	848.32	49.96	782.94	79.96	880.64	79.96	828.99
49.96	819.56	44.96	750.42	74.96	856.92		
44.96	788.55			69.96	831.98		
39.96	754.86			66.46	813.78		

Combined standard uncertainties:

$u(T) = 0.015$ K; $u(p) = 0.02$ MPa; $u^*(c) = 4.6 \times 10^{-4}c$; $u(c) = 4.6 \times 10^{-4}c$;

$u(x_{CO_2}) = 0.00016$; $u(x_{CH_3OH}) = 0.00011$; $u(x_{NO}) = 0.000026$; $u(x_{SO_2}) = 0.000015$; $u(x_{CO}) = 0.000027$.

Table S6 (continued). pcT experimental data for the CO_2 + CH_3OH + O_2 + SO_2 + CO and CO_2 + CH_3OH + NO + SO_2 + CO (doped Mix 1 and doped Mix 2) mixtures.

p **(MPa)**	***c*** **(m.s^{-1})**						
**Doped Mix 2:** $x_{CO_2} = 0.98586; x_{CH_3OH} = 0.01016; x_{NO} = 0.001396; x_{SO_2} = 0.000899; x_{CO} = 0.001683$							
***T*= 373.15±0.01 K**							
194.96	1173.50						
189.96	1160.79						
179.96	1134.62						
174.96	1121.19						
169.96	1107.46						
164.96	1093.40						
159.96	1079.02						
154.96	1064.33						
149.96	1049.28						
144.96	1033.86						
139.96	1018.07						
134.96	1001.82						
129.96	985.12						
124.96	967.93						
119.96	950.27						
114.96	932.01						
109.96	913.14						
104.97	893.62						

Combined standard uncertainties:
$u(T) = 0.015$ K; $u(p) = 0.02$ MPa; $u^*(c) = 4.6 \times 10^{-4}c; u(c) = 4.6 \times 10^{-4}c$;
$u(x_{CO_2}) = 0.00016$; $u(x_{CH_3OH}) = 0.00011$; $u(x_{NO}) = 0.000026$; $u(x_{SO_2}) = 0.000015$; $u(x_{CO}) = 0.000027$.

Table S7. Values of $p^{\#}$ and the a_i coefficients in equation (2) in the correlation of the experimental speed of sound, c, as a function of pressure, p, in the mixtures CO_2 + CH_3OH + O_2 + SO_2 +CO (doped Mix 1) and CO_2 + CH_3OH + O_2 + SO_2 + CO (doped Mix 2) with composition x at temperatures T, as well as the mean relative deviations.

**Doped Mix 1:** x_{CO_2} x_{CH_3OH} x_{O_2} x_{SO_2} x_{CO}	T/K	$p^{\#}$ (MPa)	$10 \times a_1$ (MPa·m^{-1}·s)	$10^4 \times a_2$ (MPa·m^{-2}·s^2)	$10^8 \times a_3$ (MPa·m^{-3}·s^3)	MRD_c (%)
0.95769 0.00999 0.02974 0.000893 0.001686	263.15	50	2.00848	2.32949	7.78587	0.003
	273.15	60	2.11425	2.34875	7.99256	0.008
	283.16	60	2.02102	2.28222	7.97486	0.005
	293.15	60	1.94138	2.21485	7.66894	0.008
	303.15	70	2.09175	2.26649	7.65450	0.005
	313.16	70	2.03083	2.20896	7.64049	0.005
	333.15	70	1.93085	2.09233	7.69179	0.006
	353.15	90	2.27387	2.21109	7.72984	0.002
	373.14	100	2.41854	2.22124	7.73510	0.004
	Overall mean relative deviation $\overline{\overline{MRD_c}}$ = 0.005 %					
**Doped Mix 2:** x_{CO_2} x_{CH_3OH} x_{NO} x_{SO_2} x_{CO}	T/K	$p^{\#}$ (MPa)	$10 \times a_1$ (MPa·m^{-1}·s)	$10^4 \times a_2$ (MPa·m^{-2}·s^2)	$10^8 \times a_3$ (MPa·m^{-3}·s^3)	MRD_c (%)
0.98586 0.01016 0.001396 0.000899 0.001683	263.14	30	1.57457	2.11304	7.81116	0.002
	273.16	35	1.58282	2.08726	7.82857	0.003
	283.15	45	1.72984	2.14495	7.70365	0.007
	293.15	50	1.75842	2.13648	7.58720	0.006
	303.15	70	2.13417	2.30165	7.54940	0.004
	313.15	70	2.06470	2.23931	7.55020	0.003
	333.15	90	2.36779	2.33022	7.49226	0.002
	353.14	90	2.28776	2.23098	7.67126	0.001
	373.15	115	2.69670	2.36809	7.71750	0.001
	Overall mean relative deviation $\overline{\overline{MRD_c}}$ = 0.003 %					

$MRD_c = \frac{100}{N}\sum_i^N \left|\frac{c_i - c_{i,fit}}{c_i}\right|$; N: number of experimental points at each composition and temperature.

Table S8. pcT extrapolated values for the CO_2 + CH_3OH + O_2 + SO_2 + CO and CO_2 + CH_3OH + NO + SO_2 + CO (doped Mix 1 and doped Mix 2) mixtures, using $p^{\#}$ and the coefficients from Table S7.

p **(MPa)**	***c*** **($m \cdot s^{-1}$)**	***p*** **(MPa)**	***c*** **($m \cdot s^{-1}$)**	***p*** **(MPa)**	***c*** **($m \cdot s^{-1}$)**	***p*** **(MPa)**	***c*** **($m \cdot s^{-1}$)**
**Doped Mix 1:** $x_{CO_2} = 0.95769; x_{CH_3OH} = 0.00999;\ x_{O_2} = 0.02974; x_{SO_2} = 0.000893;\ x_{CO} = 0.001686$							
T **= 263.15 K**		***T*** **= 273.15 K**		***T*** **= 283.16 K**		***T*** **= 293.15 K**	
9.00	661.06	29.00	781.22	30.00	740.59	32.00	711.90
8.00	648.81	28.00	773.70	29.00	732.56	31.00	703.64
7.00	635.93	27.00	766.01	28.00	724.34	30.00	695.16
6.00	622.30	26.00	758.15	27.00	715.92	29.00	686.47
5.00	607.82	25.00	750.09	26.00	707.27	28.00	677.53
		24.00	741.84	25.00	698.38	27.00	668.34
		23.00	733.38	24.00	689.24	26.00	658.87
		22.00	724.69	23.00	679.82	25.00	649.10
		21.00	715.75	22.00	670.11	24.00	639.00
		20.00	706.55	21.00	660.08	23.00	628.56
		19.00	697.07	20.00	649.69	22.00	617.72
		18.00	687.29	19.00	638.92	21.00	606.45
		17.00	677.17	18.00	627.73	20.00	594.71
		16.00	666.68	17.00	616.07	19.00	582.44
		15.00	655.80	16.00	603.88	18.00	569.57
		14.00	644.47	15.00	591.11	17.00	556.01
		13.00	632.65	14.00	577.67	16.00	541.68
		12.00	620.29	13.00	563.47	15.00	526.43
		11.00	607.29	12.00	548.38	14.00	510.10
		10.00	593.59	11.00	532.23	13.00	492.46
		9.00	579.06	10.00	514.82	12.00	473.19
		8.00	563.57	9.00	495.85	11.00	451.82
		7.00	546.92	8.00	474.89	10.00	427.60
		6.00	528.86	7.00	451.27	9.00	399.25
						8.00	364.05

Table S8 (continued). pcT extrapolated values for the CO_2 + CH_3OH + O_2 + SO_2 + CO and CO_2 + CH_3OH + NO + SO_2 + CO (doped Mix 1 and doped Mix 2) mixtures, using $p^{\#}$ and the coefficients from Table S7.

p **(MPa)**	***c*** **($m \cdot s^{-1}$)**	***p*** **(MPa)**	***c*** **($m \cdot s^{-1}$)**	***p*** **(MPa)**	***c*** **($m \cdot s^{-1}$)**	***p*** **(MPa)**	***c*** **($m \cdot s^{-1}$)**
**Doped Mix 1:** $x_{CO_2} = 0.95769; x_{CH_3OH} = 0.00999;\ x_{O_2} = 0.02974; x_{SO_2} = 0.000893;\ x_{CO} = 0.001686$							
T **= 303.15 K**		***T*** **= 313.16 K**		***T*** **= 333.15 K**		***T*** **= 353.15 K**	
49.00	791.62	44.00	724.77	57.00	745.82	74.00	788.46
48.00	785.34	43.00	717.62	56.00	739.67	73.00	783.31
47.00	778.96	42.00	710.34	55.00	733.43	72.00	778.10
46.00	772.47	41.00	702.91	54.00	727.10	71.00	772.83
45.00	765.88	40.00	695.33	53.00	720.66	70.00	767.51
44.00	759.17	39.00	687.59	52.00	714.13	69.00	762.13
43.00	752.34	38.00	679.68	51.00	707.49	68.00	756.68
42.00	745.39	37.00	671.59	50.00	700.74	67.00	751.17
41.00	738.30	36.00	663.31	49.00	693.87	66.00	745.59
40.00	731.08	35.00	654.82	48.00	686.89	65.00	739.94
39.00	723.71	34.00	646.12	47.00	679.78	64.00	734.22
38.00	716.19	33.00	637.19	46.00	672.54	63.00	728.43
37.00	708.50	32.00	628.01	45.00	665.16	62.00	722.57
36.00	700.65	31.00	618.57	44.00	657.64	61.00	716.62
35.00	692.62	30.00	608.84	43.00	649.97	60.00	710.60
34.00	684.40	29.00	598.80	42.00	642.14	59.00	704.50
33.00	675.97	28.00	588.44	41.00	634.14	58.00	698.30
32.00	667.33	27.00	577.70	40.00	625.96	57.00	692.02
31.00	658.46	26.00	566.57	39.00	617.60	56.00	685.65
30.00	649.34	25.00	555.00	38.00	609.05	55.00	679.19
29.00	639.95	24.00	542.95	37.00	600.28	54.00	672.62
28.00	630.28	23.00	530.36	36.00	591.29	53.00	665.95
27.00	620.30	22.00	517.16	35.00	582.06	52.00	659.18
26.00	609.98	21.00	503.27	34.00	572.59	51.00	652.30
25.00	599.29	20.00	488.60	33.00	562.84	50.00	645.30
24.00	588.20	19.00	473.01	32.00	552.80	49.00	638.18

23.00	576.67	18.00	456.35	31.00	542.44	48.00	630.93
22.00	564.64	17.00	438.40	30.00	531.75	47.00	623.56
21.00	552.06	16.00	418.86	29.00	520.69	46.00	616.05
20.00	538.85	15.00	397.33	28.00	509.23	45.00	608.40
19.00	524.94	14.00	373.16	27.00	497.33	44.00	600.59
18.00	510.21	13.00	345.30	26.00	484.95	43.00	592.63
17.00	494.52	12.00	311.79	25.00	472.03	42.00	584.51
16.00	477.70	11.00	267.98	24.00	458.52	41.00	576.21
15.00	459.51			23.00	444.34	40.00	567.73
14.00	439.60			22.00	429.40	39.00	559.06
13.00	417.48			21.00	413.59	38.00	550.19
12.00	392.36			20.00	396.78	37.00	541.10
11.00	362.85			19.00	378.79	36.00	531.79
10.00	326.02			18.00	359.41	35.00	522.23
9.00	272.98			17.00	338.31	34.00	512.41
				16.00	315.09	33.00	502.32
				15.00	289.11	32.00	491.94
						31.00	481.24
						30.00	470.20
						29.00	458.79
						28.00	446.99
						27.00	434.75
						26.00	422.04
						25.00	408.82
						24.00	395.03
						23.00	380.61
						22.00	365.48
						21.00	349.57
						20.00	332.77
						19.00	314.96

Table S8 (continued). *pcT* extrapolated values for the CO_2 + CH_3OH + O_2 + SO_2 + CO and CO_2 + CH_3OH + NO + SO_2 + CO (doped Mix 1 and doped Mix 2) mixtures, using $p^{\#}$ and the coefficients from Table S7.

***p* (MPa)**	***c* (m·s^{-1})**	***p* (MPa)**	***c* (m·s^{-1})**	***p* (MPa)**	***c* (m·s^{-1})**	***p* (MPa)**	***c* (m·s^{-1})**
**Doped Mix 1:** $x_{CO_2} = 0.95769; x_{CH_3OH} = 0.00999;\ x_{O_2} = 0.02974; x_{SO_2} = 0.000893;\ x_{CO} = 0.001686$							
***T* = 373.14 K**							
95.00	843.55						
94.00	839.23						
93.00	834.88						
92.00	830.49						
91.00	826.07						
90.00	821.61						
89.00	817.11						
88.00	812.57						
87.00	807.99						
86.00	803.37						
85.00	798.71						
84.00	794.01						
83.00	789.26						
82.00	784.47						
81.00	779.63						
80.00	774.75						
79.00	769.82						
78.00	764.84						
77.00	759.81						
76.00	754.73						
75.00	749.60						
74.00	744.41						
73.00	739.17						
72.00	733.87						
71.00	728.51						

70.00	723.10						
69.00	717.62						
68.00	712.08						
67.00	706.48						
66.00	700.81						
65.00	695.07						
64.00	689.26						
63.00	683.38						
62.00	677.42						
61.00	671.39						
60.00	665.27						
59.00	659.08						
58.00	652.80						
57.00	646.43						
56.00	639.98						
55.00	633.43						
54.00	626.78						
53.00	620.03						
52.00	613.18						
51.00	606.22						
50.00	599.15						
49.00	591.96						
48.00	584.65						
47.00	577.21						
46.00	569.65						
45.00	561.94						
44.00	554.10						
43.00	546.10						
42.00	537.95						
41.00	529.64						
40.00	521.16						
39.00	512.49						
38.00	503.64						
37.00	494.59						

36.00	485.33						
35.00	475.84						
34.00	466.13						
33.00	456.17						
32.00	445.94						
31.00	435.44						
30.00	424.65						
29.00	413.53						
28.00	402.09						
27.00	390.27						
26.00	378.08						
25.00	365.46						
24.00	352.40						
23.00	338.85						

Table S8 (Continued). pcT extrapolated values for the CO_2 + CH_3OH + O_2 + SO_2 + CO and CO_2 + CH_3OH + NO + SO_2 + CO (doped Mix 1 and doped Mix 2) mixtures, using $p^{\#}$ and the coefficients from Table S7.

p **(MPa)**	***c*** **(m·s^{-1})**	***p*** **(MPa)**	***c*** **(m·s^{-1})**	***p*** **(MPa)**	***c*** **(m·s^{-1})**	***p*** **(MPa)**	***c*** **(m·s^{-1})**
**Doped Mix 2:** $x_{CO_2} = 0.98586; x_{CH_3OH} = 0.01016; x_{NO} = 0.001396; x_{SO_2} = 0.000899; x_{CO} = 0.001683$							
***T* = 263.14 K**		***T* = 273.16 K**		***T* = 283.15 K**		***T* = 293.15 K**	
9.00	699.89	14.00	686.08	24.00	723.07	29.00	715.10
8.00	688.86	13.00	675.36	23.00	714.26	28.00	706.61
7.00	677.35	12.00	664.19	22.00	705.19	27.00	697.90
6.00	665.30	11.00	652.54	21.00	695.84	26.00	688.94
5.00	652.62	10.00	640.34	20.00	686.20	25.00	679.72
4.00	639.24	9.00	627.52	19.00	676.23	24.00	670.22
3.00	625.03	8.00	613.99	18.00	665.91	23.00	660.41
		7.00	599.63	17.00	655.20	22.00	650.26
		6.00	584.30	16.00	644.06	21.00	639.75
		5.00	567.80	15.00	632.44	20.00	628.83
		4.00	549.85	14.00	620.28	19.00	617.47
				13.00	607.52	18.00	605.61
				12.00	594.07	17.00	593.19
				11.00	579.81	16.00	580.14
				10.00	564.62	15.00	566.37
				9.00	548.30	14.00	551.75
				8.00	530.60	13.00	536.15
				7.00	511.17	12.00	519.35
				6.00	489.46	11.00	501.08
				5.00	464.56	10.00	480.94
						9.00	458.32
						8.00	432.17
						7.00	400.43
						6.00	357.73

Table S8 (continued). pcT extrapolated values for the CO_2 + CH_3OH + O_2 + SO_2 + CO and CO_2 + CH_3OH + NO + SO_2 + CO (doped Mix 1 and doped Mix 2) mixtures, using $p^\#$ and the coefficients from Table S7.

p **(MPa)**	***c*** **(m·s^{-1})**	***p*** **(MPa)**	***c*** **(m·s^{-1})**	***p*** **(MPa)**	***c*** **(m·s^{-1})**	***p*** **(MPa)**	***c*** **(m·s^{-1})**
**Doped Mix 2:** $x_{CO_2} = 0.98586$; $x_{CH_3OH} = 0.01016$; $x_{NO} = 0.001396$; $x_{SO_2} = 0.000899$; $x_{CO} = 0.001683$							
T **= 303.15 K**		***T*** **= 313.15 K**		***T*** **= 333.15 K**		***T*** **= 353.14 K**	
39.00	747.97	44.00	743.80	65.00	805.92	79.00	824.36
38.00	740.70	43.00	736.84	64.00	800.46	78.00	819.50
37.00	733.28	42.00	729.74	63.00	794.94	77.00	814.59
36.00	725.71	41.00	722.50	62.00	789.35	76.00	809.64
35.00	717.97	40.00	715.13	61.00	783.69	75.00	804.63
34.00	710.06	39.00	707.59	60.00	777.95	74.00	799.56
33.00	701.95	38.00	699.90	59.00	772.14	73.00	794.45
32.00	693.65	37.00	692.04	58.00	766.24	72.00	789.27
31.00	685.14	36.00	684.00	57.00	760.27	71.00	784.04
30.00	676.41	35.00	675.77	56.00	754.21	70.00	778.75
29.00	667.43	34.00	667.33	55.00	748.06	69.00	773.40
28.00	658.19	33.00	658.68	54.00	741.81	68.00	767.99
27.00	648.68	32.00	649.80	53.00	735.47	67.00	762.52
26.00	638.86	31.00	640.68	52.00	729.04	66.00	756.97
25.00	628.71	30.00	631.29	51.00	722.50	65.00	751.36
24.00	618.20	29.00	621.61	50.00	715.85	64.00	745.69
23.00	607.30	28.00	611.63	49.00	709.09	63.00	739.93
22.00	595.97	27.00	601.31	48.00	702.21	62.00	734.11
21.00	584.15	26.00	590.62	47.00	695.21	61.00	728.20
20.00	571.79	25.00	579.53	46.00	688.08	60.00	722.22
19.00	558.82	24.00	568.00	45.00	680.82	59.00	716.16
18.00	545.16	23.00	555.98	44.00	673.42	58.00	710.01
17.00	530.70	22.00	543.41	43.00	665.87	57.00	703.77
16.00	515.30	21.00	530.23	42.00	658.17	56.00	697.44
15.00	498.79	20.00	516.34	41.00	650.30	55.00	691.02

14.00	480.93	19.00	501.64	40.00	642.26	54.00	684.49
13.00	461.37	18.00	485.99	39.00	634.04	53.00	677.87
12.00	439.62	17.00	469.23	38.00	625.62	52.00	671.14
11.00	414.86	16.00	451.11	37.00	617.00	51.00	664.30
10.00	385.63	15.00	431.30	36.00	608.16	50.00	657.35
9.00	348.76	14.00	409.32	35.00	599.09	49.00	650.28
8.00	293.45	13.00	384.41	34.00	589.78	48.00	643.08
		12.00	355.24	33.00	580.19	47.00	635.75
		11.00	319.05	32.00	570.32	46.00	628.29
		10.00	267.77	31.00	560.14	45.00	620.68
				30.00	549.63	44.00	612.93
				29.00	538.75	43.00	605.01
				28.00	527.47	42.00	596.94
				27.00	515.76	41.00	588.69
				26.00	503.57	40.00	580.26
				25.00	490.84	39.00	571.63
				24.00	477.52	38.00	562.81
				23.00	463.52	37.00	553.76
				22.00	448.75	36.00	544.49
				21.00	433.09	35.00	534.97
				20.00	416.41	34.00	525.20
				19.00	398.49	33.00	515.14
				18.00	379.08	32.00	504.79
				17.00	357.83	31.00	494.12
				16.00	334.21	30.00	483.10
				15.00	307.38	29.00	471.71
						28.00	459.91
						27.00	447.67
						26.00	434.95
						25.00	421.69
						24.00	407.83
						23.00	393.32
						22.00	378.08
						21.00	361.99

						20.00	344.95
						19.00	326.82
						18.00	307.40

Table S8 (continued). pcT extrapolated values for the CO_2 + CH_3OH + O_2 + SO_2 + CO and CO_2 + CH_3OH + NO + SO_2 + CO (doped Mix 1 and doped Mix 2) mixtures, using $p^{\#}$ and the coefficients from Table S7.

p **(MPa)**	***c*** **(m·s⁻¹)**	***p*** **(MPa)**	***c*** **(m·s⁻¹)**	***p*** **(MPa)**	***c*** **(m·s⁻¹)**	***p*** **(MPa)**	***c*** **(m·s⁻¹)**
**Doped Mix 2:** $x_{CO_2} = 0.98586; x_{CH_3OH} = 0.01016;\ x_{NO} = 0.001396; x_{SO_2} = 0.000899;\ x_{CO} = 0.001683$							
***T* = 373.15 K**							
104.00	889.81						
103.00	885.79						
102.00	881.75						
101.00	877.68						
100.00	873.57						
99.00	869.43						
98.00	865.27						
97.00	861.07						
96.00	856.83						
95.00	852.57						
94.00	848.27						
93.00	843.93						
92.00	839.56						
91.00	835.15						
90.00	830.71						
89.00	826.22						
88.00	821.70						
87.00	817.14						
86.00	812.54						
85.00	807.89						
84.00	803.21						
83.00	798.48						
82.00	793.71						
81.00	788.89						
80.00	784.02						

79.00	779.11						
78.00	774.15						
77.00	769.14						
76.00	764.08						
75.00	758.97						
74.00	753.80						
73.00	748.58						
72.00	743.31						
71.00	737.97						
70.00	732.58						
69.00	727.12						
68.00	721.61						
67.00	716.03						
66.00	710.38						
65.00	704.66						
64.00	698.88						
63.00	693.02						
62.00	687.09						
61.00	681.08						
60.00	674.99						
59.00	668.82						
58.00	662.57						
57.00	656.23						
56.00	649.80						
55.00	643.27						
54.00	636.65						
53.00	629.93						
52.00	623.11						
51.00	616.18						
50.00	609.14						
49.00	601.98						
48.00	594.70						
47.00	587.29						
46.00	579.76						

45.00	572.08						
44.00	564.27						
43.00	556.31						
42.00	548.19						
41.00	539.90						
40.00	531.45						
39.00	522.82						
38.00	513.99						
37.00	504.97						
36.00	495.74						
35.00	486.29						
34.00	476.60						
33.00	466.67						
32.00	456.47						
31.00	446.00						
30.00	435.22						
29.00	424.13						
28.00	412.69						
27.00	400.90						
26.00	388.70						
25.00	376.09						
24.00	363.02						
23.00	349.45						
22.00	335.34						
21.00	320.64						

Table S9. $p\kappa_S T$ values for the CO_2 + O_2 + SO_2 + CO (Mix 1) and CO_2 + NO + SO_2 + CO (Mix 2) mixtures at the nominal temperatures T.

p **(MPa)**	κ_S **(10^{-3}·MPa^{-1})**	***p*** **(MPa)**	κ_S **(10^{-3}·MPa^{-1})**	***p*** **(MPa)**	κ_S **(10^{-3}·MPa^{-1})**	***p*** **(MPa)**	κ_S **(10^{-3}·MPa^{-1})**
**Mix 1: x_{CO_2} = 0.96734; x_{O_2} = 0.030038; x_{SO_2} = 0.0009035; x_{CO} = 0.0017032**							
***T* = 263.15 K**		***T* = 273.15 K**		***T* = 283.15 K**		***T* = 293.15 K**	
5	2.80	6	3.91	7	5.76	8	9.73
6	2.65	7	3.62	8	5.13	9	7.86
7	2.52	8	3.38	9	4.64	10	6.70
8	2.41	9	3.18	10	4.25	11	5.90
9	2.31	10	3.00	11	3.94	12	5.30
10	2.22	11	2.85	12	3.67	13	4.82
11	2.14	12	2.71	13	3.45	14	4.44
12	2.06	13	2.59	14	3.26	15	4.12
13	1.99	14	2.48	15	3.09	16	3.86
14	1.93	15	2.38	16	2.94	17	3.63
15	1.87	16	2.30	17	2.80	18	3.43
16	1.81	17	2.21	18	2.68	19	3.26
17	1.76	18	2.14	19	2.58	20	3.10
18	1.72	19	2.07	20	2.48		
19	1.67	20	2.01				
20	1.63						
***T* = 303.15 K**		***T* = 313.15 K**		***T* = 333.15 K**		***T* = 353.15 K**	
9	20.07	13	12.09	15	21.50	19	18.79
10	13.19	14	9.98	16	16.98	20	16.05
11	10.23	15	8.56	17	14.02	21	13.97
12	8.49	16	7.52	18	11.93	22	12.34
13	7.33	17	6.73	19	10.40	23	11.03
14	6.48	18	6.10	20	9.21	24	9.96
15	5.83	19	5.59	21	8.28	25	9.08
16	5.32	20	5.17	22	7.52	26	8.34
17	4.90			23	6.90	27	7.70
18	4.55			24	6.37	28	7.16

19	4.25			25	5.91	29	6.68
20	3.99			26	5.53	30	6.27
				27	5.19		
				28	4.89		
				29	4.63		
				30	4.39		
***T* = 373.15 K**							
23	16.56						
24	14.75						
25	13.27						
26	12.03						
27	10.99						
28	10.10						
29	9.33						
30	8.67						

Table S9 (continued). $p\kappa_S T$ values for the CO_2 + O_2 + SO_2 + CO (Mix 1) and CO_2 + NO + SO_2 + CO (Mix 2) mixtures at the nominal temperatures T.

**p (MPa)**	**κ_S (10^{-3}·MPa^{-1})**	**p (MPa)**	**κ_S (10^{-3}·MPa^{-1})**	**p (MPa)**	**κ_S (10^{-3}·MPa^{-1})**	**p (MPa)**	**κ_S (10^{-3}·MPa^{-1})**
**Mix 2: x_{CO_2}= 0.99592; x_{NO}= 0.001410; x_{SO_2}= 0.0009100; x_{CO}= 0.0017002**							
***T* = 263.15 K**		***T* = 273.15 K**		***T* = 283.15 K**		***T* = 293.15 K**	
3	2.61	4	3.56	6	4.74	6	10.05
4	2.48	5	3.31	7	4.29	7	7.74
5	2.36	6	3.10	8	3.94	8	6.49
6	2.26	7	2.92	9	3.65	9	5.67
7	2.17	8	2.76	10	3.41	10	5.07
8	2.09	9	2.63	11	3.21	11	4.61
9	2.01	10	2.51	12	3.04	12	4.24
10	1.93	11	2.40	13	2.88	13	3.93
11	1.87	12	2.31	14	2.75	14	3.68
12	1.81	13	2.22	15	2.63	15	3.46
13	1.76	14	2.14	16	2.52	16	3.27
14	1.72	15	2.07	17	2.42	17	3.10
15	1.67	16	2.00	18	2.33	18	2.96
16	1.63	17	1.94	19	2.25	19	2.83
17	1.59	18	1.88	20	2.17	20	2.71
18	1.55	19	1.83				
19	1.52	20	1.78				
20	1.48						
***T* = 303.15 K**		***T* = 313.15 K**		***T* = 333.15 K**		***T* = 353.15 K**	
8	16.62	10	22.25	15	17.60	18	19.66
9	11.08	11	14.41	16	14.10	19	16.51
10	8.74	12	11.05	17	11.80	20	14.18
11	7.36	13	9.13	18	10.12	21	12.42
12	6.42	14	7.83	19	8.94	22	11.02
13	5.73	15	6.90	20	8.00	23	9.90
14	5.19	16	6.20	21	7.24	24	8.99
15	4.76	17	5.64	22	6.62	25	8.22

16	4.41	18	5.18	23	6.11	26	7.58
17	4.11	19	4.80	24	5.67	27	7.02
18	3.86	20	4.48	25	5.29	28	6.55
19	3.64			26	4.97	29	6.13
20	3.45			27	4.68	30	5.77
				28	4.43		
				29	4.20		
				30	4.00		
***T* = 373.15 K**							
21	19.32						
22	16.87						
23	14.93						
24	13.34						
25	12.05						
26	10.96						
27	10.05						
28	9.26						
29	8.59						
30	8.00						

Table S10. $p\mu_{JT}T$ values for the CO_2 + O_2 + SO_2 + CO (Mix 1) and CO_2 + NO + SO_2 + CO (Mix 2) mixtures, at the nominal temperatures *T*.

***p* (MPa)**	**μ_{JT} (K·MPa^{-1})**	***p* (MPa)**	**μ_{JT} (K·MPa^{-1})**	***p* (MPa)**	**μ_{JT} (K·MPa^{-1})**	***p* (MPa)**	**μ_{JT} (K·MPa^{-1})**
**Mix 1: x_{CO_2}= 0.96734; x_{O_2}= 0.030038; x_{SO_2}= 0.0009035; x_{CO}= 0.0017032**							
***T* = 263.15 K**		***T* = 273.15 K**		***T* = 283.15 K**		***T* = 293.15 K**	
5	0.258	6	0.422	7	0.736	8	1.147
6	0.217	7	0.377	8	0.638	9	1.009
7	0.195	8	0.333	9	0.554	10	0.833
8	0.168	9	0.298	10	0.483	11	0.727
9	0.143	10	0.258	11	0.422	12	0.650
10	0.126	11	0.227	12	0.373	13	0.581
11	0.106	12	0.198	13	0.331	14	0.509
12	0.087	13	0.176	14	0.296	15	0.438
13	0.076	14	0.153	15	0.265	16	0.379
14	0.063	15	0.128	16	0.237	17	0.343
15	0.046	16	0.125	17	0.211	18	0.321
16	0.037	17	0.104	18	0.185	19	0.293
17	0.025	18	0.084	19	0.163	20	0.251
18	-0.004	19	0.069	20	0.146		
19	-0.010	20	0.058				
20	-0.019						
***T* = 303.15 K**		***T* = 313.15 K**		***T* = 333.15 K**		***T* = 353.15 K**	
9	2.150	13	1.542	15	2.516	19	2.112
10	1.552	14	1.270	16	2.158	20	1.899
11	1.283	15	1.102	17	1.841	21	1.709
12	1.111	16	0.971	18	1.582	22	1.531
13	0.908	17	0.860	19	1.384	23	1.382
14	0.804	18	0.771	20	1.182	24	1.254
15	0.716	19	0.701	21	1.064	25	1.147
16	0.636	20	0.639	22	0.971	26	1.051
17	0.567			23	0.890	27	0.967
18	0.509			24	0.809	28	0.875

19	0.459			25	0.729	29	0.788
20	0.406			26	0.646	30	0.718
				27	0.574		
				28	0.526		
				29	0.518		
				30	0.486		
***T* = 373.15 K**							
23	1.833						
24	1.675						
25	1.534						
26	1.395						
27	1.295						
28	1.209						
29	1.130						
30	1.042						

Table S10 (continued). $p\mu_{JT}T$ values for the CO_2 + O_2 + SO_2 + CO (Mix 1) and CO_2 + NO + SO_2 + CO (Mix 2) mixtures, at the nominal temperatures *T*.

p **(MPa)**	μ_{JT} **(K·MPa⁻¹)**	***p*** **(MPa)**	μ_{JT} **(K·MPa⁻¹)**	***p*** **(MPa)**	μ_{JT} **(K·MPa⁻¹)**	***p*** **(MPa)**	μ_{JT} **(K·MPa⁻¹)**
**Mix 2:** x_{CO_2} **= 0.99592;** x_{NO} **= 0.001410;** x_{SO_2} **= 0.0009100;** x_{CO} **= 0.0017002**							
T **= 263.15 K**		***T*** **= 273.15 K**		***T*** **= 283.15 K**		***T*** **= 293.15 K**	
4	0.191	4	0.386	6	0.629	6	1.528
5	0.143	5	0.354	7	0.536	7	1.181
6	0.172	6	0.305	8	0.466	8	0.867
7	0.143	7	0.272	9	0.410	9	0.721
8	0.105	8	0.244	10	0.366	10	0.637
9	0.095	9	0.213	11	0.328	11	0.576
10	0.092	10	0.180	12	0.292	12	0.518
11	0.075	11	0.158	13	0.260	13	0.461
12	0.057	12	0.137	14	0.232	14	0.403
13	0.047	13	0.116	15	0.206	15	0.350
14	0.024	14	0.102	16	0.184	16	0.319
15	0.029	15	0.079	17	0.164	17	0.302
16	0.016	16	0.064	18	0.145	18	0.283
17	0.010	17	0.049	19	0.124	19	0.234
18	0.002	18	0.036	20	0.096	20	0.198
19	-0.008	19	0.023				
20	-0.069	20	0.011				
T **= 303.15 K**		***T*** **= 313.15 K**		***T*** **= 333.15 K**		***T*** **= 353.15 K**	
8	2.249	10	2.835	15	2.313	18	2.275
9	1.635	11	1.935	16	1.914	19	1.987
10	1.195	12	1.591	17	1.607	20	1.771
11	1.015	13	1.214	18	1.376	21	1.578
12	0.857	14	0.960	19	1.199	22	1.424
13	0.727	15	0.890	20	1.061	23	1.285
14	0.680	16	0.785	21	0.958	24	1.159
15	0.584	17	0.730	22	0.856	25	1.052
16	0.520	18	0.647	23	0.767	26	0.961

17	0.469	19	0.580	24	0.689	27	0.883
18	0.421	20	0.520	25	0.627	28	0.813
19	0.375			26	0.579	29	0.738
20	0.361			27	0.539	30	0.635
				28	0.497		
				29	0.433		
				30	0.320		
***T* = 373.15 K**							
21	2.362						
22	2.033						
23	1.797						
24	1.620						
25	1.484						
26	1.367						
27	1.256						
28	1.148						
29	1.045						
30	0.968						

Table S11. Parameters used in the modeling of Mix 1 and Mix 2 with the PC-SAFT EoS.

System	Compound	Pure compound parameters			
		m/M (mol/g)	σ (Å)	ε/k (K)	Δv_c (cm^3/g)
**Mix 1:** CO_2 + O_2 + SO_2 + CO	**CO_2**	0.047101[a]	2.7852[a]	169.210[a]	0.02[b]
	**O_2**	0.035055[c]	3.210[c]	114.96[c]	–
	**SO_2**	0.04466[a]	2.6826[a]	205.35[a]	0.01[d]
	**CO**	0.046758[a]	3.2507[a]	92.15[a]	-0.1[b]
	**Binary interaction parameters:** $O_2 - CO_2$: $k_{ij} = 0.049$[c]; $SO_2 - CO_2$: $k_{ij} = 0.03$[d]; $CO - CO_2$: $k_{ij} = 0.12$[b] $O_2 - SO_2$, $O_2 - CO$ and $CO - SO_2$: $k_{ij} = 0$				
	**Compound**	**Pure compound parameters**			
**Mix 2:** CO_2 + NO + SO_2 + CO	**CO_2**	0.047101[a]	2.7852[a]	169.21[a]	0.02[b]
	**NO**	0.050529948[e]	3.12930084[e]	130.4152075[e]	0.41342938[e]
	**SO_2**	0.04466[a]	2.6826[a]	205.35[a]	0.01[d]
	**CO**	0.04466[a]	2.6826[a]	205.35[a]	0.01[b]
	**Binary interaction parameters:** $NO{-}CO_2$: $k_{ij} = 0.00878372 * T - 2.280368377$[e]; $SO_2 - CO_2$: $k_{ij} = 0.03$[d] $CO - CO_2$: $k_{ij} = 0.12$[b] $NO - SO_2$, $NO - CO$ and $CO - SO_2$: $k_{ij} = 0$				

(a) Gross, J.; Sadowski, G. Perturbed-Chain SAFT: An Equation of State Based on a Perturbation Theory for Chain Molecules. *Ind. Eng. Chem. Res.* **2001**, *40* (4), 1244-1260. DOI: 10.1021/ie0003887

(b) Rivas, C.; Gimeno, B.; Artal, M.; Blanco, S. T.; Fernández, J.; Velasco, I. High-pressure speed of sound in pure CO_2 and in CO_2 with SO_2 as an impurity using methanol as a doping agent. *Int. J. Greenh. Gas Con.* **2016**, *54* (2), 737-751. DOI: 10.1016/j.ijggc.2016.09.014

(c) Abolala, M.; Penyvandi, K.; Varaminian, F.; Hashemianzadeh, S. M. A comprehensive description of single-phase and VLE properties of cryogenic fluids using molecular-based equations of state. *Fluid Phase Equilibr.* **2019**, *494*, 143-160. DOI: 10.1016/j.fluid.2019.04.038

(d) Diamantonis, N. I.; Boulougouris, G. C.; Mansoor, E.; Tsangaris, D. M.; Economou, I. G. Evaluation of Cubic, SAFT, and PC-SAFT Equations of State for the Vapor-Liquid Equilibrium Modeling of CO2 Mixtures with Other Gases. *Ind. Eng. Chem. Res.* **2013**, *52* (10), 3933-3942. DOI: 10.1021/ie303248q

(e) Melendo, A. P.; Berbés, R.; Blanco, S. T.; Fernández, J. Effect of the impurities O_2 or NO present in non-purified flue gas from oxy-fuel combustion processes for carbon capture and storage technology. *Process Saf. Environ.* **2023**, *172*, 1120-1131. DOI: 10.1016/j.psep.2023.02.086

Table S12. Comparison between the experimental (exp) $p\rho T$, the experimental (exp) pcT and the extrapolated (ext) pcT data presented in this work for the CO_2 + O_2 + SO_2 + CO (Mix 1) and CO_2 + NO + SO_2 + CO (Mix 2) mixtures and those calculated using the GERG-2008, the EOS-CG or the PC-SAFT EoS with parameters from table S11. The doped mixtures for c measurements were modeled as pseudobinary mixtures in which the mole fraction of CO_2 was $x_{CO_2} = 1 - \sum_i x_i$; i: impurity.

nominal T(K)	EoS	$MRD_{\rho,exp}$(%) Mix 1: CO_2+O_2+SO_2+CO	Mix 2: CO_2+NO+SO_2+CO	$MRD_{c,exp}$(%) Mix 1: CO_2+O_2+SO_2+C	Mix 2: CO_2+NO+SO_2+CO	$MRD_{c,ext}$(%) Mix 1: CO_2+O_2+SO_2+C	Mix 2: CO_2+NO+SO_2+CO
**263.15**	EOS-CG 2019	0.22		0.69		1.45	
	GERG 2008	0.41		0.41		0.87	
	PC-SAFT	0.63		4.55		3.08	
	PC-SAFT		0.49		4.57		1.64
**273.15**	EOS-CG 2019	0.24		0.71		1.37	
	GERG 2008	0.44		0.30		1.11	
	PC-SAFT	0.76		4.35		2.42	
	PC-SAFT		0.45		4.60		2.33
**283.15**	EOS-CG 2019	0.18		0.60		1.24	
	GERG 2008	0.26		0.23		0.79	
	PC-SAFT	0.63		4.19		3.33	
	PC-SAFT)		0.88		4.66		2.39
**293.15**	EOS-CG 2019	0.22		0.42		0.93	
	GERG 2008	0.26		0.14		0.33	
	PC-SAFT	0.55		4.16		4.36	
	PC-SAFT		1.92		4.52		4.11
**303.15**	EOS-CG 2019	1.38		0.47		0.59	
	GERG 2008	1.94		0.08		0.07	
	PC-SAFT	2.10		4.30		4.37	
	PC-SAFT		0.46		4.55		4.19

**313.15**	EOS-CG 2019	1.12		0.26		0.25	
	GERG 2008	1.67		0.25		0.66	
	PC-SAFT	1.01		4.30		3.98	
	PC-SAFT		1.55		4.42		3.88
**333.15**	EOS-CG 2019	0.42		0.20		0.42	
	GERG 2008	0.72		0.21		0.91	
	PC-SAFT	1.42		4.27		2.88	
	PC-SAFT		1.62		4.37		2.91
**353.15**	EOS-CG 2019	0.35		0.15		0.44	
	GERG 2008	0.60		0.11		0.91	
	PC-SAFT	1.89		4.19		2.66	
	PC-SAFT		1.88		4.17		2.76
**373.15**	EOS-CG 2019	0.45		0.15		0.33	
	GERG 2008	0.47		0.10		0.74	
	PC-SAFT	2.33		4.06		2.73	
	PC-SAFT		1.78		4.02		2.83
$\overline{\overline{\mathbf{MRD}}}_{X}$ **(%)**	EOS-CG 2019	0.50		0.42		0.59	
	GERG 2008	0.73		0.21		0.70	
	PC-SAFT	1.42		4.27		3.23	
	PC-SAFT		1.30		4.45		3.10

$\mathrm{MRD}_{X,\mathrm{exp}}(\%) = \frac{100}{N}\sum\left|\frac{X_{\mathrm{EoS}}-X_{\mathrm{exp}}}{X_{\mathrm{exp}}}\right|$ $\mathrm{MRD}_{c,\mathrm{ext}}(\%) = \frac{100}{N}\sum\left|\frac{c_{\mathrm{ext}}-c_{\mathrm{EoS}}}{c_{\mathrm{EoS}}}\right|$ *N*: number of points for each composition and temperature.

$\overline{\overline{\mathrm{MRD}}}_{X,\mathrm{exp}}(\%) = \frac{100}{N'}\sum\left|\frac{X_{\mathrm{EoS}}-X_{\mathrm{exp}}}{X_{\mathrm{exp}}}\right|$ $\overline{\overline{\mathrm{MRD}}}_{c,\mathrm{ext}}(\%) = \frac{100}{N'}\sum\left|\frac{c_{\mathrm{ext}}-c_{\mathrm{EoS}}}{c_{\mathrm{EoS}}}\right|$ *N*’: total number of points for each property.

Table S13. Comparison between the experimental VLE data presented in this work for the CO_2 + O_2 + SO_2 + CO (Mix 1) and CO_2 + NO + SO_2 + CO (Mix 2) mixtures and those calculated using the EOS-CG 2019, the GERG-2008, or the PC-SAFT EoS with parameters from Table S11, in terms of mean relative deviation, MRD(%), and overall mean relative deviation, $\overline{\overline{\mathrm{MRD}}}$ (%).

Composition	EoS	$\mathbf{MRD}_{p_{\mathrm{dew}}}(\%)$	$\mathbf{MRD}_{p_{\mathrm{bubble}}}(\%)$	$\mathbf{MRD}_{\rho_{\mathrm{V}}}(\%)$	$\mathbf{MRD}_{\rho_{\mathrm{L}}}(\%)$
**Mix 1: (CO_2 + 3.0038 mol% O_2 + 0.09035 mol% SO_2 + 0.17032 mol% CO)**	EOS-CG 2019	0.06	0.09	0.75	0.09
	GERG 2008	0.13	9.79	0.25	0.76
	PC-SAFT	1.35	0.43	4.04	1.08
**Mix 2: (CO_2 + 0.1410 mol% NO + 0.09100 mol% SO_2 + 0.17002 mol% CO)**	PC-SAFT	2.31	1.62	3.47	1.23

$$\mathrm{MRD}_X(\%) = \frac{100}{N}\left|\frac{X_{\mathrm{EoS}} - X_{\mathrm{exp}}}{X_{\mathrm{exp}}}\right|$$

N: number of experimental points for each composition.

$$\overline{\overline{\mathrm{MRD}_X}}(\%) = \frac{100}{N'}\sum\left|\frac{X_{\mathrm{EoS}} - X_{\mathrm{exp}}}{X_{\mathrm{exp}}}\right|$$

N': number of experimental points for each property.

Table S14. Comparison between the calculated κ_S data presented in this work for Mix 1 and Mix 2 and those calculated using the evaluated EoS in terms of absolute average deviation, AAD($10^{-3}\cdot MPa^{-1}$) and overall mean absolute deviation, $\overline{\overline{AAD}}$ ($10^{-3}\cdot MPa^{-1}$).

Composition	Nominal *T* (K)	EOS-CG	GERG-2008	PC-SAFT	PC-SAFT
**Mix 1:** **(CO_2 + 3.0038 mol% O_2 + 0.09035 mol% SO_2 + 0.17032 mol% CO)**	263.15	0.037	0.018	0.071	
	273.15	0.094	0.007	0.209	
	283.15	0.108	0.057	0.412	
	293.15	0.155	0.149	0.848	
	303.15	0.290	0.385	1.925	
	313.15	0.018	0.389	1.080	
	333.15	0.059	0.213	0.135	
	353.15	0.178	0.322	0.858	
	373.15	0.182	0.219	0.947	
	**$\overline{\overline{AAD}}$ ($10^{-3}\cdot MPa^{-1}$)**	0.129	0.193	0.755	
**Mix 2:** **(CO_2 + 0.1410 mol% NO + 0.09100 mol% SO2 + 0.17002 mol% CO)**	263.15				0.059
	273.15				0.103
	283.15				0.284
	293.15				0.659
	303.15				1.257
	313.15				1.790
	333.15				0.687
	353.15				0.807
	373.15				0.907
	**$\overline{\overline{AAD}}$ ($10^{-3}\cdot MPa^{-1}$)**				0.653

PC-SAFT parameters for Mix 1 and Mix 2 from Table S11.

$$\mathrm{AAD}(10^{-3}\cdot\mathrm{MPa}^{-1}) = \frac{1}{N}\sum\left|\kappa_{\mathrm{S}_{\mathrm{EoS}}} - \kappa_{\mathrm{S}_{\mathrm{exp}}}\right|$$

N: number of experimental points for each temperature and composition.

$$\overline{\overline{\mathrm{AAD}}}(10^{-3}\cdot\mathrm{MPa}^{-1}) = \frac{1}{N'}\sum\left|\kappa_{\mathrm{S}_{\mathrm{EoS}}} - \kappa_{\mathrm{S}_{\mathrm{exp}}}\right|$$

N’: total number of experimental points.

Table S15. Comparison between the calculated μ_{JT} data presented in this work for Mix 1 and Mix 2 and those calculated using the evaluated EoS in terms of absolute average deviation, AAD(K·MPa^{-1}) and overall mean absolute deviation, $\overline{\overline{\text{AAD}}}$ (K·MPa^{-1}).

Composition	Nominal T (K)	EOS-CG	GERG-2008	PC-SAFT	PC-SAFT
**Mix 1:** (**CO_2** + **3.0038 mol% O_2** + **0.09035 mol% SO_2** + **0.17032 mol% CO**)	263.15	0.011	0.004	0.026	
	273.15	0.005	0.013	0.006	
	283.15	0.004	0.024	0.014	
	293.15	0.017	0.056	0.027	
	303.15	0.032	0.108	0.050	
	313.15	0.027	0.065	0.050	
	333.15	0.027	0.065	0.038	
	353.15	0.024	0.034	0.019	
	373.15	0.022	0.025	0.030	
	$\overline{\overline{\mathbf{AAD}}}$ $(\mathbf{K \cdot MPa^{-1}})$	0.017	0.038	0.027	
**Mix 2:** (**CO_2** + **0.1410 mol% NO** + **0.09100 mol% SO2** + **0.17002 mol% CO**)	263.15				0.026
	273.15				0.011
	283.15				0.014
	293.15				0.057
	303.15				0.141
	313.15				0.119
	333.15				0.061
	353.15				0.036
	373.15				0.055
	$\overline{\overline{\mathbf{AAD}}}$ $(\mathbf{K \cdot MPa^{-1}})$				0.054

PC-SAFT parameters for Mix 1 and Mix 2 from Table S11.

$$\mathrm{AAD}(\mathrm{K}\cdot\mathrm{MPa}^{-1}) = \frac{1}{N}\sum\left|\mu_{JT_{\mathrm{EoS}}} - \mu_{JT_{\mathrm{exp}}}\right|$$

N: number of experimental points for each temperature and composition.

$$\overline{\overline{\mathrm{AAD}}}(\mathrm{K}\cdot\mathrm{MPa}^{-1}) = \frac{1}{N'}\sum\left|\mu_{JT_{\mathrm{EoS}}} - \mu_{JT_{\mathrm{exp}}}\right|$$

N': total number of experimental points.

Table S16. Equation overview for the calculation of transport parameters in pipeline design and operation and for the calculation of injection and storage parameters[a,b,c].

	Equations	Symbols
Mass flow	$m = \rho \times v \times A$	m = mass flow (kg/s); ρ = fluid density (kg/m^3); v = fluid velocity (m/s); A = pipeline inner section (m^2).
Pipeline inner diameter	$D = \left(\frac{4 \times m}{v \times \pi \times \rho}\right)^{1/2} = \left[\frac{8 \times f \times m^2}{\rho \times \pi^2 \times \frac{\Delta P}{d}}\right]^{1/5}$	D = inner diameter (m); m = mass flow (kg/s); v = fluid velocity (m/s); ρ = fluid density (kg/m^3); f = Darcy-Weisbach friction factor; $(\Delta P/d)$ = pressure drop per metre (Pa/m).
Reynolds number	$Re = \frac{\rho \times v \times D}{\eta} = \frac{4 \times m}{\pi \times \eta \times D}$	Re = Reynolds number; ρ = fluid density (kg/m^3); v = fluid velocity (m/s); D = inner diameter (m); η = fluid viscosity (Pa.s); m = mass flow (kg/s).
Darcy-Weisbach friction factor	$f = \frac{1.325}{\left[ln\left[\frac{e}{3.7 \times D} + \frac{5.74}{Re^{0.9}}\right]\right]^2}$	f = Darcy-Weisbach friction factor; e = roughness height (m); D = inner diameter (m); Re = Reynolds number.
Pressure drop per meter	$\frac{\Delta P}{d} = \frac{8 \times f \times m^2}{\rho \times \pi^2 \times D^5}$	$(\Delta P/d)$ = pressure drop per metre (Pa/m); m = mass flow (kg/s); ρ = fluid density (kg/m^3); f = Darcy-Weisbach friction factor; D = inner diameter (m).
Normalized storage capacity	$\frac{M}{M_0} = \frac{\rho}{\rho_0\left[1 + \sum \frac{m_i}{m_0}\right]}$	M/M_0 = normalized storage capacity; ρ = mixture density (kg/m^3); ρ_0 = pure CO_2 density (kg/m^3); m_i: mass of impurity in the mixture; m_0 = mass of pure CO_2 in the mixture.
Normalized flotability in saline aquifers	$\frac{F}{F_0} = \frac{(\rho_{Br} - \rho)}{(\rho_{Br} - \rho_0)}$	F/F_0 = normalized flotability; ρ_{Br} = brine density (kg/m^3); ρ = mixture density (kg/m^3); ρ_0 = pure CO_2 density (kg/m^3).
Normalized rising velocity in saline aquifers	$\frac{v}{v_0} = \frac{F(\rho_0\eta_0)}{F_0(\rho\eta)}$	v/v_0 = normalized rising velocity; ρ_{Br} = brine density (kg/m^3); ρ = mixture density (kg/m^3); ρ_0 = pure CO_2 density (kg/m^3); η_0 = pure CO_2 viscosity (μPa.s); η = mixture viscosity (μPa.s).
Normalized permeation flux	$\frac{\dot{M}}{\dot{M}_0} = \frac{\rho\left(\frac{\eta_0}{\eta}\right)}{\rho_0\left[1 + \sum_i\left(\frac{m_i}{m_0}\right)\right]}$	$\dot{M}/\dot{M}_0$ = normalized permeation flux; ρ = mixture density (kg/m^3); ρ_0 = pure CO_2 density (kg/m^3); η_0 = pure CO_2 viscosity (μPa.s); η = mixture viscosity (μPa.s); m_i: mass of impurity in the mixture; m_0 = mass of pure CO_2 in the mixture.

(a) Wang, J.; Ryan, D.; Anthony, E. J.; Wildgust, N.; Aiken, T. Effects of impurities on CO_2 transport, injection and storage. *Energy Procedia* **2011**, *4*, 3071−3078. DOI: 10.1016/j.egypro.2011.02.219
(b) Element Energy Limited. *CO_2 pipeline infrastructure: an analysis of global challenges and opportunities*. Final Report for International Energy Agency, Greenhouse Gas Programme, 27-04-2010.
(c) Vandeginste, V.; Piessens, K. Pipeline design for a least-cost router application for CO_2 transport in the CO_2 sequestration cycle. *Int. J. Greenh. Gas Con.* **2008**, *2* (4), 571-581. DOI: 10.1016/j.ijggc.2008.02.001

Fig. S1. Experimental densities, ρ, for Mix 2 (CO_2 + 0.14 mol% NO + 0.09 mol% SO_2 + 0.17 mol% CO) versus pressure, p, at the nominal temperatures T.

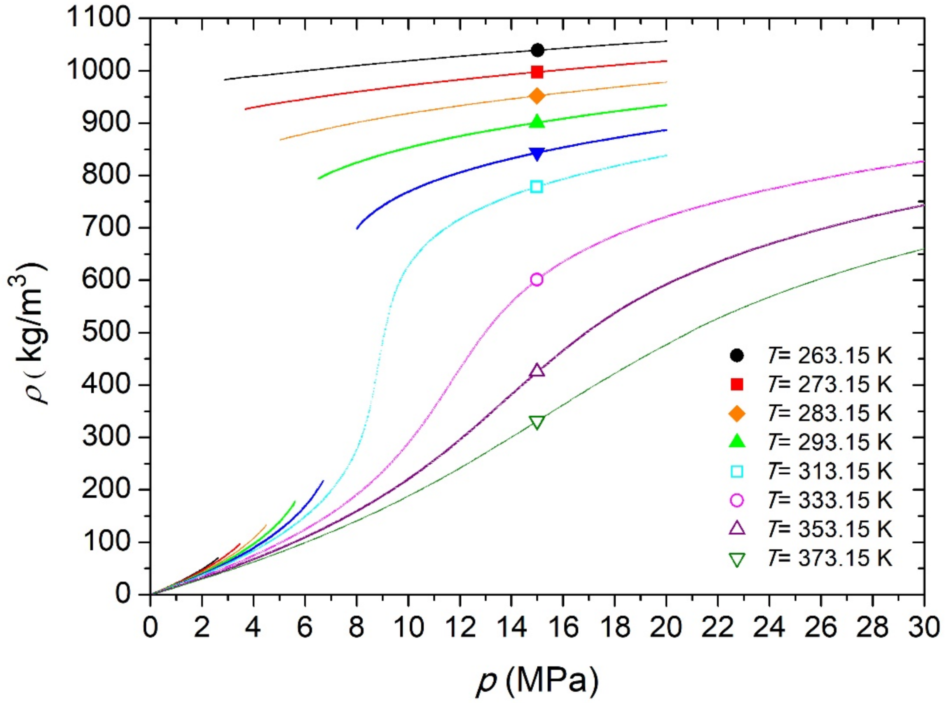

Fig. S2. Experimental densities, ρ, for Mix 1 (CO_2 + 3.00 mol% O_2 + 0.09 mol% SO_2 + 0.17 mol% CO) (a) and Mix 2 (CO_2 + 0.14 mol% NO + 0.09 mol% SO_2 + 0.17 mol% CO) (b), and for pure CO_2 versus pressure, p, at the nominal temperatures, T. (1) Whole studied range of pressures. (2) Gas phase region.

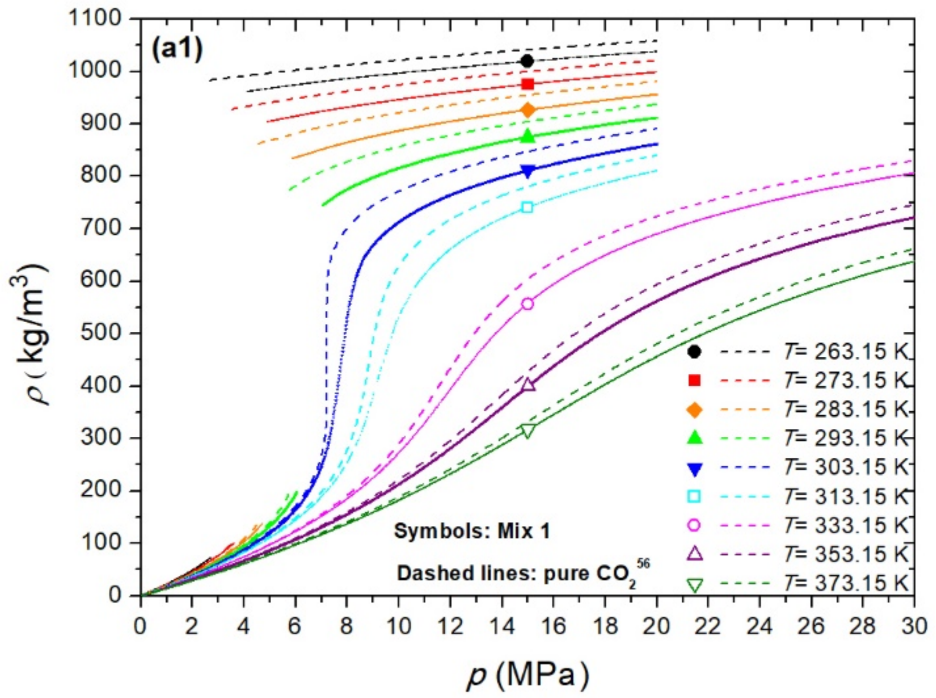

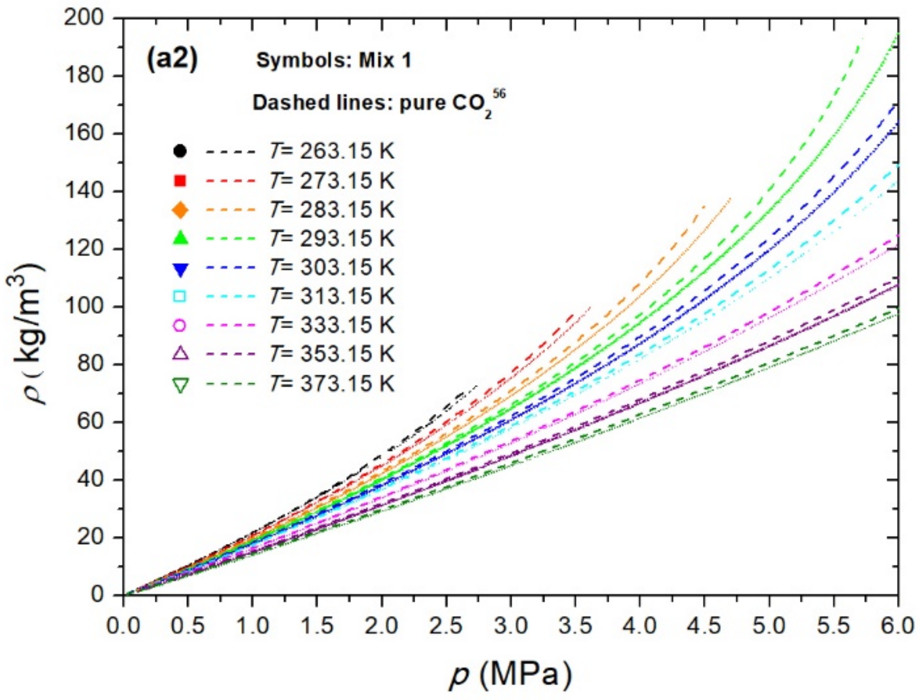

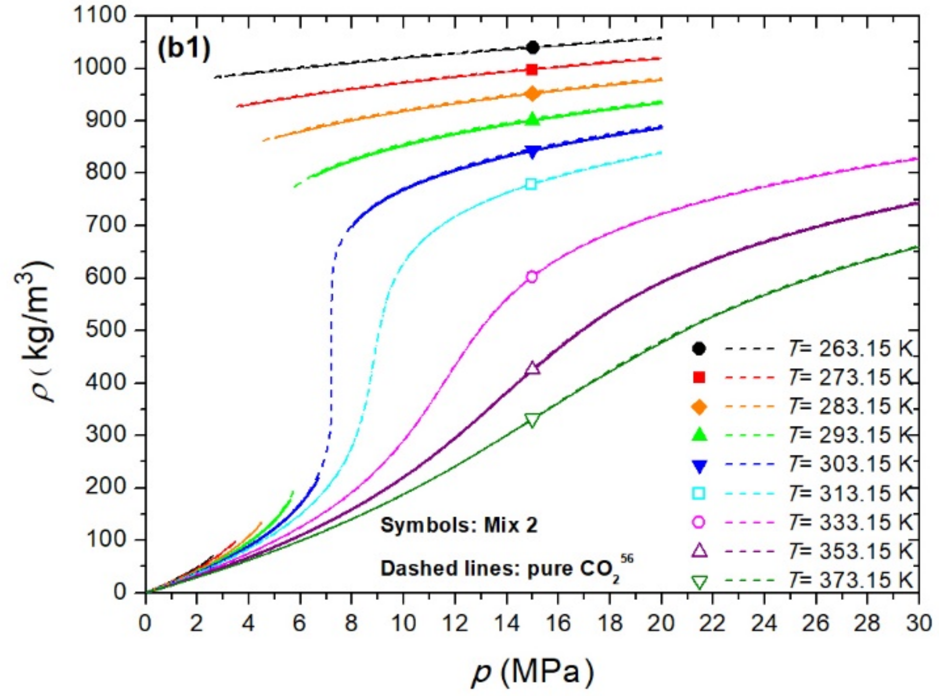
(b1)
Symbols: Mix 2
Dashed lines: pure CO_2[56]
T= 263.15 K
T= 273.15 K
T= 283.15 K
T= 293.15 K
T= 303.15 K
T= 313.15 K
T= 333.15 K
T= 353.15 K
T= 373.15 K
ρ (kg/m³)
p (MPa)

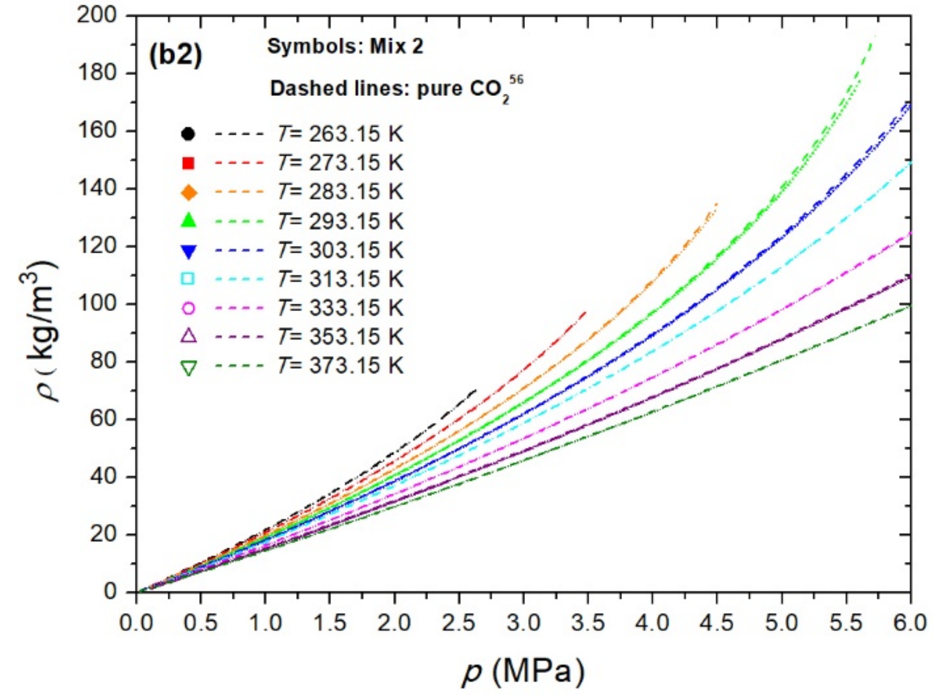
(b2)
Symbols: Mix 2
Dashed lines: pure CO_2[56]
T= 263.15 K
T= 273.15 K
T= 283.15 K
T= 293.15 K
T= 303.15 K
T= 313.15 K
T= 333.15 K
T= 353.15 K
T= 373.15 K
ρ (kg/m³)
p (MPa)

Fig. S3. Density versus temperature at selected pressures, *p*, for the mixtures Mix 1 (CO_2 + 3.00 mol% O_2 + 0.09 mol% SO_2 + 0.17 mol% CO) and Mix 2 (CO_2 + 0.14 mol% NO + 0.09 mol% SO_2 + 0.17 mol% CO), as well as for the mixtures CO_2 + 3.01 mol% O_2 , CO_2 + 3.00 mol% CO, CO_2 + 2.81 mol% CH_4, and pure CO_2.

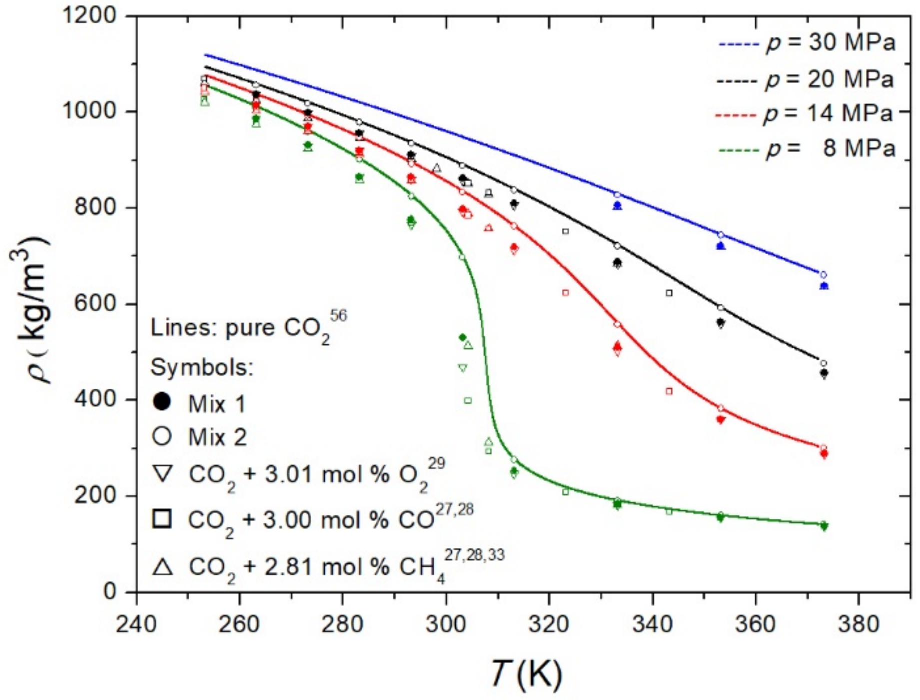

Fig. S4. Experimental and extrapolated speed of sound, c, for doped Mix 1 (CO_2 + 1.00 mol% CH_3OH + 2.97 mol% O_2 + 0.09 mol% SO_2 + 0.17 mol% CO) versus pressure, p, at the nominal temperatures T.

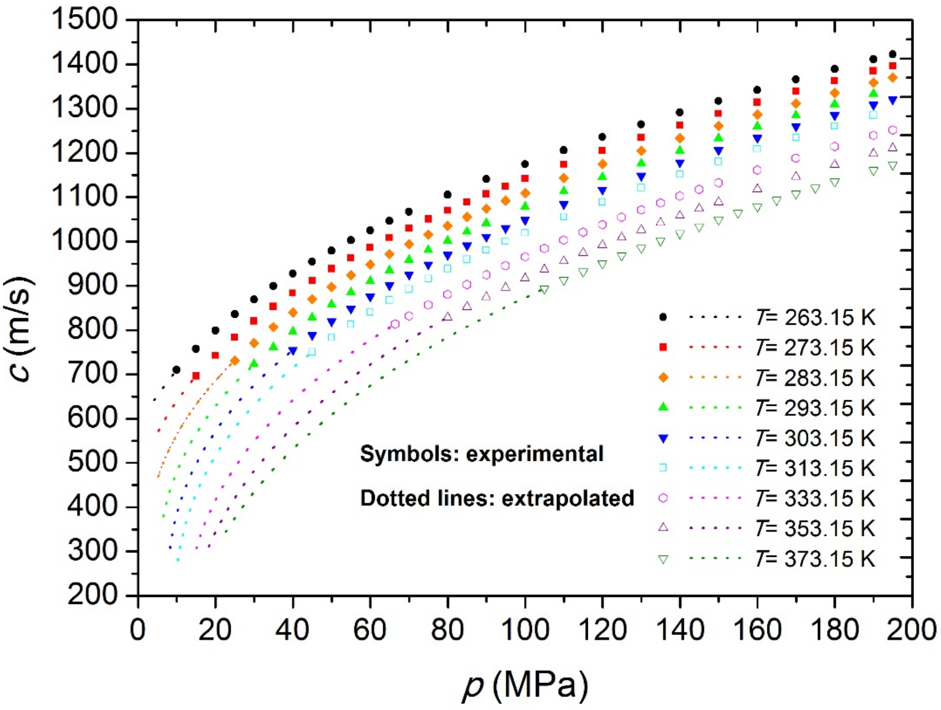

Fig. S5. Relative deviations between the experimental speeds of sound, c_{exp}, for doped Mix 1 (CO_2 + 1.00 mol% CH_3OH + 2.97 mol% O_2 + 0.09 mol% SO_2 + 0.17 mol% CO) and the values calculated for pure CO_2[56] at the nominal temperatures, T.

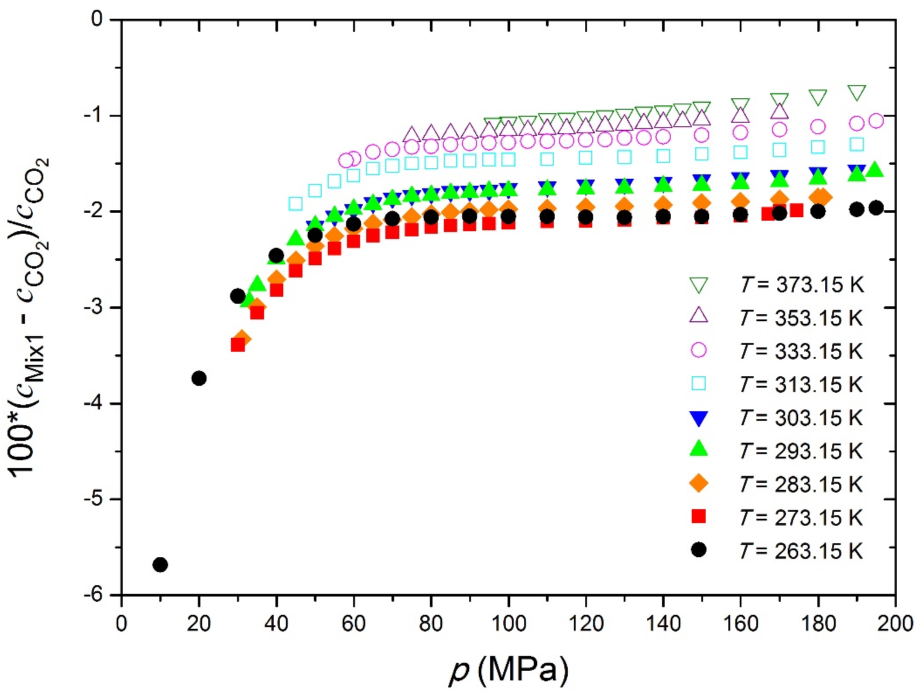

Fig. S6. Relative deviations between the experimental speeds of sound, c_{exp}, for doped Mix 2 (CO_2 + 1.02 mol% CH_3OH + 0.14 mol% NO + 0.09 mol% SO_2 + 0.17 mol% CO) and the values calculated for pure CO_2[56] at the nominal temperatures, T.

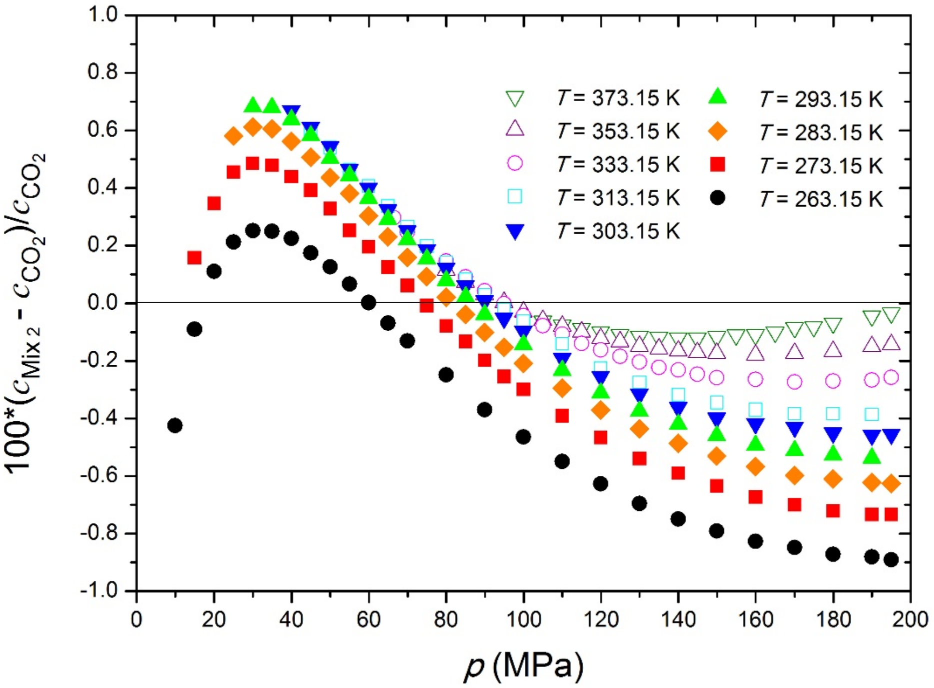

Fig. S7. Relative deviations between the experimental densities, ρ_{exp}, in this work and the values calculated from the evaluated EoS, ρ_{EoS}, for Mix 1 (CO_2 + 3.00 mol% O_2 + 0.09 mol% SO_2 + 0.17 mol% CO) (a) and Mix 2 (CO_2 + 0.14 mol% NO + 0.09 mol% SO_2 + 0.17 mol% CO) (b) at the nominal temperatures, *T*.

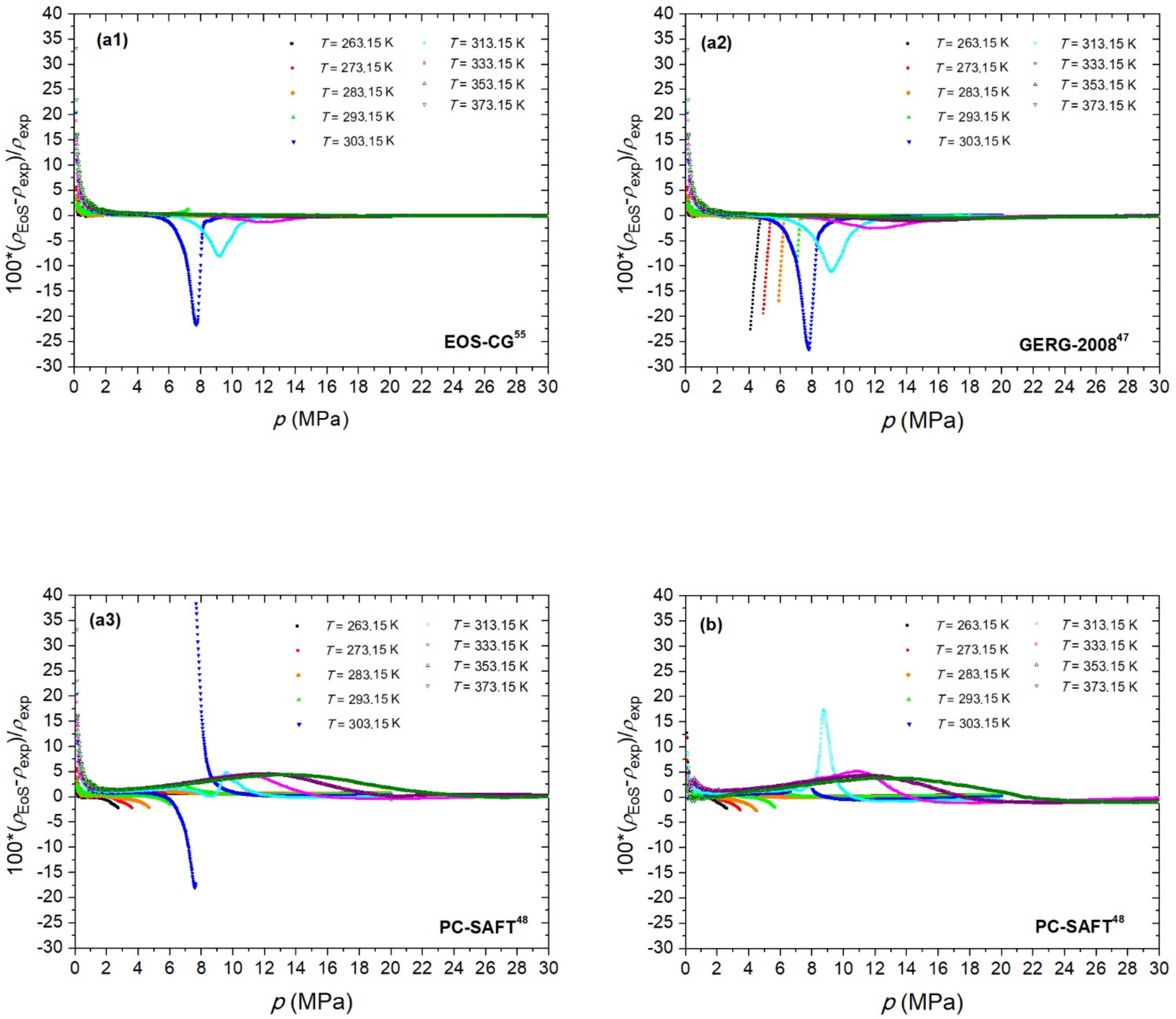

Fig. S8. Relative deviations between the experimental speed of sound, c_{exp}, in this work and the values calculated from the evaluated EoS, c_{EoS}, for doped Mix 1 (CO_2 + 1.00 mol% CH_3OH + 2.97 mol% O_2 + 0.09 mol% SO_2 + 0.17 mol% CO) (a) and doped Mix 2 (CO_2 + 1.02 mol% CH_3OH + 0.14 mol% NO + 0.09 mol% SO_2 + 0.17 mol% CO) (b) at the nominal temperatures, T.

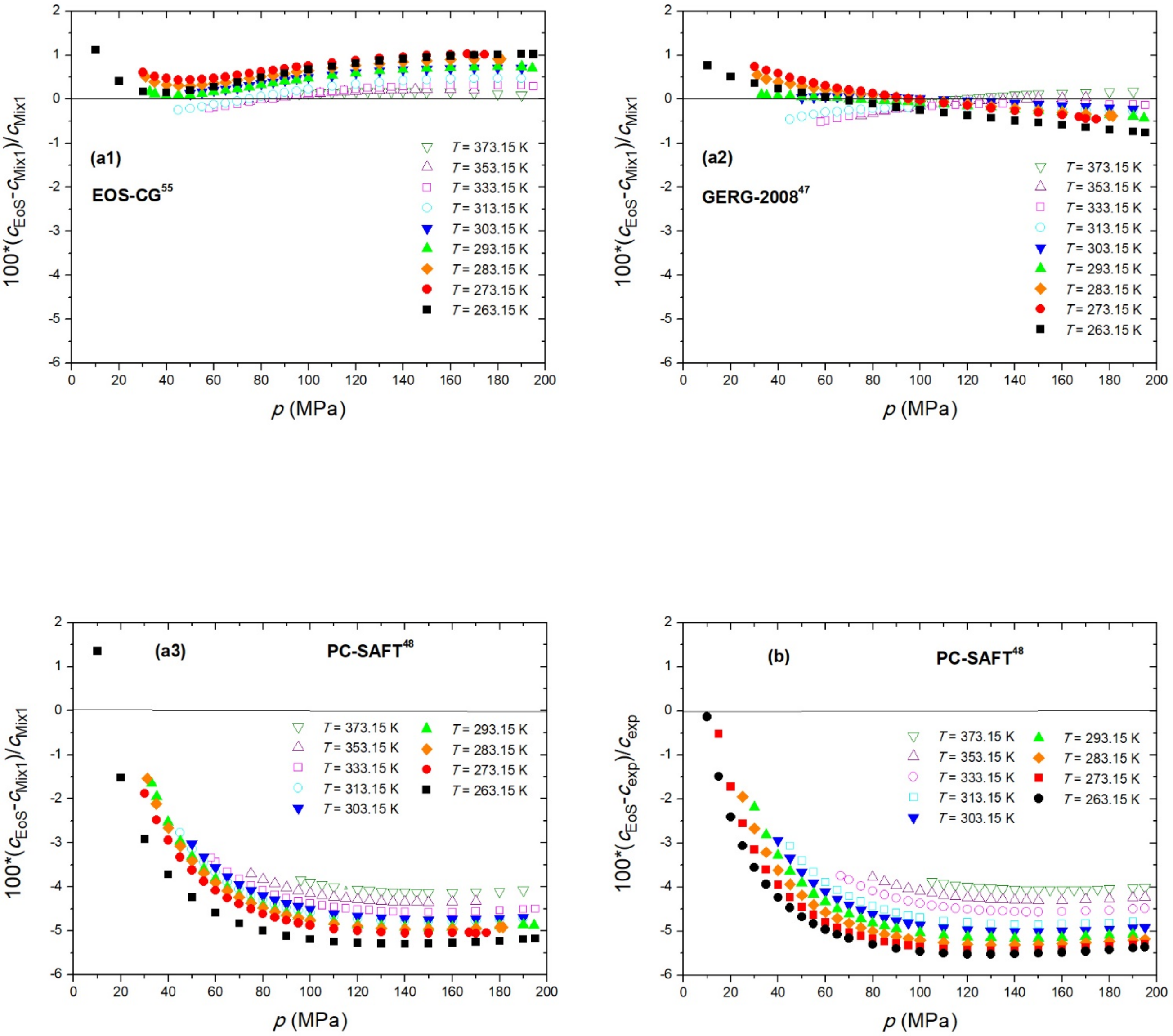

Fig. S9. Bubble pressure, p_{bubble}, versus temperature, T, for the mixtures Mix 1 (CO_2 + 3.00 mol% O_2 + 0.09 mol% SO_2 + 0.17 mol% CO) and Mix 2 (CO_2 + 0.14 mol% NO + 0.09 mol% SO_2 + 0.17 mol% CO), as well as for the mixtures CO_2 + 3.01 mol% O_2, CO_2 + 3.00 mol% CO, CO_2 + 2.81 mol% CH_4, and saturation pressure of pure CO_2.

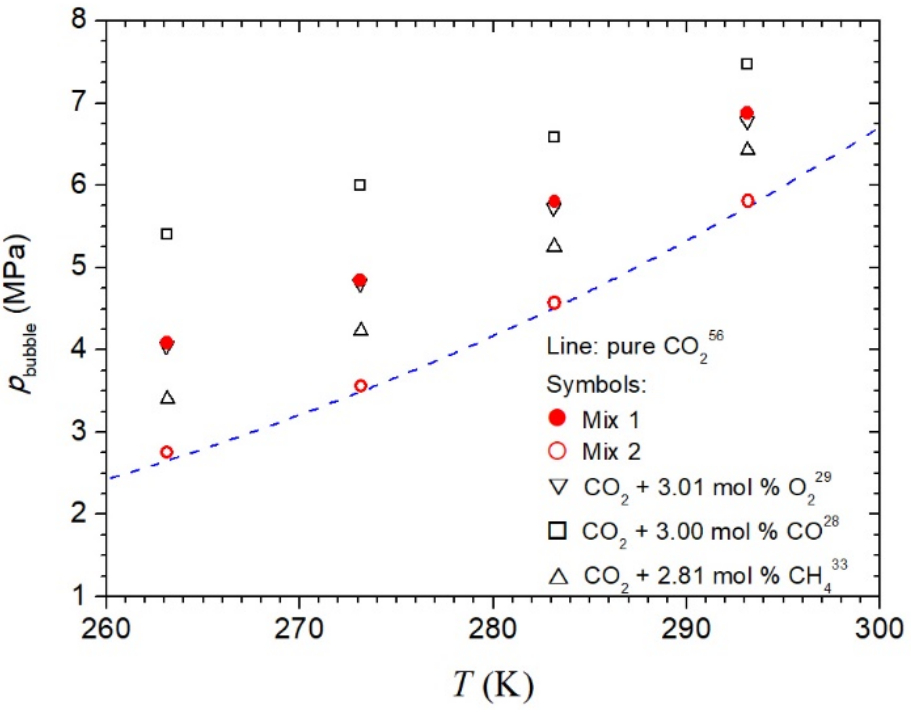

Fig. S10. Comparison of the pressure profile along the pipeline for Mix 1 (CO_2 + 3.00 mol% O_2 + 0.09 mol% SO_2 + 0.17 mol% CO) and for pure CO_2 at several transport temperatures T. A mass flow of m = 317.1 kg/s, an inner diameter of the pipeline of D = 0.508 m, and a roughness height of e = 4.6×10^{-5} m were used, along with a pipeline inlet pressure of 20.00 MPa.

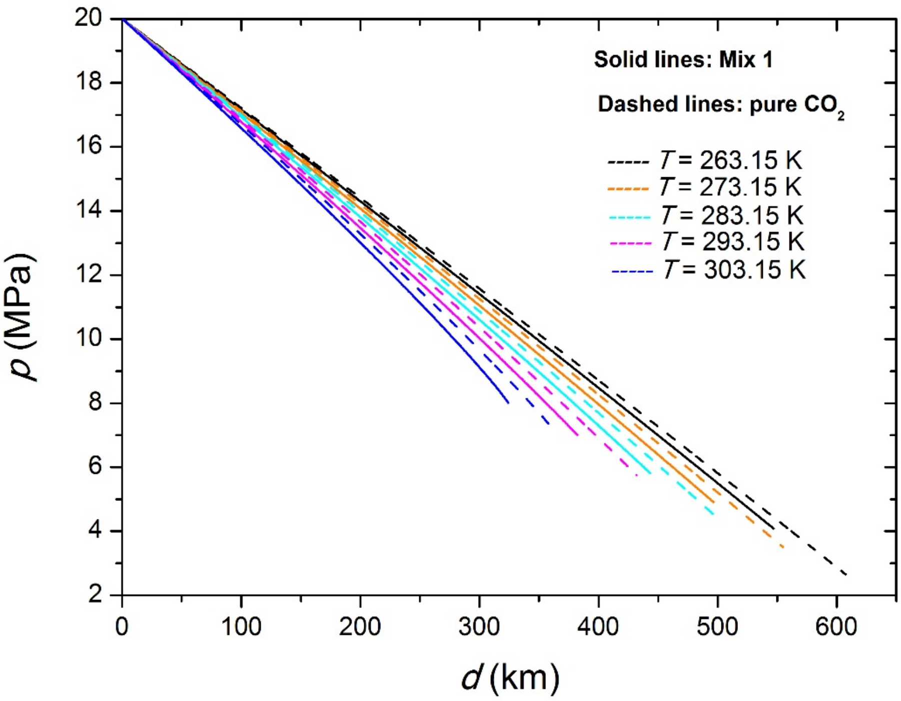

Fig. S11. Comparison of the density profile along the pipeline for Mix 1 (CO_2 + 3.00 mol% O_2 + 0.09 mol% SO_2 + 0.17 mol% CO) and for pure CO_2 at several transport temperatures T. A mass flow of m = 317.1 kg/s, an inner diameter of the pipeline of D = 0.508 m, and a roughness height of e = 4.6×10^{-5} m were used, along with a pipeline inlet pressure of 20.00 MPa

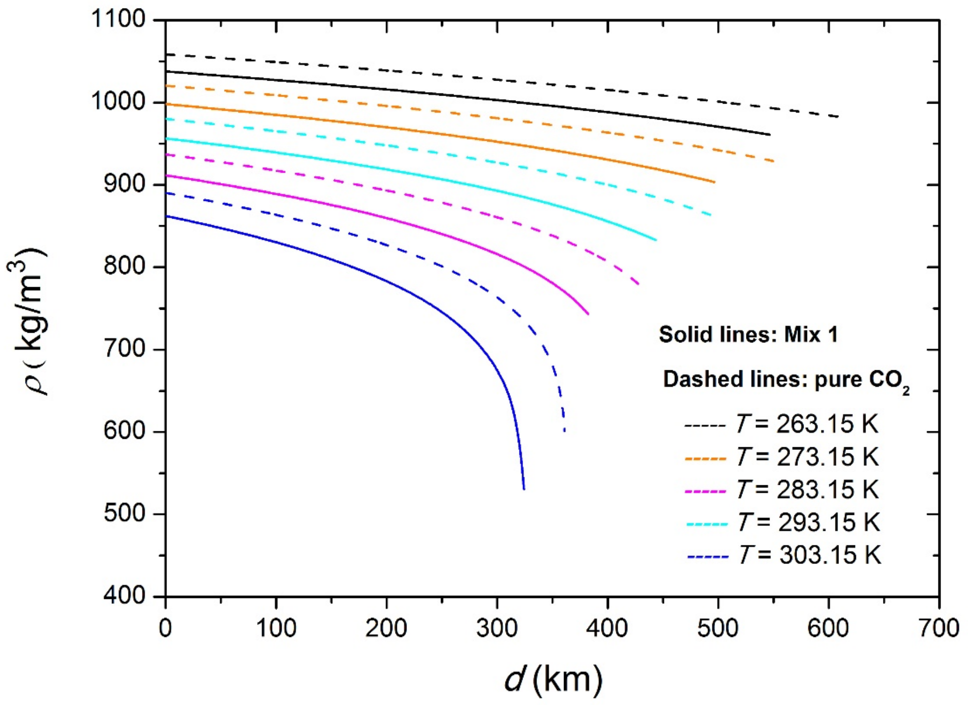

Fig. S12. Comparison of the pressure at 300 km from the pipeline inlet for Mix 1 (CO_2 + 3.00 mol% O_2 + 0.09 mol% SO_2 + 0.17 mol% CO), CO_2 + 3.01 mol% O_2 , CO_2 + 3.00 mol% CO, CO_2 + 2.81 mol% CH_4, and pure CO_2. A mass flow of m = 317.1 kg/s, an inner diameter of the pipeline of D = 0.508 m, and a roughness height of $e = 4.6\times10^{-5}$ m were used, along with a pipeline inlet pressure of 20.00 MPa.

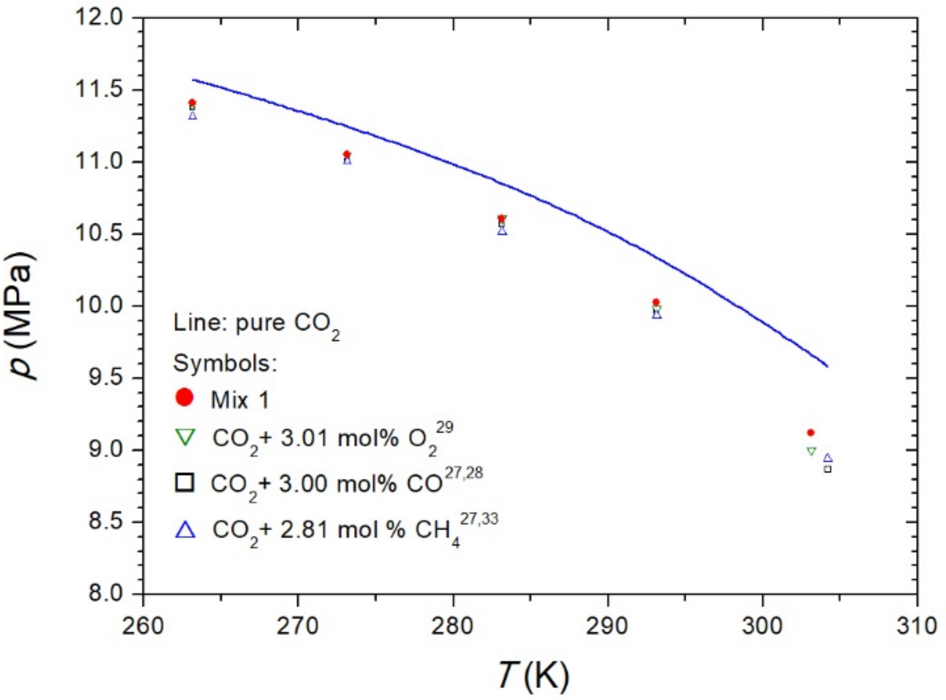

Fig. S13. Comparison of the density at 300 km from the pipeline inlet for Mix 1 (CO_2 + 3.00 mol% O_2 + 0.09 mol% SO_2 + 0.17 mol% CO), CO_2 + 3.01 mol% O_2 , CO_2 + 3.00 mol% CO, CO_2 + 2.81 mol% CH_4, and pure CO_2. A mass flow of m = 317.1 kg/s, an inner diameter of the pipeline of D = 0.508 m, and a roughness height of $e = 4.6\times10^{-5}$ m were used, along with a pipeline inlet pressure of 20.00 MPa.

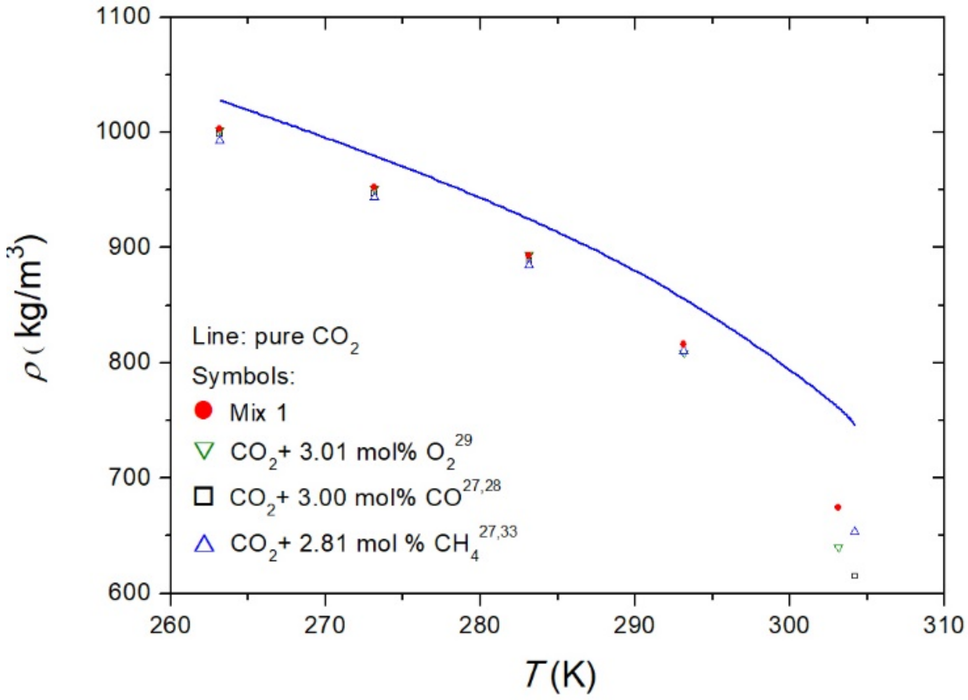

Fig. S14. Pipeline inner diameter, *D*, needed to transport a mass flow of fluid, *m*, of 317.1 kg/s for Mix 1 (CO_2 + 3.00 mol% O_2 + 0.09 mol% SO_2 + 0.17 mol% CO), CO_2 + 3.01 mol% O_2, CO_2 + 3.00 mol% CO, CO_2 + 2.81 mol% CH_4, and pure CO_2, as a function of the transport temperature, *T*, and at selected pressures, *p*. The roughness height was set at $e = 4.6\times10^{-5}$ m, and an average value for pressure drop per meter of 31.8 $Pa \cdot m^{-1}$ was used.

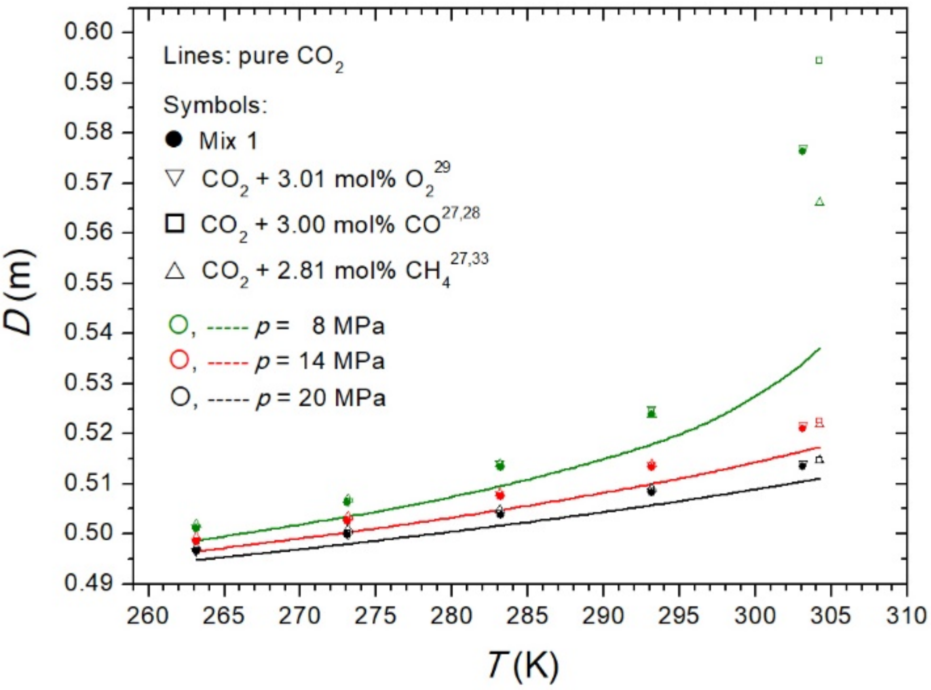

Fig. S15. Normalized storage capacity, M/M_0 for the studied mixtures versus pressure, p, at the nominal temperatures, T. (a) Mix 1 (CO_2 + 3.00 mol% O_2 + 0.09 mol% SO_2 + 0.17 mol% CO); (b) Mix 2 (CO_2 + 0.14 mol% NO + 0.09 mol% SO_2 + 0.17 mol% CO).

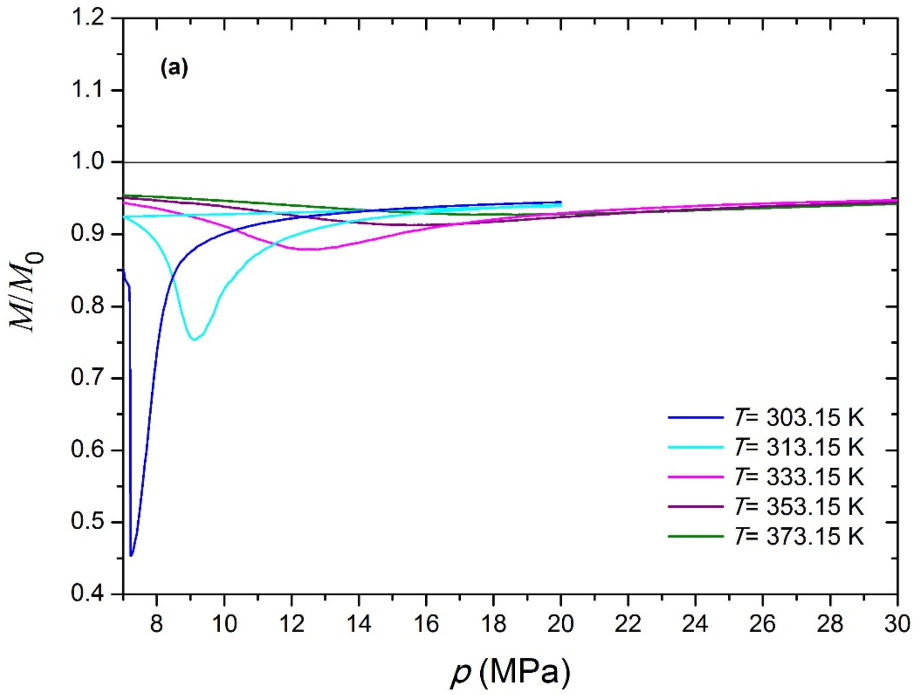

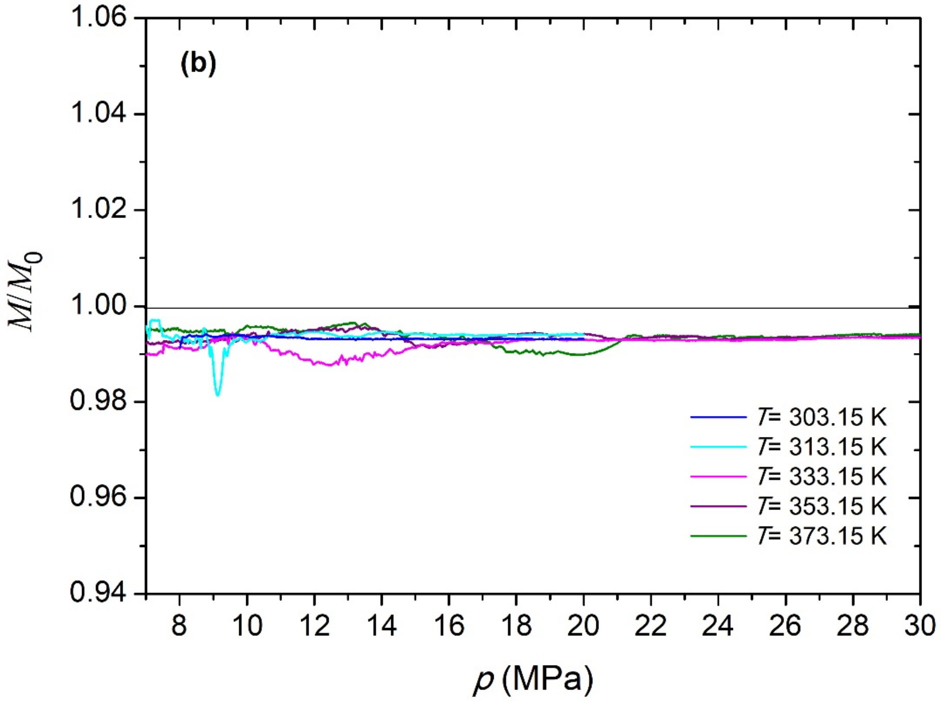

Fig. S16. Normalized rising velocity, v/v_0, in a concentrated (a) or dilute (b) saline aquifer, for Mix 1 (CO_2 + 3.00 mol% O_2 + 0.09 mol% SO_2 + 0.17 mol% CO) versus pressure, p, at the nominal temperatures, T. ρ_{br} is the density of the brine.

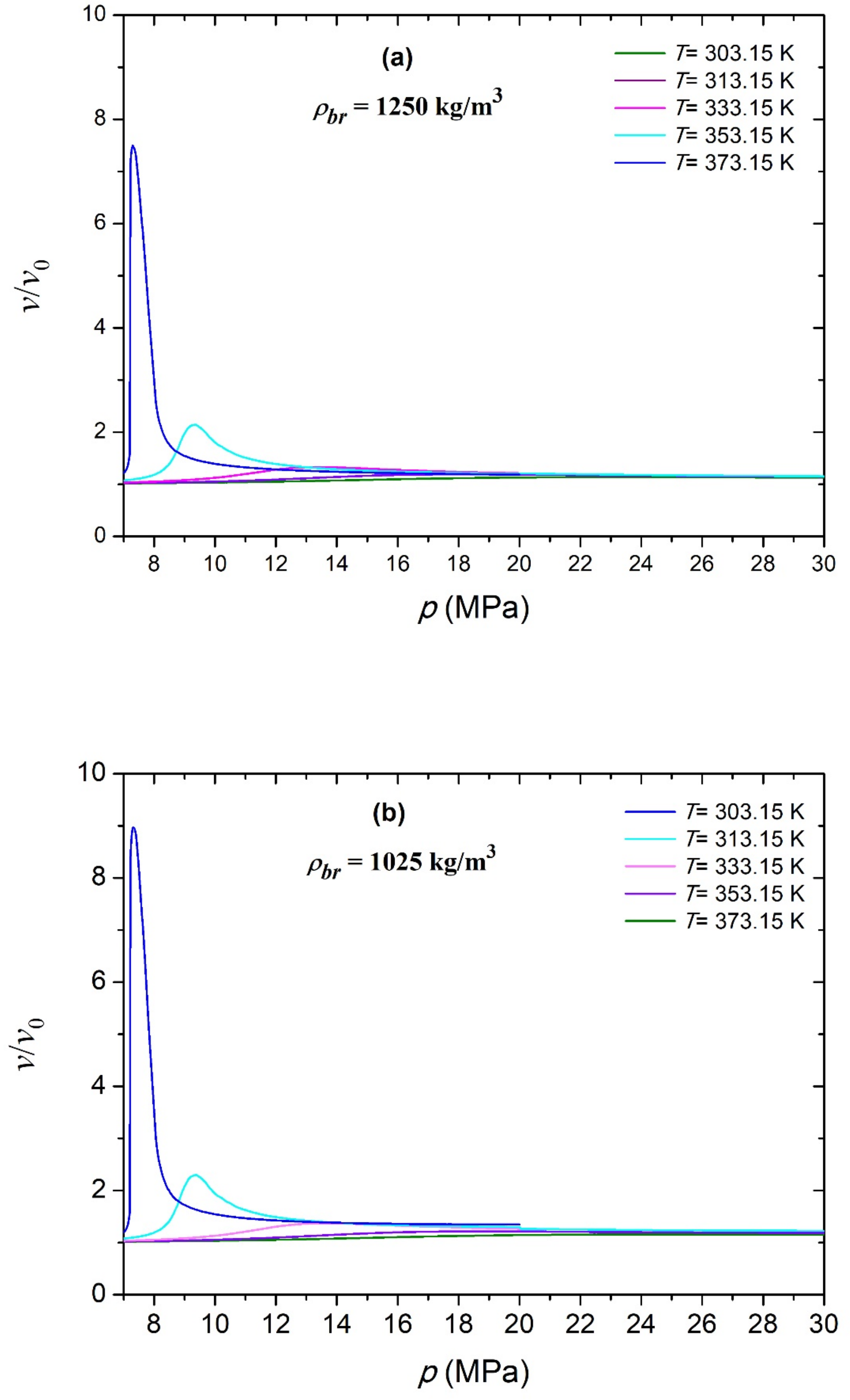

Fig. S17. Normalized permeation flux, $\dot{M}/\dot{M}_0$ for Mix 1 (CO_2 + 3.00 mol% O_2 + 0.09 mol% SO_2 + 0.17 mol% CO) versus pressure, p, at the nominal temperatures, T.

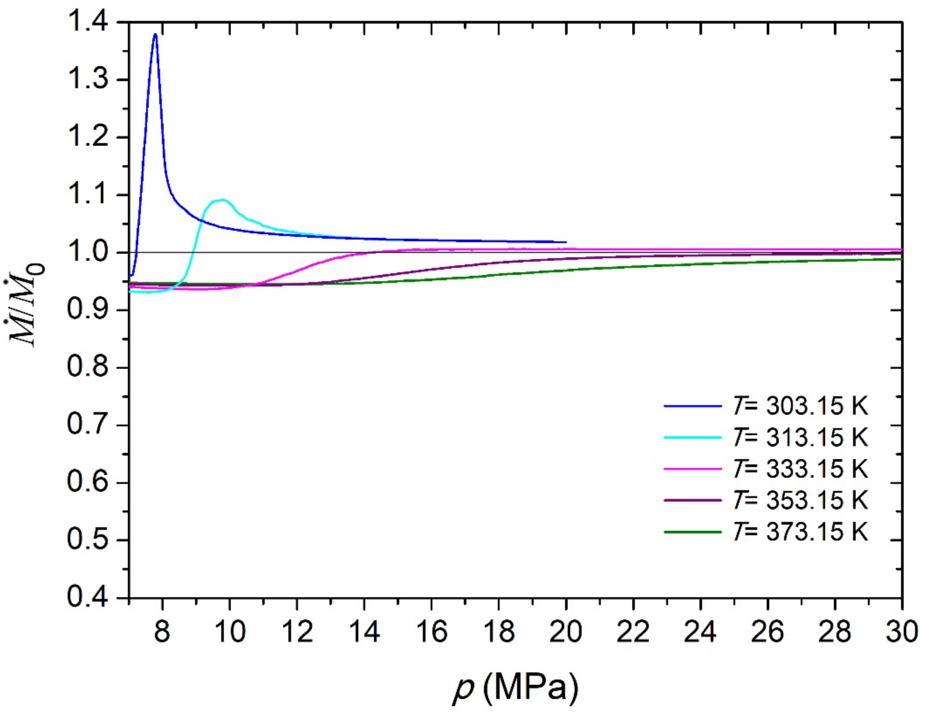

Fig. S18. Normalized permeation flux, $\dot{M}/\dot{M}_0$, for Mix 1 (CO_2 + 3.00 mol% O_2 + 0.09 mol% SO_2 + 0.17 mol% CO), CO_2 + 3.01 mol% O_2 , CO_2 + 3.00 mol% CO, and CO_2 + 2.81 mol% CH_4 under the reservoir conditions presented in Table 1.

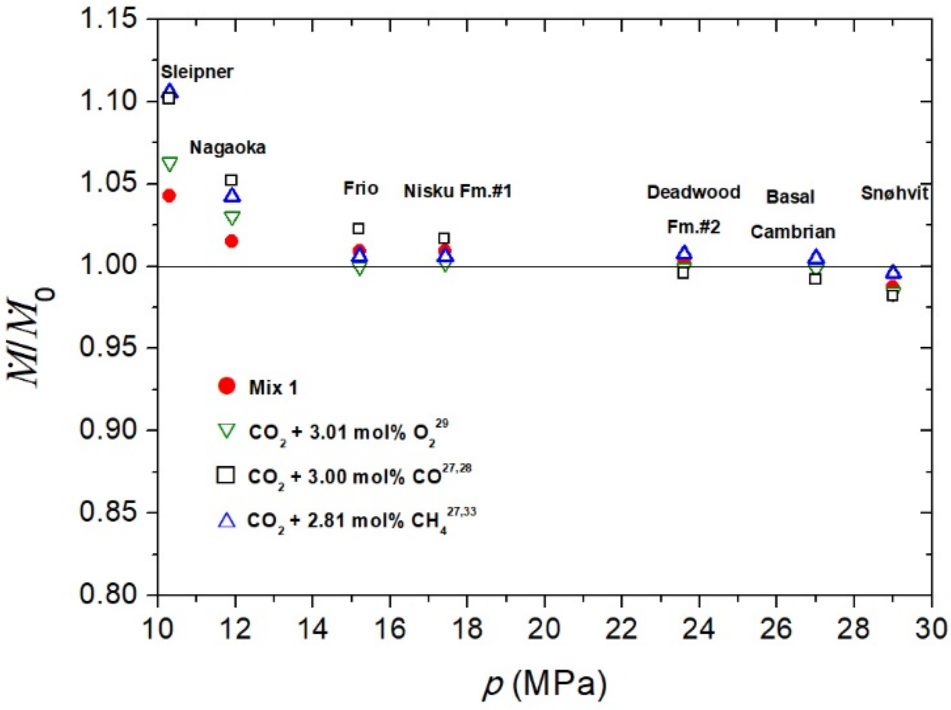

Supplement: Supplementary file 1 [file ef4c04818_si_001.pdf]
